# Supplementary material for: Genetic studies of paired metabolomes reveal enzymatic and transport processes at the interface of plasma and urine
Source: Nat Genet. 2023 Jun 5;55(6):995–1008. doi: 10.1038/s41588-023-01409-8 (PMC10260405; doi:10.1038/s41588-023-01409-8)
Supplement: Supplementary file 1 — Supplementary Results, Note, Methods and Figs. 1–3 [file 41588_2023_1409_MOESM1_ESM.pdf]

nature genetics

Article https://doi.org/10.1038/s41588-023-01409-8

Genetic studies of paired metabolomes reveal enzymatic and transport processes at the interface of plasma and urine

In the format provided by the authors and unedited

Table of Contents

SUPPLEMENTARY RESULTS **2**

COMPARISON OF PLASMA MQTLS TO OTHER GENETIC STUDIES OF THE CIRCULATING METABOLOME 2
COMPARISON OF URINE MQTLS TO GENETIC STUDIES OF THE CIRCULATING METABOLOME 3
INTERACTION OF GENETIC EFFECTS AT THE 1,299 MQTLS WITH SEX 3
DIFFERENCES IN EXPLAINED METABOLITE VARIANCE AND EFFECT DIRECTIONS OF INTER-MATRIX MQTLS 4
COLOCALIZATION OF PLASMA ANDROSTERONE SULFATE AND HYPERTENSION 5
METABOLITES MOST STRONGLY RELATED TO KIDNEY FUNCTION 6
INSIGHTS INTO KIDNEY-SPECIFIC PROCESSES THROUGH MQTLS FROM URINE-SPECIFIC METABOLITES 7
MQTL-RELATED GENES ARE ENRICHED IN TISSUES, CELL TYPES, PATHWAYS, AND MOUSE MODELS, REFLECTING CENTRAL METABOLIC FUNCTIONS 8

EXTENDED ACKNOWLEDGEMENTS **10**

SUPPLEMENTARY METHODS **11**

STUDY FOR REPLICATION: THE ARIC STUDY 11
CAUSAL GENE ASSIGNMENT 11
MENDELIAN RANDOMIZATION 12
TESTING MQTLS FOR POSITIVE SELECTION BY CALCULATING INTEGRATED HAPLOTYPE SCORE (IHS) 13
FUNCTIONAL GENOMICS FROM KIDNEY TISSUE AND TRANSCRIPTION FACTOR BINDING 14

SUPPLEMENTARY REFERENCES **17**

SUPPLEMENTARY FIGURE 1: GENETIC EFFECTS OF GCKD PLASMA MQTLS ON LEVELS OF THE CORRESPONDING METABOLITE IN PUBLISHED PLASMA/SERUM MGWAS. **19**

SUPPLEMENTARY FIGURE 2: GENETIC EFFECTS OF GCKD URINE MQTLS ON LEVELS OF THE CORRESPONDING METABOLITE IN PUBLISHED PLASMA/SERUM MGWAS. **20**

SUPPLEMENTARY FIGURE 3: COMPARISON OF GENETIC ASSOCIATIONS AT THE *DPEP1* LOCUS FOR ONE EXEMPLARY PLASMA METABOLITE, CYSTEINYLGLYCINE, AND ALL SEVEN DIGESTIVE PROTEINS IN PLASMA. **21**

SUPPLEMENTARY DATA 1: REGIONAL ASSOCIATION PLOTS FOR MQTLS IDENTIFIED IN MGWAS OF PLASMA METABOLITE LEVELS **22**

SUPPLEMENTARY DATA 2: REGIONAL ASSOCIATION PLOTS FOR MQTLS IDENTIFIED IN MGWAS OF URINE METABOLITE LEVELS **23**

Supplementary Results

Comparison of plasma mQTLs to other genetic studies of the circulating metabolome

The 1,296 GWAS of plasma metabolite levels identified 677 regions in 485 GWAS that contained at least one significantly associated SNP. A comparison of genetic effect sizes of the plasma mQTLs in each of these regions to their effects detected among 3,603 European ancestry (EA) and 818 African American (AA) participants of the ARIC study (**Supplementary Table 4**) showed strong correlations (Pearson coefficients 0.98 and 0.86, respectively), which remained almost unchanged when restricting to ARIC participants with normal kidney function (**Extended Data Figure 1**). These findings therefore suggested that genetic effects on plasma metabolite levels are generally comparable among individuals with and without reduced kidney function. Replication rates of plasma mQTLs detected in our study were 94% in the EA sample and 27% in the much smaller AA sample (Methods; **Supplementary Table 4**).

A comparison of index SNPs reported from seven large genetic studies of the plasma/serum metabolome[1-7] from EA participants that used the same technology for metabolite quantification to the findings from this study highlighted excellent correlation of genetic effects (median Spearman coefficient across the seven studies: 0.93; range 0.54-0.95) and high validation rates at different levels of statistical significance, as detailed for each study in **Supplementary Table 5**. For example, the median validation rate at a threshold of $p<0.05$/mQTLs detected in the respective study was 0.74 (range 0.31-0.98). Conversely, plasma mQTLs detected in this study showed excellent correlation of genetic effects with those from the published studies (median Spearman coefficient of 0.92), as shown together with high validation rates in **Supplementary Figure 1a-g**.

Comparison of urine mQTLs to genetic studies of the circulating metabolome

A comparison of the genetic effect sizes of the 622 significant urine mQTLs detected in our study with their respective counterparts from our own plasma mGWAS as well as the findings from seven published studies of the circulating metabolome[1-7] showed that the correlation of genetic effects was somewhat higher for our own plasma mGWAS as compared to the largest published studies (Spearman coefficient GCKD 0.81, range in published studies 0.19-0.77; median: 0.74). Validation rates of urine mQTLs in results from plasma mGWAS depended on significance level and statistical power, with much larger studies showing higher validation rates than GCKD at stringent significance levels, and similar rates at nominal significance (**Supplementary Figure 2**). A published plasma study of similar sample size (N=6,136)[5] as the GCKD plasma sample (N=5,023) showed lower validation rates of the urine mQTLs at each level of significance, which may highlight the value of studying paired metabolomes from the same study.

Interaction of genetic effects at the 1,299 mQTLs with sex

Interactions between the index SNP at each mQTL and sex (Methods) yielded 37 significant ($p<3.8E-05$) interactions after correction for multiple testing. The SNPs that showed the strongest differences by sex were consistent with the literature: for example, variants at the *CPS1* locus showed a stronger effect on the levels of plasma glycine[8,9], and also other associated plasma and urine metabolites, in women compared to men. When an mQTL for a given metabolite was detected in both plasma and urine at the *CPS1* locus, most significant sex differences detected in plasma translated to urine. A stronger genetic effect of variants at the *SULT2A1* locus on the plasma levels of androgen metabolites in men as compared to women is consistent with the gene's function in catalyzing dehydroepiandrosterone sulfation

in the adrenal cortex, and could be explained by higher levels of these metabolites in men compared to women. The significantly (p-interaction=8E-11) larger effect of the index SNP at the *SLC28A2* locus on urine adenosine levels in men as compared to women has not been reported previously. The encoded protein operates as a nucleoside transporter in the apical membrane of kidney epithelial cells where it mediates nucleoside reabsorption, including adenosine.[10] GTEx data show higher median expression levels of *SLC28A2* in men as compared to women, which may explain the observed differences.

Differences in explained metabolite variance and effect directions of inter-matrix mQTLs

The explained variance in metabolite levels for shared underlying genetic variants that were implicated by inter-matrix mQTLs was often larger in urine than in plasma. Differences of >20% were observed for mQTLs that reflect the function of known detoxification enzymes such as the ones encoded by *AKR7A2*, *NAT8*, and *UGT2B11*. These enzymes are highly expressed in both hepatocytes and tubular epithelial cells,[11] and the urine levels of their associated metabolites may reflect the cumulative detoxification function of liver and kidney with concentration of the metabolites in urine.

The direction and strength of association of almost all 204 “inter-matrix” mQTLs was nearly identical in plasma and urine (**Extended Data Figure 4**). The only exception was observed at *SLC7A9*, where the same index SNP allele was associated with higher urine and lower plasma levels of homocitrulline and X – 24736. This observation may be explained by the encoded protein’s role as a re-uptake transporter of dibasic amino acids such as lysine, cystine, and ornithine at the apical membrane of proximal tubular cells.[12] Less efficient transport activity could result in higher urine and lower plasma levels of potential substrates,

for which SLC7A9 is the major reuptake transporter and for which there is no compensatory generation or uptake into plasma.

Colocalization of plasma androsterone sulfate and hypertension

An interesting example of colocalization between an mQTL and a disease was the association with the largest absolute effect: the minor allele at a low-frequency (MAF 3%) variant in *CYP3A7*, rs45446698, was associated with lower plasma levels of its substrate androsterone sulfate (P-value=2.4×10^{-149}, effect=-2.15; **Supplementary Table 3**), as well as with other androgenic steroids in both plasma and urine. Colocalization supported a shared, positive relationship between androsterone sulfate levels and hypertension as well as other cardiometabolic traits (**Supplementary Table 13**). Investigation of potential sex-specific effects using individual-level data of unrelated participants of European ancestry in the UK Biobank (N=337,111) showed a stronger and nominally significant association of the minor G allele at rs45446698 with lower systolic and diastolic blood pressure as well as lower chance of hypertension in men as compared to women (**Table**). For diastolic blood pressure, a significant sex-specific effect modification was observed (P-value=5.1×10^{-3}). Our data suggest that these sex-specific differences may partly relate to differences in the levels of androgenic steroids.

Table: Effect estimates of rs45446698 on blood pressure traits and hypertension in UK Biobank

**Trait**	**Men**	**Women**	**Interaction**
	effect or OR (95%CI)[1]		P-value[2]
**Systolic blood pressure**	**-0.32 (-0.62;-0.02)**	-0.10 (-0.39;0.19)	4.2E-01
**Diastolic blood pressure**	**-0.20 (-0.38;-0.02)**	**0.17 (0.01;0.34)**	**5.1E-03**
**Hypertension**	**0.94 (0.90;0.97)**	0.96 (0.93;1.00)	3.4E-01

[1] effect estimate for continuous blood pressure traits and odds ratio (OR) for binary hypertension trait, together with their respective 95% confidence intervals (CI); [2] P-value for the association test of interaction between trait and sex; bold: nominally significant (p<0.05) association.

Metabolites most strongly related to kidney function

We performed three complementary analyses to examine which metabolites were most strongly related to kidney function. First, we inferred, for each plasma and urine metabolite, the proportion of metabolite variance explained by eGFR based on linear models in the GCKD study. The **Extended Data Figure 6** shows results for all investigated metabolites, ordered by the maximum of explained variance across plasma and urine. Consistent with expectations, the metabolite for which the eGFR explained the largest proportion of variance was plasma creatinine.

Second, we investigated the relationship between 424 index SNPs of a large GWAS meta-analysis of eGFR[13] with the mQTLs detected in our study, where 414 of these index SNPs were present. We identified 25 and 27 eGFR index SNPs that were significantly associated with metabolite levels in plasma (P-value <0.05/(424*1296)) and urine (P-value <0.05/(424*1401)), respectively. When focused on mQTLs with support for colocalization with eGFR, *CPS1*, *NAT8*, and *SLC6A13* were the loci at which genetic associations were shared with eGFR and at least three metabolites.

Third, we performed MR analyses at loci implicated by positive colocalizations with eGFRcrea, eGFRcys, creatinine, or cystatin C shown in **Supplementary Table 13**. Statistical support for altered kidney function (exposure) as a cause of altered metabolite levels

(outcome) was assessed. We concentrated on this direction because of the abundance of independent genetic instruments for eGFR, which enables checks of MR assumptions as well as sensitivity analyses, which is not the case for most metabolites. After thorough filtering of genetic instruments for potential pleiotropy (Methods), we identified 11 findings with significant support of altered kidney function causing a change in metabolite levels (**Supplementary Table 14**). Many of the implicated metabolites are related to the function of a detoxification enzyme almost exclusively expressed in the kidney, NAT8. It is conceivable that altered kidney function may lead to changes in the levels of substrates or products of a central renal enzyme. As expected, better kidney function was related to higher levels of all NAT8 products in plasma and urine.

Insights into kidney-specific processes through mQTLs from urine-specific metabolites

There were multiple examples of associations between variants in genes encoding transport proteins at the apical membrane of tubular cells and urine levels of the metabolites that they reabsorb from the ultrafiltrate, such as *SLC36A2* and glycine, *SLC5A9* and mannose, as well as *SLC28A1* and *SLC28A2* and several nucleosides. At the *SLC36A2* locus, fine-mapping resulted in the identification of a missense variant (p.Gly87Val, NP_861441.2) that has been reported as a cause of autosomal-dominant hyperglycinuria (MIM #138500).[14] This suggests the presence of persons with this condition in our study population, highlighting opportunities to identify causative alleles for autosomal-dominant monogenic conditions based on semi-quantitative urine metabolomics studies.

mQTL-related genes are enriched in tissues, cell types, pathways, and mouse models, reflecting central metabolic functions

The 282 prioritized genes across all mQTLs were significantly over-represented among a large number of Gene Ontology (GO) terms and KEGG pathways, as were the genes implicated by plasma or urine mQTLs separately (Methods; **Supplementary Table 17**). In general, the odds ratios of enriched terms detected from plasma mQTLs-related genes were of similar magnitude to those from urine mQTL-related genes (**Extended Data Figure 6a**). This indicates that, although some of the pathway-assigned genes may only be associated with metabolite levels in plasma or in urine, the two matrices capture a lot of shared information about metabolism-relevant pathways. When focusing on genes related to matrix-specific mQTLs, lipid metabolism-associated terms showed strong enrichment for plasma mQTL-specific genes, whereas terms related to carbohydrate, nucleoside, and catecholamine metabolism showed strong enrichment for urine mQTL-specific genes (**Extended Data Figure 6b**).

The genes implicated by either plasma or urine mQTLs were highly expressed in the same human tissues (Methods): liver, kidney cortex and medulla, pancreas, small intestine, heart, adrenal gland, and colon (**Extended Data Figure 6c**; **Supplementary Table 18**). Enrichment at the cellular level using scRNA-seq data from kidney, liver, and intestine as well as 11 organs from the Human Protein Atlas was observed for cells of the proximal tubule, hepatocytes, and enterocytes (**Extended Data Figure 6d**; **Supplementary Table 19**). In summary, mQTL-related genes were highly expressed in specific tissues and cell-types, most strongly in kidney and liver, confirming that plasma and urine metabolites are important readouts of central functions of these two organs.

Lastly, we tested whether the 282 prioritized genes were enriched among genes that, when manipulated, cause abnormal homeostasis (MP:0001764) or its sub-terms in mice (**Supplementary Tables 20**; Methods). The most significantly enriched mouse phenotypes

pointed towards abnormal levels of amino acids, dicarboxylic acids and lipids in plasma and/or urine (**Figure 4c**). The availability of a mouse model with a metabolism-related phenotype offers opportunities for targeted experimental follow-up to illuminate the function of the respective gene in the generation or transport of the implicated metabolite.

Extended Acknowledgements

List of GCKD Study Investigators

A list of nephrologists currently collaborating with the GCKD study is available at http://www.gckd.org.

University of Erlangen-Nürnberg	Kai-Uwe Eckardt, Heike Meiselbach, Markus Schneider, Mario Schiffer, Hans-Ulrich Prokosch, Barbara Bärthlein, Andreas Beck, André Reis, Arif B. Ekici, Susanne Becker, Ulrike Alberth-Schmidt, Anke Weigel, Sabine Marschall, Eugenia Schefler
University of Freiburg	Gerd Walz, Anna Köttgen, Ulla Schultheiß, Fruzsina Kotsis, Simone Meder, Erna Mitsch, Ursula Reinhard
RWTH Aachen University	Jürgen Floege, Turgay Saritas, Alice Groß
Charité, University Medicine Berlin	Elke Schaeffner, Seema Baid-Agrawal, Kerstin Theisen
Hannover Medical School	Hermann Haller
University of Heidelberg	Martin Zeier, Claudia Sommerer Mehtap Aykac
University of Jena	Gunter Wolf, Martin Busch, Rainer Paul
Ludwig-Maximilians University of München	Thomas Sitter
University of Würzburg	Christoph Wanner, Vera Krane, Antje Börner-Klein, Britta Bauer
Medical University of Innsbruck, Division of Genetic Epidemiology	Florian Kronenberg, Julia Raschenberger, Barbara Kollerits, Lukas Forer, Sebastian Schönherr, Hansi Weissensteiner
University of Regensburg, Institute of Functional Genomics	Peter Oefner, Wolfram Gronwald
Department of Medical Biometry, Informatics and Epidemiology (IMBIE), University of Bonn	Matthias Schmid, Jennifer Nadal

Supplementary Methods

Study for replication: The ARIC study

Replication of plasma metabolites was tested in the ARIC study, a prospective community-based cohort of 15,792 individuals enrolled between 1987-1989 from four U.S. communities[15]. Blood samples for the measurement of serum metabolite levels were collected at the fifth study visit (2011-2013). Institutional review boards at each of the four field centers approved of the study, and written informed consent was obtained from participants at baseline and follow-up visits. Participants of European ancestry and African American participants with available genome-wide genotypes and metabolomic profiling at visit 5 were included (N=3,603 and 818, respectively).

Causal gene assignment

Prioritized genes assigned by the automated workflow (see main text) were manually reviewed for biological plausibility and for consistency across colocalizing mQTLs and matrices. First, in cases where the prioritized gene could not be connected to the implicated metabolite through review of PubMed (https://pubmed.ncbi.nlm.nih.gov/), OMIM (https://www.omim.org/), or the Human Metabolome Database (https://hmdb.ca/), other genes in the locus with a lower number of evidence scores assigned by the automated algorithm were reviewed. In case one of these genes was plausibly linked to the implicated metabolite through a known inborn error of metabolism or experimental evidence, the causal gene was reassigned. Second, in few instances where the same index SNP was associated with different metabolites and a different most likely causal gene had been automatically assigned, the same causal gene was manually reassigned when there was a clear biological fit of one of the automatically assigned genes to all of the metabolites and the other assigned gene could

not also plausibly be connected to its associated metabolite(s). Unnamed metabolites were evaluated for correlation with named metabolites as described in Schlosser *et al*[16,17] and treated as their named counterparts when they mapped into the same eigenmetabolite. In instances where the index SNP differed, only highly correlated index SNPs (r^2>0.5, D'>0.8) were evaluated for manual reassignment. Lastly, the most likely causal gene assigned to a given metabolite was reviewed across matrices. When the automated assignment differed and there was high LD (see above) between the index SNPs, the gene with higher biological plausibility across plasma and urine was assigned, taken biological plausibility of eigenmetabolite members into consideration in case of unnamed metabolites.

Mendelian Randomization

Two-sample Mendelian randomization analysis (MR)[18] was performed using the R package MendelianRandomization (v0.6.0).[19] To examine the relationship of kidney function (exposure) as a cause of altered levels of metabolites with a positive colocalization with eGFR (outcome; 136 plasma and 96 urinary metabolites in **Supplementary Table 13**), we used GWAS summary statistics for creatinine-based eGFR for participants of European ancestry reported by Stanzick, et al. [13]. Genetic instruments were 869 SNPs with reported association P-value $<5\times10^{-8}$ that were independent ($r^2 \leq 0.2$) as identified using Plink software v1.9[20] and reference data from 1000 Genomes (phase 3).[21]

For all MR analyses, genetic instruments were investigated in the PhenoScanner database[22] to identify the potential for horizontal pleiotropy introduced by associations with other traits. All genetic instrument with potentially pleiotropic signals were excluded. As the main method to estimate causal effects, the inverse-variance weighted method was chosen and, if at least three genetic instruments were available in the respective analysis, the

weighted median method for sensitivity analysis. Per analysis and matrix, the significance threshold was defined as P-value <0.05/# evaluated metabolites.

Testing mQTLs for positive selection by calculating integrated haplotype score (iHS)

The iHS at all index or their proxy SNPs was calculated to investigate whether genetic variants associated with metabolites have experienced selective pressure and showed signals for positive selection. The iHS compares the extension of haplotype homozygosity around the ancestral and derived allele at a SNP of interest[23], for which we used 1,417,184 genotyped variants of 5,034 individuals of the GCKD study phased with SHAPEIT (version 2 r790). The ancestral allele state was assigned to each genotyped SNP based on the ancestral allele information downloaded from Ensembl, ftp://ftp.ensembl.org/pub/release-71/fasta/ancestral_alleles/homo_sapiens_ancestor_GRCh37_e71.tar.bz2. The ancestral allele could be assigned for 1,313,870 of the genotyped SNPs (92.7%) based on high probability in the assignment procedure of Ensembl. The mapping of genetic position needed for the iHS calculation was based on the genetic map of the 1000 Genome Project Phase 3 downloaded from https://mathgen.stats.ox.ac.uk/impute/1000GP_Phase3.html. To each SNP the genetic position of the reference SNP with minimal physical distance and with smaller or equal physical position was assigned. For the index SNPs of the mQTLs that were not present in the phased genotype data, we determined proxy SNPs based on maximal LD and minimal distance using plink (version 1.90 beta6.20). Selscan version v1.3.0[24] was used for genome-wide iHS calculation. For every SNP with MAF >0.1, the iHS was calculated based on the genetic position using the default parameters except for the additional stopping condition for the EHH decay curve based on physical distance, so that the EHH decay curve was only truncated for integration when the EHH decay cutoff of 0.05 was reached regardless of the physical distance

to the tested SNP. Sites with low MAF were kept to construct haplotypes. The genome-wide standardization of the iHS was conducted based on frequency bins of the derived allele with a step size of 0.05 resulting in 16 frequency bins. Since the genome-wide iHS follows approximately a standard normal distribution, we considered the 0.025-quantile and the 0.975-quantile of the standard normal distribution as critical values and reported all mQTLs with |iHS|>1.96 as candidates for targets of positive selection. The extended haplotype homozygosity (EHH)[25] was plotted using the R package ´rehh´[26] to visualize the extension of haplotype homozygosity around the ancestral and derived allele at SNPs with extreme iHS.

Functional genomics from kidney tissue and transcription factor binding

Kidney samples were obtained from macroscopically dissected cortex and medulla of tumor-adjacent normal tissue in nephrectomy specimens from three donors and has been described previously.[27] Briefly, RNA extraction and RNA-seq was performed by GeneWiz. Trimming and alignment of paired-end fastq files to human reference genome sequence hg38 was done with STAR 2.7.5b[28] with parameters --outFilterIntronMotifs RemoveNoncanonical --outFilterMismatchNoverReadLmax 0.04. Counting of the number of reads aligned to each exon (feature) was performed using *featureCounts*[29]. Visualization of read-normalized density tracks (.bw) was done with pyGenomeTracks version 3.7.[30]

ATAC-seq was carried out on snap-frozen human kidney samples by ActiveMotif and aligned to the hg38 reference genome (BWA default settings). Normalization of read depth was carried out by random down sampling to the sample with lowest coverage. Peaks were called using MACS 2.1.0 at a cutoff of q-value 0.01, without control file, and with the -nomodel option. We removed false ChIP-Seq peaks contained in the ENCODE blacklist during peak filtering.

Kidney-expressed transcription factors (TFs) were identified by screening all 675 TFs in the JASPAR 2020 core vertebrate motif database[31] for expression in our RNA-seq dataset from primary human kidney. TFs were considered to be expressed if they showed five unnormalized counts in at least two of our six kidney samples (ignoring tissue subtype cortex or medulla). For the 517 kidney-expressed TFs, we performed a motif search at the position of rs6124828 using the position weight matrices provided by JASPAR and the R package motifmatchR (p.cutoff = 5x10e-5)[32]. The motif search sequence lengths were adapted for each motif, thereby guaranteeing that each matching motif overlapped the SNP. Differential transcription factor binding P-values were computed with the online tool sTRAP.[33] The following sequences in fasta format were submitted: "TAGCCTTGTTTTAGGTCTTAGAAGCTGATCATTAACCAATTCCTGCTCCTC" (major allele) and "TAGCCTTGTTTTAGGTCTTAGAAGCAGATCATTAACCAATTCCTGCTCCTC" (minor allele) using the JASPAR vertebrates matrix files and human promoters as background. Subsequently, the results for HNF1A and HNF1B were extracted.

For the kidney-specific chromatin state maps, histone ChIP seq data (called narrow peaks) from primary kidney tissue for H3K4me3, H3K4me1, H3K36me3, H3K27me3, H3K9me3 and H3K27ac was downloaded from the ENCODE data portal[34] (two donors, accession numbers ENCBS570IQU, ENCBS438CSQ) and the IHEC data portal[35] (four donors, accession numbers MS002202, MS040102, MS040202, MS040301). The chromatin state annotation was created with ChromHMM following previously described steps.[36] Briefly, in the binarization step, the "-peak" flag was set and the input .bed files were merged for each histone mark across all samples. In the annotation step, a pre-trained 18-state model published by the ROADMAP consortium[37] was used, obtained from

https://egg2.wustl.edu/roadmap/data/byFileType/chromhmmSegmentations/ChmmModels/core_K27ac/jointModel/final/.

The transcription factor ChIP datasets for HNF1A and HNF1B were downloaded as narrow peaks from the ENCODE database (accession numbers ENCFF022QCK, ENCFF767MSS). We downloaded publicly available single-nucleus ATAC-seq data from 12,720 human kidney cells[38] (https://susztaklab.com/human_kidney/igv/) and displayed the open chromatin peaks in kidney cell types of interest along with the bulk ATAC-seq and RNA-seq tracks which we generated from human kidney cortex and medulla.

Supplementary References

1. Shin, S.Y. *et al.* An atlas of genetic influences on human blood metabolites. *Nat Genet* **46**, 543-550 (2014).
2. Long, T. *et al.* Whole-genome sequencing identifies common-to-rare variants associated with human blood metabolites. *Nat Genet* **49**, 568-578 (2017).
3. Lotta, L.A. *et al.* A cross-platform approach identifies genetic regulators of human metabolism and health. *Nat Genet* **53**, 54-64 (2021).
4. Hysi, P.G. *et al.* Metabolome Genome-Wide Association Study Identifies 74 Novel Genomic Regions Influencing Plasma Metabolites Levels. *Metabolites* **12**(2022).
5. Yin, X. *et al.* Genome-wide association studies of metabolites in Finnish men identify disease-relevant loci. *Nat Commun* **13**, 1644 (2022).
6. Surendran, P. *et al.* Rare and common genetic determinants of metabolic individuality and their effects on human health. *Nat Med* **28**, 2321-2332 (2022).
7. Chen, Y. *et al.* Genomic atlas of the plasma metabolome prioritizes metabolites implicated in human diseases. *Nat Genet* (2023).
8. Mittelstrass, K. *et al.* Discovery of sexual dimorphisms in metabolic and genetic biomarkers. *PLoS Genet* **7**, e1002215 (2011).
9. Hartiala, J.A. *et al.* Genome-wide association study and targeted metabolomics identifies sex-specific association of CPS1 with coronary artery disease. *Nat Commun* **7**, 10558 (2016).
10. Elwi, A.N. *et al.* Renal nucleoside transporters: physiological and clinical implications. *Biochem Cell Biol* **84**, 844-58 (2006).
11. The GTEx Consortium. The GTEx Consortium atlas of genetic regulatory effects across human tissues. *Science* **369**, 1318-1330 (2020).
12. Munck, B.G. Transport of neutral and cationic amino acids across the brush-border membrane of the rabbit ileum. *J Membr Biol* **83**, 1-13 (1985).
13. Stanzick, K.J. *et al.* Discovery and prioritization of variants and genes for kidney function in >1.2 million individuals. *Nat Commun* **12**, 4350 (2021).
14. Broer, S. *et al.* Iminoglycinuria and hyperglycinuria are discrete human phenotypes resulting from complex mutations in proline and glycine transporters. *J Clin Invest* **118**, 3881-92 (2008).
15. The Atherosclerosis Risk in Communities (ARIC) Study: design and objectives. The ARIC investigators. *Am J Epidemiol* **129**, 687-702 (1989).
16. Schlosser, P. *et al.* Netboost: Boosting-Supported Network Analysis Improves High-Dimensional Omics Prediction in Acute Myeloid Leukemia and Huntington's Disease. *IEEE/ACM Trans Comput Biol Bioinform* **18**, 2635-2648 (2021).
17. Schlosser, P. *et al.* Genetic studies of urinary metabolites illuminate mechanisms of detoxification and excretion in humans. *Nat Genet* (2020).
18. Pierce, B.L. & Burgess, S. Efficient design for Mendelian randomization studies: subsample and 2-sample instrumental variable estimators. *Am J Epidemiol* **178**, 1177-84 (2013).
19. Yavorska, O.O. & Burgess, S. MendelianRandomization: an R package for performing Mendelian randomization analyses using summarized data. *Int J Epidemiol* **46**, 1734-1739 (2017).
20. Chang, C.C. *et al.* Second-generation PLINK: rising to the challenge of larger and richer datasets. *Gigascience* **4**, 7 (2015).
21. 1000 Genomes Project Consortium *et al.* A global reference for human genetic variation. *Nature* **526**, 68-74 (2015).
22. Kamat, M.A. *et al.* PhenoScanner V2: an expanded tool for searching human genotype-phenotype associations. *Bioinformatics* **35**, 4851-4853 (2019).
23. Voight, B.F., Kudaravalli, S., Wen, X. & Pritchard, J.K. A map of recent positive selection in the human genome. *PLoS Biol* **4**, e72 (2006).
24. Szpiech, Z.A. & Hernandez, R.D. selscan: an efficient multithreaded program to perform EHH-based scans for positive selection. *Mol Biol Evol* **31**, 2824-7 (2014).

25. Sabeti, P.C. *et al.* Detecting recent positive selection in the human genome from haplotype structure. *Nature* **419**, 832-7 (2002).
26. Gautier, M., Klassmann, A. & Vitalis, R. rehh 2.0: a reimplementation of the R package rehh to detect positive selection from haplotype structure. *Mol Ecol Resour* **17**, 78-90 (2017).
27. Li, Y. *et al.* Genome-wide studies reveal factors associated with circulating uromodulin and its relations with complex diseases. *JCI Insight* (2022).
28. Dobin, A. *et al.* STAR: ultrafast universal RNA-seq aligner. *Bioinformatics* **29**, 15-21 (2013).
29. Liao, Y., Smyth, G.K. & Shi, W. featureCounts: an efficient general purpose program for assigning sequence reads to genomic features. *Bioinformatics* **30**, 923-30 (2014).
30. Lopez-Delisle, L. *et al.* pyGenomeTracks: reproducible plots for multivariate genomic datasets. *Bioinformatics* **37**, 422-423 (2021).
31. Fornes, O. *et al.* JASPAR 2020: update of the open-access database of transcription factor binding profiles. *Nucleic Acids Res* **48**, D87-D92 (2020).
32. Schep, A. motifmatchr: Fast Motif Matching in R. R package version 1.16.0. edn (2021).
33. Manke, T., Heinig, M. & Vingron, M. Quantifying the effect of sequence variation on regulatory interactions. *Hum Mutat* **31**, 477-83 (2010).
34. Davis, C.A. *et al.* The Encyclopedia of DNA elements (ENCODE): data portal update. *Nucleic Acids Res* **46**, D794-D801 (2018).
35. Bujold, D. *et al.* The International Human Epigenome Consortium Data Portal. *Cell Syst* **3**, 496-499 e2 (2016).
36. Ernst, J. & Kellis, M. Chromatin-state discovery and genome annotation with ChromHMM. *Nat Protoc* **12**, 2478-2492 (2017).
37. Roadmap Epigenomics Consortium *et al.* Integrative analysis of 111 reference human epigenomes. *Nature* **518**, 317-30 (2015).
38. Sheng, X. *et al.* Mapping the genetic architecture of human traits to cell types in the kidney identifies mechanisms of disease and potential treatments. *Nat Genet* **53**, 1322-1333 (2021).
39. Liu, B., Gloudemans, M.J., Rao, A.S., Ingelsson, E. & Montgomery, S.B. Abundant associations with gene expression complicate GWAS follow-up. *Nat Genet* **51**, 768-769 (2019).

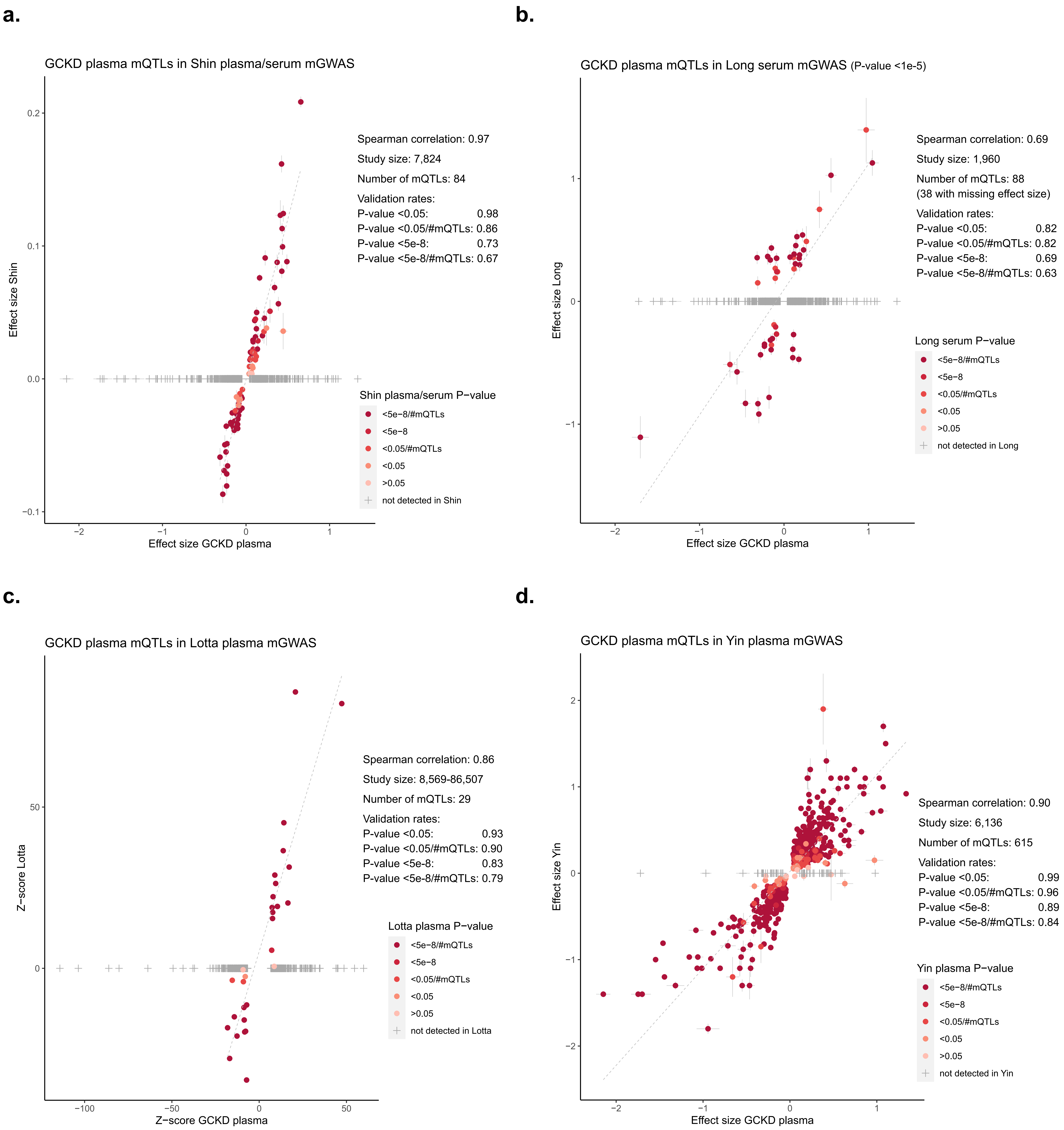
a.
GCKD plasma mQTLs in Shin plasma/serum mGWAS
Spearman correlation: 0.97
Study size: 7,824
Number of mQTLs: 84
Validation rates:
P-value <0.05: 0.98
P-value <0.05/#mQTLs: 0.86
P-value <5e-8: 0.73
P-value <5e-8/#mQTLs: 0.67
Shin plasma/serum P−value
<5e−8/#mQTLs
<5e−8
<0.05/#mQTLs
<0.05
>0.05
not detected in Shin
Effect size Shin
Effect size GCKD plasma
b.
GCKD plasma mQTLs in Long serum mGWAS (P-value <1e-5)
Spearman correlation: 0.69
Study size: 1,960
Number of mQTLs: 88
(38 with missing effect size)
Validation rates:
P-value <0.05: 0.82
P-value <0.05/#mQTLs: 0.82
P-value <5e-8: 0.69
P-value <5e-8/#mQTLs: 0.63
Long serum P−value
<5e−8/#mQTLs
<5e−8
<0.05/#mQTLs
<0.05
>0.05
not detected in Long
Effect size Long
Effect size GCKD plasma
c.
GCKD plasma mQTLs in Lotta plasma mGWAS
Spearman correlation: 0.86
Study size: 8,569-86,507
Number of mQTLs: 29
Validation rates:
P-value <0.05: 0.93
P-value <0.05/#mQTLs: 0.90
P-value <5e-8: 0.83
P-value <5e-8/#mQTLs: 0.79
Lotta plasma P−value
<5e−8/#mQTLs
<5e−8
<0.05/#mQTLs
<0.05
>0.05
not detected in Lotta
Z−score Lotta
Z−score GCKD plasma
d.
GCKD plasma mQTLs in Yin plasma mGWAS
Spearman correlation: 0.90
Study size: 6,136
Number of mQTLs: 615
Validation rates:
P-value <0.05: 0.99
P-value <0.05/#mQTLs: 0.96
P-value <5e-8: 0.89
P-value <5e-8/#mQTLs: 0.84
Yin plasma P−value
<5e−8/#mQTLs
<5e−8
<0.05/#mQTLs
<0.05
>0.05
not detected in Yin
Effect size Yin
Effect size GCKD plasma

e.

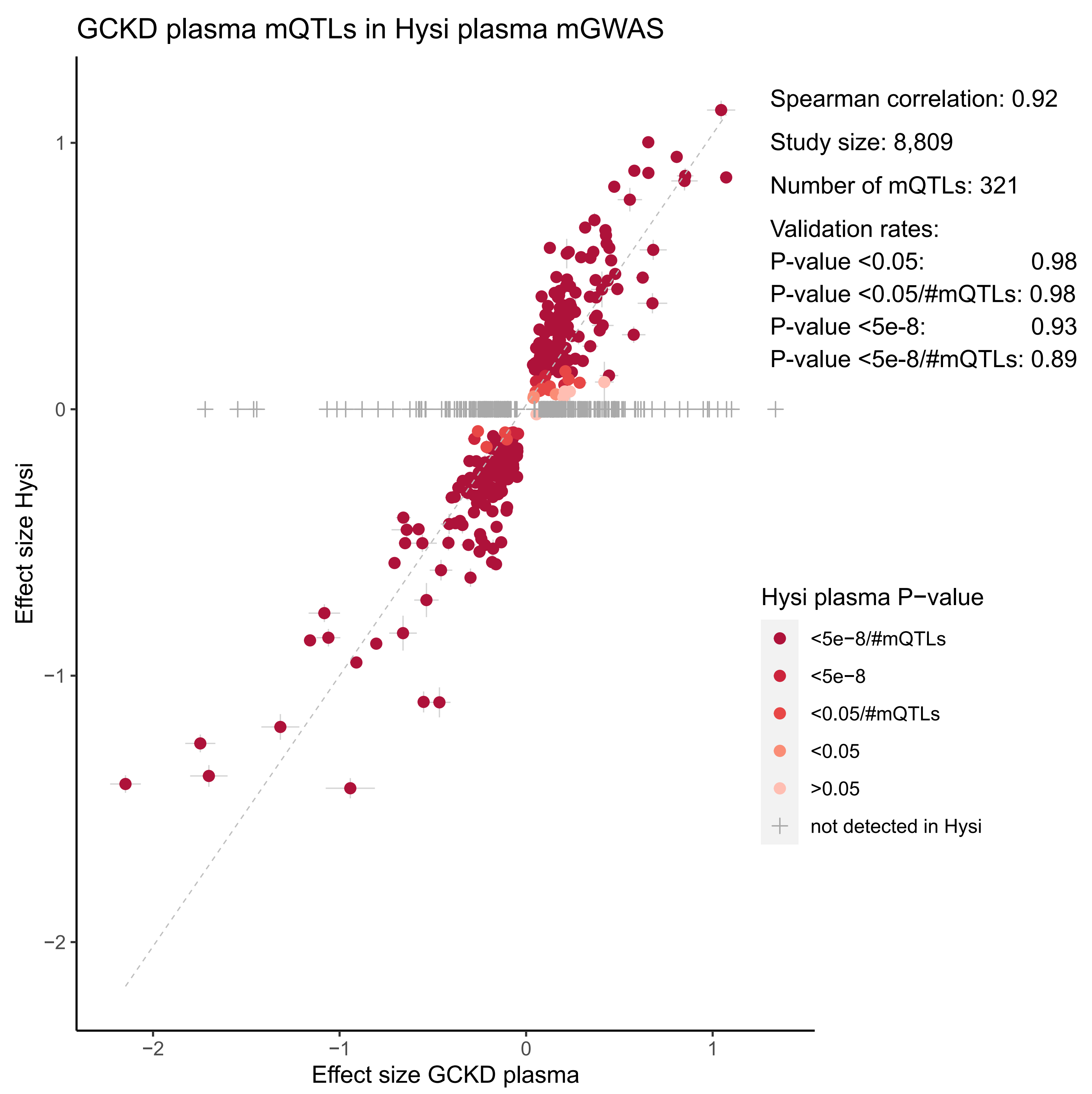
GCKD plasma mQTLs in Hysi plasma mGWAS
Spearman correlation: 0.92
Study size: 8,809
Number of mQTLs: 321
Validation rates:
P-value <0.05: 0.98
P-value <0.05/#mQTLs: 0.98
P-value <5e-8: 0.93
P-value <5e-8/#mQTLs: 0.89
Hysi plasma P−value
<5e−8/#mQTLs
<5e−8
<0.05/#mQTLs
<0.05
>0.05
not detected in Hysi
Effect size Hysi
Effect size GCKD plasma
1
0
−1
−2
−2
−1
0
1

f.

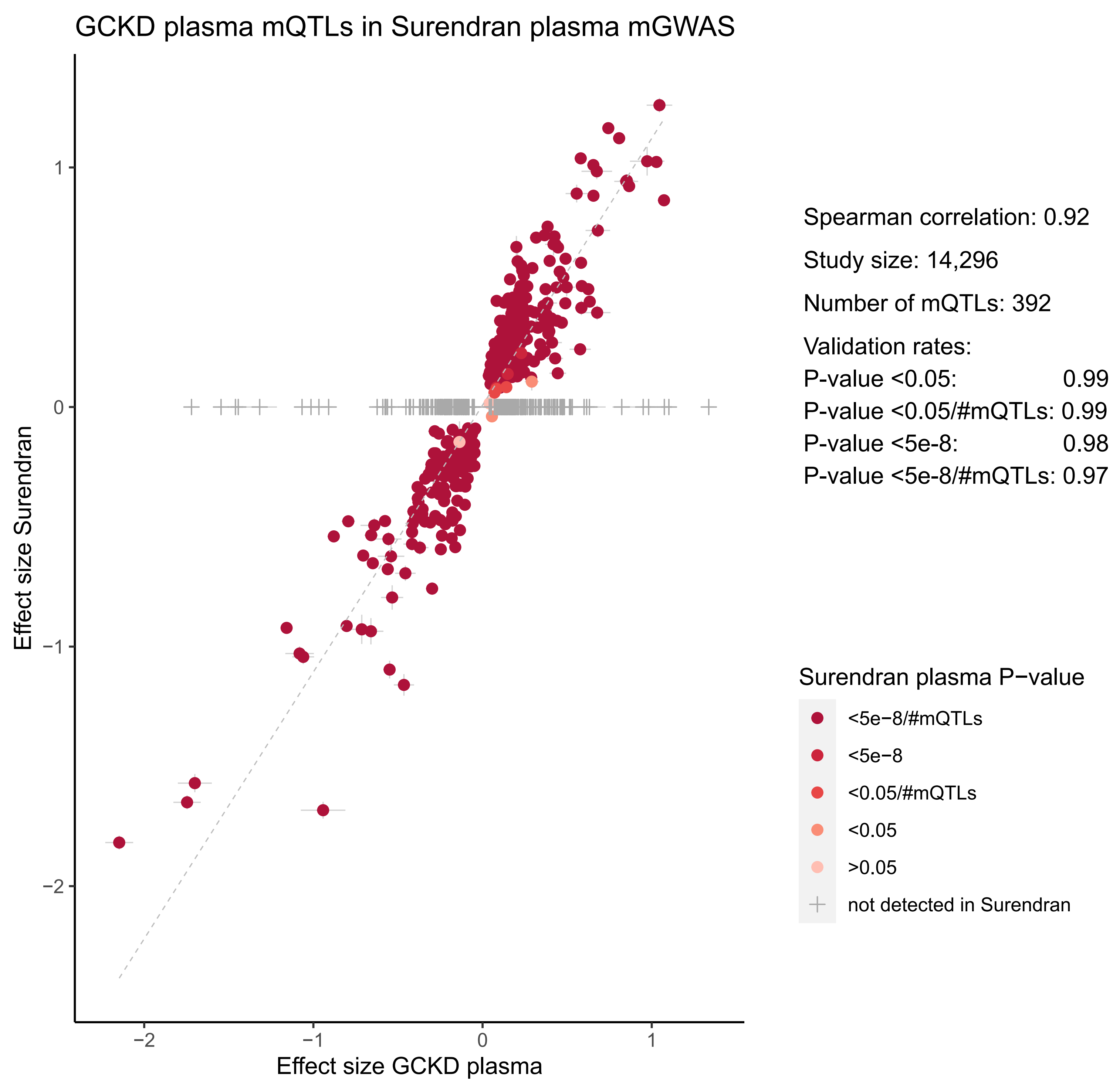
GCKD plasma mQTLs in Surendran plasma mGWAS
Spearman correlation: 0.92
Study size: 14,296
Number of mQTLs: 392
Validation rates:
P-value <0.05: 0.99
P-value <0.05/#mQTLs: 0.99
P-value <5e-8: 0.98
P-value <5e-8/#mQTLs: 0.97
Surendran plasma P−value
<5e−8/#mQTLs
<5e−8
<0.05/#mQTLs
<0.05
>0.05
not detected in Surendran
Effect size Surendran
Effect size GCKD plasma
1
0
−1
−2
−2
−1
0
1

g.

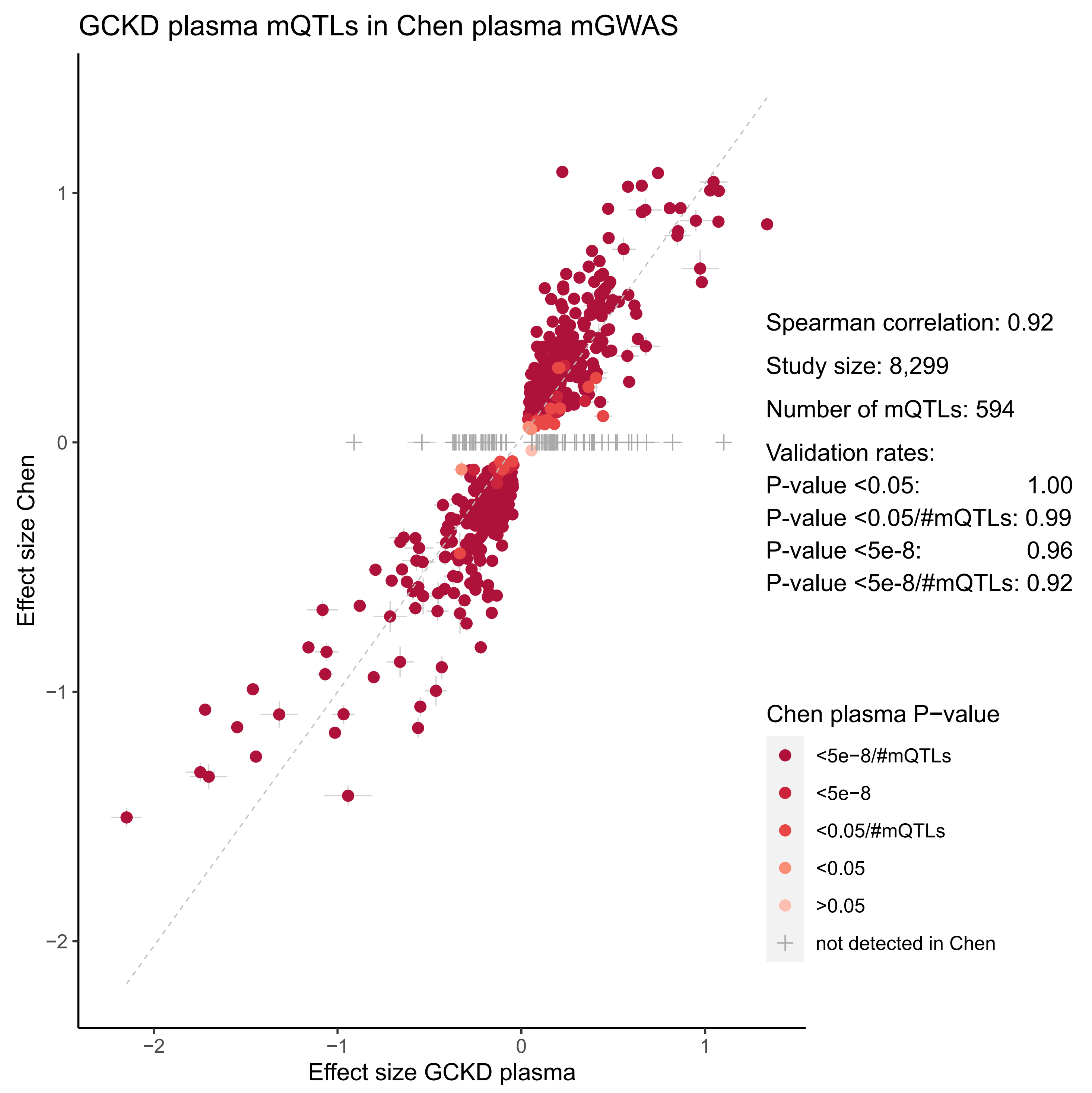
GCKD plasma mQTLs in Chen plasma mGWAS
Spearman correlation: 0.92
Study size: 8,299
Number of mQTLs: 594
Validation rates:
P-value <0.05: 1.00
P-value <0.05/#mQTLs: 0.99
P-value <5e-8: 0.96
P-value <5e-8/#mQTLs: 0.92
Chen plasma P−value
<5e−8/#mQTLs
<5e−8
<0.05/#mQTLs
<0.05
>0.05
not detected in Chen
Effect size Chen
Effect size GCKD plasma
1
0
−1
−2
−2
−1
0
1

Supplementary Figure 1: Genetic effects of GCKD plasma mQTLs on levels of the corresponding metabolite in published plasma/serum mGWAS.

The effect sizes or z-scores of GCKD plasma mQTLs are shown on the x-axis, whereas the effect sizes or z-scores of the corresponding metabolite at the corresponding index SNP or proxy SNP in high LD (r^2>0.8) in published summary statistics of Shin *et al* 2014 (a), Long *et al* 2017 (b), Lotta *et al* 2021 (c), Yin *et al* 2022 (d), Hysi *et al* 2022 (e), Surendran *et al* 2022 (f), and Chen *et al* 2023 (g) are shown on the y-axis. The color indicates the P-value in the corresponding published plasma/serum mGWAS summary statistics, and the gray dashed line is the linear regression line. The gray crosses represent GCKD plasma index SNPs for which the corresponding metabolite, SNP (and proxy SNP respectively), or both were not detected in the corresponding plasma/serum mGWAS study. Validation rates at different levels of significance only consider direction-consistent effects. Serum mGWAS summary statistics from Long *et al* 2017 are only available for associations with P-value <1e-5.

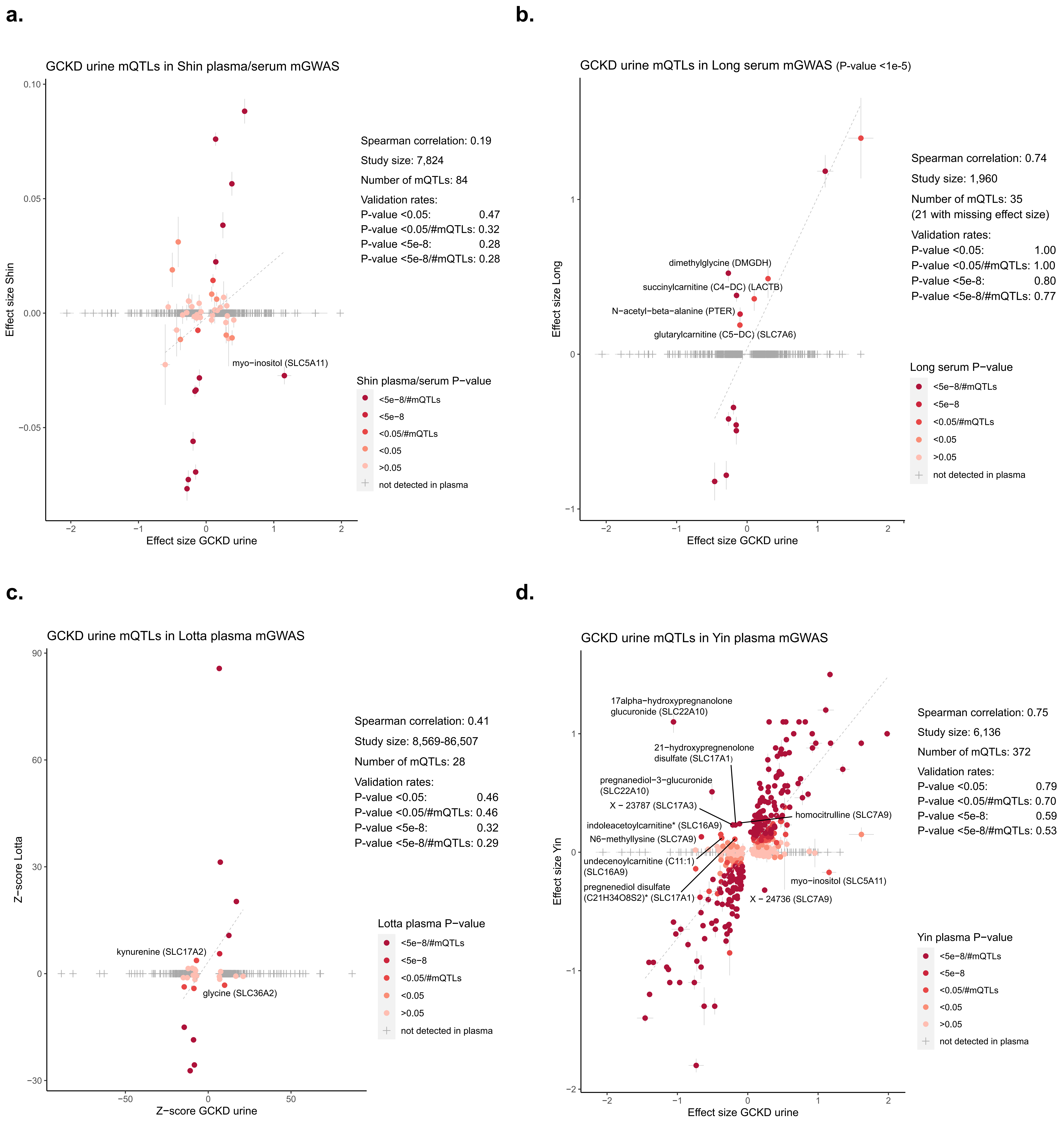
a.
GCKD urine mQTLs in Shin plasma/serum mGWAS
Effect size Shin
Effect size GCKD urine
myo−inositol (SLC5A11)
Spearman correlation: 0.19
Study size: 7,824
Number of mQTLs: 84
Validation rates:
P-value <0.05: 0.47
P-value <0.05/#mQTLs: 0.32
P-value <5e-8: 0.28
P-value <5e-8/#mQTLs: 0.28
Shin plasma/serum P−value
<5e−8/#mQTLs
<5e−8
<0.05/#mQTLs
<0.05
>0.05
not detected in plasma
b.
GCKD urine mQTLs in Long serum mGWAS (P-value <1e-5)
Effect size Long
Effect size GCKD urine
dimethylglycine (DMGDH)
succinylcarnitine (C4−DC) (LACTB)
N−acetyl−beta−alanine (PTER)
glutarylcarnitine (C5−DC) (SLC7A6)
Spearman correlation: 0.74
Study size: 1,960
Number of mQTLs: 35
(21 with missing effect size)
Validation rates:
P-value <0.05: 1.00
P-value <0.05/#mQTLs: 1.00
P-value <5e-8: 0.80
P-value <5e-8/#mQTLs: 0.77
Long serum P−value
<5e−8/#mQTLs
<5e−8
<0.05/#mQTLs
<0.05
>0.05
not detected in plasma
c.
GCKD urine mQTLs in Lotta plasma mGWAS
Z−score Lotta
Z−score GCKD urine
kynurenine (SLC17A2)
glycine (SLC36A2)
Spearman correlation: 0.41
Study size: 8,569-86,507
Number of mQTLs: 28
Validation rates:
P-value <0.05: 0.46
P-value <0.05/#mQTLs: 0.46
P-value <5e-8: 0.32
P-value <5e-8/#mQTLs: 0.29
Lotta plasma P−value
<5e−8/#mQTLs
<5e−8
<0.05/#mQTLs
<0.05
>0.05
not detected in plasma
d.
GCKD urine mQTLs in Yin plasma mGWAS
Effect size Yin
Effect size GCKD urine
17alpha−hydroxypregnanolone glucuronide (SLC22A10)
21−hydroxypregnenolone disulfate (SLC17A1)
pregnanediol−3−glucuronide (SLC22A10)
X − 23787 (SLC17A3)
indoleacetoylcarnitine* (SLC16A9)
N6−methyllysine (SLC7A9)
undecenoylcarnitine (C11:1) (SLC16A9)
pregnenediol disulfate (C21H34O8S2)* (SLC17A1)
homocitrulline (SLC7A9)
myo−inositol (SLC5A11)
X − 24736 (SLC7A9)
Spearman correlation: 0.75
Study size: 6,136
Number of mQTLs: 372
Validation rates:
P-value <0.05: 0.79
P-value <0.05/#mQTLs: 0.70
P-value <5e-8: 0.59
P-value <5e-8/#mQTLs: 0.53
Yin plasma P−value
<5e−8/#mQTLs
<5e−8
<0.05/#mQTLs
<0.05
>0.05
not detected in plasma

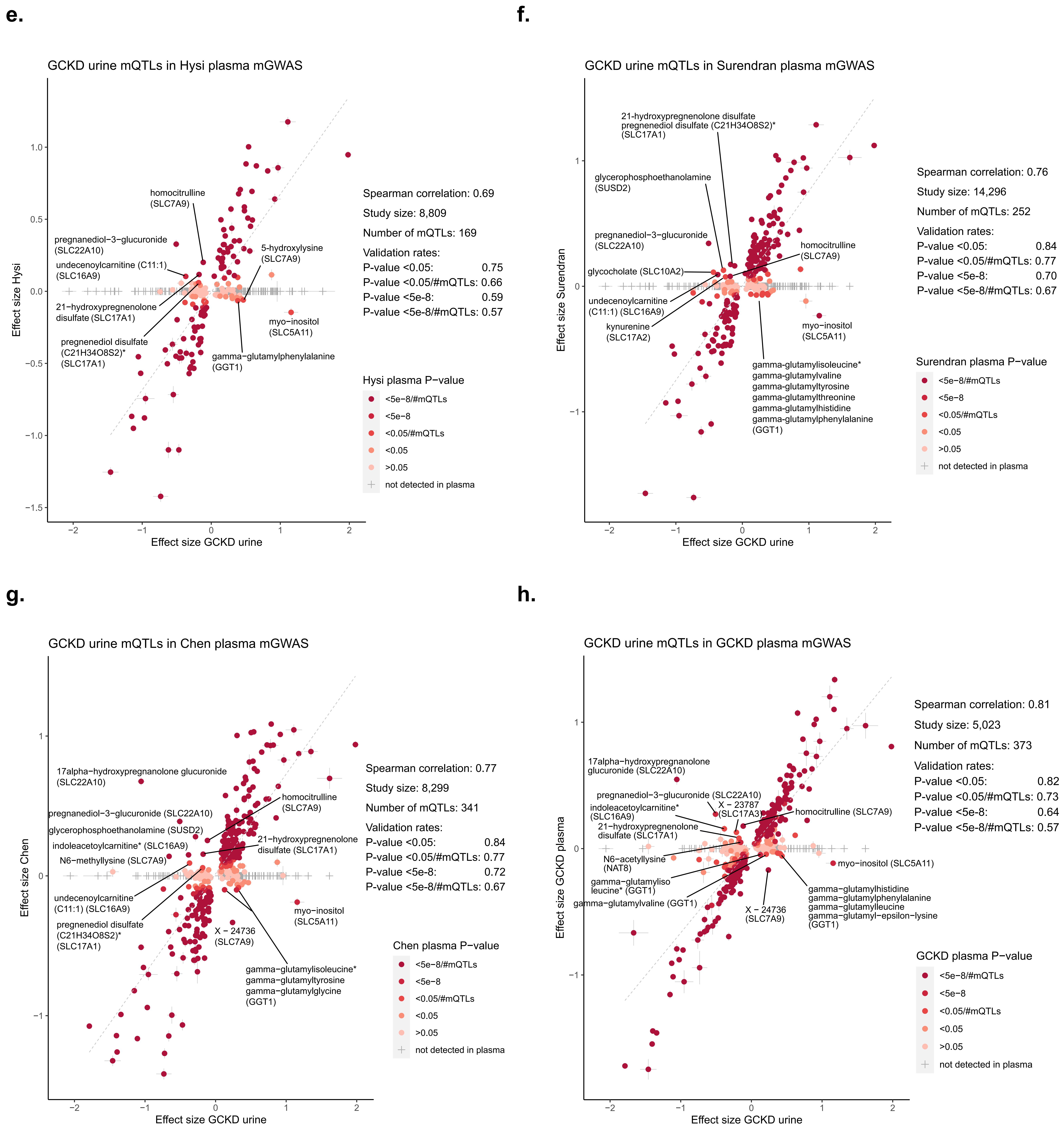
e.
GCKD urine mQTLs in Hysi plasma mGWAS
homocitrulline (SLC7A9)
pregnanediol−3−glucuronide (SLC22A10)
undecenoylcarnitine (C11:1) (SLC16A9)
5-hydroxylysine (SLC7A9)
21−hydroxypregnenolone disulfate (SLC17A1)
pregnenediol disulfate (C21H34O8S2)* (SLC17A1)
myo−inositol (SLC5A11)
gamma−glutamylphenylalanine (GGT1)
Effect size Hysi
Effect size GCKD urine
Spearman correlation: 0.69
Study size: 8,809
Number of mQTLs: 169
Validation rates:
P-value <0.05: 0.75
P-value <0.05/#mQTLs: 0.66
P-value <5e-8: 0.59
P-value <5e-8/#mQTLs: 0.57
Hysi plasma P−value
<5e−8/#mQTLs
<5e−8
<0.05/#mQTLs
<0.05
>0.05
not detected in plasma
f.
GCKD urine mQTLs in Surendran plasma mGWAS
21−hydroxypregnenolone disulfate
pregnenediol disulfate (C21H34O8S2)* (SLC17A1)
glycerophosphoethanolamine (SUSD2)
pregnanediol−3−glucuronide (SLC22A10)
glycocholate (SLC10A2)
homocitrulline (SLC7A9)
undecenoylcarnitine (C11:1) (SLC16A9)
kynurenine (SLC17A2)
myo−inositol (SLC5A11)
gamma−glutamylisoleucine*
gamma−glutamylvaline
gamma−glutamyltyrosine
gamma−glutamylthreonine
gamma−glutamylhistidine
gamma−glutamylphenylalanine
(GGT1)
Effect size Surendran
Effect size GCKD urine
Spearman correlation: 0.76
Study size: 14,296
Number of mQTLs: 252
Validation rates:
P-value <0.05: 0.84
P-value <0.05/#mQTLs: 0.77
P-value <5e-8: 0.70
P-value <5e-8/#mQTLs: 0.67
Surendran plasma P−value
<5e−8/#mQTLs
<5e−8
<0.05/#mQTLs
<0.05
>0.05
not detected in plasma
g.
GCKD urine mQTLs in Chen plasma mGWAS
17alpha−hydroxypregnanolone glucuronide (SLC22A10)
pregnanediol−3−glucuronide (SLC22A10)
glycerophosphoethanolamine (SUSD2)
indoleacetoylcarnitine* (SLC16A9)
N6−methyllysine (SLC7A9)
homocitrulline (SLC7A9)
21−hydroxypregnenolone disulfate (SLC17A1)
undecenoylcarnitine (C11:1) (SLC16A9)
pregnenediol disulfate (C21H34O8S2)* (SLC17A1)
X − 24736 (SLC7A9)
myo−inositol (SLC5A11)
gamma−glutamylisoleucine*
gamma−glutamyltyrosine
gamma−glutamylglycine
(GGT1)
Effect size Chen
Effect size GCKD urine
Spearman correlation: 0.77
Study size: 8,299
Number of mQTLs: 341
Validation rates:
P-value <0.05: 0.84
P-value <0.05/#mQTLs: 0.77
P-value <5e-8: 0.72
P-value <5e-8/#mQTLs: 0.67
Chen plasma P−value
<5e−8/#mQTLs
<5e−8
<0.05/#mQTLs
<0.05
>0.05
not detected in plasma
h.
GCKD urine mQTLs in GCKD plasma mGWAS
17alpha−hydroxypregnanolone glucuronide (SLC22A10)
pregnanediol−3−glucuronide (SLC22A10)
X − 23787 (SLC17A3)
indoleacetoylcarnitine* (SLC16A9)
21−hydroxypregnenolone disulfate (SLC17A1)
homocitrulline (SLC7A9)
N6−acetyllysine (NAT8)
myo−inositol (SLC5A11)
gamma−glutamyliso leucine* (GGT1)
gamma−glutamylvaline (GGT1)
X − 24736 (SLC7A9)
gamma−glutamylhistidine
gamma−glutamylphenylalanine
gamma−glutamylleucine
gamma−glutamyl−epsilon−lysine
(GGT1)
Effect size GCKD plasma
Effect size GCKD urine
Spearman correlation: 0.81
Study size: 5,023
Number of mQTLs: 373
Validation rates:
P-value <0.05: 0.82
P-value <0.05/#mQTLs: 0.73
P-value <5e-8: 0.64
P-value <5e-8/#mQTLs: 0.57
GCKD plasma P−value
<5e−8/#mQTLs
<5e−8
<0.05/#mQTLs
<0.05
>0.05
not detected in plasma

Supplementary Figure 2: Genetic effects of GCKD urine mQTLs on levels of the corresponding metabolite in published plasma/serum mGWAS.

The effect sizes or z-scores of GCKD urine mQTLs are shown on the x-axis, whereas the effect sizes or z-scores of the corresponding metabolite at the corresponding index SNP or proxy SNP in high LD (r^2>0.8) in published summary statistics of Shin *et al* 2014 (a), Long *et al* 2017 (b), Lotta *et al* 2021 (c), Yin *et al* 2022 (d), Hysi *et al* 2022 (e), Surendran *et al* 2022 (f), Chen *et al* 2023 (g), and of our GCKD plasma mGWAS (h) are shown on the y-axis. The color indicates the P-value in the corresponding published plasma/serum mGWAS summary statistics, and the gray dashed line is the linear regression line. The gray crosses represent GCKD urine mQTLs for which the corresponding metabolite, SNP (or, if applicable, proxy SNP), or both were not detected in the corresponding plasma/serum mGWAS study. Validation rates at different levels of significance consider all effects regardless of their direction consistency, because there are several metabolites where an inverse association in urine versus plasma is biologically plausible. All mQTLs with an inconsistent effect direction in urine versus plasma/serum and a P-value <0.05/#mQTLs in the corresponding published plasma/serum study are labeled with the corresponding biochemical name and assigned gene. Serum mGWAS summary statistics from Long *et al* 2017 are only available for associations with P-value <1e-5.

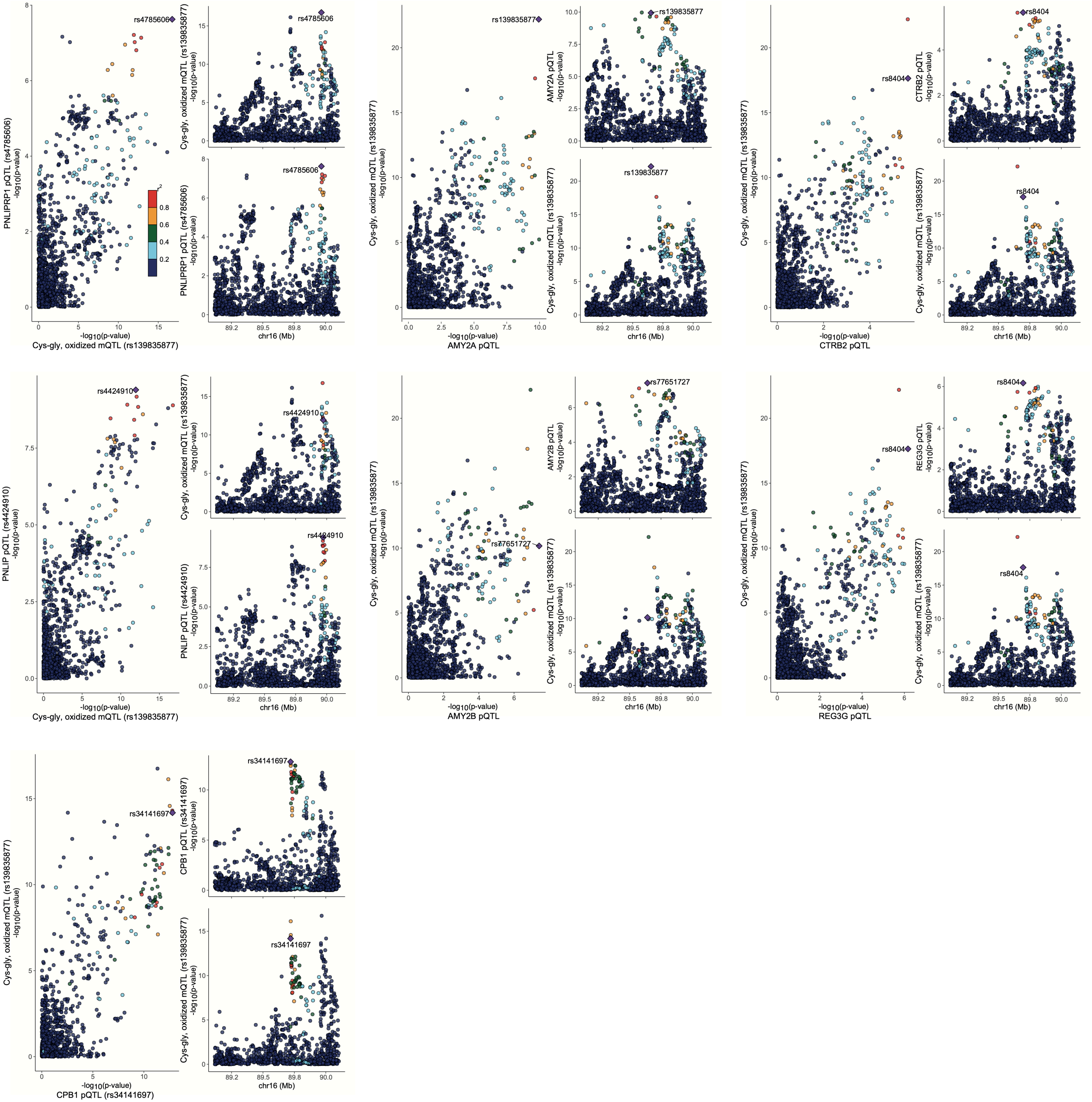
rs4785606
PNLIPRP1 pQTL (rs4785606)
-log10(p-value)
Cys-gly, oxidized mQTL (rs139835877)
PNLIPRP1 pQTL (rs4785606)
r2
0.8
0.6
0.4
0.2
chr16 (Mb)
rs139835877
AMY2A pQTL
CTRB2 pQTL
rs8404
rs4424910
PNLIP pQTL (rs4424910)
rs77651727
AMY2B pQTL
REG3G pQTL
rs34141697
CPB1 pQTL (rs34141697)

Supplementary Figure 3: Comparison of genetic associations at the *DPEP1* locus for one exemplary plasma metabolite, cysteinylglycine, and all seven digestive proteins in plasma.

Association patterns were visualized using LocusCompare[39] version 1.0.0 and display conditional colocalization statistics between the mQTL for plasma cysteinylglycine, oxidized (independent SNP rs139835877) and each of the pQTLs for PNLIPRPI, AMY2A, CTRB2, PNLIP, AMY2B, REG3 and CPB1 (Methods). Conditional two-sided P-values are shown for PNLIPRP1 (independent SNP rs4785606), PNLIP (independent SNP rs4424910) and CPB1 (independent SNP rs34141697). Marginal P-values are displayed for AMY2A, CTRB2, AMY2B and REG3. SNPs are color-coded to reflect their LD with this SNP (from pairwise R^2 values from the HapMap CEU).

Supplementary Data 1: Regional association plots for mQTLs identified in mGWAS of plasma metabolite levels

For each of the 677 mQTLs, the region for plotting was selected as 1-Mb for regions with a single mQTL, and as the outer borders of merged overlapping 1-Mb windows for regions with more than one adjacent index variants associated with the same metabolite. The associated metabolite is included in the title of each plot. The extended MHC region was treated as one region. A measure of linkage disequilibrium with the index SNP (lowest P-value, marked in purple), is presented as color-coded r^2. P-values were calculated based on linear regression.

See separate file.

Supplementary Data 2: Regional association plots for mQTLs identified in mGWAS of urine metabolite levels

For each of the 622 mQTLs, the region for plotting was selected as 1-Mb for regions with a single mQTL, and as the outer borders of merged overlapping 1-Mb windows for regions with more than one adjacent index variants associated with the same metabolite. The associated metabolite is included in the title of each plot. The extended MHC region was treated as one region. A measure of linkage disequilibrium with the index SNP (lowest P-value, marked in purple), is presented as color-coded r^2. P-values were calculated based on linear regression.

See separate file.
