# Supplementary material for: Genetic studies of paired metabolomes reveal enzymatic and transport processes at the interface of plasma and urine
Source: Nat Genet. 2023 Jun 5;55(6):995–1008. doi: 10.1038/s41588-023-01409-8 (PMC10260405; doi:10.1038/s41588-023-01409-8)
Supplement: Supplementary file 2 — Reporting Summary [file 41588_2023_1409_MOESM2_ESM.pdf]

nature portfolio

Corresponding author(s): Pascal Schlosser, Anna Köttgen

Last updated by author(s): Mar 23, 2023

Reporting Summary

Nature Portfolio wishes to improve the reproducibility of the work that we publish. This form provides structure for consistency and transparency in reporting. For further information on Nature Portfolio policies, see our Editorial Policies and the Editorial Policy Checklist.

Statistics

For all statistical analyses, confirm that the following items are present in the figure legend, table legend, main text, or Methods section.

n/a	Confirmed	
☐	☒	The exact sample size (*n*) for each experimental group/condition, given as a discrete number and unit of measurement
☒	☐	A statement on whether measurements were taken from distinct samples or whether the same sample was measured repeatedly
☐	☒	The statistical test(s) used AND whether they are one- or two-sided<br>*Only common tests should be described solely by name; describe more complex techniques in the Methods section.*
☐	☒	A description of all covariates tested
☐	☒	A description of any assumptions or corrections, such as tests of normality and adjustment for multiple comparisons
☐	☒	A full description of the statistical parameters including central tendency (e.g. means) or other basic estimates (e.g. regression coefficient) AND variation (e.g. standard deviation) or associated estimates of uncertainty (e.g. confidence intervals)
☐	☒	For null hypothesis testing, the test statistic (e.g. *F*, *t*, *r*) with confidence intervals, effect sizes, degrees of freedom and *P* value noted<br>*Give P values as exact values whenever suitable.*
☐	☒	For Bayesian analysis, information on the choice of priors and Markov chain Monte Carlo settings
☒	☐	For hierarchical and complex designs, identification of the appropriate level for tests and full reporting of outcomes
☐	☒	Estimates of effect sizes (e.g. Cohen's *d*, Pearson's *r*), indicating how they were calculated

Our web collection on statistics for biologists contains articles on many of the points above.

Software and code

Policy information about availability of computer code

Data collection	Data preparation, quality control and statistical analyses of the data presented in this manuscript were performed at the Institute of Genetic Epidemiology, Medical Center - University of Freiburg, Freiburg (Germany) and the Department of Epidemiology, Johns Hopkins University, Baltimore, MD, USA.
Data analysis	- Software-tools for genotype imputation:<br>Michigan Imputation Server (incl. minimac3 v2.0.1, Eagle v2.3)<br>- Software-tools for association analyses: in-house pipeline based on SNPTEST v2.5.2<br>- Software-tools for postprocessing of results: LocusZoom v1.3, Circos v0.69-6, R-package gtx v2.1.6, Netboost v1.0.0, Seurat v2.3.4, GCTA v1.91.6 beta, pyGenomeTracks v3.7, locuscompare v1.0.0, R package MendelianRandomization (v0.6.0), PLINK v1.90<br>- Data bases, publicly available: SNiPA v3.3 (incl. CADD version 1.3, Ensembl VEP tool), NephQTL browser, GTEx V8, Ensembl Biomart, GO, KEGG, GeneAtlas (UK Biobank project), PhenoScanner V2<br>- Miscellaneous: R v3.5.3<br>References or website addresses are provided in manuscript.

For manuscripts utilizing custom algorithms or software that are central to the research but not yet described in published literature, software must be made available to editors and reviewers. We strongly encourage code deposition in a community repository (e.g. GitHub). See the Nature Portfolio guidelines for submitting code & software for further information.

Data

Policy information about availability of data

All manuscripts must include a data availability statement. This statement should provide the following information, where applicable:

- Accession codes, unique identifiers, or web links for publicly available datasets
- A description of any restrictions on data availability
- For clinical datasets or third party data, please ensure that the statement adheres to our policy

Genome-wide summary statistics are available through the NHGRI-EBI GWAS Catalog (GCST90264176-90266872, https://www.ebi.ac.uk/gwas/).

Field-specific reporting

Please select the one below that is the best fit for your research. If you are not sure, read the appropriate sections before making your selection.

☒ Life sciences ☐ Behavioural & social sciences ☐ Ecological, evolutionary & environmental sciences

For a reference copy of the document with all sections, see nature.com/documents/nr-reporting-summary-flat.pdf

Life sciences study design

All studies must disclose on these points even when the disclosure is negative.

Sample size	We included all 5023 GCKD participants with available plasma or urine metabolite quantification. A previous analysis of a subsample of 1627 GCKD participants with urine metabolite measurements has identified 240 mQTLs.
Data exclusions	Metabolites were excluded for high proportions of missingness (>80%). Samples were excluded if no genotypes were available. This is clearly described in the methods.
Replication	We evaluated reproducibility of our findings by three means :<br>- We assessed the presence and correlation of genetics effects using data from seven large studies of common variants and the plasma/serum metabolome as quantified with the Metabolon assay in European ancestry populations that were published in peer-reviewed journals, to maximize overlap with our findings. We observed excellent consistency of effect directions and validation rates, as outlined in the article.<br>- For urine mQTLs, 98.2% of 226 published urine mQTLs showed significant P-values ($<5\times10^{-8}/226$) and consistent effect direction.<br>- Independent replication testing was performed using genetic and plasma metabolome data from 3,603 European ancestry and 818 African American participants of the population-based Atherosclerosis Risk in Communities (ARIC) study (Methods). The results are described in the manuscript and include high replication rates for plasma mQTLs (94%) in the larger European ancestry sample.
Randomization	Not relevant to this study because this is an observational study
Blinding	Not relevant to this study because this is an observational study

Reporting for specific materials, systems and methods

We require information from authors about some types of materials, experimental systems and methods used in many studies. Here, indicate whether each material, system or method listed is relevant to your study. If you are not sure if a list item applies to your research, read the appropriate section before selecting a response.

Materials & experimental systems

n/a	Involved in the study	
☒	☐	Antibodies
☒	☐	Eukaryotic cell lines
☒	☐	Palaeontology and archaeology
☒	☐	Animals and other organisms
☐	☒	Human research participants
☐	☒	Clinical data
☒	☐	Dual use research of concern

Methods

n/a	Involved in the study	
☒	☐	ChIP-seq
☒	☐	Flow cytometry
☒	☐	MRI-based neuroimaging

Human research participants

Policy information about studies involving human research participants

Population characteristics	Please see Supplementary Table 1.<br>Characteristic Overall (N=5,023)

	Female sex 39.9% (2003)<br>Hemoglobin A1C (mmol/mol) 45.78 (11.21)<br>Age (years) 60.09 (11.97)<br>eGFRcr (mL/min/1.73m²) 49.45 (18.13)<br>Urinary albumin-to-creatinine ratio (mg/g) 50.52 [9.55;386.19]<br>Systolic blood pressure (mm Hg) 139.45 (20.35)<br>BMI (kg/m²) 29.8 (5.95)<br>Albumin (g/l) 38.34 (4.44)
Recruitment	The GCKD study is an ongoing prospective observational cohort study of participants with CKD. Between 2010 and 2012, 5,217 adult persons with CKD under regular care by nephrologists provided written informed consent and were enrolled into the study at nine participating study centers across Germany (Aachen, Berlin, Erlangen, Freiburg, Hannover, Heidelberg, Jena, München, Würzburg). Participants could not self-select into the study, but it cannot be excluded that eligible persons with many or severe comorbidities were less likely to participate than other eligible participants. For this project, all participants with available plasma or urine collected at the baseline visit were selected (N=5,023).
Ethics oversight	The GCKD Study was registered in the national registry for clinical studies (DRKS 00003971) and approved by all local ethic committees of the participating centers (Universities or Medical Faculties of Aachen, Berlin, Erlangen, Freiburg, Hannover, Heidelberg, Jena, München, Würzburg).

Note that full information on the approval of the study protocol must also be provided in the manuscript.

Clinical data

Policy information about clinical studies

All manuscripts should comply with the ICMJE guidelines for publication of clinical research and a completed CONSORT checklist must be included with all submissions.

Clinical trial registration	This study is an observational study (DRKS 00003971).
Study protocol	The study protocol and design has been published (PMID: 21862458).
Data collection	Data was collected during GCKD study visits by trained personnel following a published pre-specified protocol and standard operating procedures, and captured with the software Askimed (https://www.askimed.com/). The participants are currently followed for clinical outcomes for more than 10 years.
Outcomes	The primary outcomes of this study were metabolite levels, which was defined before study initiation by the authors. Non-targeted MS analysis was performed at Metabolon, Inc. from plasma and urine samples collected at the study's baseline visit, as described in detail in the publication.
