# Supplementary material for: Genetic studies of paired metabolomes reveal enzymatic and transport processes at the interface of plasma and urine
Source: Nat Genet. 2023 Jun 5;55(6):995–1008. doi: 10.1038/s41588-023-01409-8 (PMC10260405; doi:10.1038/s41588-023-01409-8)

arachidonate (20:4n6)

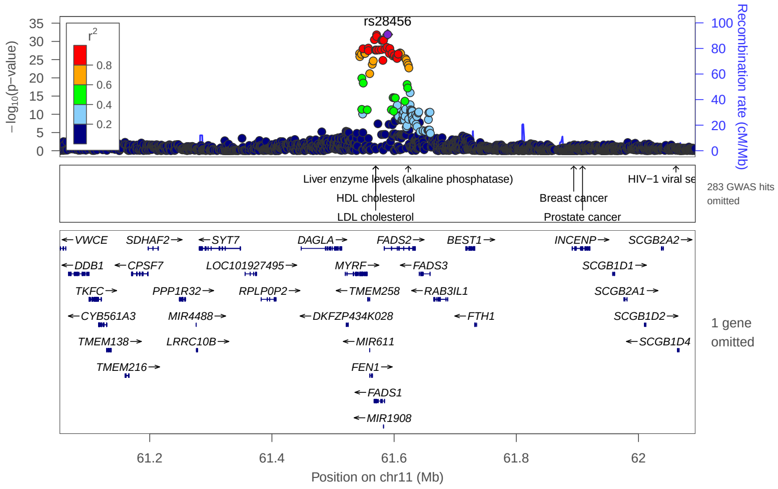

beta−hydroxyisovalerate

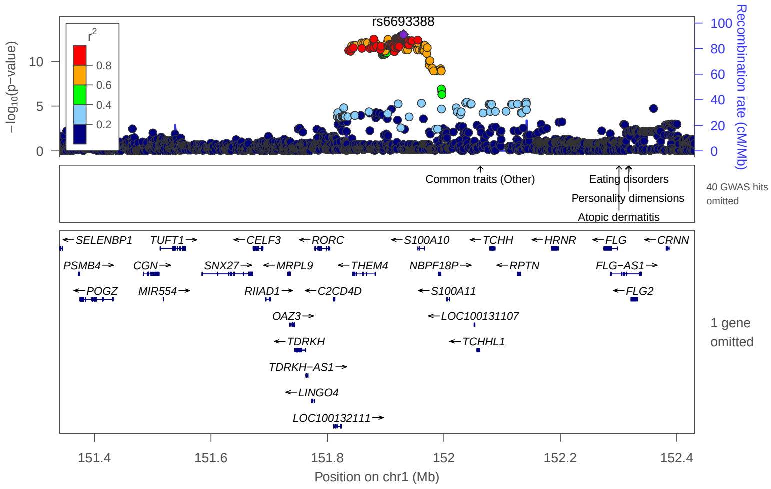

beta−hydroxyisovalerate

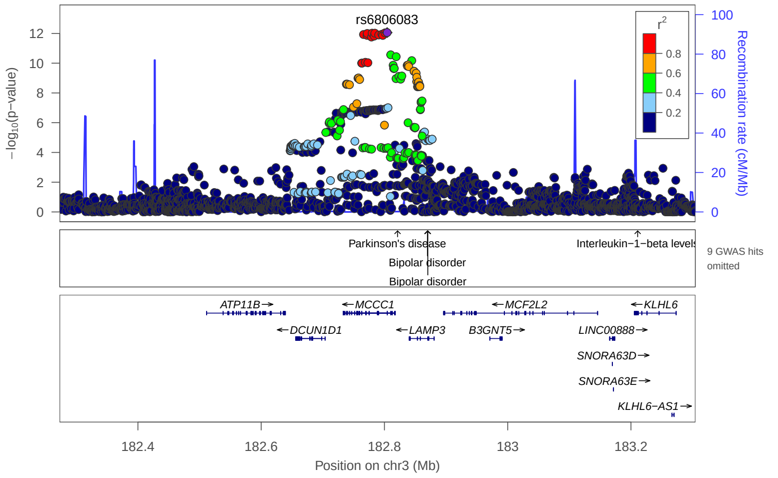

beta−hydroxyisovalerate

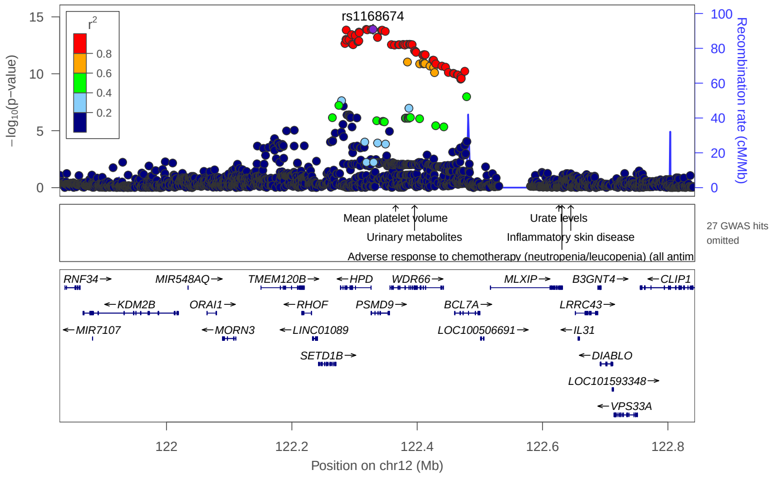

lysine

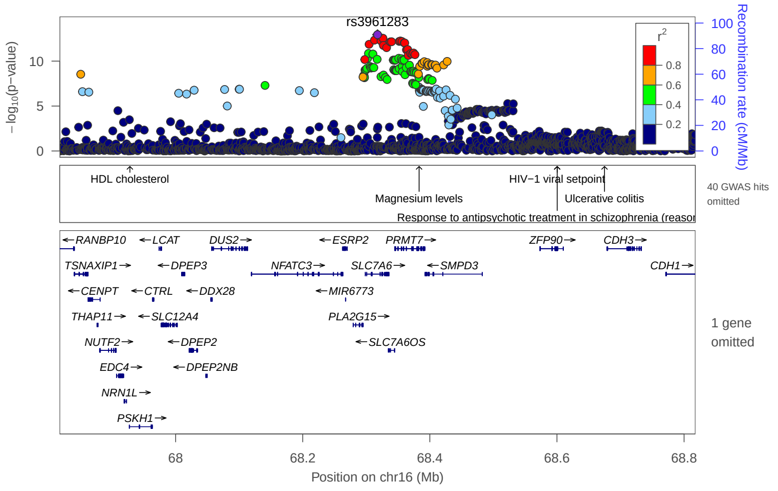

malate

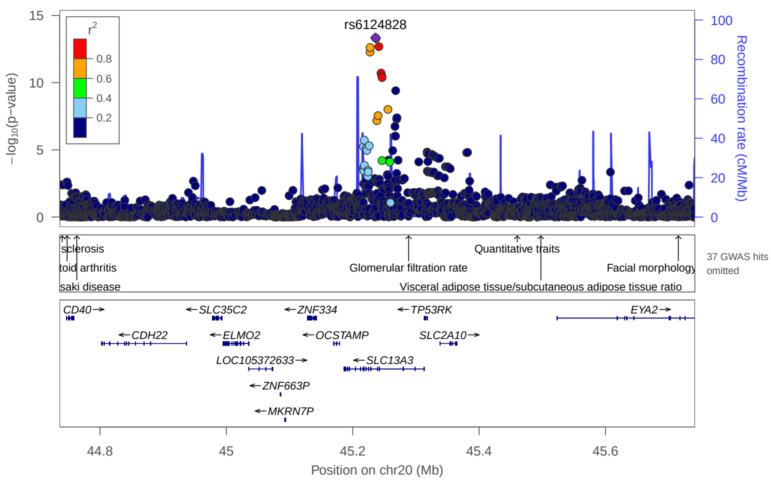

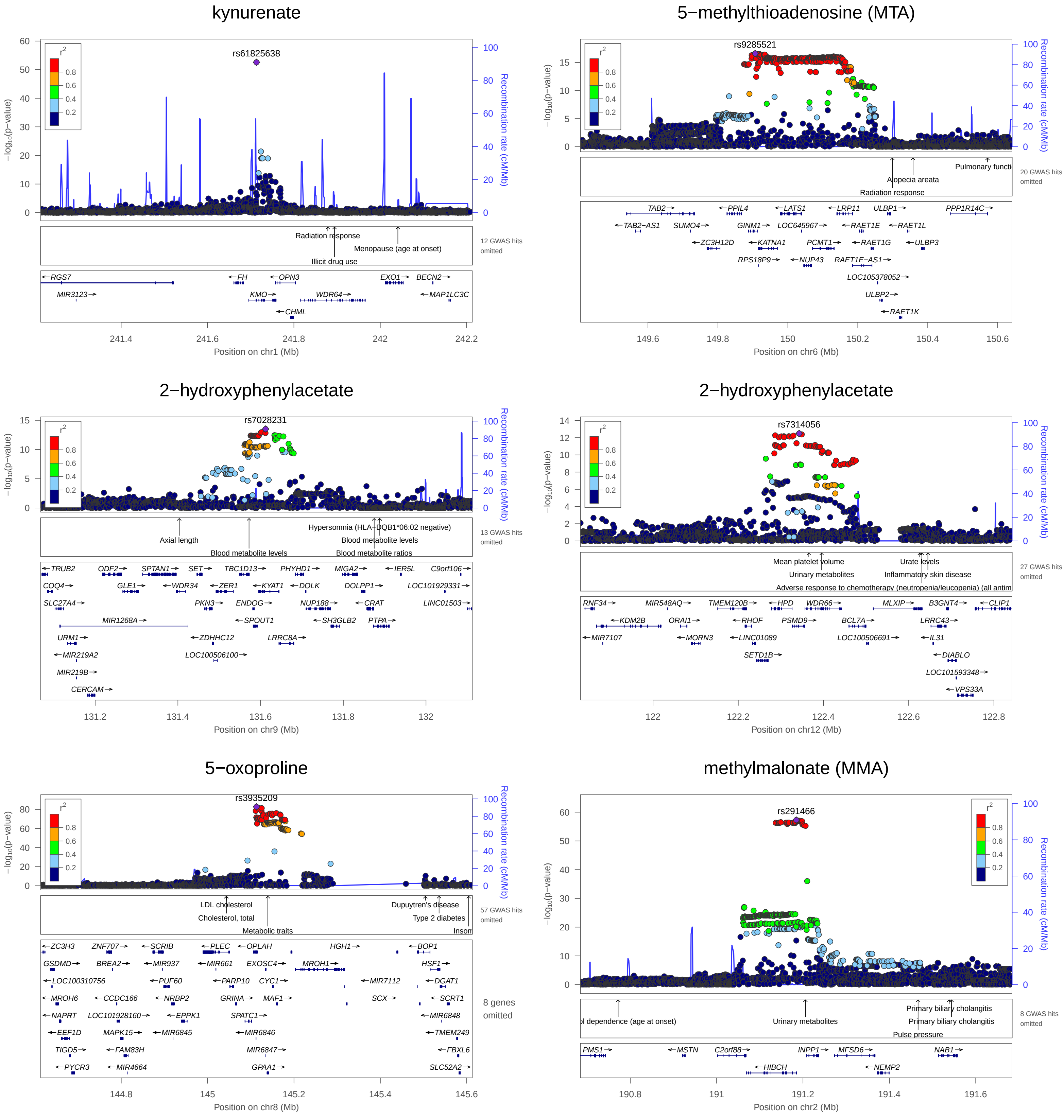

kynurenate
rs61825638
−log10(p−value)
Recombination rate (cM/Mb)
Radiation response
Menopause (age at onset)
Illicit drug use
12 GWAS hits omitted
RGS7
MIR3123
FH
OPN3
KMO
WDR64
CHML
EXO1
BECN2
MAP1LC3C
Position on chr1 (Mb)
5−methylthioadenosine (MTA)
rs9285521
Pulmonary functi
Alopecia areata
Radiation response
20 GWAS hits omitted
TAB2
TAB2−AS1
SUMO4
PPIL4
GINM1
ZC3H12D
KATNA1
RPS18P9
LATS1
LOC645967
PCMT1
NUP43
LRP11
RAET1E
RAET1G
RAET1E−AS1
LOC105378052
ULBP1
RAET1L
ULBP3
ULBP2
RAET1K
PPP1R14C
Position on chr6 (Mb)
2−hydroxyphenylacetate
rs7028231
Axial length
Blood metabolite levels
Hypersomnia (HLA−DQB1*06:02 negative)
Blood metabolite levels
Blood metabolite ratios
13 GWAS hits omitted
TRUB2
COQ4
SLC27A4
MIR1268A
URM1
MIR219A2
MIR219B
CERCAM
ODF2
GLE1
SPTAN1
WDR34
SET
PKN3
ZDHHC12
LOC100506100
TBC1D13
ZER1
ENDOG
SPOUT1
PHYHD1
KYAT1
DOLK
NUP188
SH3GLB2
LRRC8A
MIGA2
DOLPP1
CRAT
PTPA
IER5L
C9orf106
LOC101929331
LINC01503
Position on chr9 (Mb)
2−hydroxyphenylacetate
rs7314056
Mean platelet volume
Urinary metabolites
Urate levels
Inflammatory skin disease
Adverse response to chemotherapy (neutropenia/leucopenia) (all antim
27 GWAS hits omitted
RNF34
KDM2B
MIR7107
MIR548AQ
ORAI1
MORN3
TMEM120B
RHOF
LINC01089
SETD1B
HPD
PSMD9
WDR66
BCL7A
LOC100506691
MLXIP
B3GNT4
LRRC43
IL31
DIABLO
LOC101593348
VPS33A
CLIP1
Position on chr12 (Mb)
5−oxoproline
rs3935209
LDL cholesterol
Cholesterol, total
Metabolic traits
Dupuytren's disease
Type 2 diabetes
Insom
57 GWAS hits omitted
ZC3H3
GSDMD
LOC100310756
MROH6
NAPRT
EEF1D
TIGD5
PYCR3
ZNF707
BREA2
CCDC166
LOC101928160
MAPK15
FAM83H
MIR4664
SCRIB
MIR937
PUF60
NRBP2
EPPK1
MIR6845
PLEC
MIR661
PARP10
GRINA
SPATC1
MIR6846
MIR6847
GPAA1
OPLAH
EXOSC4
CYC1
MAF1
MROH1
HGH1
MIR7112
SCX
BOP1
HSF1
DGAT1
SCRT1
MIR6848
TMEM249
FBXL6
SLC52A2
8 genes omitted
Position on chr8 (Mb)
methylmalonate (MMA)
rs291466
ol dependence (age at onset)
Urinary metabolites
Primary biliary cholangitis
Primary biliary cholangitis
Pulse pressure
8 GWAS hits omitted
PMS1
MSTN
C2orf88
HIBCH
INPP1
MFSD6
NEMP2
NAB1
Position on chr2 (Mb)

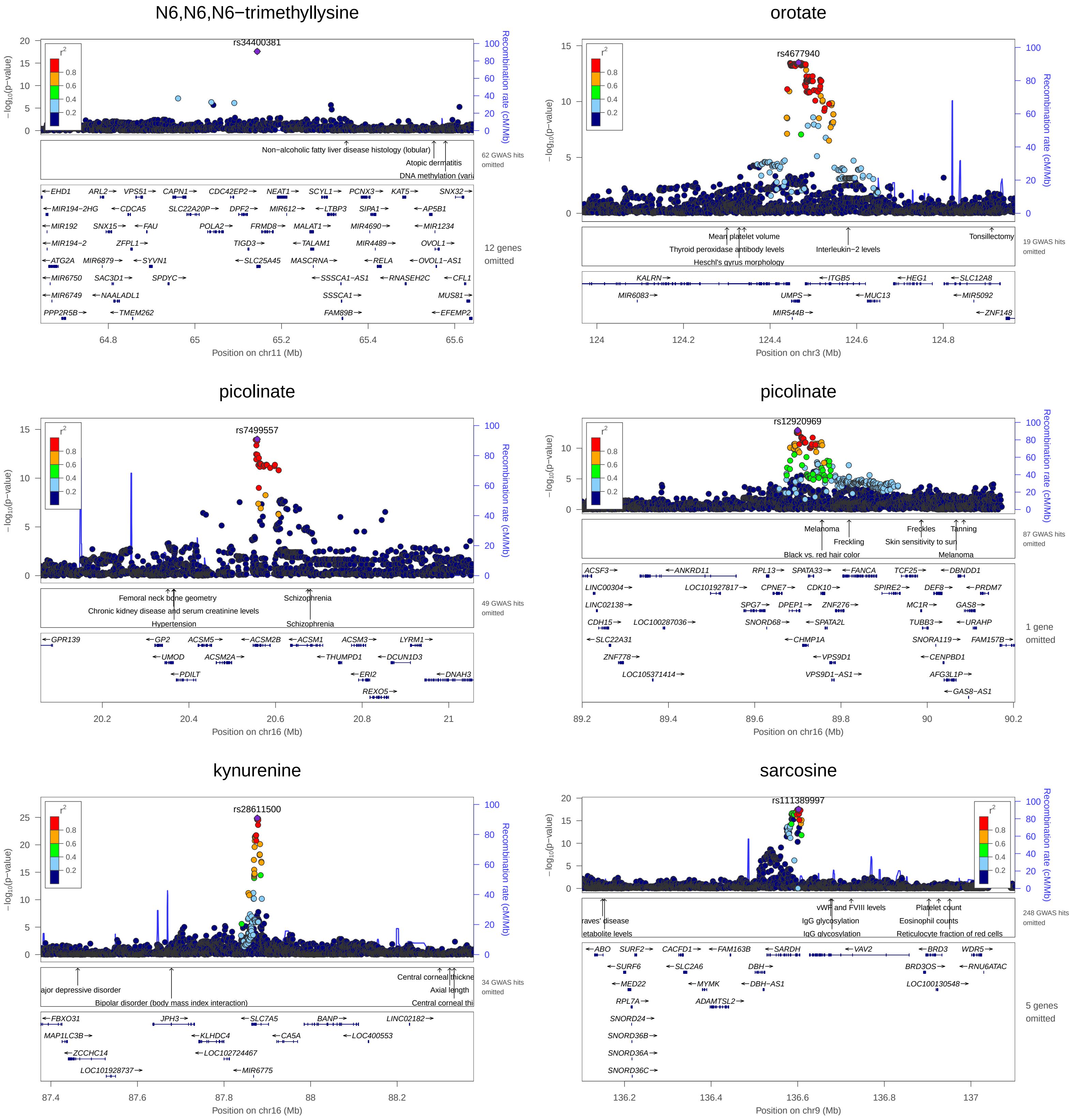
N6,N6,N6−trimethyllysine
rs34400381
Non−alcoholic fatty liver disease histology (lobular)
Atopic dermatitis
62 GWAS hits omitted
EHD1 ARL2 VPS51 CAPN1 CDC42EP2 NEAT1 SCYL1 PCNX3 KAT5 SNX32
MIR194−2HG CDCA5 SLC22A20P DPF2 MIR612 LTBP3 SIPA1 AP5B1
MIR192 SNX15 FAU POLA2 FRMD8 MALAT1 MIR4690 MIR1234
MIR194−2 ZFPL1 TIGD3 TALAM1 MIR4489 OVOL1
ATG2A MIR6879 SYVN1 SLC25A45 MASCRNA RELA OVOL1−AS1
MIR6750 SAC3D1 SPDYC SSSCA1−AS1 RNASEH2C CFL1
MIR6749 NAALADL1 SSSCA1 MUS81
PPP2R5B TMEM262 FAM89B EFEMP2
12 genes omitted
Position on chr11 (Mb)
orotate
rs4677940
Mean platelet volume
Thyroid peroxidase antibody levels
Heschl's gyrus morphology
Interleukin−2 levels
Tonsillectomy
19 GWAS hits omitted
KALRN ITGB5 HEG1 SLC12A8
MIR6083 UMPS MUC13 MIR5092
MIR544B ZNF148
Position on chr3 (Mb)
picolinate
rs7499557
Femoral neck bone geometry
Chronic kidney disease and serum creatinine levels
Hypertension
Schizophrenia
49 GWAS hits omitted
GPR139 GP2 ACSM5 ACSM2B ACSM1 ACSM3 LYRM1
UMOD ACSM2A THUMPD1 DCUN1D3
PDILT ERI2 DNAH3
REXO5
Position on chr16 (Mb)
picolinate
rs12920969
Melanoma
Freckling
Black vs. red hair color
Freckles
Skin sensitivity to sun
Tanning
87 GWAS hits omitted
ACSF3 ANKRD11 RPL13 SPATA33 FANCA TCF25 DBNDD1
LINC00304 LOC101927817 CPNE7 CDK10 SPIRE2 DEF8 PRDM7
LINC02138 SPG7 DPEP1 ZNF276 MC1R GAS8
CDH15 LOC100287036 SNORD68 SPATA2L TUBB3 URAHP
SLC22A31 CHMP1A SNORA119 FAM157B
ZNF778 VPS9D1 CENPBD1
LOC105371414 VPS9D1−AS1 AFG3L1P
GAS8−AS1
1 gene omitted
Position on chr16 (Mb)
kynurenine
rs28611500
Major depressive disorder
Bipolar disorder (body mass index interaction)
Central corneal thickness
Axial length
34 GWAS hits omitted
FBXO31 JPH3 SLC7A5 BANP LINC02182
MAP1LC3B KLHDC4 CA5A LOC400553
ZCCHC14 LOC102724467
LOC101928737 MIR6775
Position on chr16 (Mb)
sarcosine
rs111389997
Graves' disease
Metabolite levels
vWF and FVIII levels
IgG glycosylation
Platelet count
Eosinophil counts
Reticulocyte fraction of red cells
248 GWAS hits omitted
ABO SURF2 CACFD1 FAM163B SARDH VAV2 BRD3 WDR5
SURF6 SLC2A6 DBH BRD3OS RNU6ATAC
MED22 MYMK DBH−AS1 LOC100130548
RPL7A ADAMTSL2
SNORD24
SNORD36B
SNORD36A
SNORD36C
5 genes omitted
Position on chr9 (Mb)
−log10(p−value)
Recombination rate (cM/Mb)

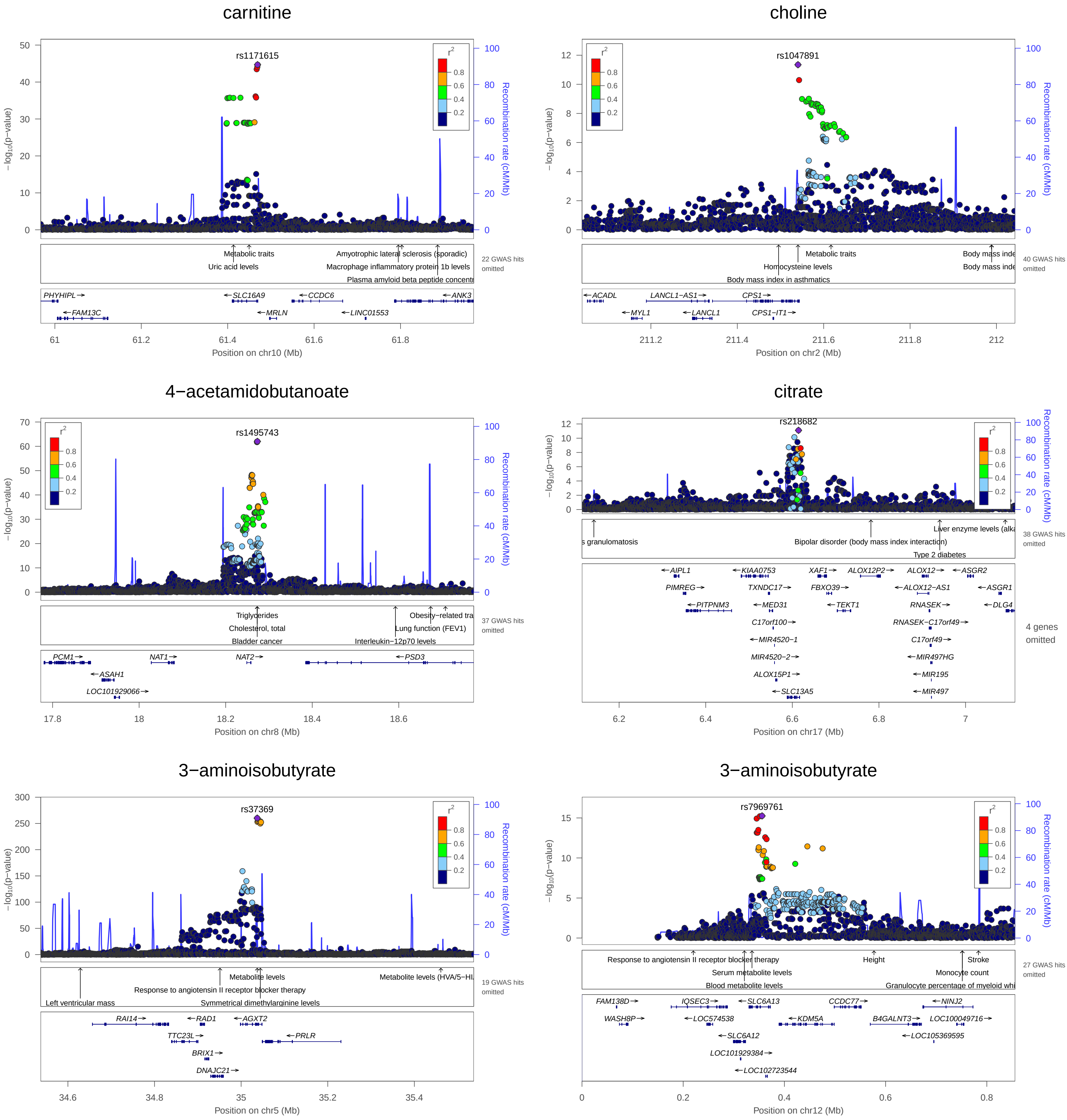

carnitine
rs1171615
−log10(p−value)
Recombination rate (cM/Mb)
Metabolic traits
Uric acid levels
Amyotrophic lateral sclerosis (sporadic)
Macrophage inflammatory protein 1b levels
22 GWAS hits omitted
PHYHIPL
FAM13C
SLC16A9
MRLN
CCDC6
LINC01553
ANK3
Position on chr10 (Mb)
choline
rs1047891
Metabolic traits
Homocysteine levels
Body mass index in asthmatics
40 GWAS hits omitted
ACADL
MYL1
LANCL1−AS1
LANCL1
CPS1
CPS1−IT1
Position on chr2 (Mb)
4−acetamidobutanoate
rs1495743
Triglycerides
Cholesterol, total
Bladder cancer
Lung function (FEV1)
Interleukin−12p70 levels
37 GWAS hits omitted
PCM1
ASAH1
LOC101929066
NAT1
NAT2
PSD3
Position on chr8 (Mb)
citrate
rs218682
Bipolar disorder (body mass index interaction)
Type 2 diabetes
38 GWAS hits omitted
AIPL1
PIMREG
PITPNM3
KIAA0753
TXNDC17
MED31
C17orf100
MIR4520−1
MIR4520−2
ALOX15P1
SLC13A5
XAF1
FBXO39
TEKT1
ALOX12P2
ALOX12
ALOX12−AS1
RNASEK
RNASEK−C17orf49
C17orf49
MIR497HG
MIR195
MIR497
ASGR2
ASGR1
DLG4
4 genes omitted
Position on chr17 (Mb)
3−aminoisobutyrate
rs37369
Left ventricular mass
Response to angiotensin II receptor blocker therapy
Metabolite levels
Symmetrical dimethylarginine levels
19 GWAS hits omitted
RAI14
TTC23L
BRIX1
DNAJC21
RAD1
AGXT2
PRLR
Position on chr5 (Mb)
3−aminoisobutyrate
rs7969761
Response to angiotensin II receptor blocker therapy
Serum metabolite levels
Blood metabolite levels
Height
Monocyte count
Stroke
27 GWAS hits omitted
FAM138D
WASH8P
IQSEC3
LOC574538
SLC6A12
LOC101929384
LOC102723544
SLC6A13
KDM5A
CCDC77
B4GALNT3
NINJ2
LOC100049716
LOC105369595
Position on chr12 (Mb)

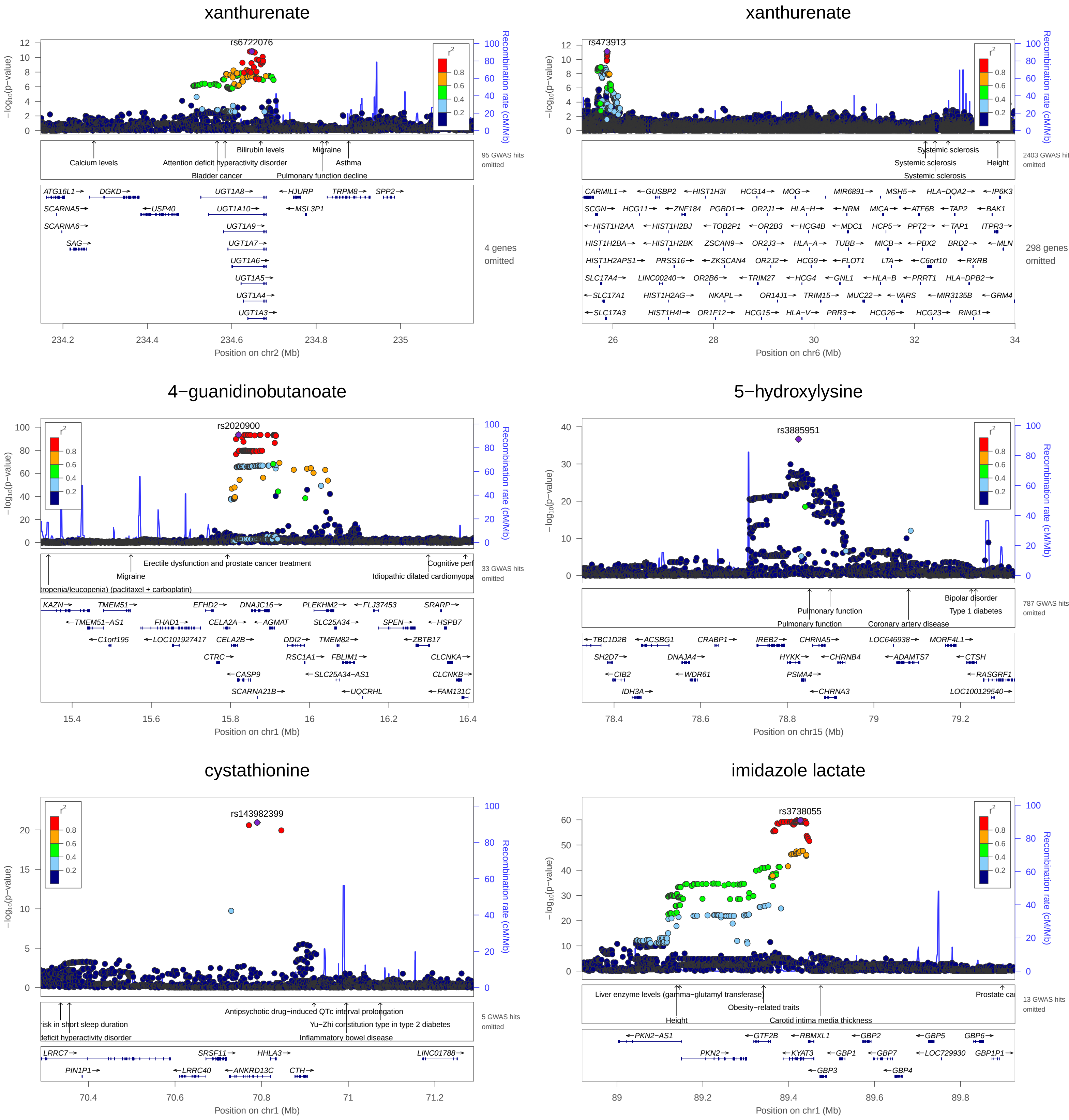

xanthurenate
rs6722076
−log10(p−value)
Recombination rate (cM/Mb)
Calcium levels
Attention deficit hyperactivity disorder
Bladder cancer
Bilirubin levels
Migraine
Asthma
Pulmonary function decline
95 GWAS hits omitted
ATG16L1 DGKD UGT1A8 HJURP TRPM8 SPP2
SCARNA5 USP40 UGT1A10 MSL3P1
SCARNA6 UGT1A9
SAG UGT1A7
UGT1A6
UGT1A5
UGT1A4
UGT1A3
4 genes omitted
234.2 234.4 234.6 234.8 235
Position on chr2 (Mb)
xanthurenate
rs473913
Systemic sclerosis
Systemic sclerosis
Systemic sclerosis
Height
2403 GWAS hits omitted
298 genes omitted
26 28 30 32 34
Position on chr6 (Mb)
4−guanidinobutanoate
rs2020900
Erectile dysfunction and prostate cancer treatment
Migraine
Cognitive perf
Idiopathic dilated cardiomyopa
33 GWAS hits omitted
KAZN TMEM51 EFHD2 DNAJC16 PLEKHM2 FLJ37453 SRARP
TMEM51−AS1 FHAD1 CELA2A AGMAT SLC25A34 SPEN HSPB7
C1orf195 LOC101927417 CELA2B DDI2 TMEM82 ZBTB17
CTRC RSC1A1 FBLIM1 CLCNKA
CASP9 SLC25A34−AS1 CLCNKB
SCARNA21B UQCRHL FAM131C
15.4 15.6 15.8 16 16.2 16.4
Position on chr1 (Mb)
5−hydroxylysine
rs3885951
Pulmonary function
Pulmonary function
Coronary artery disease
Bipolar disorder
Type 1 diabetes
787 GWAS hits omitted
TBC1D2B ACSBG1 CRABP1 IREB2 CHRNA5 LOC646938 MORF4L1
SH2D7 DNAJA4 HYKK CHRNB4 ADAMTS7 CTSH
CIB2 WDR61 PSMA4 RASGRF1
IDH3A CHRNA3 LOC100129540
78.4 78.6 78.8 79 79.2
Position on chr15 (Mb)
cystathionine
rs143982399
Antipsychotic drug−induced QTc interval prolongation
Yu−Zhi constitution type in type 2 diabetes
Inflammatory bowel disease
5 GWAS hits omitted
LRRC7 SRSF11 HHLA3 LINC01788
PIN1P1 LRRC40 ANKRD13C CTH
70.4 70.6 70.8 71 71.2
Position on chr1 (Mb)
imidazole lactate
rs3738055
Liver enzyme levels (gamma−glutamyl transferase)
Height
Obesity−related traits
Carotid intima media thickness
13 GWAS hits omitted
PKN2−AS1 GTF2B RBMXL1 GBP2 GBP5 GBP6
PKN2 KYAT3 GBP1 GBP7 LOC729930 GBP1P1
GBP3 GBP4
89 89.2 89.4 89.6 89.8
Position on chr1 (Mb)

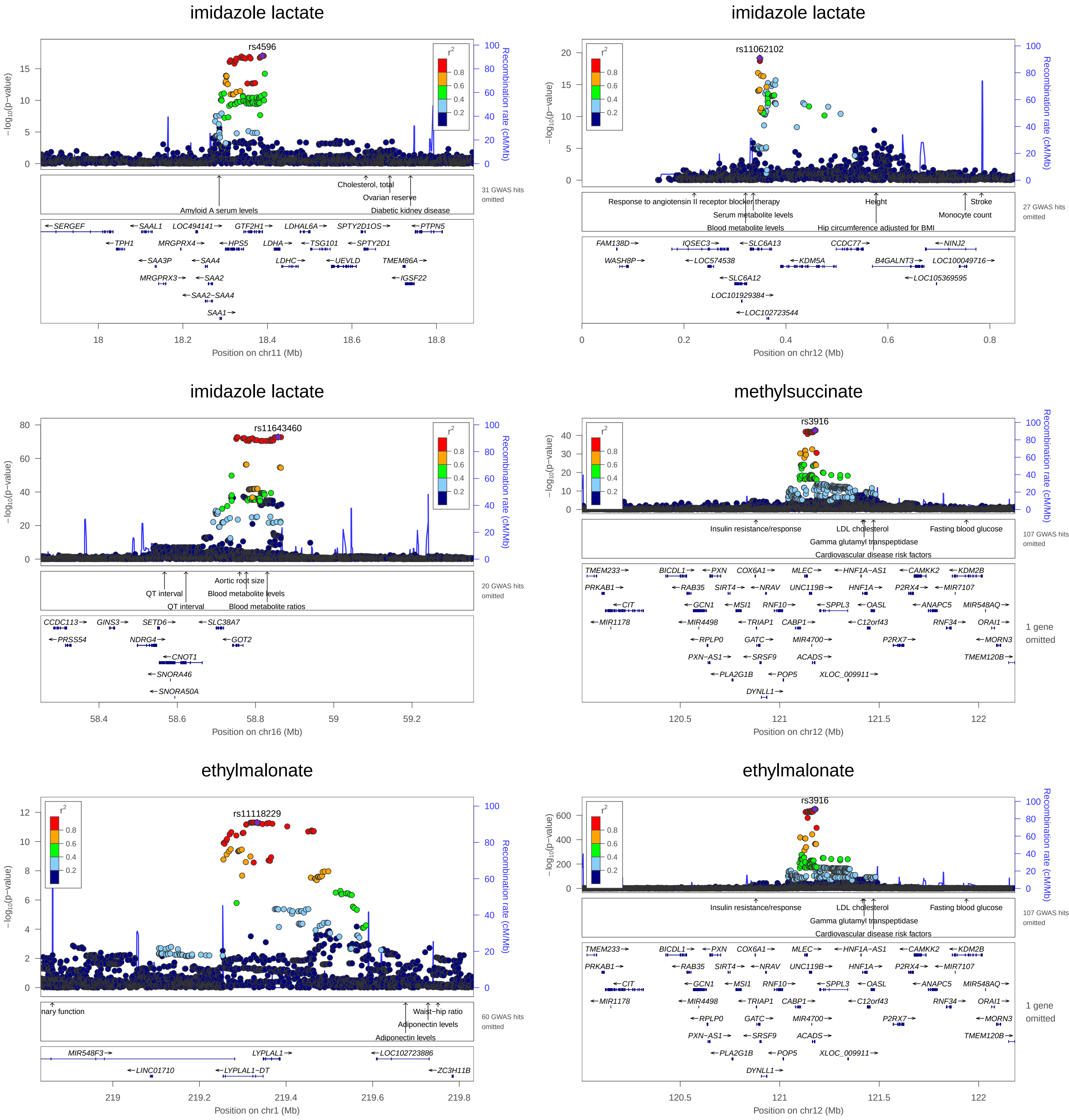
imidazole lactate
rs4596
−log10(p−value)
Recombination rate (cM/Mb)
Cholesterol, total
Ovarian reserve
Amyloid A serum levels
Diabetic kidney disease
31 GWAS hits omitted
SERGEF SAAL1 LOC494141 GTF2H1 LDHAL6A SPTY2D1OS PTPN5
TPH1 MRGPRX4 HPS5 LDHA TSG101 SPTY2D1
SAA3P SAA4 LDHC UEVLD TMEM86A
MRGPRX3 SAA2 IGSF22
SAA2−SAA4
SAA1
Position on chr11 (Mb)
imidazole lactate
rs11062102
Response to angiotensin II receptor blocker therapy
Height
Stroke
Serum metabolite levels
Monocyte count
Blood metabolite levels
Hip circumference adjusted for BMI
27 GWAS hits omitted
FAM138D IQSEC3 SLC6A13 CCDC77 NINJ2
WASH8P LOC574538 KDM5A B4GALNT3 LOC100049716
SLC6A12 LOC105369595
LOC101929384
LOC102723544
Position on chr12 (Mb)
imidazole lactate
rs11643460
Aortic root size
QT interval
Blood metabolite levels
QT interval
Blood metabolite ratios
20 GWAS hits omitted
CCDC113 GINS3 SETD6 SLC38A7
PRSS54 NDRG4 GOT2
CNOT1
SNORA46
SNORA50A
Position on chr16 (Mb)
methylsuccinate
rs3916
Insulin resistance/response
LDL cholesterol
Fasting blood glucose
Gamma glutamyl transpeptidase
Cardiovascular disease risk factors
107 GWAS hits omitted
TMEM233 BICDL1 PXN COX6A1 MLEC HNF1A−AS1 CAMKK2 KDM2B
PRKAB1 RAB35 SIRT4 NRAV UNC119B HNF1A P2RX4 MIR7107
CIT GCN1 MSI1 RNF10 SPPL3 OASL ANAPC5 MIR548AQ
MIR1178 MIR4498 TRIAP1 CABP1 C12orf43 RNF34 ORAI1
RPLP0 GATC MIR4700 P2RX7 MORN3
PXN−AS1 SRSF9 ACADS TMEM120B
PLA2G1B POP5 XLOC_009911
DYNLL1
1 gene omitted
Position on chr12 (Mb)
ethylmalonate
rs11118229
nary function
Waist−hip ratio
Adiponectin levels
Adiponectin levels
60 GWAS hits omitted
MIR548F3 LYPLAL1 LOC102723886
LINC01710 LYPLAL1−DT ZC3H11B
Position on chr1 (Mb)
ethylmalonate
rs3916
Insulin resistance/response
LDL cholesterol
Fasting blood glucose
Gamma glutamyl transpeptidase
Cardiovascular disease risk factors
107 GWAS hits omitted
1 gene omitted
Position on chr12 (Mb)

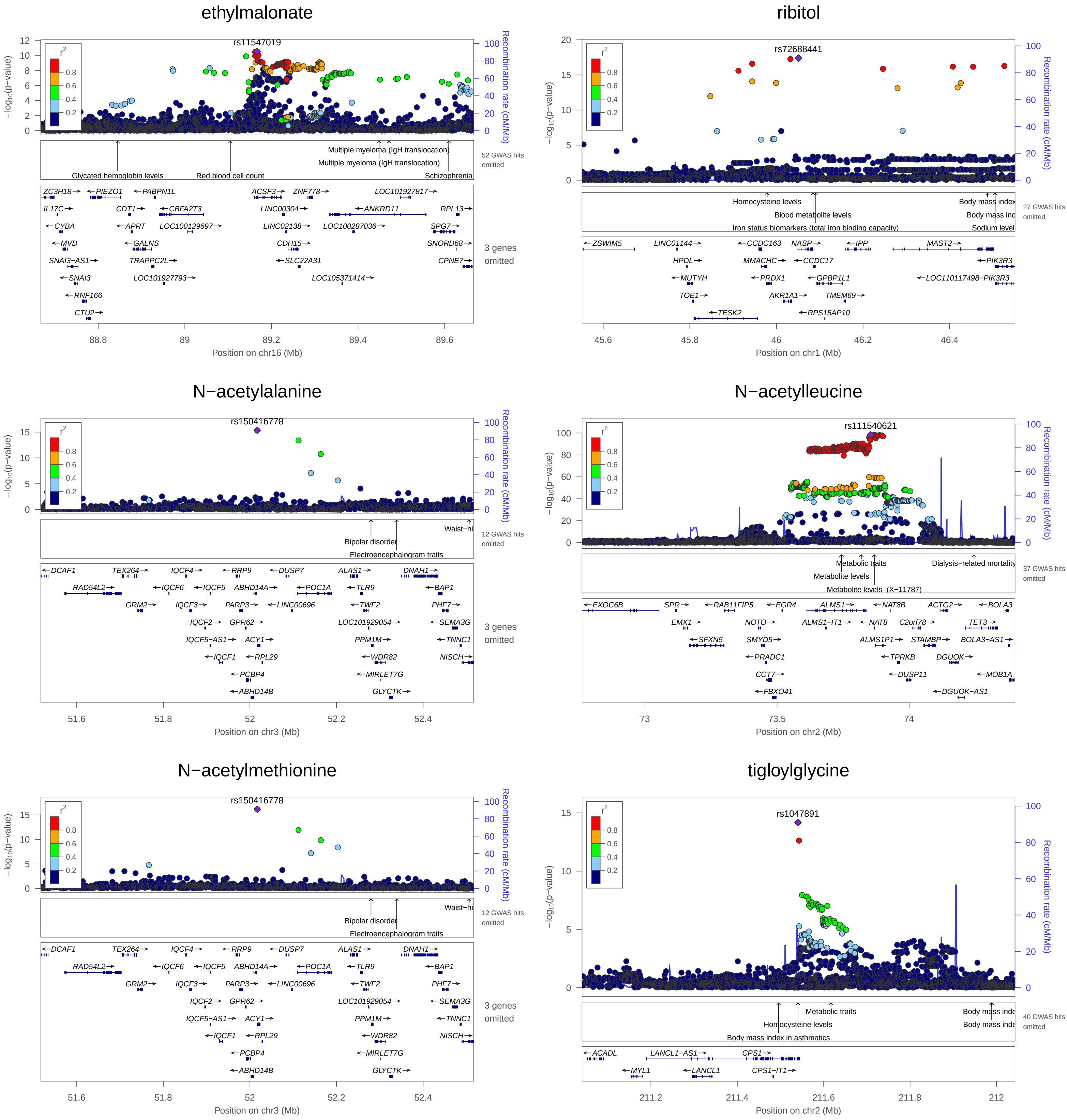

ethylmalonate
rs11547019
−log10(p−value)
Recombination rate (cM/Mb)
r2
Glycated hemoglobin levels
Red blood cell count
Multiple myeloma (IgH translocation)
Multiple myeloma (IgH translocation)
Schizophrenia
52 GWAS hits omitted
ZC3H18 PIEZO1 PABPN1L ACSF3 ZNF778 LOC101927817
IL17C CDT1 CBFA2T3 LINC00304 ANKRD11 RPL13
CYBA APRT LOC100129697 LINC02138 LOC100287036 SPG7
MVD GALNS CDH15 SNORD68
SNAI3−AS1 TRAPPC2L SLC22A31 CPNE7
SNAI3 LOC101927793 LOC105371414
RNF166
CTU2
3 genes omitted
Position on chr16 (Mb)
ribitol
rs72688441
Homocysteine levels
Blood metabolite levels
Iron status biomarkers (total iron binding capacity)
Body mass index
Sodium level
27 GWAS hits omitted
ZSWIM5 LINC01144 CCDC163 NASP IPP MAST2
HPDL MMACHC CCDC17 PIK3R3
MUTYH PRDX1 GPBP1L1 LOC110117498−PIK3R3
TOE1 AKR1A1 TMEM69
TESK2 RPS15AP10
Position on chr1 (Mb)
N−acetylalanine
rs150416778
Bipolar disorder
Electroencephalogram traits
Waist−hi
12 GWAS hits omitted
DCAF1 TEX264 IQCF4 RRP9 DUSP7 ALAS1 DNAH1
RAD54L2 IQCF6 IQCF5 ABHD14A POC1A TLR9 BAP1
GRM2 IQCF3 PARP3 LINC00696 TWF2 PHF7
IQCF2 GPR62 LOC101929054 SEMA3G
IQCF5−AS1 ACY1 PPM1M TNNC1
IQCF1 RPL29 WDR82 NISCH
PCBP4 MIRLET7G
ABHD14B GLYCTK
3 genes omitted
Position on chr3 (Mb)
N−acetylleucine
rs111540621
Metabolic traits
Metabolite levels
Metabolite levels (X−11787)
Dialysis−related mortality
37 GWAS hits omitted
EXOC6B SPR RAB11FIP5 EGR4 ALMS1 NAT8B ACTG2 BOLA3
EMX1 NOTO ALMS1−IT1 NAT8 C2orf78 TET3
SFXN5 SMYD5 ALMS1P1 STAMBP BOLA3−AS1
PRADC1 TPRKB DGUOK
CCT7 DUSP11 MOB1A
FBXO41 DGUOK−AS1
Position on chr2 (Mb)
N−acetylmethionine
rs150416778
Bipolar disorder
Electroencephalogram traits
Waist−hi
12 GWAS hits omitted
3 genes omitted
Position on chr3 (Mb)
tigloylglycine
rs1047891
Metabolic traits
Homocysteine levels
Body mass index in asthmatics
40 GWAS hits omitted
ACADL LANCL1−AS1 CPS1
MYL1 LANCL1 CPS1−IT1
Position on chr2 (Mb)

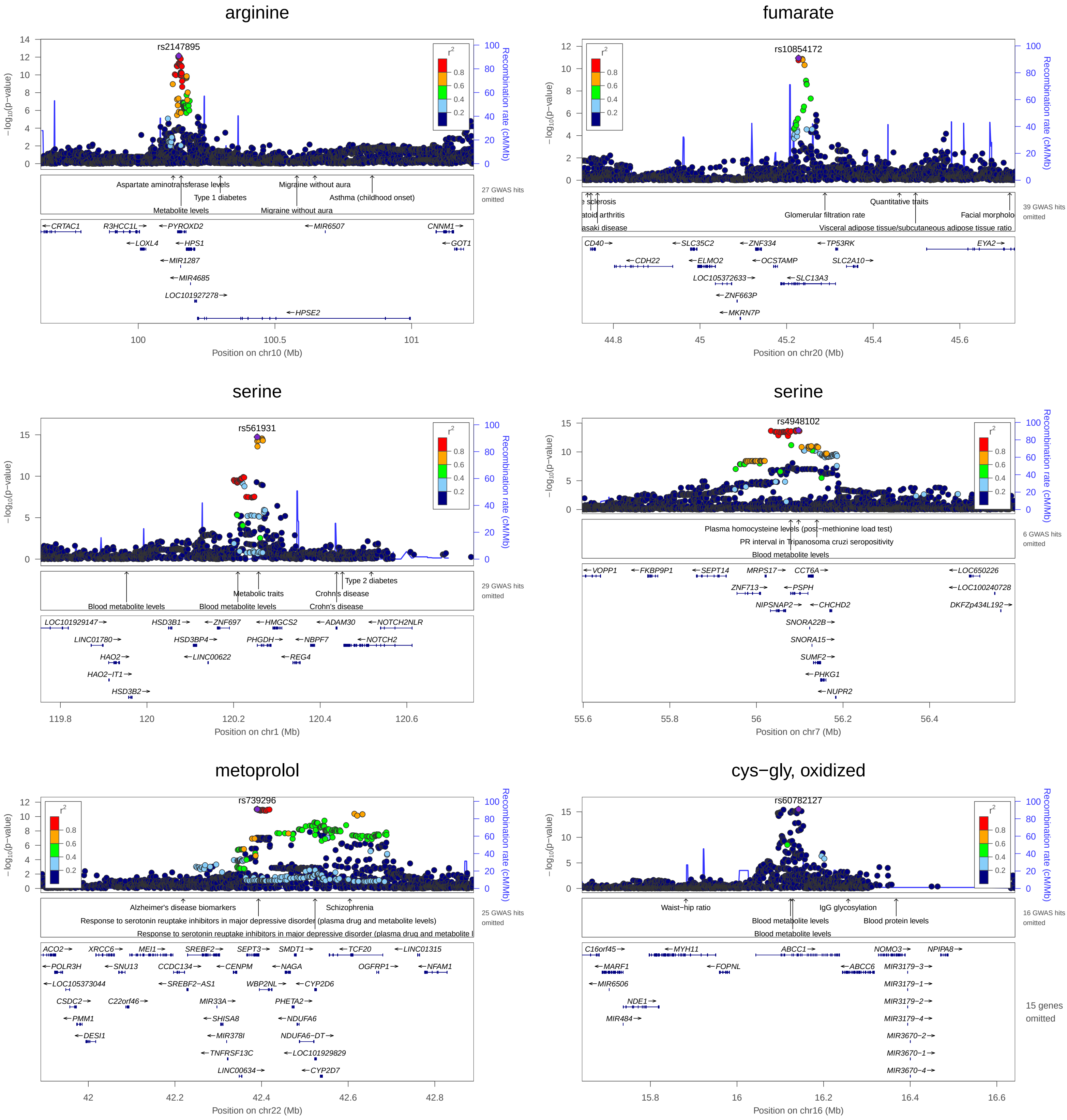

arginine
rs2147895
−log10(p−value)
Recombination rate (cM/Mb)
r2
Aspartate aminotransferase levels
Type 1 diabetes
Metabolite levels
Migraine without aura
Asthma (childhood onset)
Migraine without aura
27 GWAS hits omitted
CRTAC1
R3HCC1L
PYROXD2
MIR6507
CNNM1
LOXL4
HPS1
GOT1
MIR1287
MIR4685
LOC101927278
HPSE2
Position on chr10 (Mb)
fumarate
rs10854172
Glomerular filtration rate
Quantitative traits
Facial morpholo
Visceral adipose tissue/subcutaneous adipose tissue ratio
39 GWAS hits omitted
CD40
SLC35C2
ZNF334
TP53RK
EYA2
CDH22
ELMO2
OCSTAMP
SLC2A10
LOC105372633
SLC13A3
ZNF663P
MKRN7P
Position on chr20 (Mb)
serine
rs561931
Blood metabolite levels
Metabolic traits
Blood metabolite levels
Type 2 diabetes
Crohn's disease
Crohn's disease
29 GWAS hits omitted
LOC101929147
HSD3B1
ZNF697
HMGCS2
ADAM30
NOTCH2NLR
LINC01780
HSD3BP4
PHGDH
NBPF7
NOTCH2
HAO2
LINC00622
REG4
HAO2−IT1
HSD3B2
Position on chr1 (Mb)
serine
rs4948102
Plasma homocysteine levels (post−methionine load test)
PR interval in Tripanosoma cruzi seropositivity
Blood metabolite levels
6 GWAS hits omitted
VOPP1
FKBP9P1
SEPT14
MRPS17
CCT6A
LOC650226
ZNF713
PSPH
LOC100240728
NIPSNAP2
CHCHD2
DKFZp434L192
SNORA22B
SNORA15
SUMF2
PHKG1
NUPR2
Position on chr7 (Mb)
metoprolol
rs739296
Alzheimer's disease biomarkers
Schizophrenia
Response to serotonin reuptake inhibitors in major depressive disorder (plasma drug and metabolite levels)
25 GWAS hits omitted
ACO2
XRCC6
MEI1
SREBF2
SEPT3
SMDT1
TCF20
LINC01315
POLR3H
SNU13
CCDC134
CENPM
NAGA
OGFRP1
NFAM1
LOC105373044
SREBF2−AS1
WBP2NL
CYP2D6
CSDC2
C22orf46
MIR33A
PHETA2
PMM1
SHISA8
NDUFA6
DESI1
MIR378I
NDUFA6−DT
TNFRSF13C
LOC101929829
LINC00634
CYP2D7
Position on chr22 (Mb)
cys−gly, oxidized
rs60782127
Waist−hip ratio
IgG glycosylation
Blood metabolite levels
Blood protein levels
Blood metabolite levels
16 GWAS hits omitted
C16orf45
MYH11
ABCC1
NOMO3
NPIPA8
MARF1
FOPNL
ABCC6
MIR3179−3
MIR6506
MIR3179−1
NDE1
MIR3179−2
MIR484
MIR3179−4
MIR3670−2
MIR3670−1
MIR3670−4
15 genes omitted
Position on chr16 (Mb)

cys−gly, oxidized

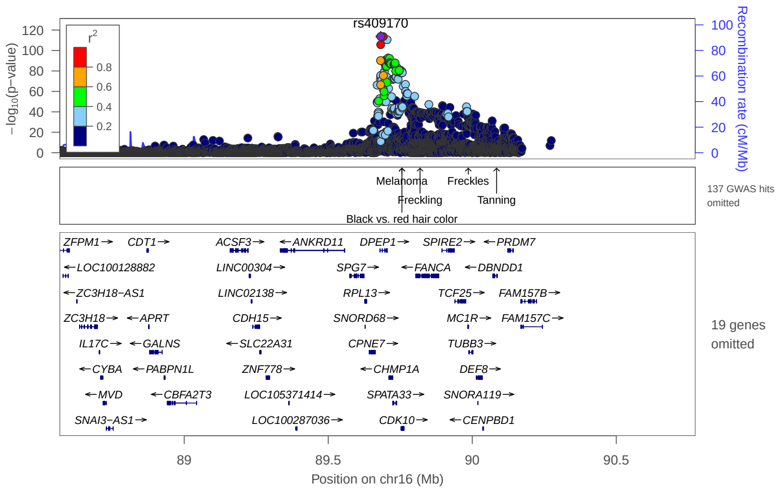

eicosapentaenoate (EPA; 20:5n3)

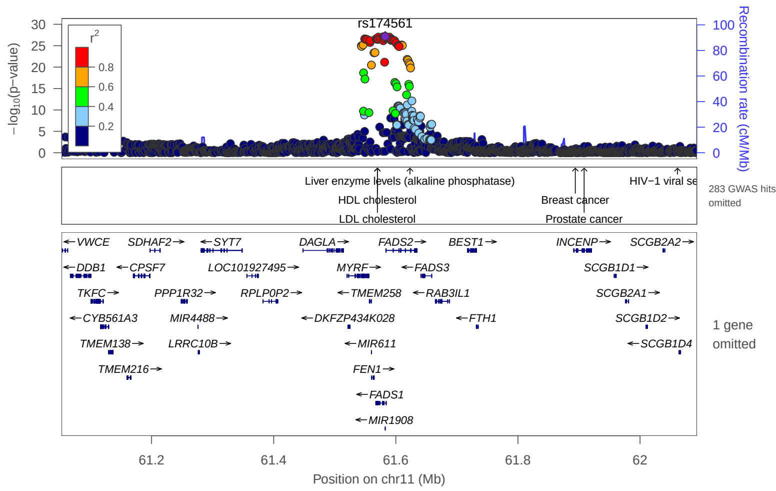

proline

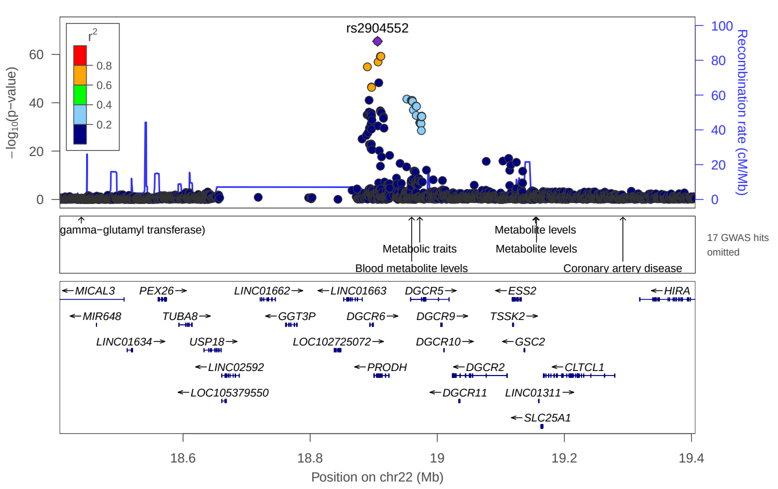

1,2−dipalmitoyl−GPC (16:0/16:0)

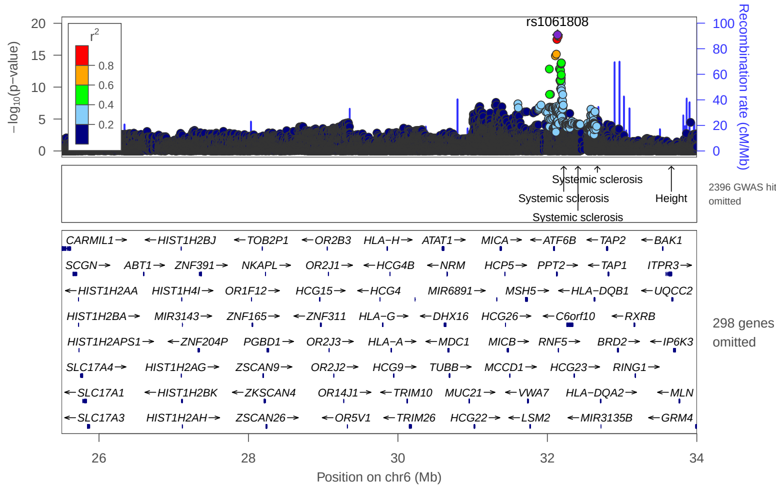

1,2−dipalmitoyl−GPC (16:0/16:0)

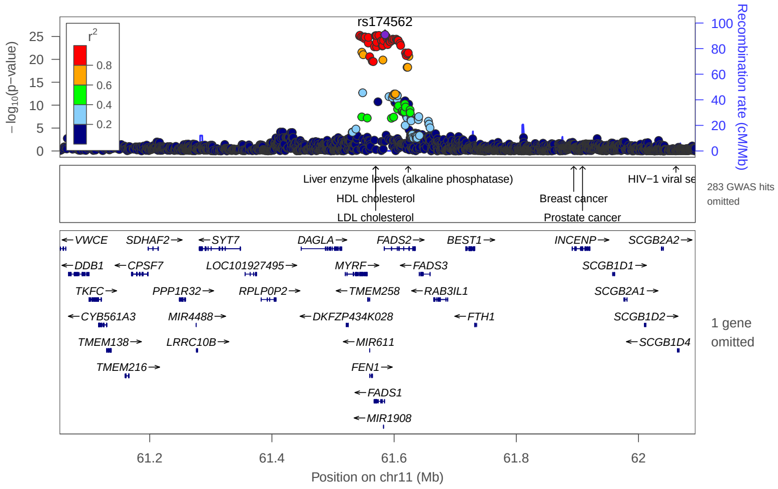

1−palmitoyl−2−oleoyl−GPE (16:0/18:1)

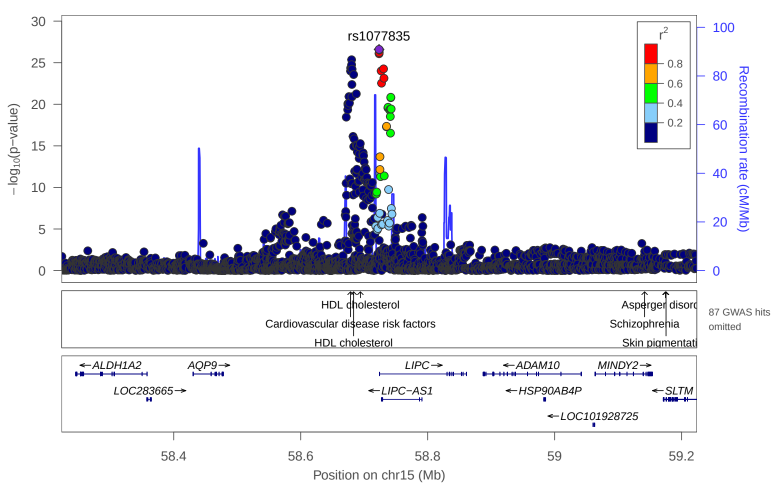

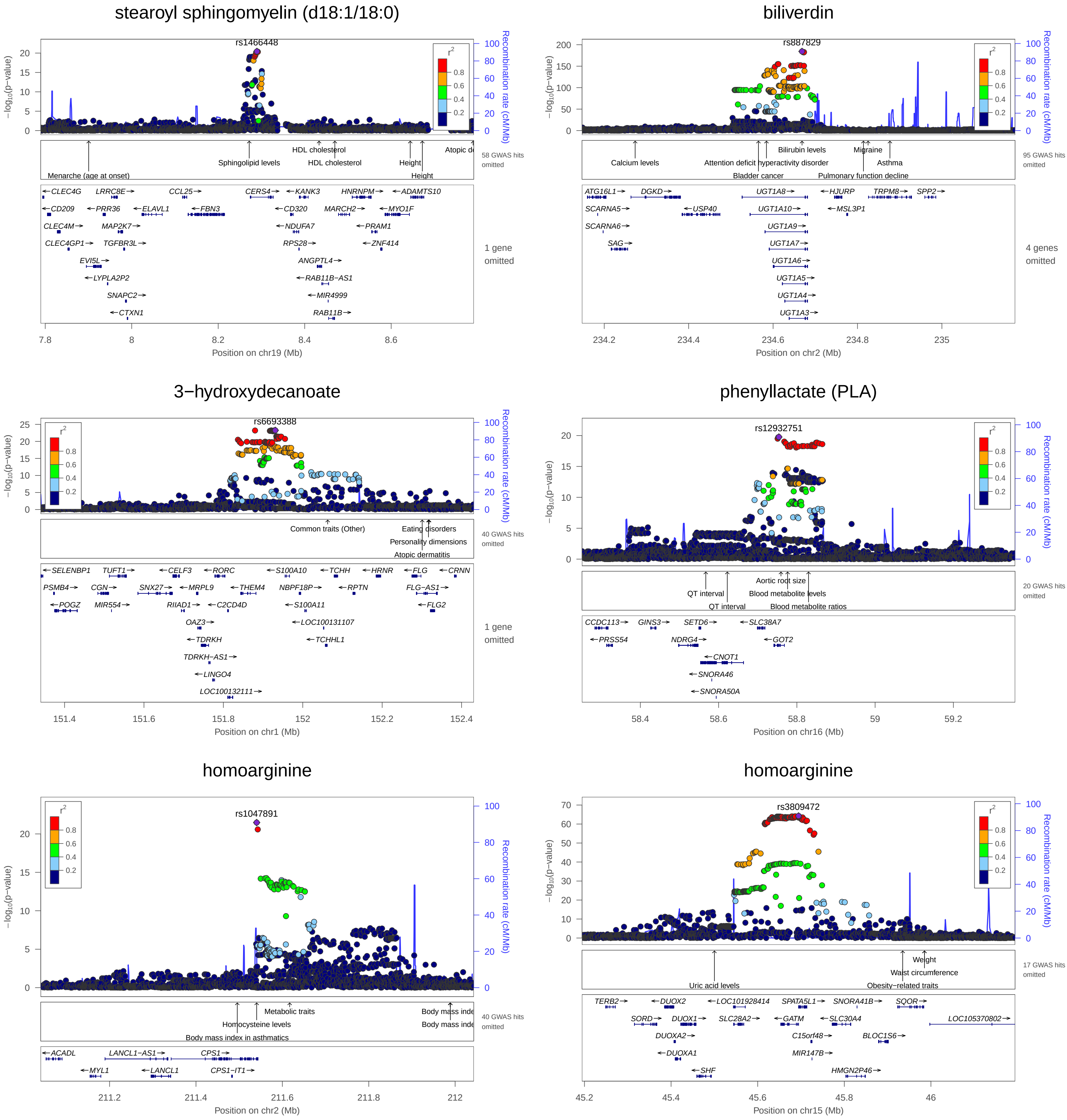
stearoyl sphingomyelin (d18:1/18:0)
rs1466448
−log10(p−value)
Recombination rate (cM/Mb)
r2
58 GWAS hits omitted
Menarche (age at onset)
Sphingolipid levels
HDL cholesterol
HDL cholesterol
Height
Height
CLEC4G
LRRC8E
CCL25
CERS4
KANK3
HNRNPM
ADAMTS10
CD209
PRR36
ELAVL1
FBN3
CD320
MARCH2
MYO1F
CLEC4M
MAP2K7
NDUFA7
PRAM1
CLEC4GP1
TGFBR3L
RPS28
ZNF414
EVI5L
ANGPTL4
LYPLA2P2
RAB11B−AS1
SNAPC2
MIR4999
CTXN1
RAB11B
1 gene omitted
Position on chr19 (Mb)
biliverdin
rs887829
95 GWAS hits omitted
Calcium levels
Attention deficit hyperactivity disorder
Bladder cancer
Bilirubin levels
Migraine
Asthma
Pulmonary function decline
ATG16L1
DGKD
UGT1A8
HJURP
TRPM8
SPP2
SCARNA5
USP40
UGT1A10
MSL3P1
SCARNA6
UGT1A9
SAG
UGT1A7
UGT1A6
UGT1A5
UGT1A4
UGT1A3
4 genes omitted
Position on chr2 (Mb)
3−hydroxydecanoate
rs6693388
40 GWAS hits omitted
Common traits (Other)
Eating disorders
Personality dimensions
Atopic dermatitis
SELENBP1
TUFT1
CELF3
RORC
S100A10
TCHH
HRNR
FLG
CRNN
PSMB4
CGN
SNX27
MRPL9
THEM4
NBPF18P
RPTN
FLG−AS1
POGZ
MIR554
RIIAD1
C2CD4D
S100A11
FLG2
OAZ3
LOC100131107
TDRKH
TCHHL1
TDRKH−AS1
LINGO4
LOC100132111
1 gene omitted
Position on chr1 (Mb)
phenyllactate (PLA)
rs12932751
20 GWAS hits omitted
QT interval
QT interval
Aortic root size
Blood metabolite levels
Blood metabolite ratios
CCDC113
GINS3
SETD6
SLC38A7
PRSS54
NDRG4
GOT2
CNOT1
SNORA46
SNORA50A
Position on chr16 (Mb)
homoarginine
rs1047891
40 GWAS hits omitted
Metabolic traits
Homocysteine levels
Body mass index in asthmatics
ACADL
LANCL1−AS1
CPS1
MYL1
LANCL1
CPS1−IT1
Position on chr2 (Mb)
homoarginine
rs3809472
17 GWAS hits omitted
Uric acid levels
Weight
Waist circumference
Obesity−related traits
TERB2
DUOX2
LOC101928414
SPATA5L1
SNORA41B
SQOR
SORD
DUOX1
SLC28A2
GATM
SLC30A4
LOC105370802
DUOXA2
C15orf48
BLOC1S6
DUOXA1
MIR147B
SHF
HMGN2P46
Position on chr15 (Mb)

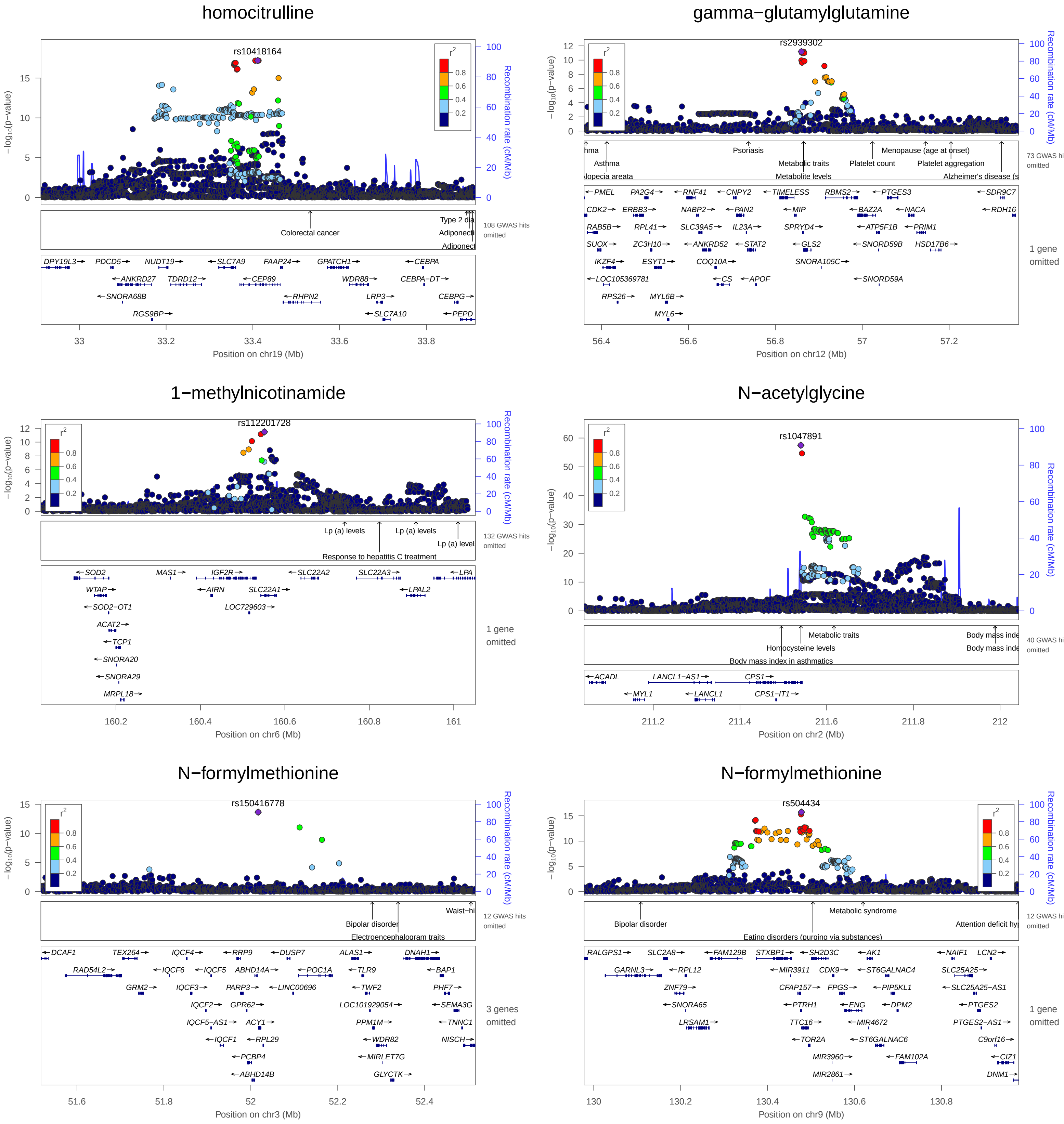

homocitrulline
rs10418164
−log10(p−value)
Recombination rate (cM/Mb)
r2
0.8
0.6
0.4
0.2
Colorectal cancer
Type 2 dia
Adiponecti
Adiponect
108 GWAS hits omitted
DPY19L3 PDCD5 NUDT19 SLC7A9 FAAP24 GPATCH1 CEBPA
ANKRD27 TDRD12 CEP89 WDR88 CEBPA−DT
SNORA68B RHPN2 LRP3 CEBPG
RGS9BP SLC7A10 PEPD
Position on chr19 (Mb)
gamma−glutamylglutamine
rs2939302
Asthma
Psoriasis
Metabolic traits
Platelet count
Menopause (age at onset)
Platelet aggregation
Metabolite levels
Alzheimer's disease
73 GWAS hits omitted
PMEL PA2G4 RNF41 CNPY2 TIMELESS RBMS2 PTGES3 SDR9C7
CDK2 ERBB3 NABP2 PAN2 MIP BAZ2A NACA RDH16
RAB5B RPL41 SLC39A5 IL23A SPRYD4 ATP5F1B PRIM1
SUOX ZC3H10 ANKRD52 STAT2 GLS2 SNORD59B HSD17B6
IKZF4 ESYT1 COQ10A SNORA105C
LOC105369781 CS APOF SNORD59A
RPS26 MYL6B
MYL6
1 gene omitted
Position on chr12 (Mb)
1−methylnicotinamide
rs112201728
Lp (a) levels
Lp (a) levels
Lp (a) levels
Response to hepatitis C treatment
132 GWAS hits omitted
SOD2 MAS1 IGF2R SLC22A2 SLC22A3 LPA
WTAP AIRN SLC22A1 LPAL2
SOD2−OT1 LOC729603
ACAT2
TCP1
SNORA20
SNORA29
MRPL18
1 gene omitted
Position on chr6 (Mb)
N−acetylglycine
rs1047891
Metabolic traits
Homocysteine levels
Body mass index in asthmatics
Body mass inde
Body mass inde
40 GWAS hits omitted
ACADL LANCL1−AS1 CPS1
MYL1 LANCL1 CPS1−IT1
Position on chr2 (Mb)
N−formylmethionine
rs150416778
Bipolar disorder
Electroencephalogram traits
Waist−hi
12 GWAS hits omitted
DCAF1 TEX264 IQCF4 RRP9 DUSP7 ALAS1 DNAH1
RAD54L2 IQCF6 IQCF5 ABHD14A POC1A TLR9 BAP1
GRM2 IQCF3 PARP3 LINC00696 TWF2 PHF7
IQCF2 GPR62 LOC101929054 SEMA3G
IQCF5−AS1 ACY1 PPM1M TNNC1
IQCF1 RPL29 WDR82 NISCH
PCBP4 MIRLET7G
ABHD14B GLYCTK
3 genes omitted
Position on chr3 (Mb)
N−formylmethionine
rs504434
Bipolar disorder
Metabolic syndrome
Eating disorders (purging via substances)
Attention deficit hyp
12 GWAS hits omitted
RALGPS1 SLC2A8 FAM129B STXBP1 SH2D3C AK1 NAIF1 LRP2
GARNL3 RPL12 MIR3911 CDK9 ST6GALNAC4 SLC25A25
ZNF79 CFAP157 FPGS PIP5KL1 SLC25A25−AS1
SNORA65 PTRH1 ENG DPM2 PTGES2
LRSAM1 TTC16 MIR4672 PTGES2−AS1
TOR2A ST6GALNAC6 C9orf16
MIR3960 FAM102A CIZ1
MIR2861 DNM1
1 gene omitted
Position on chr9 (Mb)

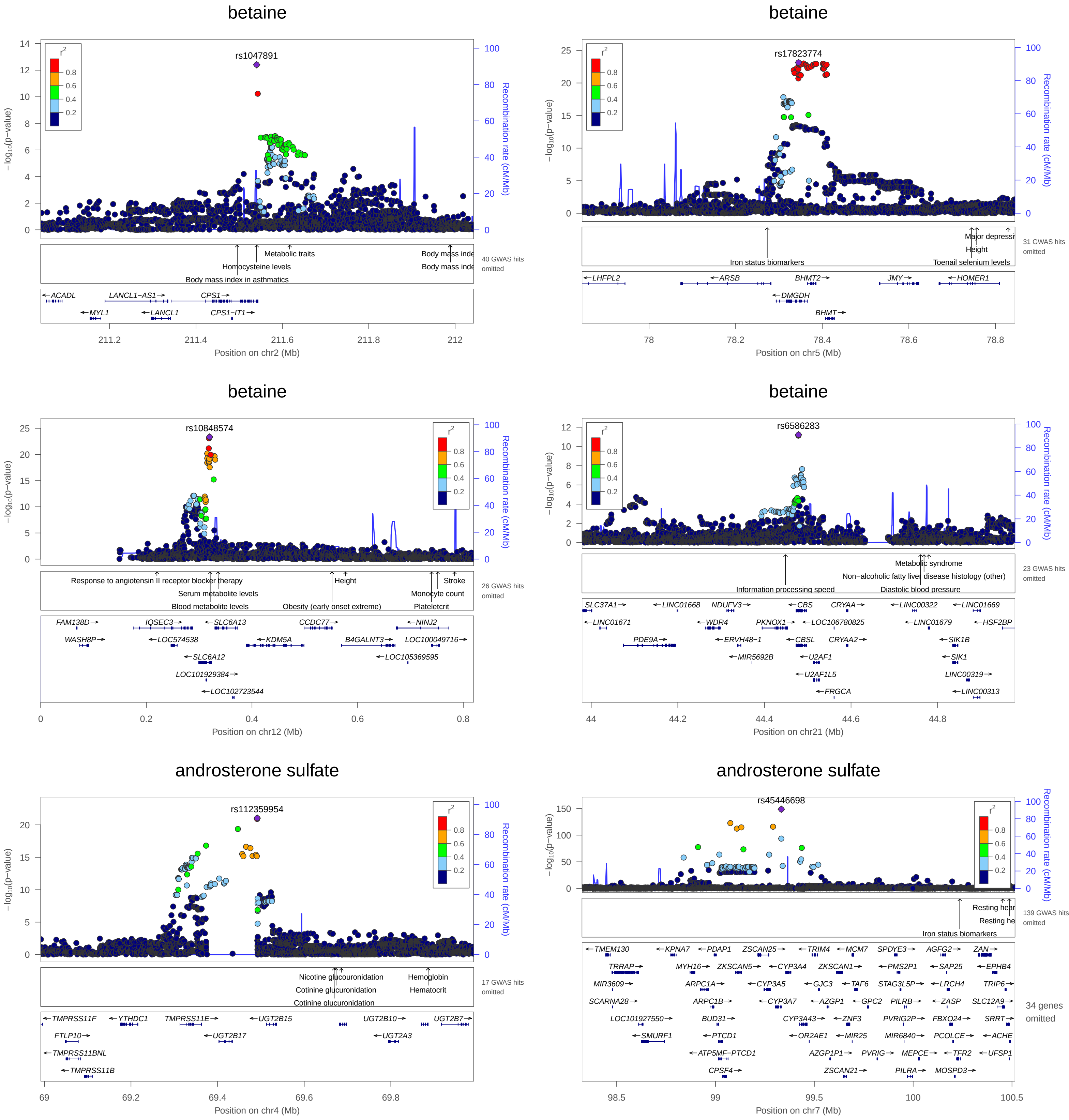

betaine
rs1047891
−log10(p−value)
Recombination rate (cM/Mb)
Metabolic traits
Homocysteine levels
Body mass index in asthmatics
Body mass inde
Body mass inde
40 GWAS hits omitted
ACADL
LANCL1−AS1
CPS1
MYL1
LANCL1
CPS1−IT1
Position on chr2 (Mb)
betaine
rs17823774
Iron status biomarkers
Major depressi
Height
Toenail selenium levels
31 GWAS hits omitted
LHFPL2
ARSB
BHMT2
JMY
HOMER1
DMGDH
BHMT
Position on chr5 (Mb)
betaine
rs10848574
Response to angiotensin II receptor blocker therapy
Serum metabolite levels
Blood metabolite levels
Height
Obesity (early onset extreme)
Stroke
Monocyte count
Plateletcrit
26 GWAS hits omitted
FAM138D
IQSEC3
SLC6A13
CCDC77
NINJ2
WASH8P
LOC574538
KDM5A
B4GALNT3
LOC100049716
SLC6A12
LOC105369595
LOC101929384
LOC102723544
Position on chr12 (Mb)
betaine
rs6586283
Metabolic syndrome
Non−alcoholic fatty liver disease histology (other)
Information processing speed
Diastolic blood pressure
23 GWAS hits omitted
SLC37A1
LINC01668
NDUFV3
CBS
CRYAA
LINC00322
LINC01669
LINC01671
WDR4
PKNOX1
LOC106780825
LINC01679
HSF2BP
PDE9A
ERVH48−1
CBSL
CRYAA2
SIK1B
MIR5692B
U2AF1
SIK1
U2AF1L5
LINC00319
FRGCA
LINC00313
Position on chr21 (Mb)
androsterone sulfate
rs112359954
Nicotine glucuronidation
Cotinine glucuronidation
Cotinine glucuronidation
Hemoglobin
Hematocrit
17 GWAS hits omitted
TMPRSS11F
YTHDC1
TMPRSS11E
UGT2B15
UGT2B10
UGT2B7
FTLP10
UGT2B17
UGT2A3
TMPRSS11BNL
TMPRSS11B
Position on chr4 (Mb)
androsterone sulfate
rs45446698
Resting hear
Resting he
Iron status biomarkers
139 GWAS hits omitted
34 genes omitted
Position on chr7 (Mb)

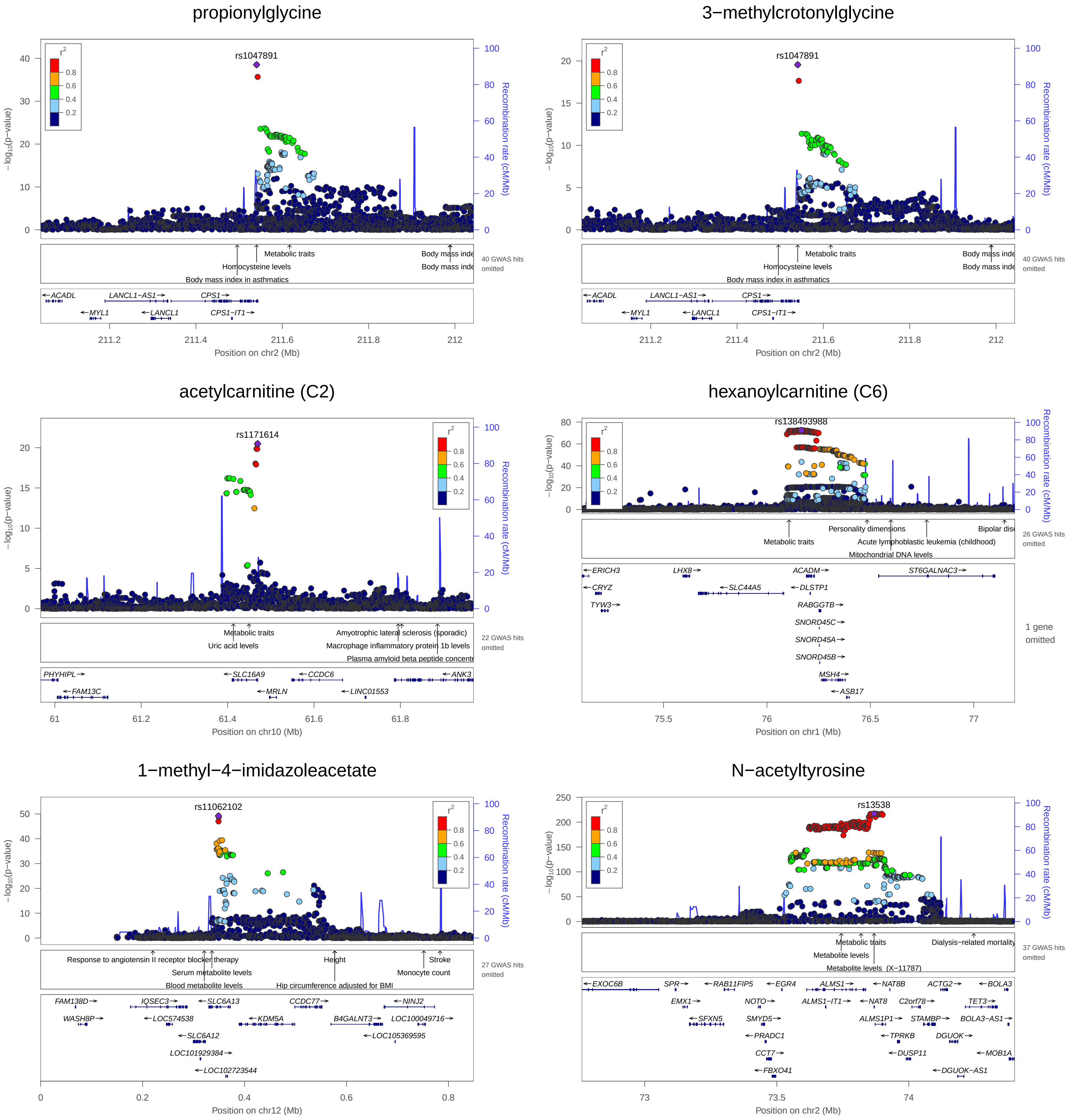

propionylglycine
rs1047891
−log10(p−value)
Recombination rate (cM/Mb)
Metabolic traits
Homocysteine levels
Body mass index in asthmatics
40 GWAS hits omitted
ACADL
LANCL1−AS1
CPS1
MYL1
LANCL1
CPS1−IT1
Position on chr2 (Mb)
3−methylcrotonylglycine
rs1047891
acetylcarnitine (C2)
rs1171614
Metabolic traits
Uric acid levels
Amyotrophic lateral sclerosis (sporadic)
Macrophage inflammatory protein 1b levels
Plasma amyloid beta peptide concentr
22 GWAS hits omitted
PHYHIPL
FAM13C
SLC16A9
MRLN
CCDC6
LINC01553
ANK3
Position on chr10 (Mb)
hexanoylcarnitine (C6)
rs138493988
Personality dimensions
Metabolic traits
Acute lymphoblastic leukemia (childhood)
Mitochondrial DNA levels
Bipolar dis
26 GWAS hits omitted
ERICH3
CRYZ
TYW3
LHX8
SLC44A5
ACADM
DLSTP1
RABGGTB
SNORD45C
SNORD45A
SNORD45B
MSH4
ASB17
ST6GALNAC3
1 gene omitted
Position on chr1 (Mb)
1−methyl−4−imidazoleacetate
rs11062102
Response to angiotensin II receptor blocker therapy
Serum metabolite levels
Blood metabolite levels
Height
Hip circumference adjusted for BMI
Stroke
Monocyte count
27 GWAS hits omitted
FAM138D
WASH8P
IQSEC3
LOC574538
SLC6A12
LOC101929384
LOC102723544
SLC6A13
KDM5A
CCDC77
B4GALNT3
NINJ2
LOC100049716
LOC105369595
Position on chr12 (Mb)
N−acetyltyrosine
rs13538
Metabolic traits
Metabolite levels
Metabolite levels (X−11787)
Dialysis−related mortality
37 GWAS hits omitted
EXOC6B
SPR
EMX1
SFXN5
RAB11FIP5
NOTO
SMYD5
PRADC1
CCT7
FBXO41
EGR4
ALMS1
ALMS1−IT1
NAT8B
NAT8
ALMS1P1
TPRKB
DUSP11
C2orf78
STAMBP
ACTG2
DGUOK
DGUOK−AS1
TET3
BOLA3
BOLA3−AS1
MOB1A
Position on chr2 (Mb)

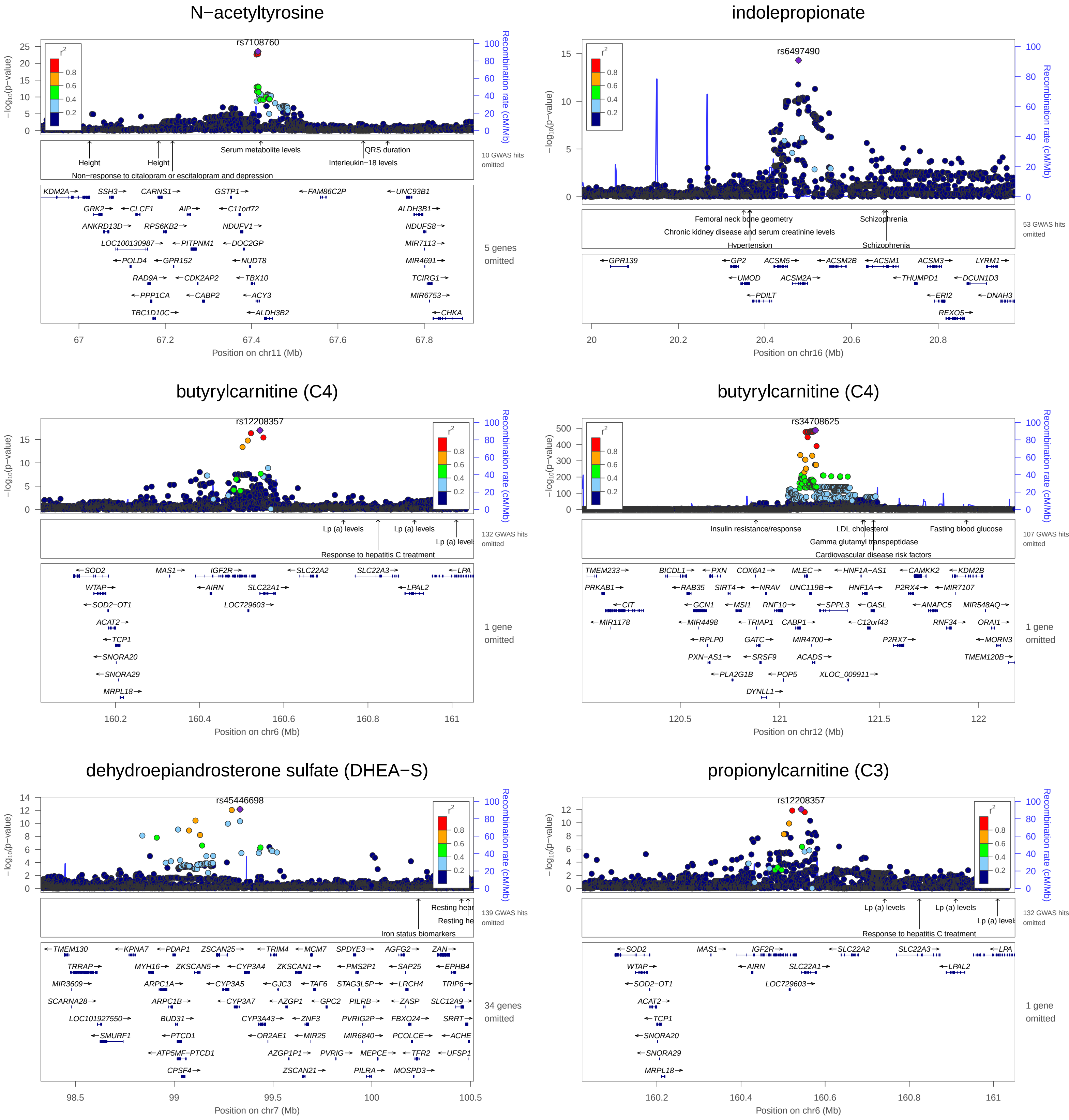
N−acetyltyrosine
rs7108760
−log10(p−value)
Recombination rate (cM/Mb)
Height
Height
Serum metabolite levels
QRS duration
Interleukin−18 levels
Non−response to citalopram or escitalopram and depression
10 GWAS hits omitted
KDM2A SSH3 CARNS1 GSTP1 FAM86C2P UNC93B1
GRK2 CLCF1 AIP C11orf72 ALDH3B1
ANKRD13D RPS6KB2 NDUFV1 NDUFS8
LOC100130987 PITPNM1 DOC2GP MIR7113
POLD4 GPR152 NUDT8 MIR4691
RAD9A CDK2AP2 TBX10 TCIRG1
PPP1CA CABP2 ACY3 MIR6753
TBC1D10C ALDH3B2 CHKA
5 genes omitted
Position on chr11 (Mb)
indolepropionate
rs6497490
Femoral neck bone geometry
Schizophrenia
Chronic kidney disease and serum creatinine levels
Hypertension
Schizophrenia
53 GWAS hits omitted
GPR139 GP2 ACSM5 ACSM2B ACSM1 ACSM3 LYRM1
UMOD ACSM2A THUMPD1 DCUN1D3
PDILT ERI2 DNAH3
REXO5
Position on chr16 (Mb)
butyrylcarnitine (C4)
rs12208357
Lp (a) levels
Lp (a) levels
Lp (a) levels
Response to hepatitis C treatment
132 GWAS hits omitted
SOD2 MAS1 IGF2R SLC22A2 SLC22A3 LPA
WTAP AIRN SLC22A1 LPAL2
SOD2−OT1 LOC729603
ACAT2
TCP1
SNORA20
SNORA29
MRPL18
1 gene omitted
Position on chr6 (Mb)
butyrylcarnitine (C4)
rs34708625
Insulin resistance/response
LDL cholesterol
Fasting blood glucose
Gamma glutamyl transpeptidase
Cardiovascular disease risk factors
107 GWAS hits omitted
TMEM233 BICDL1 PXN COX6A1 MLEC HNF1A−AS1 CAMKK2 KDM2B
PRKAB1 RAB35 SIRT4 NRAV UNC119B HNF1A P2RX4 MIR7107
CIT GCN1 MSI1 RNF10 SPPL3 OASL ANAPC5 MIR548AQ
MIR1178 MIR4498 TRIAP1 CABP1 C12orf43 RNF34 ORAI1
RPLP0 GATC MIR4700 P2RX7 MORN3
PXN−AS1 SRSF9 ACADS TMEM120B
PLA2G1B POP5 XLOC_009911
DYNLL1
1 gene omitted
Position on chr12 (Mb)
dehydroepiandrosterone sulfate (DHEA−S)
rs45446698
Resting hear
Resting he
Iron status biomarkers
139 GWAS hits omitted
TMEM130 KPNA7 PDAP1 ZSCAN25 TRIM4 MCM7 SPDYE3 AGFG2 ZAN
TRRAP MYH16 ZKSCAN5 CYP3A4 ZKSCAN1 PMS2P1 SAP25 EPHB4
MIR3609 ARPC1A CYP3A5 GJC3 TAF6 STAG3L5P LRCH4 TRIP6
SCARNA28 ARPC1B CYP3A7 AZGP1 GPC2 PILRB ZASP SLC12A9
LOC101927550 BUD31 CYP3A43 ZNF3 PVRIG2P FBXO24 SRRT
SMURF1 PTCD1 OR2AE1 MIR25 MIR6840 PCOLCE ACHE
ATP5MF−PTCD1 AZGP1P1 PVRIG MEPCE TFR2 UFSP1
CPSF4 ZSCAN21 PILRA MOSPD3
34 genes omitted
Position on chr7 (Mb)
propionylcarnitine (C3)
rs12208357
Lp (a) levels
Lp (a) levels
Lp (a) level
Response to hepatitis C treatment
132 GWAS hits omitted
SOD2 MAS1 IGF2R SLC22A2 SLC22A3 LPA
WTAP AIRN SLC22A1 LPAL2
SOD2−OT1 LOC729603
ACAT2
TCP1
SNORA20
SNORA29
MRPL18
1 gene omitted
Position on chr6 (Mb)

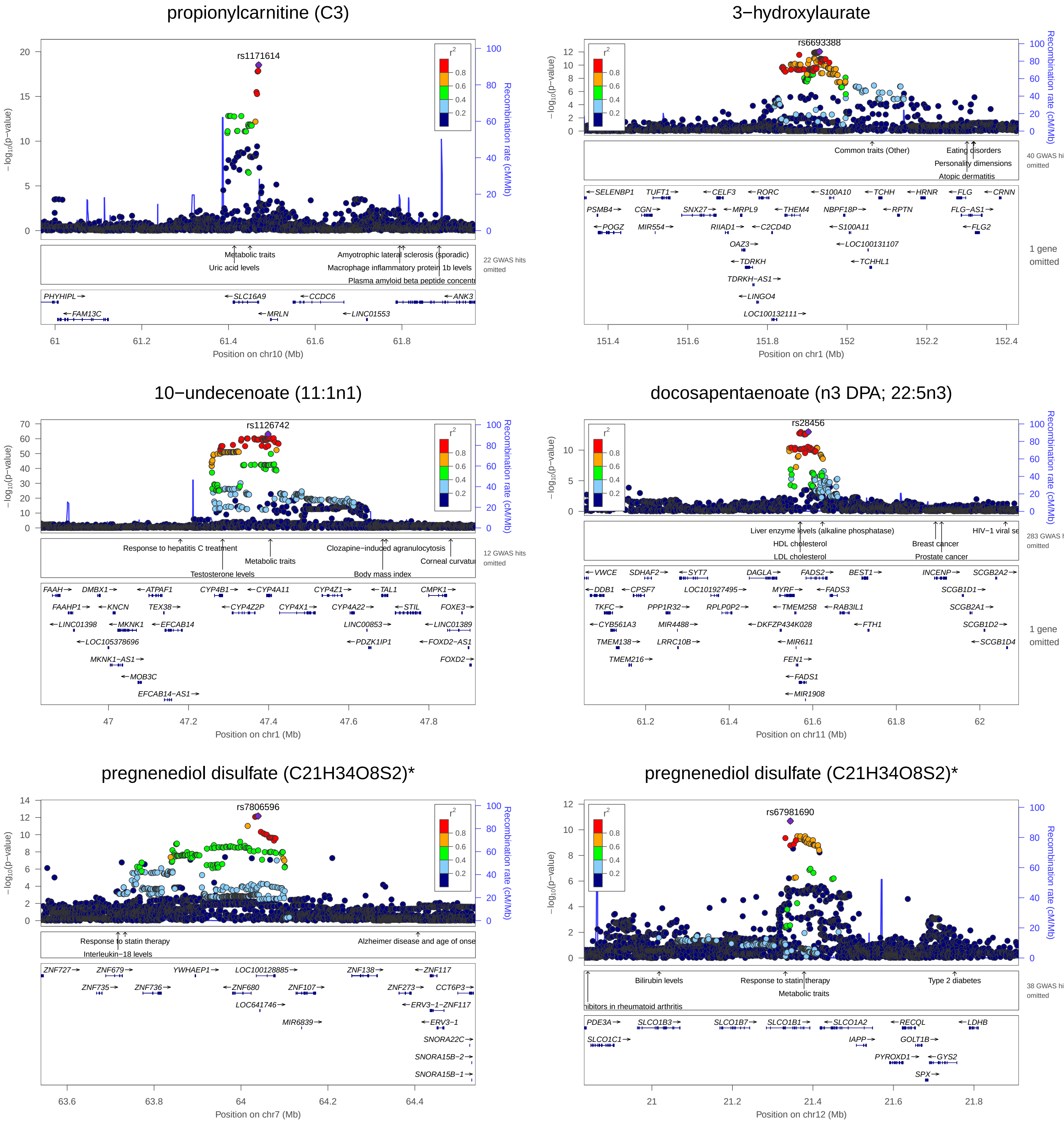
propionylcarnitine (C3)
rs1171614
−log10(p−value)
Recombination rate (cM/Mb)
Metabolic traits
Uric acid levels
Amyotrophic lateral sclerosis (sporadic)
Macrophage inflammatory protein 1b levels
Plasma amyloid beta peptide concentr
22 GWAS hits omitted
PHYHIPL
FAM13C
SLC16A9
MRLN
CCDC6
LINC01553
ANK3
Position on chr10 (Mb)
3−hydroxylaurate
rs6693388
Common traits (Other)
Eating disorders
Personality dimensions
Atopic dermatitis
40 GWAS hits omitted
SELENBP1
TUFT1
CELF3
RORC
S100A10
TCHH
HRNR
FLG
CRNN
PSMB4
CGN
SNX27
MRPL9
THEM4
NBPF18P
RPTN
FLG−AS1
POGZ
MIR554
RIIAD1
C2CD4D
S100A11
FLG2
OAZ3
LOC100131107
TDRKH
TCHHL1
TDRKH−AS1
LINGO4
LOC100132111
1 gene omitted
Position on chr1 (Mb)
10−undecenoate (11:1n1)
rs1126742
Response to hepatitis C treatment
Metabolic traits
Testosterone levels
Clozapine−induced agranulocytosis
Corneal curvatu
Body mass index
12 GWAS hits omitted
FAAH
DMBX1
ATPAF1
CYP4B1
CYP4A11
CYP4Z1
TAL1
CMPK1
FAAHP1
KNCN
TEX38
CYP4Z2P
CYP4X1
CYP4A22
STIL
FOXE3
LINC01398
MKNK1
EFCAB14
LINC00853
LINC01389
LOC105378696
PDZK1IP1
FOXD2−AS1
MKNK1−AS1
FOXD2
MOB3C
EFCAB14−AS1
Position on chr1 (Mb)
docosapentaenoate (n3 DPA; 22:5n3)
rs28456
Liver enzyme levels (alkaline phosphatase)
HDL cholesterol
LDL cholesterol
Breast cancer
Prostate cancer
HIV−1 viral se
283 GWAS hits omitted
VWCE
SDHAF2
SYT7
DAGLA
FADS2
BEST1
INCENP
SCGB2A2
DDB1
CPSF7
LOC101927495
MYRF
FADS3
SCGB1D1
TKFC
PPP1R32
RPLP0P2
TMEM258
RAB3IL1
SCGB2A1
CYB561A3
MIR4488
DKFZP434K028
FTH1
SCGB1D2
TMEM138
LRRC10B
MIR611
SCGB1D4
TMEM216
FEN1
FADS1
MIR1908
1 gene omitted
Position on chr11 (Mb)
pregnenediol disulfate (C21H34O8S2)*
rs7806596
Response to statin therapy
Interleukin−18 levels
Alzheimer disease and age of onse
ZNF727
ZNF679
YWHAEP1
LOC100128885
ZNF138
ZNF117
ZNF735
ZNF736
ZNF680
ZNF107
ZNF273
CCT6P3
LOC641746
ERV3−1−ZNF117
MIR6839
ERV3−1
SNORA22C
SNORA15B−2
SNORA15B−1
Position on chr7 (Mb)
pregnenediol disulfate (C21H34O8S2)*
rs67981690
Bilirubin levels
Response to statin therapy
Metabolic traits
Type 2 diabetes
nibitors in rheumatoid arthritis
38 GWAS hits omitted
PDE3A
SLCO1B3
SLCO1B7
SLCO1B1
SLCO1A2
RECQL
LDHB
SLCO1C1
IAPP
GOLT1B
PYROXD1
GYS2
SPX
Position on chr12 (Mb)

pregnenediol disulfate (C21H34O8S2)*

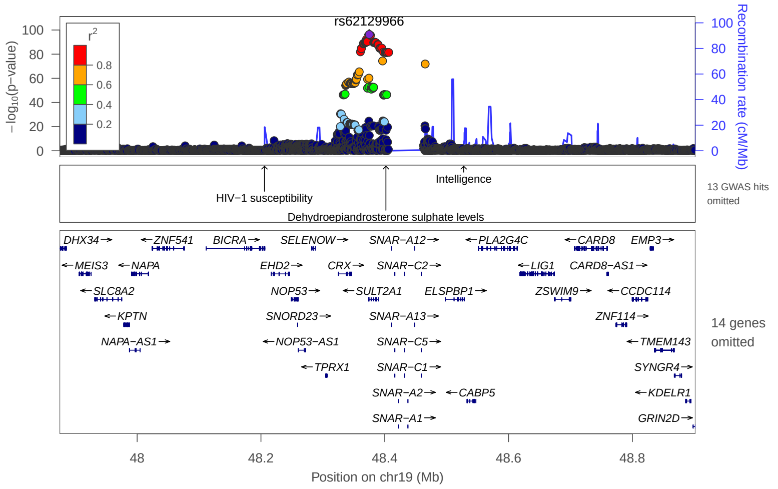

bilirubin (E,E)*

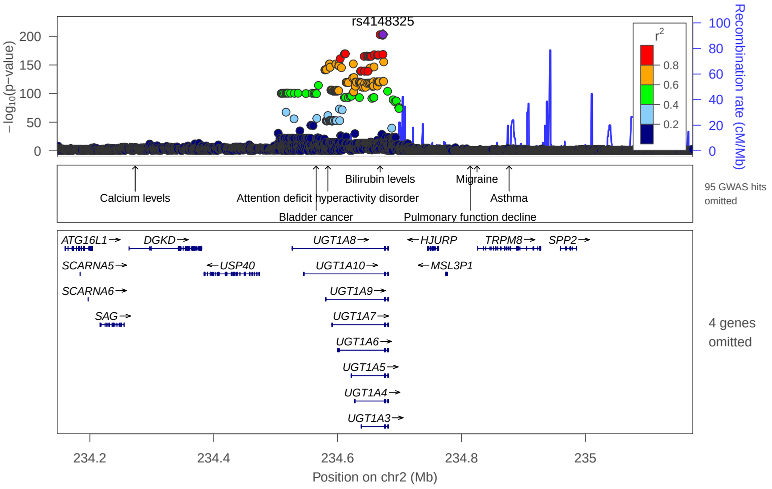

glycolithocholate sulfate*

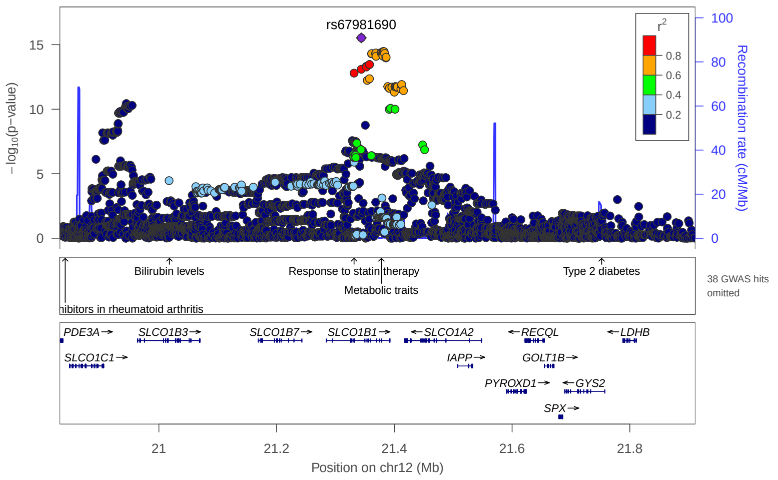

taurocholenate sulfate*

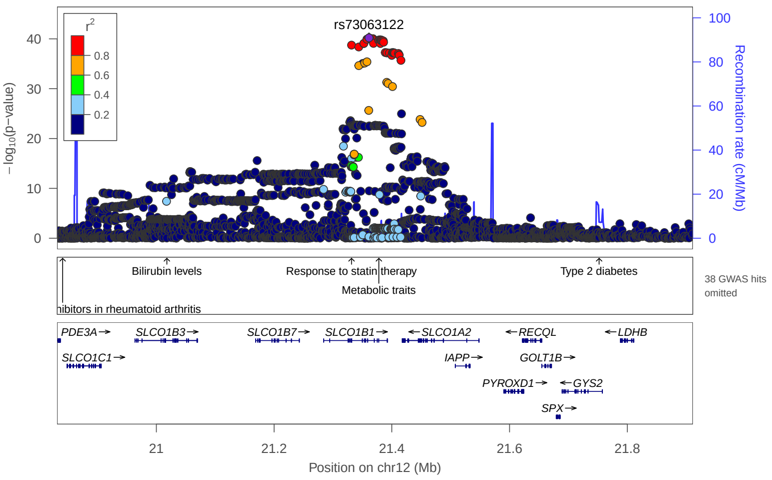

andro steroid monosulfate C19H28O6S (1)*

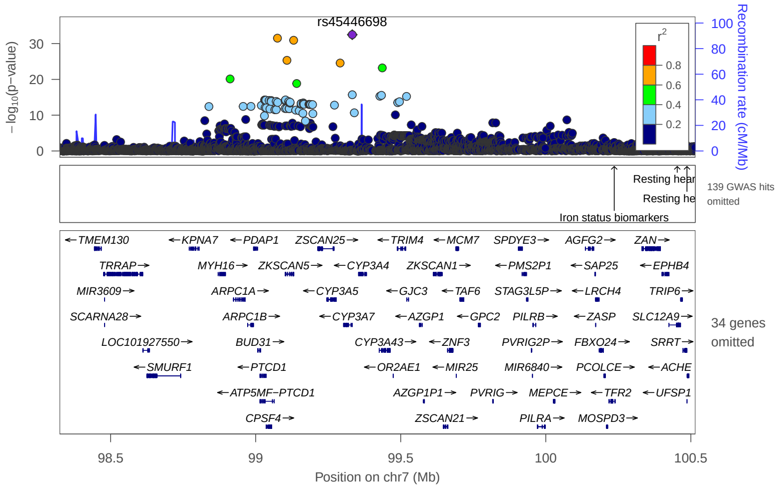

adrenate (22:4n6)

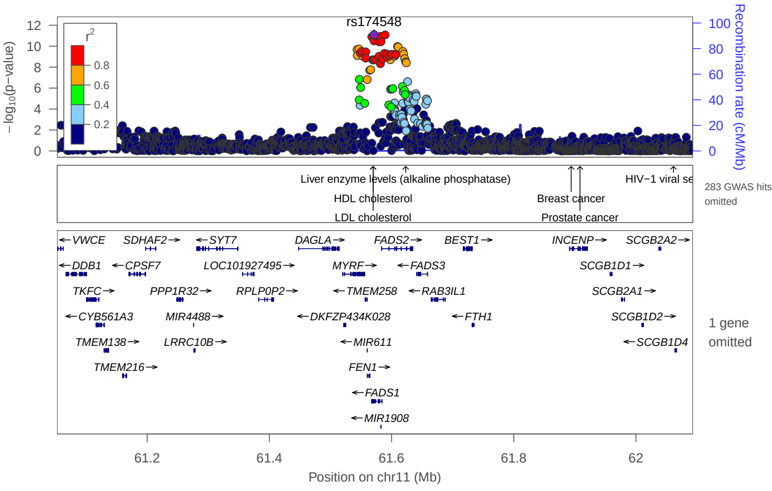

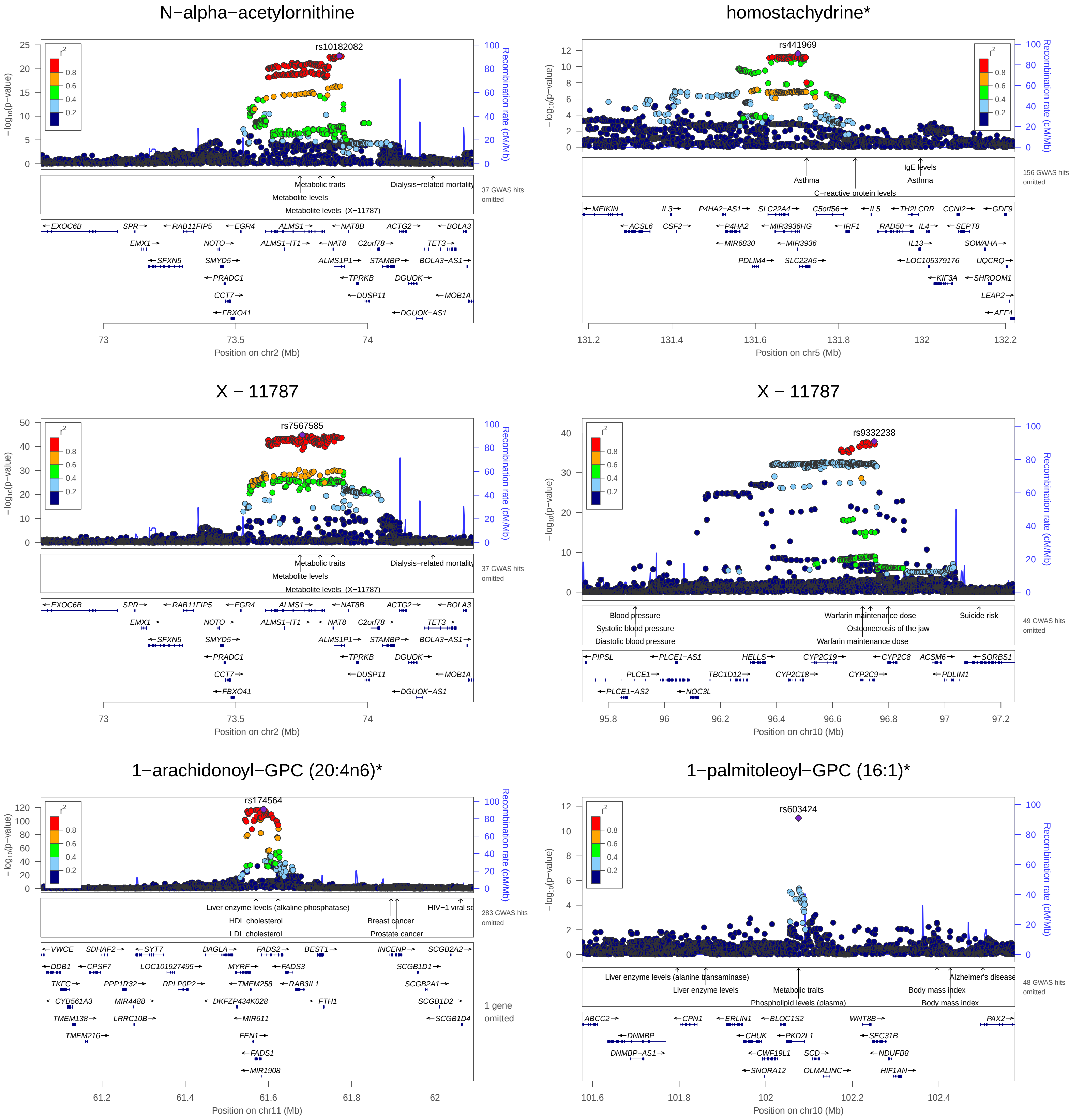

N−alpha−acetylornithine
rs10182082
−log10(p−value)
Recombination rate (cM/Mb)
Metabolic traits
Metabolite levels
Metabolite levels (X−11787)
Dialysis−related mortality
37 GWAS hits omitted
EXOC6B
SPR
RAB11FIP5
EGR4
ALMS1
NAT8B
ACTG2
BOLA3
EMX1
NOTO
ALMS1−IT1
NAT8
C2orf78
TET3
SFXN5
SMYD5
ALMS1P1
STAMBP
BOLA3−AS1
PRADC1
TPRKB
DGUOK
CCT7
DUSP11
MOB1A
FBXO41
DGUOK−AS1
Position on chr2 (Mb)
homostachydrine*
rs441969
Asthma
C−reactive protein levels
IgE levels
156 GWAS hits omitted
MEIKIN
IL3
P4HA2−AS1
SLC22A4
C5orf56
IL5
TH2LCRR
CCNI2
GDF9
ACSL6
CSF2
P4HA2
MIR3936HG
IRF1
RAD50
IL4
SEPT8
MIR6830
MIR3936
IL13
SOWAHA
PDLIM4
SLC22A5
LOC105379176
UQCRQ
KIF3A
SHROOM1
LEAP2
AFF4
Position on chr5 (Mb)
X − 11787
rs7567585
Position on chr2 (Mb)
X − 11787
rs9332238
Blood pressure
Systolic blood pressure
Diastolic blood pressure
Warfarin maintenance dose
Osteonecrosis of the jaw
Suicide risk
49 GWAS hits omitted
PIPSL
PLCE1−AS1
HELLS
CYP2C19
CYP2C8
ACSM6
SORBS1
PLCE1
TBC1D12
CYP2C18
CYP2C9
PDLIM1
PLCE1−AS2
NOC3L
Position on chr10 (Mb)
1−arachidonoyl−GPC (20:4n6)*
rs174564
Liver enzyme levels (alkaline phosphatase)
HDL cholesterol
LDL cholesterol
Breast cancer
Prostate cancer
HIV−1 viral se
283 GWAS hits omitted
VWCE
SDHAF2
SYT7
DAGLA
FADS2
BEST1
INCENP
SCGB2A2
DDB1
CPSF7
LOC101927495
MYRF
FADS3
SCGB1D1
TKFC
PPP1R32
RPLP0P2
TMEM258
RAB3IL1
SCGB2A1
CYB561A3
MIR4488
DKFZP434K028
FTH1
SCGB1D2
TMEM138
LRRC10B
MIR611
SCGB1D4
TMEM216
FEN1
FADS1
MIR1908
1 gene omitted
Position on chr11 (Mb)
1−palmitoleoyl−GPC (16:1)*
rs603424
Liver enzyme levels (alanine transaminase)
Liver enzyme levels
Metabolic traits
Phospholipid levels (plasma)
Body mass index
Alzheimer's disease
48 GWAS hits omitted
ABCC2
CPN1
ERLIN1
BLOC1S2
WNT8B
PAX2
DNMBP
CHUK
PKD2L1
SEC31B
DNMBP−AS1
CWF19L1
SCD
NDUFB8
SNORA12
OLMALINC
HIF1AN
Position on chr10 (Mb)

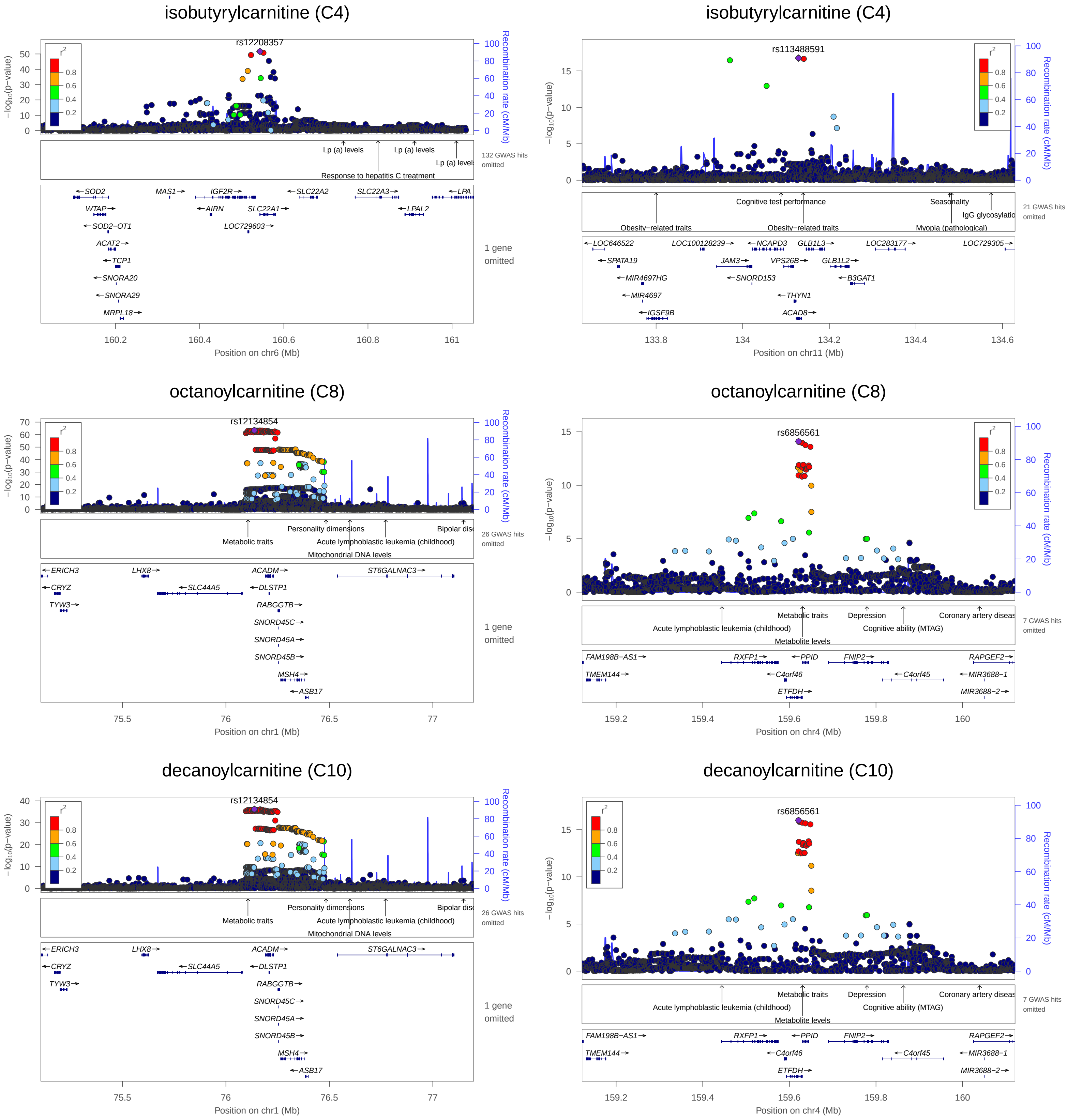

isobutyrylcarnitine (C4)
rs12208357
Lp (a) levels
Response to hepatitis C treatment
132 GWAS hits omitted
SOD2
WTAP
SOD2−OT1
ACAT2
TCP1
SNORA20
SNORA29
MRPL18
MAS1
IGF2R
AIRN
SLC22A1
LOC729603
SLC22A2
SLC22A3
LPAL2
LPA
1 gene omitted
Position on chr6 (Mb)
isobutyrylcarnitine (C4)
rs113488591
Cognitive test performance
Seasonality
IgG glycosylation
Obesity−related traits
Myopia (pathological)
21 GWAS hits omitted
LOC646522
SPATA19
MIR4697HG
MIR4697
IGSF9B
LOC100128239
NCAPD3
JAM3
SNORD153
VPS26B
THYN1
ACAD8
GLB1L3
GLB1L2
B3GAT1
LOC283177
LOC729305
Position on chr11 (Mb)
octanoylcarnitine (C8)
rs12134854
Personality dimensions
Metabolic traits
Acute lymphoblastic leukemia (childhood)
Mitochondrial DNA levels
26 GWAS hits omitted
ERICH3
CRYZ
TYW3
LHX8
SLC44A5
ACADM
DLSTP1
RABGGTB
SNORD45C
SNORD45A
SNORD45B
MSH4
ASB17
ST6GALNAC3
1 gene omitted
Position on chr1 (Mb)
octanoylcarnitine (C8)
rs6856561
Metabolic traits
Depression
Acute lymphoblastic leukemia (childhood)
Cognitive ability (MTAG)
Metabolite levels
7 GWAS hits omitted
FAM198B−AS1
TMEM144
RXFP1
C4orf46
ETFDH
PPID
FNIP2
C4orf45
RAPGEF2
MIR3688−1
MIR3688−2
Position on chr4 (Mb)
decanoylcarnitine (C10)
rs12134854
Position on chr1 (Mb)
decanoylcarnitine (C10)
rs6856561
Position on chr4 (Mb)
−log10(p−value)
Recombination rate (cM/Mb)

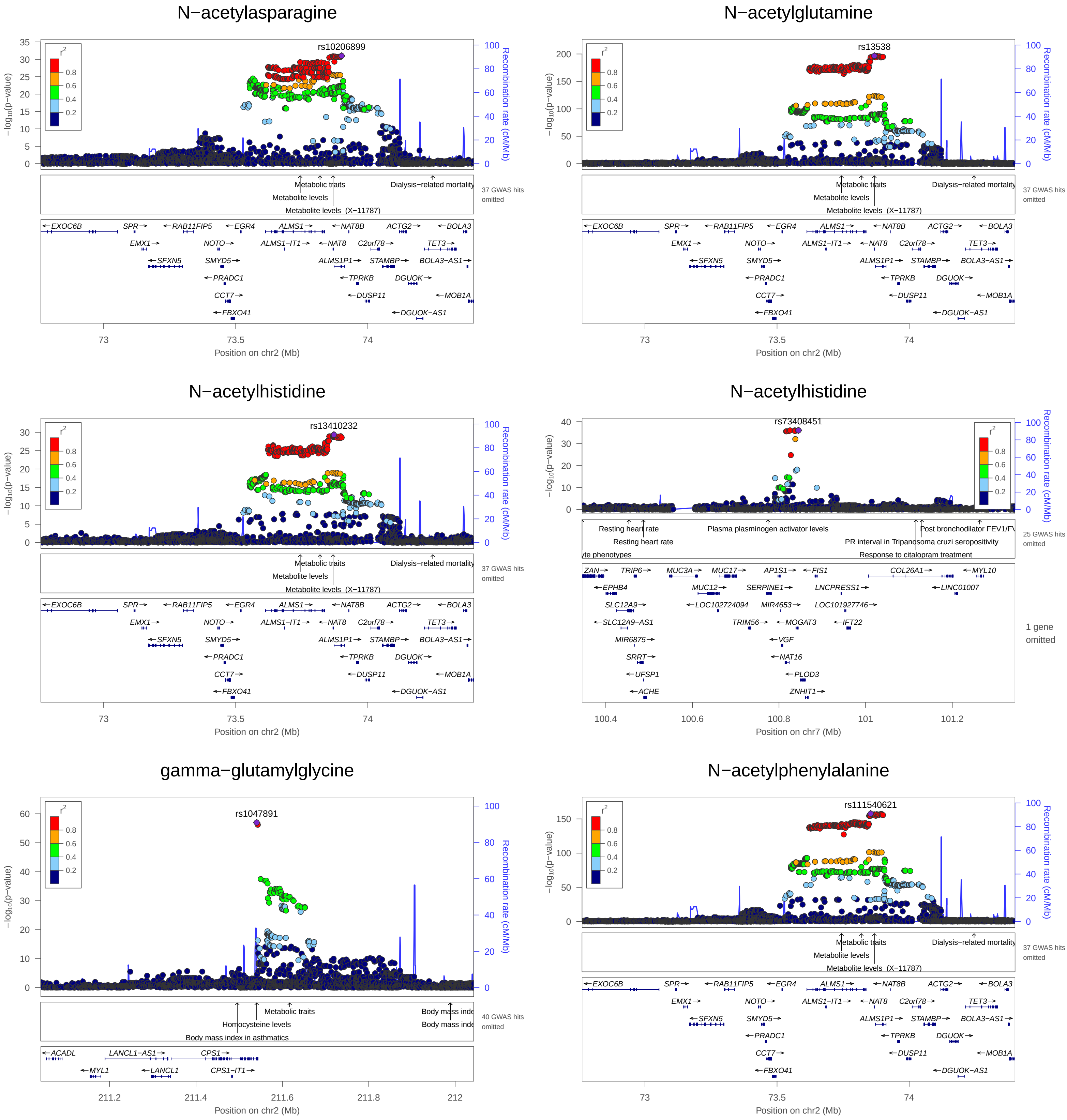
N−acetylasparagine
rs10206899
N−acetylglutamine
rs13538
N−acetylhistidine
rs13410232
N−acetylhistidine
rs73408451
gamma−glutamylglycine
rs1047891
N−acetylphenylalanine
rs111540621
−log10(p−value)
Recombination rate (cM/Mb)
r2
0.8
0.6
0.4
0.2
Metabolic traits
Metabolite levels
Metabolite levels (X−11787)
Dialysis−related mortality
37 GWAS hits omitted
25 GWAS hits omitted
40 GWAS hits omitted
1 gene omitted
Resting heart rate
Plasma plasminogen activator levels
Post bronchodilator FEV1/FV
PR interval in Tripanosoma cruzi seropositivity
Response to citalopram treatment
Homocysteine levels
Body mass index in asthmatics
EXOC6B
SPR
RAB11FIP5
EGR4
ALMS1
NAT8B
ACTG2
BOLA3
EMX1
NOTO
ALMS1−IT1
NAT8
C2orf78
TET3
SFXN5
SMYD5
ALMS1P1
STAMBP
BOLA3−AS1
PRADC1
TPRKB
DGUOK
CCT7
DUSP11
MOB1A
FBXO41
DGUOK−AS1
ZAN
TRIP6
MUC3A
MUC17
AP1S1
FIS1
COL26A1
MYL10
EPHB4
MUC12
SERPINE1
LNCPRESS1
LINC01007
SLC12A9
LOC102724094
MIR4653
LOC101927746
SLC12A9−AS1
TRIM56
MOGAT3
IFT22
MIR6875
VGF
SRRT
NAT16
UFSP1
PLOD3
ACHE
ZNHIT1
ACADL
LANCL1−AS1
CPS1
MYL1
LANCL1
CPS1−IT1
Position on chr2 (Mb)
Position on chr7 (Mb)

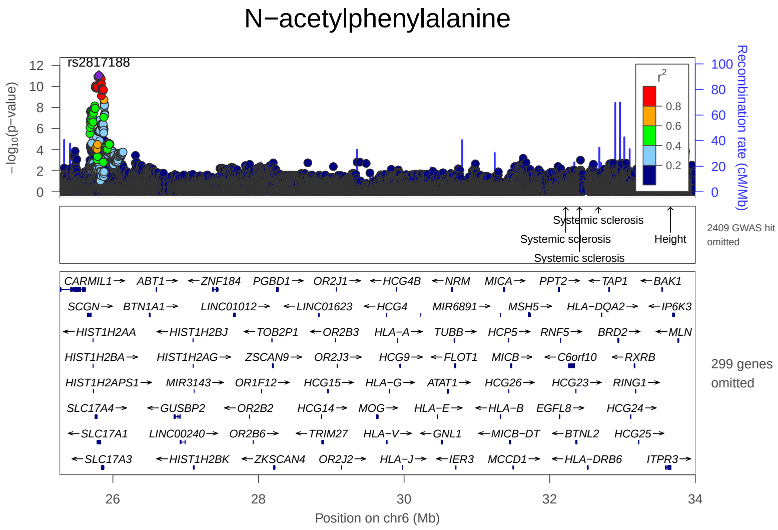
N−acetylphenylalanine
rs2817188
−log10(p−value)
Recombination rate (cM/Mb)
r2
Systemic sclerosis
Systemic sclerosis
Height
Systemic sclerosis
2409 GWAS hit omitted
CARMIL1 ABT1 ZNF184 PGBD1 OR2J1 HCG4B NRM MICA PPT2 TAP1 BAK1
SCGN BTN1A1 LINC01012 LINC01623 HCG4 MIR6891 MSH5 HLA−DQA2 IP6K3
HIST1H2AA HIST1H2BJ TOB2P1 OR2B3 HLA−A TUBB HCP5 RNF5 BRD2 MLN
HIST1H2BA HIST1H2AG ZSCAN9 OR2J3 HCG9 FLOT1 MICB C6orf10 RXRB
HIST1H2APS1 MIR3143 OR1F12 HCG15 HLA−G ATAT1 HCG26 HCG23 RING1
SLC17A4 GUSBP2 OR2B2 HCG14 MOG HLA−E HLA−B EGFL8 HCG24
SLC17A1 LINC00240 OR2B6 TRIM27 HLA−V GNL1 MICB−DT BTNL2 HCG25
SLC17A3 HIST1H2BK ZKSCAN4 OR2J2 HLA−J IER3 MCCD1 HLA−DRB6 ITPR3
299 genes omitted
Position on chr6 (Mb)

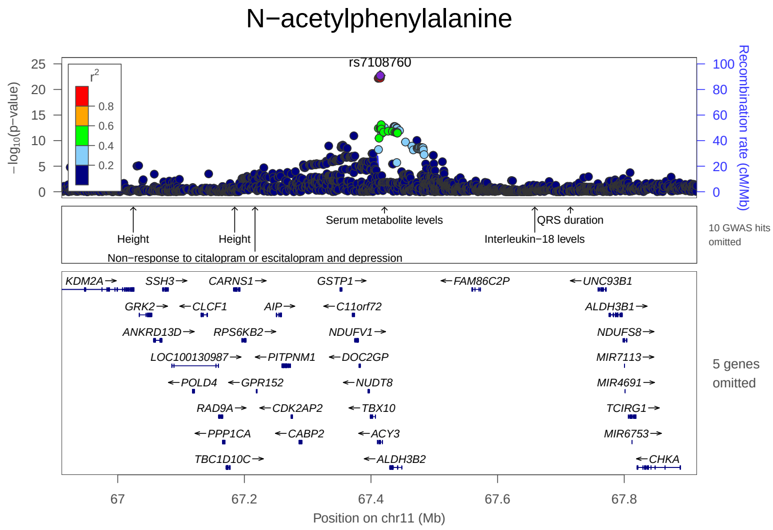
N−acetylphenylalanine
rs7108760
−log10(p−value)
Recombination rate (cM/Mb)
r2
Serum metabolite levels
QRS duration
Height
Height
Interleukin−18 levels
Non−response to citalopram or escitalopram and depression
10 GWAS hits omitted
KDM2A SSH3 CARNS1 GSTP1 FAM86C2P UNC93B1
GRK2 CLCF1 AIP C11orf72 ALDH3B1
ANKRD13D RPS6KB2 NDUFV1 NDUFS8
LOC100130987 PITPNM1 DOC2GP MIR7113
POLD4 GPR152 NUDT8 MIR4691
RAD9A CDK2AP2 TBX10 TCIRG1
PPP1CA CABP2 ACY3 MIR6753
TBC1D10C ALDH3B2 CHKA
5 genes omitted
Position on chr11 (Mb)

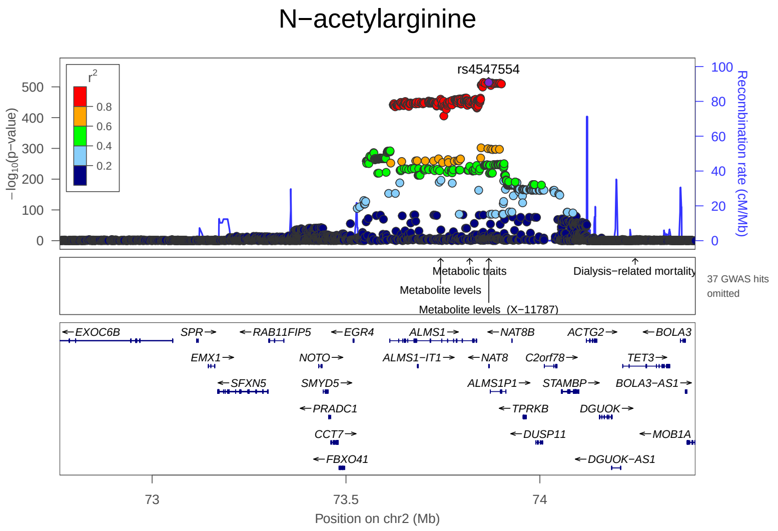
N−acetylarginine
rs4547554
−log10(p−value)
Recombination rate (cM/Mb)
r2
Metabolic traits
Dialysis−related mortality
Metabolite levels
Metabolite levels (X−11787)
37 GWAS hits omitted
EXOC6B SPR RAB11FIP5 EGR4 ALMS1 NAT8B ACTG2 BOLA3
EMX1 NOTO ALMS1−IT1 NAT8 C2orf78 TET3
SFXN5 SMYD5 ALMS1P1 STAMBP BOLA3−AS1
PRADC1 TPRKB DGUOK
CCT7 DUSP11 MOB1A
FBXO41 DGUOK−AS1
Position on chr2 (Mb)

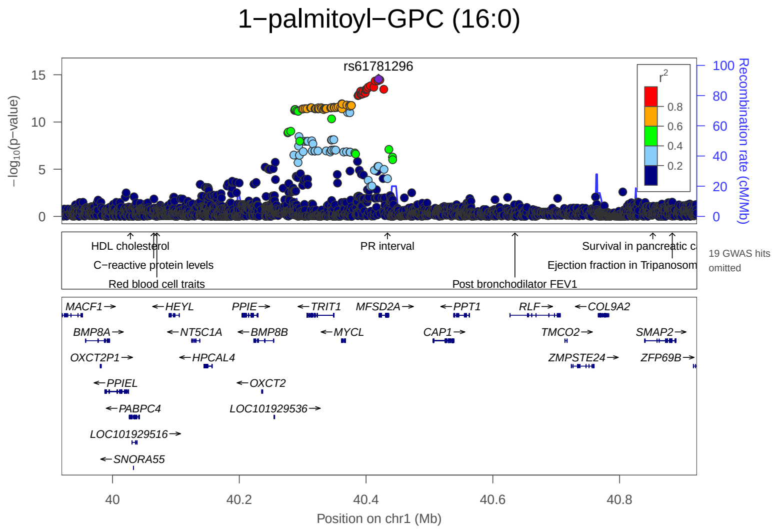
1−palmitoyl−GPC (16:0)
rs61781296
−log10(p−value)
Recombination rate (cM/Mb)
r2
HDL cholesterol
PR interval
Survival in pancreatic c
C−reactive protein levels
Ejection fraction in Tripanosom
Red blood cell traits
Post bronchodilator FEV1
19 GWAS hits omitted
MACF1 HEYL PPIE TRIT1 MFSD2A PPT1 RLF COL9A2
BMP8A NT5C1A BMP8B MYCL CAP1 TMCO2 SMAP2
OXCT2P1 HPCAL4 ZMPSTE24 ZFP69B
PPIEL OXCT2
PABPC4 LOC101929536
LOC101929516
SNORA55
Position on chr1 (Mb)

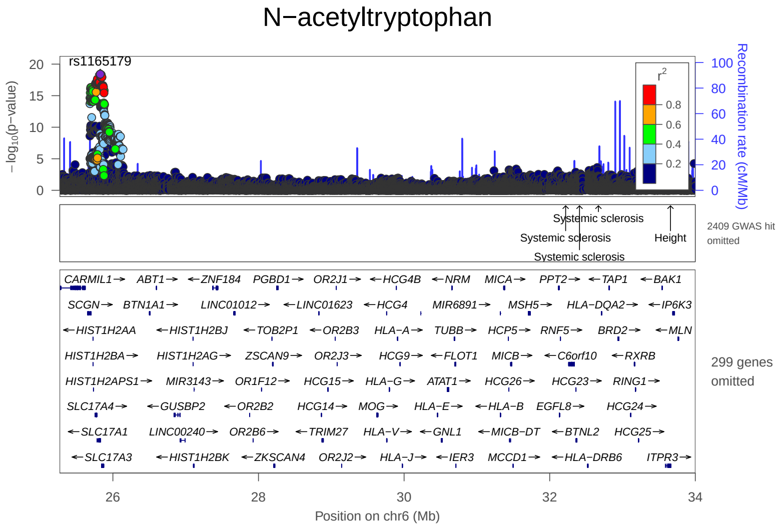
N−acetyltryptophan
rs1165179
−log10(p−value)
Recombination rate (cM/Mb)
r2
Systemic sclerosis
Systemic sclerosis
Height
Systemic sclerosis
2409 GWAS hit omitted
CARMIL1 ABT1 ZNF184 PGBD1 OR2J1 HCG4B NRM MICA PPT2 TAP1 BAK1
SCGN BTN1A1 LINC01012 LINC01623 HCG4 MIR6891 MSH5 HLA−DQA2 IP6K3
HIST1H2AA HIST1H2BJ TOB2P1 OR2B3 HLA−A TUBB HCP5 RNF5 BRD2 MLN
HIST1H2BA HIST1H2AG ZSCAN9 OR2J3 HCG9 FLOT1 MICB C6orf10 RXRB
HIST1H2APS1 MIR3143 OR1F12 HCG15 HLA−G ATAT1 HCG26 HCG23 RING1
SLC17A4 GUSBP2 OR2B2 HCG14 MOG HLA−E HLA−B EGFL8 HCG24
SLC17A1 LINC00240 OR2B6 TRIM27 HLA−V GNL1 MICB−DT BTNL2 HCG25
SLC17A3 HIST1H2BK ZKSCAN4 OR2J2 HLA−J IER3 MCCD1 HLA−DRB6 ITPR3
299 genes omitted
Position on chr6 (Mb)

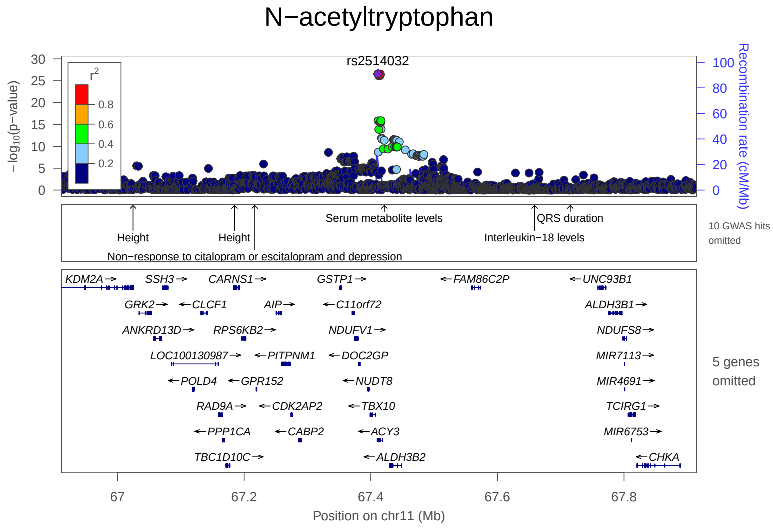
N−acetyltryptophan
rs2514032
−log10(p−value)
Recombination rate (cM/Mb)
r2
Serum metabolite levels
QRS duration
Height
Height
Interleukin−18 levels
Non−response to citalopram or escitalopram and depression
10 GWAS hits omitted
KDM2A SSH3 CARNS1 GSTP1 FAM86C2P UNC93B1
GRK2 CLCF1 AIP C11orf72 ALDH3B1
ANKRD13D RPS6KB2 NDUFV1 NDUFS8
LOC100130987 PITPNM1 DOC2GP MIR7113
POLD4 GPR152 NUDT8 MIR4691
RAD9A CDK2AP2 TBX10 TCIRG1
PPP1CA CABP2 ACY3 MIR6753
TBC1D10C ALDH3B2 CHKA
5 genes omitted
Position on chr11 (Mb)

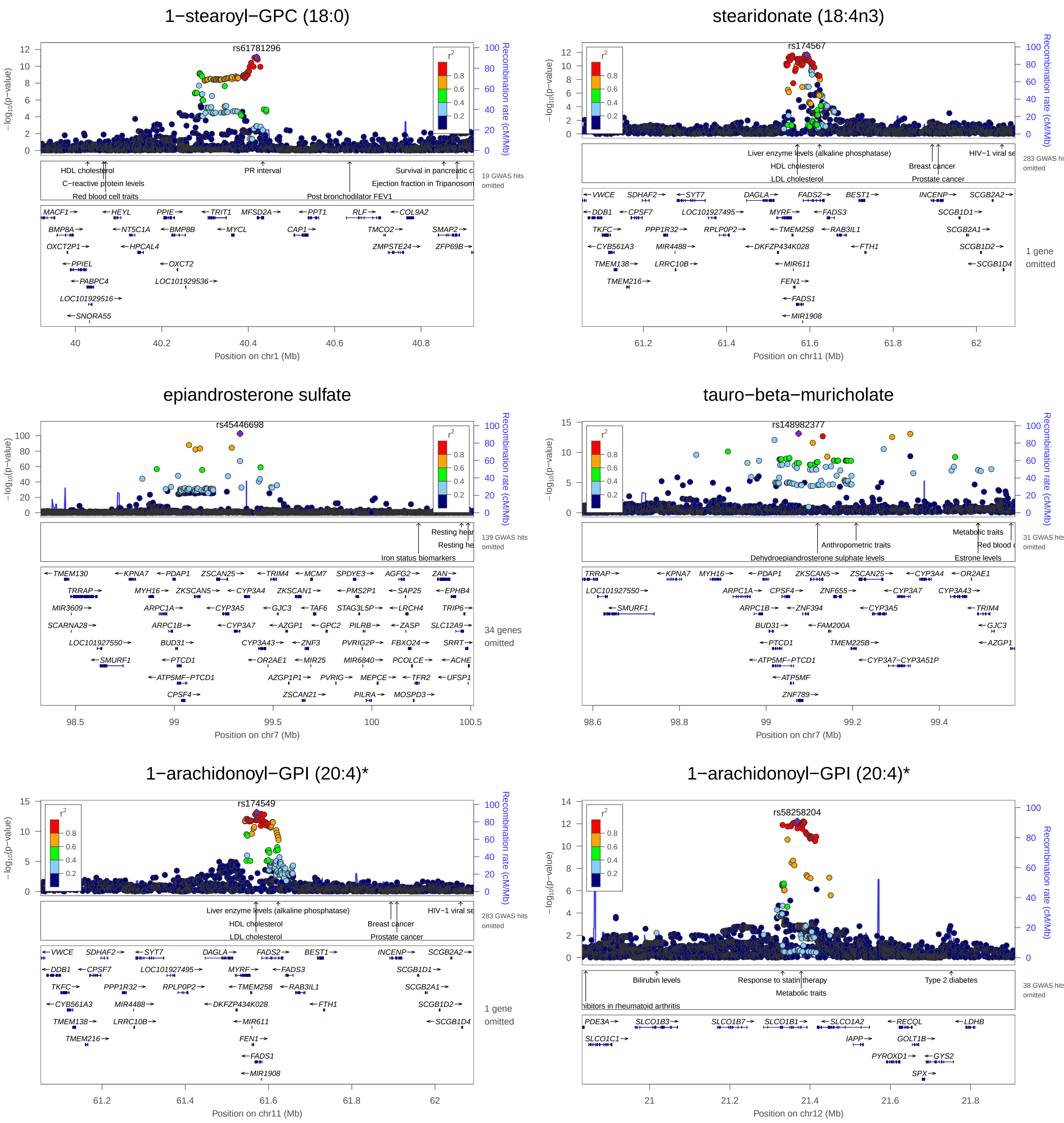
1−stearoyl−GPC (18:0)
rs61781296
−log10(p−value)
Recombination rate (cM/Mb)
HDL cholesterol
C−reactive protein levels
Red blood cell traits
PR interval
Post bronchodilator FEV1
Survival in pancreatic c
Ejection fraction in Tripanosom
19 GWAS hits omitted
MACF1 HEYL NT5C1A HPCAL4 PPIE BMP8B TRIT1 MYCL MFSD2A PPT1 CAP1 RLF COL9A2 TMCO2 ZMPSTE24 SMAP2 ZFP69B BMP8A OXCT2P1 PPIEL PABPC4 LOC101929516 SNORA55 OXCT2 LOC101929536
Position on chr1 (Mb)
stearidonate (18:4n3)
rs174567
Liver enzyme levels (alkaline phosphatase)
HDL cholesterol
LDL cholesterol
Breast cancer
Prostate cancer
HIV−1 viral se
283 GWAS hits omitted
VWCE DDB1 TKFC CYB561A3 TMEM138 TMEM216 SDHAF2 CPSF7 PPP1R32 MIR4488 LRRC10B SYT7 LOC101927495 RPLP0P2 DAGLA MYRF TMEM258 DFKZP434K028 MIR611 FEN1 FADS1 MIR1908 FADS2 FADS3 RAB3IL1 FTH1 BEST1 INCENP SCGB2A2 SCGB1D1 SCGB2A1 SCGB1D2 SCGB1D4
1 gene omitted
Position on chr11 (Mb)
epiandrosterone sulfate
rs45446698
Resting hear
Resting he
Iron status biomarkers
139 GWAS hits omitted
TMEM130 TRRAP MIR3609 SCARNA28 LOC101927550 SMURF1 KPNA7 MYH16 ARPC1A ARPC1B BUD31 PTCD1 ATP5MF−PTCD1 CPSF4 PDAP1 ZKSCAN5 CYP3A5 CYP3A7 CYP3A43 OR2AE1 ZSCAN25 CYP3A4 GJC3 AZGP1 ZNF3 MIR25 AZGP1P1 ZSCAN21 TRIM4 MCM7 ZKSCAN1 TAF6 GPC2 PVRIG PVRIG2P MIR6840 PMS2P1 STAG3L5P PILRB PILRA MEPCE MOSPD3 SPDYE3 AGFG2 SAP25 LRCH4 ZASP FBXO24 PCOLCE TFR2 ZAN EPHB4 TRIP6 SLC12A9 SRRT ACHE UFSP1
34 genes omitted
Position on chr7 (Mb)
tauro−beta−muricholate
rs148982377
Anthropometric traits
Dehydroepiandrosterone sulphate levels
Metabolic traits
Red blood c
Estrone levels
31 GWAS hits omitted
TRRAP LOC101927550 SMURF1 KPNA7 MYH16 ARPC1A ARPC1B BUD31 PTCD1 ATP5MF−PTCD1 ATP5MF ZNF789 PDAP1 CPSF4 ZNF394 FAM200A ZKSCAN5 ZNF655 ZSCAN25 TMEM225B CYP3A4 CYP3A7 CYP3A5 CYP3A7−CYP3A51P OR2AE1 CYP3A43 TRIM4 GJC3 AZGP1
Position on chr7 (Mb)
1−arachidonoyl−GPI (20:4)*
rs174549
Liver enzyme levels (alkaline phosphatase)
HDL cholesterol
LDL cholesterol
Breast cancer
Prostate cancer
HIV−1 viral se
283 GWAS hits omitted
1 gene omitted
Position on chr11 (Mb)
1−arachidonoyl−GPI (20:4)*
rs58258204
Bilirubin levels
Response to statin therapy
Metabolic traits
Type 2 diabetes
hibitors in rheumatoid arthritis
38 GWAS hits omitted
PDE3A SLCO1C1 SLCO1B3 SLCO1B7 SLCO1B1 SLCO1A2 IAPP PYROXD1 RECQL GOLT1B GYS2 SPX LDHB
Position on chr12 (Mb)

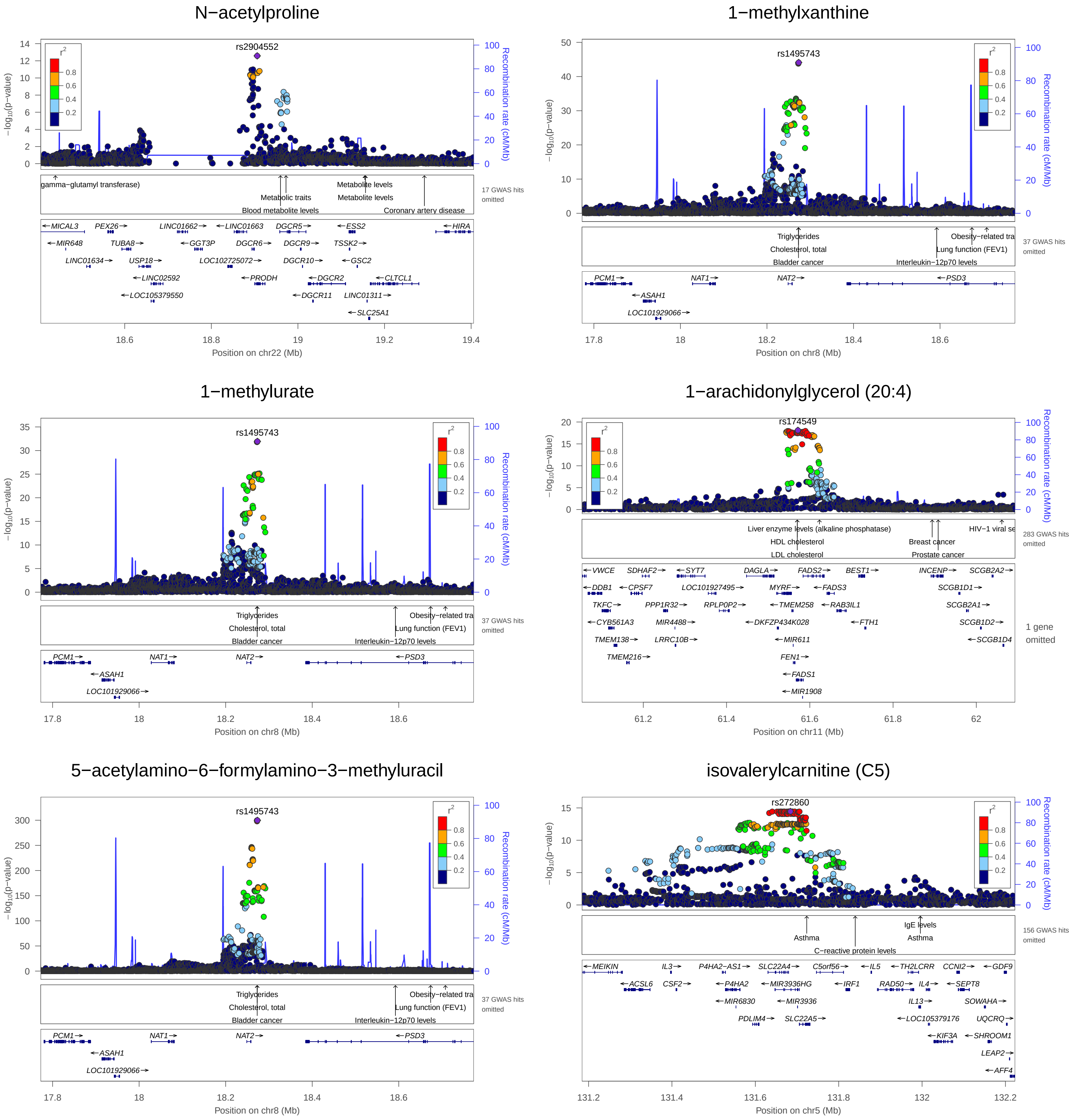
N−acetylproline
rs2904552
−log10(p−value)
Recombination rate (cM/Mb)
r2
gamma−glutamyl transferase)
Metabolic traits
Blood metabolite levels
Metabolite levels
Metabolite levels
Coronary artery disease
17 GWAS hits omitted
MICAL3
PEX26
LINC01662
LINC01663
DGCR5
ESS2
HIRA
MIR648
TUBA8
GGT3P
DGCR6
DGCR9
TSSK2
LINC01634
USP18
LOC102725072
DGCR10
GSC2
LINC02592
PRODH
DGCR2
CLTCL1
LOC105379550
DGCR11
LINC01311
SLC25A1
Position on chr22 (Mb)
1−methylxanthine
rs1495743
Triglycerides
Cholesterol, total
Bladder cancer
Obesity−related tra
Lung function (FEV1)
Interleukin−12p70 levels
37 GWAS hits omitted
PCM1
NAT1
NAT2
PSD3
ASAH1
LOC101929066
Position on chr8 (Mb)
1−methylurate
rs1495743
Triglycerides
Cholesterol, total
Bladder cancer
Obesity−related tra
Lung function (FEV1)
Interleukin−12p70 levels
37 GWAS hits omitted
PCM1
NAT1
NAT2
PSD3
ASAH1
LOC101929066
Position on chr8 (Mb)
1−arachidonylglycerol (20:4)
rs174549
Liver enzyme levels (alkaline phosphatase)
HDL cholesterol
LDL cholesterol
Breast cancer
Prostate cancer
HIV−1 viral se
283 GWAS hits omitted
1 gene omitted
VWCE
SDHAF2
SYT7
DAGLA
FADS2
BEST1
INCENP
SCGB2A2
DDB1
CPSF7
LOC101927495
MYRF
FADS3
SCGB1D1
TKFC
PPP1R32
RPLP0P2
TMEM258
RAB3IL1
SCGB2A1
CYB561A3
MIR4488
DKFZP434K028
FTH1
SCGB1D2
TMEM138
LRRC10B
MIR611
SCGB1D4
TMEM216
FEN1
FADS1
MIR1908
Position on chr11 (Mb)
5−acetylamino−6−formylamino−3−methyluracil
rs1495743
Triglycerides
Cholesterol, total
Bladder cancer
Obesity−related tra
Lung function (FEV1)
Interleukin−12p70 levels
37 GWAS hits omitted
PCM1
NAT1
NAT2
PSD3
ASAH1
LOC101929066
Position on chr8 (Mb)
isovalerylcarnitine (C5)
rs272860
Asthma
C−reactive protein levels
IgE levels
Asthma
156 GWAS hits omitted
MEIKIN
IL3
P4HA2−AS1
SLC22A4
C5orf56
IL5
TH2LCRR
CCNI2
GDF9
ACSL6
CSF2
P4HA2
MIR3936HG
IRF1
RAD50
IL4
SEPT8
MIR6830
MIR3936
IL13
SOWAHA
PDLIM4
SLC22A5
LOC105379176
UQCRQ
KIF3A
SHROOM1
LEAP2
AFF4
Position on chr5 (Mb)

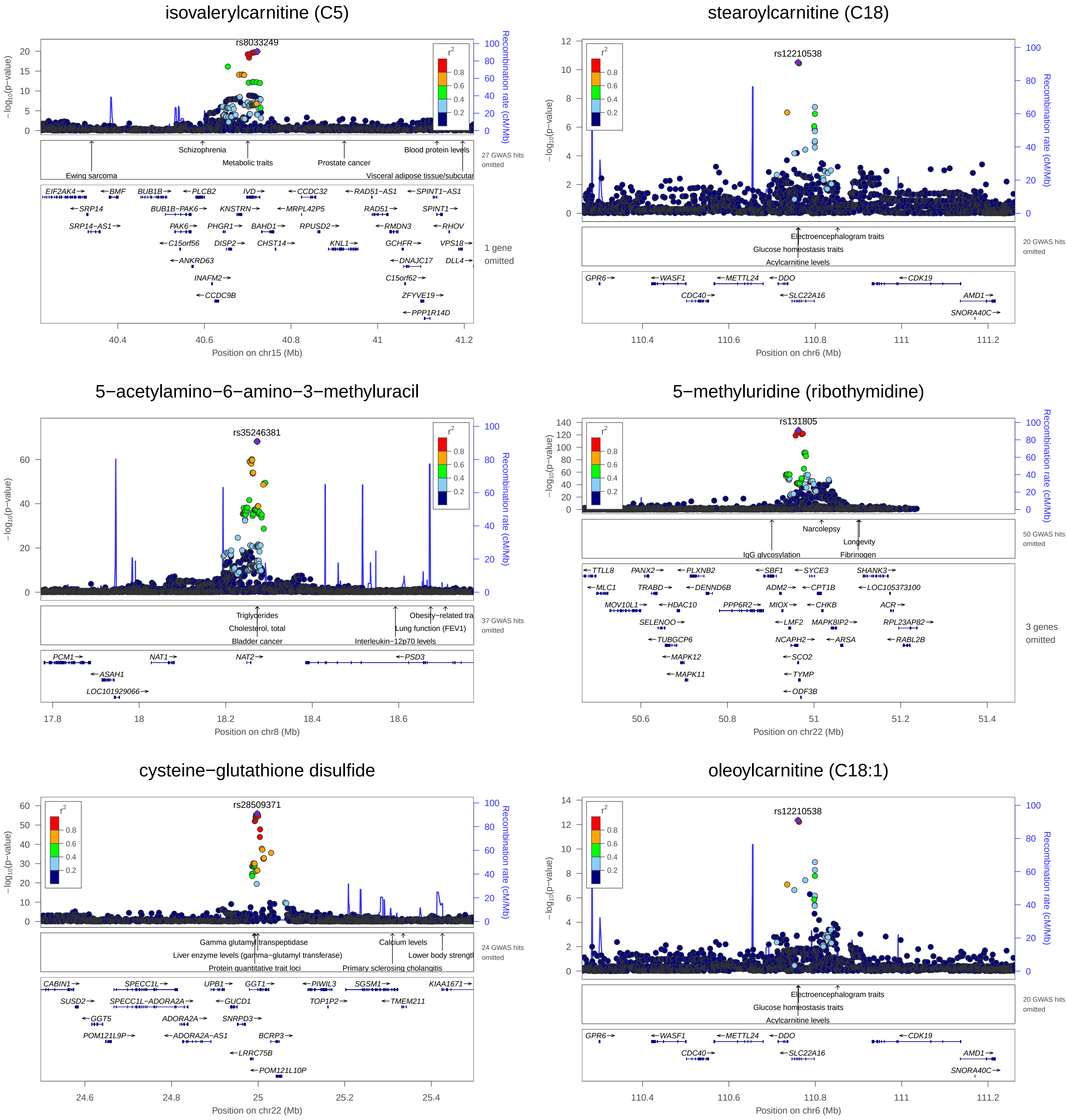

isovalerylcarnitine (C5)
rs8033249
−log10(p−value)
Recombination rate (cM/Mb)
Ewing sarcoma
Schizophrenia
Metabolic traits
Prostate cancer
Blood protein levels
Visceral adipose tissue/subcutar
27 GWAS hits omitted
EIF2AK4 BMF BUB1B PLCB2 IVD CCDC32 RAD51−AS1 SPINT1−AS1
SRP14 BUB1B−PAK6 KNSTRN MRPL42P5 RAD51 SPINT1
SRP14−AS1 PAK6 PHGR1 BAHD1 RPUSD2 RMDN3 RHOV
C15orf56 DISP2 CHST14 KNL1 GCHFR VPS18
ANKRD63 DNAJC17 DLL4
INAFM2 C15orf62
CCDC9B ZFYVE19
PPP1R14D
1 gene omitted
Position on chr15 (Mb)
stearoylcarnitine (C18)
rs12210538
Electroencephalogram traits
Glucose homeostasis traits
Acylcarnitine levels
20 GWAS hits omitted
GPR6 WASF1 METTL24 DDO CDK19
CDC40 SLC22A16 AMD1
SNORA40C
Position on chr6 (Mb)
5−acetylamino−6−amino−3−methyluracil
rs35246381
Triglycerides
Cholesterol, total
Bladder cancer
Obesity−related tra
Lung function (FEV1)
Interleukin−12p70 levels
37 GWAS hits omitted
PCM1 NAT1 NAT2 PSD3
ASAH1
LOC101929066
Position on chr8 (Mb)
5−methyluridine (ribothymidine)
rs131805
Narcolepsy
Longevity
IgG glycosylation
Fibrinogen
50 GWAS hits omitted
TTLL8 PANX2 PLXNB2 SBF1 SYCE3 SHANK3
MLC1 TRABD DENND6B ADM2 CPT1B LOC105373100
MOV10L1 HDAC10 PPP6R2 MIOX CHKB ACR
SELENOO LMF2 MAPK8IP2 RPL23AP82
TUBGCP6 NCAPH2 ARSA RABL2B
MAPK12 SCO2
MAPK11 TYMP
ODF3B
3 genes omitted
Position on chr22 (Mb)
cysteine−glutathione disulfide
rs28509371
Gamma glutamyl transpeptidase
Liver enzyme levels (gamma−glutamyl transferase)
Protein quantitative trait loci
Calcium levels
Lower body strengtl
Primary sclerosing cholangitis
24 GWAS hits omitted
CABIN1 SPECC1L UPB1 GGT1 PIWIL3 SGSM1 KIAA1671
SUSD2 SPECC1L−ADORA2A GUCD1 TOP1P2 TMEM211
GGT5 ADORA2A SNRPD3
POM121L9P ADORA2A−AS1 BCRP3
LRRC75B
POM121L10P
Position on chr22 (Mb)
oleoylcarnitine (C18:1)
rs12210538
Electroencephalogram traits
Glucose homeostasis traits
Acylcarnitine levels
20 GWAS hits omitted
GPR6 WASF1 METTL24 DDO CDK19
CDC40 SLC22A16 AMD1
SNORA40C
Position on chr6 (Mb)

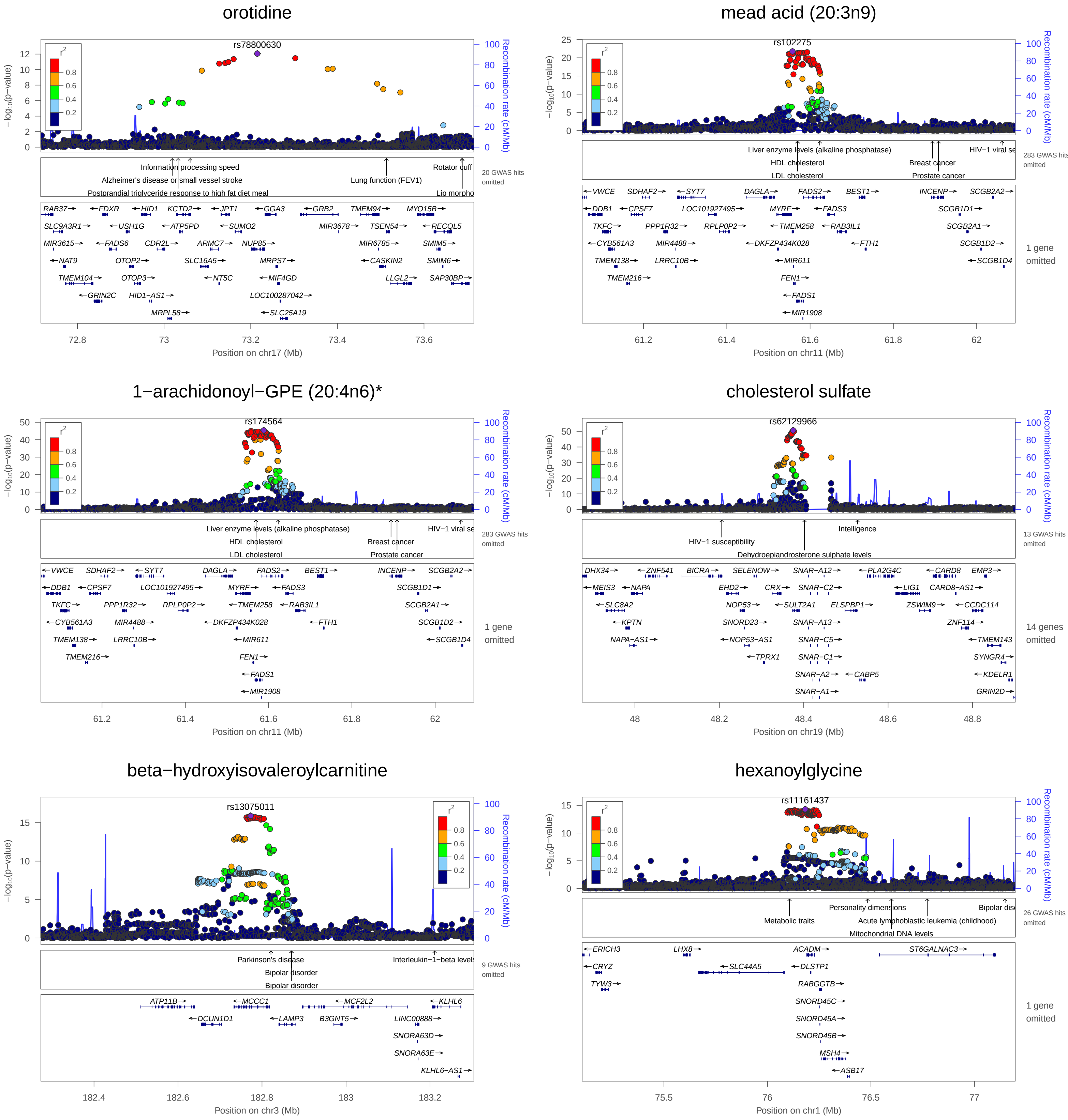
orotidine
rs78800630
Information processing speed
Alzheimer's disease or small vessel stroke
Postprandial triglyceride response to high fat diet meal
Lung function (FEV1)
Rotator cuff
Lip morpho
20 GWAS hits omitted
Position on chr17 (Mb)
mead acid (20:3n9)
rs102275
Liver enzyme levels (alkaline phosphatase)
HDL cholesterol
LDL cholesterol
Breast cancer
Prostate cancer
HIV−1 viral se
283 GWAS hits omitted
1 gene omitted
Position on chr11 (Mb)
1−arachidonoyl−GPE (20:4n6)*
rs174564
Liver enzyme levels (alkaline phosphatase)
HDL cholesterol
LDL cholesterol
Breast cancer
Prostate cancer
HIV−1 viral se
283 GWAS hits omitted
1 gene omitted
Position on chr11 (Mb)
cholesterol sulfate
rs62129966
HIV−1 susceptibility
Intelligence
Dehydroepiandrosterone sulphate levels
13 GWAS hits omitted
14 genes omitted
Position on chr19 (Mb)
beta−hydroxyisovaleroylcarnitine
rs13075011
Parkinson's disease
Bipolar disorder
Bipolar disorder
Interleukin−1−beta levels
9 GWAS hits omitted
Position on chr3 (Mb)
hexanoylglycine
rs11161437
Metabolic traits
Personality dimensions
Acute lymphoblastic leukemia (childhood)
Mitochondrial DNA levels
Bipolar dis
26 GWAS hits omitted
1 gene omitted
Position on chr1 (Mb)
−log10(p−value)
Recombination rate (cM/Mb)
r2

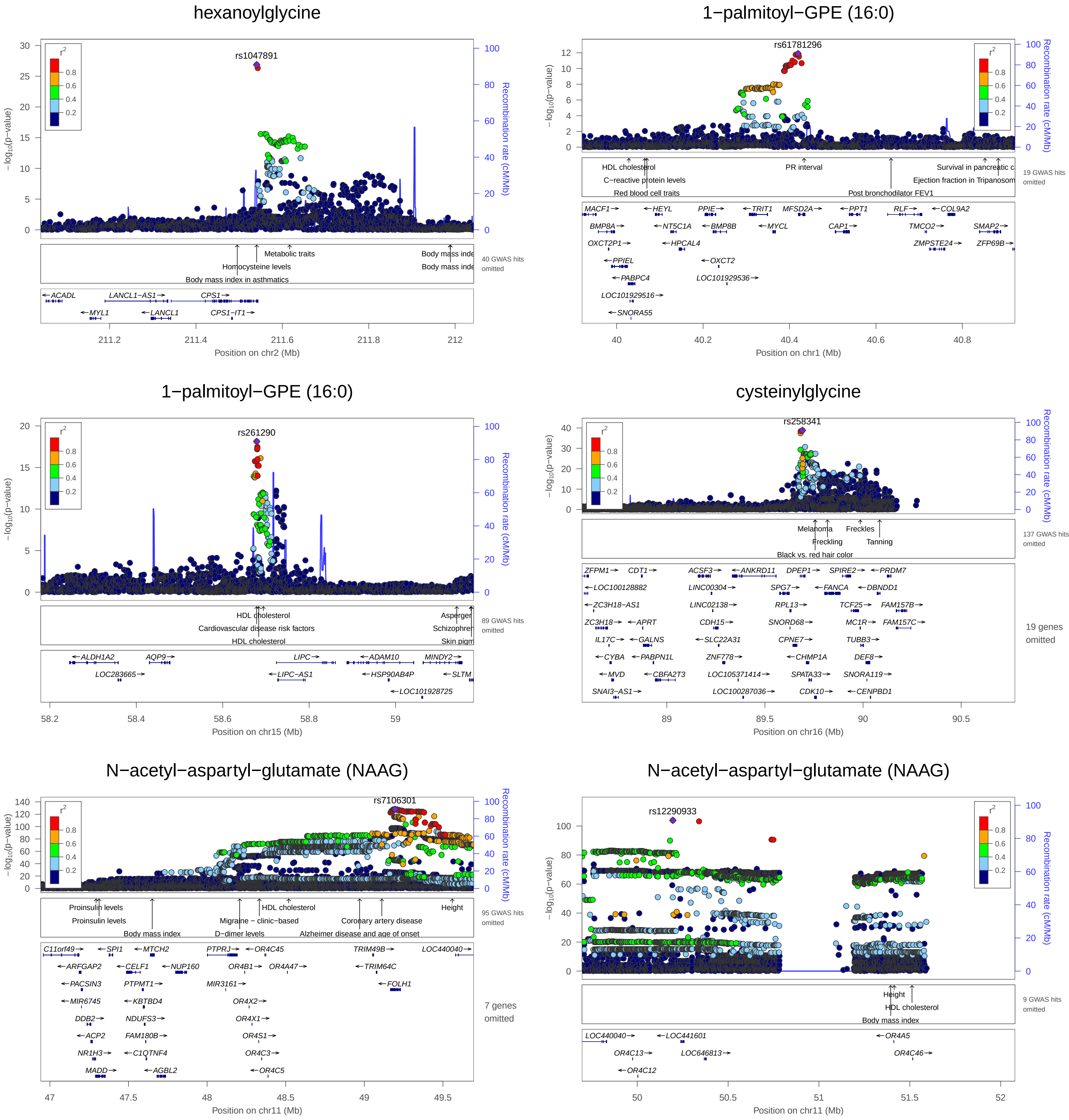
hexanoylglycine
rs1047891
−log10(p−value)
Recombination rate (cM/Mb)
Metabolic traits
Homocysteine levels
Body mass index in asthmatics
Body mass inde
40 GWAS hits omitted
ACADL
LANCL1−AS1
CPS1
MYL1
LANCL1
CPS1−IT1
Position on chr2 (Mb)
1−palmitoyl−GPE (16:0)
rs61781296
HDL cholesterol
C−reactive protein levels
Red blood cell traits
PR interval
Post bronchodilator FEV1
Survival in pancreatic c
Ejection fraction in Tripanosom
19 GWAS hits omitted
MACF1
HEYL
PPIE
TRIT1
MFSD2A
PPT1
RLF
COL9A2
BMP8A
NT5C1A
BMP8B
MYCL
CAP1
TMCO2
SMAP2
OXCT2P1
HPCAL4
ZMPSTE24
ZFP69B
PPIEL
OXCT2
PABPC4
LOC101929536
LOC101929516
SNORA55
Position on chr1 (Mb)
1−palmitoyl−GPE (16:0)
rs261290
HDL cholesterol
Cardiovascular disease risk factors
Asperger
Schizophre
Skin pigm
89 GWAS hits omitted
ALDH1A2
AQP9
LIPC
ADAM10
MINDY2
LOC283665
LIPC−AS1
HSP90AB4P
SLTM
LOC101928725
Position on chr15 (Mb)
cysteinylglycine
rs258341
Melanoma
Freckles
Freckling
Tanning
Black vs. red hair color
137 GWAS hits omitted
ZFPM1
CDT1
ACSF3
ANKRD11
DPEP1
SPIRE2
PRDM7
LOC100128882
LINC00304
SPG7
FANCA
DBNDD1
ZC3H18−AS1
LINC02138
RPL13
TCF25
FAM157B
ZC3H18
APRT
CDH15
SNORD68
MC1R
FAM157C
IL17C
GALNS
SLC22A31
CPNE7
TUBB3
CYBA
PABPN1L
ZNF778
CHMP1A
DEF8
MVD
CBFA2T3
LOC105371414
SPATA33
SNORA119
SNAI3−AS1
LOC100287036
CDK10
CENPBD1
19 genes omitted
Position on chr16 (Mb)
N−acetyl−aspartyl−glutamate (NAAG)
rs7106301
Proinsulin levels
Body mass index
Migraine − clinic−based
D−dimer levels
HDL cholesterol
Coronary artery disease
Alzheimer disease and age of onset
Height
95 GWAS hits omitted
C11orf49
SPI1
MTCH2
PTPRJ
OR4C45
TRIM49B
LOC440040
ARFGAP2
CELF1
NUP160
OR4B1
OR4A47
TRIM64C
PACSIN3
PTPMT1
MIR3161
FOLH1
MIR6745
KBTBD4
OR4X2
DDB2
NDUFS3
OR4X1
ACP2
FAM180B
OR4S1
NR1H3
C1QTNF4
OR4C3
MADD
AGBL2
OR4C5
7 genes omitted
Position on chr11 (Mb)
N−acetyl−aspartyl−glutamate (NAAG)
rs12290933
Height
HDL cholesterol
Body mass index
9 GWAS hits omitted
LOC440040
LOC441601
OR4A5
OR4C13
LOC646813
OR4C46
OR4C12
Position on chr11 (Mb)

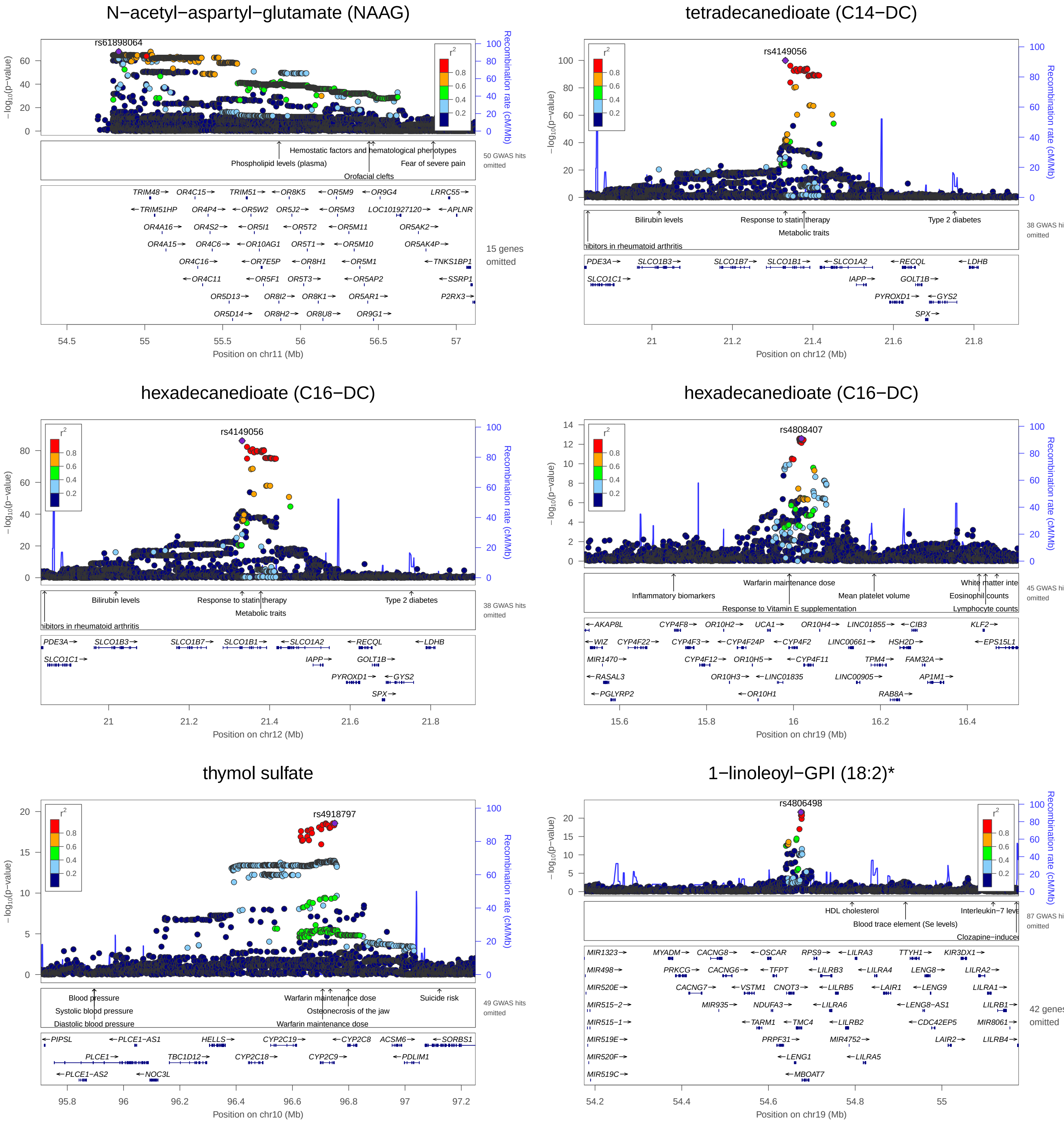

N−acetyl−aspartyl−glutamate (NAAG)
rs61898064
Hemostatic factors and hematological phenotypes
Phospholipid levels (plasma)
Fear of severe pain
Orofacial clefts
50 GWAS hits omitted
15 genes omitted
Position on chr11 (Mb)
tetradecanedioate (C14−DC)
rs4149056
Bilirubin levels
Response to statin therapy
Type 2 diabetes
Metabolic traits
38 GWAS hits omitted
Position on chr12 (Mb)
hexadecanedioate (C16−DC)
rs4149056
Bilirubin levels
Response to statin therapy
Type 2 diabetes
Metabolic traits
38 GWAS hits omitted
Position on chr12 (Mb)
hexadecanedioate (C16−DC)
rs4808407
Warfarin maintenance dose
Inflammatory biomarkers
Mean platelet volume
Eosinophil counts
Response to Vitamin E supplementation
Lymphocyte counts
45 GWAS hits omitted
Position on chr19 (Mb)
thymol sulfate
rs4918797
Blood pressure
Systolic blood pressure
Diastolic blood pressure
Warfarin maintenance dose
Osteonecrosis of the jaw
Suicide risk
49 GWAS hits omitted
Position on chr10 (Mb)
1−linoleoyl−GPI (18:2)*
rs4806498
HDL cholesterol
Blood trace element (Se levels)
87 GWAS hits omitted
42 genes omitted
Position on chr19 (Mb)
−log10(p−value)
Recombination rate (cM/Mb)

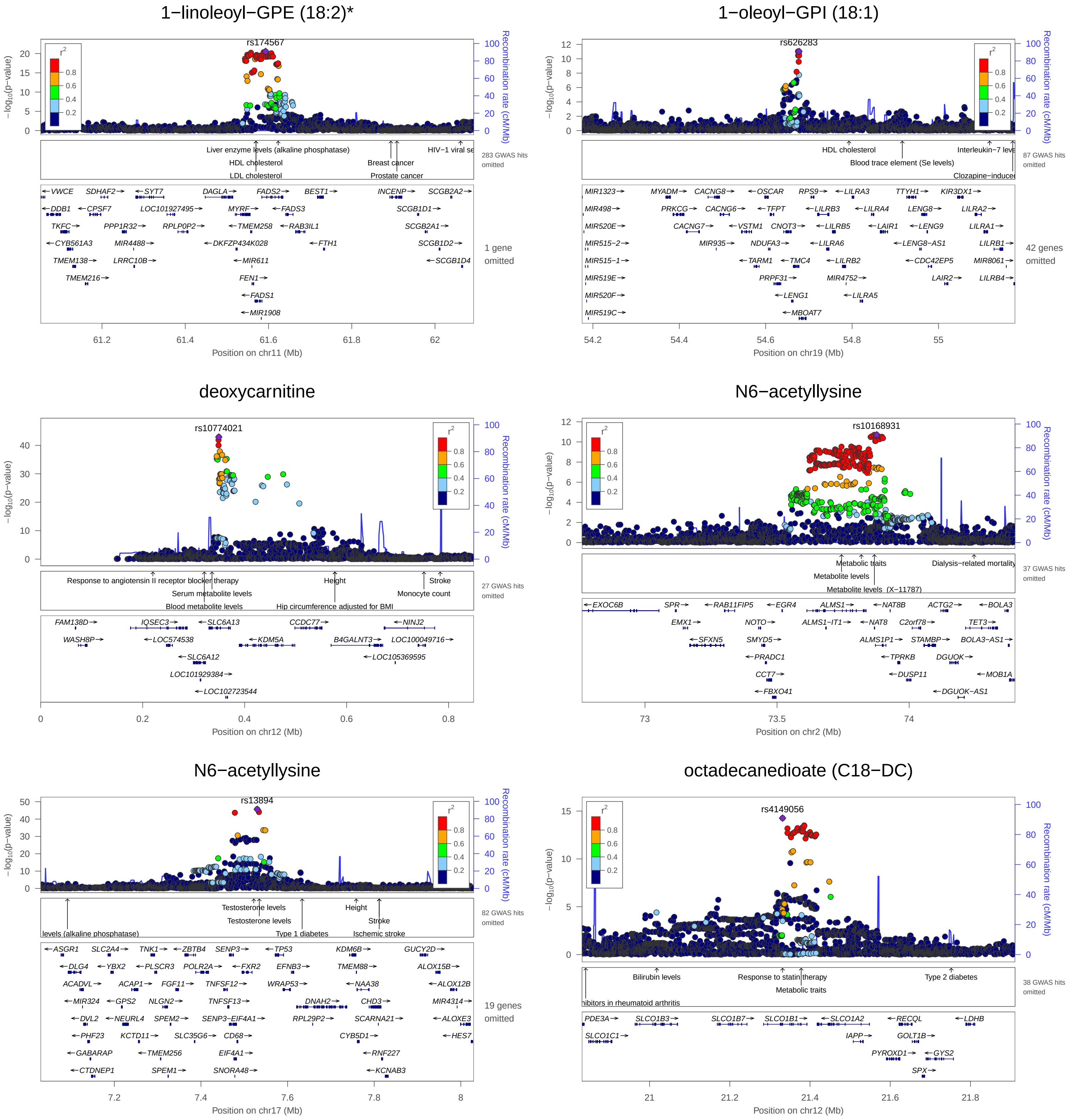

1−linoleoyl−GPE (18:2)*
rs174567
−log10(p−value)
Recombination rate (cM/Mb)
r2
Liver enzyme levels (alkaline phosphatase)
HDL cholesterol
LDL cholesterol
Breast cancer
Prostate cancer
HIV−1 viral se
283 GWAS hits omitted
VWCE SDHAF2 SYT7 DAGLA FADS2 BEST1 INCENP SCGB2A2
DDB1 CPSF7 LOC101927495 MYRF FADS3 SCGB1D1
TKFC PPP1R32 RPLP0P2 TMEM258 RAB3IL1 SCGB2A1
CYB561A3 MIR4488 DKFZP434K028 FTH1 SCGB1D2
TMEM138 LRRC10B MIR611 SCGB1D4
TMEM216 FEN1 FADS1 MIR1908
1 gene omitted
Position on chr11 (Mb)
1−oleoyl−GPI (18:1)
rs626283
HDL cholesterol
Blood trace element (Se levels)
Interleukin−7 leve
Clozapine−induce
87 GWAS hits omitted
MIR1323 MYADM CACNG8 OSCAR RPS9 LILRA3 TTYH1 KIR3DX1
MIR498 PRKCG CACNG6 TFPT LILRB3 LILRA4 LENG8 LILRA2
MIR520E CACNG7 VSTM1 CNOT3 LILRB5 LAIR1 LENG9 LILRA1
MIR515−2 MIR935 NDUFA3 LILRA6 LENG8−AS1 LILRB1
MIR515−1 TARM1 TMC4 LILRB2 CDC42EP5 MIR8061
MIR519E PRPF31 MIR4752 LAIR2 LILRB4
MIR520F LENG1 LILRA5
MIR519C MBOAT7
42 genes omitted
Position on chr19 (Mb)
deoxycarnitine
rs10774021
Response to angiotensin II receptor blocker therapy
Serum metabolite levels
Blood metabolite levels
Height
Hip circumference adjusted for BMI
Stroke
Monocyte count
27 GWAS hits omitted
FAM138D IQSEC3 SLC6A13 CCDC77 NINJ2
WASH8P LOC574538 KDM5A B4GALNT3 LOC100049716
SLC6A12 LOC105369595
LOC101929384
LOC102723544
Position on chr12 (Mb)
N6−acetyllysine
rs10168931
Metabolic traits
Metabolite levels
Metabolite levels (X−11787)
Dialysis−related mortality
37 GWAS hits omitted
EXOC6B SPR RAB11FIP5 EGR4 ALMS1 NAT8B TET2 BOLA3
EMX1 NOTO ALMS1−IT1 NAT8 C2orf78 TET3
SFXN5 SMYD5 ALMS1P1 STAMBP BOLA3−AS1
PRADC1 TPRKB DGUOK
CCT7 DUSP11 MOB1A
FBXO41 DGUOK−AS1
Position on chr2 (Mb)
N6−acetyllysine
rs13894
levels (alkaline phosphatase)
Testosterone levels
Testosterone levels
Type 1 diabetes
Height
Stroke
Ischemic stroke
82 GWAS hits omitted
ASGR1 SLC2A4 TNK1 ZBTB4 SENP3 TP53 KDM6B GUCY2D
DLG4 YBX2 PLSCR3 POLR2A FXR2 EFNB3 TMEM88 ALOX15B
ACADVL ACAP1 FGF11 TNFSF12 WRAP53 NAA38 ALOX12B
MIR324 GPS2 NLGN2 TNFSF13 DNAH2 CHD3 MIR4314
DVL2 NEURL4 SPEM2 SENP3−EIF4A1 RPL29P2 SCARNA21 ALOXE3
PHF23 KCTD11 SLC35G6 CD68 CYB5D1 HES7
GABARAP TMEM256 EIF4A1 RNF227
CTDNEP1 SPEM1 SNORA48 KCNAB3
19 genes omitted
Position on chr17 (Mb)
octadecanedioate (C18−DC)
rs4149056
Bilirubin levels
Response to statin therapy
Metabolic traits
Type 2 diabetes
hibitors in rheumatoid arthritis
38 GWAS hits omitted
PDE3A SLCO1B3 SLCO1B7 SLCO1B1 SLCO1A2 RECQL LDHB
SLCO1C1 IAPP GOLT1B
PYROXD1 GYS2
SPX
Position on chr12 (Mb)

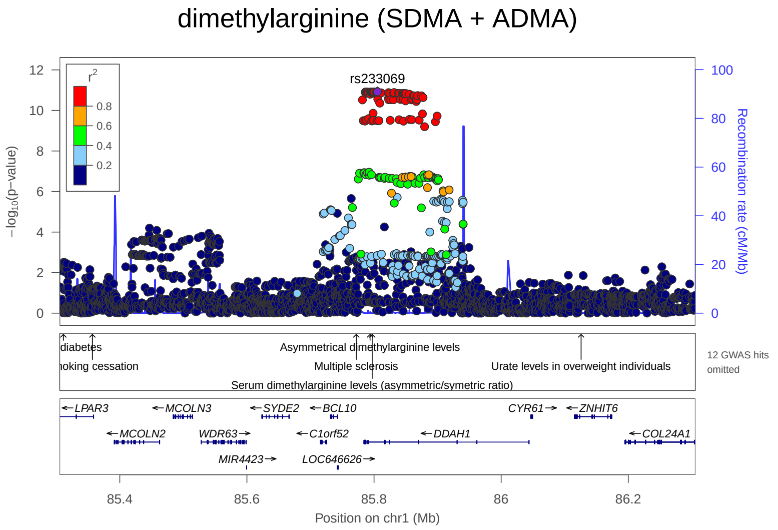
dimethylarginine (SDMA + ADMA)
rs233069
−log10(p−value)
Recombination rate (cM/Mb)
r2
0.8
0.6
0.4
0.2
diabetes
noking cessation
Asymmetrical dimethylarginine levels
Multiple sclerosis
Urate levels in overweight individuals
Serum dimethylarginine levels (asymmetric/symetric ratio)
12 GWAS hits omitted
LPAR3
MCOLN3
SYDE2
BCL10
CYR61
ZNHIT6
MCOLN2
WDR63
C1orf52
DDAH1
COL24A1
MIR4423
LOC646626
85.4
85.6
85.8
86
86.2
Position on chr1 (Mb)

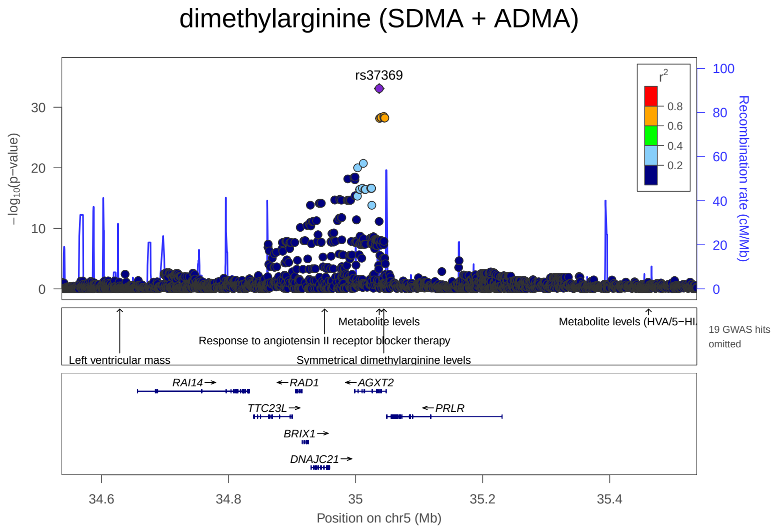
dimethylarginine (SDMA + ADMA)
rs37369
−log10(p−value)
Recombination rate (cM/Mb)
Left ventricular mass
Metabolite levels
Response to angiotensin II receptor blocker therapy
Symmetrical dimethylarginine levels
Metabolite levels (HVA/5−HI
19 GWAS hits omitted
RAI14
RAD1
AGXT2
TTC23L
PRLR
BRIX1
DNAJC21
34.6
34.8
35
35.2
35.4
Position on chr5 (Mb)

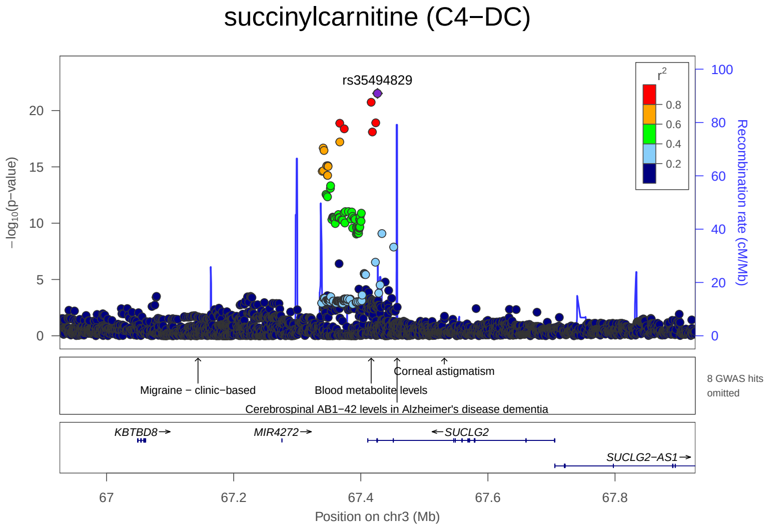
succinylcarnitine (C4−DC)
rs35494829
−log10(p−value)
Recombination rate (cM/Mb)
Migraine − clinic−based
Blood metabolite levels
Corneal astigmatism
Cerebrospinal AB1−42 levels in Alzheimer's disease dementia
8 GWAS hits omitted
KBTBD8
MIR4272
SUCLG2
SUCLG2−AS1
67
67.2
67.4
67.6
67.8
Position on chr3 (Mb)

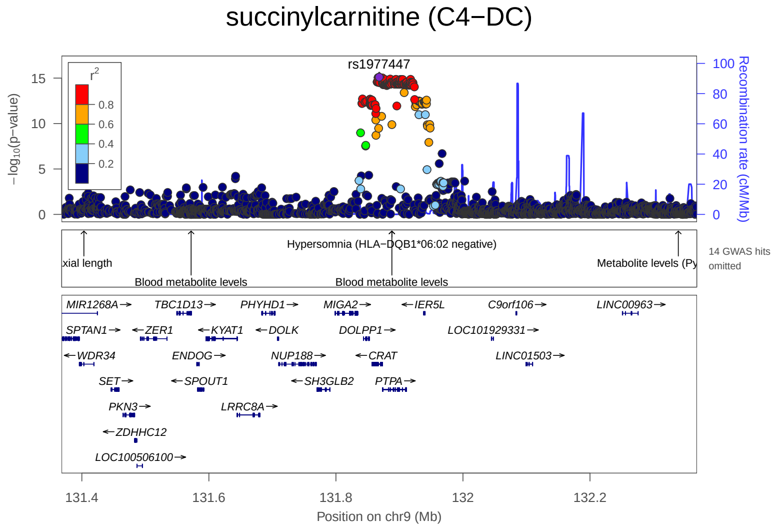
succinylcarnitine (C4−DC)
rs1977447
−log10(p−value)
Recombination rate (cM/Mb)
xial length
Blood metabolite levels
Hypersomnia (HLA−DQB1*06:02 negative)
Blood metabolite levels
Metabolite levels (Py
14 GWAS hits omitted
MIR1268A
TBC1D13
PHYHD1
MIGA2
IER5L
C9orf106
LINC00963
SPTAN1
ZER1
KYAT1
DOLK
DOLPP1
LOC101929331
WDR34
ENDOG
NUP188
CRAT
LINC01503
SET
SPOUT1
SH3GLB2
PTPA
PKN3
LRRC8A
ZDHHC12
LOC100506100
131.4
131.6
131.8
132
132.2
Position on chr9 (Mb)

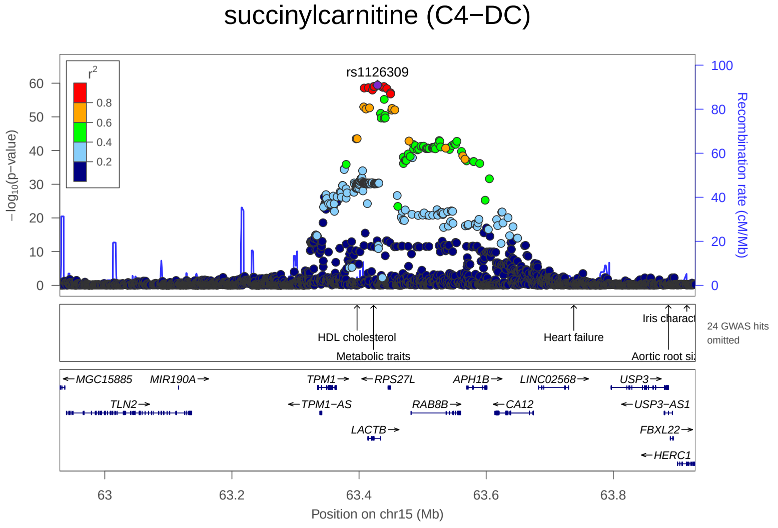
succinylcarnitine (C4−DC)
rs1126309
−log10(p−value)
Recombination rate (cM/Mb)
HDL cholesterol
Metabolic traits
Heart failure
Iris charact
Aortic root si
24 GWAS hits omitted
MGC15885
MIR190A
TPM1
RPS27L
APH1B
LINC02568
USP3
TLN2
TPM1−AS
RAB8B
CA12
USP3−AS1
LACTB
FBXL22
HERC1
63
63.2
63.4
63.6
63.8
Position on chr15 (Mb)

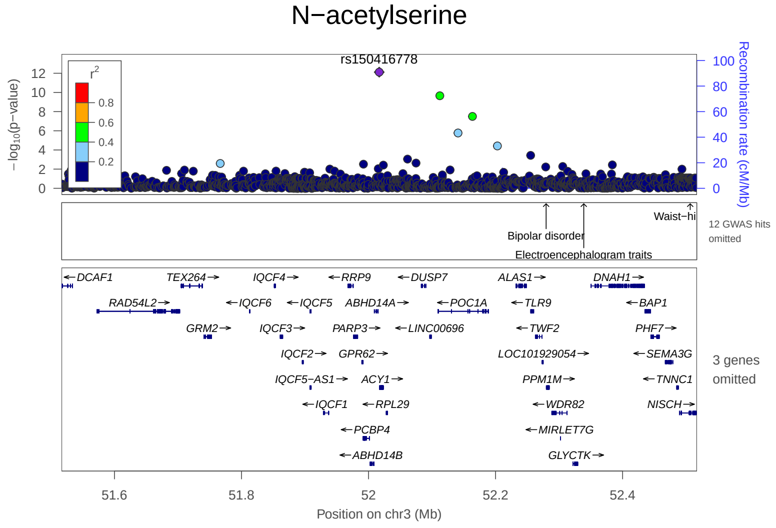
N−acetylserine
rs150416778
−log10(p−value)
Recombination rate (cM/Mb)
Bipolar disorder
Electroencephalogram traits
Waist−hi
12 GWAS hits omitted
DCAF1
TEX264
IQCF4
RRP9
DUSP7
ALAS1
DNAH1
RAD54L2
IQCF6
IQCF5
ABHD14A
POC1A
TLR9
BAP1
GRM2
IQCF3
PARP3
LINC00696
TWF2
PHF7
IQCF2
GPR62
LOC101929054
SEMA3G
IQCF5−AS1
ACY1
PPM1M
TNNC1
IQCF1
RPL29
WDR82
NISCH
PCBP4
MIRLET7G
ABHD14B
GLYCTK
3 genes omitted
51.6
51.8
52
52.2
52.4
Position on chr3 (Mb)

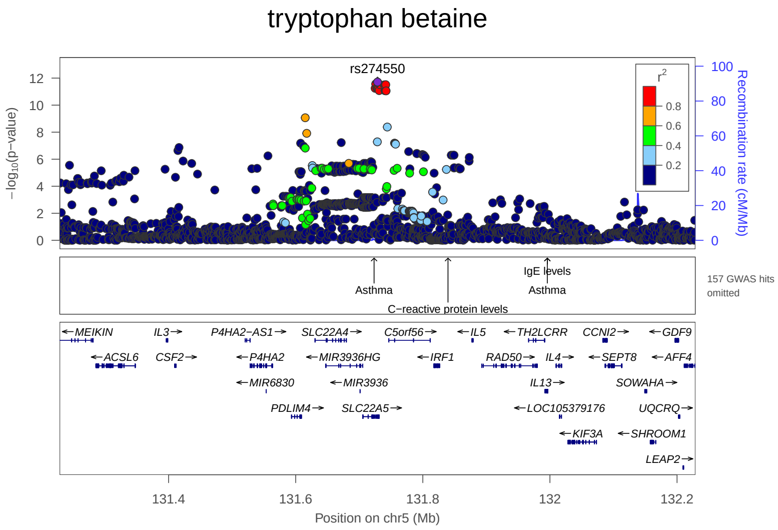
tryptophan betaine
rs274550
−log10(p−value)
Recombination rate (cM/Mb)
r2
Asthma
IgE levels
Asthma
C−reactive protein levels
157 GWAS hits omitted
MEIKIN IL3 P4HA2−AS1 SLC22A4 C5orf56 IL5 TH2LCRR CCNI2 GDF9
ACSL6 CSF2 P4HA2 MIR3936HG IRF1 RAD50 IL4 SEPT8 AFF4
MIR6830 MIR3936 IL13 SOWAHA
PDLIM4 SLC22A5 LOC105379176 UQCRQ
KIF3A SHROOM1
LEAP2
Position on chr5 (Mb)
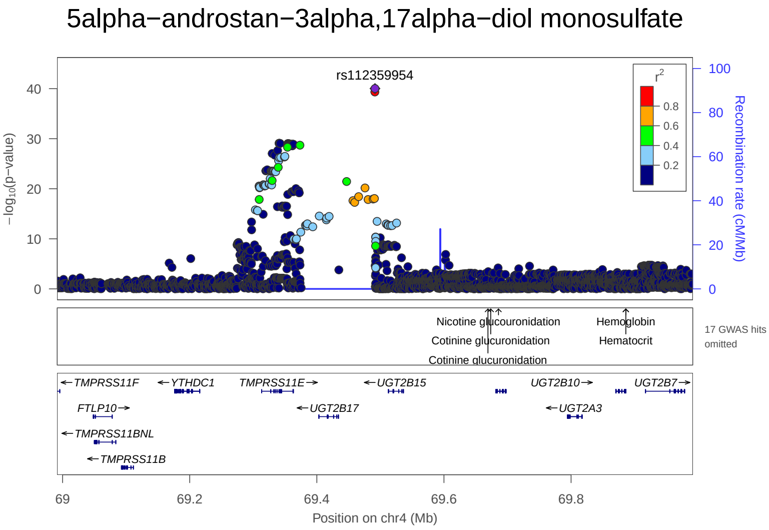
5alpha−androstan−3alpha,17alpha−diol monosulfate
rs112359954
−log10(p−value)
Recombination rate (cM/Mb)
r2
Nicotine glucouronidation
Cotinine glucuronidation
Cotinine glucuronidation
Hemoglobin
Hematocrit
17 GWAS hits omitted
TMPRSS11F YTHDC1 TMPRSS11E UGT2B15 UGT2B10 UGT2B7
FTLP10 UGT2B17 UGT2A3
TMPRSS11BNL
TMPRSS11B
Position on chr4 (Mb)
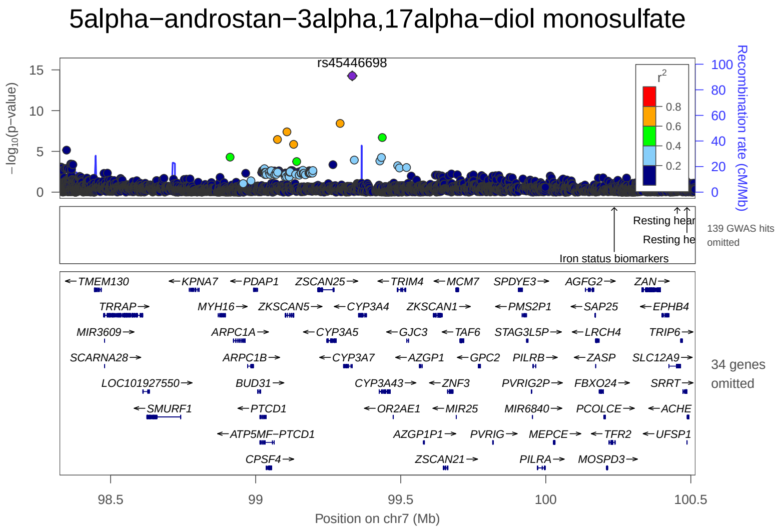
5alpha−androstan−3alpha,17alpha−diol monosulfate
rs45446698
−log10(p−value)
Recombination rate (cM/Mb)
r2
Resting hear
Resting he
Iron status biomarkers
139 GWAS hits omitted
34 genes omitted
TMEM130 KPNA7 PDAP1 ZSCAN25 TRIM4 MCM7 SPDYE3 AGFG2 ZAN
TRRAP MYH16 ZKSCAN5 CYP3A4 ZKSCAN1 PMS2P1 SAP25 EPHB4
MIR3609 ARPC1A CYP3A5 GJC3 TAF6 STAG3L5P LRCH4 TRIP6
SCARNA28 ARPC1B CYP3A7 AZGP1 GPC2 PILRB ZASP SLC12A9
LOC101927550 BUD31 CYP3A43 ZNF3 PVRIG2P FBXO24 SRRT
SMURF1 PTCD1 OR2AE1 MIR25 MIR6840 PCOLCE ACHE
ATP5MF−PTCD1 AZGP1P1 PVRIG MEPCE TFR2 UFSP1
CPSF4 ZSCAN21 PILRA MOSPD3
Position on chr7 (Mb)
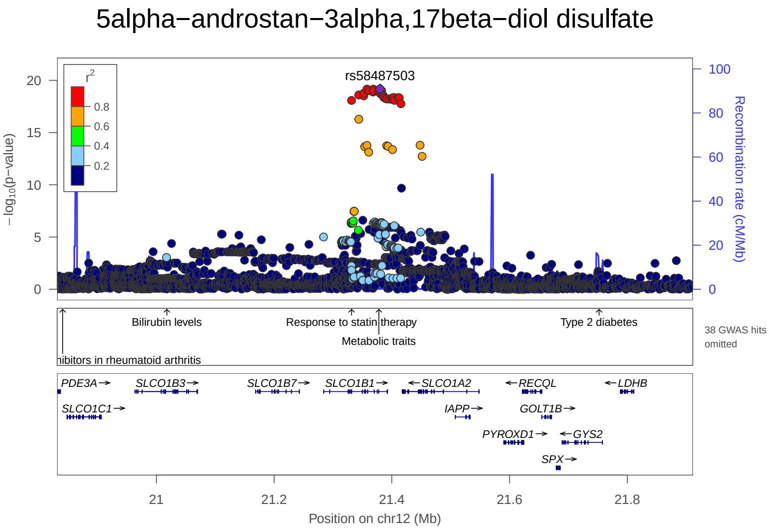
5alpha−androstan−3alpha,17beta−diol disulfate
rs58487503
−log10(p−value)
Recombination rate (cM/Mb)
r2
Bilirubin levels
Response to statin therapy
Metabolic traits
Type 2 diabetes
nibitors in rheumatoid arthritis
38 GWAS hits omitted
PDE3A SLCO1B3 SLCO1B7 SLCO1B1 SLCO1A2 RECQL LDHB
SLCO1C1 IAPP GOLT1B
PYROXD1 GYS2
SPX
Position on chr12 (Mb)
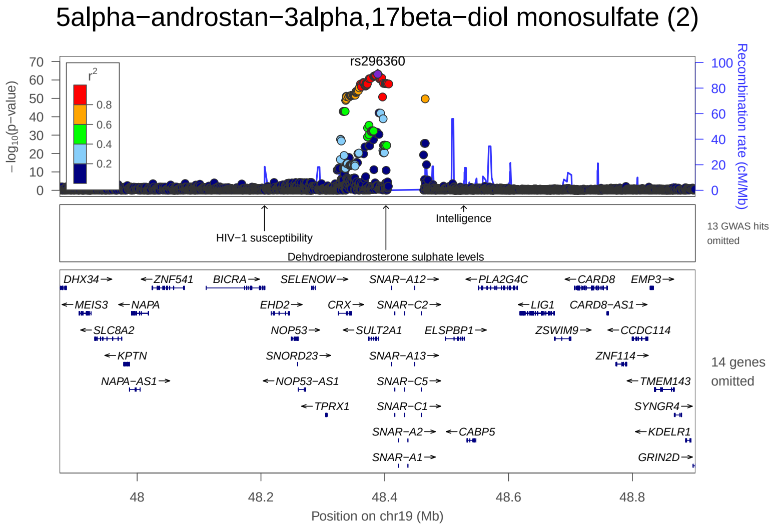
5alpha−androstan−3alpha,17beta−diol monosulfate (2)
rs296360
−log10(p−value)
Recombination rate (cM/Mb)
r2
HIV−1 susceptibility
Intelligence
Dehydroepiandrosterone sulphate levels
13 GWAS hits omitted
14 genes omitted
DHX34 ZNF541 BICRA SELENOW SNAR−A12 PLA2G4C CARD8 EMP3
MEIS3 NAPA EHD2 CRX SNAR−C2 LIG1 CARD8−AS1
SLC8A2 NOP53 SULT2A1 ELSPBP1 ZSWIM9 CCDC114
KPTN SNORD23 SNAR−A13 ZNF114
NAPA−AS1 NOP53−AS1 SNAR−C5 TMEM143
TPRX1 SNAR−C1 SYNGR4
SNAR−A2 CABP5 KDELR1
SNAR−A1 GRIN2D
Position on chr19 (Mb)
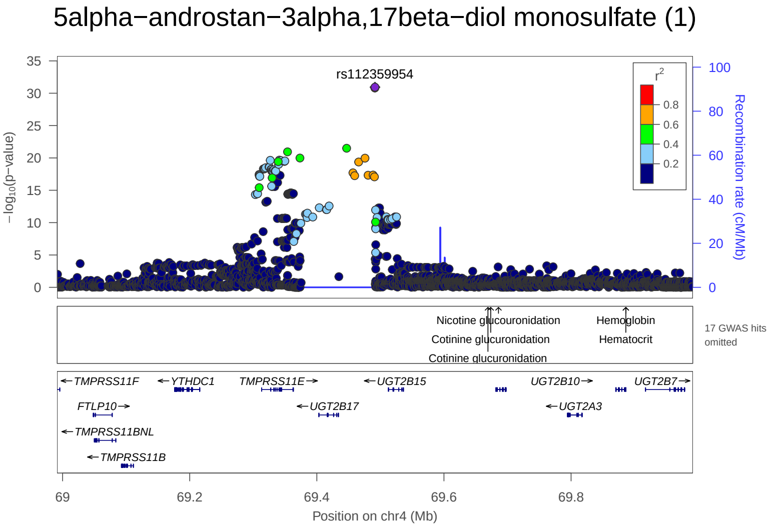
5alpha−androstan−3alpha,17beta−diol monosulfate (1)
rs112359954
−log10(p−value)
Recombination rate (cM/Mb)
r2
Nicotine glucouronidation
Cotinine glucuronidation
Cotinine glucuronidation
Hemoglobin
Hematocrit
17 GWAS hits omitted
TMPRSS11F YTHDC1 TMPRSS11E UGT2B15 UGT2B10 UGT2B7
FTLP10 UGT2B17 UGT2A3
TMPRSS11BNL
TMPRSS11B
Position on chr4 (Mb)

5alpha−androstan−3alpha,17beta−diol monosulfate (1)

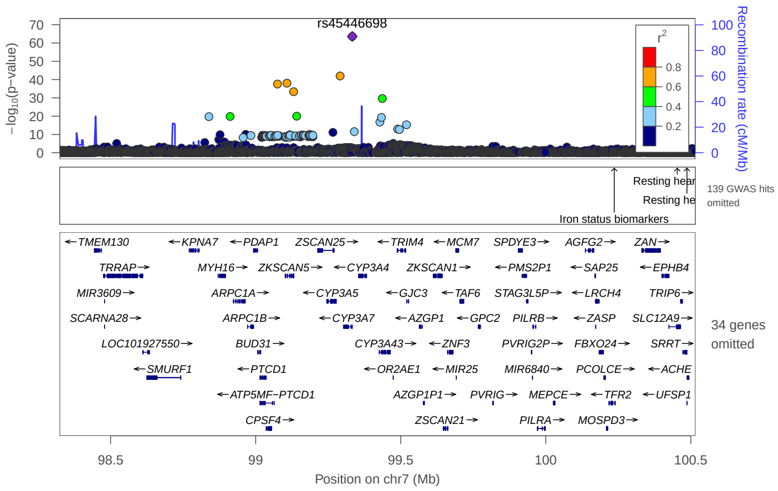

5alpha−androstan−3beta,17beta−diol disulfate

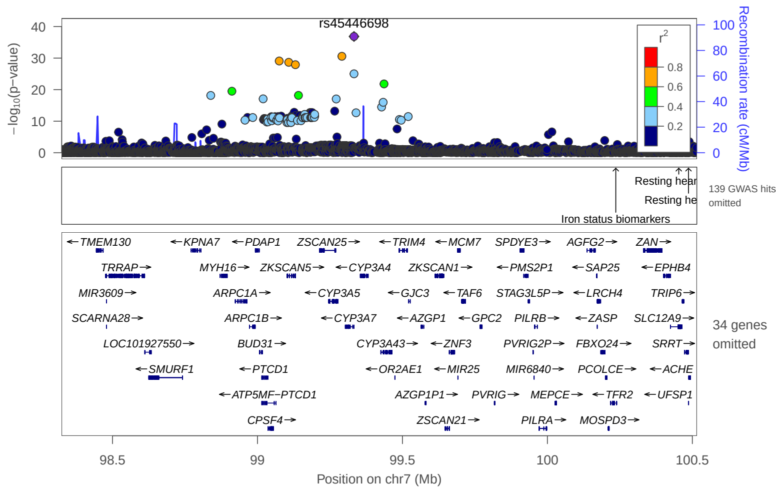

5alpha−androstan−3beta,17beta−diol disulfate

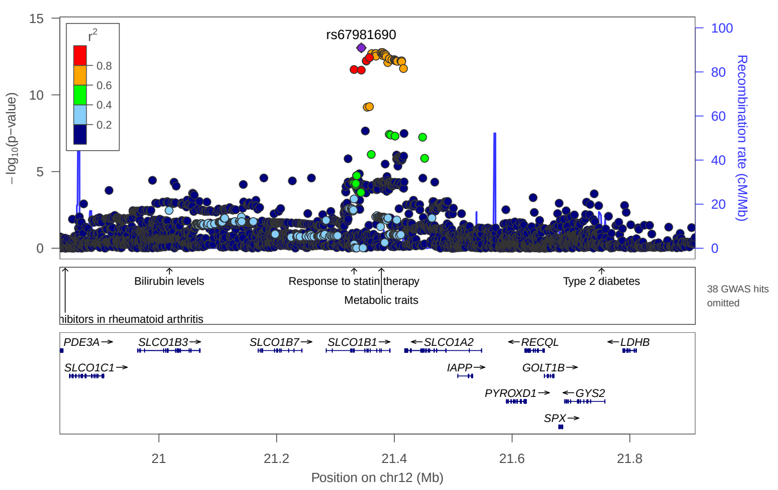

5alpha−androstan−3beta,17beta−diol disulfate

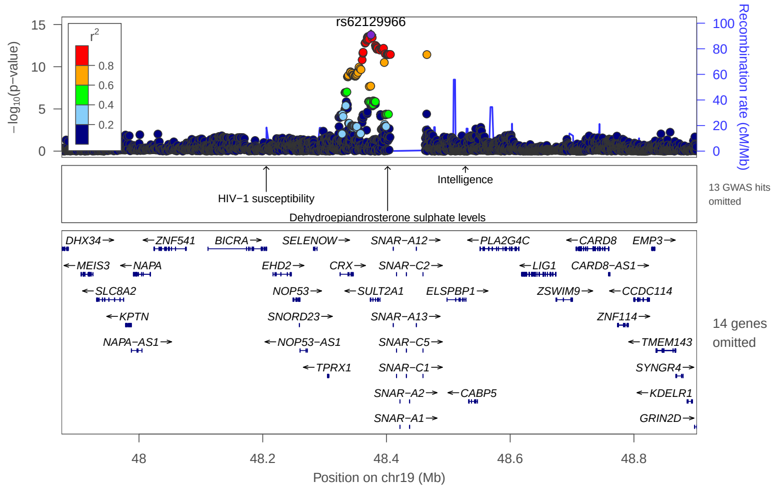

5alpha−androstan−3beta,17beta−diol monosulfate (2)

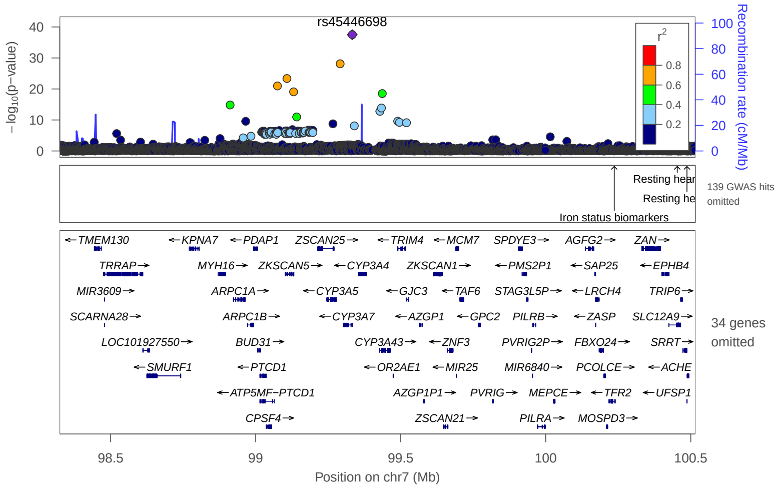

5alpha−pregnan−3beta,20beta−diol monosulfate (1)

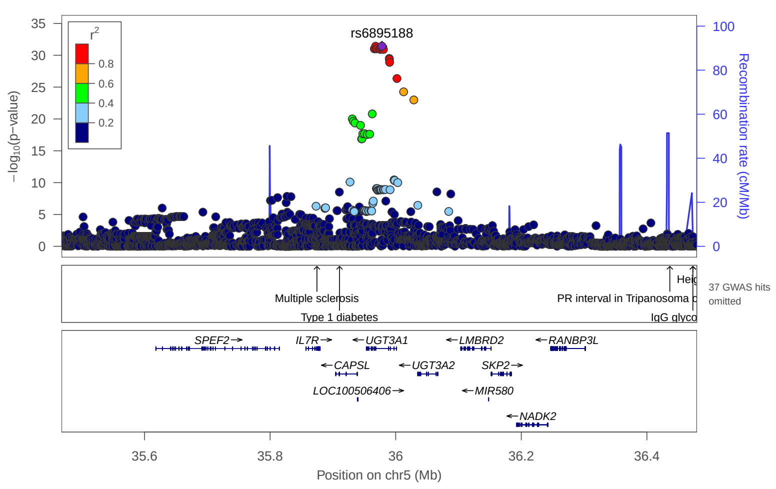

5alpha−pregnan−3beta,20alpha−diol disulfate

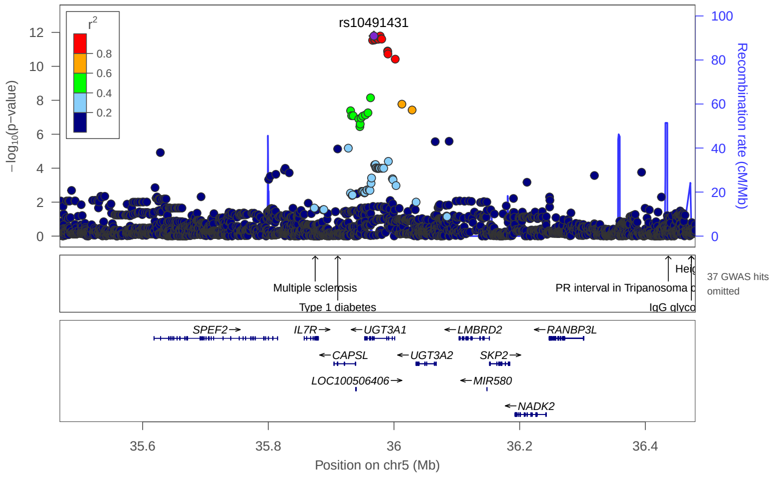

5alpha−pregnan−3beta,20alpha−diol disulfate

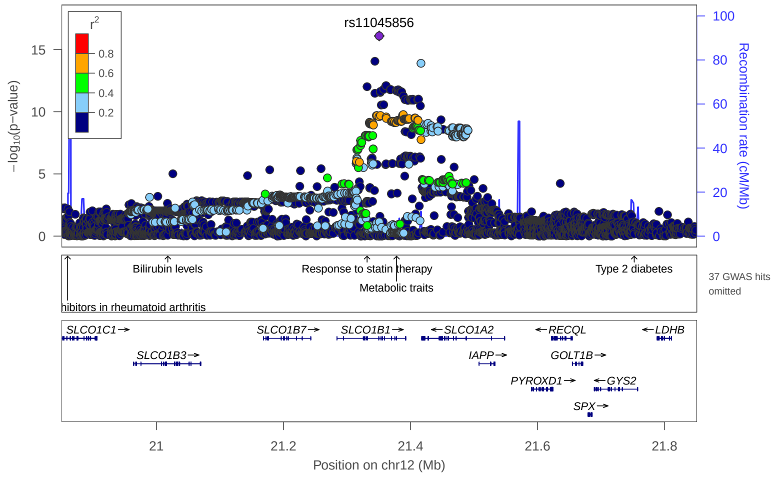

5alpha−pregnan−3beta,20alpha−diol disulfate

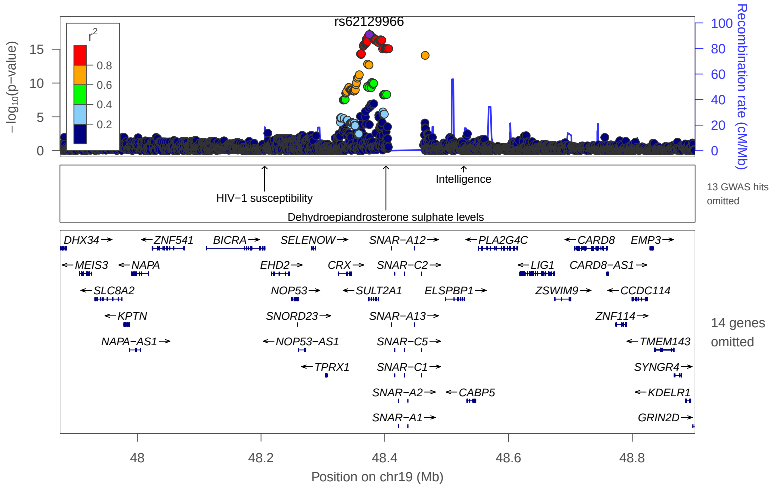

5alpha−pregnan−3beta,20alpha−diol monosulfate (2)

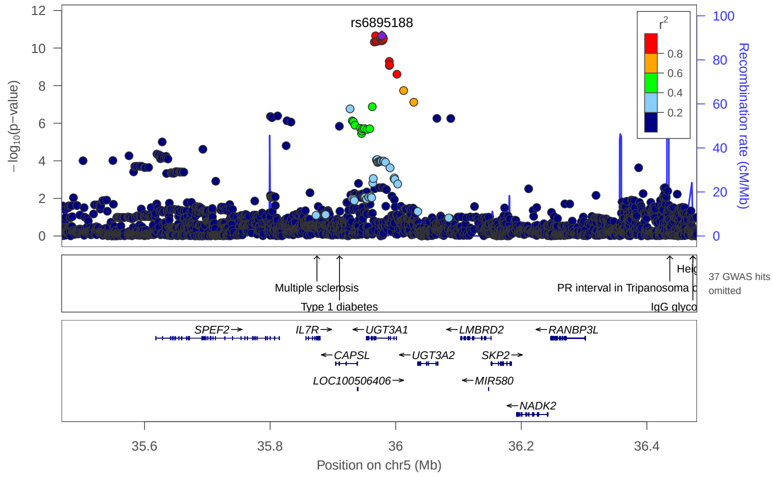

androstenediol (3beta,17beta) disulfate (1)

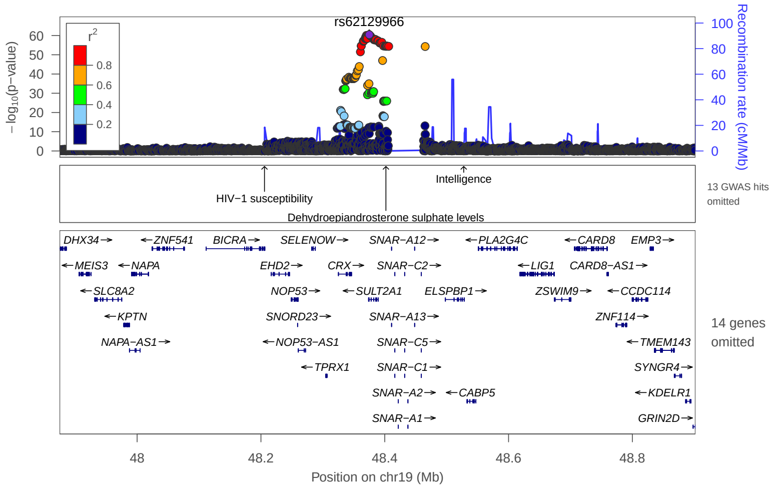

androstenediol (3beta,17beta) disulfate (2)

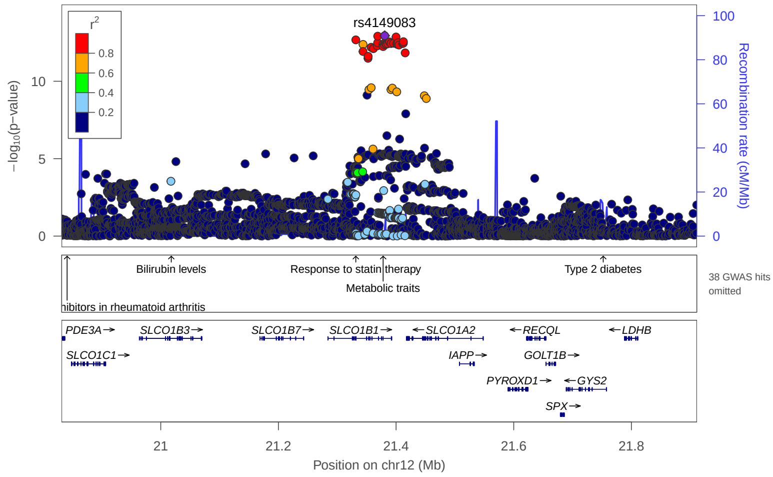

androstenediol (3alpha, 17alpha) monosulfate (2)

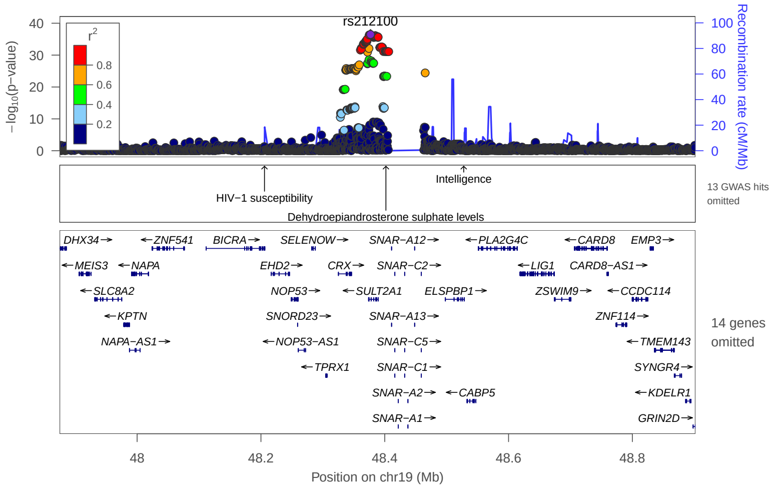

androstenediol (3alpha, 17alpha) monosulfate (3)

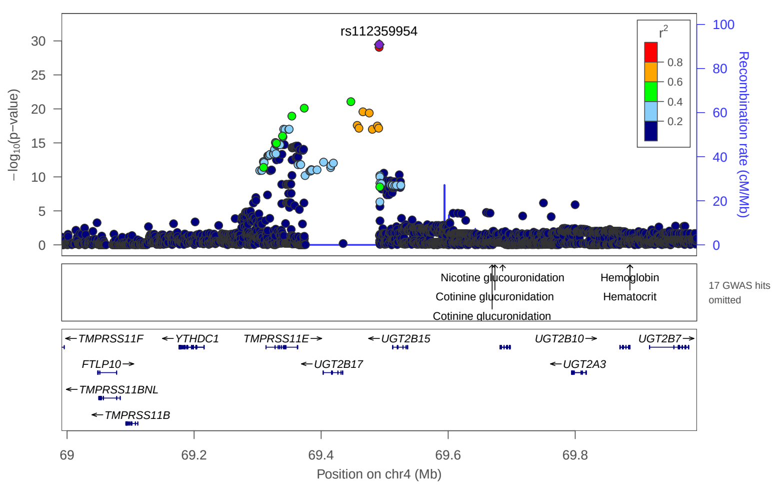

androstenediol (3alpha, 17alpha) monosulfate (3)

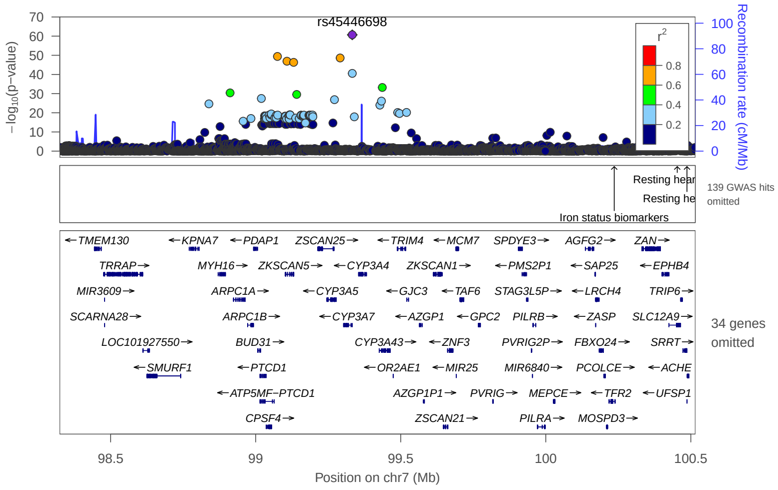

androstenediol (3alpha, 17alpha) monosulfate (3)

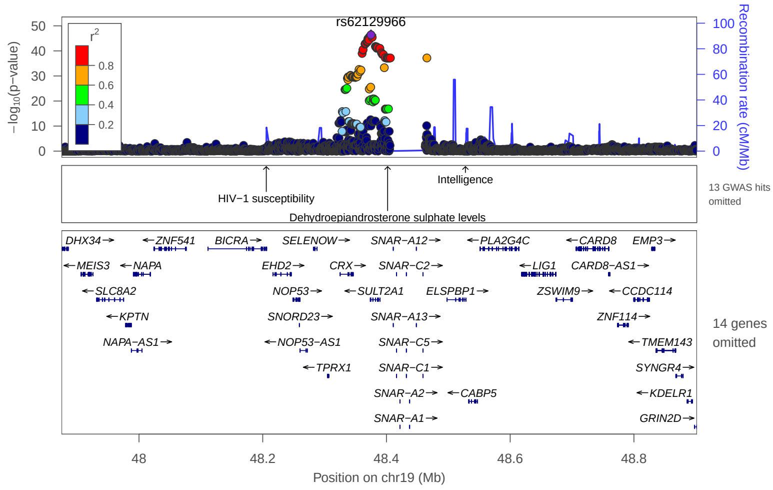

androstenediol (3beta,17beta) monosulfate (1)

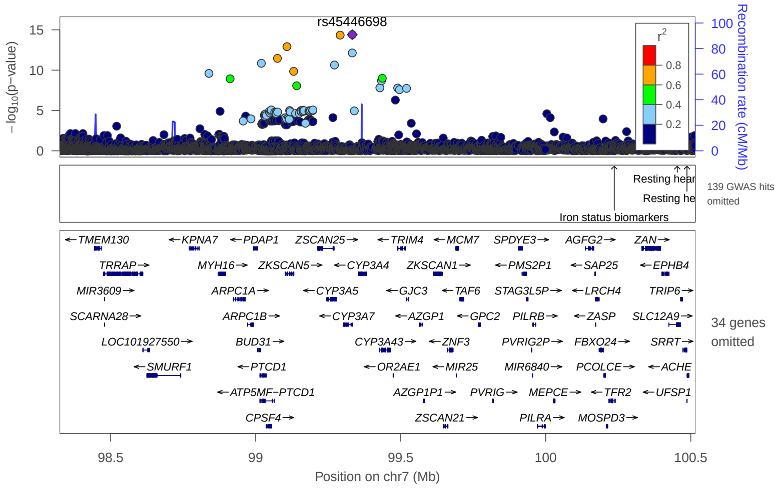

N−acetyl−beta−alanine

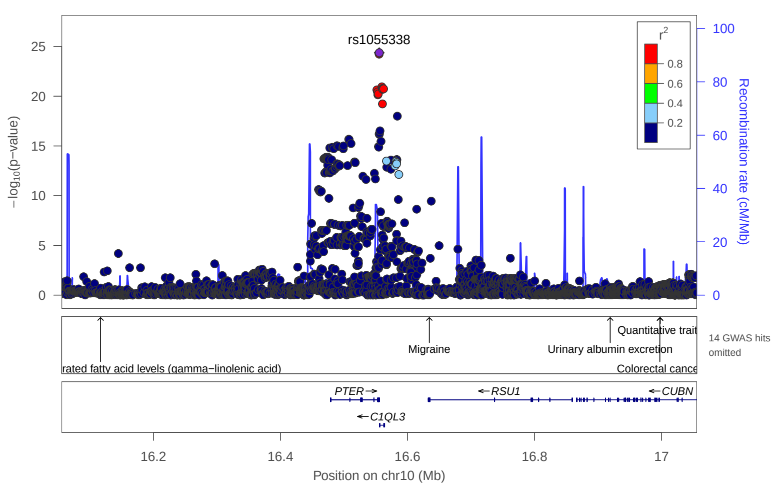

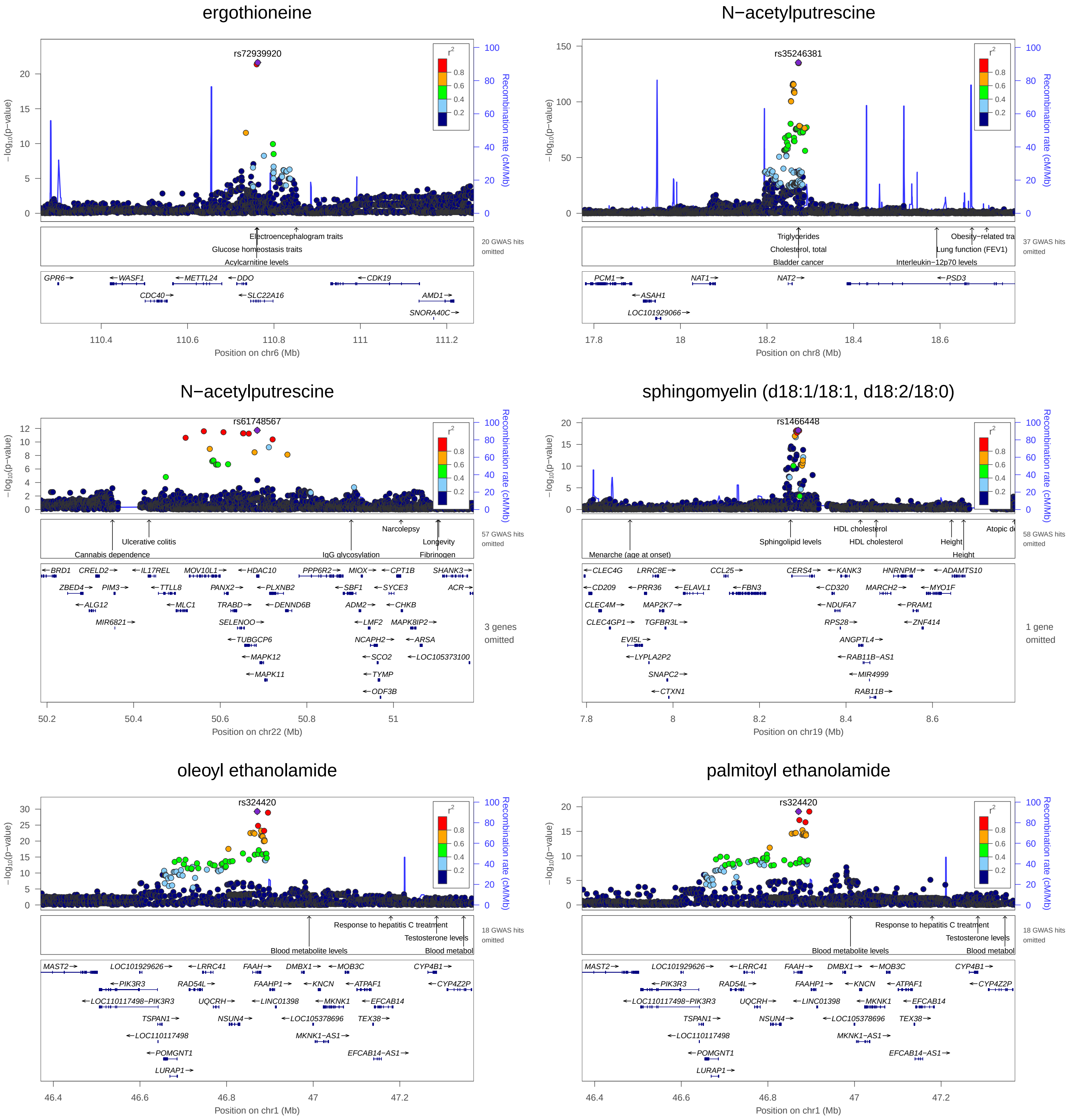

ergothioneine
rs72939920
N−acetylputrescine
rs35246381
N−acetylputrescine
rs61748567
sphingomyelin (d18:1/18:1, d18:2/18:0)
rs1466448
oleoyl ethanolamide
rs324420
palmitoyl ethanolamide
rs324420
−log10(p−value)
Recombination rate (cM/Mb)
r2
0.8
0.6
0.4
0.2
Electroencephalogram traits
Glucose homeostasis traits
Acylcarnitine levels
20 GWAS hits omitted
GPR6 WASF1 METTL24 DDO CDK19 CDC40 SLC22A16 AMD1 SNORA40C
Position on chr6 (Mb)
Triglycerides
Cholesterol, total
Bladder cancer
Obesity−related tra
Lung function (FEV1)
Interleukin−12p70 levels
37 GWAS hits omitted
PCM1 NAT1 NAT2 PSD3 ASAH1 LOC101929066
Position on chr8 (Mb)
Narcolepsy
Ulcerative colitis
Longevity
Cannabis dependence
IgG glycosylation
Fibrinogen
57 GWAS hits omitted
BRD1 CRELD2 IL17REL MOV10L1 HDAC10 PPP6R2 MIOX CPT1B SHANK3 ZBED4 PIM3 TTLL8 PANX2 PLXNB2 SBF1 SYCE3 ACR ALG12 MLC1 TRABD DENND6B ADM2 CHKB MIR6821 SELENOO LMF2 MAPK8IP2 TUBGCP6 NCAPH2 ARSA MAPK12 SCO2 LOC105373100 MAPK11 TYMP ODF3B
3 genes omitted
Position on chr22 (Mb)
HDL cholesterol
Atopic d
Sphingolipid levels
HDL cholesterol
Height
Menarche (age at onset)
Height
58 GWAS hits omitted
CLEC4G LRRC8E CCL25 CERS4 KANK3 HNRNPM ADAMTS10 CD209 PRR36 ELAVL1 FBN3 CD320 MARCH2 MYO1F CLEC4M MAP2K7 NDUFA7 PRAM1 CLEC4GP1 TGFBR3L RPS28 ZNF414 EVI5L ANGPTL4 LYPLA2P2 RAB11B−AS1 SNAPC2 MIR4999 CTXN1 RAB11B
1 gene omitted
Position on chr19 (Mb)
Response to hepatitis C treatment
Testosterone levels
Blood metabolite levels
Blood metabol
18 GWAS hits omitted
MAST2 LOC101929626 LRRC41 FAAH DMBX1 MOB3C CYP4B1 PIK3R3 RAD54L FAAHP1 KNCN ATPAF1 CYP4Z2P LOC110117498−PIK3R3 UQCRH LINC01398 MKNK1 EFCAB14 TSPAN1 NSUN4 LOC105378696 TEX38 LOC110117498 MKNK1−AS1 POMGNT1 EFCAB14−AS1 LURAP1
Position on chr1 (Mb)

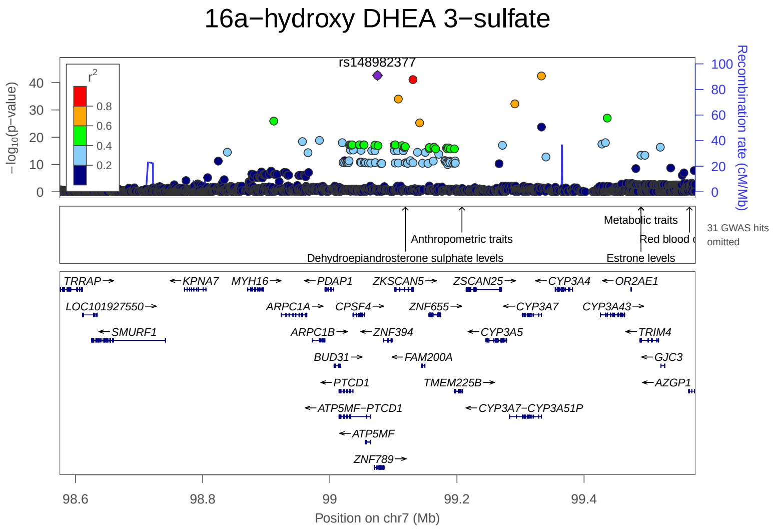
16a−hydroxy DHEA 3−sulfate
rs148982377
−log10(p−value)
Recombination rate (cM/Mb)
Anthropometric traits
Metabolic traits
Red blood c
Dehydroepiandrosterone sulphate levels
Estrone levels
31 GWAS hits omitted
TRRAP KPNA7 MYH16 PDAP1 ZKSCAN5 ZSCAN25 CYP3A4 OR2AE1
LOC101927550 ARPC1A CPSF4 ZNF655 CYP3A7 CYP3A43
SMURF1 ARPC1B ZNF394 CYP3A5 TRIM4
BUD31 FAM200A GJC3
PTCD1 TMEM225B AZGP1
ATP5MF−PTCD1 CYP3A7−CYP3A51P
ATP5MF
ZNF789
Position on chr7 (Mb)

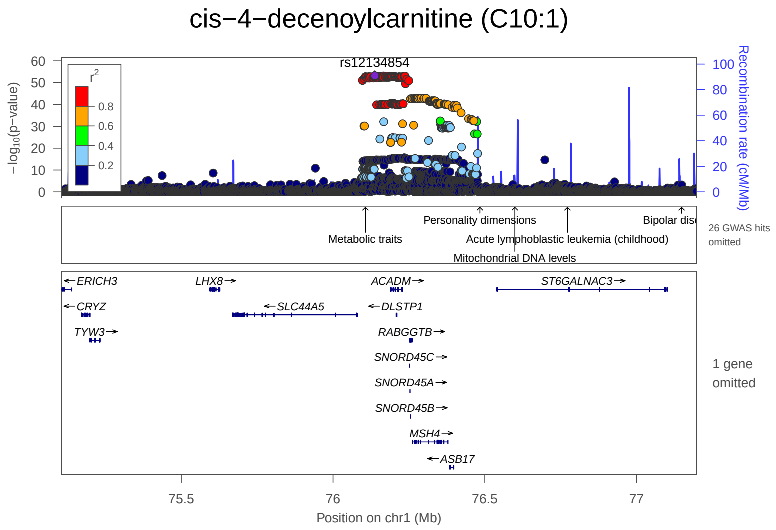
cis−4−decenoylcarnitine (C10:1)
rs12134854
−log10(p−value)
Recombination rate (cM/Mb)
Personality dimensions
Bipolar dis
Metabolic traits
Acute lymphoblastic leukemia (childhood)
Mitochondrial DNA levels
26 GWAS hits omitted
ERICH3 LHX8 ACADM ST6GALNAC3
CRYZ SLC44A5 DLSTP1
TYW3 RABGGTB
SNORD45C
SNORD45A
SNORD45B
MSH4
ASB17
1 gene omitted
Position on chr1 (Mb)

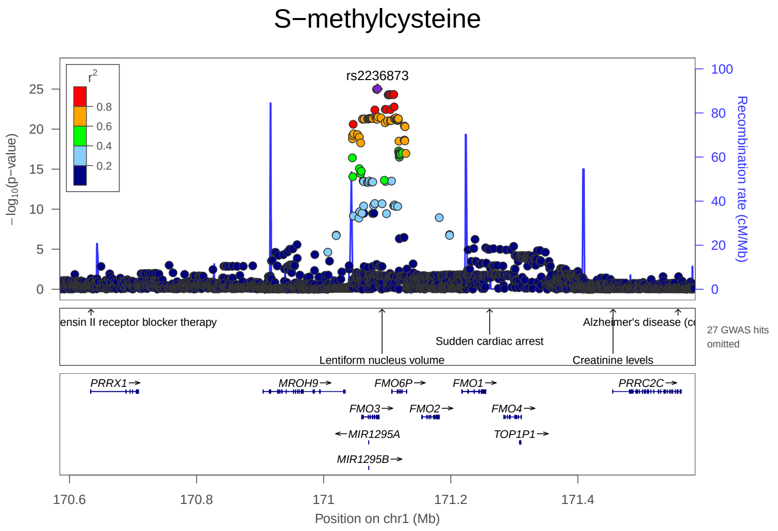
S−methylcysteine
rs2236873
−log10(p−value)
Recombination rate (cM/Mb)
ensin II receptor blocker therapy
Alzheimer's disease (c
Sudden cardiac arrest
Lentiform nucleus volume
Creatinine levels
27 GWAS hits omitted
PRRX1 MROH9 FMO6P FMO1 PRRC2C
FMO3 FMO2 FMO4
MIR1295A TOP1P1
MIR1295B
Position on chr1 (Mb)

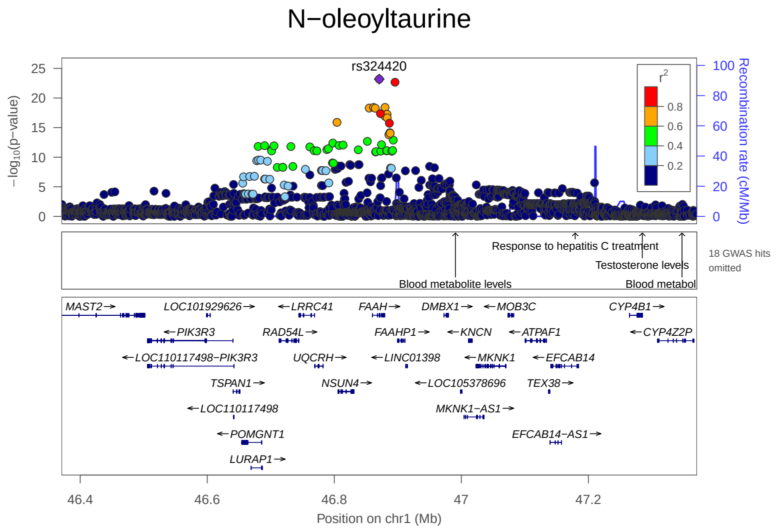
N−oleoyltaurine
rs324420
−log10(p−value)
Recombination rate (cM/Mb)
Response to hepatitis C treatment
Testosterone levels
Blood metabolite levels
Blood metabol
18 GWAS hits omitted
MAST2 LOC101929626 LRRC41 FAAH DMBX1 MOB3C CYP4B1
PIK3R3 RAD54L FAAHP1 KNCN ATPAF1 CYP4Z2P
LOC110117498−PIK3R3 UQCRH LINC01398 MKNK1 EFCAB14
TSPAN1 NSUN4 LOC105378696 TEX38
LOC110117498 MKNK1−AS1
POMGNT1 EFCAB14−AS1
LURAP1
Position on chr1 (Mb)

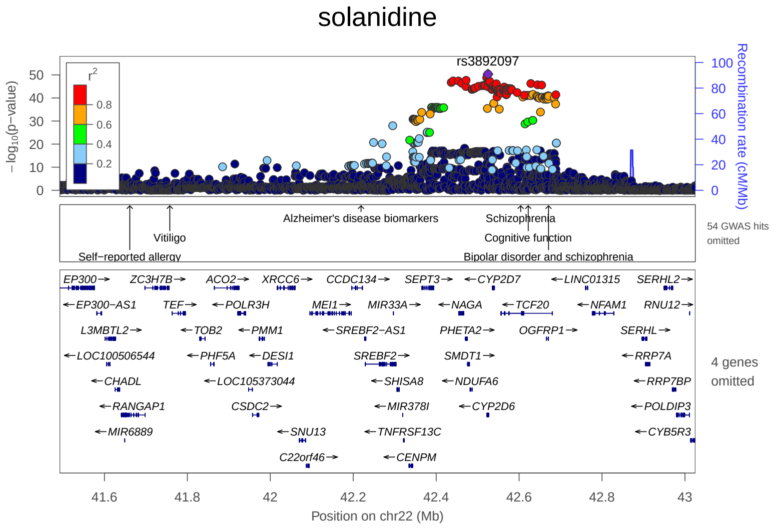
solanidine
rs3892097
−log10(p−value)
Recombination rate (cM/Mb)
Alzheimer's disease biomarkers
Schizophrenia
Vitiligo
Cognitive function
Self−reported allergy
Bipolar disorder and schizophrenia
54 GWAS hits omitted
EP300 ZC3H7B ACO2 XRCC6 CCDC134 SEPT3 CYP2D7 LINC01315 SERHL2
EP300−AS1 TEF POLR3H MEI1 MIR33A NAGA TCF20 NFAM1 RNU12
L3MBTL2 TOB2 PMM1 SREBF2−AS1 PHETA2 OGFRP1 SERHL
LOC100506544 PHF5A DESI1 SREBF2 SMDT1 RRP7A
CHADL LOC105373044 SHISA8 NDUFA6 RRP7BP
RANGAP1 CSDC2 MIR378I CYP2D6 POLDIP3
MIR6889 SNU13 TNFRSF13C CYB5R3
C22orf46 CENPM
4 genes omitted
Position on chr22 (Mb)

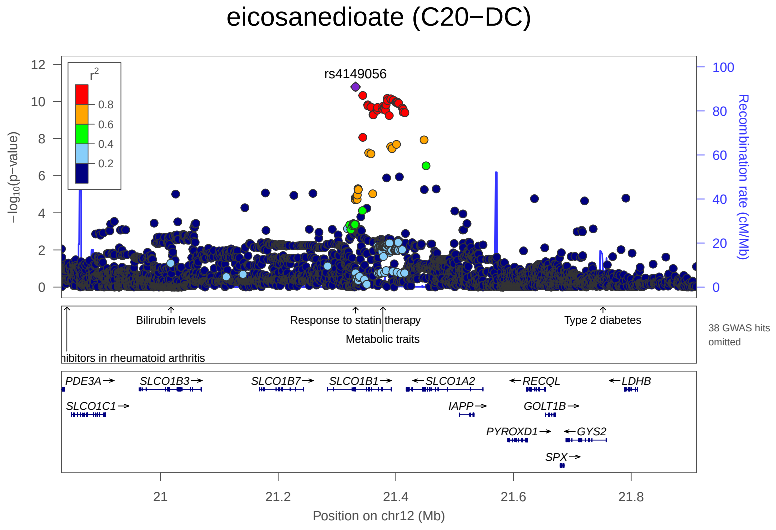
eicosanedioate (C20−DC)
rs4149056
−log10(p−value)
Recombination rate (cM/Mb)
Bilirubin levels
Response to statin therapy
Type 2 diabetes
Metabolic traits
hibitors in rheumatoid arthritis
38 GWAS hits omitted
PDE3A SLCO1B3 SLCO1B7 SLCO1B1 SLCO1A2 RECQL LDHB
SLCO1C1 IAPP GOLT1B
PYROXD1 GYS2
SPX
Position on chr12 (Mb)

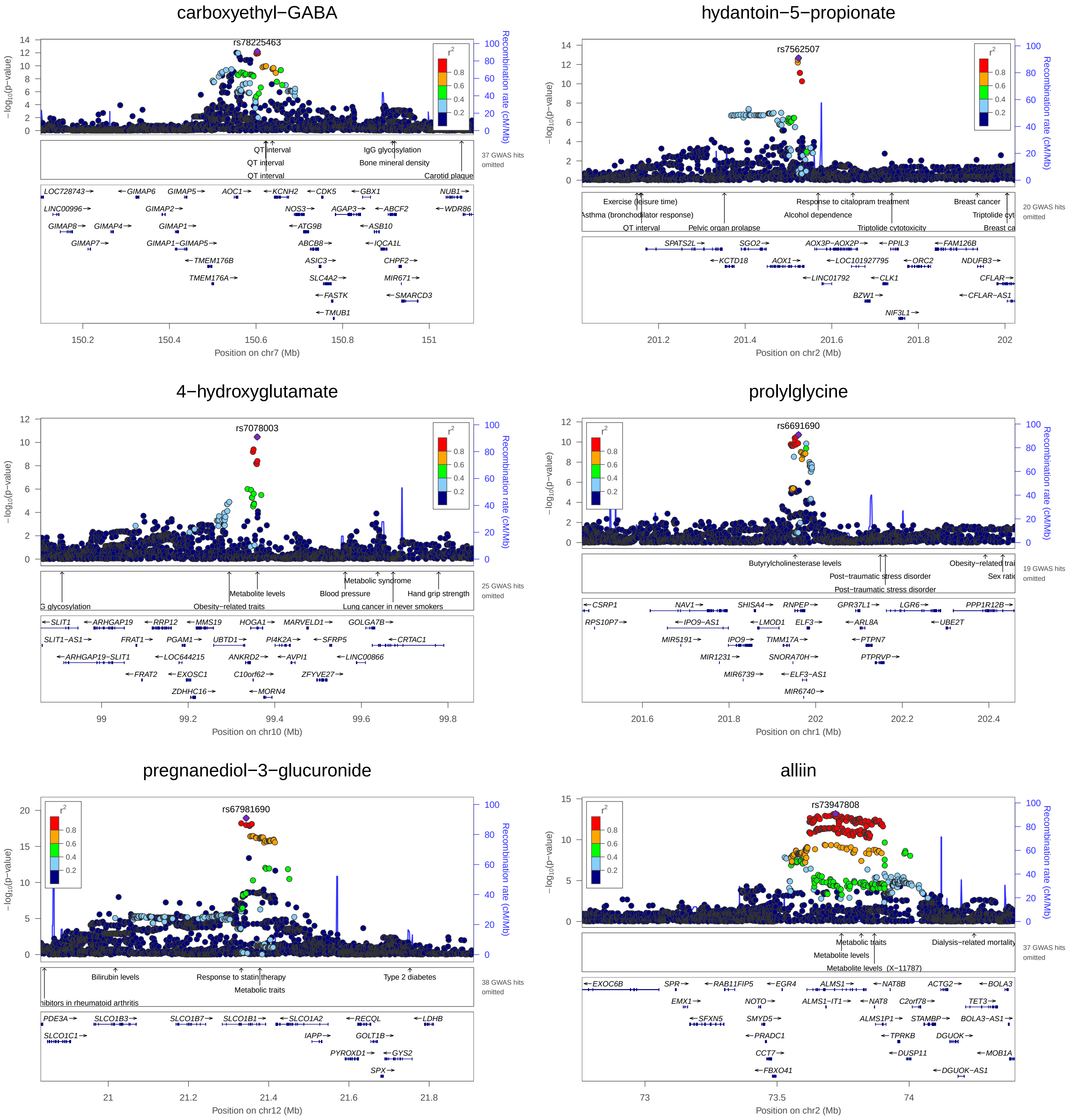
carboxyethyl−GABA
rs78225463
−log10(p−value)
Recombination rate (cM/Mb)
r2
QT interval
IgG glycosylation
Bone mineral density
Carotid plaque
37 GWAS hits omitted
LOC728743 GIMAP6 GIMAP5 AOC1 KCNH2 CDK5 GBX1 NUB1
LINC00996 GIMAP2 NOS3 AGAP3 ABCF2 WDR86
GIMAP8 GIMAP4 GIMAP1 ATG9B ASB10
GIMAP7 GIMAP1−GIMAP5 ABCB8 IQCA1L
TMEM176B ASIC3 CHPF2
TMEM176A SLC4A2 MIR671
FASTK SMARCD3
TMUB1
Position on chr7 (Mb)
hydantoin−5−propionate
rs7562507
Exercise (leisure time)
Asthma (bronchodilator response)
QT interval
Pelvic organ prolapse
Response to citalopram treatment
Alcohol dependence
Triptolide cytotoxicity
Breast cancer
20 GWAS hits omitted
SPATS2L SGO2 AOX3P−AOX2P PPIL3 FAM126B
KCTD18 AOX1 LOC101927795 ORC2 NDUFB3
LINC01792 CLK1 CFLAR
BZW1 CFLAR−AS1
NIF3L1
Position on chr2 (Mb)
4−hydroxyglutamate
rs7078003
Metabolic syndrome
Metabolite levels
Blood pressure
Hand grip strength
Obesity−related traits
Lung cancer in never smokers
25 GWAS hits omitted
SLIT1 ARHGAP19 RRP12 MMS19 HOGA1 MARVELD1 GOLGA7B
SLIT1−AS1 FRAT1 PGAM1 UBTD1 PI4K2A SFRP5 CRTAC1
ARHGAP19−SLIT1 LOC644215 ANKRD2 AVPI1 LINC00866
FRAT2 EXOSC1 C10orf62 ZFYVE27
ZDHHC16 MORN4
Position on chr10 (Mb)
prolylglycine
rs6691690
Butyrylcholinesterase levels
Post−traumatic stress disorder
Obesity−related traits
19 GWAS hits omitted
CSRP1 NAV1 SHISA4 RNPEP GPR37L1 LGR6 PPP1R12B
RPS10P7 IPO9−AS1 LMOD1 ELF3 ARL8A UBE2T
MIR5191 IPO9 TIMM17A PTPN7
MIR1231 SNORA70H PTPRVP
MIR6739 ELF3−AS1
MIR6740
Position on chr1 (Mb)
pregnanediol−3−glucuronide
rs67981690
Bilirubin levels
Response to statin therapy
Metabolic traits
Type 2 diabetes
38 GWAS hits omitted
PDE3A SLCO1B3 SLCO1B7 SLCO1B1 SLCO1A2 RECQL LDHB
SLCO1C1 IAPP GOLT1B
PYROXD1 GYS2
SPX
Position on chr12 (Mb)
alliin
rs73947808
Metabolic traits
Dialysis−related mortality
Metabolite levels (X−11787)
37 GWAS hits omitted
EXOC6B SPR RAB11FIP5 EGR4 ALMS1 NAT8B ACTG2 BOLA3
EMX1 NOTO ALMS1−IT1 NAT8 C2orf78 TET3
SFXN5 SMYD5 ALMS1P1 STAMBP BOLA3−AS1
PRADC1 TPRKB DGUOK
CCT7 DUSP11 MOB1A
FBXO41 DGUOK−AS1
Position on chr2 (Mb)

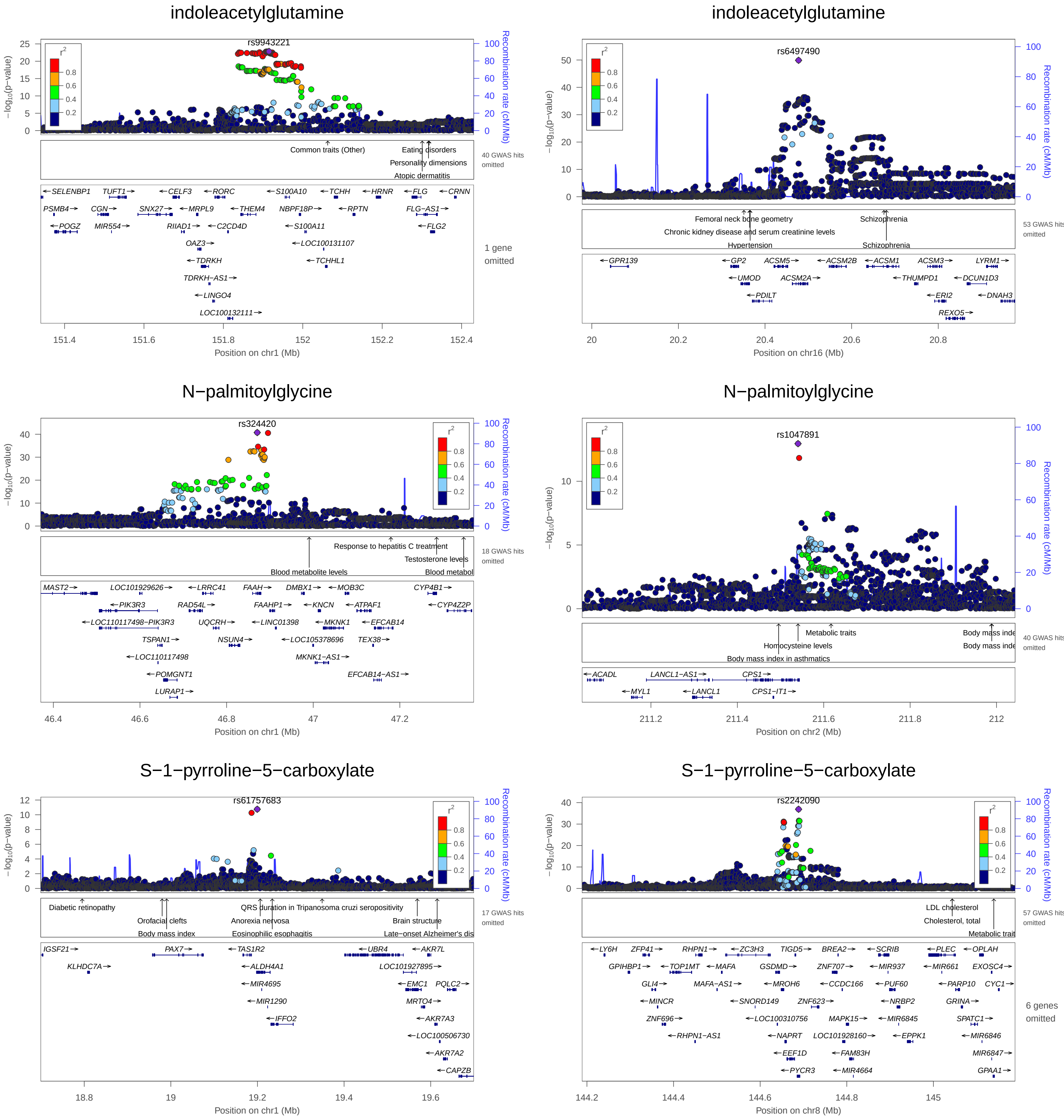
indoleacetylglutamine
rs9943221
−log10(p−value)
Recombination rate (cM/Mb)
Common traits (Other)
Eating disorders
Personality dimensions
Atopic dermatitis
40 GWAS hits omitted
1 gene omitted
Position on chr1 (Mb)
indoleacetylglutamine
rs6497490
Femoral neck bone geometry
Schizophrenia
Chronic kidney disease and serum creatinine levels
Hypertension
53 GWAS hits omitted
Position on chr16 (Mb)
N−palmitoylglycine
rs324420
Response to hepatitis C treatment
Testosterone levels
Blood metabolite levels
18 GWAS hits omitted
Position on chr1 (Mb)
N−palmitoylglycine
rs1047891
Metabolic traits
Homocysteine levels
Body mass index in asthmatics
40 GWAS hits omitted
Position on chr2 (Mb)
S−1−pyrroline−5−carboxylate
rs61757683
Diabetic retinopathy
Orofacial clefts
Body mass index
QRS duration in Tripanosoma cruzi seropositivity
Anorexia nervosa
Eosinophilic esophagitis
Brain structure
17 GWAS hits omitted
Position on chr1 (Mb)
S−1−pyrroline−5−carboxylate
rs2242090
LDL cholesterol
Cholesterol, total
Metabolic trait
57 GWAS hits omitted
6 genes omitted
Position on chr8 (Mb)

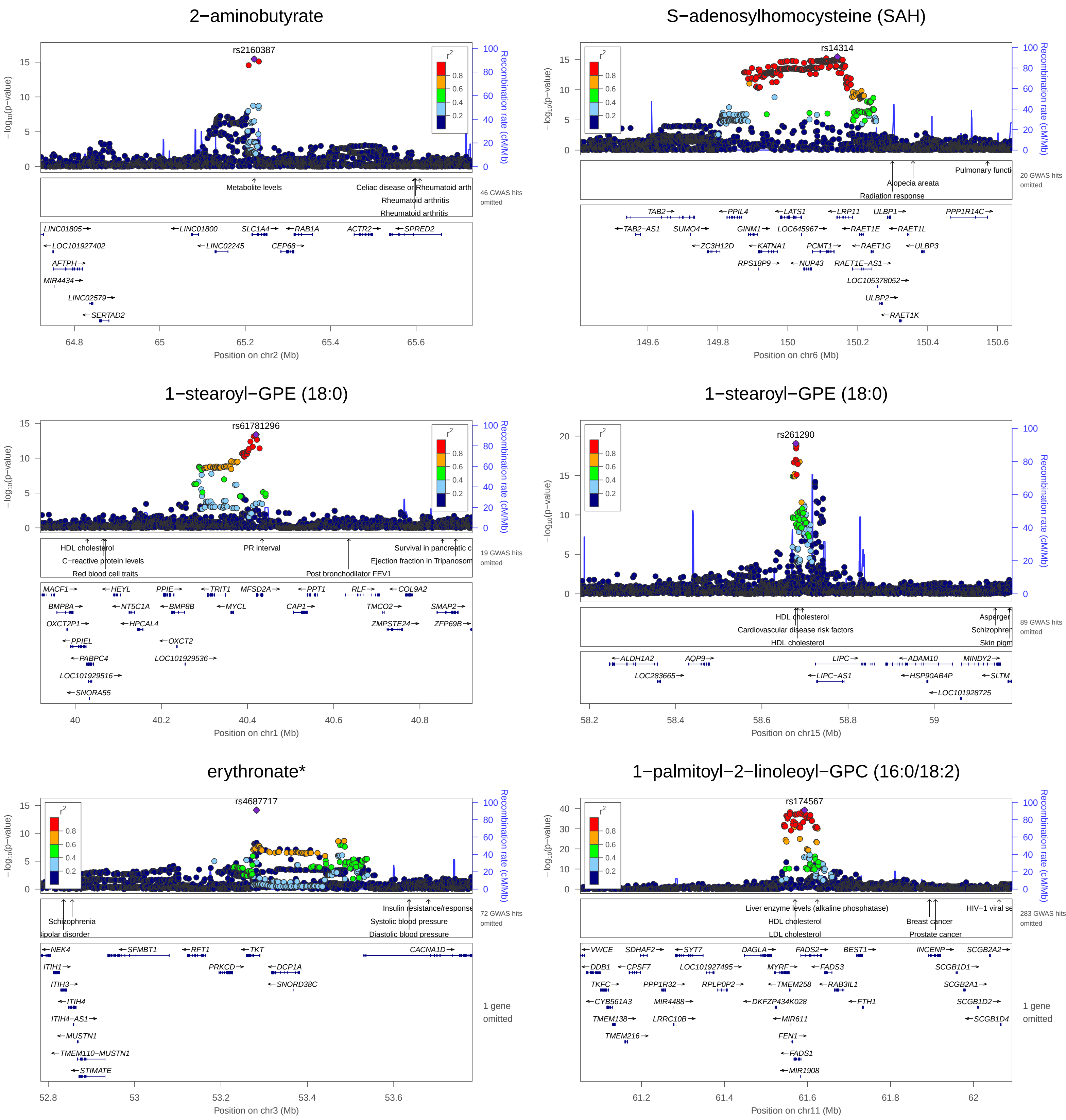

2−aminobutyrate
rs2160387
−log10(p−value)
Recombination rate (cM/Mb)
r2
Metabolite levels
Celiac disease or Rheumatoid arth
Rheumatoid arthritis
Rheumatoid arthritis
46 GWAS hits omitted
LINC01805
LOC101927402
AFTPH
MIR4434
LINC02579
SERTAD2
LINC01800
LINC02245
SLC1A4
CEP68
RAB1A
ACTR2
SPRED2
Position on chr2 (Mb)
S−adenosylhomocysteine (SAH)
rs14314
Pulmonary functi
Alopecia areata
Radiation response
20 GWAS hits omitted
TAB2
TAB2−AS1
SUMO4
ZC3H12D
PPIL4
GINM1
RPS18P9
LATS1
KATNA1
LOC645967
NUP43
LRP11
PCMT1
ULBP1
RAET1E
RAET1G
RAET1E−AS1
LOC105378052
ULBP2
RAET1K
RAET1L
ULBP3
PPP1R14C
Position on chr6 (Mb)
1−stearoyl−GPE (18:0)
rs61781296
HDL cholesterol
C−reactive protein levels
Red blood cell traits
PR interval
Survival in pancreatic c
Ejection fraction in Tripanosom
Post bronchodilator FEV1
19 GWAS hits omitted
MACF1
BMP8A
OXCT2P1
PPIEL
PABPC4
LOC101929516
SNORA55
HEYL
NT5C1A
HPCAL4
PPIE
BMP8B
OXCT2
LOC101929536
TRIT1
MYCL
MFSD2A
PPT1
CAP1
RLF
TMCO2
ZMPSTE24
COL9A2
SMAP2
ZFP69B
Position on chr1 (Mb)
1−stearoyl−GPE (18:0)
rs261290
HDL cholesterol
Cardiovascular disease risk factors
HDL cholesterol
Asperger
Schizophren
Skin pigm
89 GWAS hits omitted
ALDH1A2
LOC283665
AQP9
LIPC
LIPC−AS1
ADAM10
HSP90AB4P
LOC101928725
MINDY2
SLTM
Position on chr15 (Mb)
erythronate*
rs4687717
Schizophrenia
ipolar disorder
Insulin resistance/response
Systolic blood pressure
Diastolic blood pressure
72 GWAS hits omitted
NEK4
ITIH1
ITIH3
ITIH4
ITIH4−AS1
MUSTN1
TMEM110−MUSTN1
STIMATE
SFMBT1
RFT1
PRKCD
TKT
DCP1A
SNORD38C
CACNA1D
1 gene omitted
Position on chr3 (Mb)
1−palmitoyl−2−linoleoyl−GPC (16:0/18:2)
rs174567
Liver enzyme levels (alkaline phosphatase)
HDL cholesterol
LDL cholesterol
Breast cancer
Prostate cancer
HIV−1 viral se
283 GWAS hits omitted
VWCE
DDB1
TKFC
CYB561A3
TMEM138
TMEM216
SDHAF2
CPSF7
PPP1R32
MIR4488
LRRC10B
SYT7
LOC101927495
RPLP0P2
DAGLA
MYRF
TMEM258
DKFZP434K028
MIR611
FEN1
FADS1
MIR1908
FADS2
FADS3
RAB3IL1
FTH1
BEST1
INCENP
SCGB1D1
SCGB2A1
SCGB1D2
SCGB1D4
SCGB2A2
1 gene omitted
Position on chr11 (Mb)

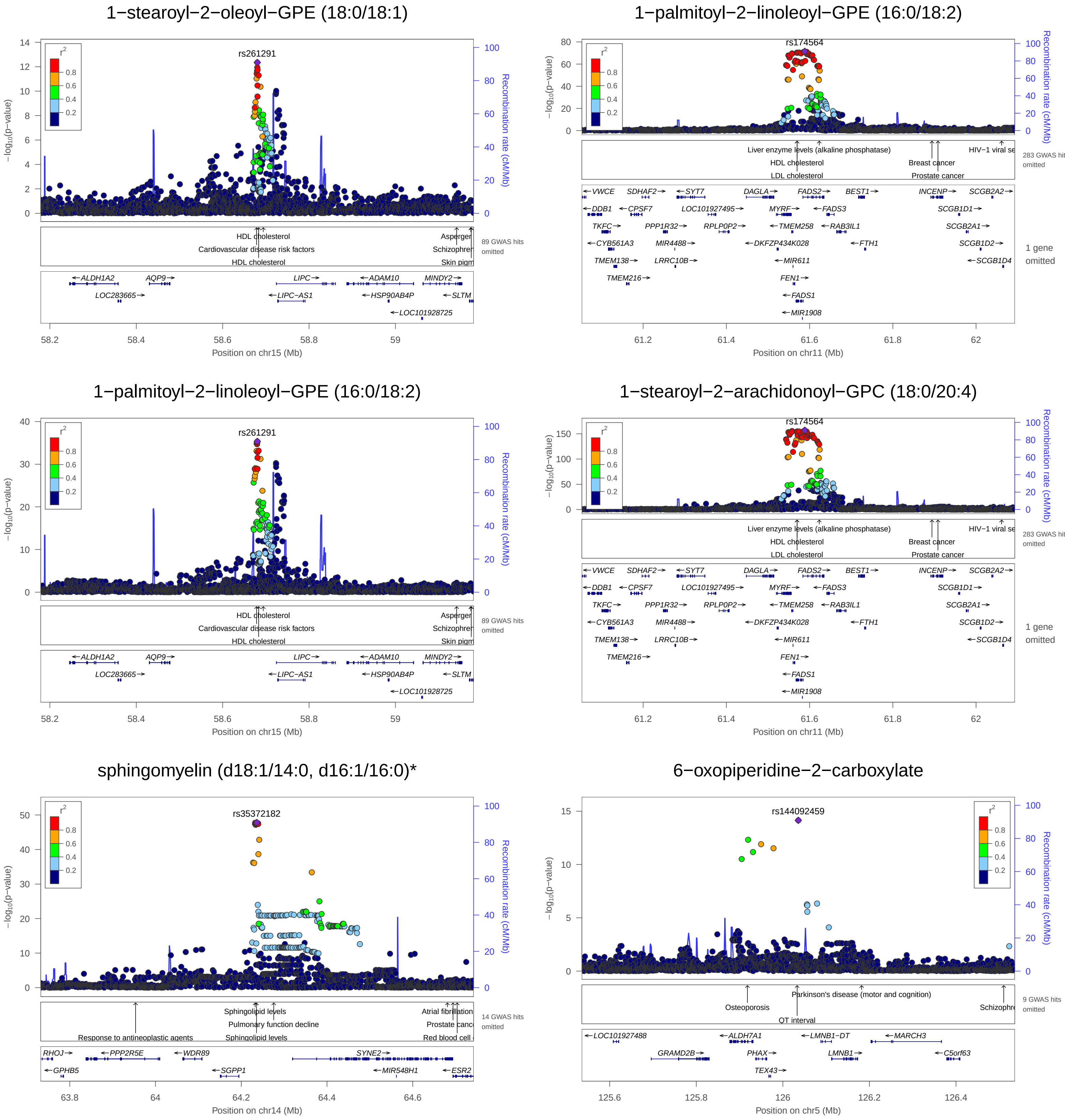

1−stearoyl−2−oleoyl−GPE (18:0/18:1)
rs261291
−log10(p−value)
Recombination rate (cM/Mb)
r2
HDL cholesterol
Cardiovascular disease risk factors
HDL cholesterol
Asperger
Schizophren
Skin pigm
89 GWAS hits omitted
ALDH1A2
AQP9
LIPC
ADAM10
MINDY2
LOC283665
LIPC−AS1
HSP90AB4P
SLTM
LOC101928725
Position on chr15 (Mb)
1−palmitoyl−2−linoleoyl−GPE (16:0/18:2)
rs174564
Liver enzyme levels (alkaline phosphatase)
HDL cholesterol
LDL cholesterol
Breast cancer
Prostate cancer
HIV−1 viral se
283 GWAS hits omitted
VWCE
SDHAF2
SYT7
DAGLA
FADS2
BEST1
INCENP
SCGB2A2
DDB1
CPSF7
LOC101927495
MYRF
FADS3
SCGB1D1
TKFC
PPP1R32
RPLP0P2
TMEM258
RAB3IL1
SCGB2A1
CYB561A3
MIR4488
DKFZP434K028
FTH1
SCGB1D2
TMEM138
LRRC10B
MIR611
SCGB1D4
TMEM216
FEN1
FADS1
MIR1908
1 gene omitted
Position on chr11 (Mb)
1−palmitoyl−2−linoleoyl−GPE (16:0/18:2)
rs261291
1−stearoyl−2−arachidonoyl−GPC (18:0/20:4)
rs174564
sphingomyelin (d18:1/14:0, d16:1/16:0)*
rs35372182
Response to antineoplastic agents
Sphingolipid levels
Pulmonary function decline
Sphingolipid levels
Atrial fibrillation
Prostate canc
Red blood cell
14 GWAS hits omitted
RHOJ
PPP2R5E
WDR89
SYNE2
GPHB5
SGPP1
MIR548H1
ESR2
Position on chr14 (Mb)
6−oxopiperidine−2−carboxylate
rs144092459
Osteoporosis
Parkinson's disease (motor and cognition)
QT interval
Schizophre
9 GWAS hits omitted
LOC101927488
ALDH7A1
LMNB1−DT
MARCH3
GRAMD2B
PHAX
LMNB1
C5orf63
TEX43
Position on chr5 (Mb)

6−oxopiperidine−2−carboxylate

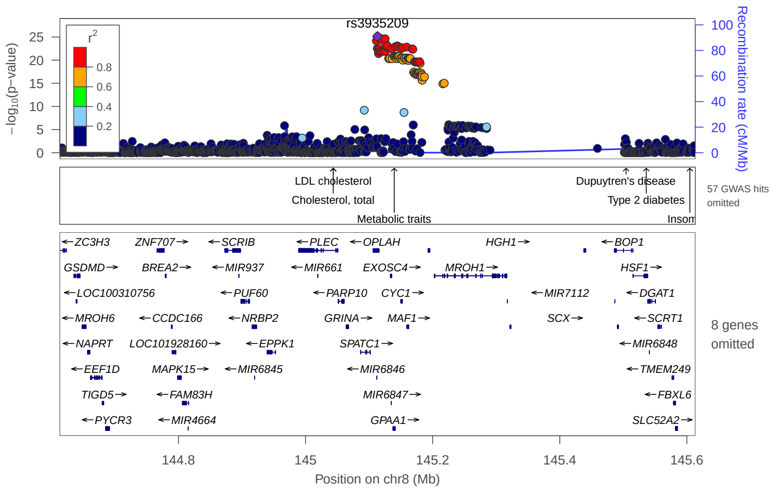

N−delta−acetylornithine

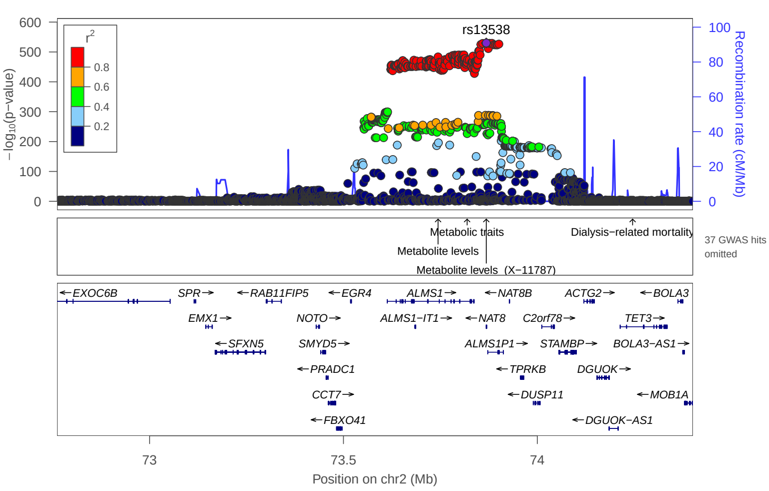

N−acetyl−1−methylhistidine*

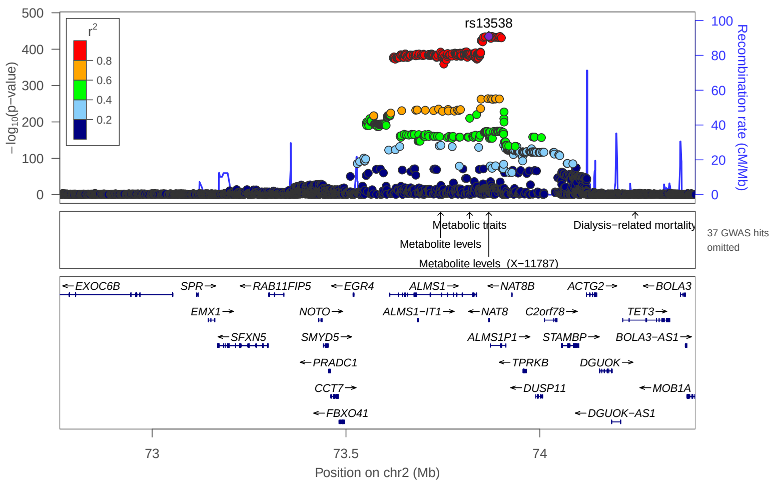

N−acetyl−3−methylhistidine*

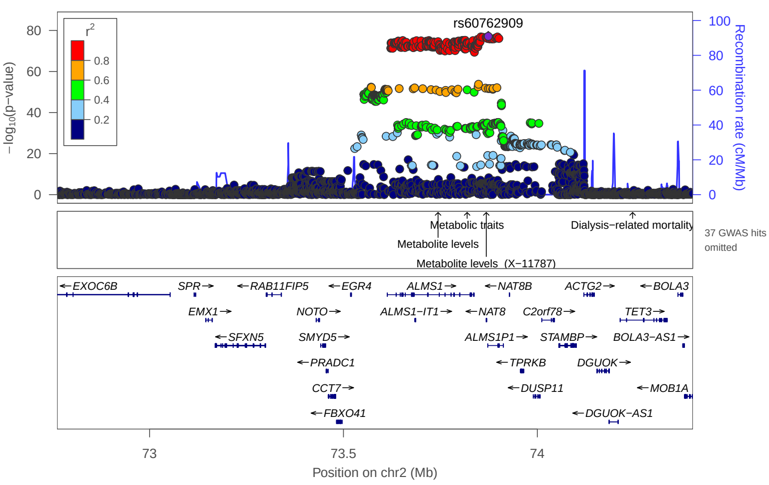

acisoga

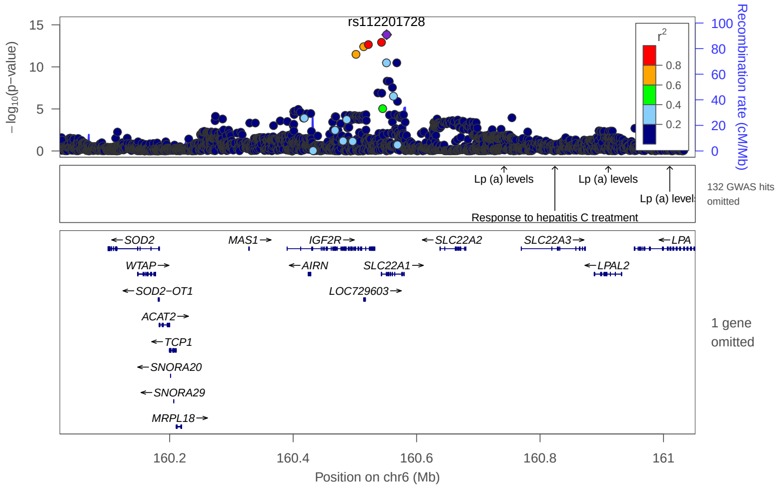

acisoga

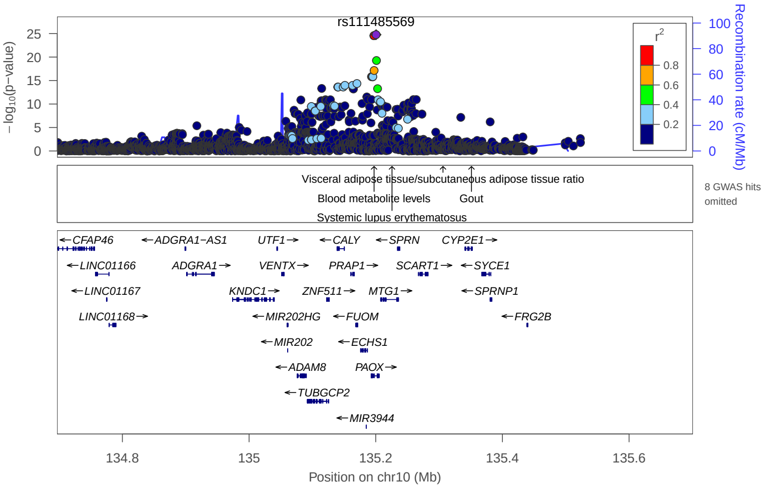

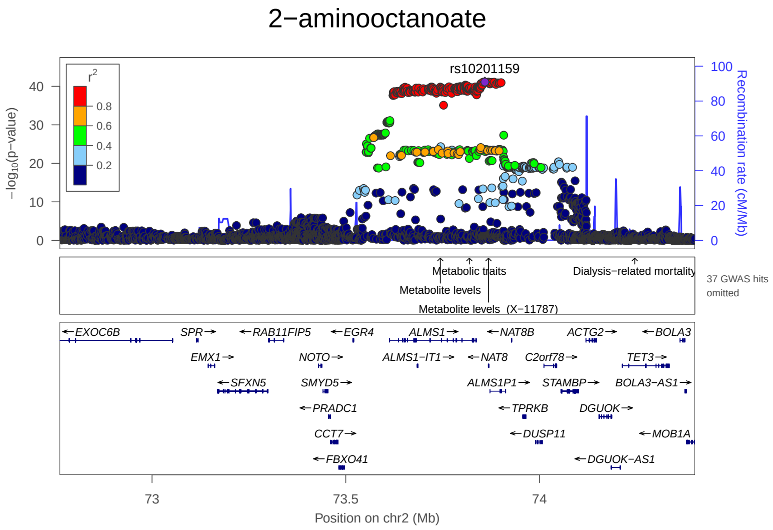
2−aminooctanoate
rs10201159
−log10(p−value)
Recombination rate (cM/Mb)
Metabolic traits
Metabolite levels
Metabolite levels (X−11787)
Dialysis−related mortality
37 GWAS hits omitted
EXOC6B SPR RAB11FIP5 EGR4 ALMS1 NAT8B ACTG2 BOLA3
EMX1 NOTO ALMS1−IT1 NAT8 C2orf78 TET3
SFXN5 SMYD5 ALMS1P1 STAMBP BOLA3−AS1
PRADC1 TPRKB DGUOK
CCT7 DUSP11 MOB1A
FBXO41 DGUOK−AS1
Position on chr2 (Mb)
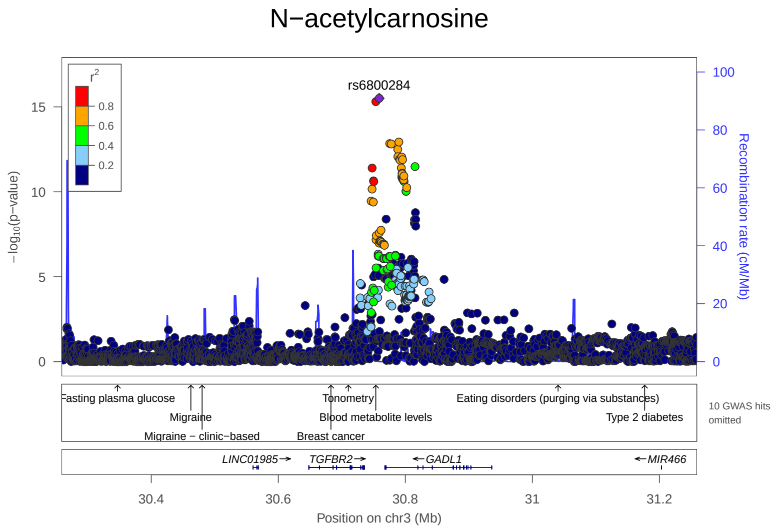
N−acetylcarnosine
rs6800284
−log10(p−value)
Recombination rate (cM/Mb)
Fasting plasma glucose
Migraine
Migraine − clinic−based
Tonometry
Blood metabolite levels
Breast cancer
Eating disorders (purging via substances)
Type 2 diabetes
10 GWAS hits omitted
LINC01985 TGFBR2 GADL1 MIR466
Position on chr3 (Mb)
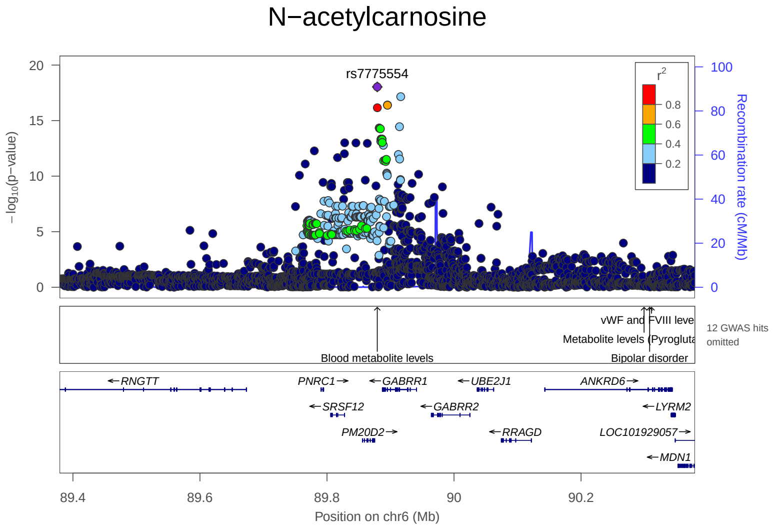
N−acetylcarnosine
rs7775554
−log10(p−value)
Recombination rate (cM/Mb)
Blood metabolite levels
vWF and FVIII leve
Metabolite levels (Pyrogluta
Bipolar disorder
12 GWAS hits omitted
RNGTT PNRC1 GABRR1 UBE2J1 ANKRD6
SRSF12 GABRR2 LYRM2
PM20D2 RRAGD LOC101929057
MDN1
Position on chr6 (Mb)
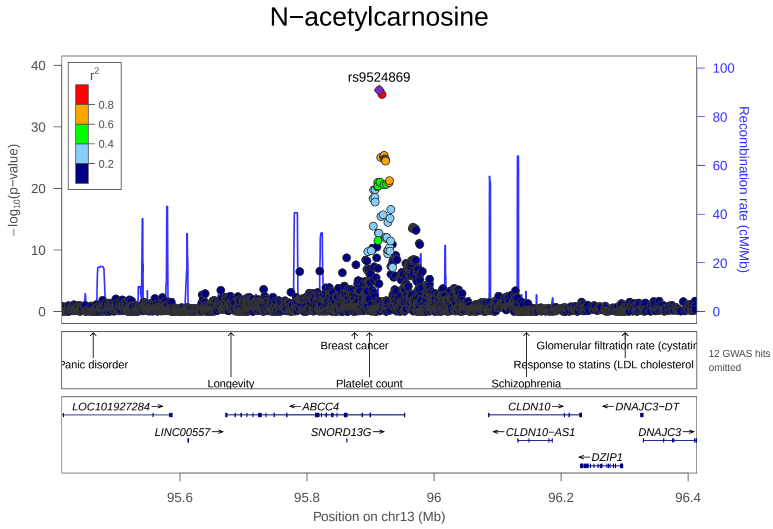
N−acetylcarnosine
rs9524869
−log10(p−value)
Recombination rate (cM/Mb)
Panic disorder
Longevity
Breast cancer
Platelet count
Schizophrenia
Response to statins (LDL cholesterol
Glomerular filtration rate (cystatin
12 GWAS hits omitted
LOC101927284 ABCC4 CLDN10 DNAJC3−DT
LINC00557 SNORD13G CLDN10−AS1 DNAJC3
DZIP1
Position on chr13 (Mb)
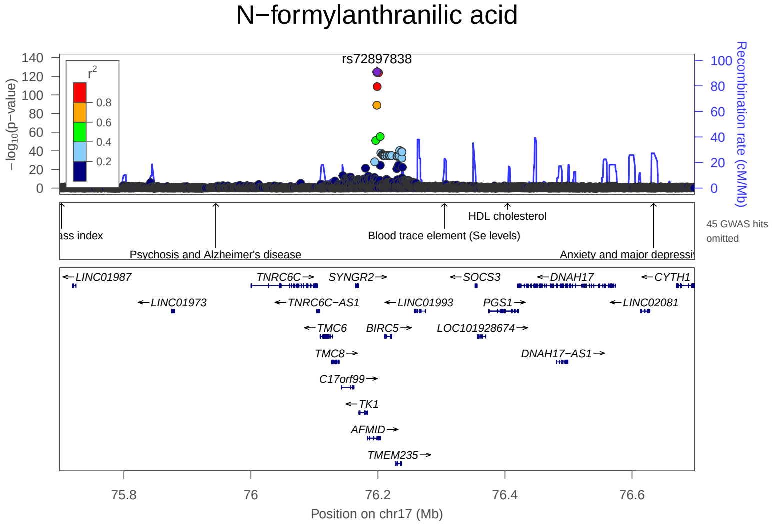
N−formylanthranilic acid
rs72897838
−log10(p−value)
Recombination rate (cM/Mb)
ass index
Psychosis and Alzheimer's disease
HDL cholesterol
Blood trace element (Se levels)
Anxiety and major depressi
45 GWAS hits omitted
LINC01987 TNRC6C SYNGR2 SOCS3 DNAH17 CYTH1
LINC01973 TNRC6C−AS1 LINC01993 PGS1 LINC02081
TMC6 BIRC5 LOC101928674
TMC8 DNAH17−AS1
C17orf99
TK1
AFMID
TMEM235
Position on chr17 (Mb)
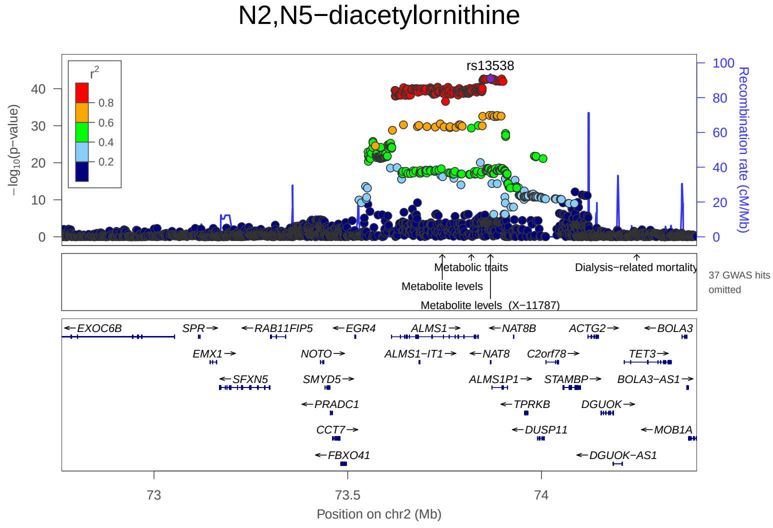
N2,N5−diacetylornithine
rs13538
−log10(p−value)
Recombination rate (cM/Mb)
Metabolic traits
Metabolite levels
Metabolite levels (X−11787)
Dialysis−related mortality
37 GWAS hits omitted
EXOC6B SPR RAB11FIP5 EGR4 ALMS1 NAT8B ACTG2 BOLA3
EMX1 NOTO ALMS1−IT1 NAT8 C2orf78 TET3
SFXN5 SMYD5 ALMS1P1 STAMBP BOLA3−AS1
PRADC1 TPRKB DGUOK
CCT7 DUSP11 MOB1A
FBXO41 DGUOK−AS1
Position on chr2 (Mb)

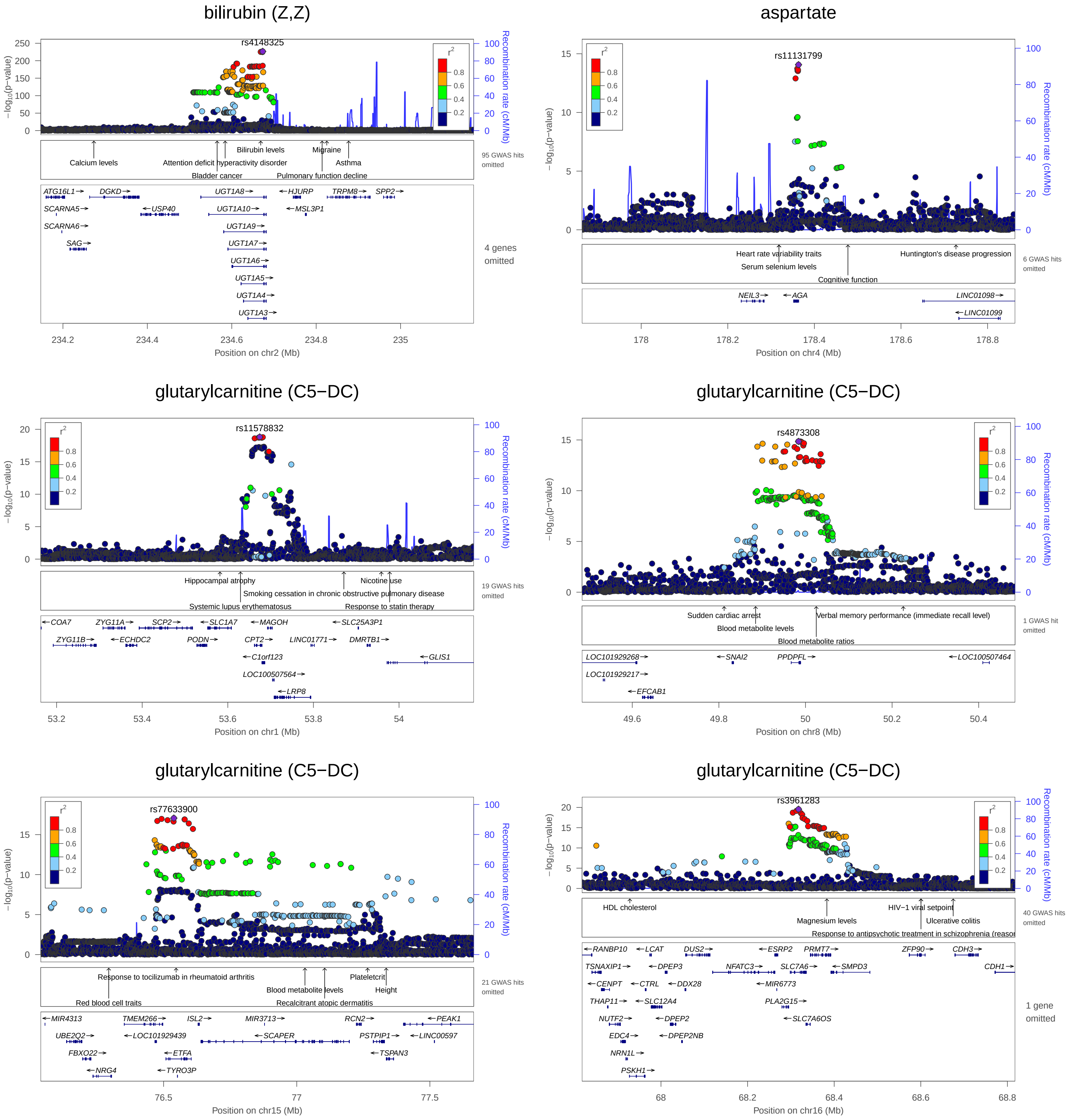

bilirubin (Z,Z)
rs4148325
−log10(p−value)
Recombination rate (cM/Mb)
Calcium levels
Attention deficit hyperactivity disorder
Bladder cancer
Bilirubin levels
Migraine
Asthma
Pulmonary function decline
95 GWAS hits omitted
ATG16L1
DGKD
SCARNA5
USP40
SCARNA6
SAG
UGT1A8
UGT1A10
UGT1A9
UGT1A7
UGT1A6
UGT1A5
UGT1A4
UGT1A3
HJURP
MSL3P1
TRPM8
SPP2
4 genes omitted
Position on chr2 (Mb)
aspartate
rs11131799
Heart rate variability traits
Serum selenium levels
Cognitive function
Huntington's disease progression
6 GWAS hits omitted
NEIL3
AGA
LINC01098
LINC01099
Position on chr4 (Mb)
glutarylcarnitine (C5−DC)
rs11578832
Hippocampal atrophy
Systemic lupus erythematosus
Smoking cessation in chronic obstructive pulmonary disease
Nicotine use
Response to statin therapy
19 GWAS hits omitted
COA7
ZYG11A
SCP2
SLC1A7
MAGOH
SLC25A3P1
ZYG11B
ECHDC2
PODN
CPT2
LINC01771
DMRTB1
C1orf123
GLIS1
LOC100507564
LRP8
Position on chr1 (Mb)
glutarylcarnitine (C5−DC)
rs4873308
Sudden cardiac arrest
Blood metabolite levels
Verbal memory performance (immediate recall level)
Blood metabolite ratios
1 GWAS hit omitted
LOC101929268
LOC101929217
EFCAB1
SNAI2
PPDPFL
LOC100507464
Position on chr8 (Mb)
glutarylcarnitine (C5−DC)
rs77633900
Response to tocilizumab in rheumatoid arthritis
Red blood cell traits
Blood metabolite levels
Recalcitrant atopic dermatitis
Plateletcrit
Height
21 GWAS hits omitted
MIR4313
TMEM266
ISL2
MIR3713
RCN2
PEAK1
UBE2Q2
LOC101929439
SCAPER
PSTPIP1
LINC00597
FBXO22
ETFA
TSPAN3
NRG4
TYRO3P
Position on chr15 (Mb)
glutarylcarnitine (C5−DC)
rs3961283
HDL cholesterol
Magnesium levels
HIV−1 viral setpoint
Ulcerative colitis
Response to antipsychotic treatment in schizophrenia (reasor
40 GWAS hits omitted
RANBP10
LCAT
DUS2
ESRP2
PRMT7
ZFP90
CDH3
TSNAXIP1
DPEP3
NFATC3
SLC7A6
SMPD3
CDH1
CENPT
CTRL
DDX28
MIR6773
THAP11
SLC12A4
PLA2G15
NUTF2
DPEP2
SLC7A6OS
EDC4
DPEP2NB
NRN1L
PSKH1
1 gene omitted
Position on chr16 (Mb)

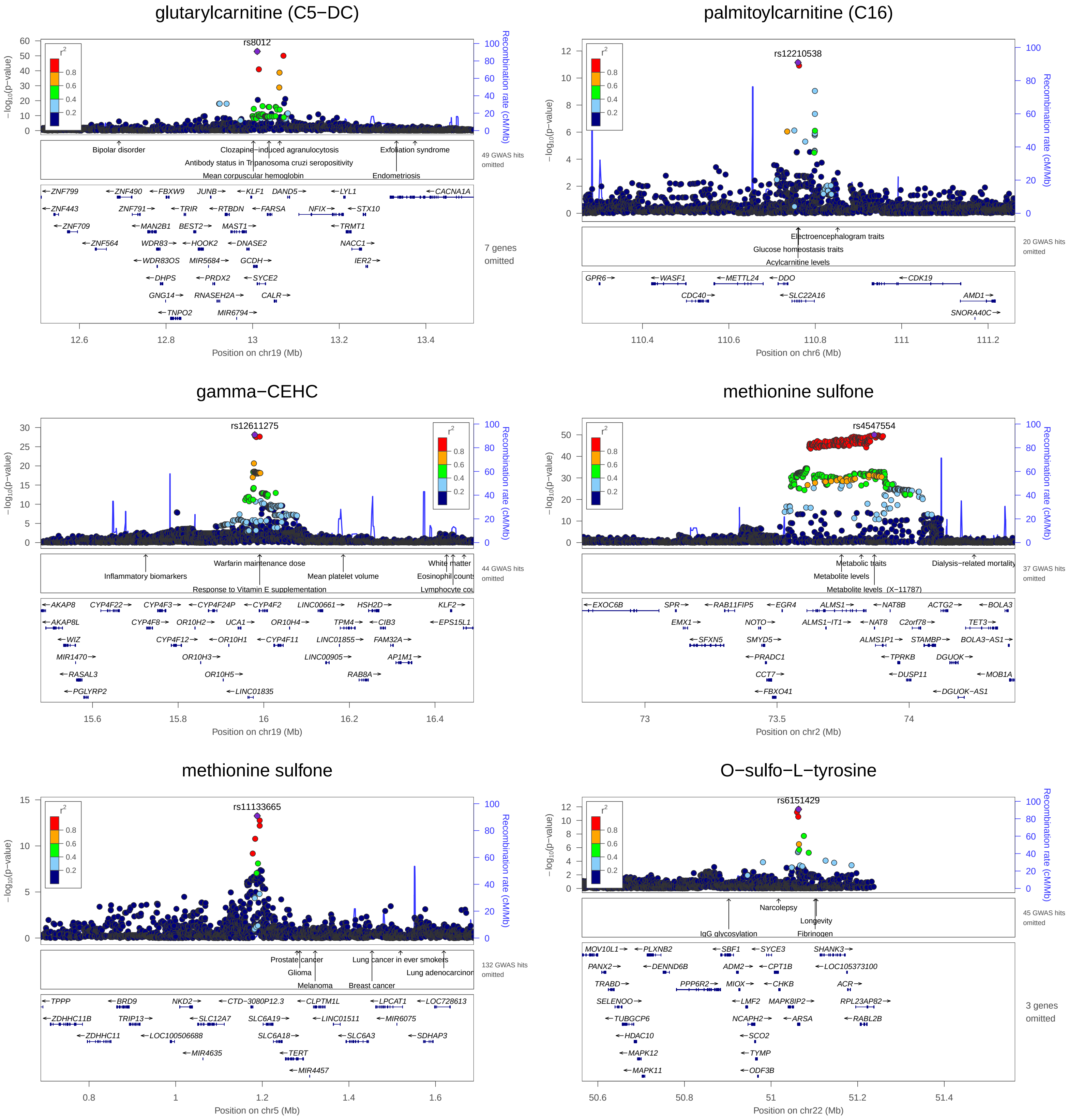

glutarylcarnitine (C5−DC)
rs8012
−log10(p−value)
Recombination rate (cM/Mb)
Bipolar disorder
Clozapine−induced agranulocytosis
Antibody status in Trypanosoma cruzi seropositivity
Mean corpuscular hemoglobin
Exfoliation syndrome
Endometriosis
49 GWAS hits omitted
ZNF799 ZNF490 FBXW9 JUNB KLF1 DAND5 LYL1 CACNA1A
ZNF443 ZNF791 TRIR RTBDN FARSA NFIX STX10
ZNF709 MAN2B1 BEST2 MAST1 TRMT1
ZNF564 WDR83 HOOK2 DNASE2 NACC1
WDR83OS MIR5684 GCDH IER2
DHPS PRDX2 SYCE2
GNG14 RNASEH2A CALR
TNPO2 MIR6794
7 genes omitted
Position on chr19 (Mb)
palmitoylcarnitine (C16)
rs12210538
Electroencephalogram traits
Glucose homeostasis traits
Acylcarnitine levels
20 GWAS hits omitted
GPR6 WASF1 METTL24 DDO CDK19
CDC40 SLC22A16 AMD1
SNORA40C
Position on chr6 (Mb)
gamma−CEHC
rs12611275
Warfarin maintenance dose
Inflammatory biomarkers
Mean platelet volume
White matter
Eosinophil counts
Response to Vitamin E supplementation
Lymphocyte cou
44 GWAS hits omitted
AKAP8 CYP4F22 CYP4F3 CYP4F24P CYP4F2 LINC00661 HSH2D KLF2
AKAP8L CYP4F8 OR10H2 UCA1 OR10H4 TPM4 CIB3 EPS15L1
WIZ CYP4F12 OR10H1 CYP4F11 LINC01855 FAM32A
MIR1470 OR10H3 LINC00905 AP1M1
RASAL3 OR10H5 RAB8A
PGLYRP2 LINC01835
Position on chr19 (Mb)
methionine sulfone
rs4547554
Metabolic traits
Metabolite levels
Metabolite levels (X−11787)
Dialysis−related mortality
37 GWAS hits omitted
EXOC6B SPR RAB11FIP5 EGR4 ALMS1 NAT8B ACTG2 BOLA3
EMX1 NOTO ALMS1−IT1 NAT8 C2orf78 TET3
SFXN5 SMYD5 ALMS1P1 STAMBP BOLA3−AS1
PRADC1 TPRKB DGUOK
CCT7 DUSP11 MOB1A
FBXO41 DGUOK−AS1
Position on chr2 (Mb)
methionine sulfone
rs11133665
Prostate cancer
Glioma
Melanoma
Lung cancer in ever smokers
Breast cancer
Lung adenocarcinoma
132 GWAS hits omitted
TPPP BRD9 NKD2 CTD−3080P12.3 CLPTM1L LPCAT1 LOC728613
ZDHHC11B TRIP13 SLC12A7 SLC6A19 LINC01511 MIR6075
ZDHHC11 LOC100506688 SLC6A18 SLC6A3 SDHAP3
MIR4635 TERT
MIR4457
Position on chr5 (Mb)
O−sulfo−L−tyrosine
rs6151429
Narcolepsy
Longevity
IgG glycosylation
Fibrinogen
45 GWAS hits omitted
MOV10L1 PLXNB2 SBF1 SYCE3 SHANK3
PANX2 DENND6B ADM2 CPT1B LOC105373160
TRABD PPP6R2 MIOX CHKB ACR
SELENOO LMF2 MAPK8IP2 RPL23AP82
TUBGCP6 NCAPH2 ARSA RABL2B
HDAC10 SCO2
MAPK12 TYMP
MAPK11 ODF3B
3 genes omitted
Position on chr22 (Mb)

1−oleoyl−GPG (18:1)*

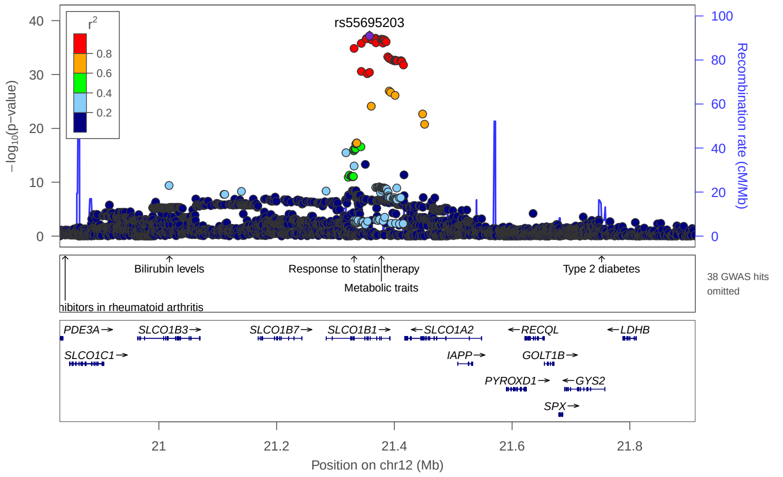

1−palmitoyl−GPG (16:0)*

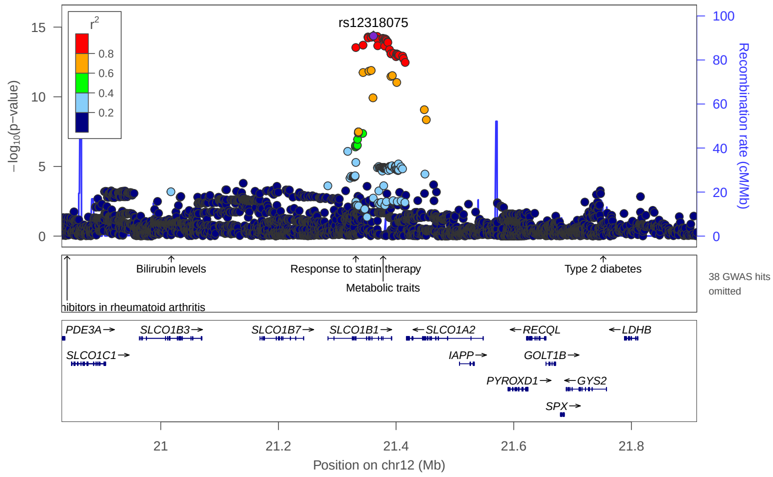

21−hydroxypregnenolone disulfate

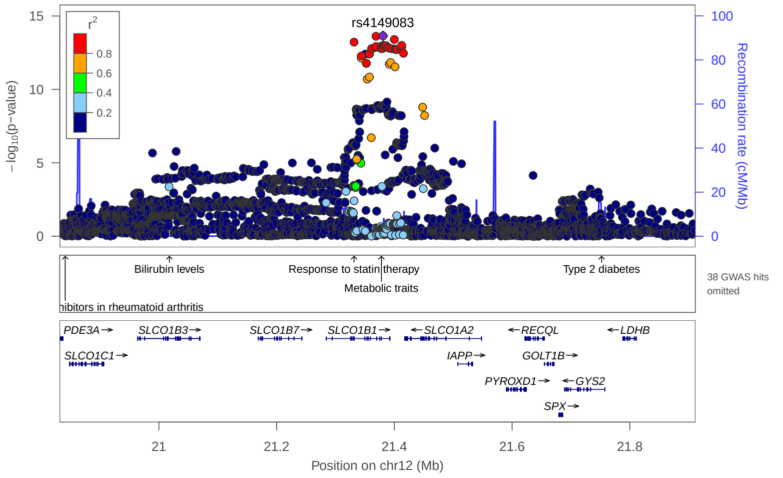

methyl glucopyranoside (alpha + beta)

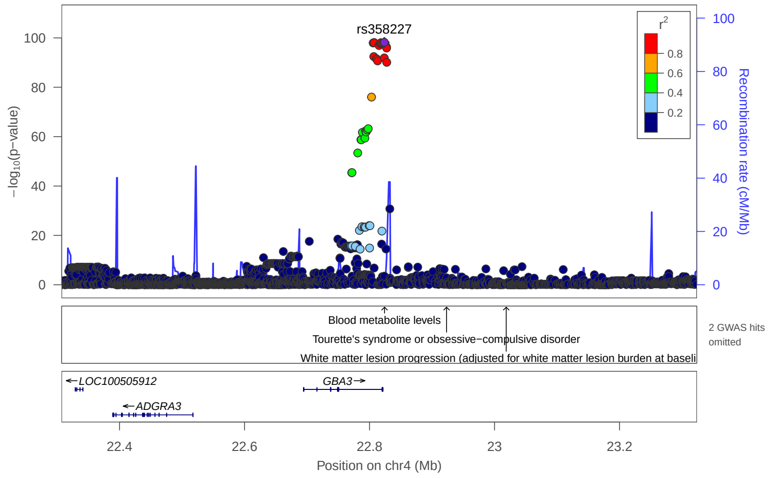

linoleoylcarnitine (C18:2)*

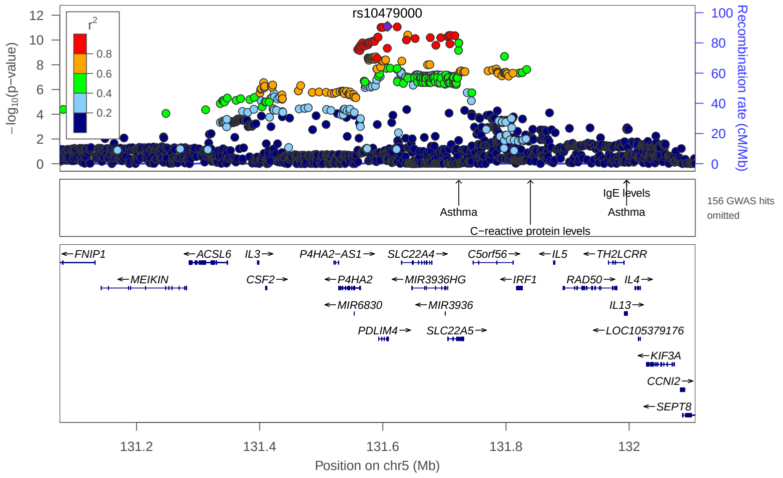

linoleoylcarnitine (C18:2)*

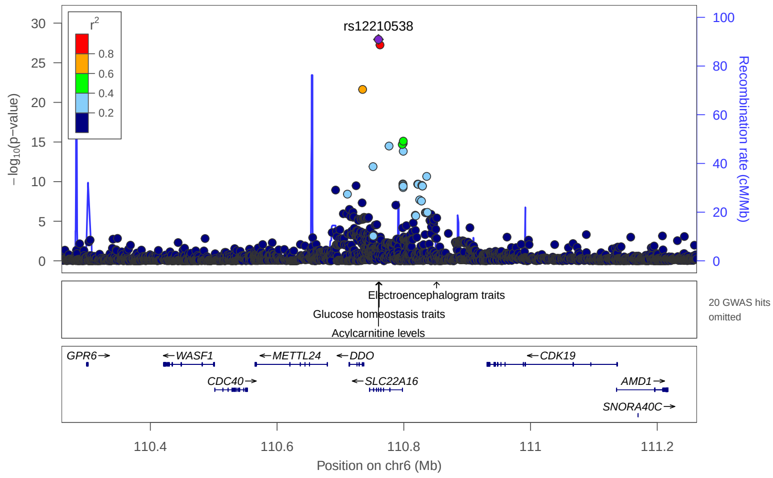

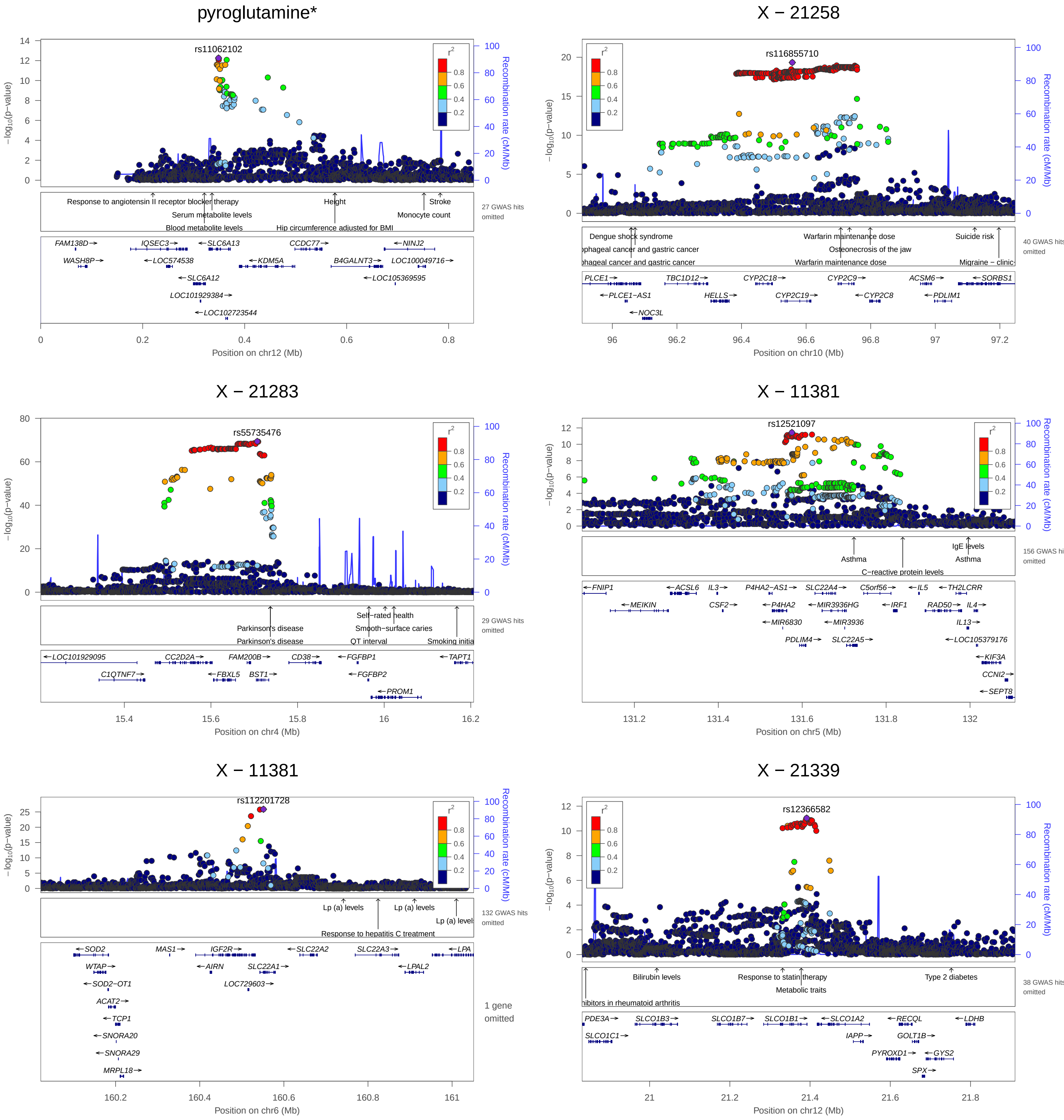

pyroglutamine*
rs11062102
−log10(p−value)
Recombination rate (cM/Mb)
Response to angiotensin II receptor blocker therapy
Serum metabolite levels
Blood metabolite levels
Height
Hip circumference adjusted for BMI
Stroke
Monocyte count
27 GWAS hits omitted
FAM138D
WASH8P
IQSEC3
LOC574538
SLC6A12
LOC101929384
LOC102723544
SLC6A13
KDM5A
CCDC77
B4GALNT3
LOC105369595
NINJ2
LOC100049716
Position on chr12 (Mb)
X − 21258
rs116855710
Dengue shock syndrome
Warfarin maintenance dose
Osteonecrosis of the jaw
Suicide risk
Migraine − clinic
40 GWAS hits omitted
PLCE1
PLCE1−AS1
NOC3L
TBC1D12
HELLS
CYP2C18
CYP2C19
CYP2C9
CYP2C8
ACSM6
PDLIM1
SORBS1
Position on chr10 (Mb)
X − 21283
rs55735476
Parkinson's disease
Self−rated health
Smooth−surface caries
QT interval
Smoking initia
29 GWAS hits omitted
LOC101929095
C1QTNF7
CC2D2A
FBXL5
FAM200B
BST1
CD38
FGFBP1
FGFBP2
PROM1
TAPT1
Position on chr4 (Mb)
X − 11381
rs12521097
Asthma
IgE levels
C−reactive protein levels
156 GWAS hits omitted
FNIP1
MEIKIN
ACSL6
IL3
CSF2
P4HA2−AS1
P4HA2
MIR6830
PDLIM4
SLC22A4
MIR3936HG
MIR3936
SLC22A5
C5orf56
IRF1
IL5
RAD50
TH2LCRR
IL4
IL13
LOC105379176
KIF3A
CCNI2
SEPT8
Position on chr5 (Mb)
X − 11381
rs112201728
Lp (a) levels
Response to hepatitis C treatment
132 GWAS hits omitted
SOD2
WTAP
SOD2−OT1
ACAT2
TCP1
SNORA20
SNORA29
MRPL18
MAS1
IGF2R
AIRN
SLC22A1
LOC729603
SLC22A2
SLC22A3
LPAL2
LPA
1 gene omitted
Position on chr6 (Mb)
X − 21339
rs12366582
Bilirubin levels
Response to statin therapy
Metabolic traits
Type 2 diabetes
38 GWAS hits omitted
PDE3A
SLCO1C1
SLCO1B3
SLCO1B7
SLCO1B1
SLCO1A2
IAPP
PYROXD1
RECQL
GOLT1B
GYS2
SPX
LDHB
Position on chr12 (Mb)

X − 11308

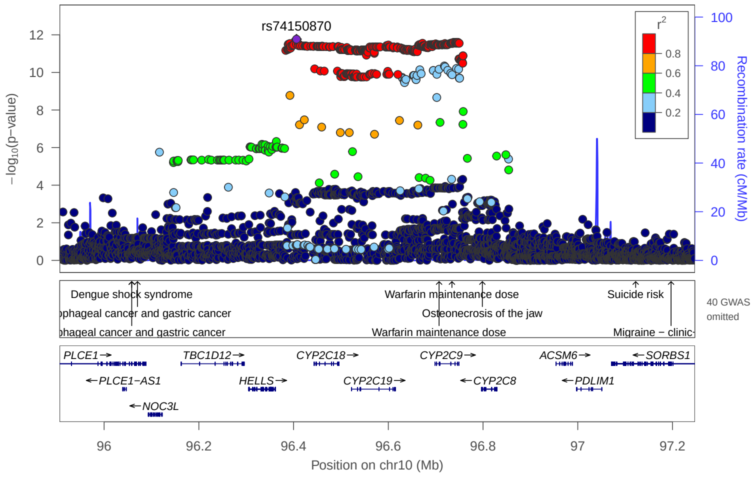

X − 21353

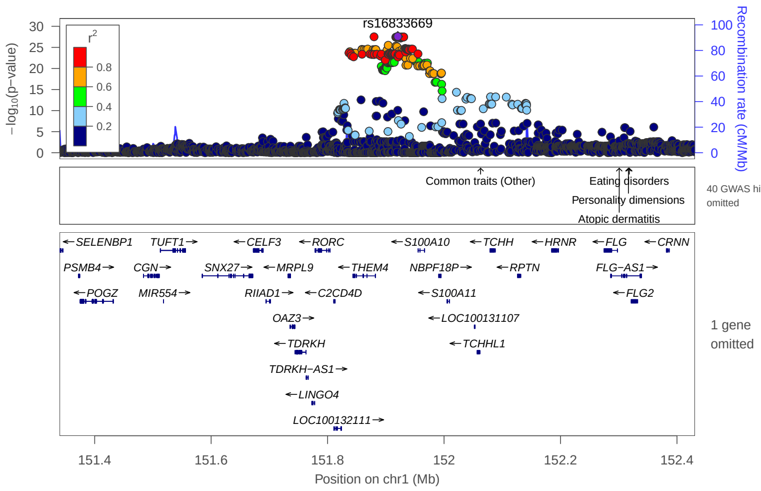

X − 13866

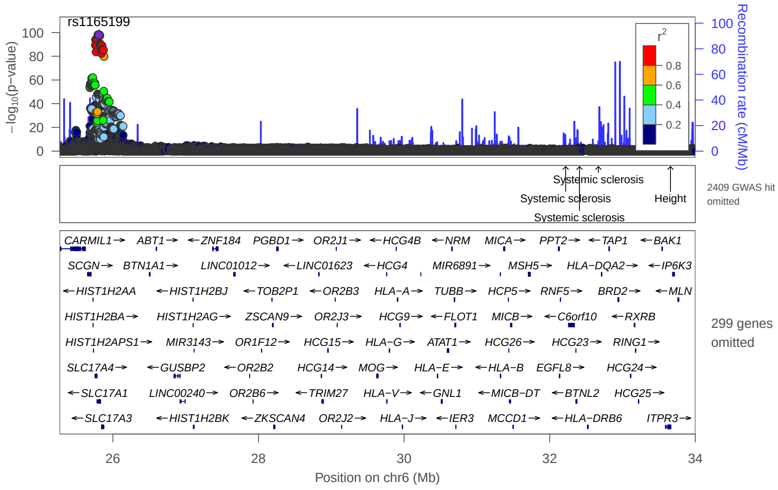

X − 21410

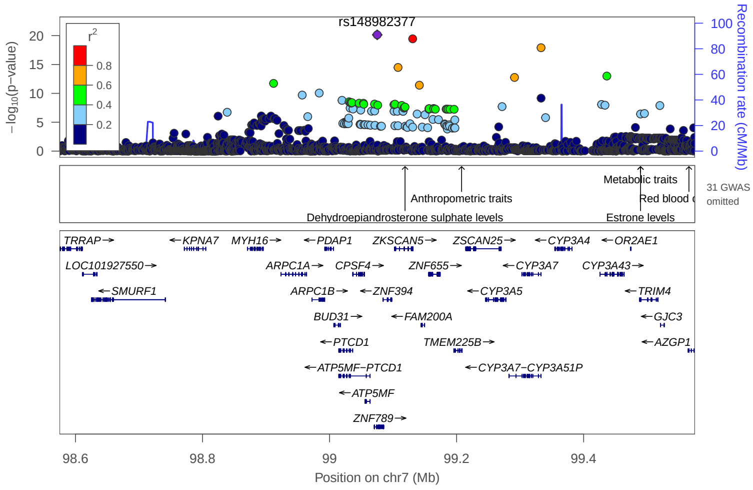

X − 11444

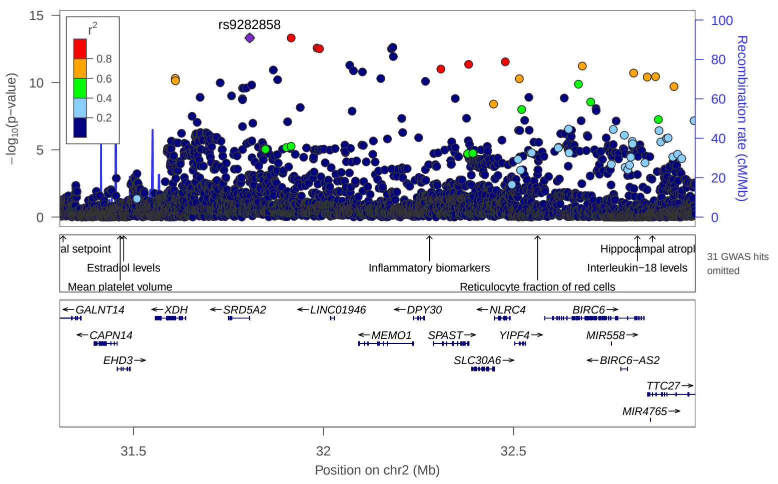

X − 21441

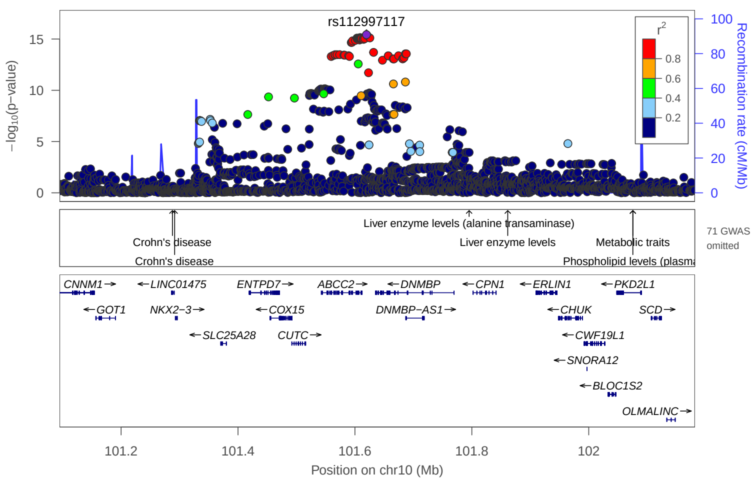

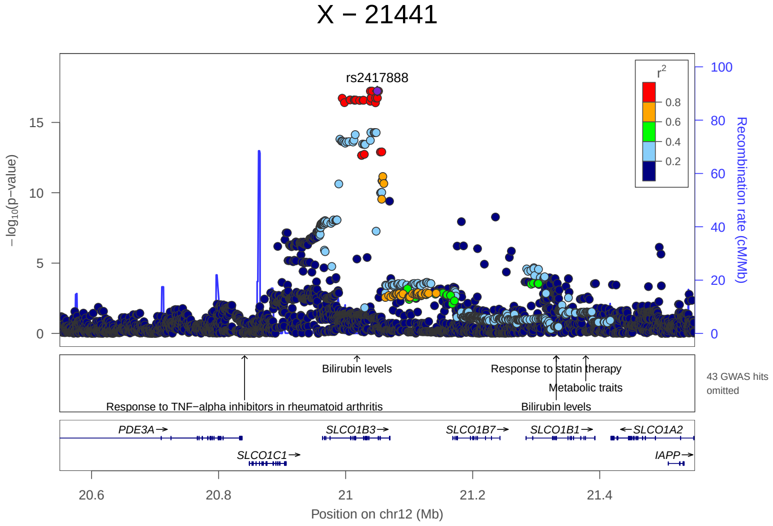
X − 21441
rs2417888
Bilirubin levels
Response to statin therapy
Metabolic traits
Response to TNF−alpha inhibitors in rheumatoid arthritis
Bilirubin levels
43 GWAS hits omitted
Position on chr12 (Mb)

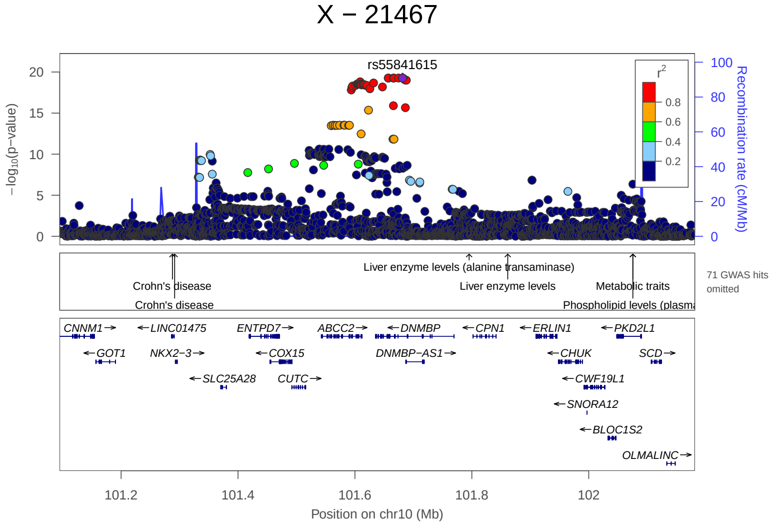
X − 21467
rs55841615
Crohn's disease
Crohn's disease
Liver enzyme levels (alanine transaminase)
Liver enzyme levels
Metabolic traits
Phospholipid levels (plasma
71 GWAS hits omitted
Position on chr10 (Mb)

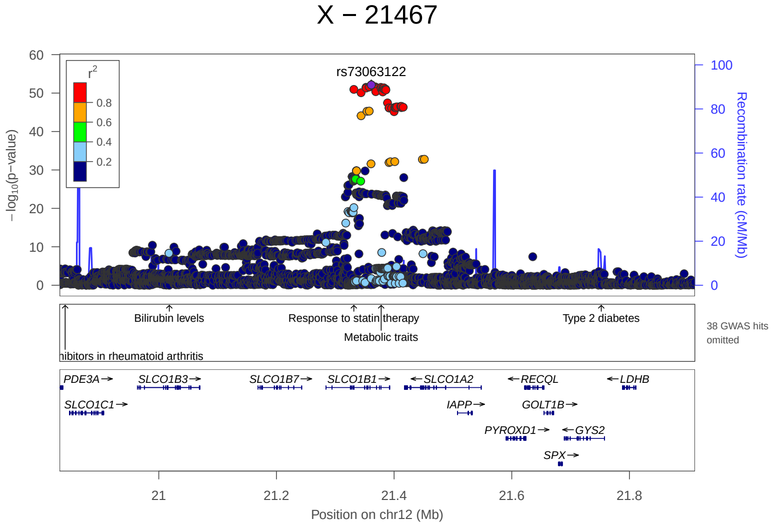
X − 21467
rs73063122
Bilirubin levels
Response to statin therapy
Metabolic traits
Type 2 diabetes
hibitors in rheumatoid arthritis
38 GWAS hits omitted
Position on chr12 (Mb)

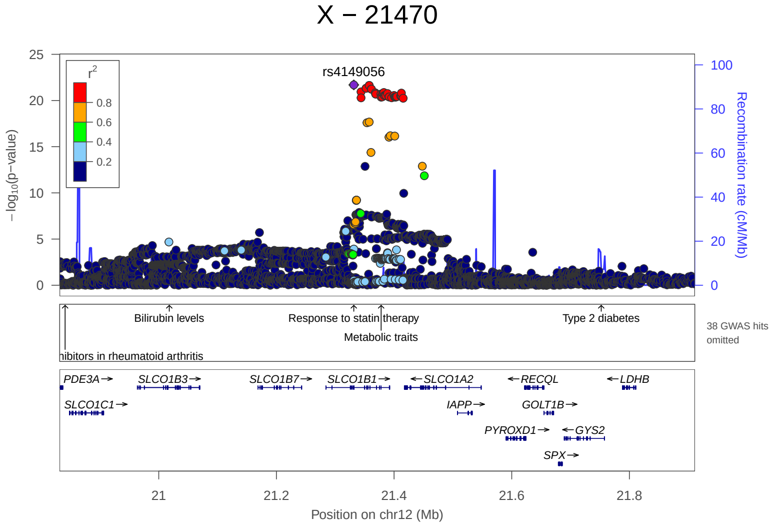
X − 21470
rs4149056
Bilirubin levels
Response to statin therapy
Metabolic traits
Type 2 diabetes
hibitors in rheumatoid arthritis
38 GWAS hits omitted
Position on chr12 (Mb)

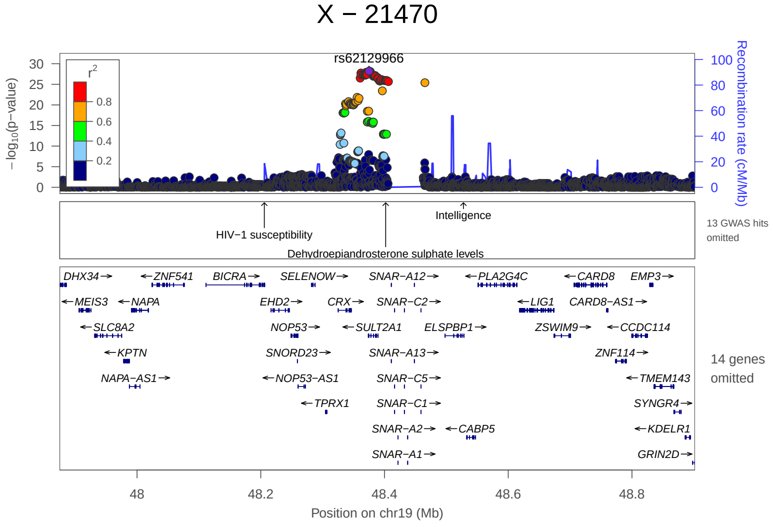
X − 21470
rs62129966
HIV−1 susceptibility
Intelligence
Dehydroepiandrosterone sulphate levels
13 GWAS hits omitted
14 genes omitted
Position on chr19 (Mb)

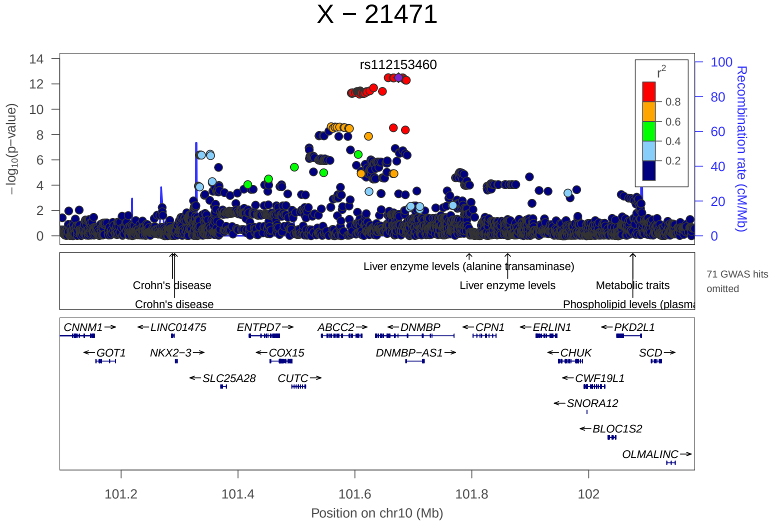
X − 21471
rs112153460
Crohn's disease
Crohn's disease
Liver enzyme levels (alanine transaminase)
Liver enzyme levels
Metabolic traits
Phospholipid levels (plasma
71 GWAS hits omitted
Position on chr10 (Mb)

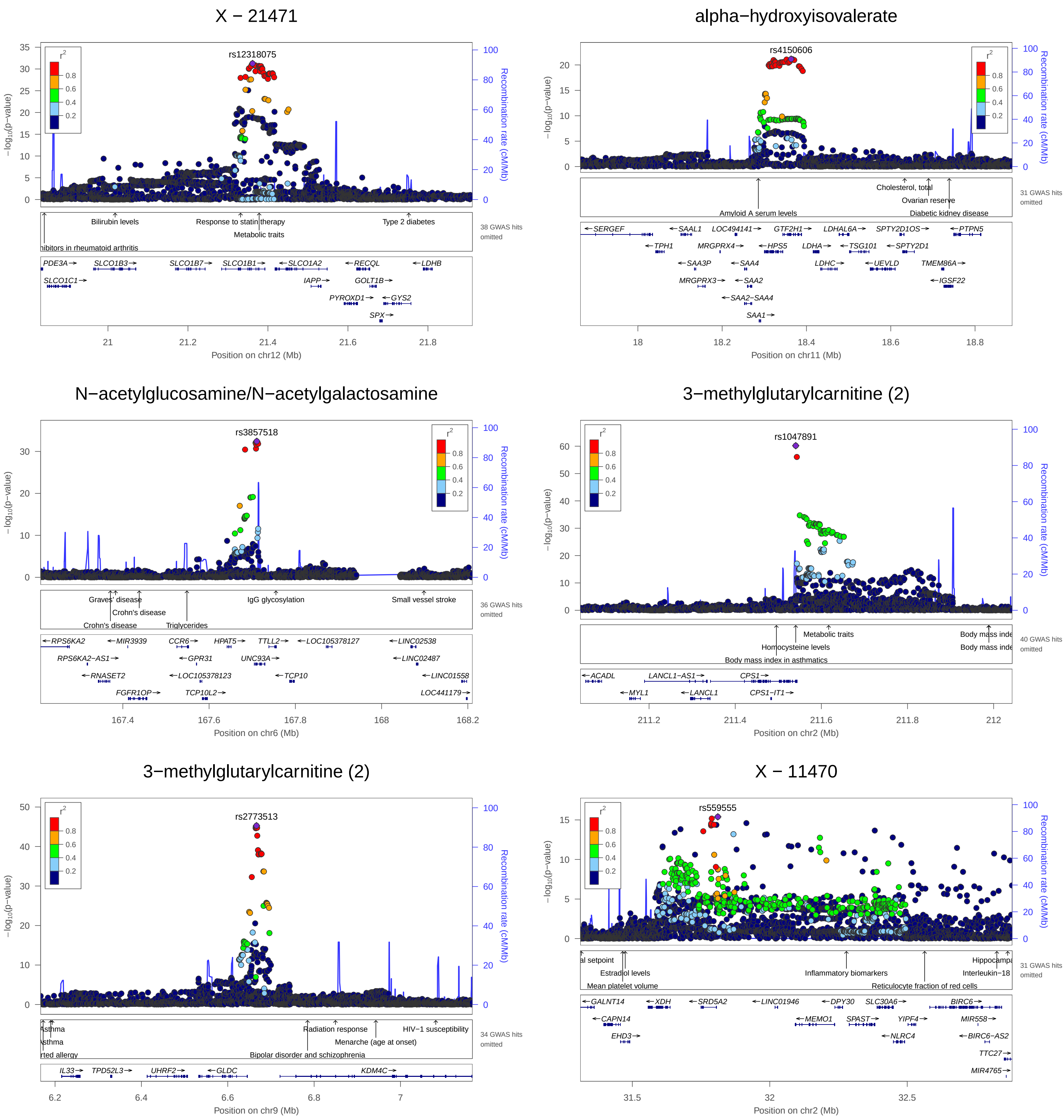

X − 21471
rs12318075
−log10(p−value)
Recombination rate (cM/Mb)
Position on chr12 (Mb)
Bilirubin levels
Response to statin therapy
Metabolic traits
Type 2 diabetes
38 GWAS hits omitted
PDE3A
SLCO1B3
SLCO1B7
SLCO1B1
SLCO1A2
RECQL
LDHB
SLCO1C1
IAPP
GOLT1B
PYROXD1
GYS2
SPX
alpha−hydroxyisovalerate
rs4150606
Position on chr11 (Mb)
Cholesterol, total
Ovarian reserve
Amyloid A serum levels
Diabetic kidney disease
31 GWAS hits omitted
SERGEF
SAAL1
LOC494141
GTF2H1
LDHAL6A
SPTY2D1OS
PTPN5
TPH1
MRGPRX4
HPS5
LDHA
TSG101
SPTY2D1
SAA3P
SAA4
LDHC
UEVLD
TMEM86A
MRGPRX3
SAA2
IGSF22
SAA2−SAA4
SAA1
N−acetylglucosamine/N−acetylgalactosamine
rs3857518
Position on chr6 (Mb)
Graves' disease
Crohn's disease
Triglycerides
IgG glycosylation
Small vessel stroke
36 GWAS hits omitted
RPS6KA2
MIR3939
CCR6
HPAT5
TTLL2
LOC105378127
LINC02538
RPS6KA2−AS1
GPR31
UNC93A
LINC02487
RNASET2
LOC105378123
TCP10
LINC01558
FGFR1OP
TCP10L2
LOC441179
3−methylglutarylcarnitine (2)
rs1047891
Position on chr2 (Mb)
Metabolic traits
Homocysteine levels
Body mass index in asthmatics
40 GWAS hits omitted
ACADL
LANCL1−AS1
CPS1
MYL1
LANCL1
CPS1−IT1
3−methylglutarylcarnitine (2)
rs2773513
Position on chr9 (Mb)
Asthma
Radiation response
HIV−1 susceptibility
Menarche (age at onset)
Bipolar disorder and schizophrenia
34 GWAS hits omitted
IL33
TPD52L3
UHRF2
GLDC
KDM4C
X − 11470
rs559555
Position on chr2 (Mb)
Estradiol levels
Mean platelet volume
Inflammatory biomarkers
Interleukin−18
Reticulocyte fraction of red cells
31 GWAS hits omitted
GALNT14
XDH
SRD5A2
LINC01946
DPY30
SLC30A6
BIRC6
CAPN14
MEMO1
SPAST
YIPF4
MIR558
EHD3
NLRC4
BIRC6−AS2
TTC27
MIR4765

X − 11470

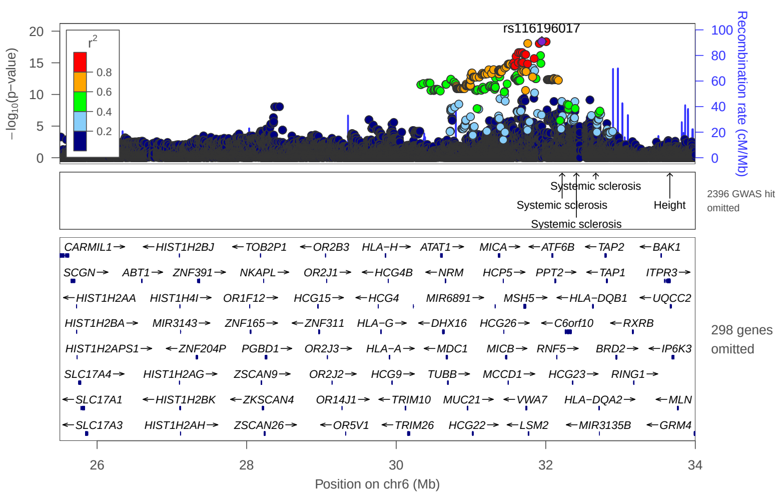

X − 11478

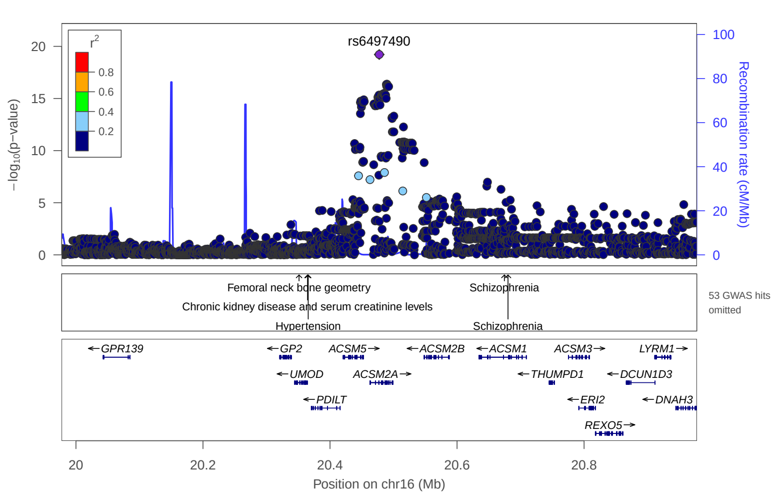

X − 11849

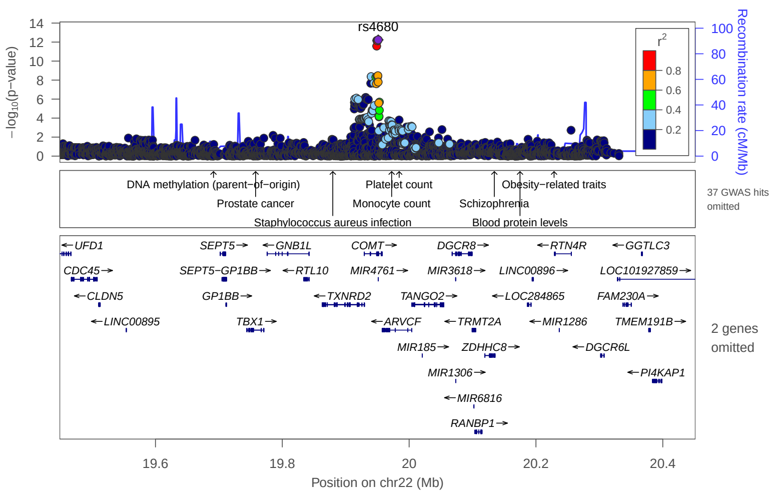

X − 12216

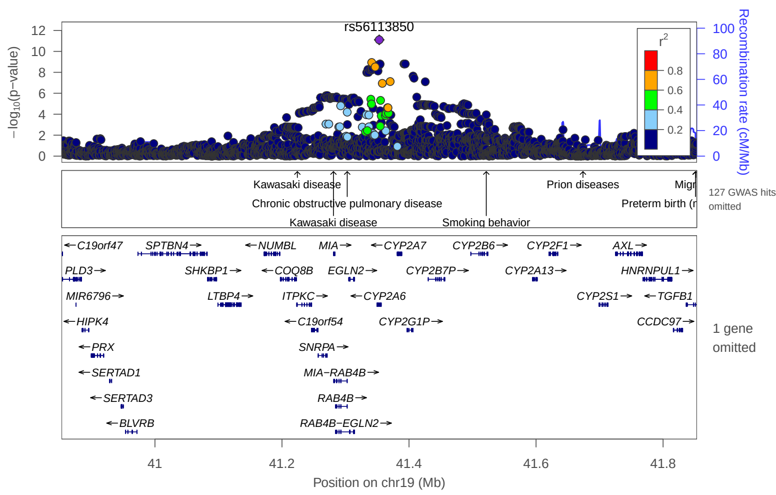

X − 12411

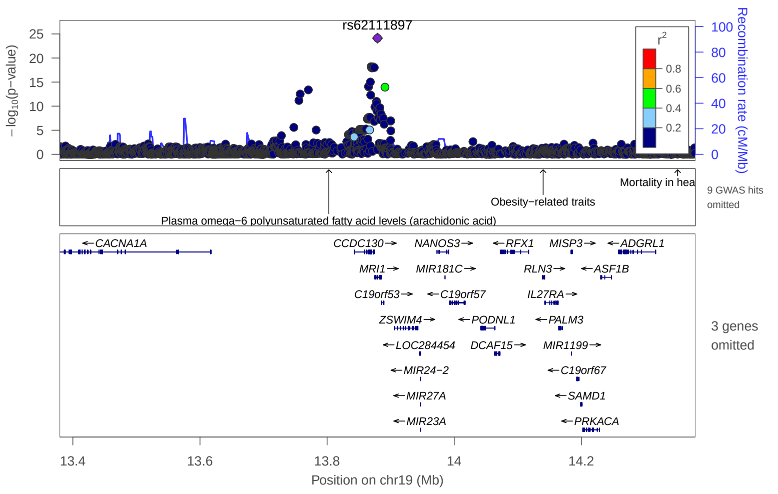

X − 12798

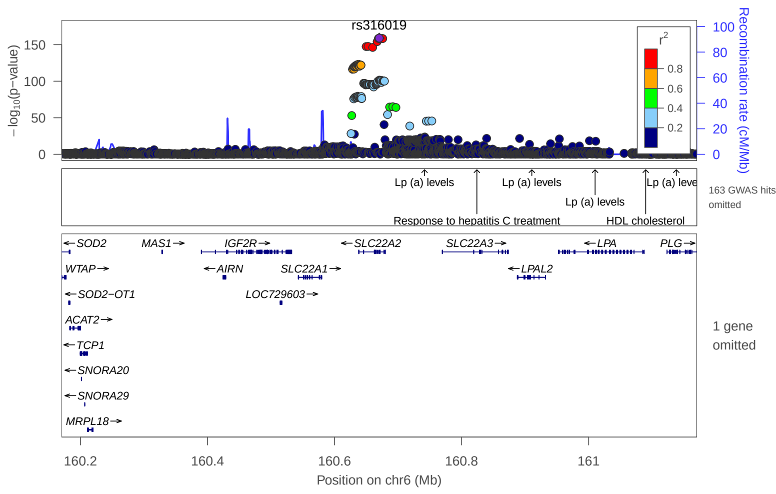

X − 12798

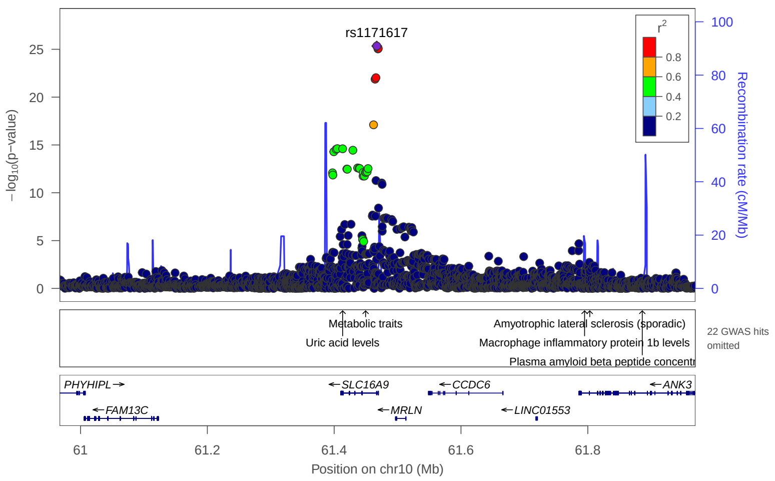

X − 12844

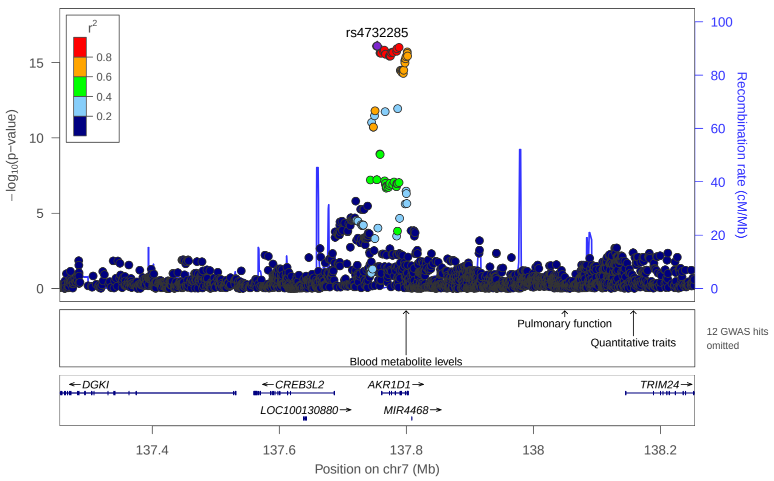

X − 14939

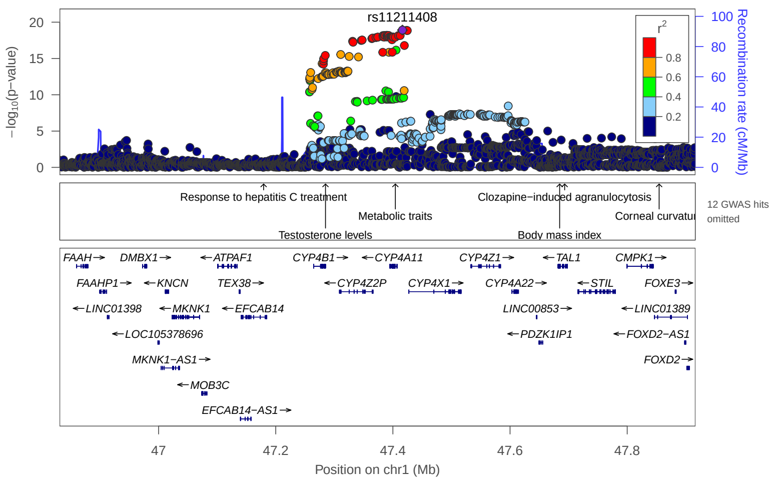

X − 16576

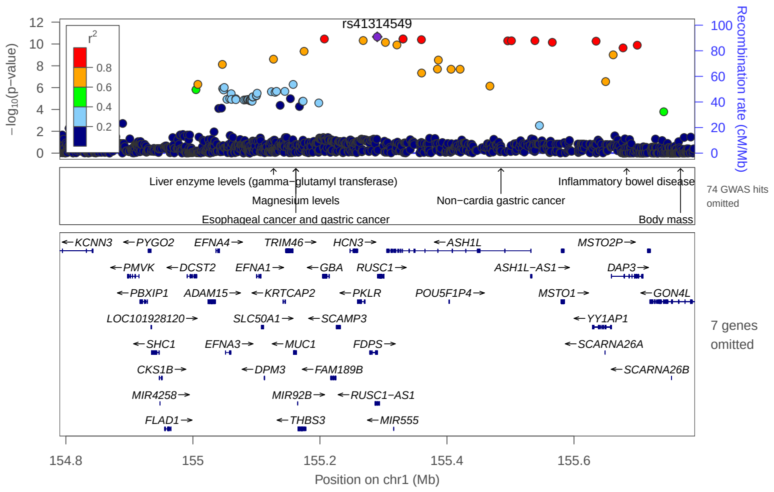

X − 21607

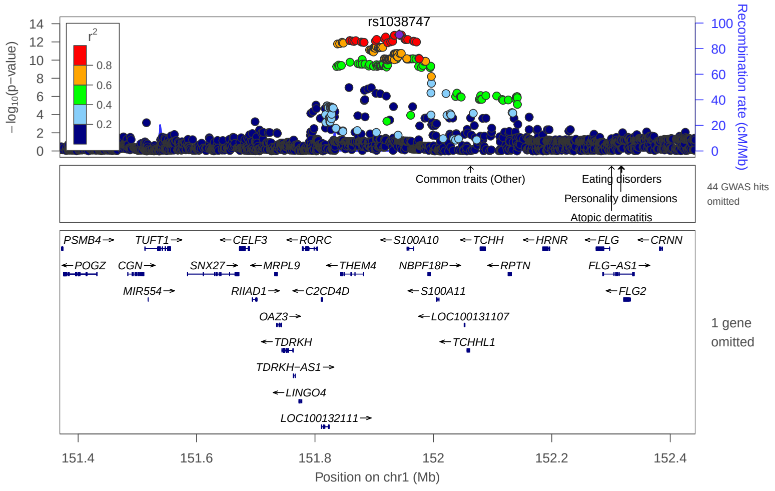

X − 21607

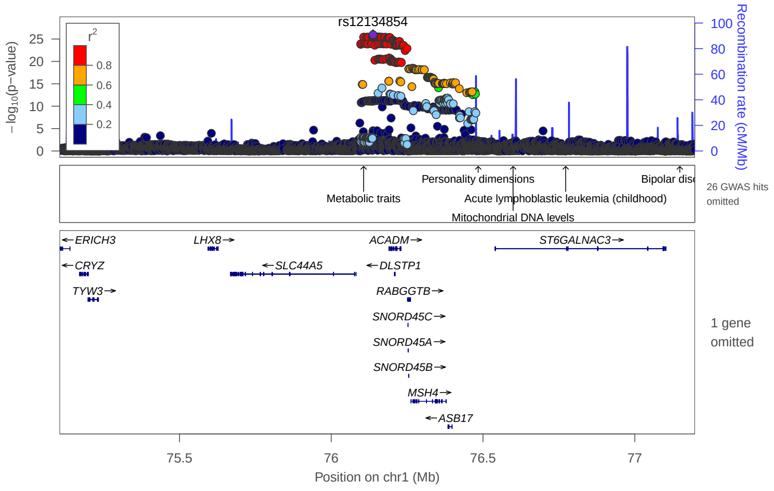

X − 21607

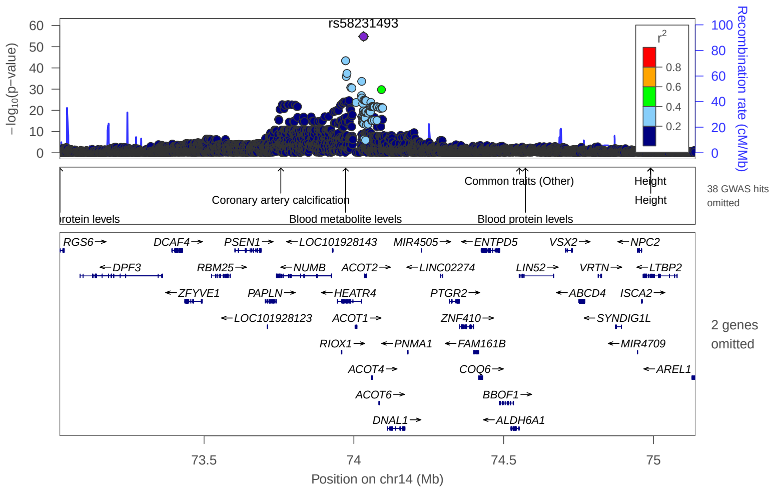

X − 18922

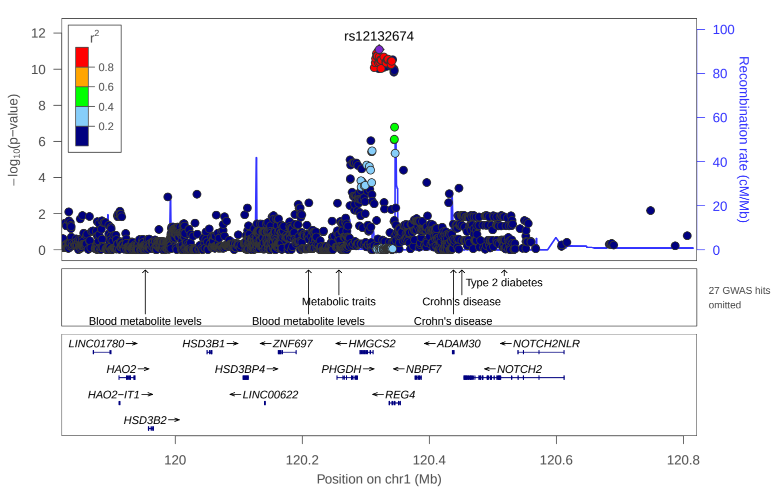

X − 18922

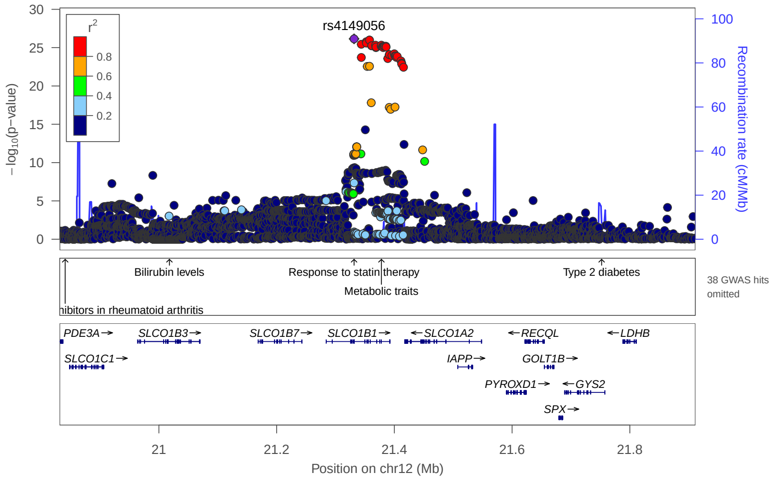

X − 19141

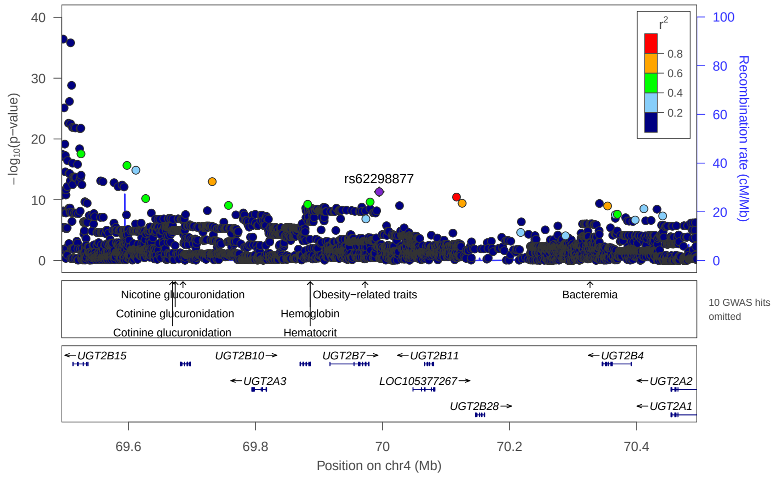

X − 19141

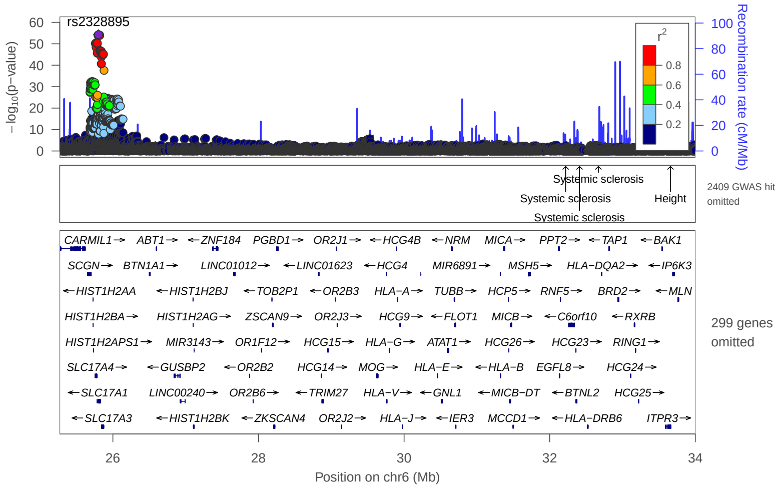

X − 17653

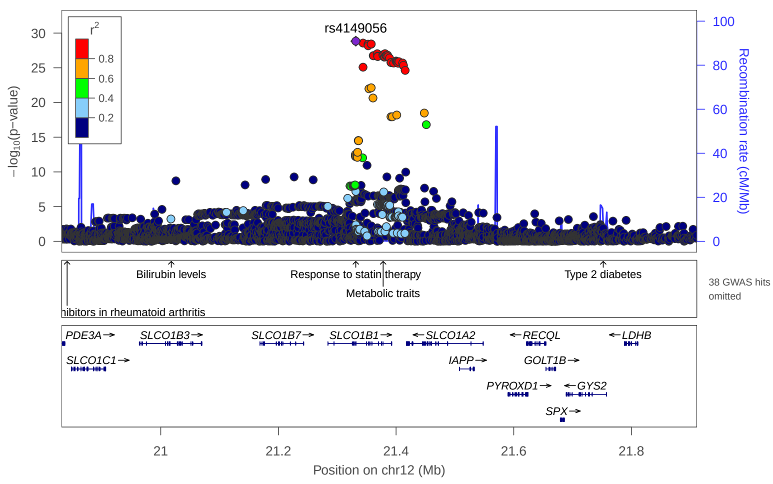

X − 17653

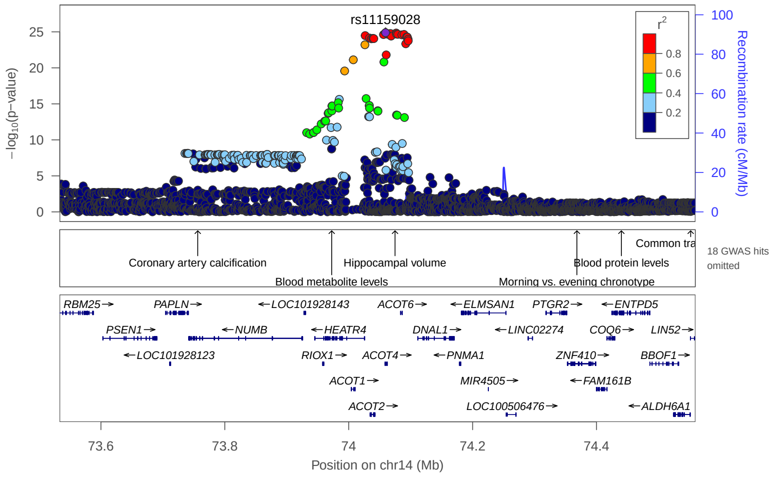

X − 17654

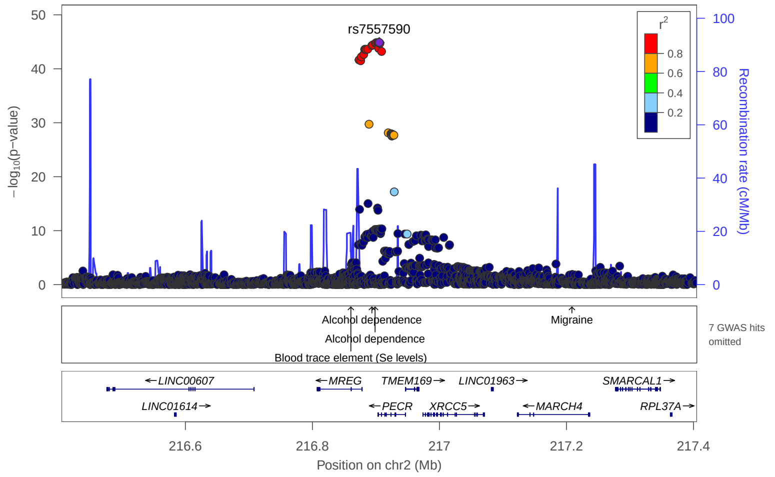

X − 17654

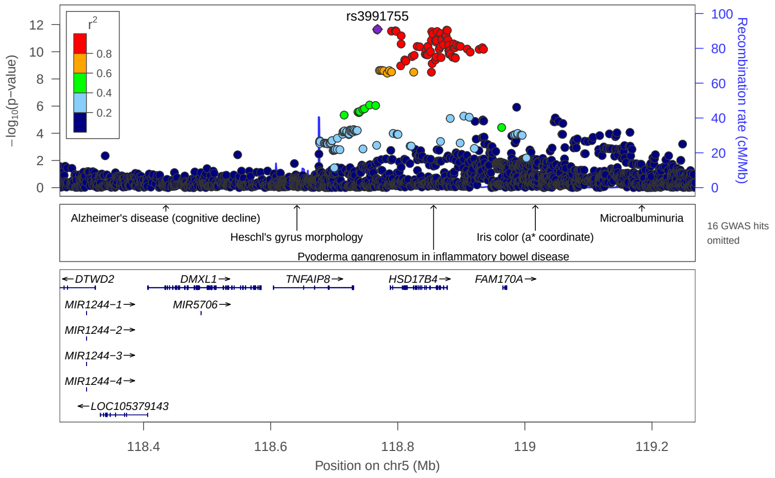

X − 17654

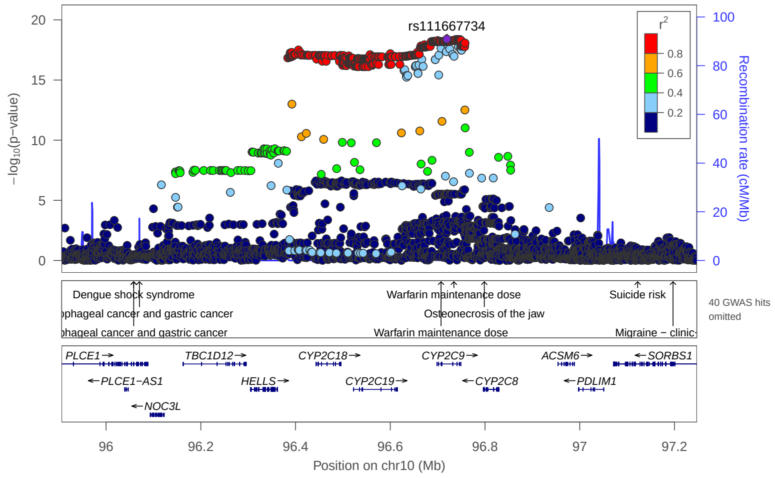

X − 17654

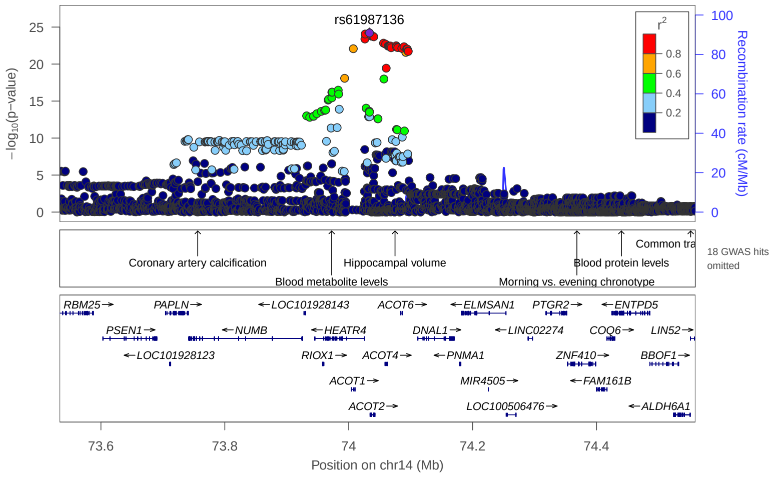

oleoyl−linoleoyl−glycerol (18:1/18:2) [1]

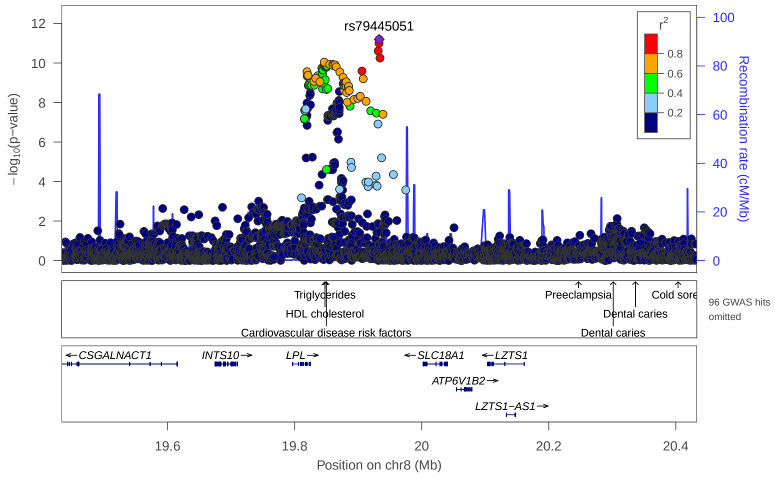

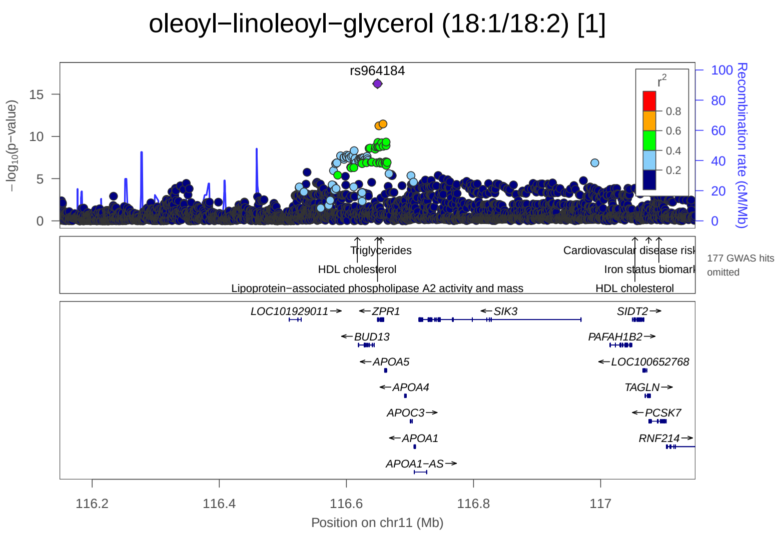
oleoyl−linoleoyl−glycerol (18:1/18:2) [1]
rs964184
r2
0.8
0.6
0.4
0.2
−log10(p−value)
Recombination rate (cM/Mb)
Triglycerides
HDL cholesterol
Lipoprotein−associated phospholipase A2 activity and mass
Cardiovascular disease risk
Iron status biomarkers
HDL cholesterol
177 GWAS hits omitted
LOC101929011
ZPR1
SIK3
SIDT2
BUD13
PAFAH1B2
APOA5
LOC100652768
APOA4
TAGLN
APOC3
PCSK7
APOA1
RNF214
APOA1−AS
Position on chr11 (Mb)

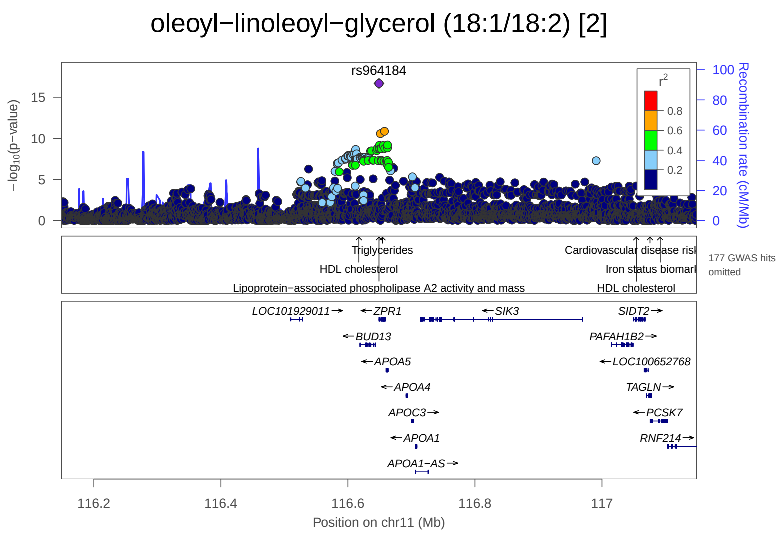
oleoyl−linoleoyl−glycerol (18:1/18:2) [2]
rs964184
r2
0.8
0.6
0.4
0.2
−log10(p−value)
Recombination rate (cM/Mb)
Triglycerides
HDL cholesterol
Lipoprotein−associated phospholipase A2 activity and mass
Cardiovascular disease risk
Iron status biomarkers
HDL cholesterol
177 GWAS hits omitted
LOC101929011
ZPR1
SIK3
SIDT2
BUD13
PAFAH1B2
APOA5
LOC100652768
APOA4
TAGLN
APOC3
PCSK7
APOA1
RNF214
APOA1−AS
Position on chr11 (Mb)

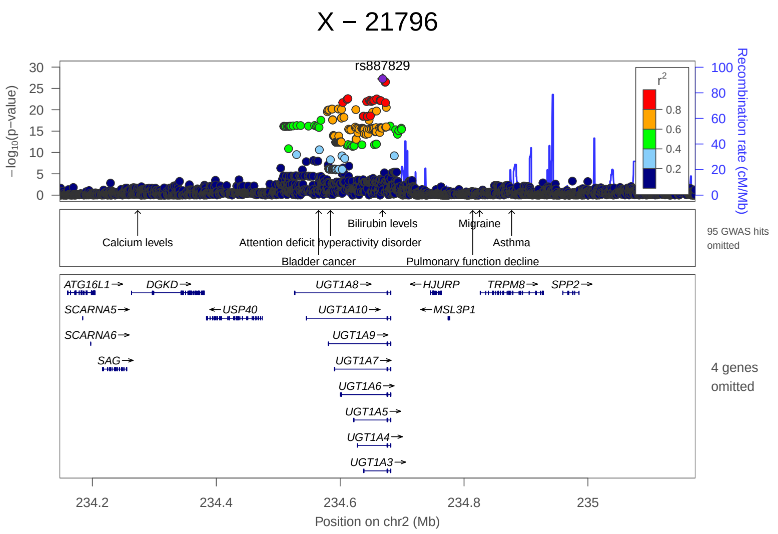
X − 21796
rs887829
r2
0.8
0.6
0.4
0.2
−log10(p−value)
Recombination rate (cM/Mb)
Bilirubin levels
Migraine
Calcium levels
Attention deficit hyperactivity disorder
Asthma
Bladder cancer
Pulmonary function decline
95 GWAS hits omitted
ATG16L1
DGKD
UGT1A8
HJURP
TRPM8
SPP2
SCARNA5
USP40
UGT1A10
MSL3P1
SCARNA6
UGT1A9
SAG
UGT1A7
UGT1A6
UGT1A5
UGT1A4
UGT1A3
4 genes omitted
Position on chr2 (Mb)

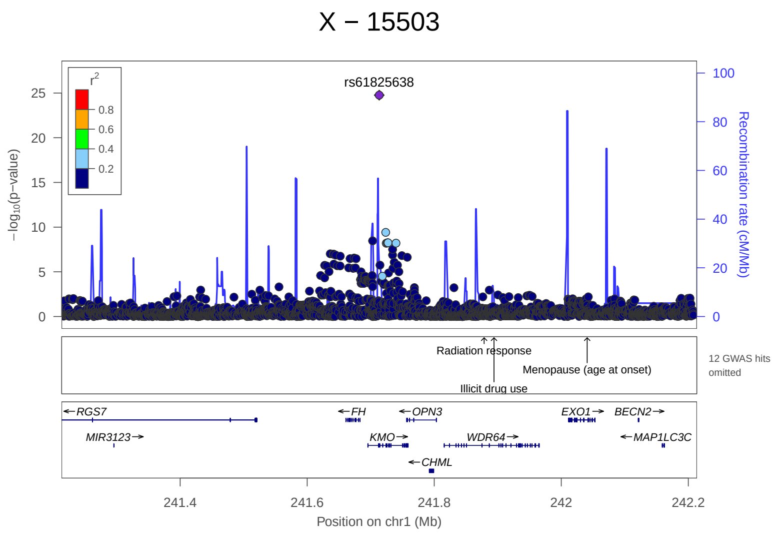
X − 15503
rs61825638
r2
0.8
0.6
0.4
0.2
−log10(p−value)
Recombination rate (cM/Mb)
Radiation response
Menopause (age at onset)
Illicit drug use
12 GWAS hits omitted
RGS7
FH
OPN3
EXO1
BECN2
MIR3123
KMO
WDR64
MAP1LC3C
CHML
Position on chr1 (Mb)

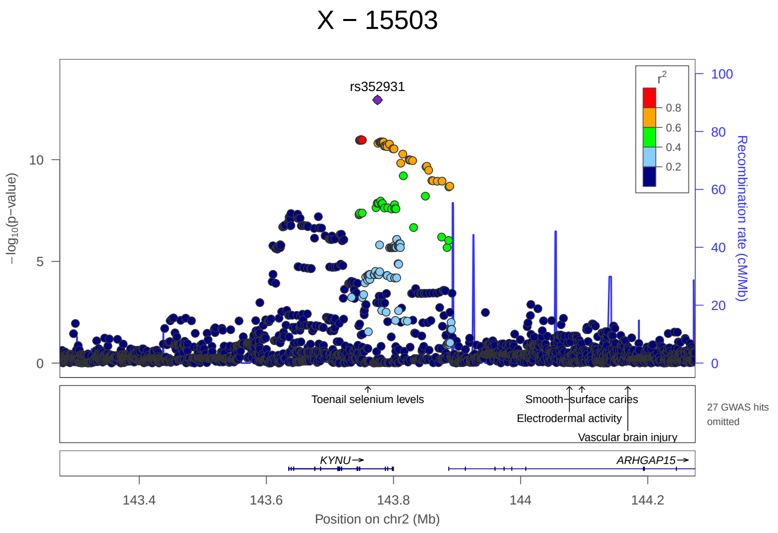
X − 15503
rs352931
r2
0.8
0.6
0.4
0.2
−log10(p−value)
Recombination rate (cM/Mb)
Toenail selenium levels
Smooth−surface caries
Electrodermal activity
Vascular brain injury
27 GWAS hits omitted
KYNU
ARHGAP15
Position on chr2 (Mb)

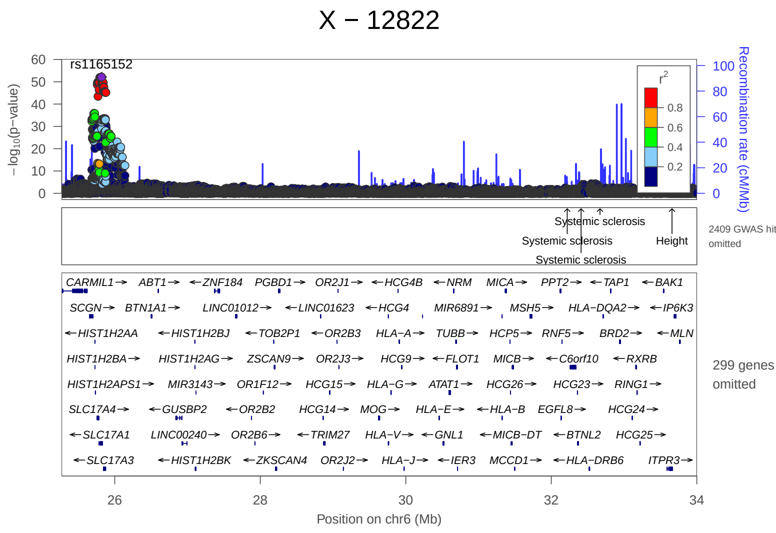
X − 12822
rs1165152
r2
0.8
0.6
0.4
0.2
−log10(p−value)
Recombination rate (cM/Mb)
Systemic sclerosis
Systemic sclerosis
Height
Systemic sclerosis
2409 GWAS hits omitted
CARMIL1
ABT1
ZNF184
PGBD1
OR2J1
HCG4B
NRM
MICA
PPT2
TAP1
BAK1
SCGN
BTN1A1
LINC01012
LINC01623
HCG4
MIR6891
MSH5
HLA−DQA2
IP6K3
HIST1H2AA
HIST1H2BJ
TOB2P1
OR2B3
HLA−A
TUBB
HCP5
RNF5
BRD2
MLN
HIST1H2BA
HIST1H2AG
ZSCAN9
OR2J3
HCG9
FLOT1
MICB
C6orf10
RXRB
HIST1H2APS1
MIR3143
OR1F12
HCG15
HLA−G
ATAT1
HCG26
HCG23
RING1
SLC17A4
GUSBP2
OR2B2
HCG14
MOG
HLA−E
HLA−B
EGFL8
HCG24
SLC17A1
LINC00240
OR2B6
TRIM27
HLA−V
GNL1
MICB−DT
BTNL2
HCG25
SLC17A3
HIST1H2BK
ZKSCAN4
OR2J2
HLA−J
IER3
MCCD1
HLA−DRB6
ITPR3
299 genes omitted
Position on chr6 (Mb)

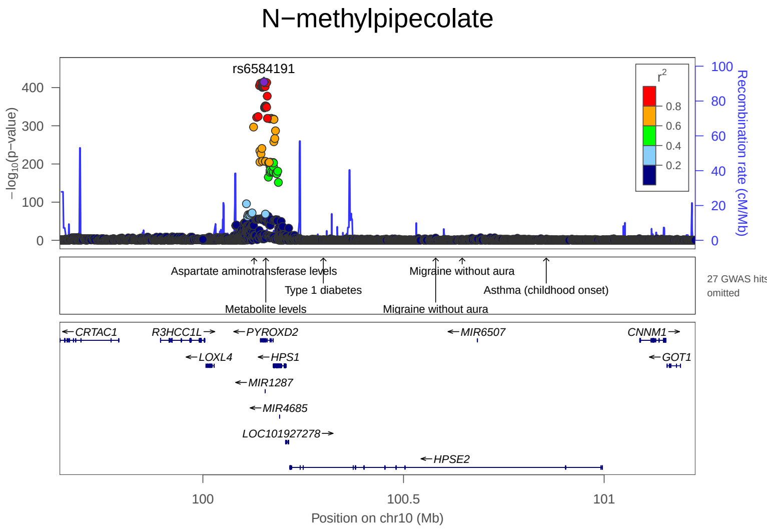
N−methylpipecolate
rs6584191
Aspartate aminotransferase levels
Type 1 diabetes
Metabolite levels
Migraine without aura
Asthma (childhood onset)
Migraine without aura
27 GWAS hits omitted
Position on chr10 (Mb)
Recombination rate (cM/Mb)

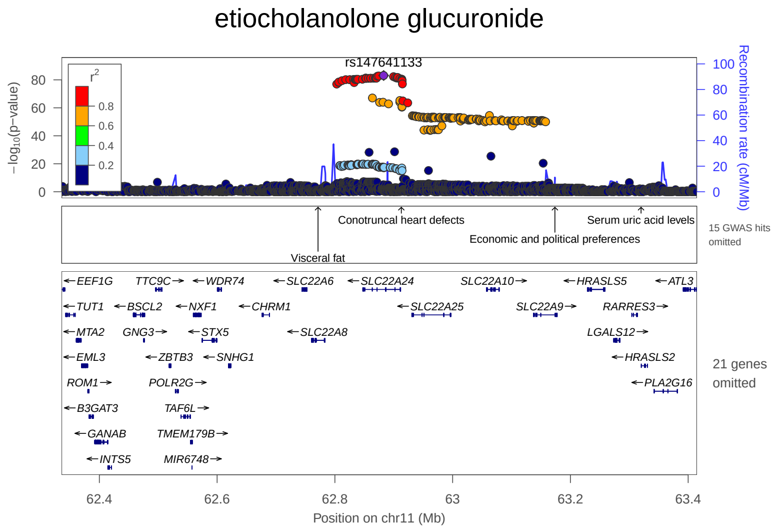
etiocholanolone glucuronide
rs147641133
Conotruncal heart defects
Serum uric acid levels
Economic and political preferences
Visceral fat
15 GWAS hits omitted
21 genes omitted
Position on chr11 (Mb)
Recombination rate (cM/Mb)

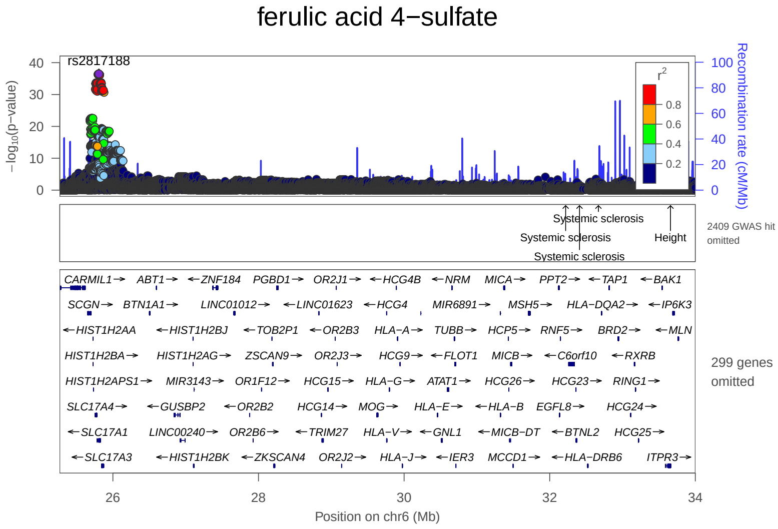
ferulic acid 4−sulfate
rs2817188
Systemic sclerosis
Systemic sclerosis
Height
Systemic sclerosis
2409 GWAS hit omitted
299 genes omitted
Position on chr6 (Mb)
Recombination rate (cM/Mb)

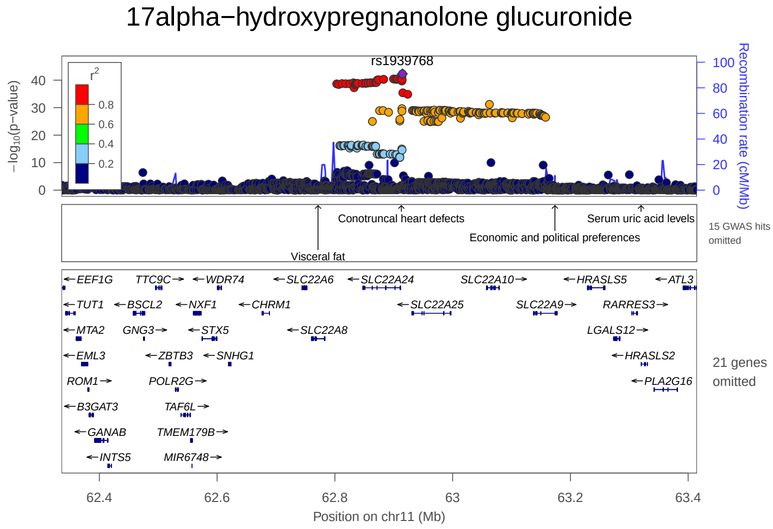
17alpha−hydroxypregnanolone glucuronide
rs1939768
Conotruncal heart defects
Serum uric acid levels
Economic and political preferences
Visceral fat
15 GWAS hits omitted
21 genes omitted
Position on chr11 (Mb)
Recombination rate (cM/Mb)

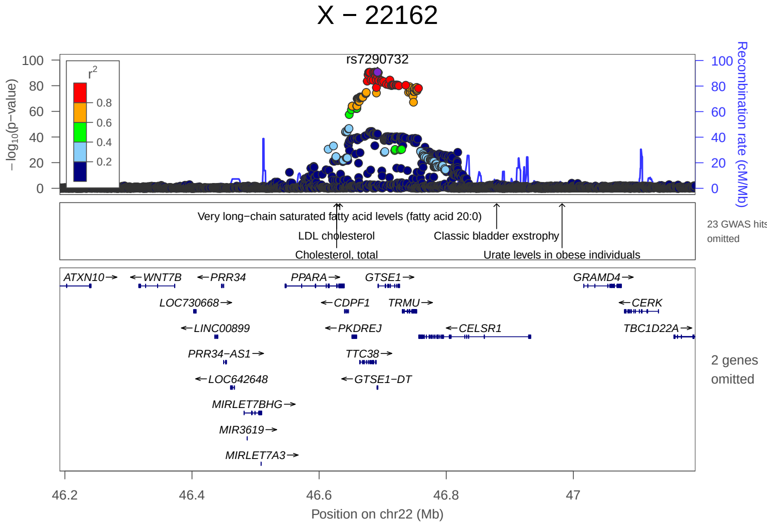
X − 22162
rs7290732
Very long−chain saturated fatty acid levels (fatty acid 20:0)
LDL cholesterol
Classic bladder exstrophy
Cholesterol, total
Urate levels in obese individuals
23 GWAS hits omitted
2 genes omitted
Position on chr22 (Mb)
Recombination rate (cM/Mb)

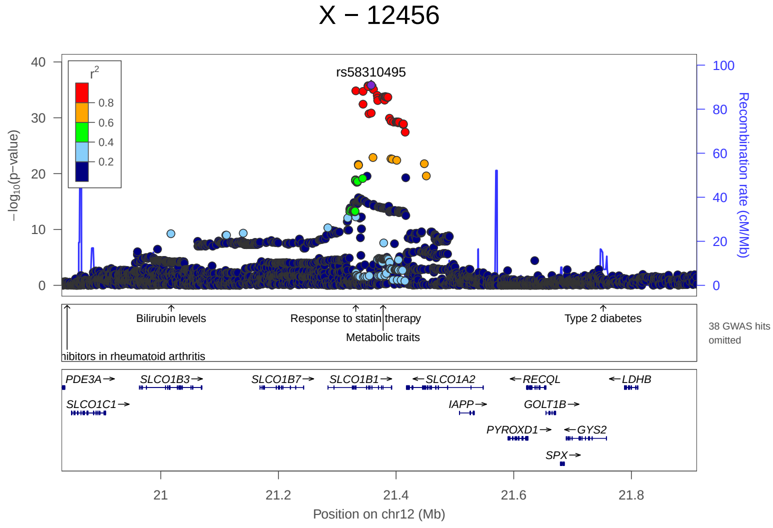
X − 12456
rs58310495
Bilirubin levels
Response to statin therapy
Type 2 diabetes
Metabolic traits
38 GWAS hits omitted
Position on chr12 (Mb)
Recombination rate (cM/Mb)

X − 18921

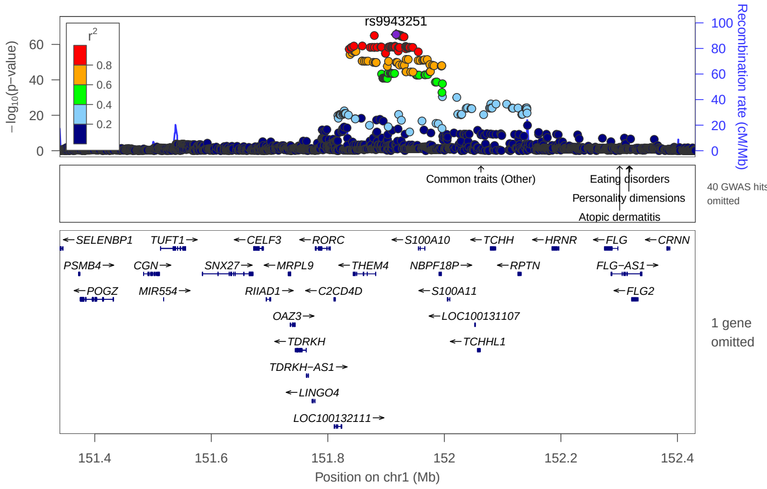

X − 18921

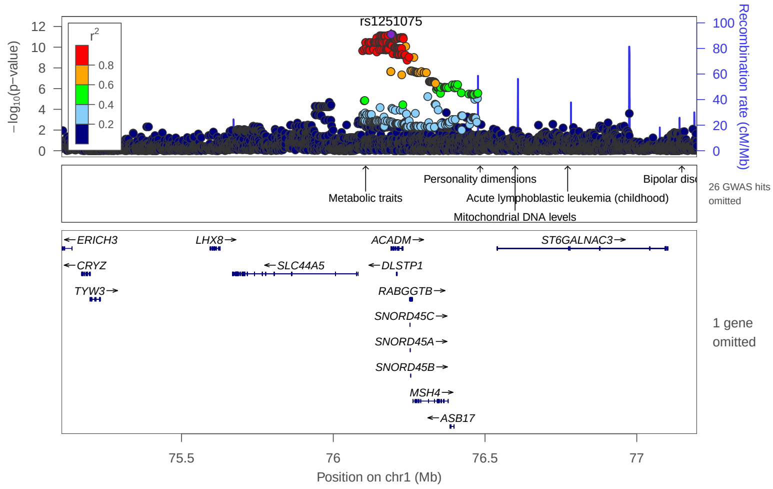

X − 12100

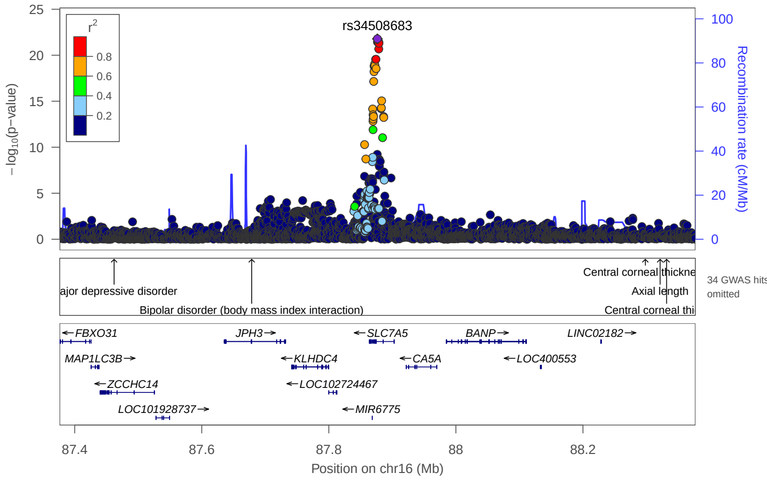

X − 12112

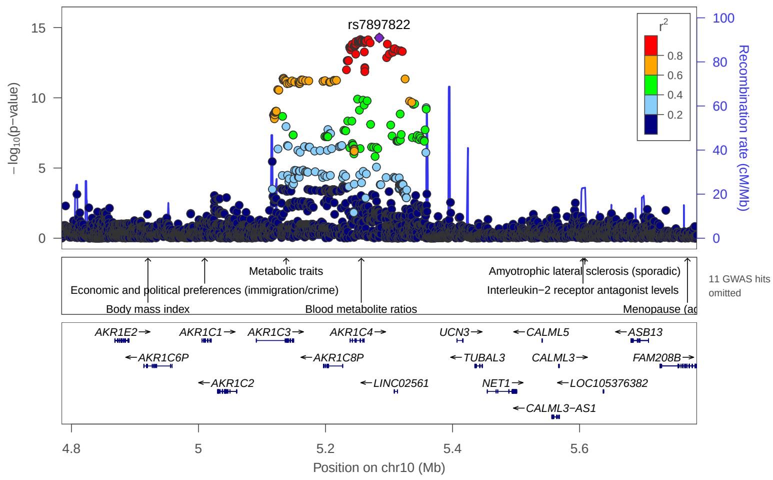

X − 12112

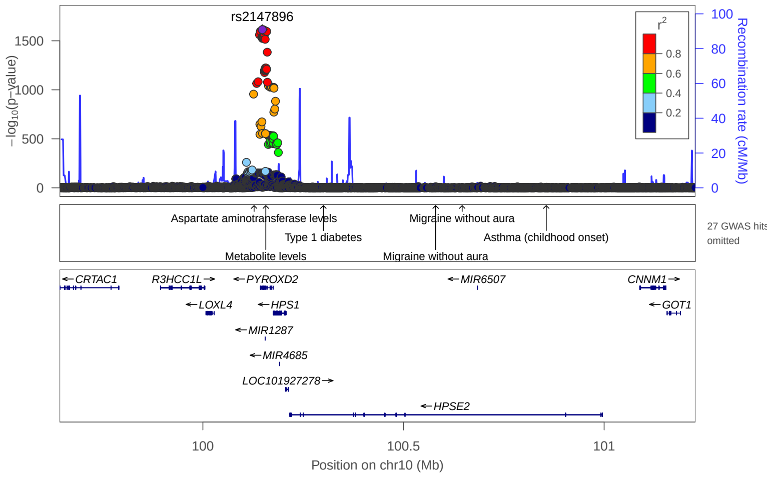

X − 12117

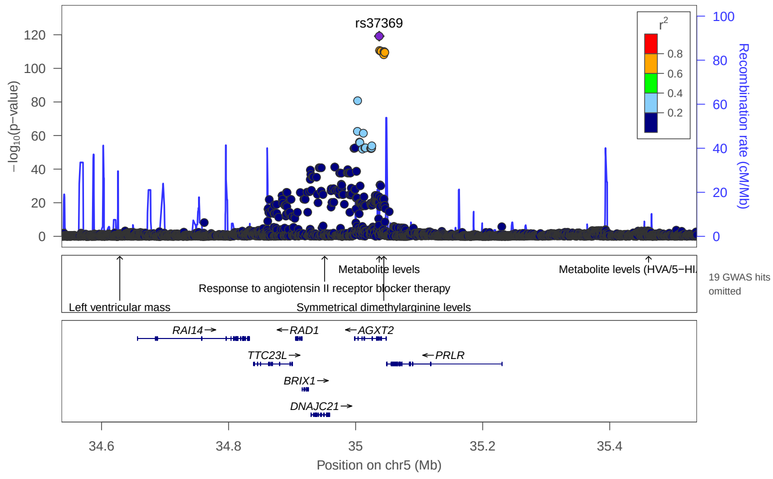

X – 12199

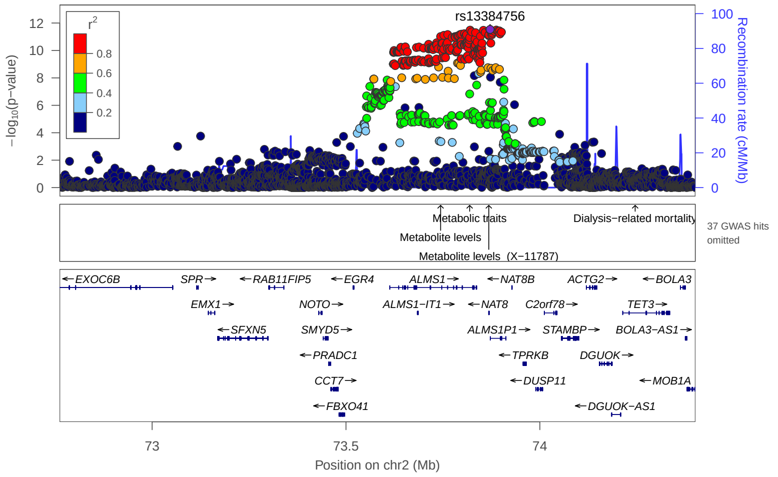

X – 12689

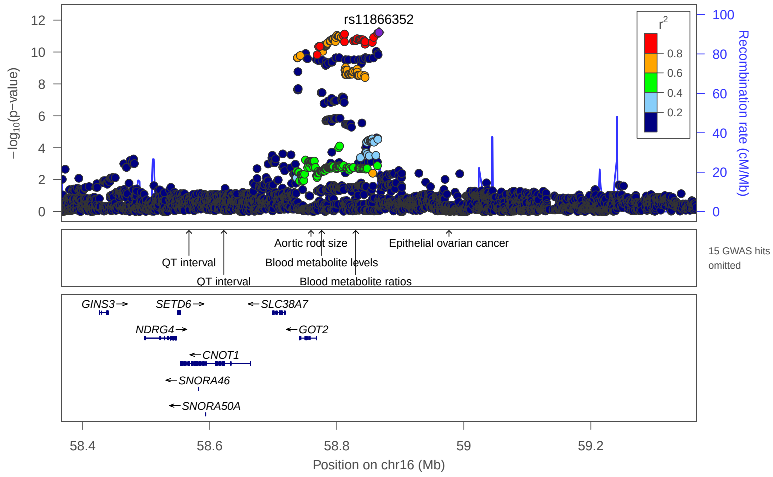

X – 12753

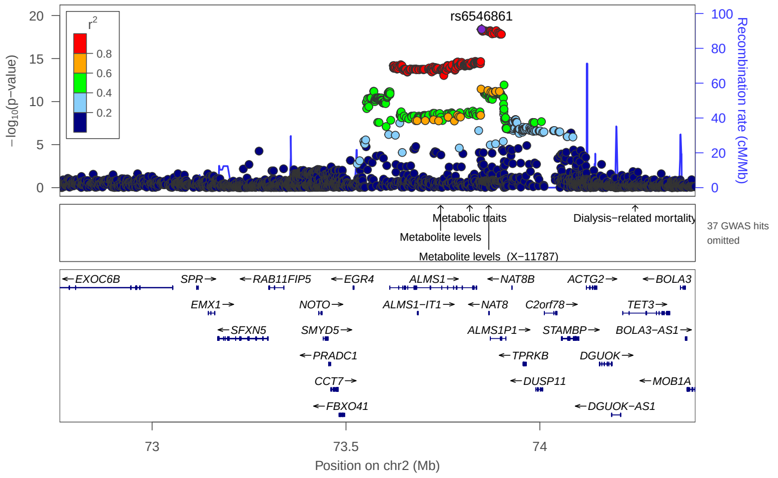

X – 13431

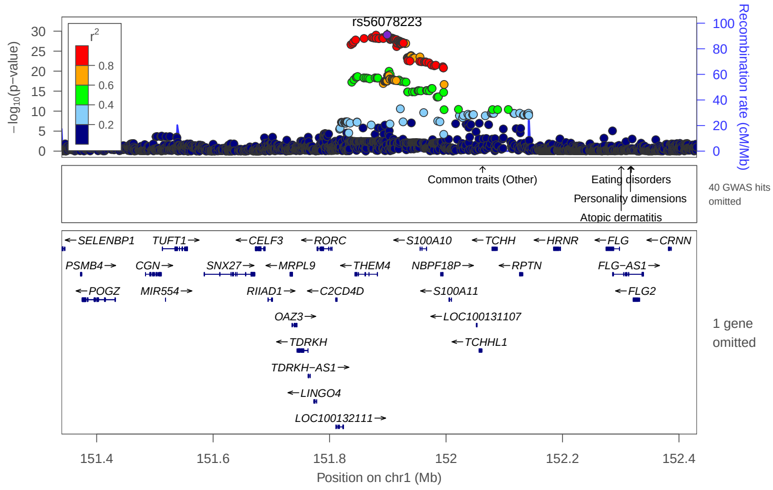

X – 13431

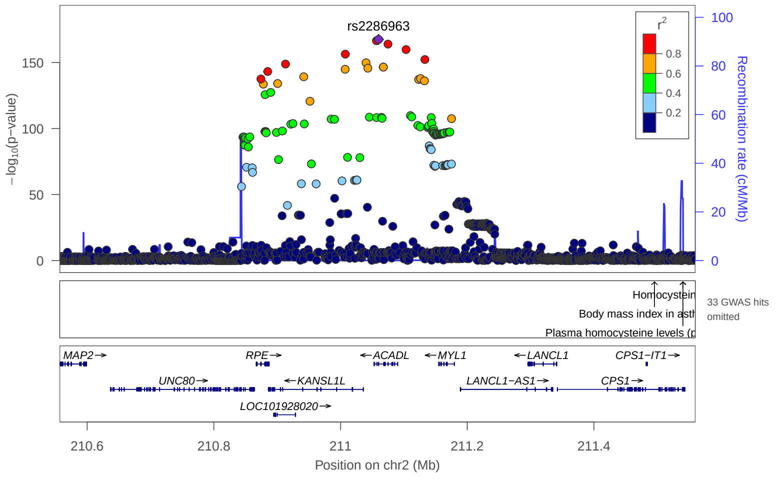

X – 13684

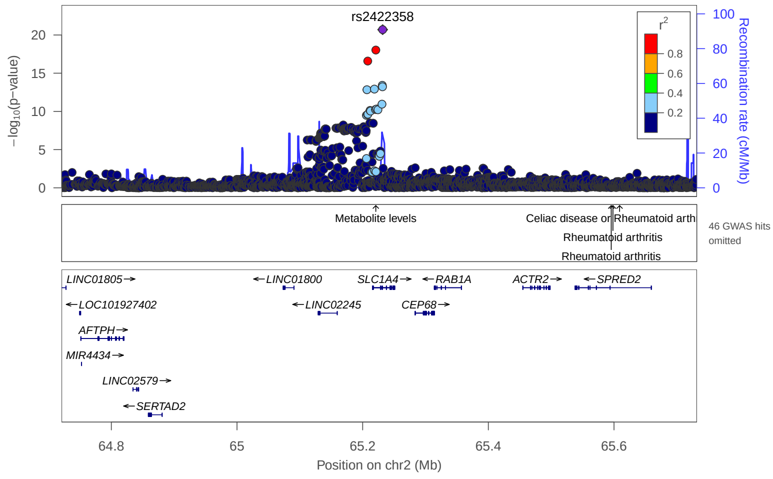

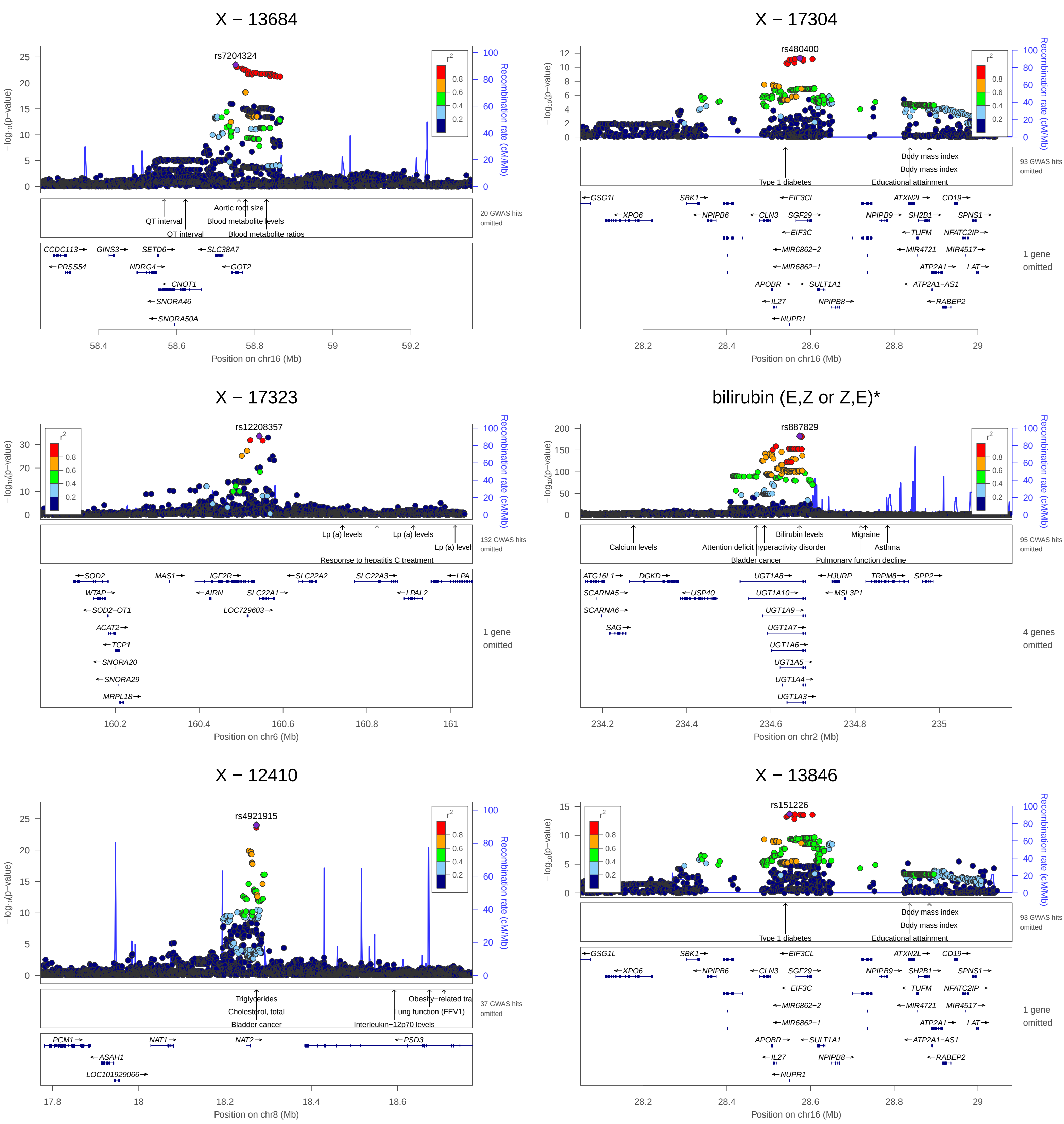

X − 13684
rs7204324
−log10(p−value)
Recombination rate (cM/Mb)
QT interval
Aortic root size
Blood metabolite levels
QT interval
Blood metabolite ratios
20 GWAS hits omitted
CCDC113 GINS3 SETD6 SLC38A7 PRSS54 NDRG4 GOT2 CNOT1 SNORA46 SNORA50A
Position on chr16 (Mb)
X − 17304
rs480400
Body mass index
Body mass index
Type 1 diabetes
Educational attainment
93 GWAS hits omitted
GSG1L SBK1 EIF3CL ATXN2L CD19 XPO6 NPIPB6 CLN3 SGF29 NPIPB9 SH2B1 SPNS1 EIF3C TUFM NFATC2IP MIR6862−2 MIR4721 MIR4517 MIR6862−1 ATP2A1 LAT APOBR SULT1A1 ATP2A1−AS1 IL27 NPIPB8 RABEP2 NUPR1
1 gene omitted
Position on chr16 (Mb)
X − 17323
rs12208357
Lp (a) levels
Lp (a) levels
Lp (a) levels
Response to hepatitis C treatment
132 GWAS hits omitted
SOD2 MAS1 IGF2R SLC22A2 SLC22A3 LPA WTAP AIRN SLC22A1 LPAL2 SOD2−OT1 LOC729603 ACAT2 TCP1 SNORA20 SNORA29 MRPL18
1 gene omitted
Position on chr6 (Mb)
bilirubin (E,Z or Z,E)*
rs887829
Calcium levels
Bilirubin levels
Migraine
Attention deficit hyperactivity disorder
Asthma
Bladder cancer
Pulmonary function decline
95 GWAS hits omitted
ATG16L1 DGKD UGT1A8 HJURP TRPM8 SPP2 SCARNA5 USP40 UGT1A10 MSL3P1 SCARNA6 UGT1A9 SAG UGT1A7 UGT1A6 UGT1A5 UGT1A4 UGT1A3
4 genes omitted
Position on chr2 (Mb)
X − 12410
rs4921915
Triglycerides
Obesity−related tra
Cholesterol, total
Lung function (FEV1)
Bladder cancer
Interleukin−12p70 levels
37 GWAS hits omitted
PCM1 NAT1 NAT2 PSD3 ASAH1 LOC101929066
Position on chr8 (Mb)
X − 13846
rs151226
Body mass index
Body mass index
Type 1 diabetes
Educational attainment
93 GWAS hits omitted
1 gene omitted
Position on chr16 (Mb)

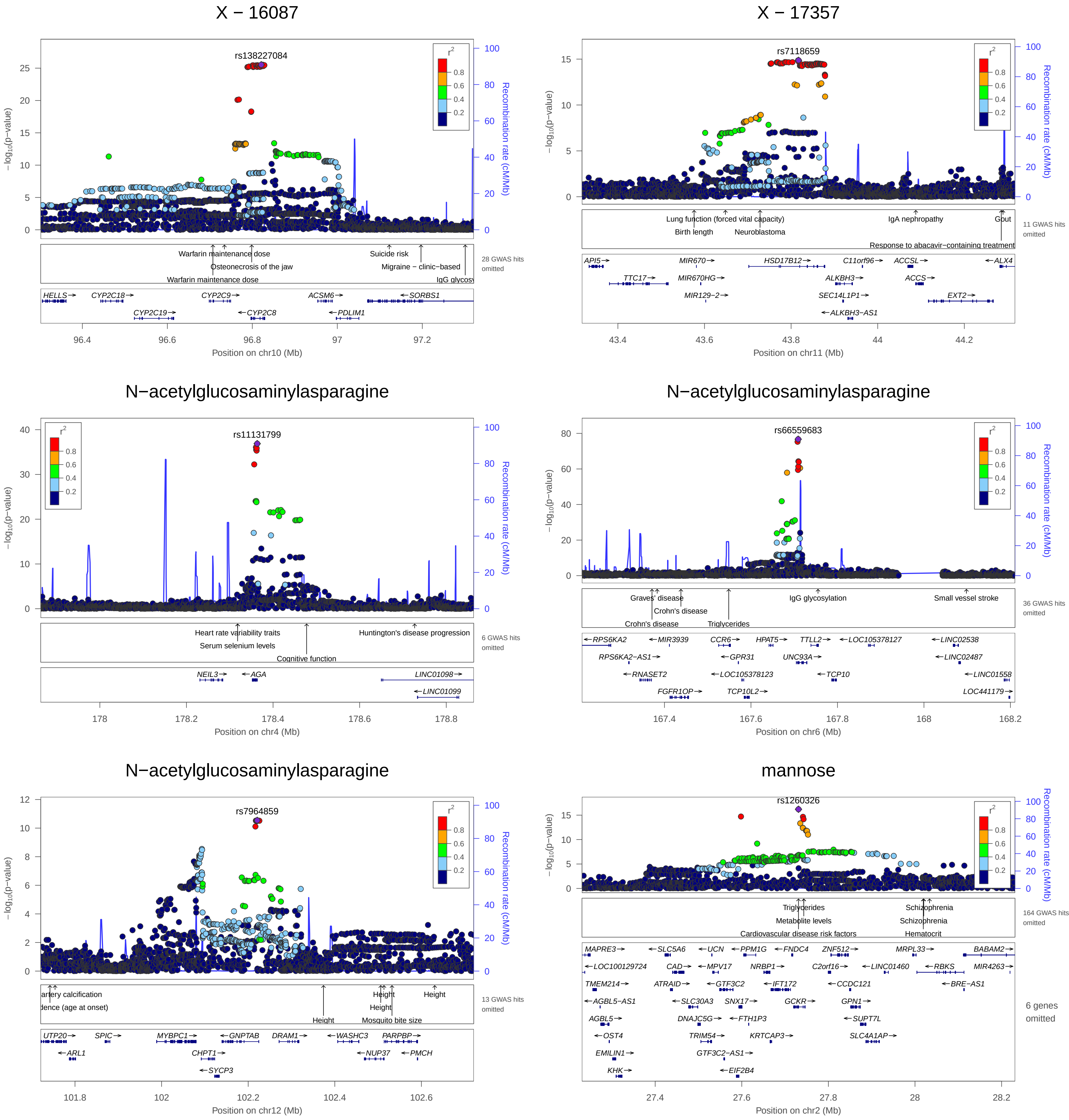

X − 16087
rs138227084
Position on chr10 (Mb)
28 GWAS hits omitted
X − 17357
rs7118659
Position on chr11 (Mb)
11 GWAS hits omitted
N−acetylglucosaminylasparagine
rs11131799
Position on chr4 (Mb)
6 GWAS hits omitted
N−acetylglucosaminylasparagine
rs66559683
Position on chr6 (Mb)
36 GWAS hits omitted
N−acetylglucosaminylasparagine
rs7964859
Position on chr12 (Mb)
13 GWAS hits omitted
mannose
rs1260326
Position on chr2 (Mb)
164 GWAS hits omitted
6 genes omitted
−log10(p−value)
Recombination rate (cM/Mb)

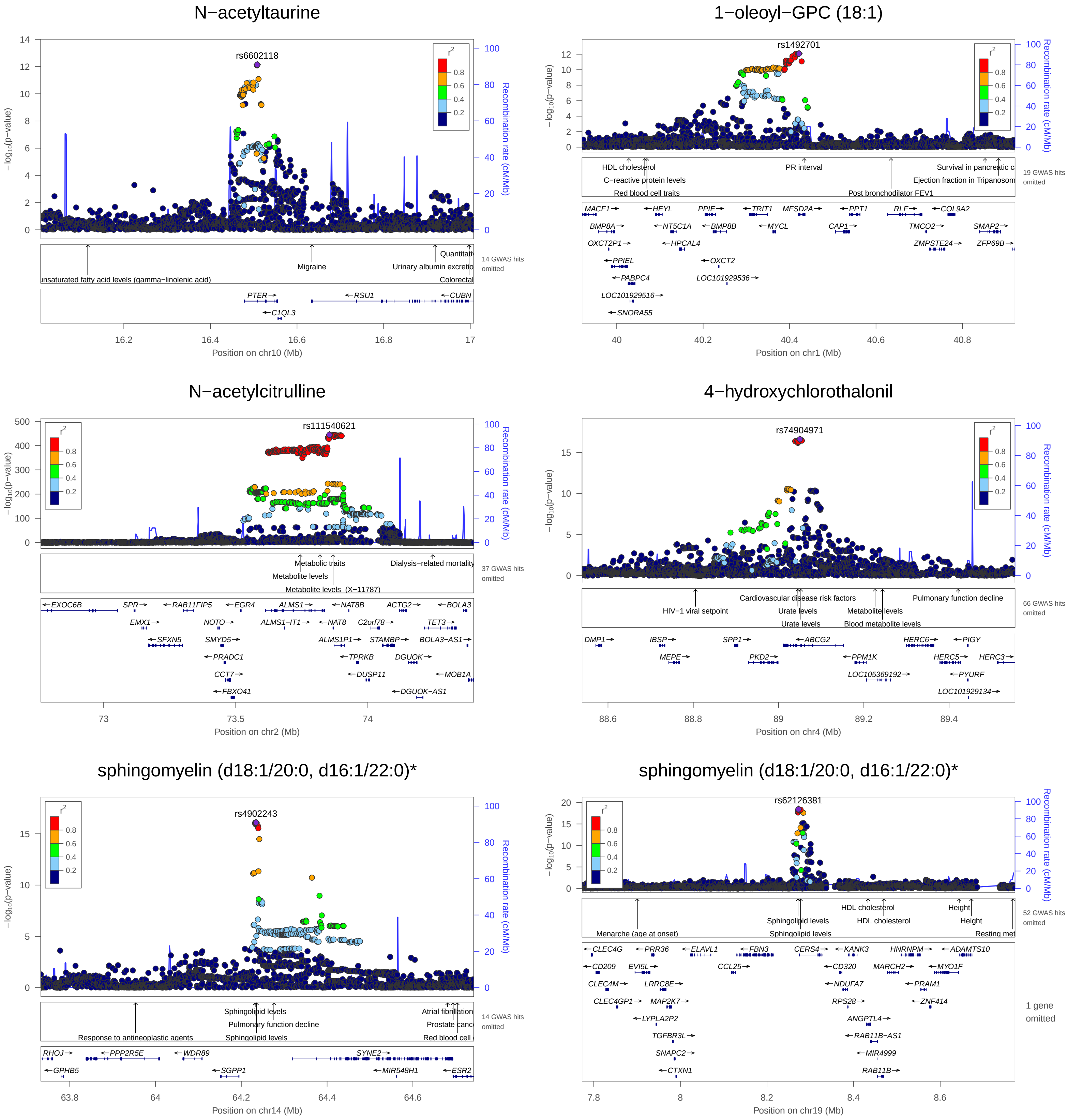

N−acetyltaurine
rs6602118
Position on chr10 (Mb)
14 GWAS hits omitted
PTER
C1QL3
RSU1
CUBN
Migraine
Urinary albumin excretio
Quantitati
Colorectal
unsaturated fatty acid levels (gamma−linolenic acid)
1−oleoyl−GPC (18:1)
rs1492701
Position on chr1 (Mb)
19 GWAS hits omitted
HDL cholesterol
C−reactive protein levels
Red blood cell traits
PR interval
Post bronchodilator FEV1
Survival in pancreatic c
Ejection fraction in Tripanosom
MACF1
BMP8A
OXCT2P1
PPIEL
PABPC4
LOC101929516
SNORA55
HEYL
NT5C1A
HPCAL4
PPIE
BMP8B
OXCT2
LOC101929536
TRIT1
MYCL
MFSD2A
CAP1
PPT1
RLF
TMCO2
ZMPSTE24
COL9A2
SMAP2
ZFP69B
N−acetylcitrulline
rs111540621
Position on chr2 (Mb)
37 GWAS hits omitted
Metabolic traits
Metabolite levels
Metabolite levels (X−11787)
Dialysis−related mortality
EXOC6B
SPR
EMX1
SFXN5
RAB11FIP5
NOTO
SMYD5
PRADC1
CCT7
FBXO41
EGR4
ALMS1
ALMS1−IT1
ALMS1P1
NAT8
NAT8B
TPRKB
DUSP11
C2orf78
STAMBP
ACTG2
DGUOK
DGUOK−AS1
TET3
BOLA3
BOLA3−AS1
MOB1A
4−hydroxychlorothalonil
rs74904971
Position on chr4 (Mb)
66 GWAS hits omitted
HIV−1 viral setpoint
Cardiovascular disease risk factors
Urate levels
Metabolite levels
Blood metabolite levels
Pulmonary function decline
DMP1
IBSP
MEPE
SPP1
PKD2
ABCG2
PPM1K
LOC105369192
HERC6
HERC5
PIGY
HERC3
PYURF
LOC101929134
sphingomyelin (d18:1/20:0, d16:1/22:0)*
rs4902243
Position on chr14 (Mb)
14 GWAS hits omitted
Response to antineoplastic agents
Sphingolipid levels
Pulmonary function decline
Atrial fibrillation
Prostate canc
Red blood cell
RHOJ
GPHB5
PPP2R5E
WDR89
SGPP1
SYNE2
MIR548H1
ESR2
sphingomyelin (d18:1/20:0, d16:1/22:0)*
rs62126381
Position on chr19 (Mb)
52 GWAS hits omitted
1 gene omitted
Menarche (age at onset)
Sphingolipid levels
HDL cholesterol
Height
Resting met
CLEC4G
CD209
CLEC4M
CLEC4GP1
PRR36
EVI5L
LRRC8E
MAP2K7
LYPLA2P2
TGFBR3L
SNAPC2
CTXN1
ELAVL1
FBN3
CCL25
CERS4
CD320
NDUFA7
RPS28
KANK3
ANGPTL4
RAB11B−AS1
MIR4999
RAB11B
HNRNPM
MARCH2
ZNF414
PRAM1
MYO1F
ADAMTS10
− log10(p−value)
Recombination rate (cM/Mb)

sphingomyelin (d18:1/20:0, d16:1/22:0)*

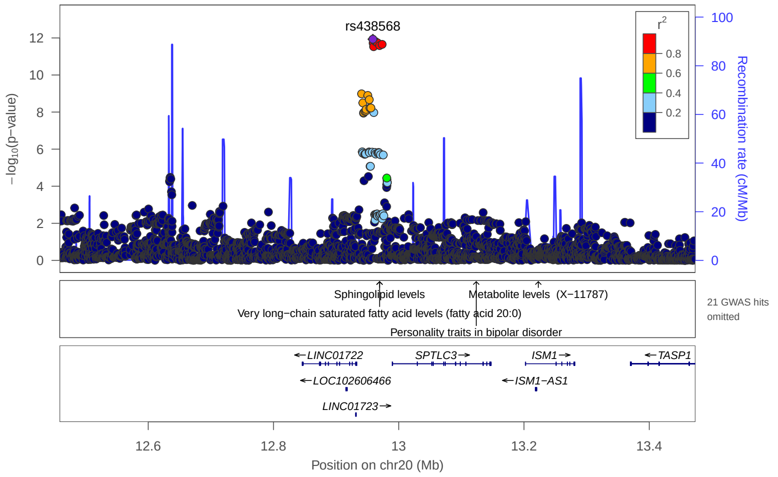

sphingomyelin (d18:1/20:1, d18:2/20:0)*

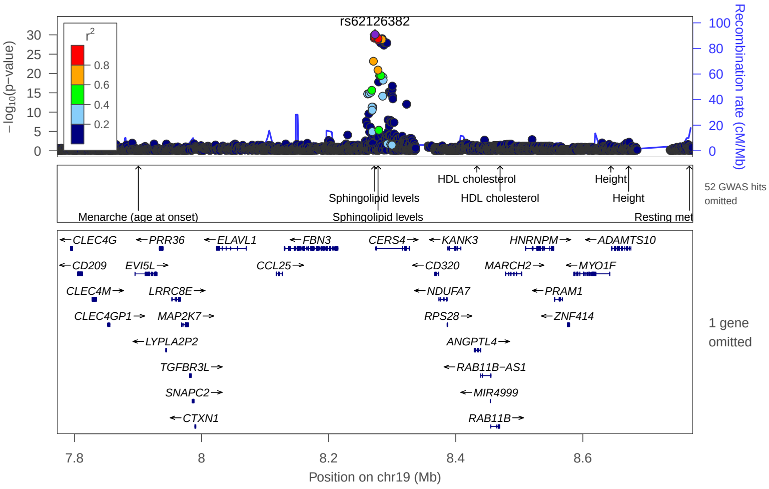

sphingomyelin (d18:1/22:1, d18:2/22:0, d16:1/24:1)*

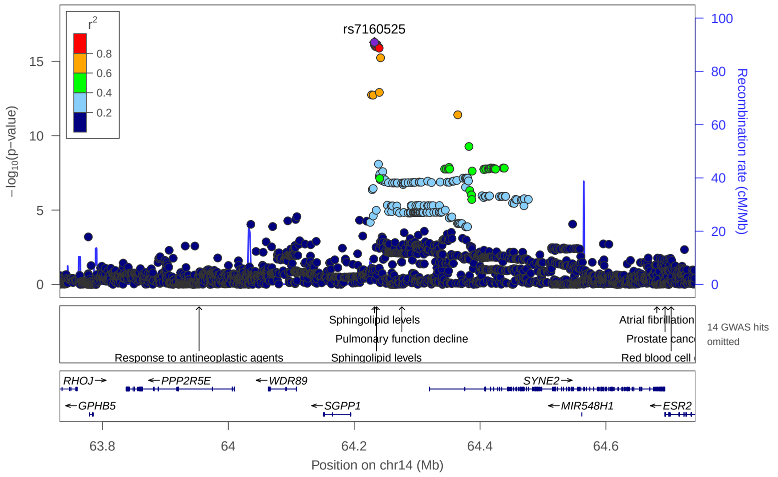

maltol sulfate

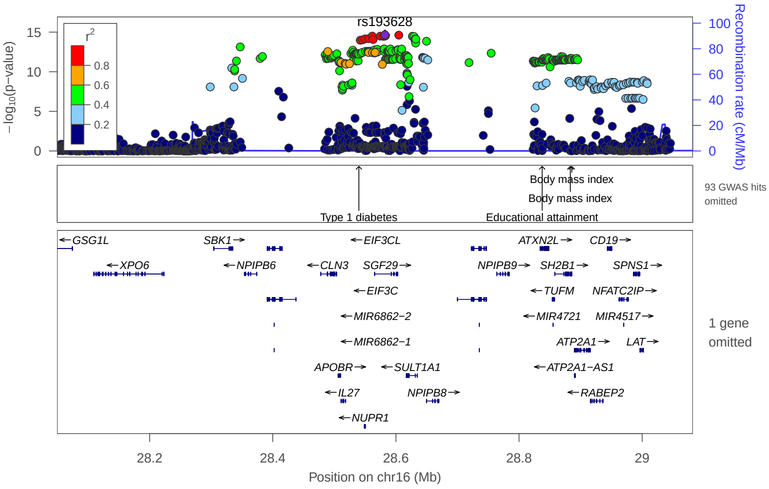

N−acetylkynurenine (2)

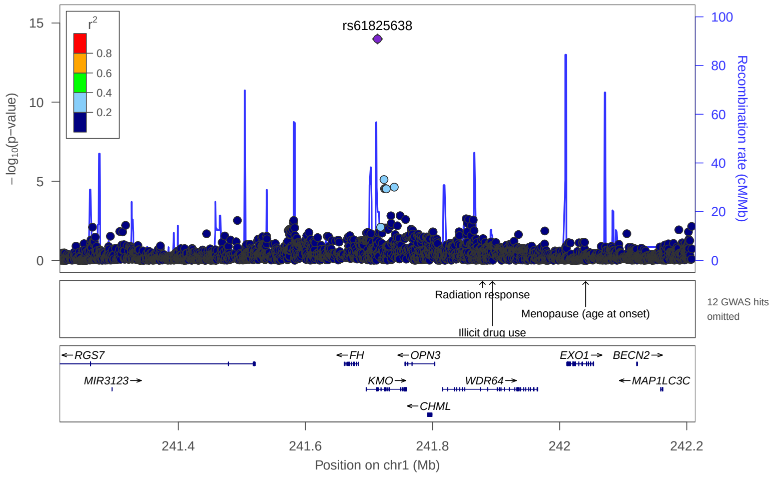

N−acetylkynurenine (2)

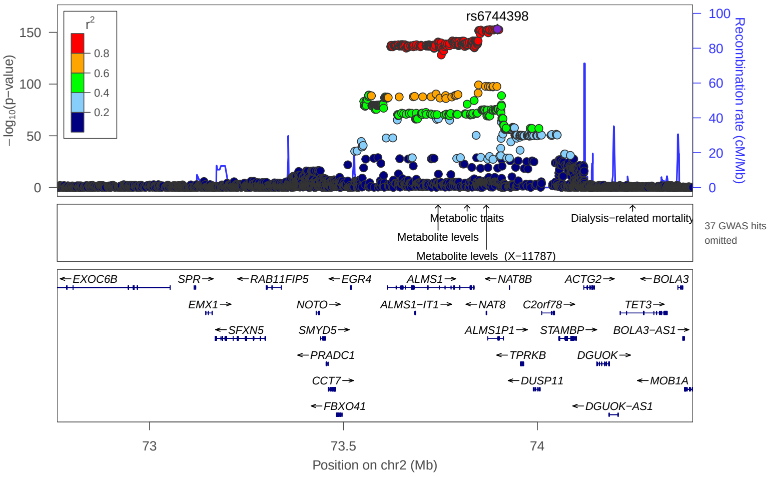

N−acetylkynurenine (2)

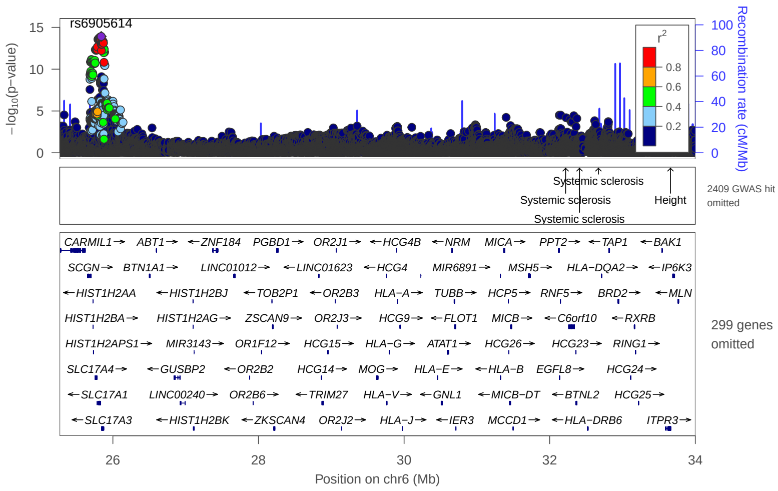

N−acetylkynurenine (2)

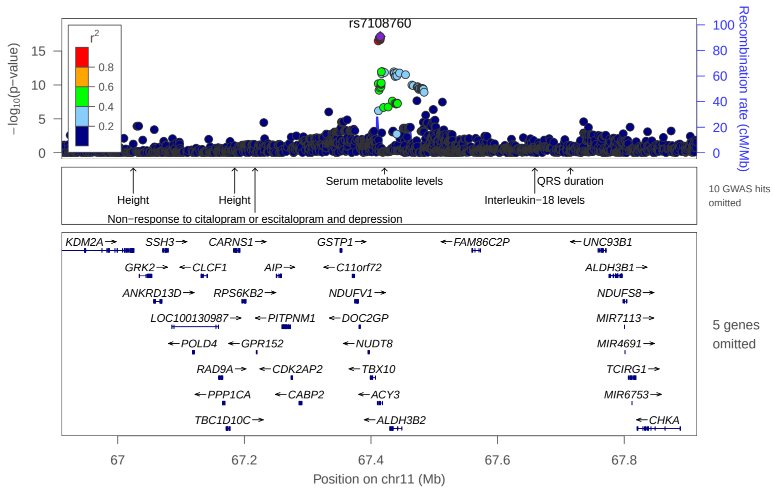

X − 23636

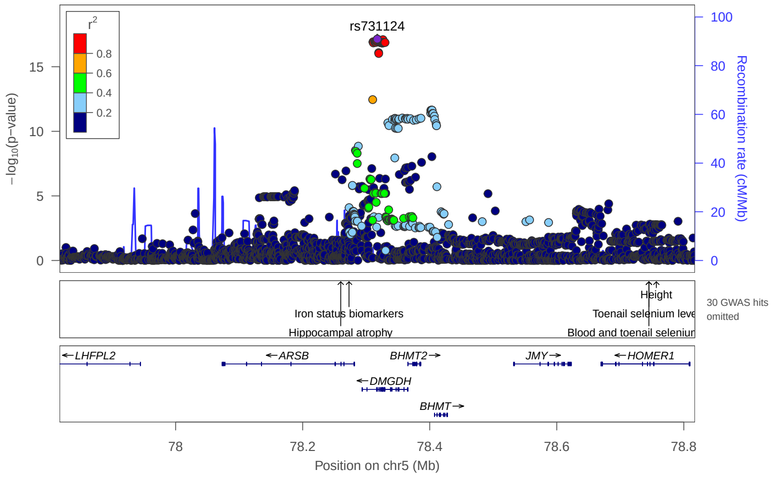

X − 23636

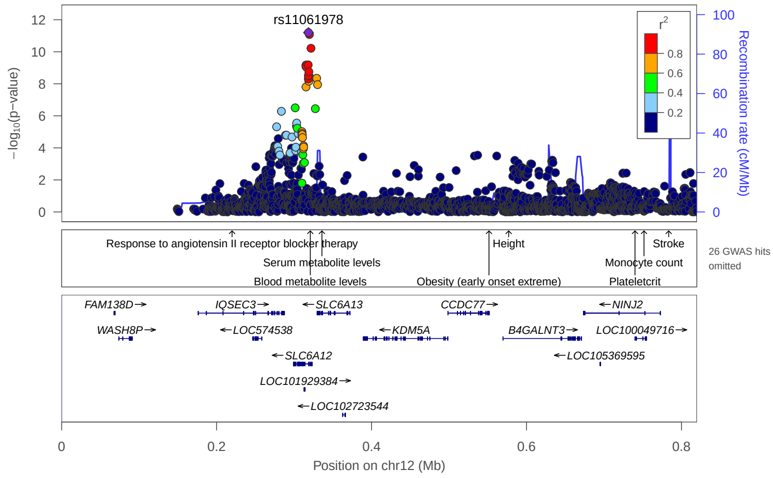

X − 23641

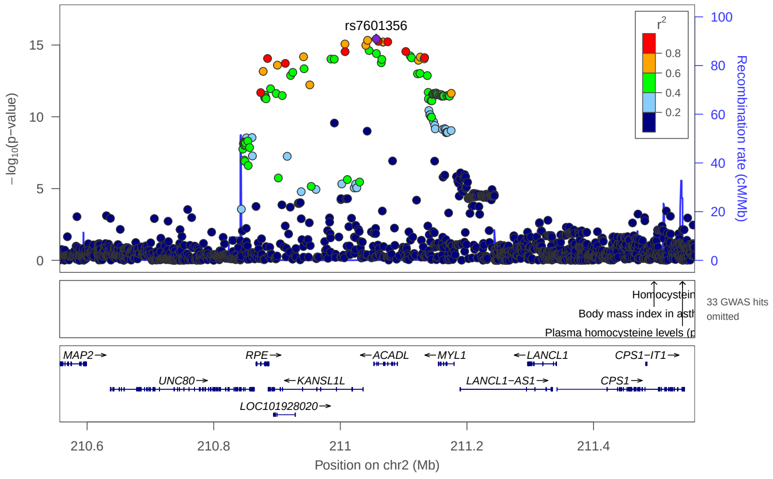

X − 23641

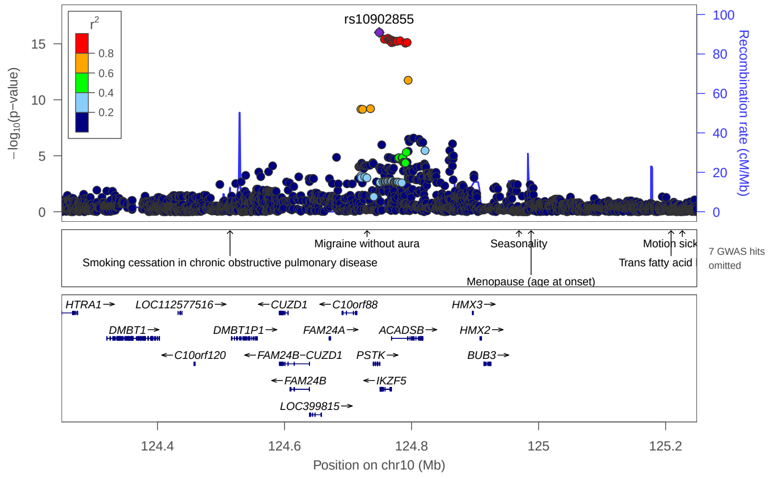

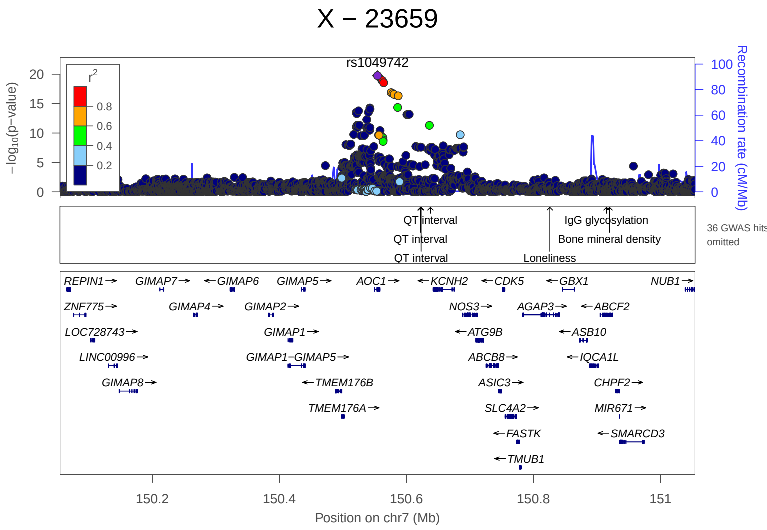
X − 23659
rs1049742
−log10(p−value)
Recombination rate (cM/Mb)
QT interval
QT interval
QT interval
IgG glycosylation
Bone mineral density
Loneliness
36 GWAS hits omitted
REPIN1 GIMAP7 GIMAP6 GIMAP5 AOC1 KCNH2 CDK5 GBX1 NUB1
ZNF775 GIMAP4 GIMAP2 NOS3 AGAP3 ABCF2
LOC728743 GIMAP1 ATG9B ASB10
LINC00996 GIMAP1−GIMAP5 ABCB8 IQCA1L
GIMAP8 TMEM176B ASIC3 CHPF2
TMEM176A SLC4A2 MIR671
FASTK SMARCD3
TMUB1
Position on chr7 (Mb)

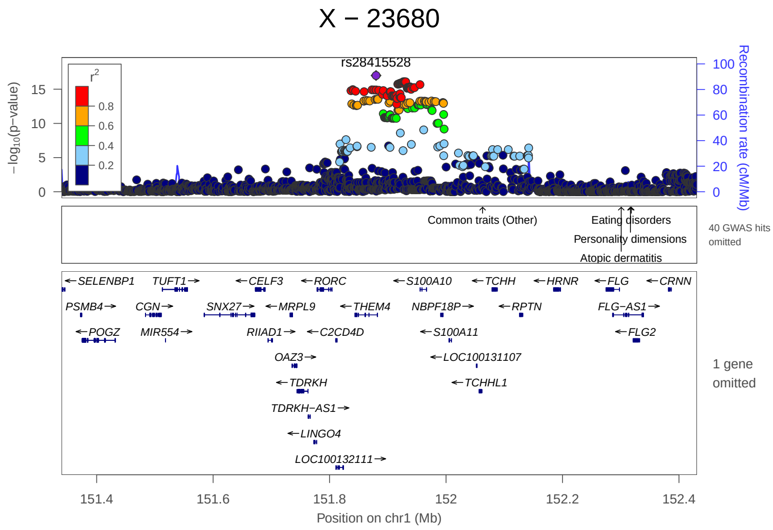
X − 23680
rs28415528
−log10(p−value)
Recombination rate (cM/Mb)
Common traits (Other)
Eating disorders
Personality dimensions
Atopic dermatitis
40 GWAS hits omitted
SELENBP1 TUFT1 CELF3 RORC S100A10 TCHH HRNR FLG CRNN
PSMB4 CGN SNX27 MRPL9 THEM4 NBPF18P RPTN FLG−AS1
POGZ MIR554 RIIAD1 C2CD4D S100A11 FLG2
OAZ3 LOC100131107
TDRKH TCHHL1
TDRKH−AS1
LINGO4
LOC100132111
1 gene omitted
Position on chr1 (Mb)

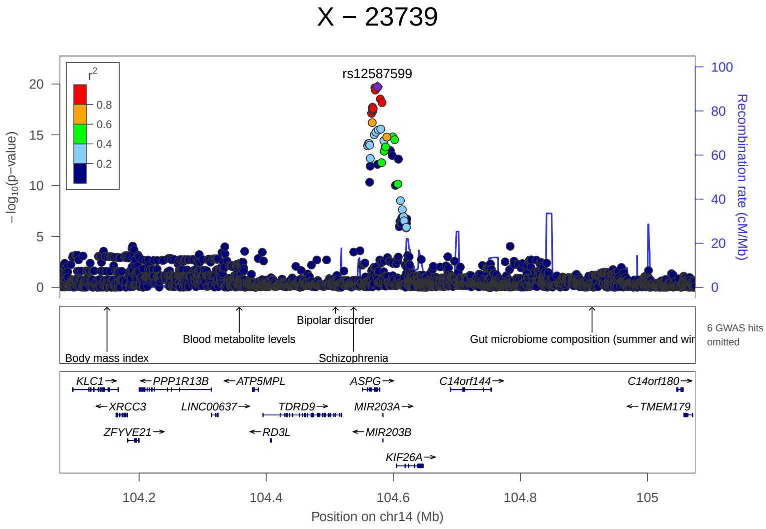
X − 23739
rs12587599
−log10(p−value)
Recombination rate (cM/Mb)
Bipolar disorder
Blood metabolite levels
Gut microbiome composition (summer and wir
Body mass index
Schizophrenia
6 GWAS hits omitted
KLC1 PPP1R13B ATP5MPL ASPG C14orf144 C14orf180
XRCC3 LINC00637 TDRD9 MIR203A TMEM179
ZFYVE21 RD3L MIR203B
KIF26A
Position on chr14 (Mb)

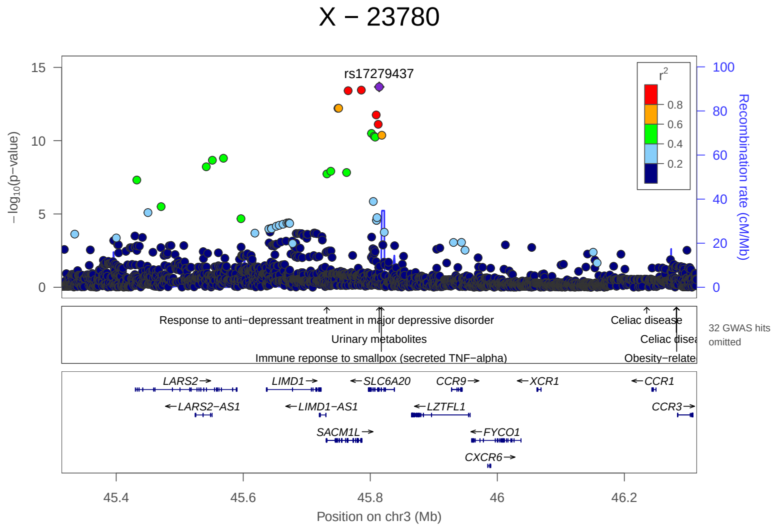
X − 23780
rs17279437
−log10(p−value)
Recombination rate (cM/Mb)
Response to anti−depressant treatment in major depressive disorder
Urinary metabolites
Immune reponse to smallpox (secreted TNF−alpha)
Celiac disease
Celiac disea
Obesity−relate
32 GWAS hits omitted
LARS2 LIMD1 SLC6A20 CCR9 XCR1 CCR1
LARS2−AS1 LIMD1−AS1 LZTFL1 CCR3
SACM1L FYCO1
CXCR6
Position on chr3 (Mb)

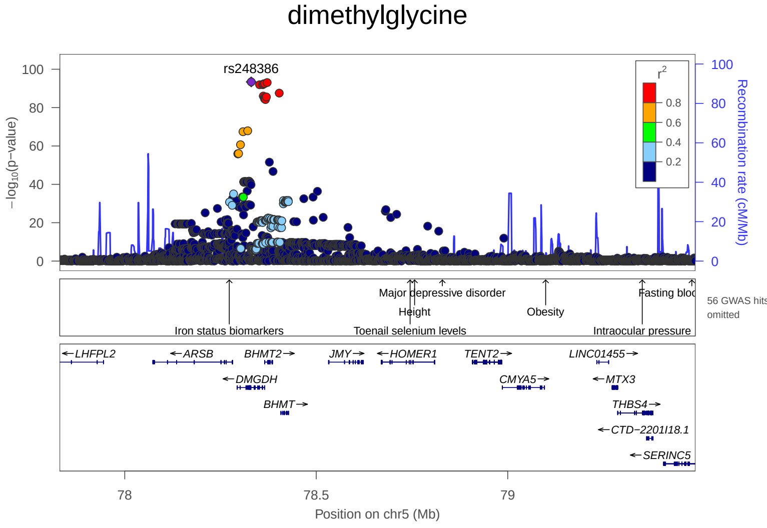
dimethylglycine
rs248386
−log10(p−value)
Recombination rate (cM/Mb)
Major depressive disorder
Height
Obesity
Fasting bloo
Iron status biomarkers
Toenail selenium levels
Intraocular pressure
56 GWAS hits omitted
LHFPL2 ARSB BHMT2 JMY HOMER1 TENT2 LINC01455
DMGDH CMYA5 MTX3
BHMT THBS4
CTD−2201I18.1
SERINC5
Position on chr5 (Mb)

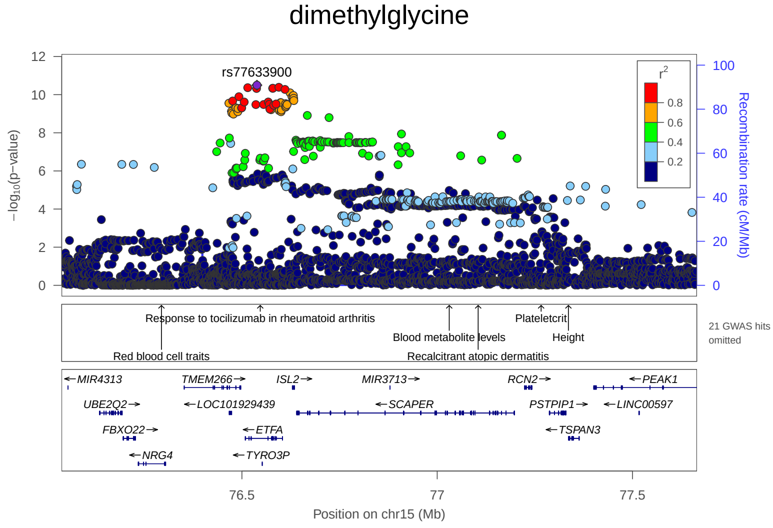
dimethylglycine
rs77633900
−log10(p−value)
Recombination rate (cM/Mb)
Response to tocilizumab in rheumatoid arthritis
Plateletcrit
Blood metabolite levels
Height
Red blood cell traits
Recalcitrant atopic dermatitis
21 GWAS hits omitted
MIR4313 TMEM266 ISL2 MIR3713 RCN2 PEAK1
UBE2Q2 LOC101929439 SCAPER PSTPIP1 LINC00597
FBXO22 ETFA TSPAN3
NRG4 TYRO3P
Position on chr15 (Mb)

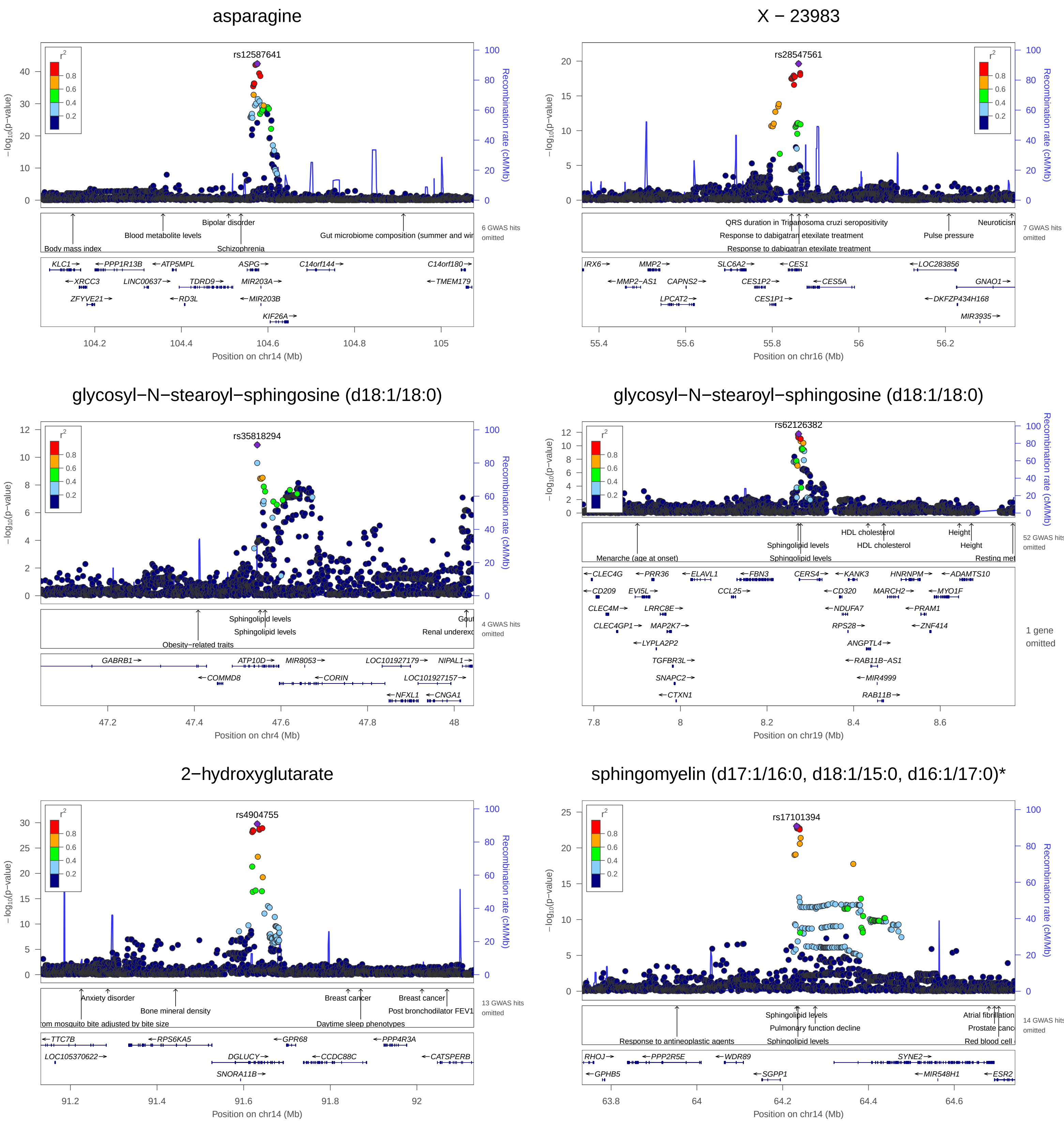

asparagine
rs12587641
−log10(p−value)
Recombination rate (cM/Mb)
Body mass index
Blood metabolite levels
Bipolar disorder
Schizophrenia
Gut microbiome composition (summer and wir
6 GWAS hits omitted
KLC1
PPP1R13B
ATP5MPL
ASPG
C14orf144
C14orf180
XRCC3
LINC00637
TDRD9
MIR203A
TMEM179
ZFYVE21
RD3L
MIR203B
KIF26A
Position on chr14 (Mb)
X − 23983
rs28547561
QRS duration in Trypanosoma cruzi seropositivity
Response to dabigatran etexilate treatment
Response to dabigatran etexilate treatment
Pulse pressure
Neuroticism
7 GWAS hits omitted
IRX6
MMP2
SLC6A2
CES1
LOC283856
MMP2−AS1
CAPNS2
CES1P2
CES5A
GNAO1
LPCAT2
CES1P1
DKFZP434H168
MIR3935
Position on chr16 (Mb)
glycosyl−N−stearoyl−sphingosine (d18:1/18:0)
rs35818294
Sphingolipid levels
Sphingolipid levels
Obesity−related traits
Gout
Renal underexc
4 GWAS hits omitted
GABRB1
ATP10D
MIR8053
LOC101927179
NIPAL1
COMMD8
CORIN
LOC101927157
NFXL1
CNGA1
Position on chr4 (Mb)
glycosyl−N−stearoyl−sphingosine (d18:1/18:0)
rs62126382
Menarche (age at onset)
Sphingolipid levels
Sphingolipid levels
HDL cholesterol
HDL cholesterol
Height
Height
Resting met
52 GWAS hits omitted
CLEC4G
PRR36
ELAVL1
FBN3
CERS4
KANK3
HNRNPM
ADAMTS10
CD209
EVI5L
CCL25
CD320
MARCH2
MYO1F
CLEC4M
LRRC8E
NDUFA7
PRAM1
CLEC4GP1
MAP2K7
RPS28
ZNF414
LYPLA2P2
ANGPTL4
TGFBR3L
RAB11B−AS1
SNAPC2
MIR4999
CTXN1
RAB11B
1 gene omitted
Position on chr19 (Mb)
2−hydroxyglutarate
rs4904755
Anxiety disorder
Bone mineral density
om mosquito bite adjusted by bite size
Breast cancer
Daytime sleep phenotypes
Breast cancer
Post bronchodilator FEV1
13 GWAS hits omitted
TTC7B
RPS6KA5
GPR68
PPP4R3A
LOC105370622
DGLUCY
CCDC88C
CATSPERB
SNORA11B
Position on chr14 (Mb)
sphingomyelin (d17:1/16:0, d18:1/15:0, d16:1/17:0)*
rs17101394
Response to antineoplastic agents
Sphingolipid levels
Pulmonary function decline
Sphingolipid levels
Atrial fibrillation
Prostate canc
Red blood cell
14 GWAS hits omitted
RHOJ
PPP2R5E
WDR89
SYNE2
GPHB5
SGPP1
MIR548H1
ESR2
Position on chr14 (Mb)

1−stearoyl−2−linoleoyl−GPE (18:0/18:2)*

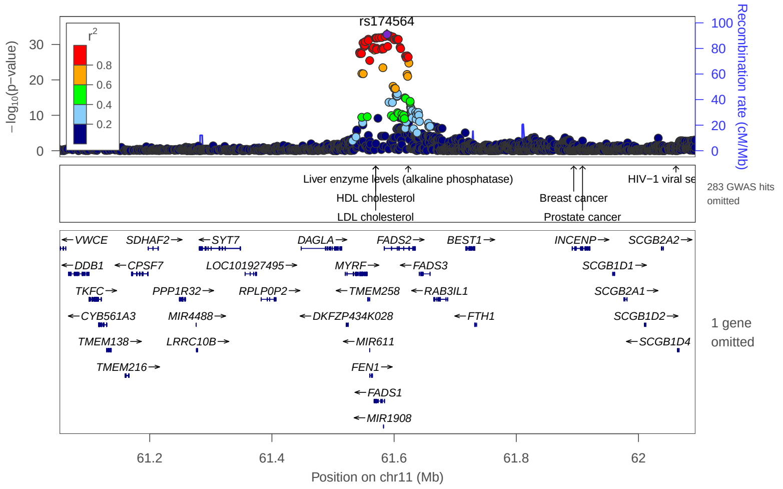

1−stearoyl−2−linoleoyl−GPE (18:0/18:2)*

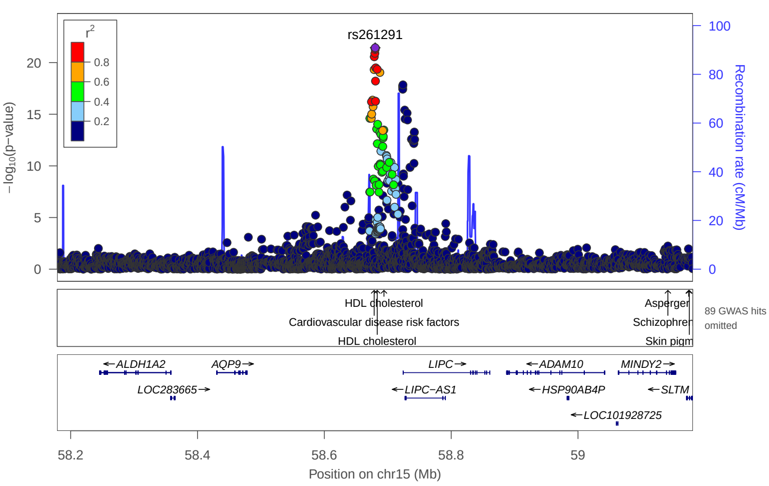

1−stearoyl−2−arachidonoyl−GPE (18:0/20:4)

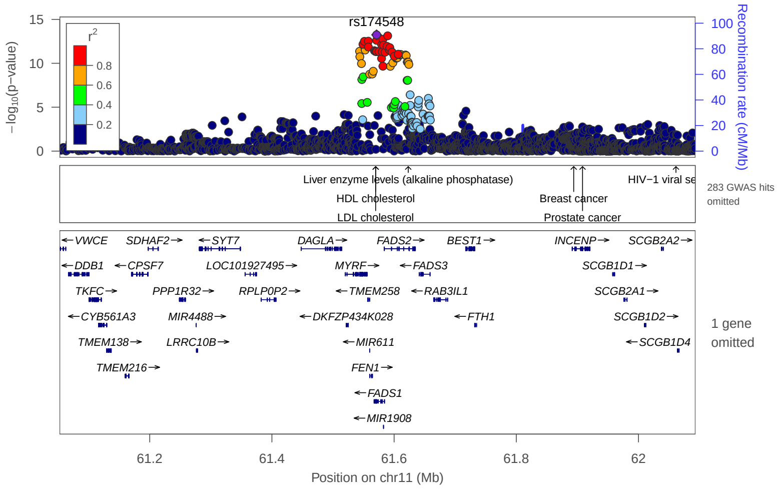

1−stearoyl−2−arachidonoyl−GPE (18:0/20:4)

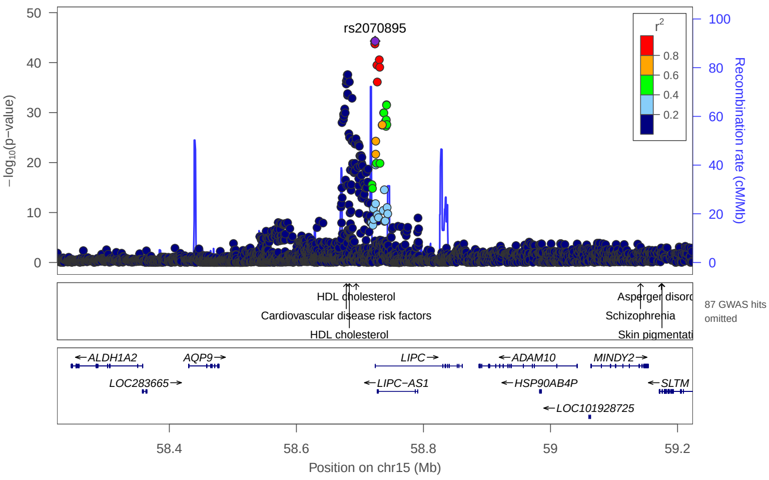

1−stearoyl−2−arachidonoyl−GPI (18:0/20:4)

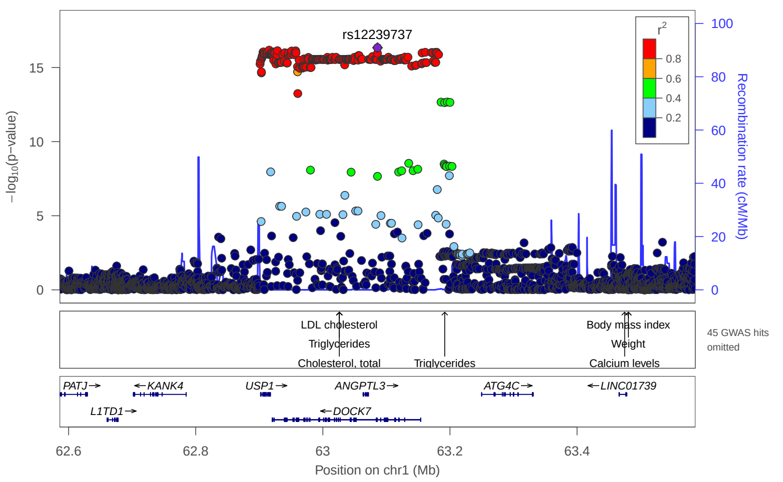

1−stearoyl−2−arachidonoyl−GPI (18:0/20:4)

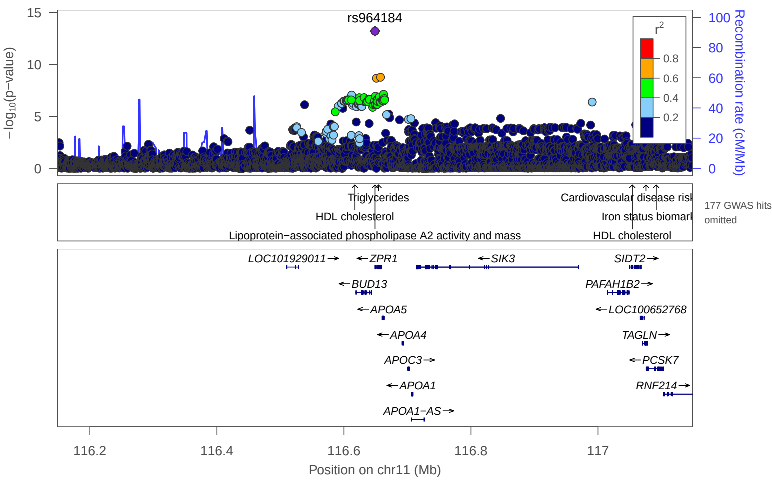

1−stearoyl−2−arachidonoyl−GPI (18:0/20:4)

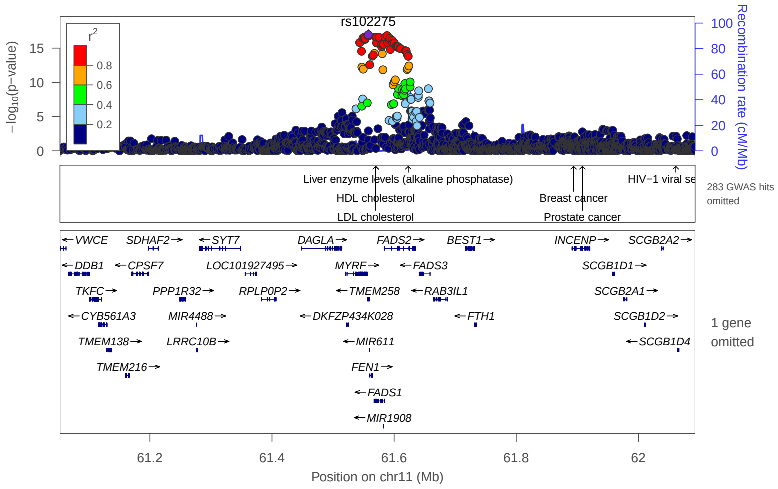

1−stearoyl−2−arachidonoyl−GPI (18:0/20:4)

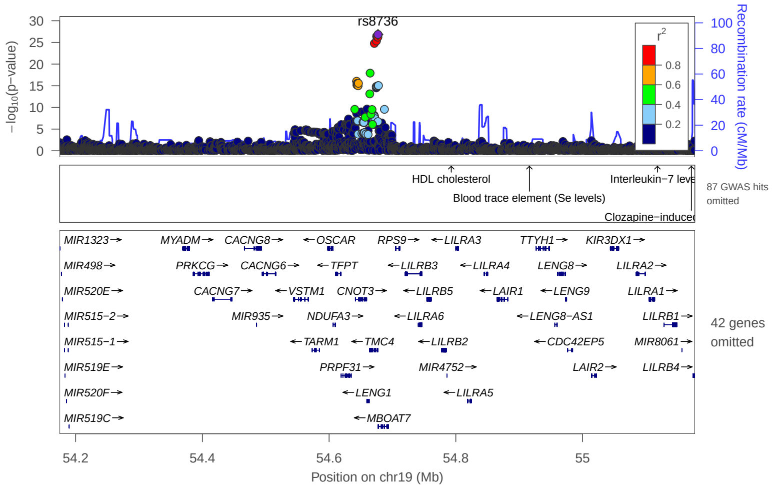

1−palmitoyl−2−linoleoyl−GPI (16:0/18:2)

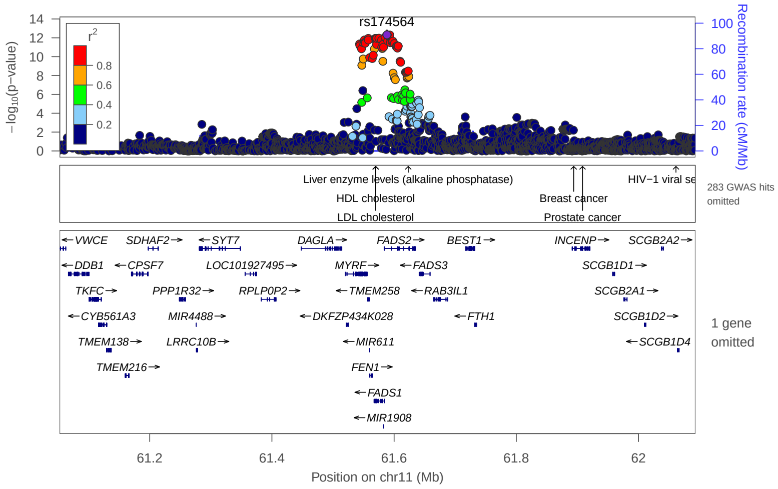

1−palmitoyl−2−linoleoyl−GPI (16:0/18:2)

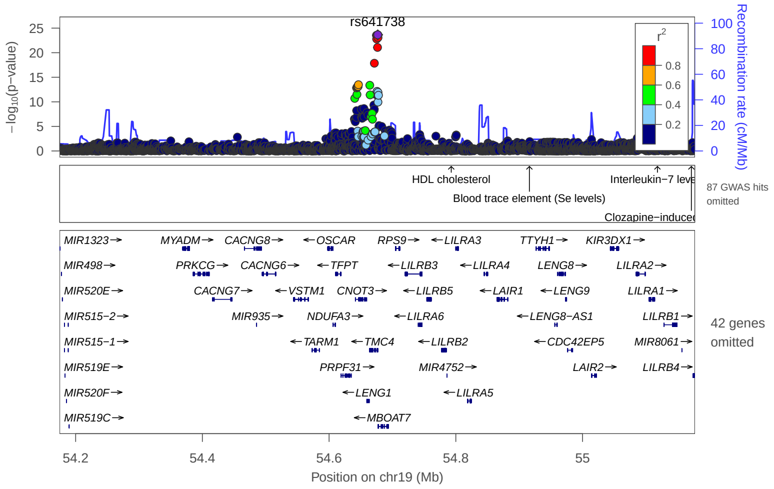

1−stearoyl−2−linoleoyl−GPC (18:0/18:2)*

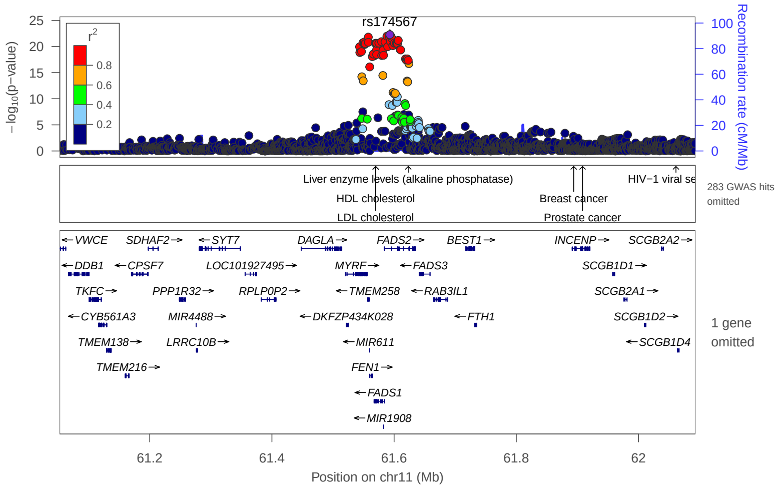

1−palmitoyl−2−dihomo−linolenoyl−GPC (16:0/20:3n3 or 6)*

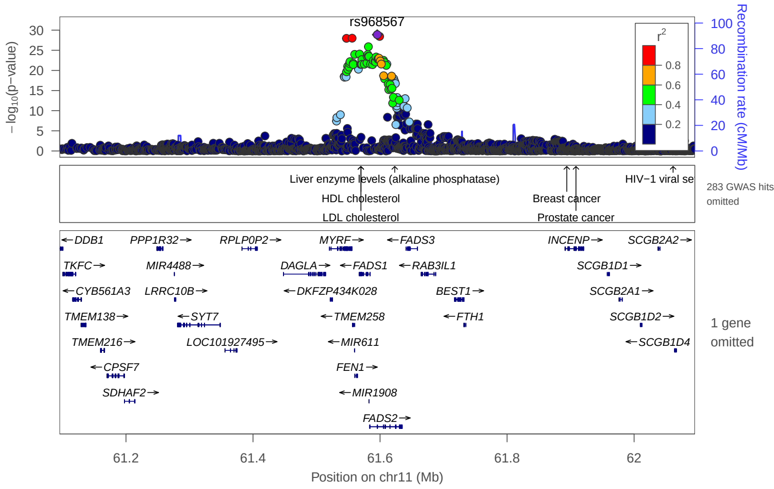

1−palmitoyl−2−dihomo−linolenoyl−GPC (16:0/20:3n3 or 6)*

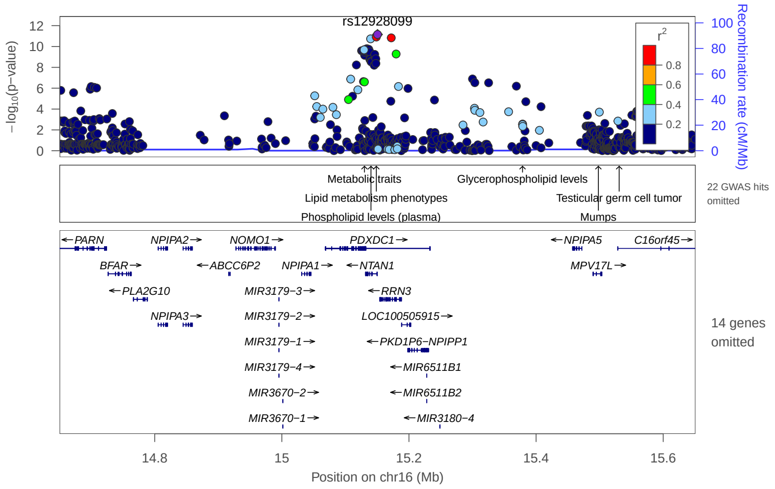

1−palmitoyl−2−arachidonoyl−GPC (16:0/20:4n6)

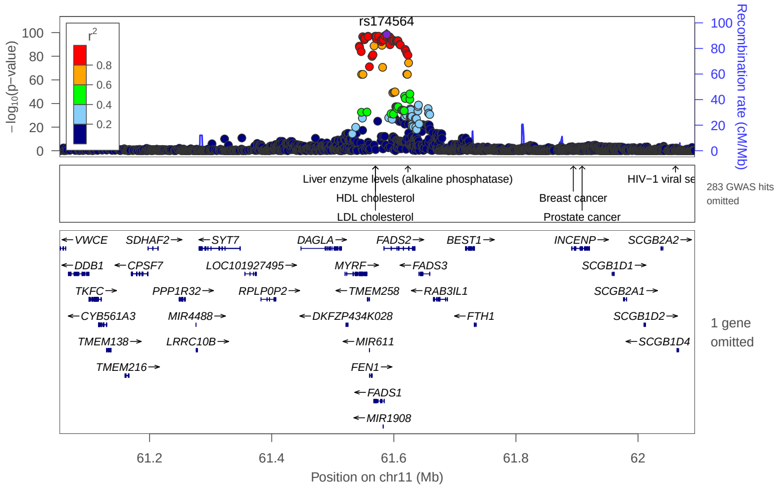

1−palmitoyl−2−arachidonoyl−GPE (16:0/20:4)*

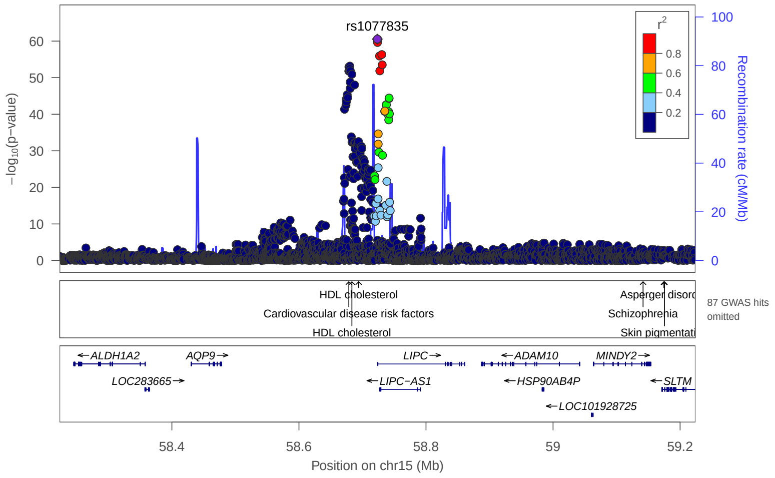

1−palmitoyl−2−docosahexaenoyl−GPE (16:0/22:6)*

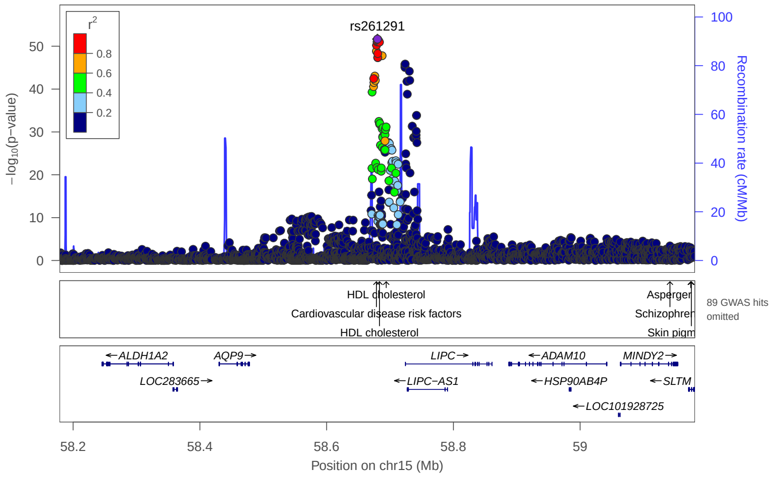

1−stearoyl−2−docosahexaenoyl−GPE (18:0/22:6)*

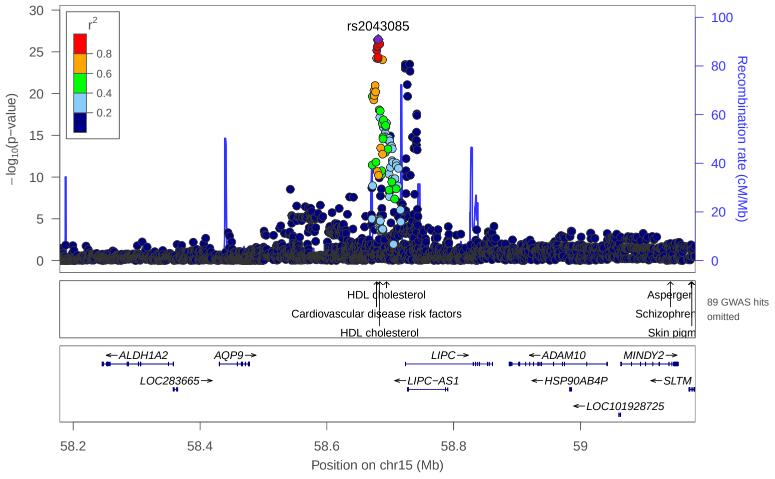

1−stearoyl−2−linoleoyl−GPI (18:0/18:2)

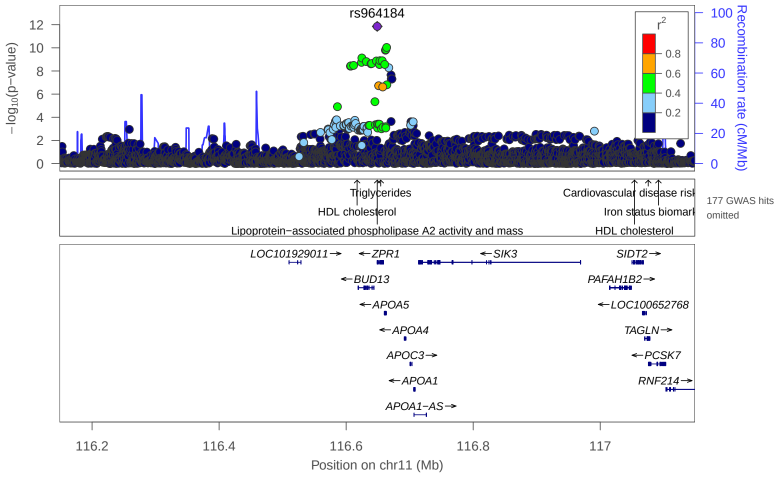

1−stearoyl−2−linoleoyl−GPI (18:0/18:2)

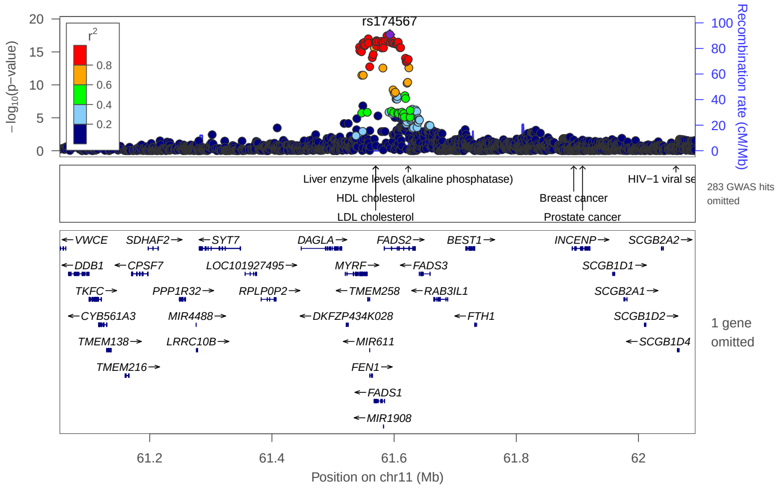

1−stearoyl−2−linoleoyl−GPI (18:0/18:2)

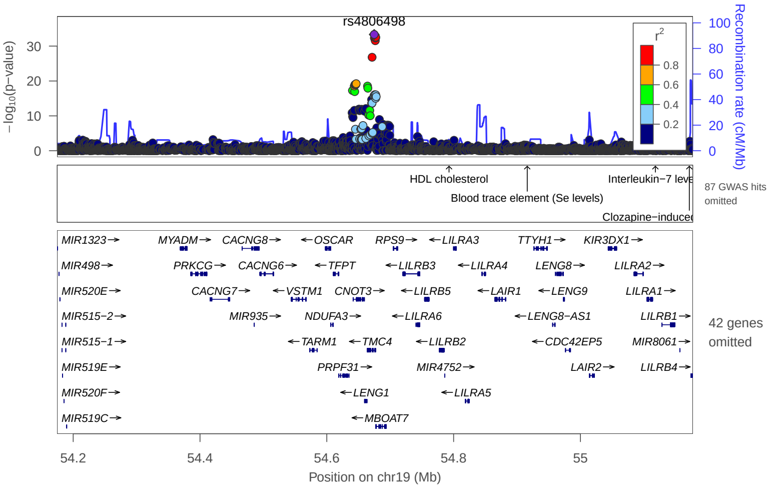

1−(1−enyl−palmitoyl)−GPC (P−16:0)*

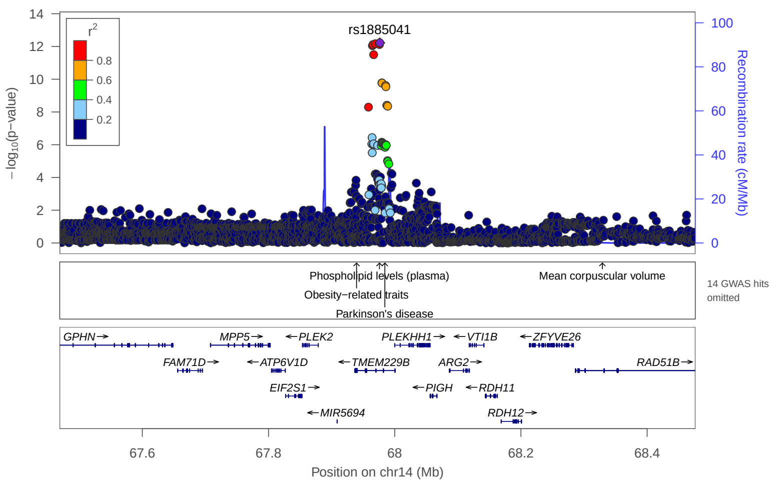

1−(1−enyl−stearoyl)−2−arachidonoyl−GPE (P−18:0/20:4)*

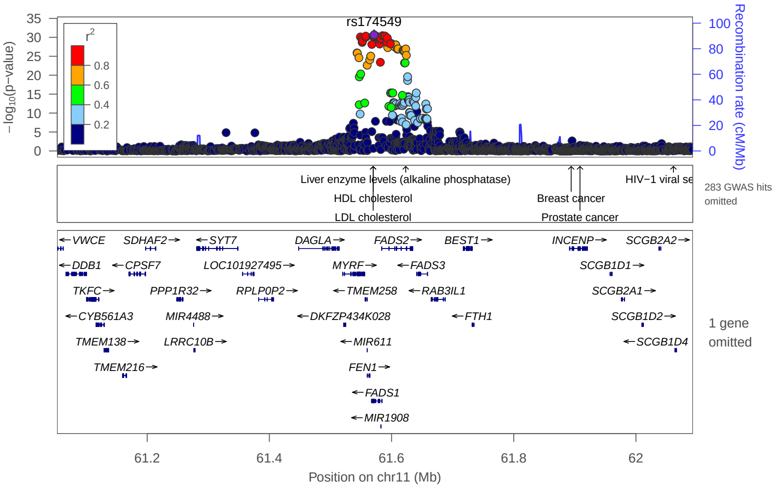

1−(1−enyl−palmitoyl)−2−oleoyl−GPC (P−16:0/18:1)*

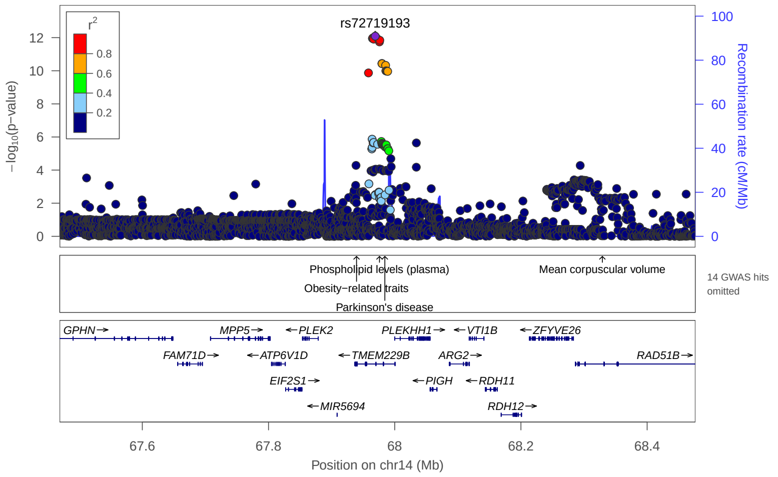

sphingomyelin (d18:1/21:0, d17:1/22:0, d16:1/23:0)*

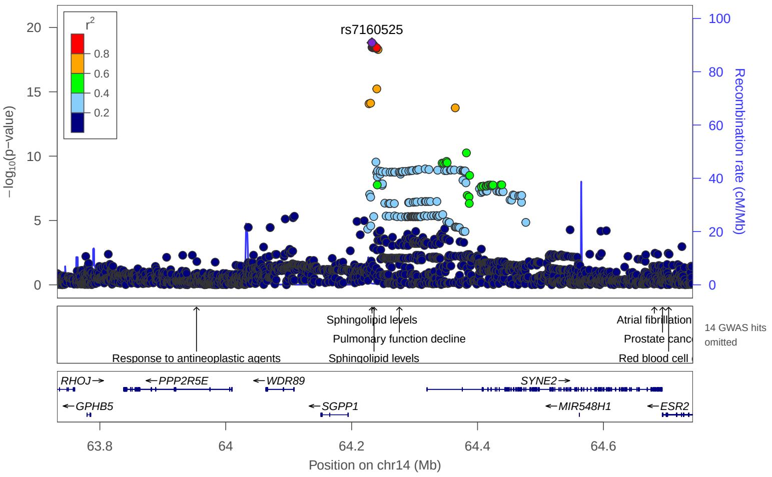

sphingomyelin (d18:1/21:0, d17:1/22:0, d16:1/23:0)*

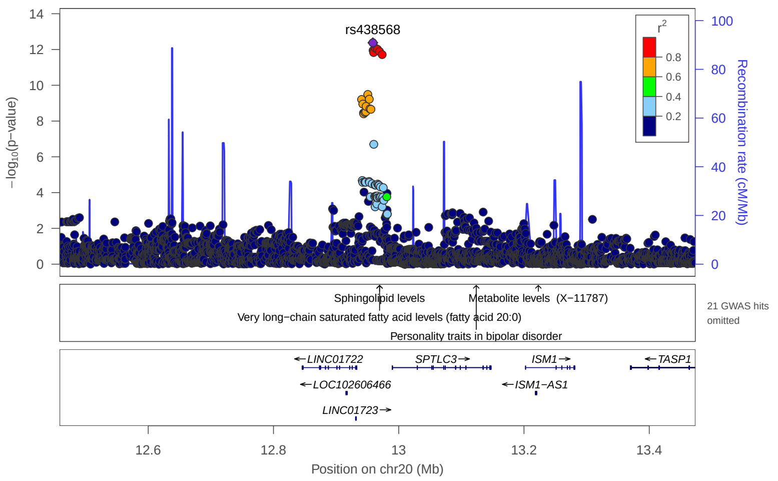

X − 24337

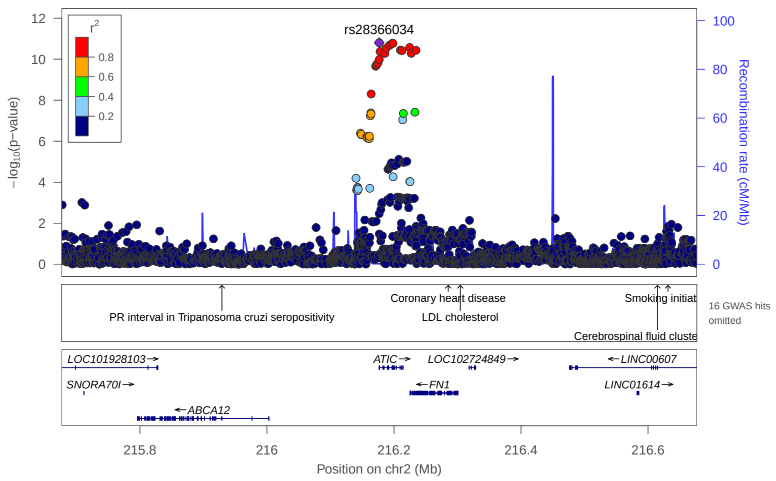

2'−deoxyuridine

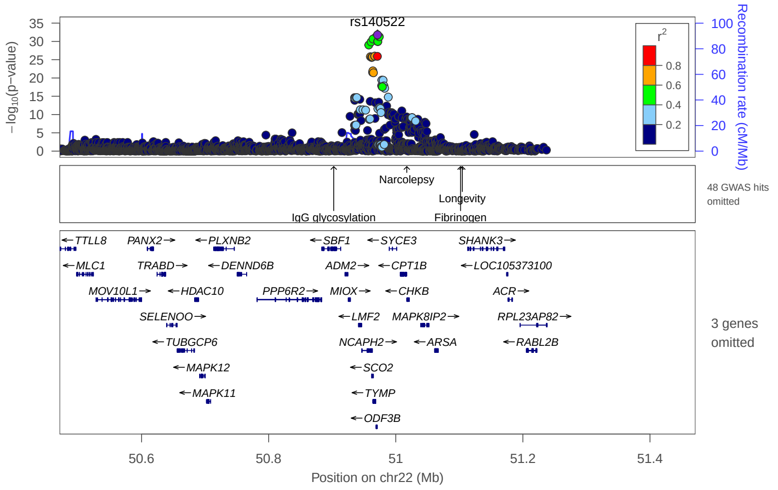

1,2−dilinoleoyl−GPC (18:2/18:2)

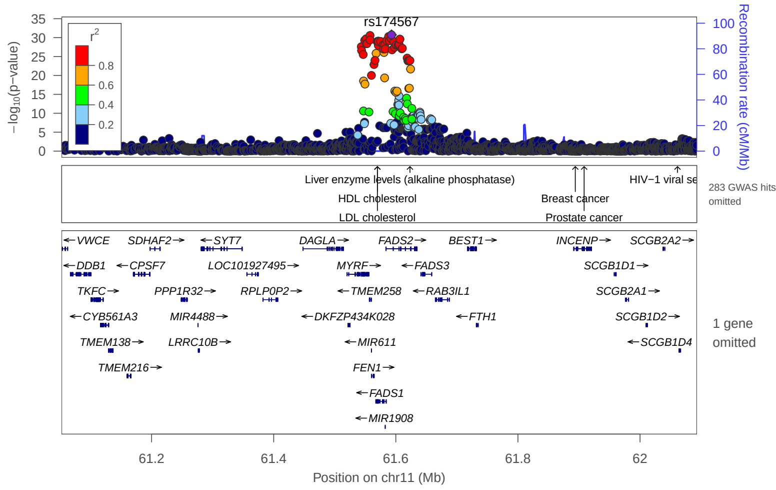

N−palmitoyl−sphinganine (d18:0/16:0)

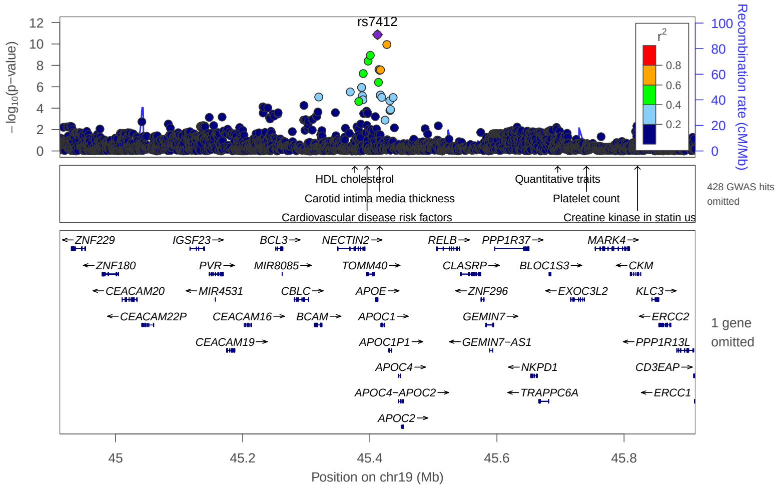

1−stearoyl−2−docosahexaenoyl−GPC (18:0/22:6)

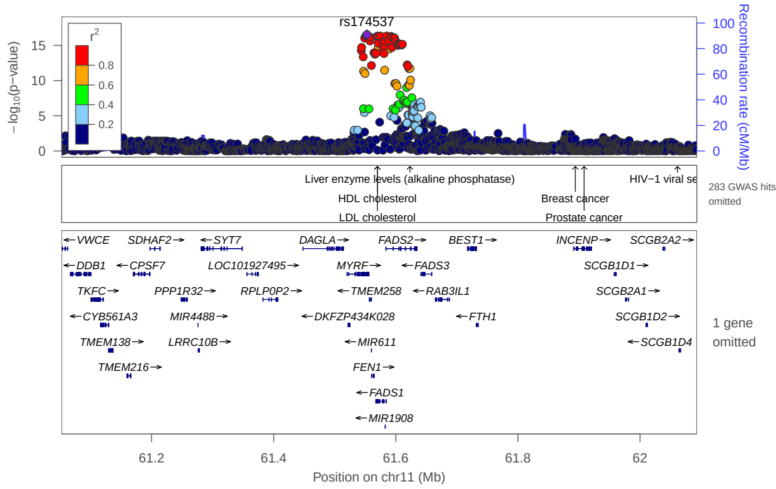

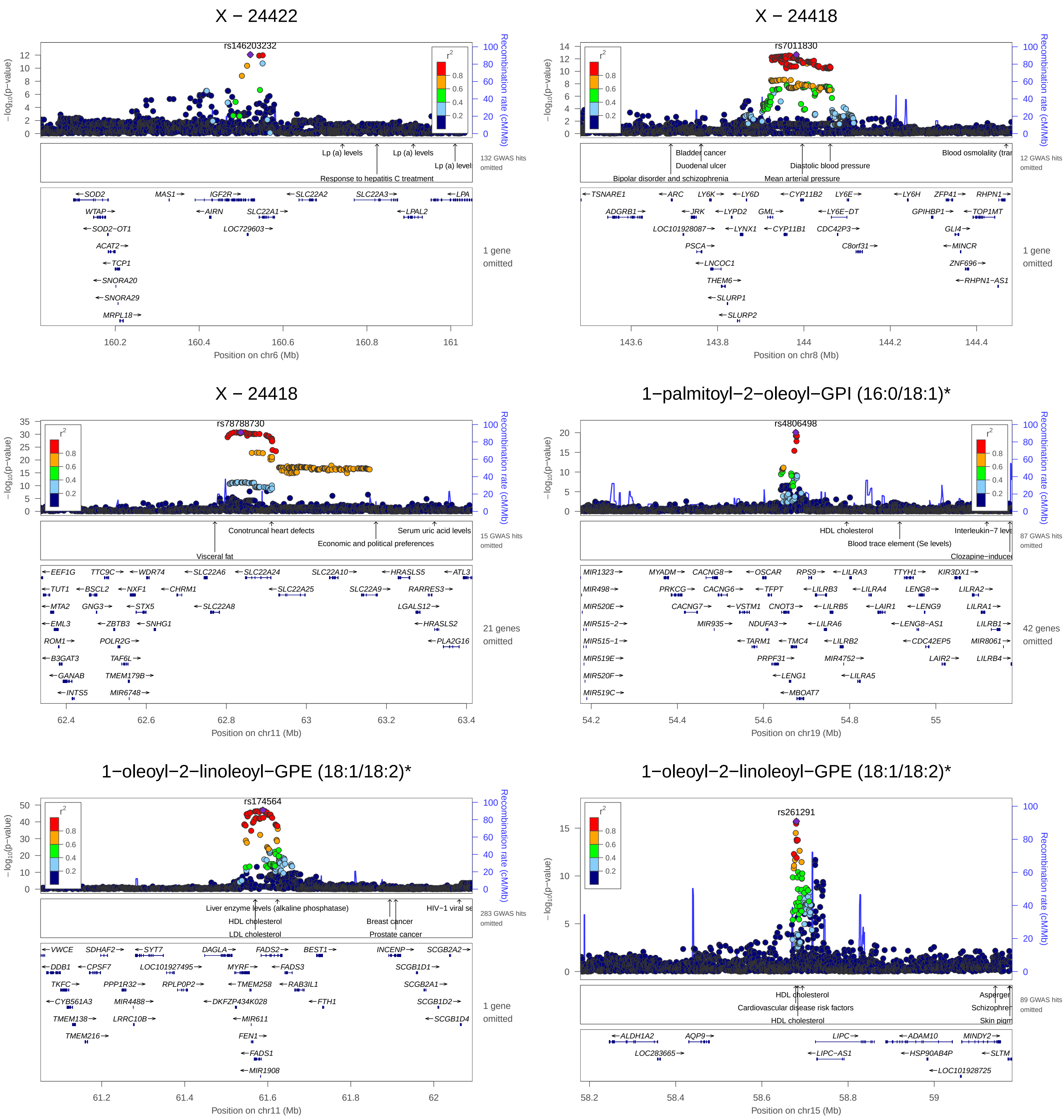

X − 24422
rs146203232
Lp (a) levels
Response to hepatitis C treatment
132 GWAS hits omitted
SOD2 WTAP SOD2−OT1 ACAT2 TCP1 SNORA20 SNORA29 MRPL18 MAS1 IGF2R AIRN LOC729603 SLC22A1 SLC22A2 SLC22A3 LPAL2 LPA
1 gene omitted
Position on chr6 (Mb)
X − 24418
rs7011830
Bladder cancer
Duodenal ulcer
Bipolar disorder and schizophrenia
Diastolic blood pressure
Mean arterial pressure
Blood osmolality (tra
12 GWAS hits omitted
TSNARE1 ADGRB1 ARC LOC101928087 JRK PSCA LY6K LYPD2 LYNX1 LNCOC1 THEM6 SLURP1 SLURP2 LY6D GML CYP11B1 CYP11B2 LY6E LY6E−DT CDC42P3 C8orf31 LY6H GPIHBP1 ZFP41 GLI4 MINCR ZNF696 RHPN1 TOP1MT RHPN1−AS1
1 gene omitted
Position on chr8 (Mb)
X − 24418
rs78788730
Conotruncal heart defects
Visceral fat
Economic and political preferences
Serum uric acid levels
15 GWAS hits omitted
EEF1G TUT1 MTA2 EML3 ROM1 B3GAT3 GANAB INTS5 TTC9C BSCL2 GNG3 ZBTB3 POLR2G TAF6L TMEM179B MIR6748 WDR74 NXF1 STX5 SNHG1 CHRM1 SLC22A6 SLC22A8 SLC22A24 SLC22A25 SLC22A10 SLC22A9 HRASLS5 RARRES3 LGALS12 HRASLS2 PLA2G16 ATL3
21 genes omitted
Position on chr11 (Mb)
1−palmitoyl−2−oleoyl−GPI (16:0/18:1)*
rs4806498
HDL cholesterol
Blood trace element (Se levels)
Interleukin−7 leve
Clozapine−induce
87 GWAS hits omitted
MIR1323 MIR498 MIR520E MIR515−2 MIR515−1 MIR519E MIR520F MIR519C MYADM PRKCG CACNG7 CACNG8 CACNG6 MIR935 VSTM1 OSCAR TFPT CNOT3 NDUFA3 TARM1 PRPF31 LENG1 MBOAT7 TMC4 RPS9 LILRB3 LILRB5 LILRA6 LILRB2 MIR4752 LILRA3 LILRA4 LILRA5 LAIR1 TTYH1 LENG8 LENG9 LENG8−AS1 CDC42EP5 LAIR2 KIR3DX1 LILRA2 LILRA1 LILRB1 MIR8061 LILRB4
42 genes omitted
Position on chr19 (Mb)
1−oleoyl−2−linoleoyl−GPE (18:1/18:2)*
rs174564
Liver enzyme levels (alkaline phosphatase)
HDL cholesterol
LDL cholesterol
Breast cancer
Prostate cancer
HIV−1 viral se
283 GWAS hits omitted
VWCE DDB1 TKFC CYB561A3 TMEM138 TMEM216 SDHAF2 CPSF7 PPP1R32 MIR4488 LRRC10B SYT7 LOC101927495 RPLP0P2 DAGLA MYRF TMEM258 DKFZP434K028 MIR611 FEN1 FADS1 MIR1908 FADS2 FADS3 RAB3IL1 BEST1 FTH1 INCENP SCGB1D1 SCGB2A1 SCGB1D2 SCGB2A2 SCGB1D4
1 gene omitted
Position on chr11 (Mb)
1−oleoyl−2−linoleoyl−GPE (18:1/18:2)*
rs261291
HDL cholesterol
Cardiovascular disease risk factors
HDL cholesterol
Asperger
Schizophre
Skin pigm
89 GWAS hits omitted
ALDH1A2 LOC283665 AQP9 LIPC LIPC−AS1 ADAM10 HSP90AB4P LOC101928725 MINDY2 SLTM
Position on chr15 (Mb)
−log10(p−value)
Recombination rate (cM/Mb)
r2

1−(1−enyl−palmitoyl)−2−arachidonoyl−GPC (P−16:0/20:4)*

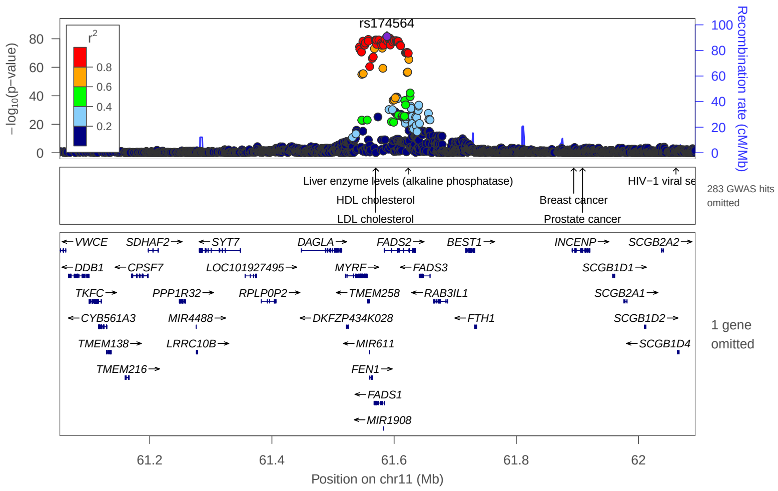

1−(1−enyl−palmitoyl)−2−arachidonoyl−GPC (P−16:0/20:4)*

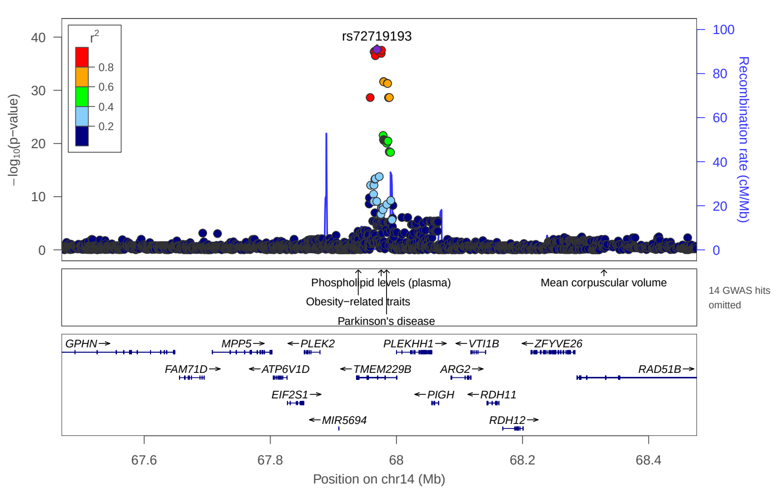

1−linoleoyl−2−arachidonoyl−GPC (18:2/20:4n6)*

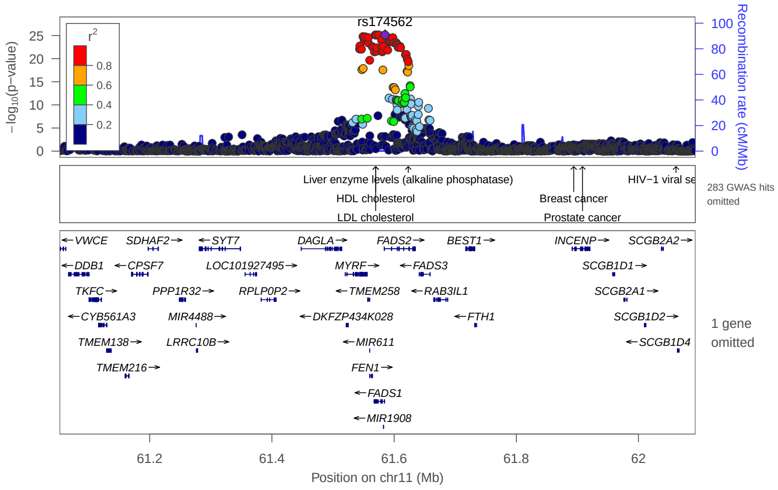

1−(1−enyl−palmitoyl)−2−palmitoyl−GPC (P−16:0/16:0)*

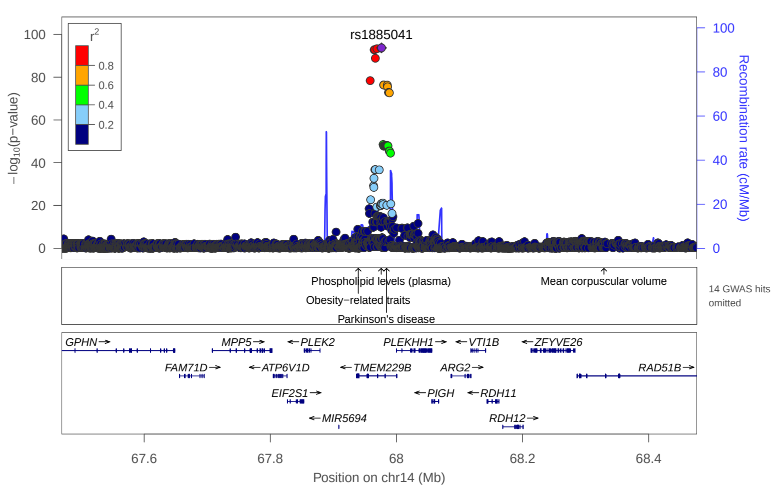

1−(1−enyl−palmitoyl)−2−palmitoyl−GPC (P−16:0/16:0)*

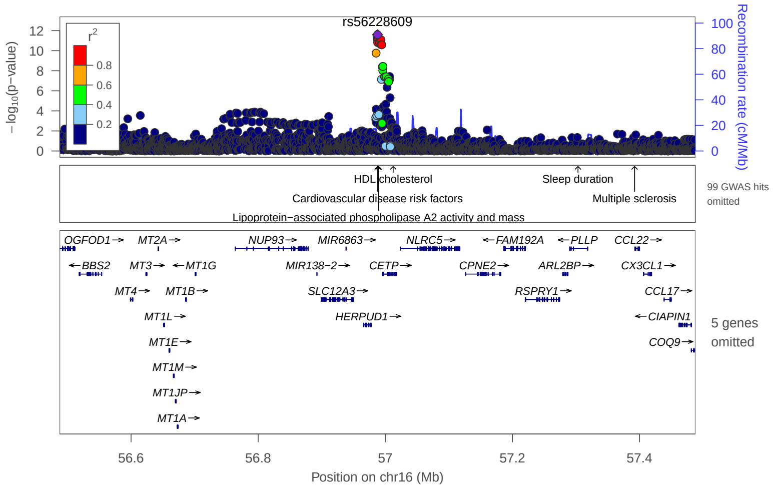

1−stearoyl−2−oleoyl−GPI (18:0/18:1)*

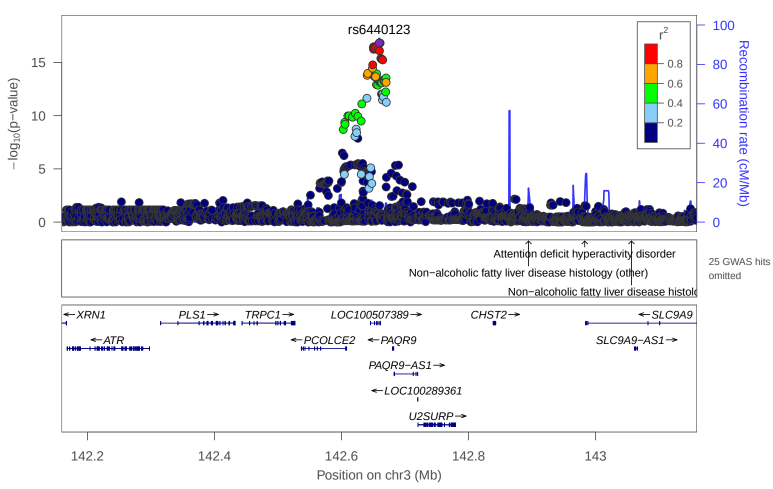

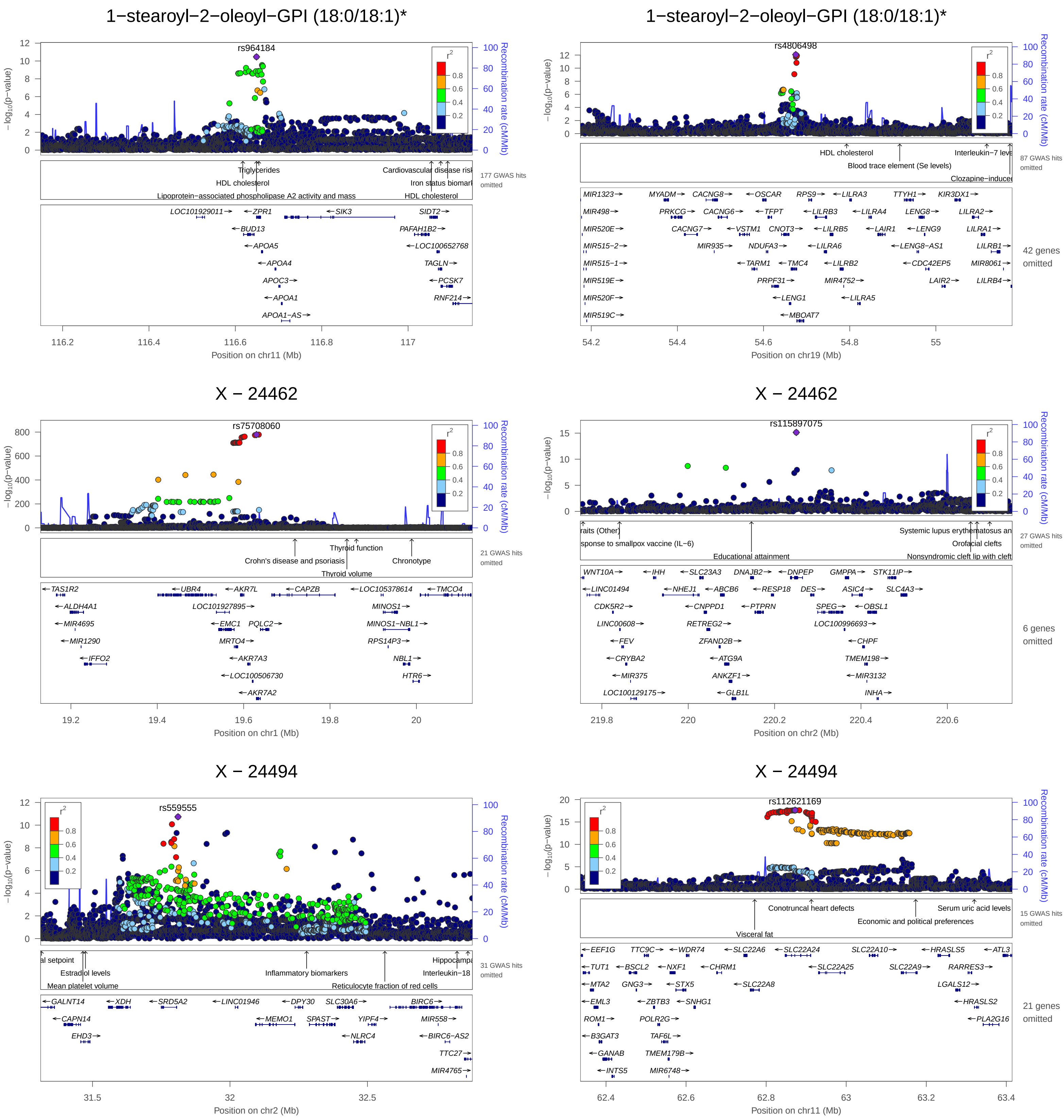

1−stearoyl−2−oleoyl−GPI (18:0/18:1)*
rs964184
Triglycerides
HDL cholesterol
Lipoprotein−associated phospholipase A2 activity and mass
Cardiovascular disease risk
Iron status biomarkers
177 GWAS hits omitted
Position on chr11 (Mb)
1−stearoyl−2−oleoyl−GPI (18:0/18:1)*
rs4806498
HDL cholesterol
Blood trace element (Se levels)
Interleukin−7 levels
Clozapine−induced
87 GWAS hits omitted
42 genes omitted
Position on chr19 (Mb)
X − 24462
rs75708060
Thyroid function
Crohn's disease and psoriasis
Chronotype
Thyroid volume
21 GWAS hits omitted
Position on chr1 (Mb)
X − 24462
rs115897075
raits (Other)
sponse to smallpox vaccine (IL−6)
Educational attainment
Systemic lupus erythematosus and
Orofacial clefts
Nonsyndromic cleft lip with cleft
27 GWAS hits omitted
6 genes omitted
Position on chr2 (Mb)
X − 24494
rs559555
al setpoint
Estradiol levels
Mean platelet volume
Inflammatory biomarkers
Reticulocyte fraction of red cells
Hippocampal
Interleukin−18
31 GWAS hits omitted
Position on chr2 (Mb)
X − 24494
rs112621169
Conotruncal heart defects
Visceral fat
Serum uric acid levels
Economic and political preferences
15 GWAS hits omitted
21 genes omitted
Position on chr11 (Mb)
−log10(p−value)
Recombination rate (cM/Mb)

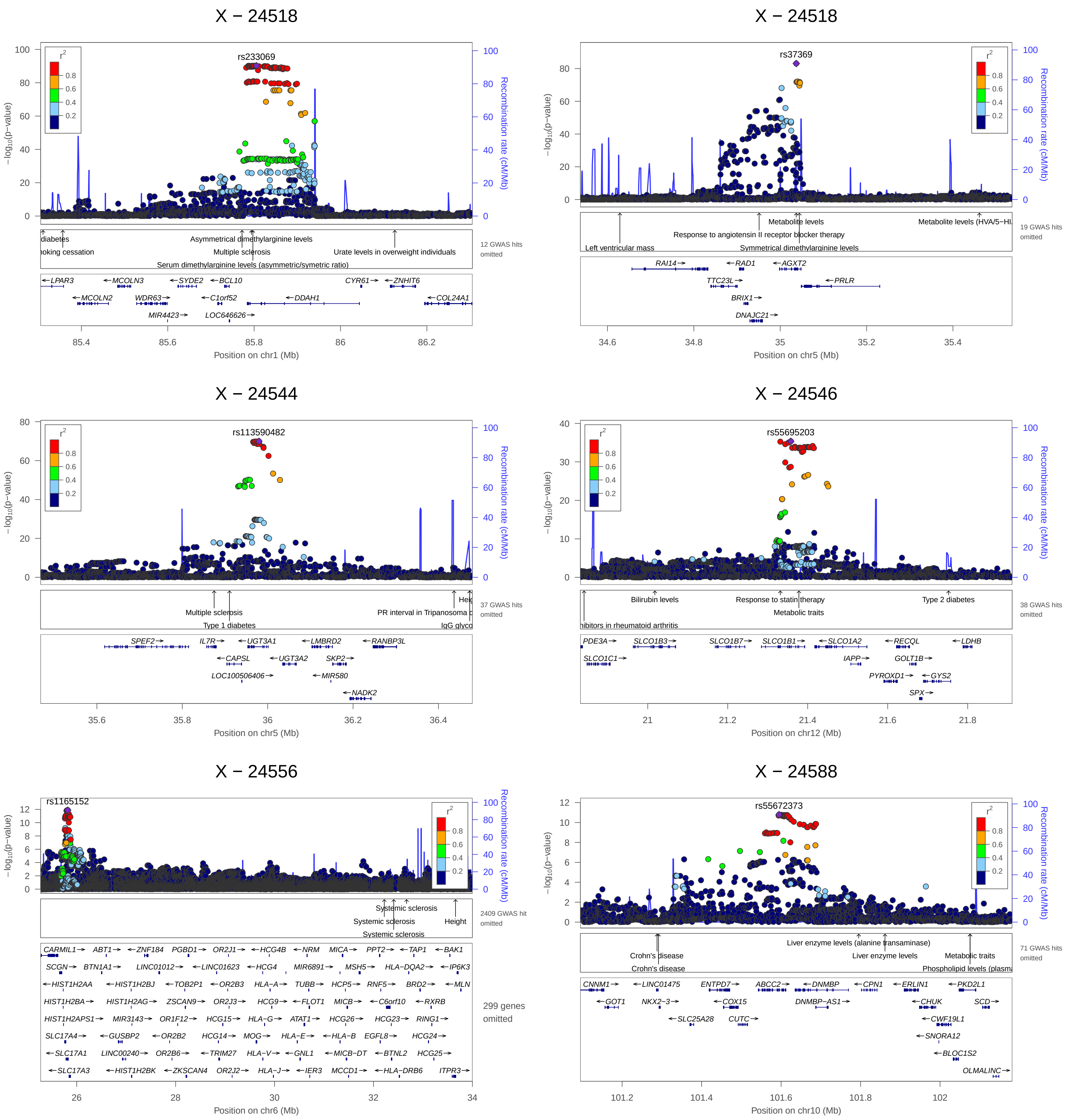
X − 24518
rs233069
Position on chr1 (Mb)
12 GWAS hits omitted
X − 24518
rs37369
Position on chr5 (Mb)
19 GWAS hits omitted
X − 24544
rs113590482
Position on chr5 (Mb)
37 GWAS hits omitted
X − 24546
rs55695203
Position on chr12 (Mb)
38 GWAS hits omitted
X − 24556
rs1165152
Position on chr6 (Mb)
2409 GWAS hit omitted
299 genes omitted
X − 24588
rs55672373
Position on chr10 (Mb)
71 GWAS hits omitted
−log10(p−value)
Recombination rate (cM/Mb)

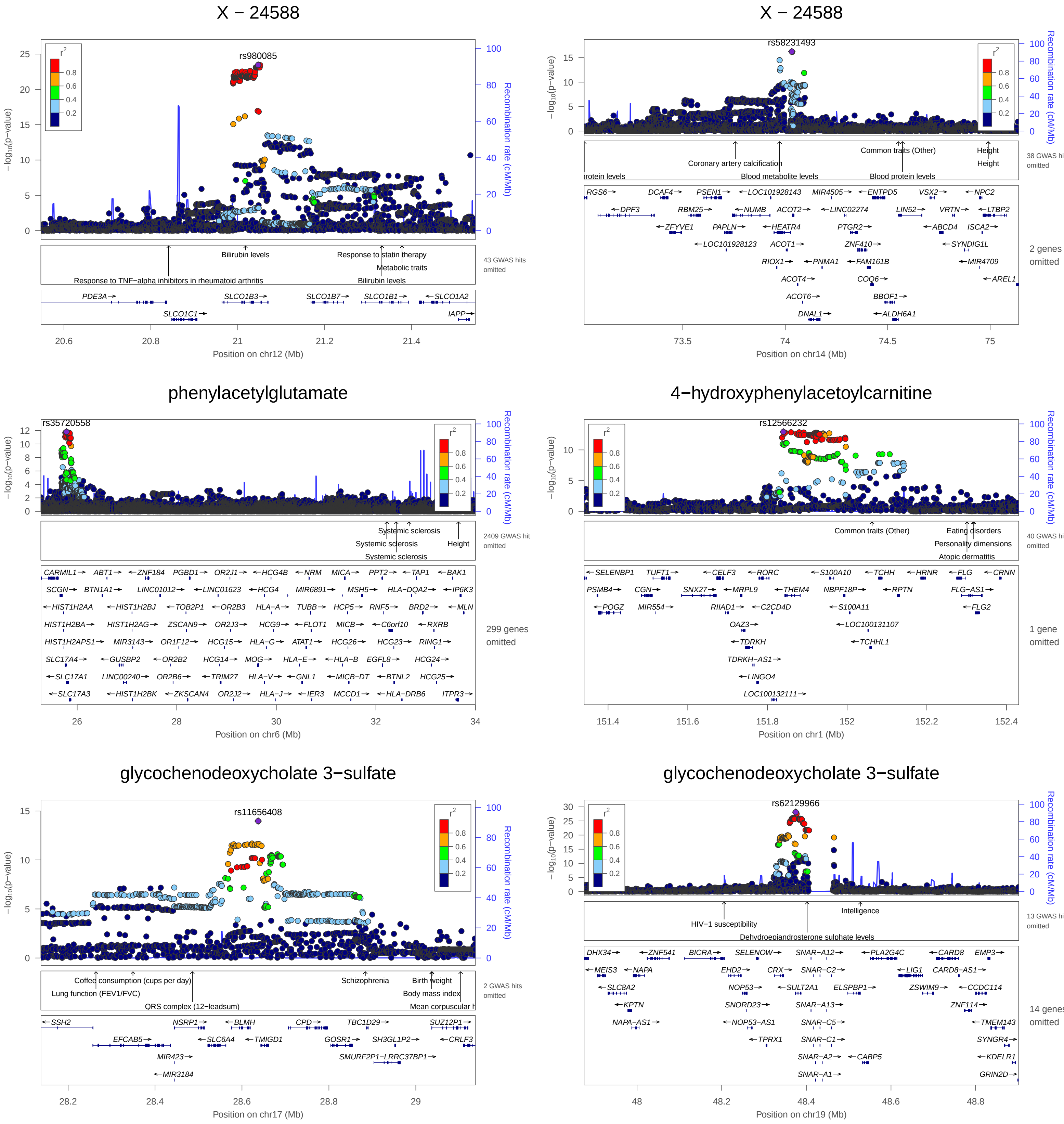

X − 24588
rs980085
−log10(p−value)
Recombination rate (cM/Mb)
Bilirubin levels
Response to statin therapy
Metabolic traits
Response to TNF−alpha inhibitors in rheumatoid arthritis
Bilirubin levels
43 GWAS hits omitted
PDE3A
SLCO1B3
SLCO1B7
SLCO1B1
SLCO1A2
SLCO1C1
IAPP
Position on chr12 (Mb)
X − 24588
rs58231493
Coronary artery calcification
Common traits (Other)
Height
Height
rotein levels
Blood metabolite levels
Blood protein levels
38 GWAS hits omitted
2 genes omitted
Position on chr14 (Mb)
phenylacetylglutamate
rs35720558
Systemic sclerosis
Systemic sclerosis
Height
Systemic sclerosis
2409 GWAS hit omitted
299 genes omitted
Position on chr6 (Mb)
4−hydroxyphenylacetoylcarnitine
rs12566232
Common traits (Other)
Eating disorders
Personality dimensions
Atopic dermatitis
40 GWAS hits omitted
1 gene omitted
Position on chr1 (Mb)
glycochenodeoxycholate 3−sulfate
rs11656408
Coffee consumption (cups per day)
Lung function (FEV1/FVC)
QRS complex (12−leadsum)
Schizophrenia
Birth weight
Body mass index
Mean corpuscular h
2 GWAS hits omitted
Position on chr17 (Mb)
glycochenodeoxycholate 3−sulfate
rs62129966
HIV−1 susceptibility
Intelligence
Dehydroepiandrosterone sulphate levels
13 GWAS hits omitted
14 genes omitted
Position on chr19 (Mb)

glycodeoxycholate 3−sulfate

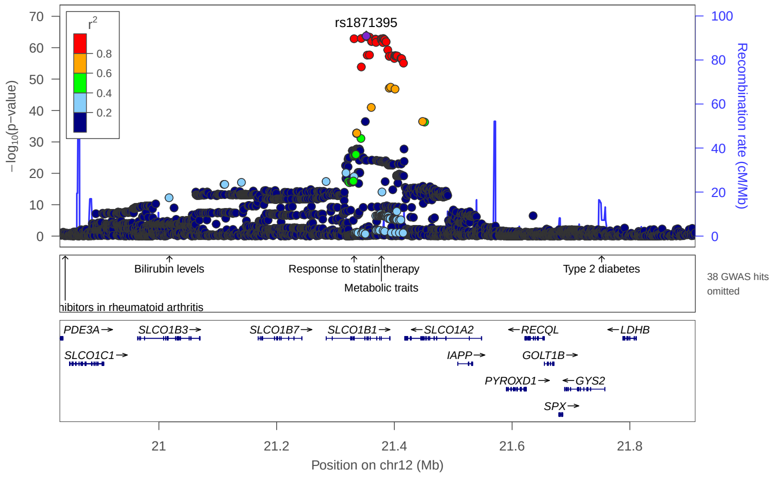

glycodeoxycholate 3−sulfate

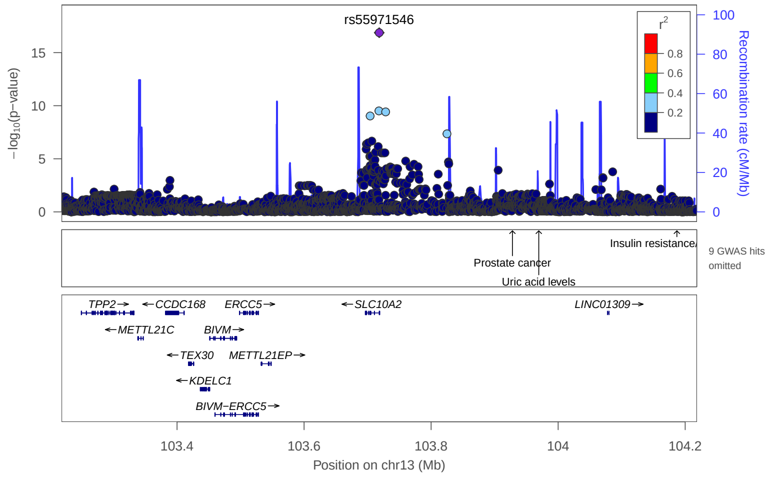

glycodeoxycholate 3−sulfate

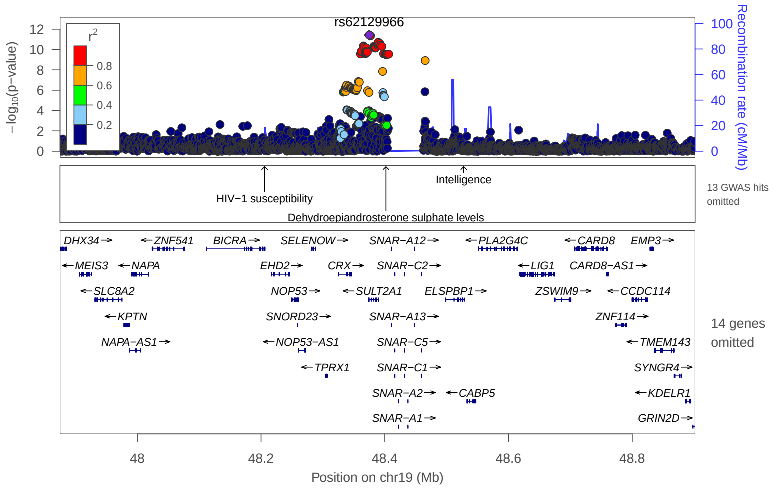

glycochenodeoxycholate glucuronide (1)

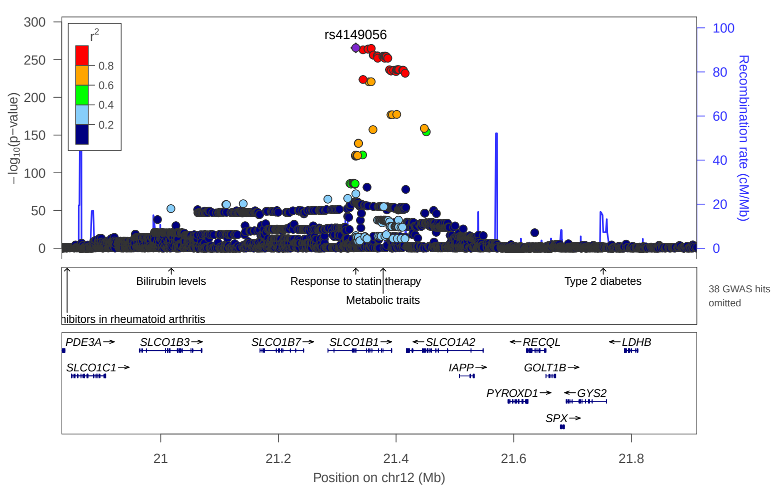

adipoylcarnitine (C6−DC)

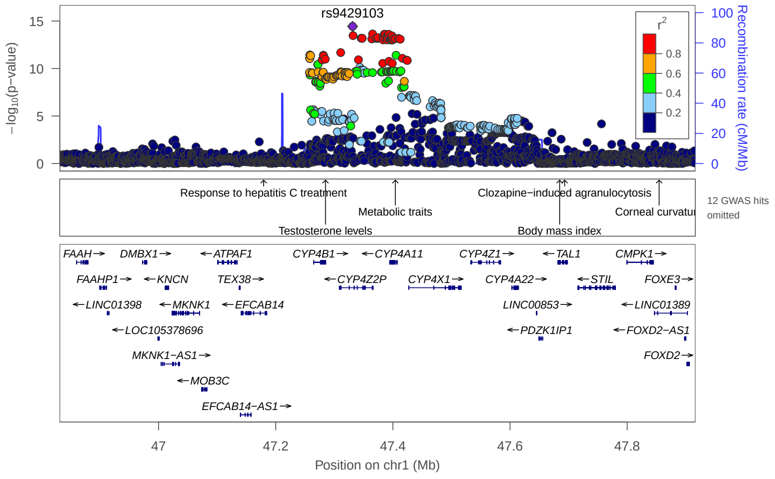

adipoylcarnitine (C6−DC)

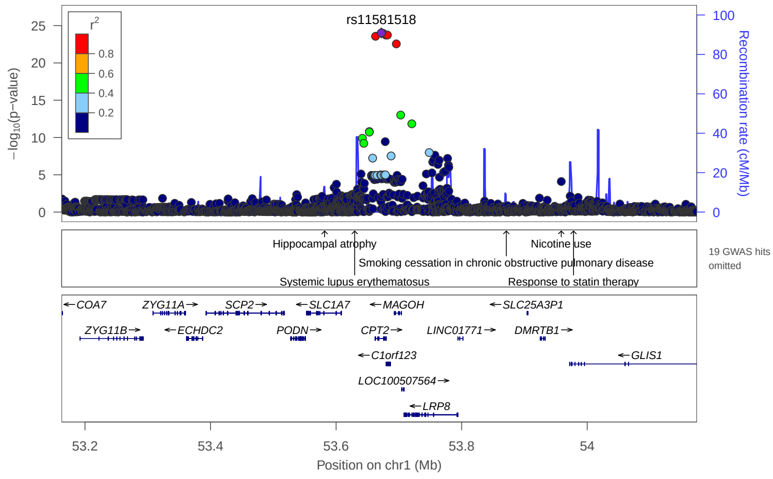

adipoylcarnitine (C6−DC)

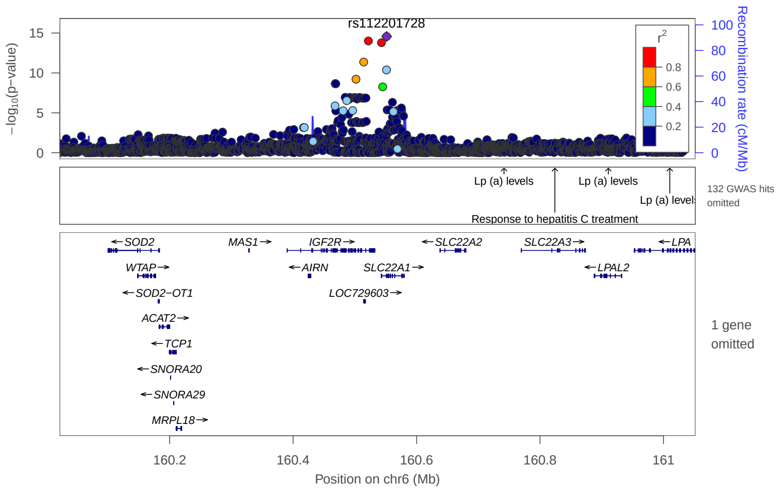

suberoylcarnitine (C8−DC)

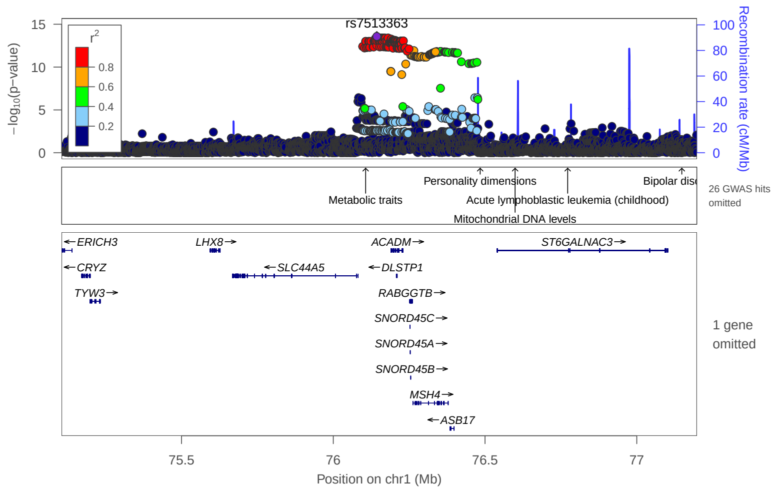

suberoylcarnitine (C8−DC)

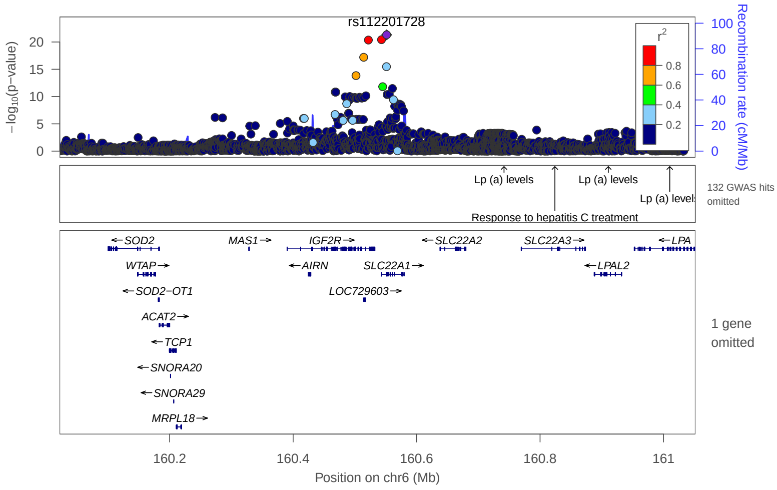

suberoylcarnitine (C8−DC)

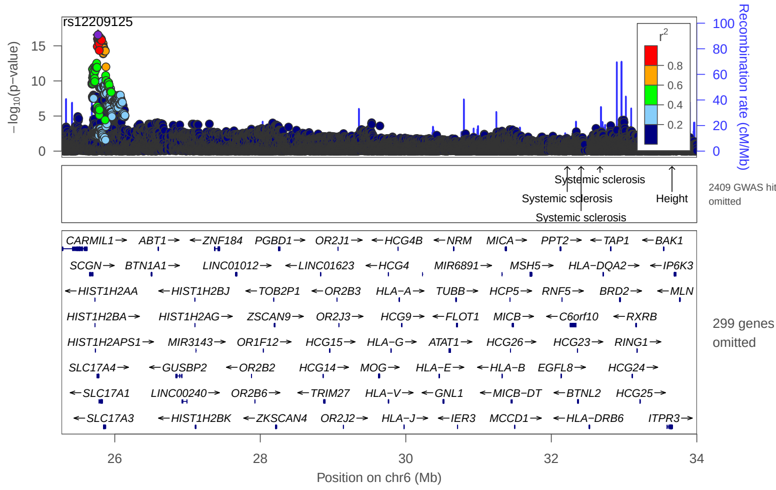

lactosyl−N−palmitoyl−sphingosine (d18:1/16:0)

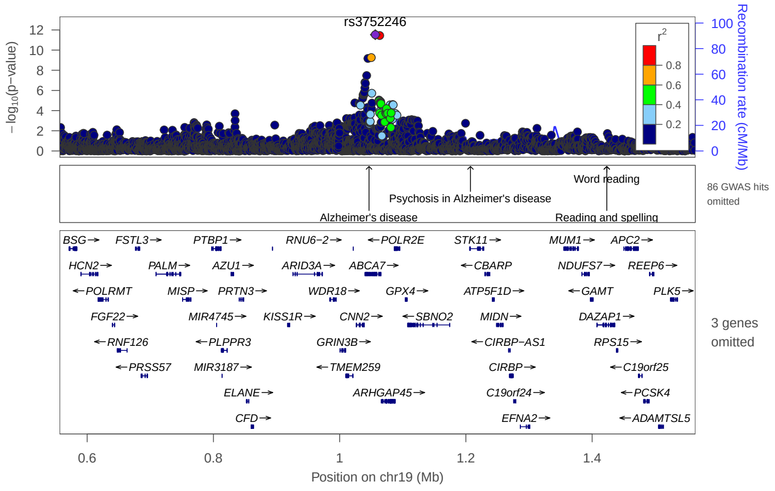

glycosyl−N−palmitoyl−sphingosine (d18:1/16:0)

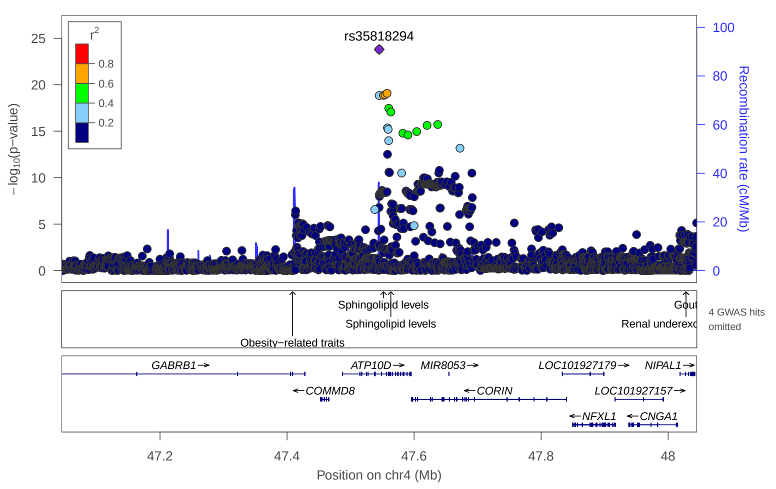

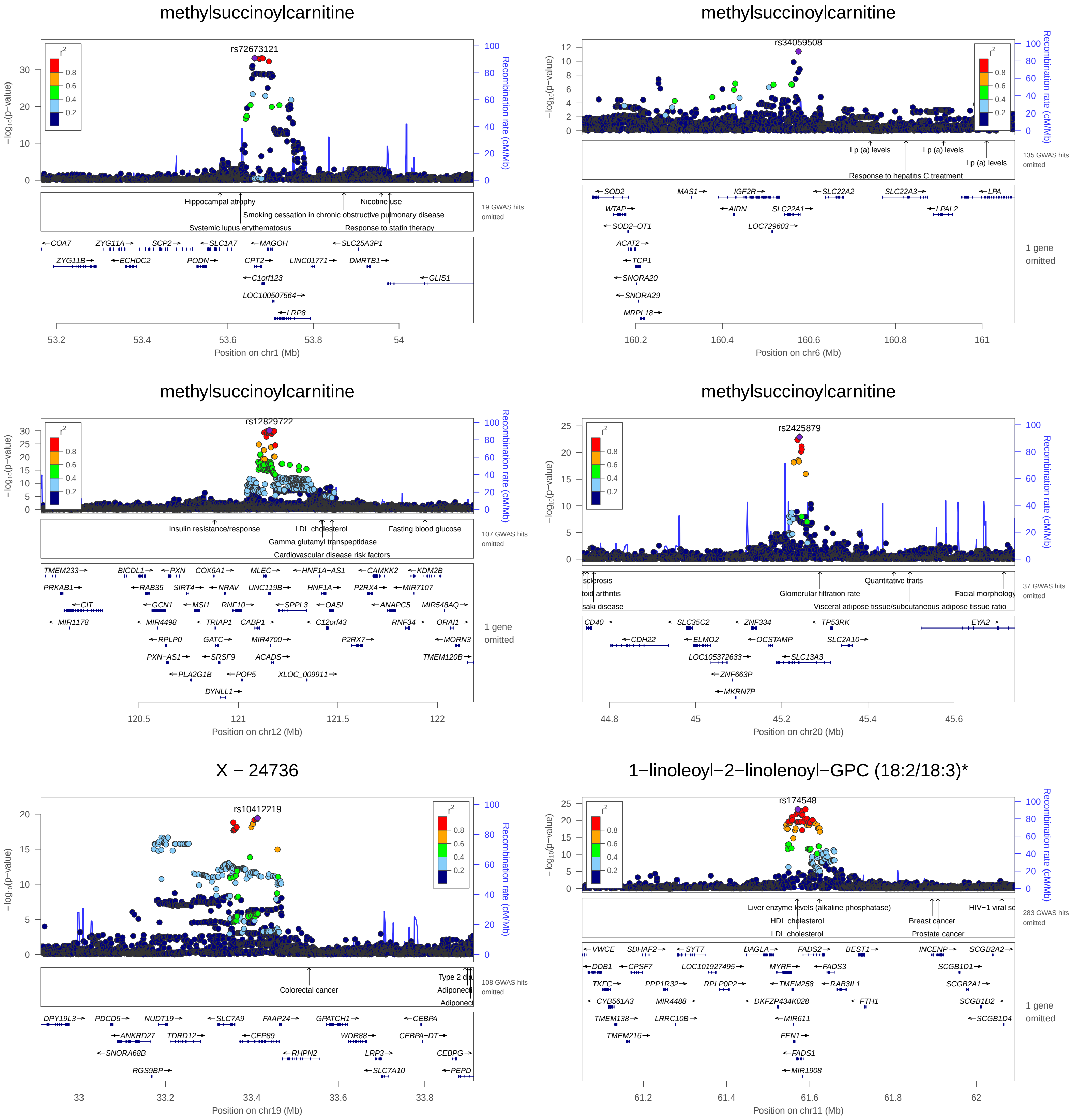
methylsuccinoylcarnitine
rs72673121
Position on chr1 (Mb)
Hippocampal atrophy
Nicotine use
Smoking cessation in chronic obstructive pulmonary disease
Systemic lupus erythematosus
Response to statin therapy
19 GWAS hits omitted
methylsuccinoylcarnitine
rs34059508
Position on chr6 (Mb)
Lp (a) levels
Response to hepatitis C treatment
135 GWAS hits omitted
1 gene omitted
methylsuccinoylcarnitine
rs12829722
Position on chr12 (Mb)
Insulin resistance/response
LDL cholesterol
Fasting blood glucose
Gamma glutamyl transpeptidase
Cardiovascular disease risk factors
107 GWAS hits omitted
1 gene omitted
methylsuccinoylcarnitine
rs2425879
Position on chr20 (Mb)
Glomerular filtration rate
Quantitative traits
Facial morphology
Visceral adipose tissue/subcutaneous adipose tissue ratio
37 GWAS hits omitted
X − 24736
rs10412219
Position on chr19 (Mb)
Colorectal cancer
108 GWAS hits omitted
1−linoleoyl−2−linolenoyl−GPC (18:2/18:3)*
rs174548
Position on chr11 (Mb)
Liver enzyme levels (alkaline phosphatase)
HDL cholesterol
LDL cholesterol
Breast cancer
Prostate cancer
283 GWAS hits omitted
1 gene omitted
−log10(p−value)
Recombination rate (cM/Mb)

1−palmitoleoyl−2−linolenoyl−GPC (16:1/18:3)*

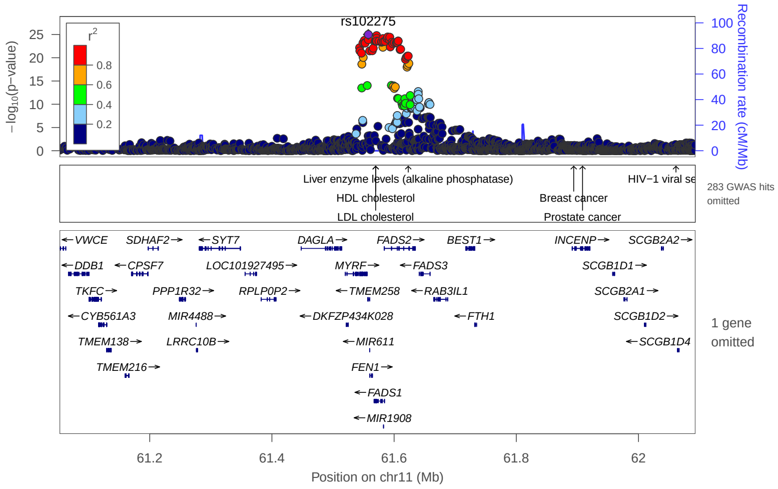

1−myristoyl−2−arachidonoyl−GPC (14:0/20:4)*

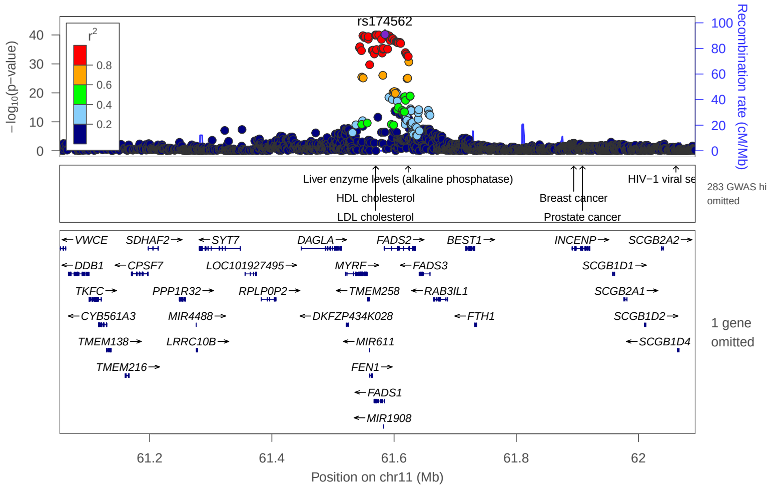

pimeloylcarnitine/3−methyladipoylcarnitine (C7−DC)

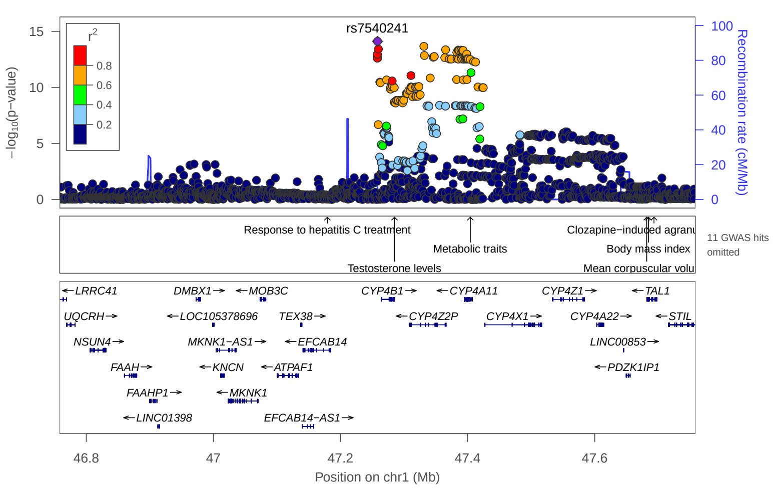

pimeloylcarnitine/3−methyladipoylcarnitine (C7−DC)

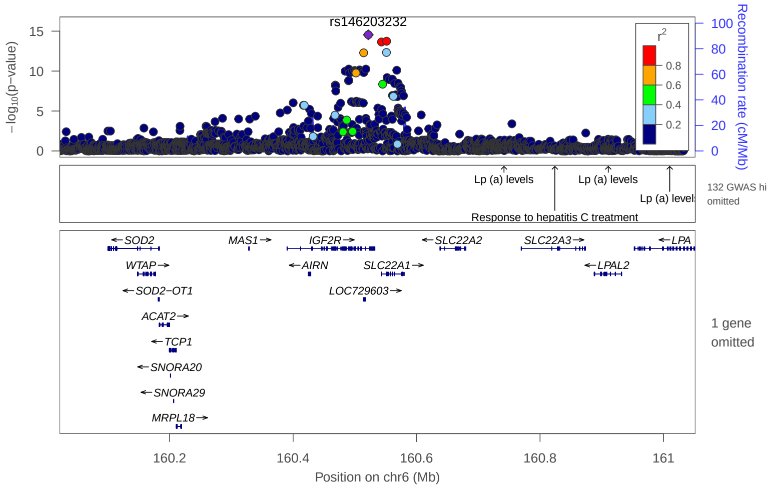

3−hydroxyhexanoate

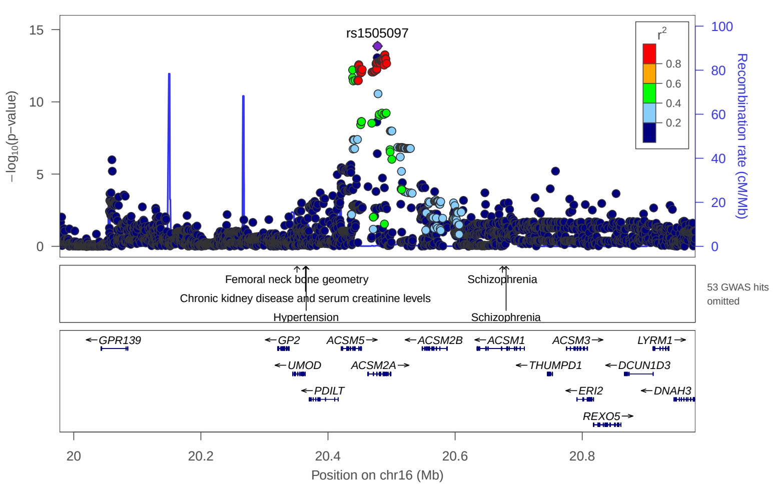

arachidonoylcholine

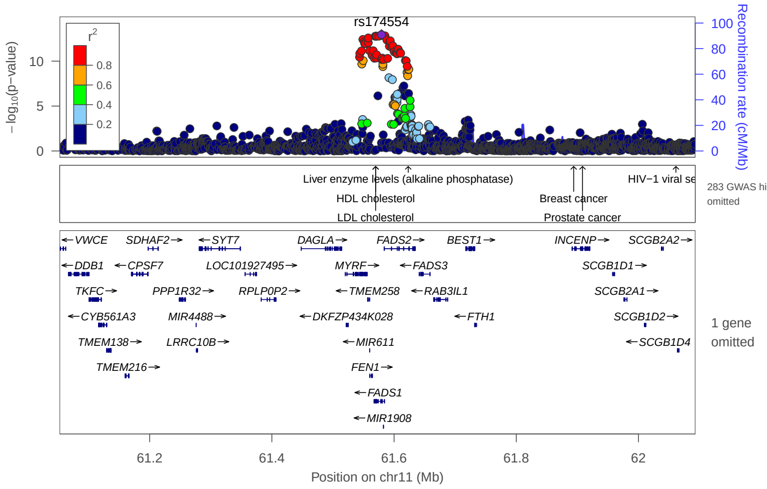

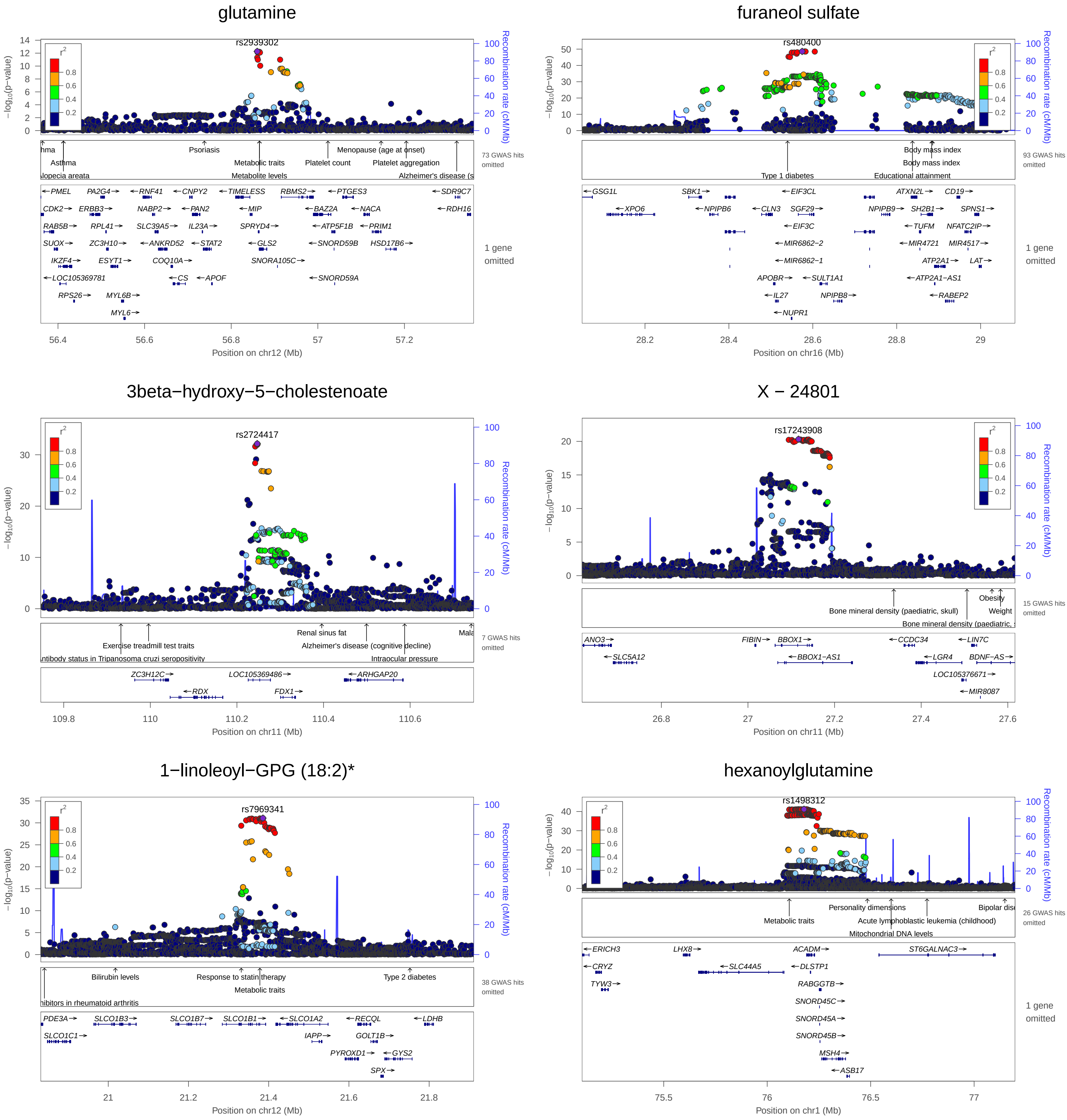

glutamine
rs2939302
Position on chr12 (Mb)
73 GWAS hits omitted
1 gene omitted
furaneol sulfate
rs480400
Position on chr16 (Mb)
93 GWAS hits omitted
1 gene omitted
3beta−hydroxy−5−cholestenoate
rs2724417
Position on chr11 (Mb)
7 GWAS hits omitted
X − 24801
rs17243908
Position on chr11 (Mb)
15 GWAS hits omitted
1−linoleoyl−GPG (18:2)*
rs7969341
Position on chr12 (Mb)
38 GWAS hits omitted
hexanoylglutamine
rs1498312
Position on chr1 (Mb)
26 GWAS hits omitted
1 gene omitted
−log10(p−value)
Recombination rate (cM/Mb)

hexanoylglutamine

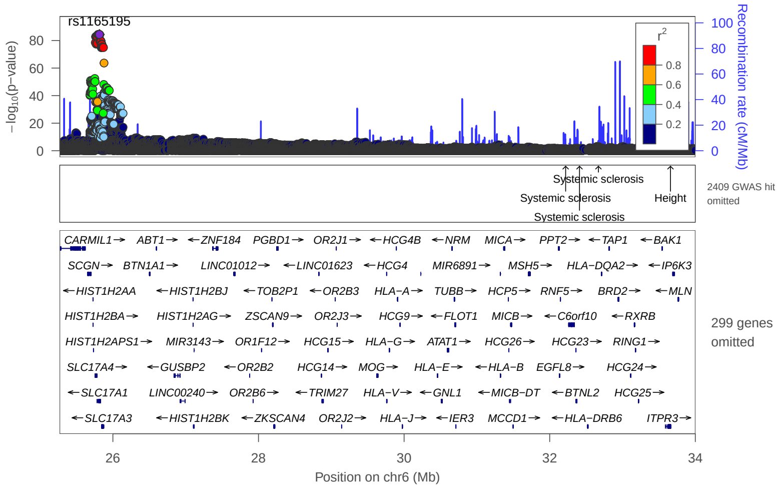

beta−citrylglutamate

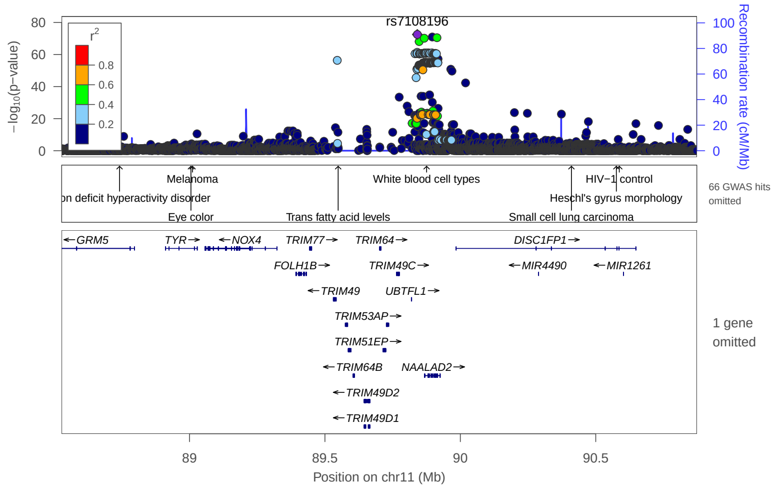

oleoyl−oleoyl−glycerol (18:1/18:1) [1]*

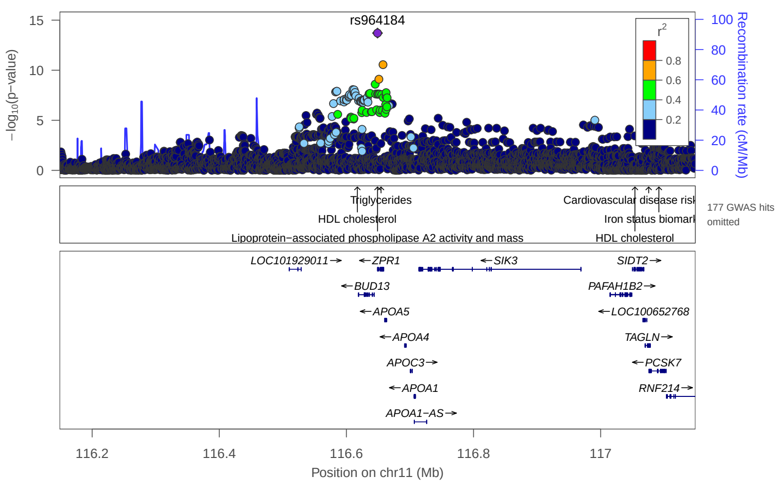

oleoyl−oleoyl−glycerol (18:1/18:1) [2]*

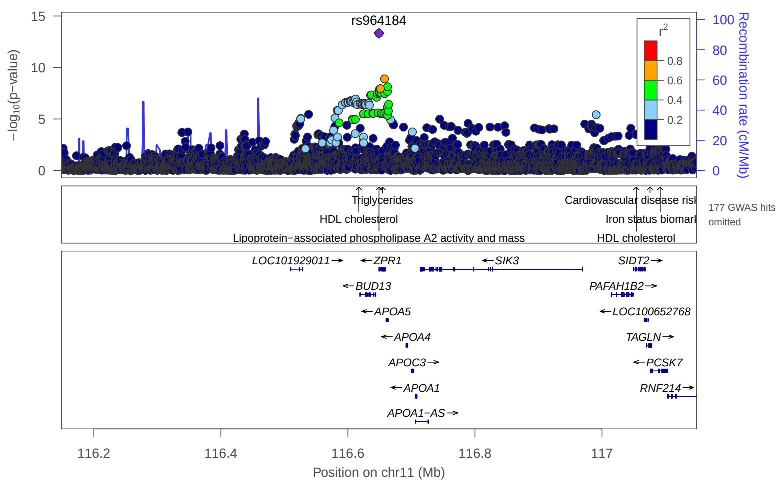

linoleoyl−arachidonoyl−glycerol (18:2/20:4) [1]*

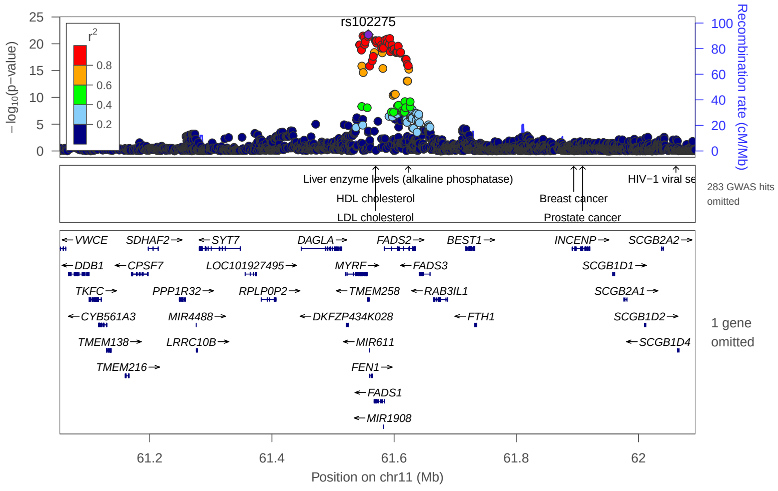

linoleoyl−arachidonoyl−glycerol (18:2/20:4) [1]*

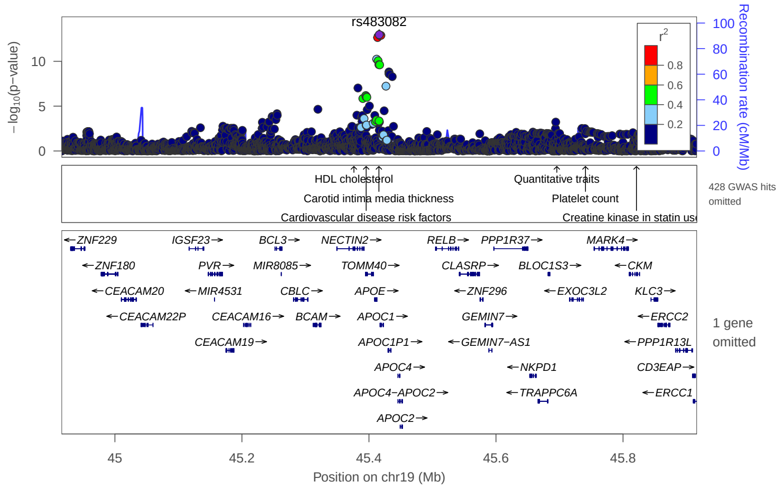

linoleoyl−arachidonoyl−glycerol (18:2/20:4) [2]*

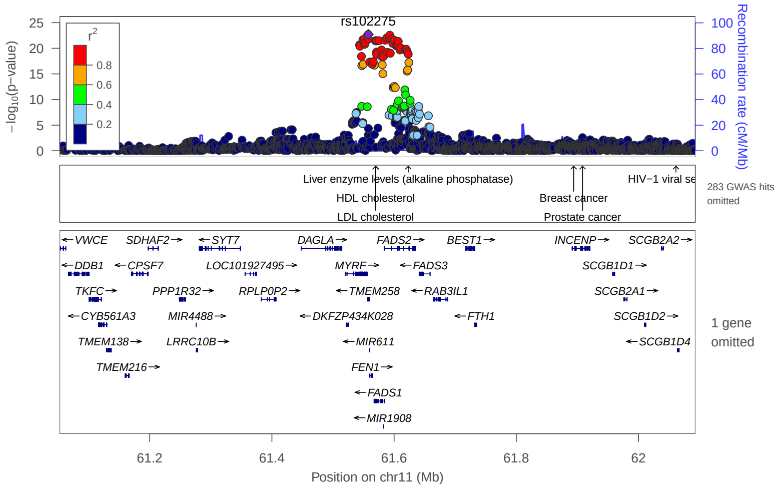

linoleoyl−arachidonoyl−glycerol (18:2/20:4) [2]*

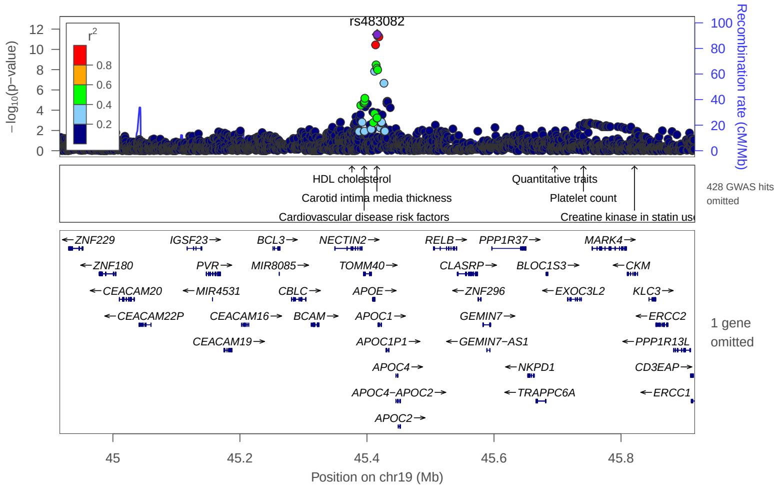

oleoyl−arachidonoyl−glycerol (18:1/20:4) [1]*

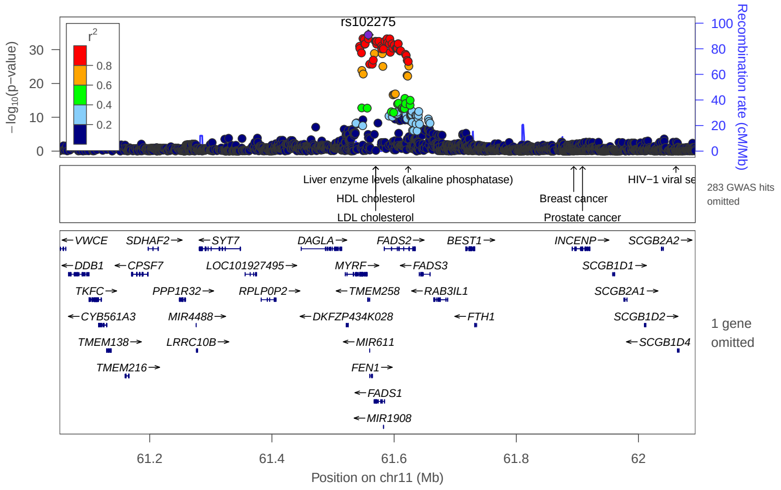

oleoyl−arachidonoyl−glycerol (18:1/20:4) [1]*

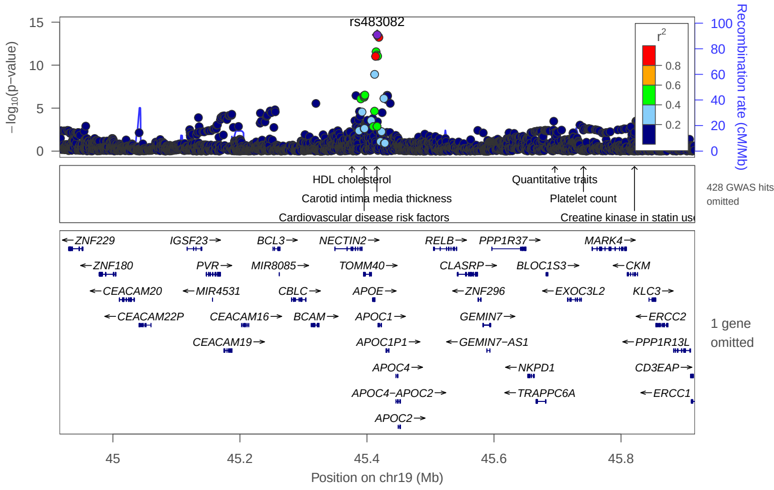

oleoyl−arachidonoyl−glycerol (18:1/20:4) [2]*

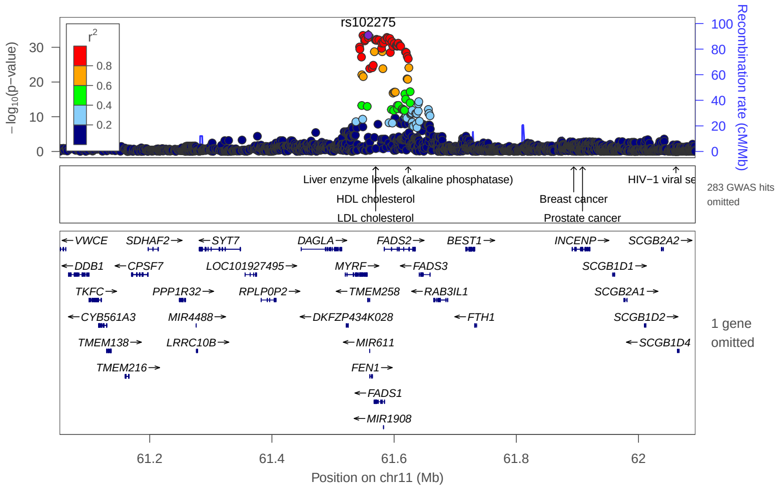

oleoyl−arachidonoyl−glycerol (18:1/20:4) [2]*

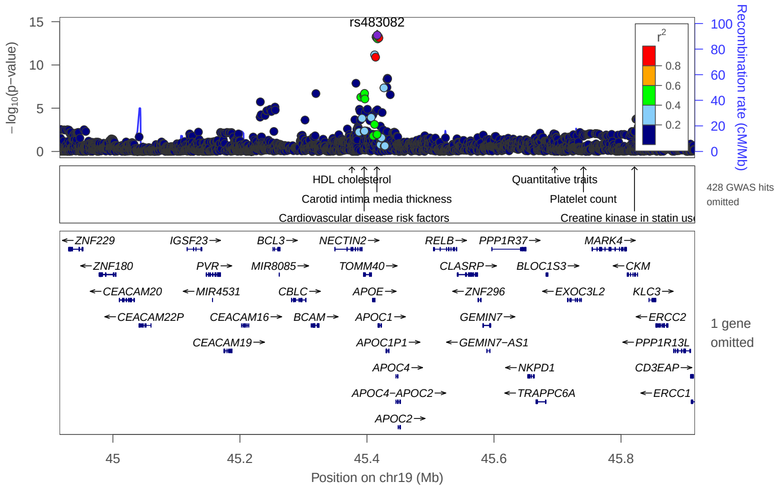

linoleoyl−linoleoyl−glycerol (18:2/18:2) [1]*

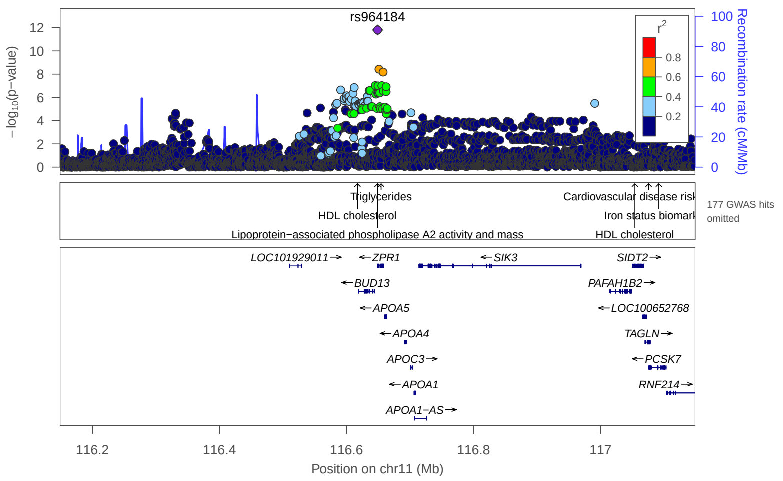

N−stearoyl−sphingosine (d18:1/18:0)*

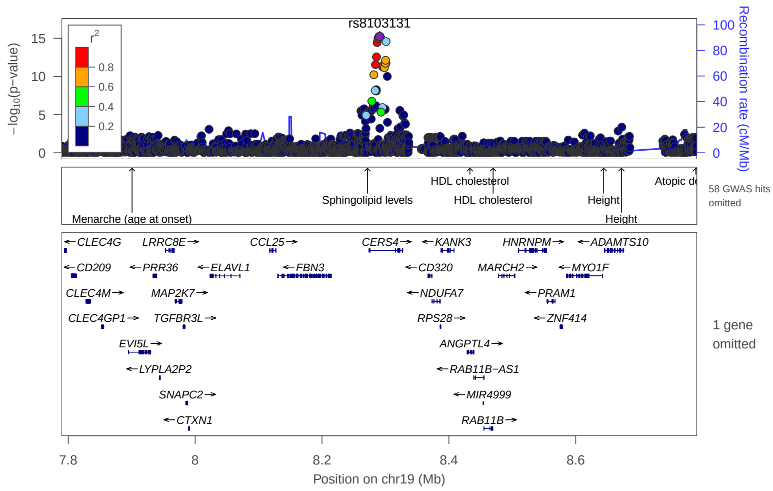

N−stearoyl−sphingosine (d18:1/18:0)*

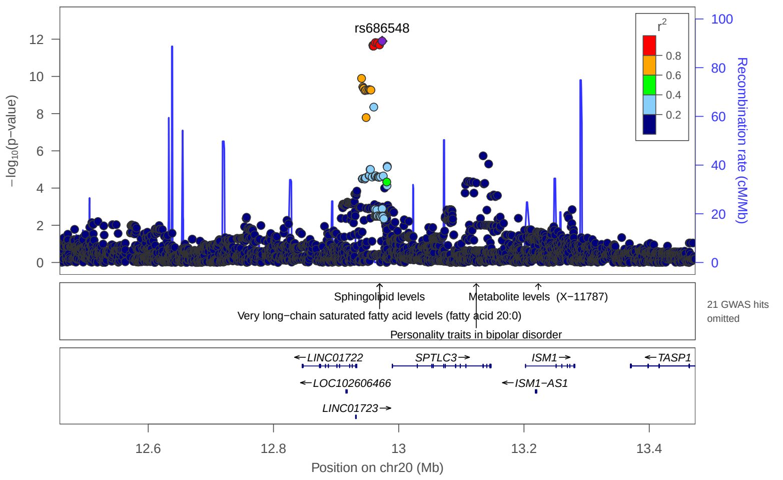

1−oleoyl−2−arachidonoyl−GPE (18:1/20:4)*

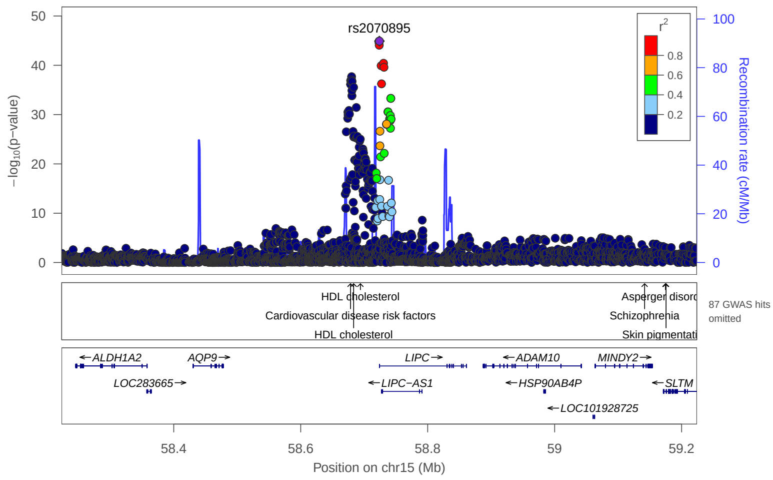

behenoyl dihydrosphingomyelin (d18:0/22:0)*

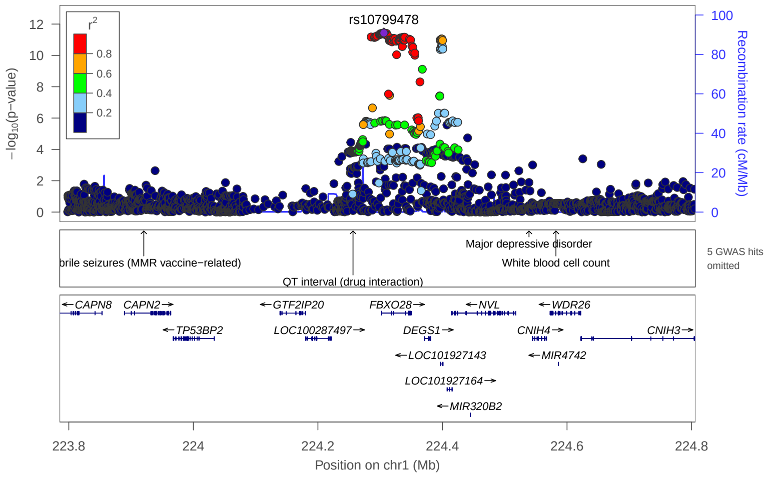

myristoyl dihydrosphingomyelin (d18:0/14:0)*

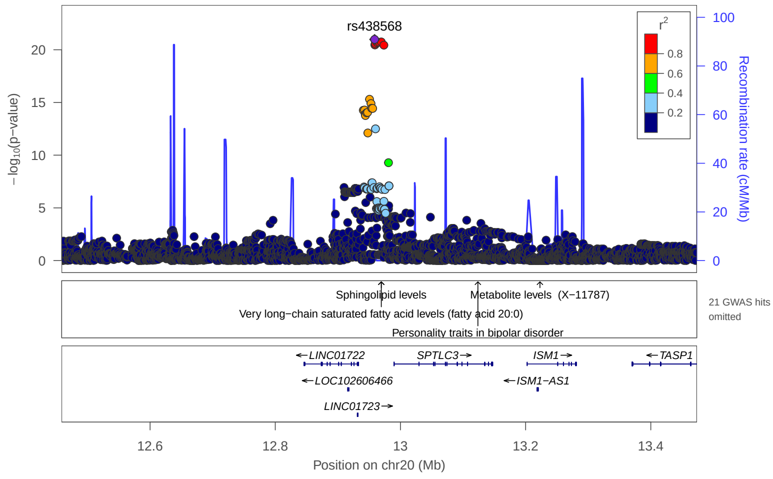

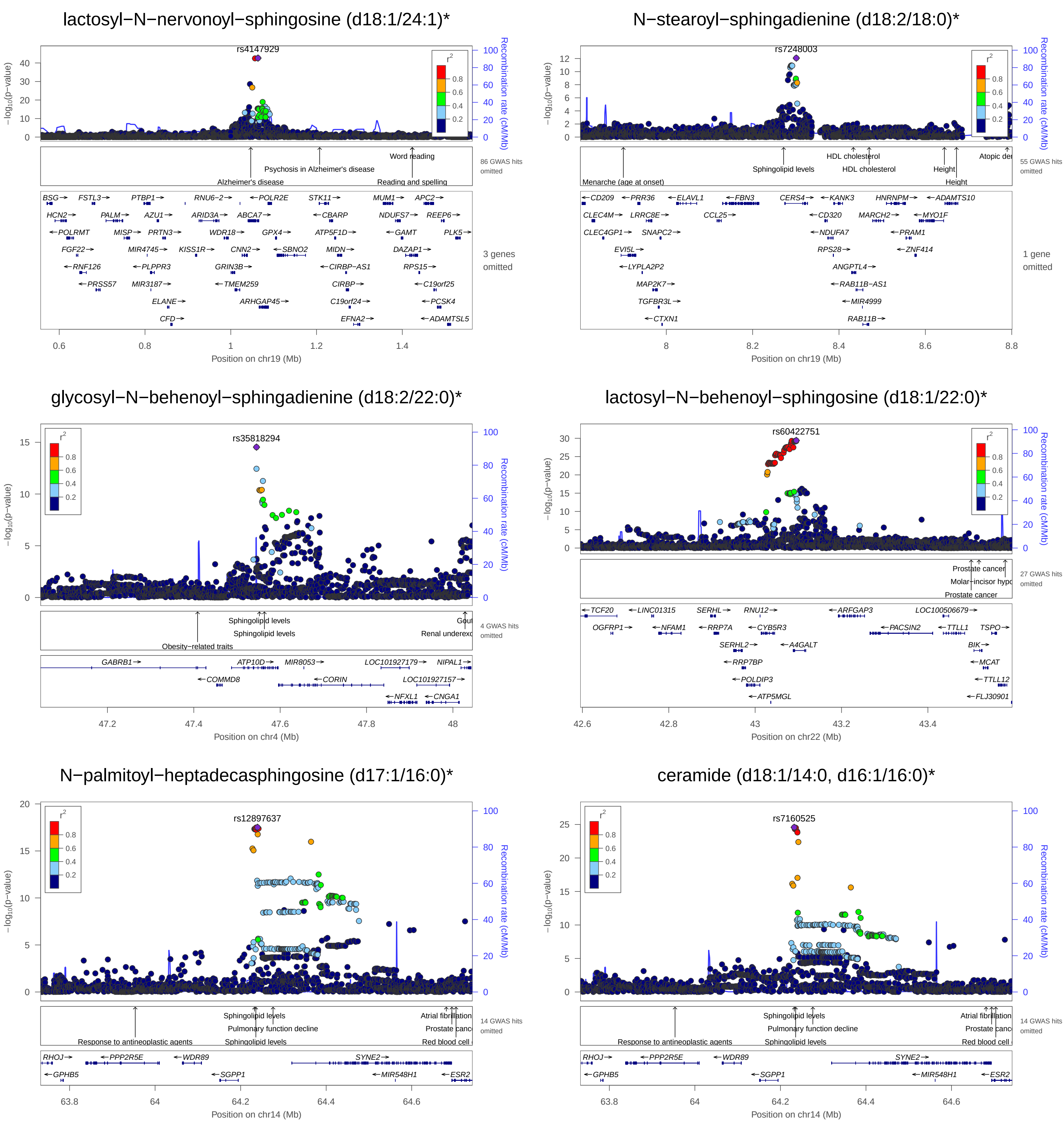

lactosyl−N−nervonoyl−sphingosine (d18:1/24:1)*
rs4147929
Alzheimer's disease
Psychosis in Alzheimer's disease
Word reading
Reading and spelling
86 GWAS hits omitted
3 genes omitted
Position on chr19 (Mb)
N−stearoyl−sphingadienine (d18:2/18:0)*
rs7248003
Menarche (age at onset)
Sphingolipid levels
HDL cholesterol
Height
55 GWAS hits omitted
1 gene omitted
Position on chr19 (Mb)
glycosyl−N−behenoyl−sphingadienine (d18:2/22:0)*
rs35818294
Obesity−related traits
Sphingolipid levels
4 GWAS hits omitted
Position on chr4 (Mb)
lactosyl−N−behenoyl−sphingosine (d18:1/22:0)*
rs60422751
Prostate cancer
27 GWAS hits omitted
Position on chr22 (Mb)
N−palmitoyl−heptadecasphingosine (d17:1/16:0)*
rs12897637
Response to antineoplastic agents
Sphingolipid levels
Pulmonary function decline
Atrial fibrillation
14 GWAS hits omitted
Position on chr14 (Mb)
ceramide (d18:1/14:0, d16:1/16:0)*
rs7160525
Response to antineoplastic agents
Sphingolipid levels
Pulmonary function decline
Atrial fibrillation
14 GWAS hits omitted
Position on chr14 (Mb)
−log10(p−value)
Recombination rate (cM/Mb)

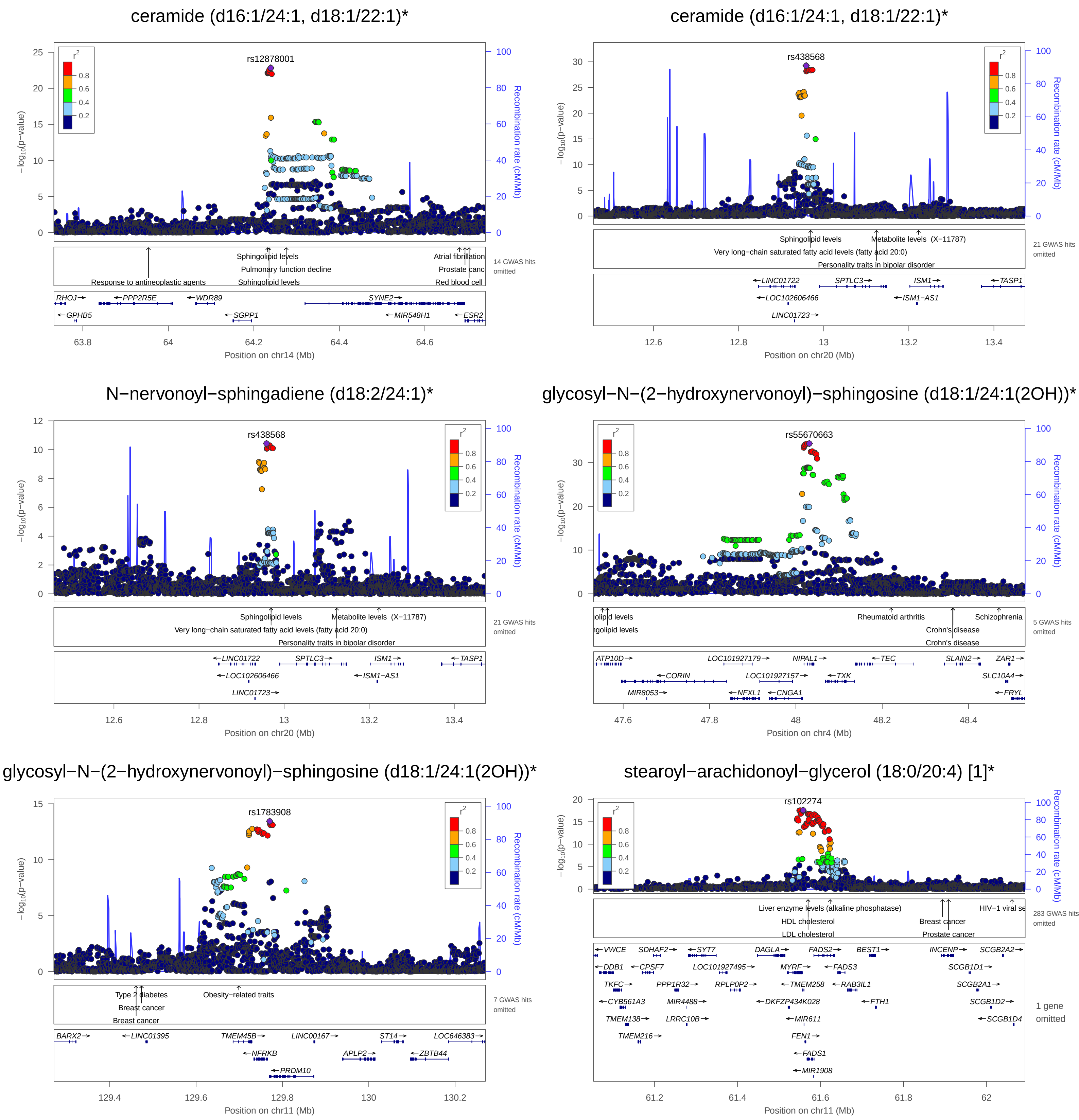

ceramide (d16:1/24:1, d18:1/22:1)*
rs12878001
Sphingolipid levels
Pulmonary function decline
Sphingolipid levels
Response to antineoplastic agents
Atrial fibrillation
Red blood cell
14 GWAS hits omitted
RHOJ
PPP2R5E
WDR89
SYNE2
GPHB5
SGPP1
MIR548H1
ESR2
Position on chr14 (Mb)
ceramide (d16:1/24:1, d18:1/22:1)*
rs438568
Sphingolipid levels
Metabolite levels (X−11787)
Very long−chain saturated fatty acid levels (fatty acid 20:0)
Personality traits in bipolar disorder
21 GWAS hits omitted
LINC01722
SPTLC3
ISM1
TASP1
LOC102606466
ISM1−AS1
LINC01723
Position on chr20 (Mb)
N−nervonoyl−sphingadiene (d18:2/24:1)*
rs438568
Sphingolipid levels
Metabolite levels (X−11787)
Very long−chain saturated fatty acid levels (fatty acid 20:0)
Personality traits in bipolar disorder
21 GWAS hits omitted
LINC01722
SPTLC3
ISM1
TASP1
LOC102606466
ISM1−AS1
LINC01723
Position on chr20 (Mb)
glycosyl−N−(2−hydroxynervonoyl)−sphingosine (d18:1/24:1(2OH))*
rs55670663
Rheumatoid arthritis
Schizophrenia
Crohn's disease
Crohn's disease
5 GWAS hits omitted
ATP10D
LOC101927179
NIPAL1
TEC
SLAIN2
ZAR1
CORIN
LOC101927157
TXK
SLC10A4
MIR8053
NFXL1
CNGA1
FRYL
Position on chr4 (Mb)
glycosyl−N−(2−hydroxynervonoyl)−sphingosine (d18:1/24:1(2OH))*
rs1783908
Type 2 diabetes
Breast cancer
Breast cancer
Obesity−related traits
7 GWAS hits omitted
BARX2
LINC01395
TMEM45B
LINC00167
ST14
LOC646383
NFRKB
APLP2
ZBTB44
PRDM10
Position on chr11 (Mb)
stearoyl−arachidonoyl−glycerol (18:0/20:4) [1]*
rs102274
Liver enzyme levels (alkaline phosphatase)
HDL cholesterol
LDL cholesterol
Breast cancer
Prostate cancer
283 GWAS hits omitted
VWCE
SDHAF2
SYT7
DAGLA
FADS2
BEST1
INCENP
SCGB2A2
DDB1
CPSF7
LOC101927495
MYRF
FADS3
SCGB1D1
TKFC
PPP1R32
RPLP0P2
TMEM258
RAB3IL1
SCGB2A1
CYB561A3
MIR4488
DKFZP434K028
FTH1
SCGB1D2
TMEM138
LRRC10B
MIR611
SCGB1D4
TMEM216
FEN1
FADS1
MIR1908
1 gene omitted
Position on chr11 (Mb)

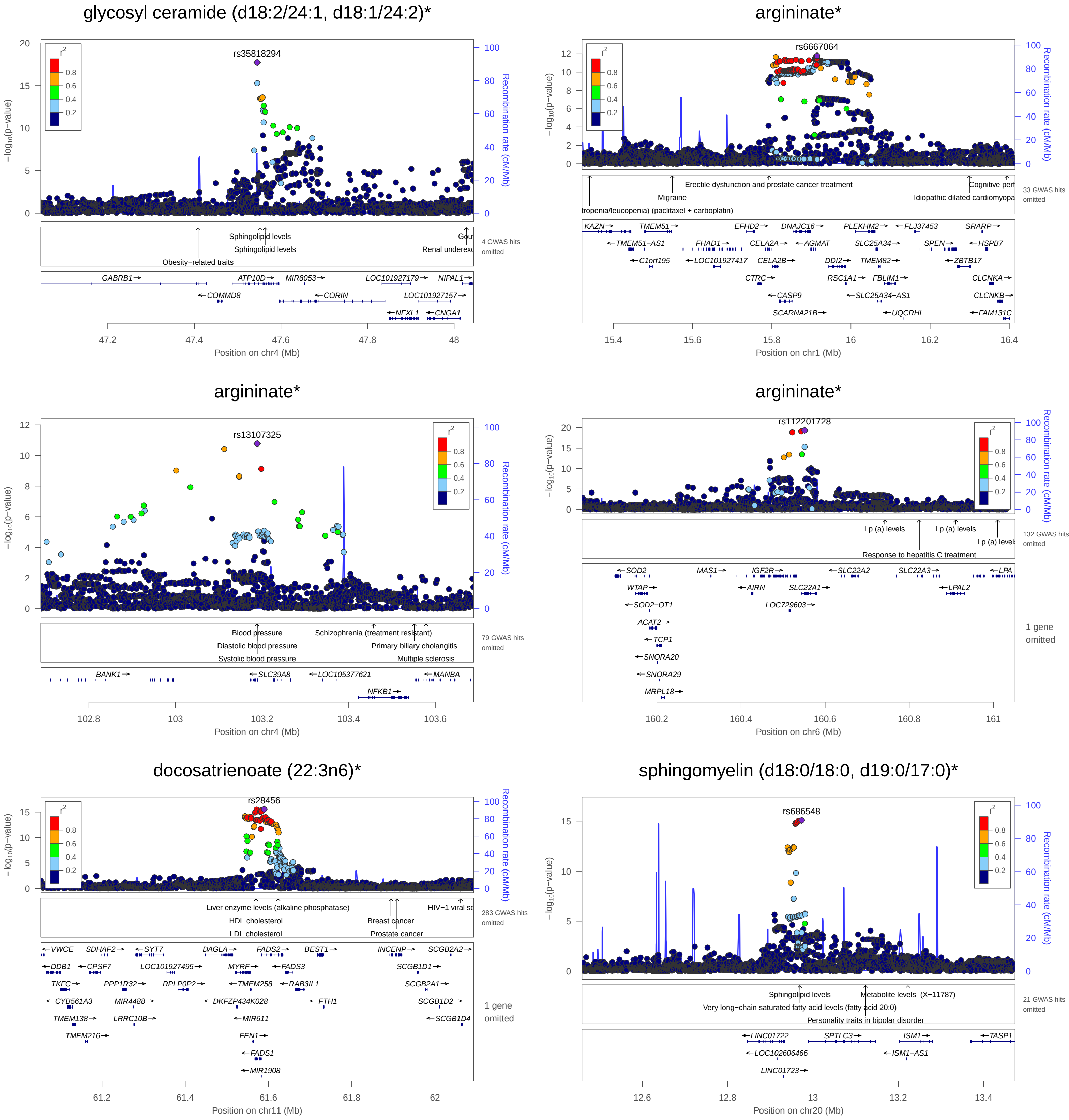
glycosyl ceramide (d18:2/24:1, d18:1/24:2)*
rs35818294
Sphingolipid levels
Obesity−related traits
Gout
Renal underexc
4 GWAS hits omitted
GABRB1 ATP10D MIR8053 LOC101927179 NIPAL1 COMMD8 CORIN LOC101927157 NFXL1 CNGA1
Position on chr4 (Mb)
arginate*
rs6667064
Erectile dysfunction and prostate cancer treatment
Migraine
Cognitive perf
Idiopathic dilated cardiomyopa
tropenia/leucopenia) (paclitaxel + carboplatin)
33 GWAS hits omitted
KAZN TMEM51 EFHD2 DNAJC16 PLEKHM2 FLJ37453 SRARP TMEM51−AS1 FHAD1 CELA2A AGMAT SLC25A34 SPEN HSPB7 C1orf195 LOC101927417 CELA2B DDI2 TMEM82 ZBTB17 CTRC RSC1A1 FBLIM1 CLCNKA CASP9 SLC25A34−AS1 CLCNKB SCARNA21B UQCRHL FAM131C
Position on chr1 (Mb)
arginate*
rs13107325
Blood pressure
Diastolic blood pressure
Systolic blood pressure
Schizophrenia (treatment resistant)
Primary biliary cholangitis
Multiple sclerosis
79 GWAS hits omitted
BANK1 SLC39A8 LOC105377621 MANBA NFKB1
Position on chr4 (Mb)
arginate*
rs112201728
Lp (a) levels
Response to hepatitis C treatment
132 GWAS hits omitted
SOD2 MAS1 IGF2R SLC22A2 SLC22A3 LPA WTAP AIRN SLC22A1 LPAL2 SOD2−OT1 LOC729603 ACAT2 TCP1 SNORA20 SNORA29 MRPL18
1 gene omitted
Position on chr6 (Mb)
docosatrienoate (22:3n6)*
rs28456
Liver enzyme levels (alkaline phosphatase)
HDL cholesterol
LDL cholesterol
Breast cancer
Prostate cancer
HIV−1 viral se
283 GWAS hits omitted
VWCE SDHAF2 SYT7 DAGLA FADS2 BEST1 INCENP SCGB2A2 DDB1 CPSF7 LOC101927495 MYRF FADS3 SCGB1D1 TKFC PPP1R32 RPLP0P2 TMEM258 RAB3IL1 SCGB2A1 CYB561A3 MIR4488 DKFZP434K028 FTH1 SCGB1D2 TMEM138 LRRC10B MIR611 SCGB1D4 TMEM216 FEN1 FADS1 MIR1908
1 gene omitted
Position on chr11 (Mb)
sphingomyelin (d18:0/18:0, d19:0/17:0)*
rs686548
Sphingolipid levels
Metabolite levels (X−11787)
Very long−chain saturated fatty acid levels (fatty acid 20:0)
Personality traits in bipolar disorder
21 GWAS hits omitted
LINC01722 SPTLC3 ISM1 TASP1 LOC102606466 ISM1−AS1 LINC01723
Position on chr20 (Mb)
−log10(p−value)
Recombination rate (cM/Mb)
r2

sphingomyelin (d18:1/19:0, d19:1/18:0)*

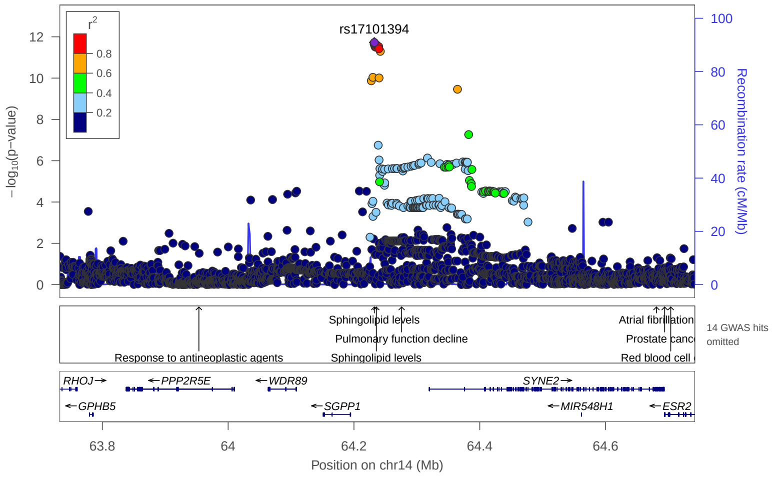

sphingomyelin (d18:1/19:0, d19:1/18:0)*

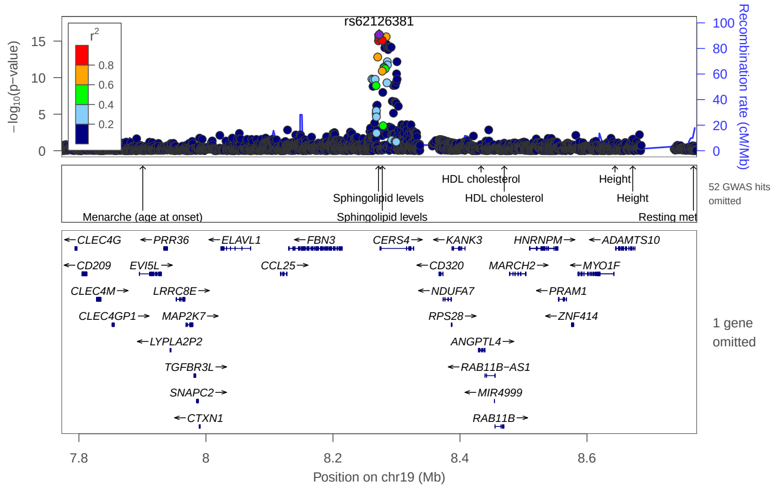

sphingomyelin (d18:1/19:0, d19:1/18:0)*

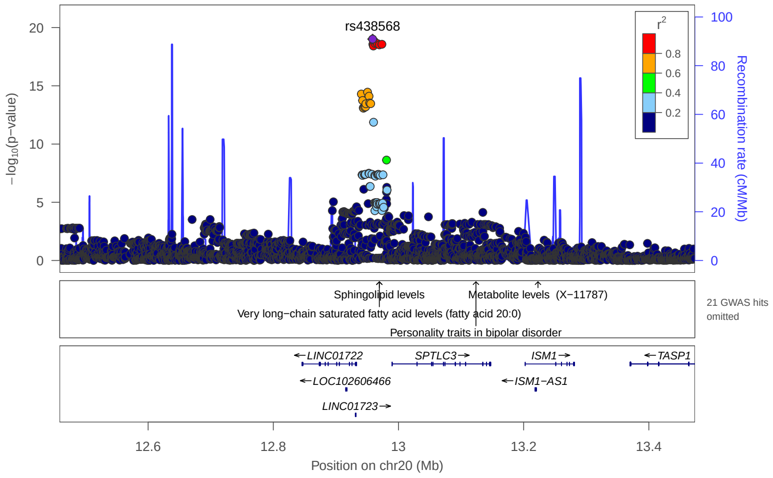

sphingomyelin (d18:0/20:0, d16:0/22:0)*

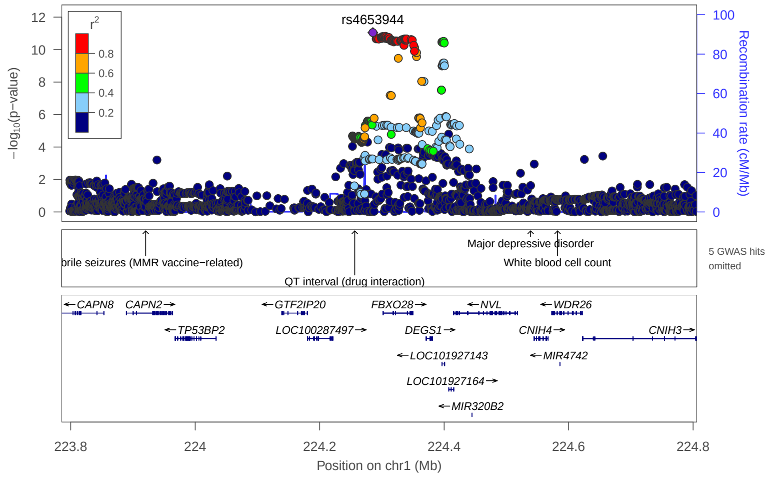

sphingomyelin (d18:0/20:0, d16:0/22:0)*

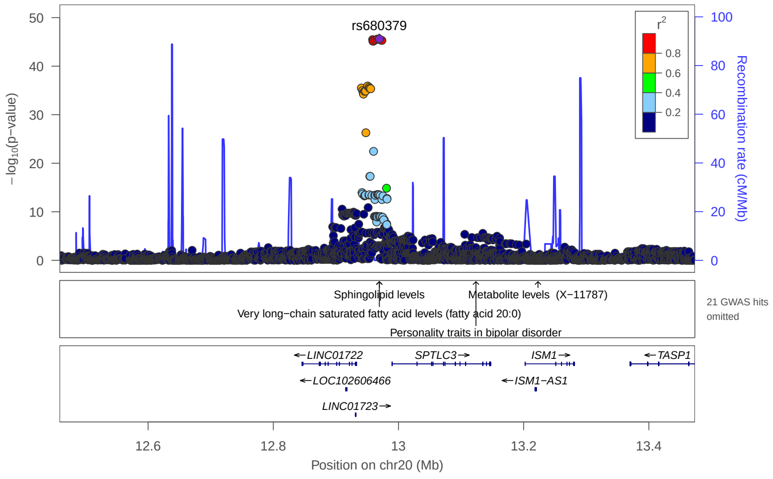

sphingomyelin (d18:1/22:2, d18:2/22:1, d16:1/24:2)*

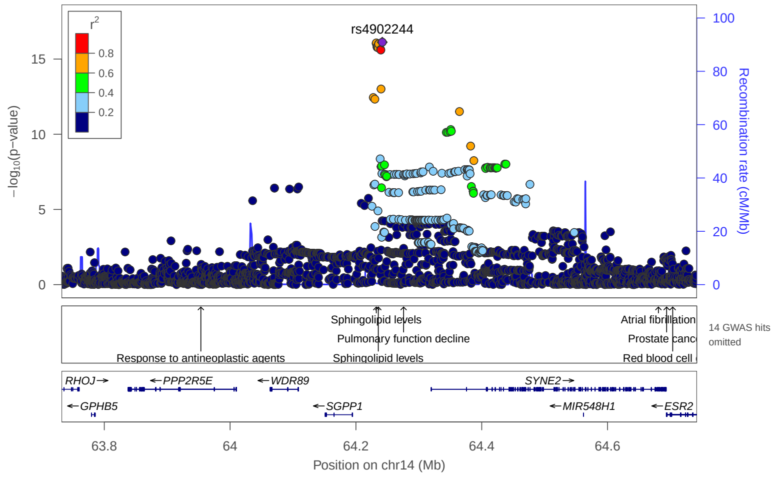

sphingomyelin (d18:1/22:2, d18:2/22:1, d16:1/24:2)*

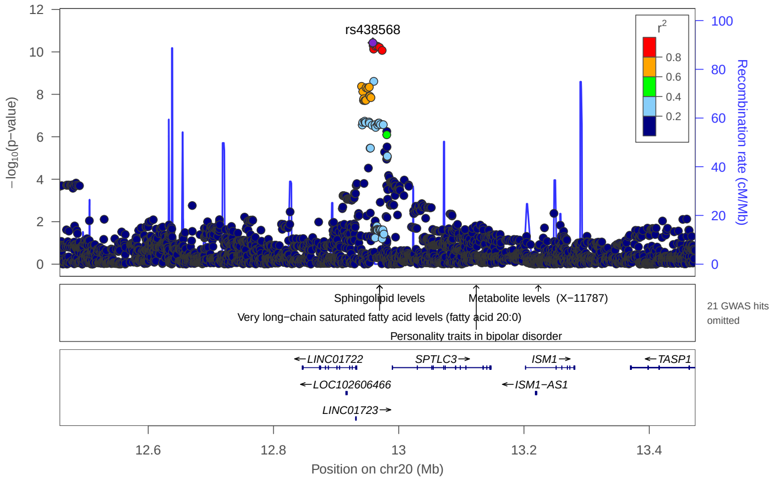

sphingomyelin (d18:1/25:0, d19:0/24:1, d20:1/23:0, d19:1/24:0)*

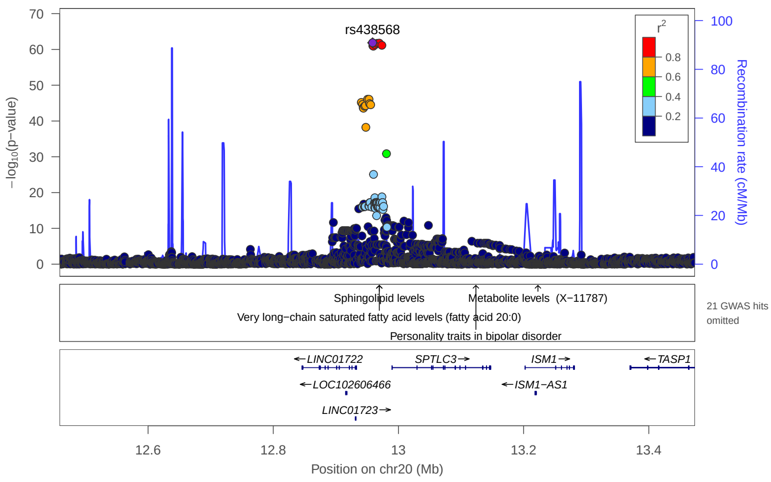

sphingomyelin (d18:2/21:0, d16:2/23:0)*

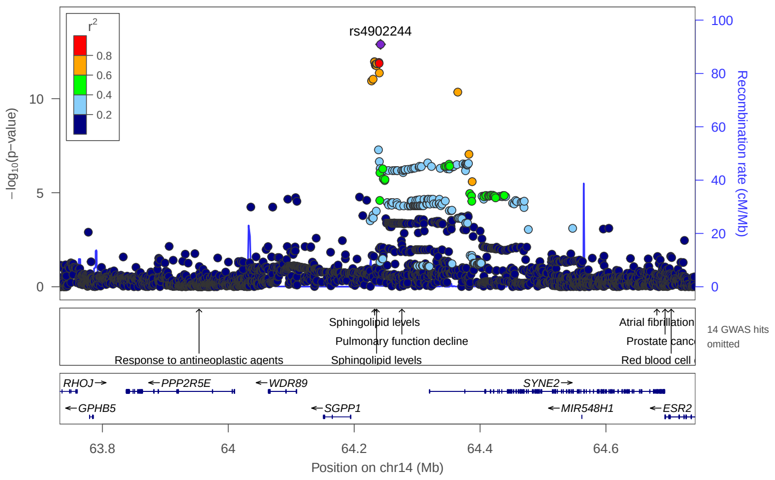

sphingomyelin (d17:2/16:0, d18:2/15:0)*

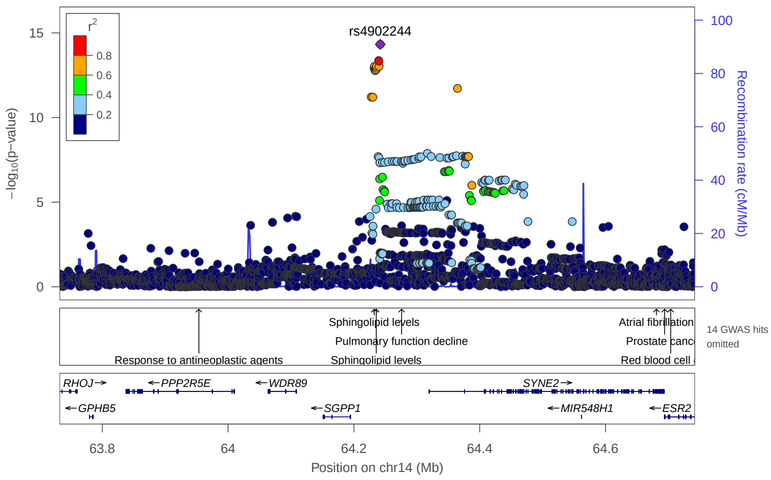

linolenoylcarnitine (C18:3)*

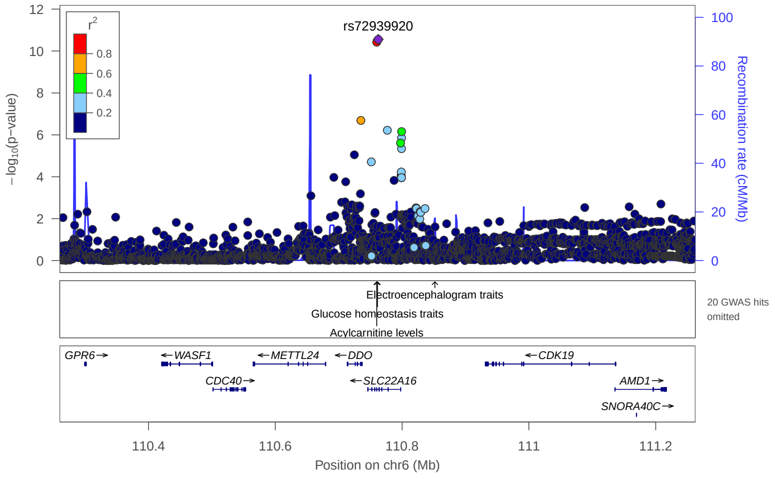

behenoylcarnitine (C22)*

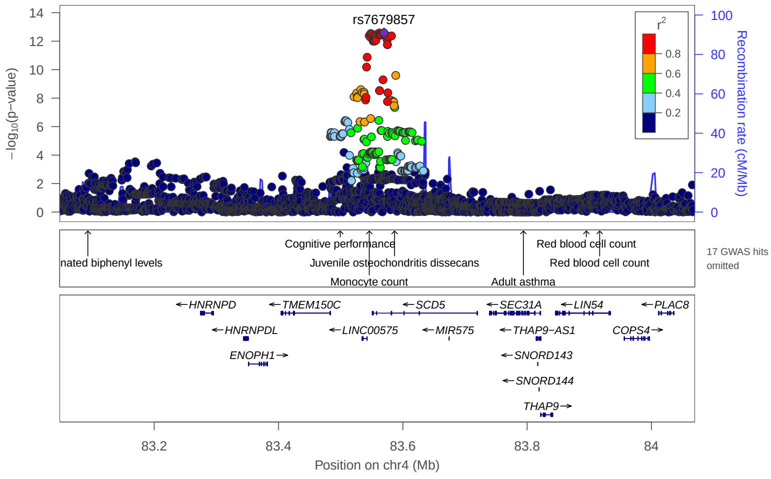

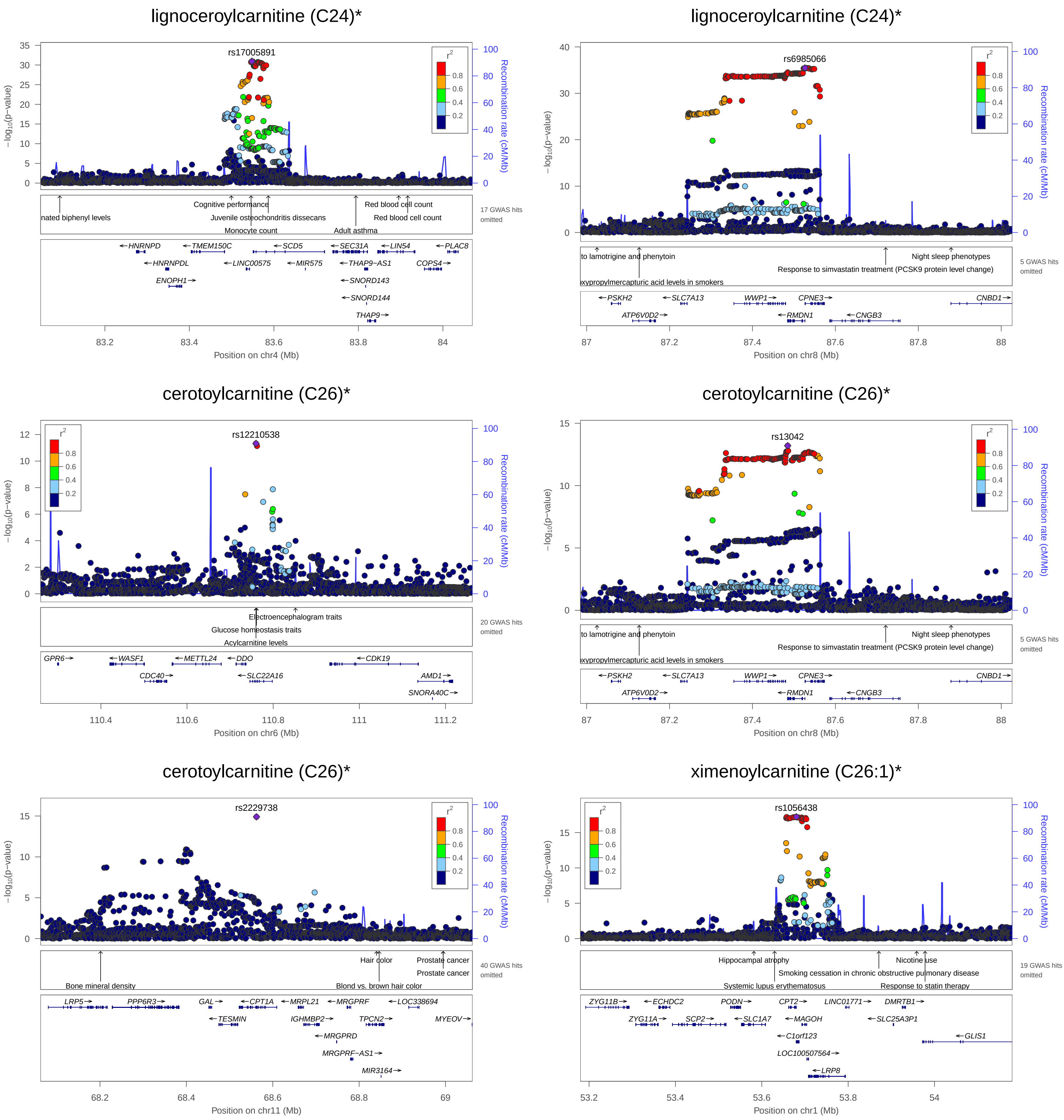
lignoceroylcarnitine (C24)*
rs17005891
−log10(p−value)
Recombination rate (cM/Mb)
r2
0.8
0.6
0.4
0.2
nated biphenyl levels
Cognitive performance
Juvenile osteochondritis dissecans
Monocyte count
Red blood cell count
Red blood cell count
Adult asthma
17 GWAS hits omitted
HNRNPD
TMEM150C
SCD5
SEC31A
LIN54
PLAC8
HNRNPDL
LINC00575
MIR575
THAP9−AS1
COPS4
ENOPH1
SNORD143
SNORD144
THAP9
Position on chr4 (Mb)
lignoceroylcarnitine (C24)*
rs6985066
to lamotrigine and phenytoin
Night sleep phenotypes
Response to simvastatin treatment (PCSK9 protein level change)
xypropylmercapturic acid levels in smokers
5 GWAS hits omitted
PSKH2
SLC7A13
WWP1
CPNE3
CNBD1
ATP6V0D2
RMDN1
CNGB3
Position on chr8 (Mb)
cerotoylcarnitine (C26)*
rs12210538
Electroencephalogram traits
Glucose homeostasis traits
Acylcarnitine levels
20 GWAS hits omitted
GPR6
WASF1
METTL24
DDO
CDK19
CDC40
SLC22A16
AMD1
SNORA40C
Position on chr6 (Mb)
cerotoylcarnitine (C26)*
rs13042
Position on chr8 (Mb)
cerotoylcarnitine (C26)*
rs2229738
Hair color
Prostate cancer
Prostate cancer
Bone mineral density
Blond vs. brown hair color
40 GWAS hits omitted
LRP5
PPP6R3
GAL
CPT1A
MRPL21
MRGPRF
LOC338694
TESMIN
IGHMBP2
TPCN2
MYEOV
MRGPRD
MRGPRF−AS1
MIR3164
Position on chr11 (Mb)
ximenoylcarnitine (C26:1)*
rs1056438
Hippocampal atrophy
Nicotine use
Smoking cessation in chronic obstructive pulmonary disease
Systemic lupus erythematosus
Response to statin therapy
19 GWAS hits omitted
ZYG11B
ECHDC2
PODN
CPT2
LINC01771
DMRTB1
ZYG11A
SCP2
SLC1A7
MAGOH
SLC25A3P1
C1orf123
GLIS1
LOC100507564
LRP8
Position on chr1 (Mb)

ximenoylcarnitine (C26:1)*

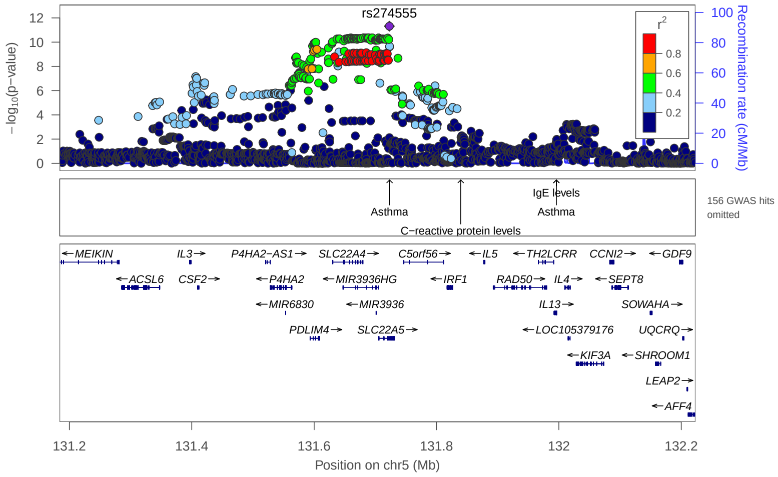

ximenoylcarnitine (C26:1)*

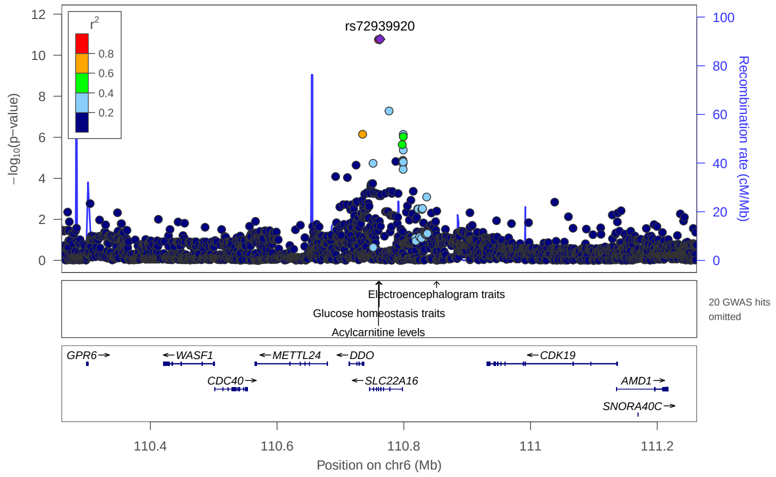

ximenoylcarnitine (C26:1)*

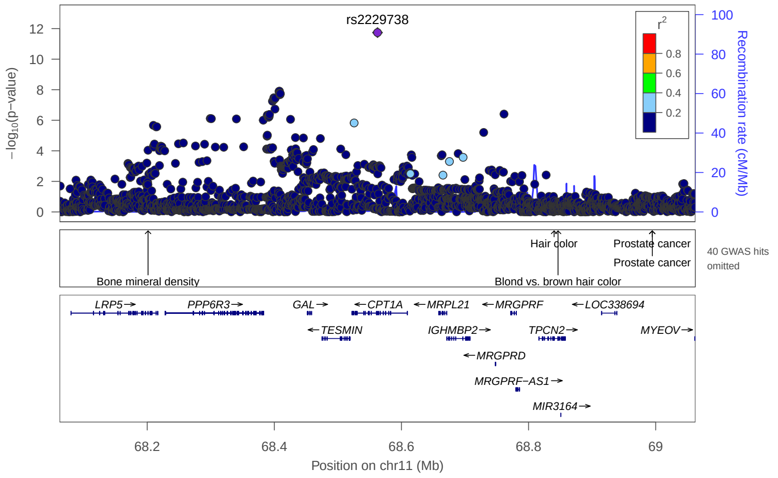

arachidonoylcarnitine (C20:4)

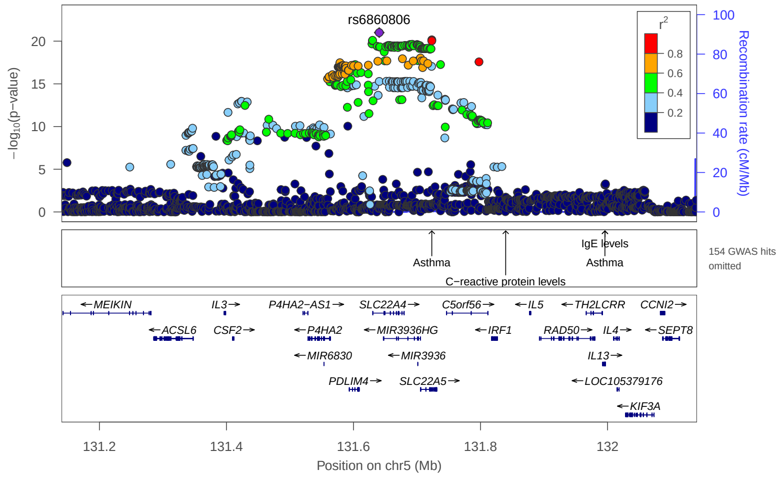

arachidonoylcarnitine (C20:4)

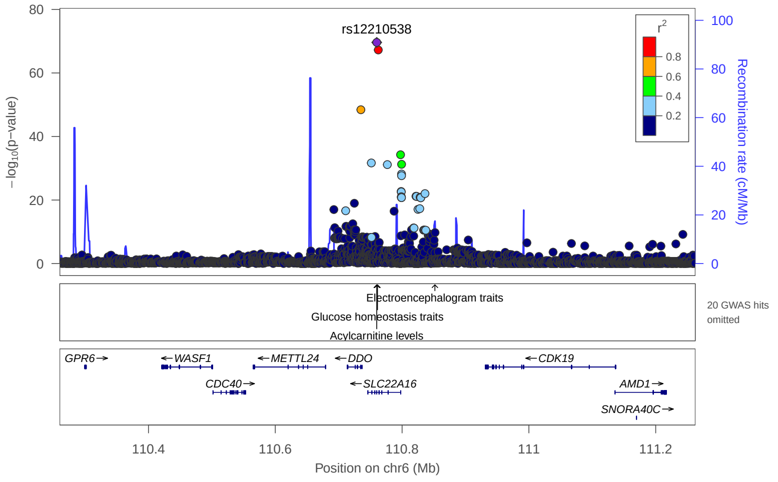

arachidonoylcarnitine (C20:4)

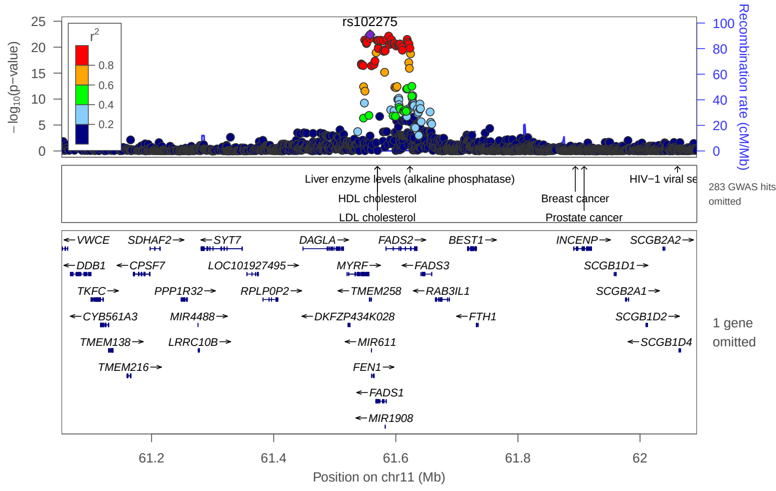

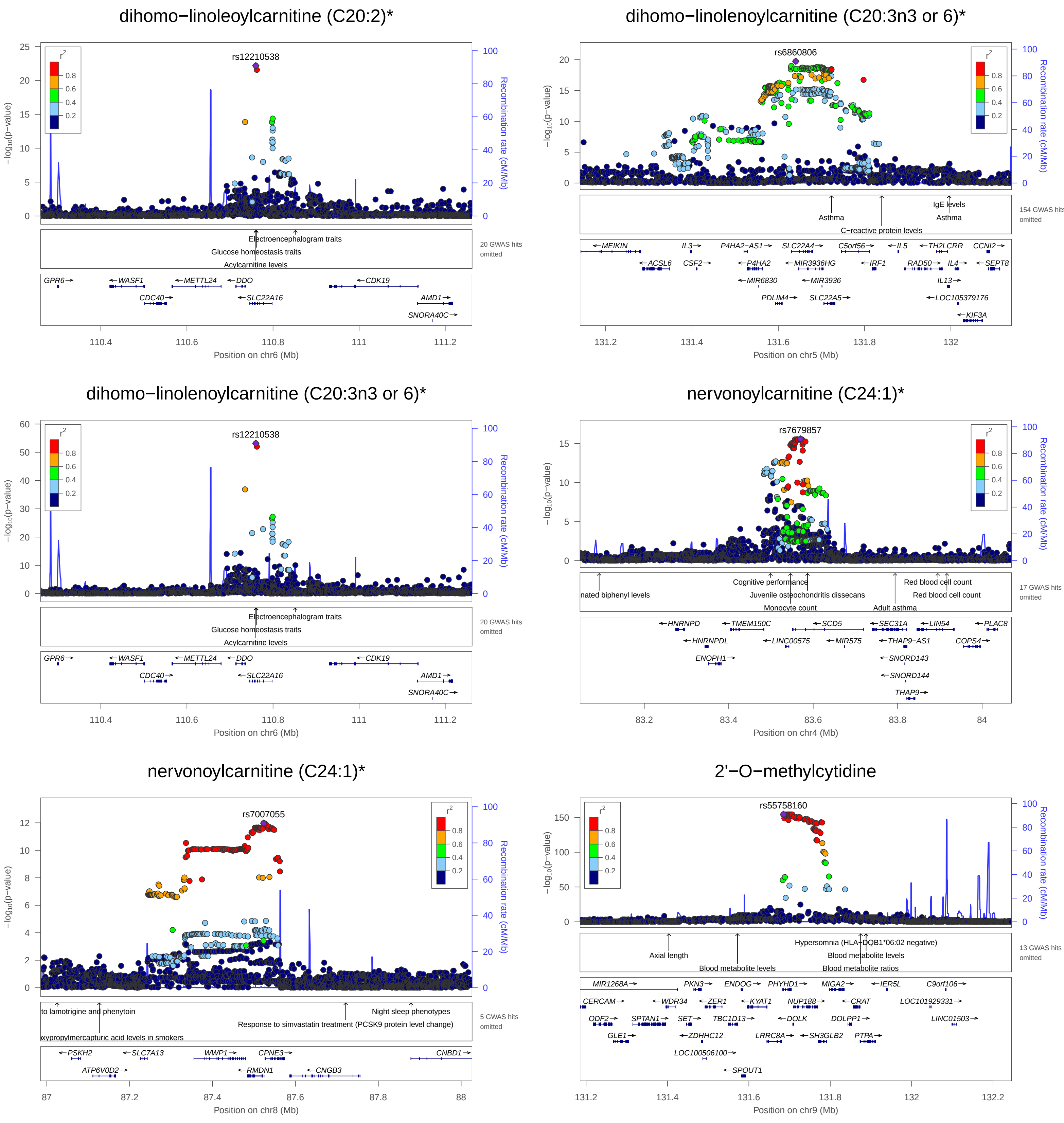

dihomo−linoleoylcarnitine (C20:2)*
rs12210538
Electroencephalogram traits
Glucose homeostasis traits
Acylcarnitine levels
20 GWAS hits omitted
GPR6 WASF1 METTL24 DDO CDK19 CDC40 SLC22A16 AMD1 SNORA40C
Position on chr6 (Mb)
dihomo−linolenoylcarnitine (C20:3n3 or 6)*
rs6860806
IgE levels
Asthma
Asthma
C−reactive protein levels
154 GWAS hits omitted
MEIKIN IL3 P4HA2−AS1 SLC22A4 C5orf56 IL5 TH2LCRR CCNI2 ACSL6 CSF2 P4HA2 MIR3936HG IRF1 RAD50 IL4 SEPT8 MIR6830 MIR3936 IL13 PDLIM4 SLC22A5 LOC105379176 KIF3A
Position on chr5 (Mb)
dihomo−linolenoylcarnitine (C20:3n3 or 6)*
rs12210538
Electroencephalogram traits
Glucose homeostasis traits
Acylcarnitine levels
20 GWAS hits omitted
GPR6 WASF1 METTL24 DDO CDK19 CDC40 SLC22A16 AMD1 SNORA40C
Position on chr6 (Mb)
nervonoylcarnitine (C24:1)*
rs7679857
Cognitive performance
nated biphenyl levels
Juvenile osteochondritis dissecans
Monocyte count
Red blood cell count
Red blood cell count
Adult asthma
17 GWAS hits omitted
HNRNPD TMEM150C SCD5 SEC31A LIN54 PLAC8 HNRNPDL LINC00575 MIR575 THAP9−AS1 COPS4 ENOPH1 SNORD143 SNORD144 THAP9
Position on chr4 (Mb)
nervonoylcarnitine (C24:1)*
rs7007055
to lamotrigine and phenytoin
xypropylmercapturic acid levels in smokers
Night sleep phenotypes
Response to simvastatin treatment (PCSK9 protein level change)
5 GWAS hits omitted
PSKH2 SLC7A13 WWP1 CPNE3 CNBD1 ATP6V0D2 RMDN1 CNGB3
Position on chr8 (Mb)
2'−O−methylcytidine
rs55758160
Axial length
Blood metabolite levels
Hypersomnia (HLA−DQB1*06:02 negative)
Blood metabolite levels
Blood metabolite ratios
13 GWAS hits omitted
MIR1268A PKN3 ENDOG PHYHD1 MIGA2 IER5L C9orf106 CERCAM WDR34 ZER1 KYAT1 NUP188 CRAT LOC101929331 ODF2 SPTAN1 SET TBC1D13 DOLK DOLPP1 LINC01503 GLE1 ZDHHC12 LRRC8A SH3GLB2 PTPA LOC100506100 SPOUT1
Position on chr9 (Mb)
−log10(p−value)
Recombination rate (cM/Mb)

glycosyl ceramide (d18:1/20:0, d16:1/22:0)*

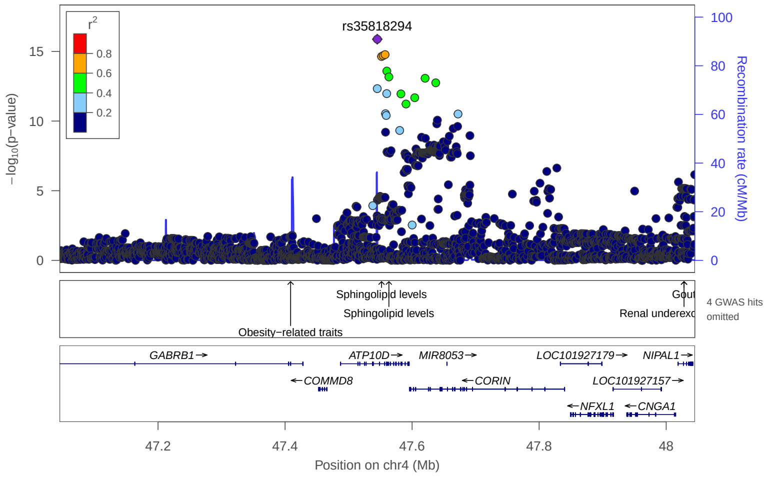

glycosyl ceramide (d18:1/20:0, d16:1/22:0)*

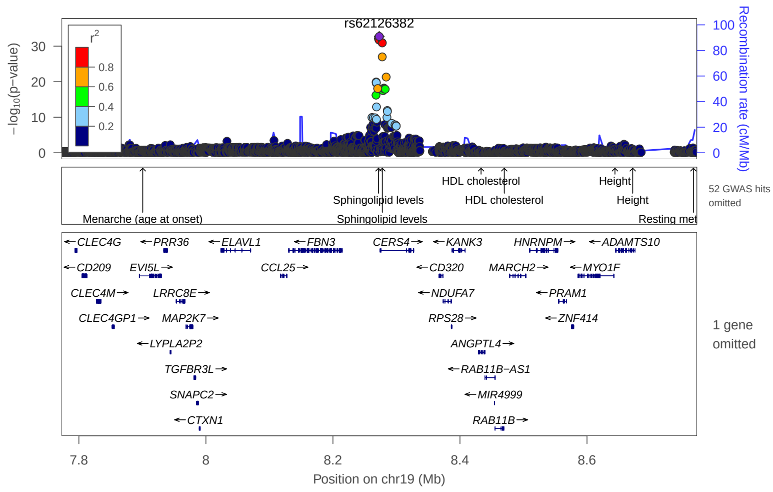

carotene diol (1)

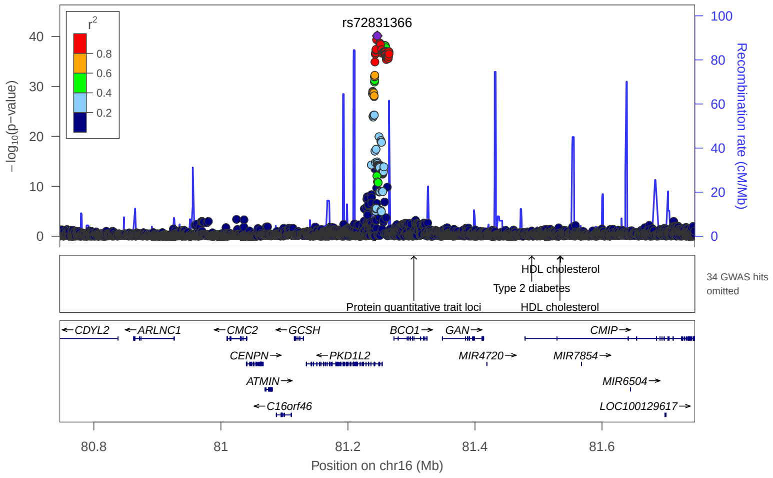

carotene diol (2)

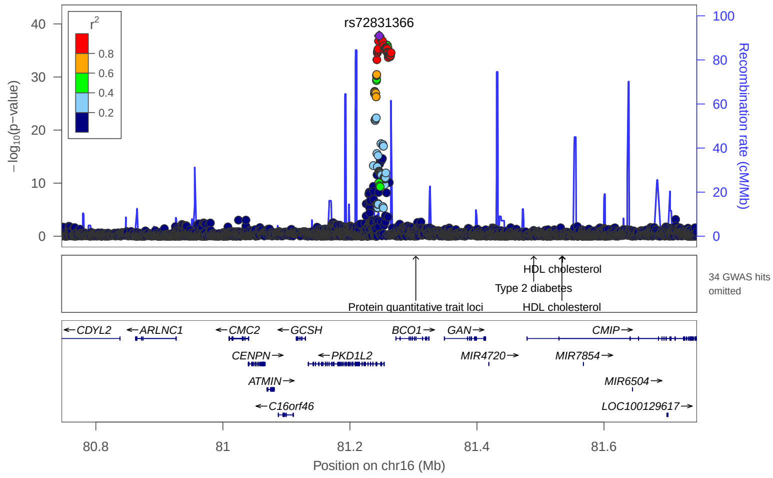

carotene diol (3)

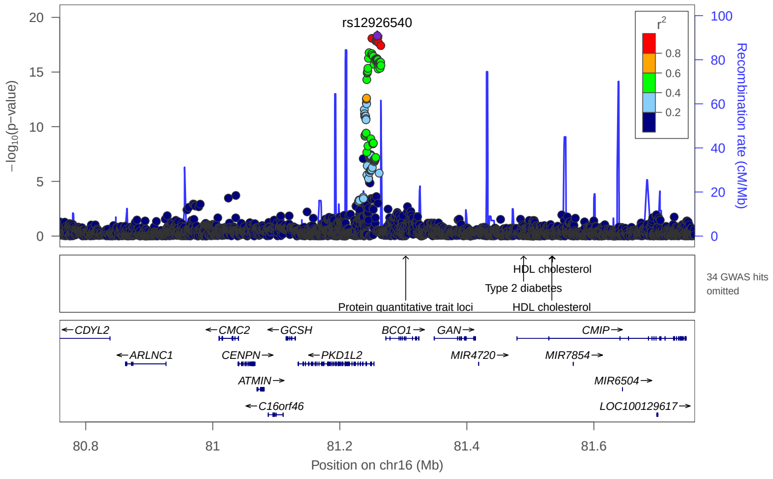

2'−O−methyluridine

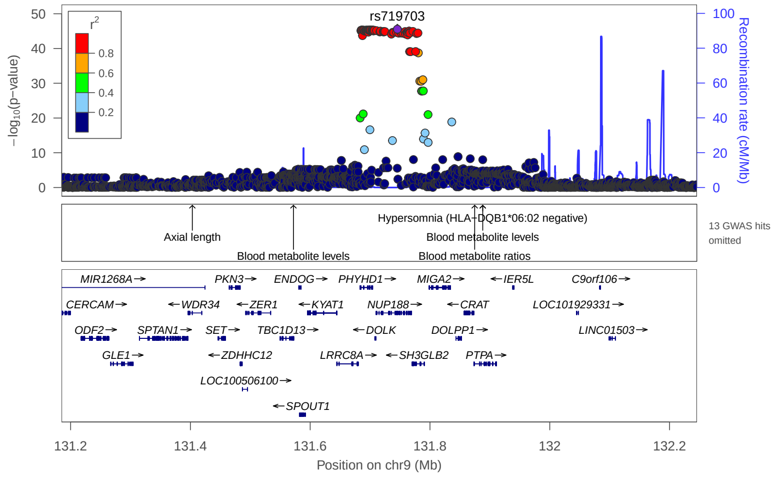

N−oleoylserine

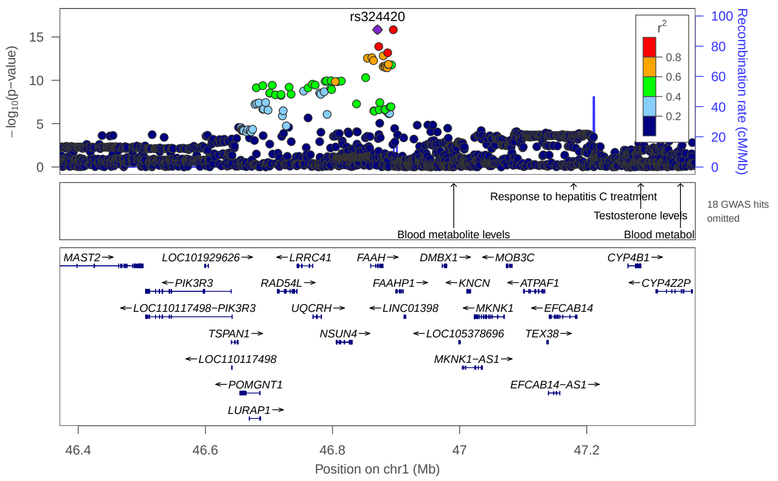

N,N,N−trimethyl−5−aminovalerate

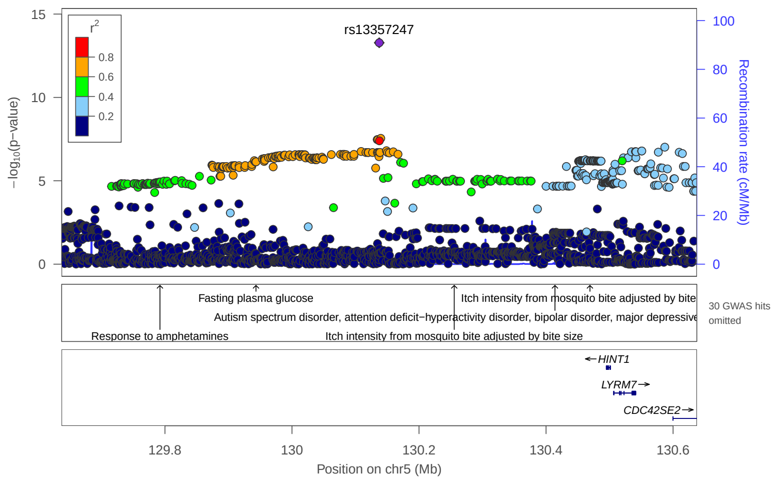

N,N,N−trimethyl−5−aminovalerate

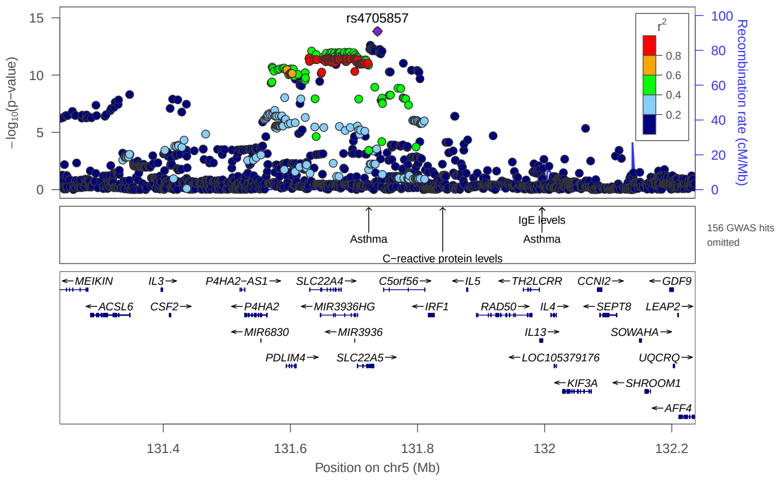

N('1)−acetylspermidine

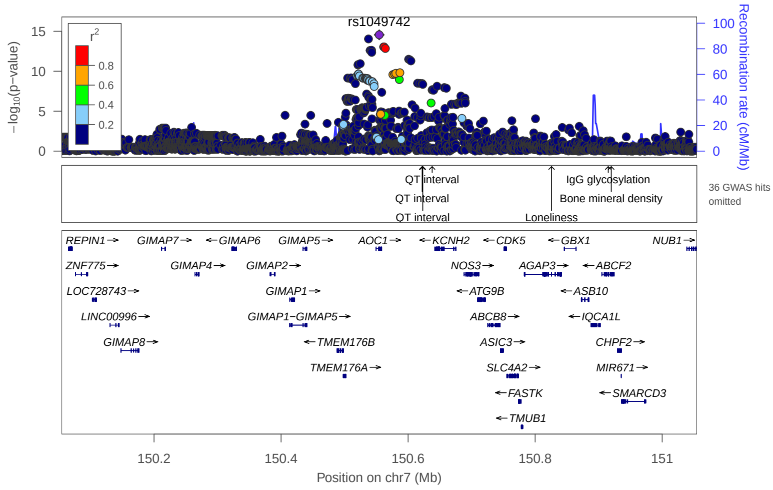

N('1)−acetylspermidine

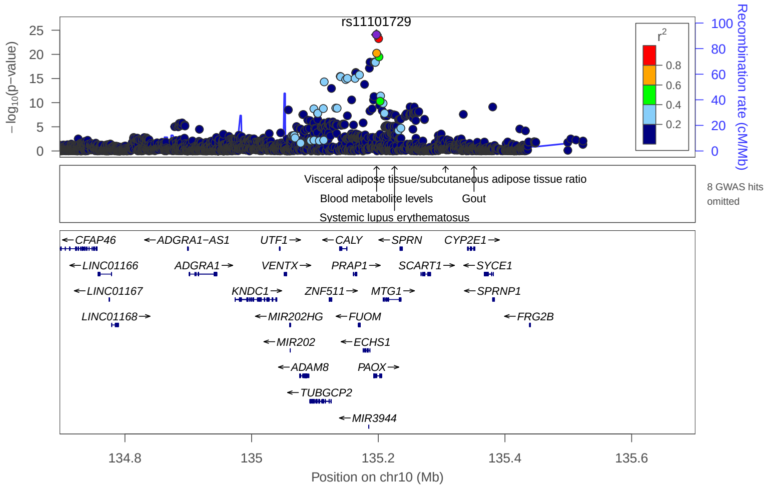

N('1)−acetylspermidine

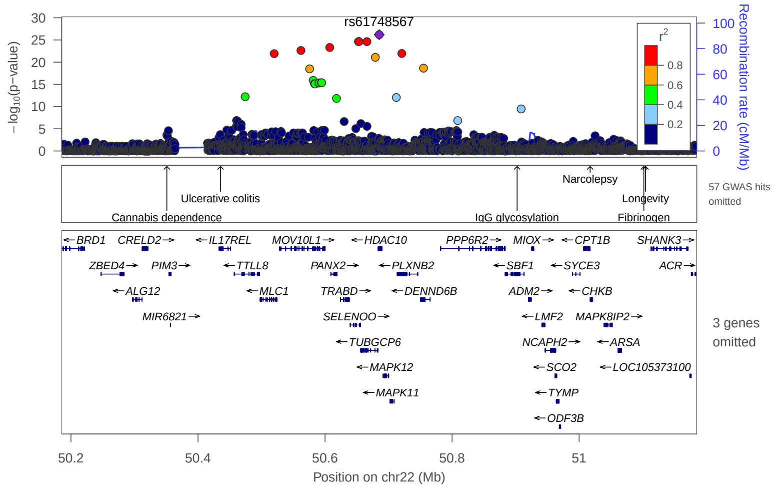

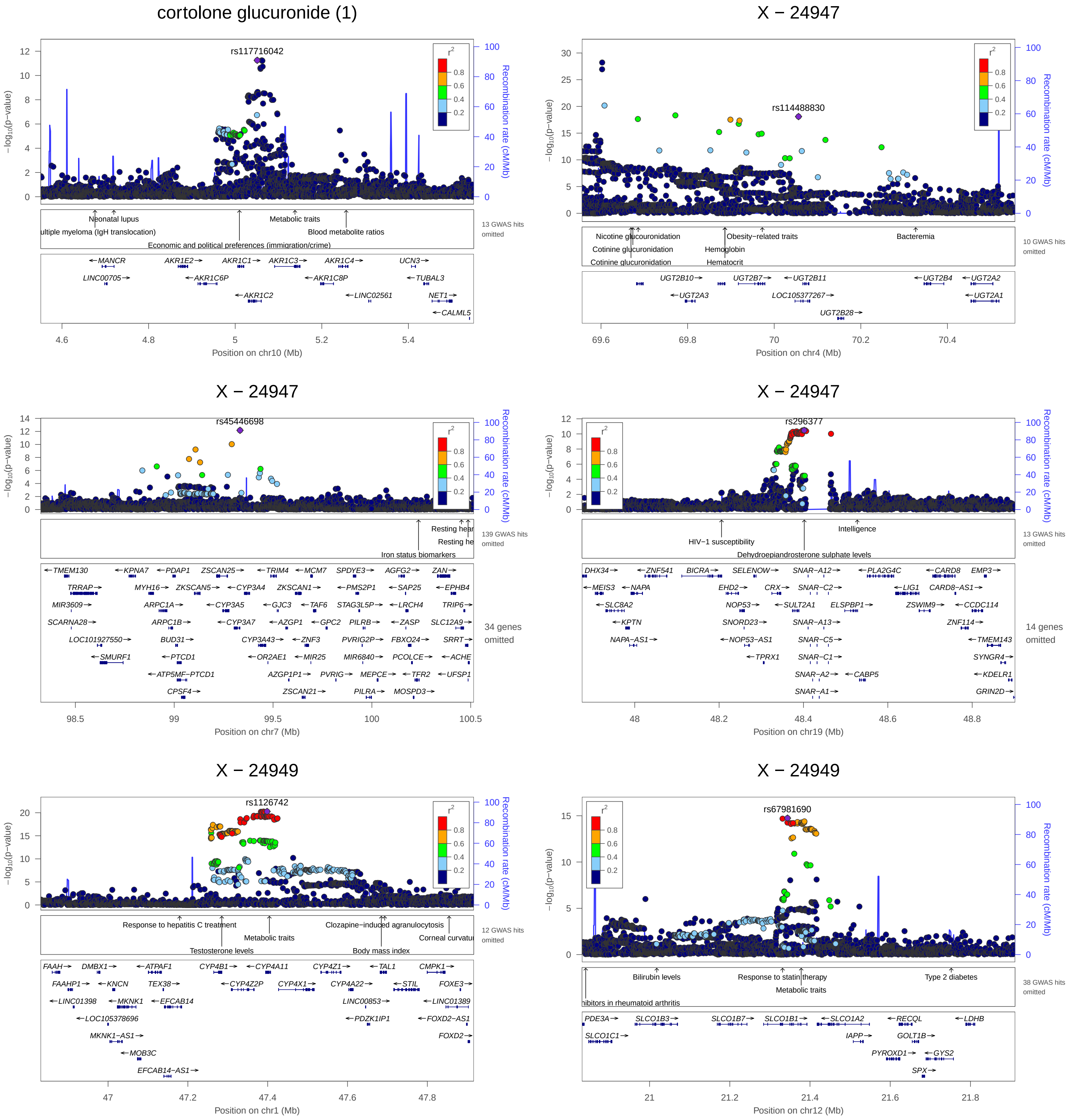
cortolone glucuronide (1)
rs117716042
Neonatal lupus
Multiple myeloma (IgH translocation)
Metabolic traits
Blood metabolite ratios
Economic and political preferences (immigration/crime)
13 GWAS hits omitted
MANCR
LINC00705
AKR1E2
AKR1C6P
AKR1C1
AKR1C2
AKR1C3
AKR1C4
AKR1C8P
LINC02561
UCN3
TUBAL3
NET1
CALML5
Position on chr10 (Mb)
X − 24947
rs114488830
Nicotine glucouronidation
Cotinine glucuronidation
Obesity−related traits
Hemoglobin
Hematocrit
Bacteremia
10 GWAS hits omitted
UGT2B10
UGT2A3
UGT2B7
UGT2B11
LOC105377267
UGT2B28
UGT2B4
UGT2A2
UGT2A1
Position on chr4 (Mb)
X − 24947
rs45446698
Resting heart
Iron status biomarkers
139 GWAS hits omitted
34 genes omitted
Position on chr7 (Mb)
X − 24947
rs296377
HIV−1 susceptibility
Intelligence
Dehydroepiandrosterone sulphate levels
13 GWAS hits omitted
14 genes omitted
Position on chr19 (Mb)
X − 24949
rs1126742
Response to hepatitis C treatment
Metabolic traits
Testosterone levels
Clozapine−induced agranulocytosis
Body mass index
Corneal curvature
12 GWAS hits omitted
Position on chr1 (Mb)
X − 24949
rs67981690
Bilirubin levels
Response to statin therapy
Metabolic traits
Type 2 diabetes
38 GWAS hits omitted
Position on chr12 (Mb)
−log10(p−value)
Recombination rate (cM/Mb)

glycocholenate sulfate*

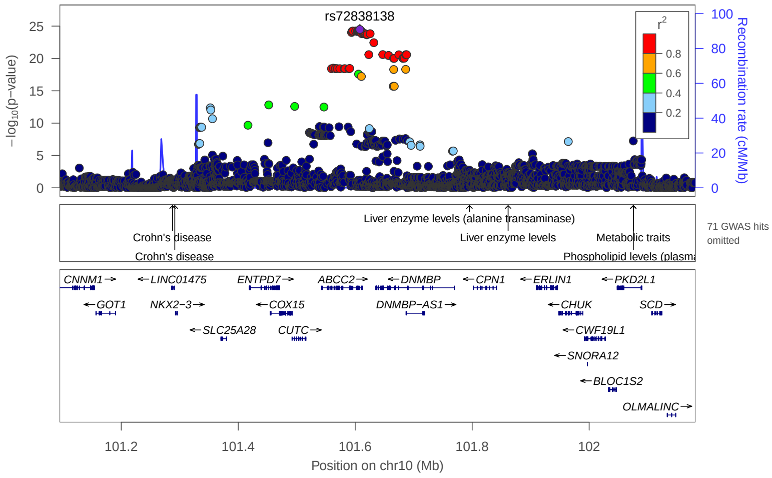

glycocholenate sulfate*

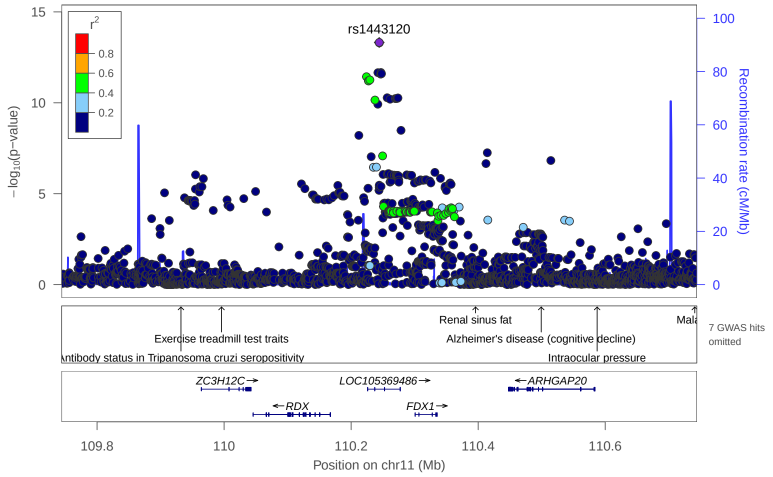

glycocholenate sulfate*

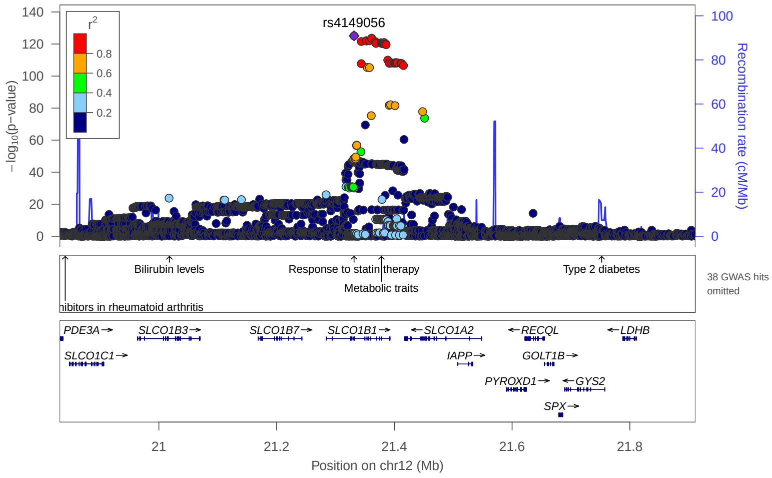

3−methylglutaconate

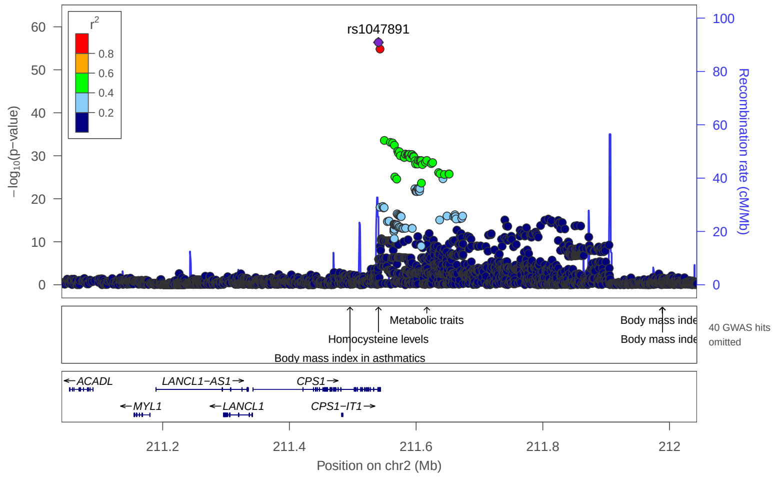

3−methylglutaconate

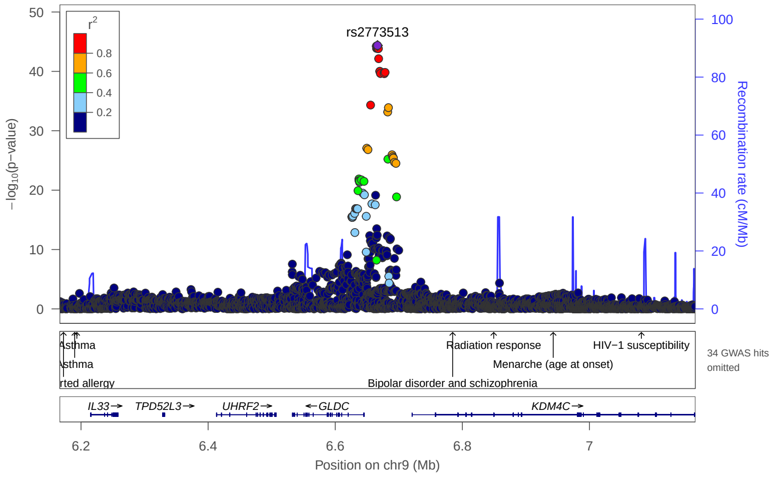

gluconate

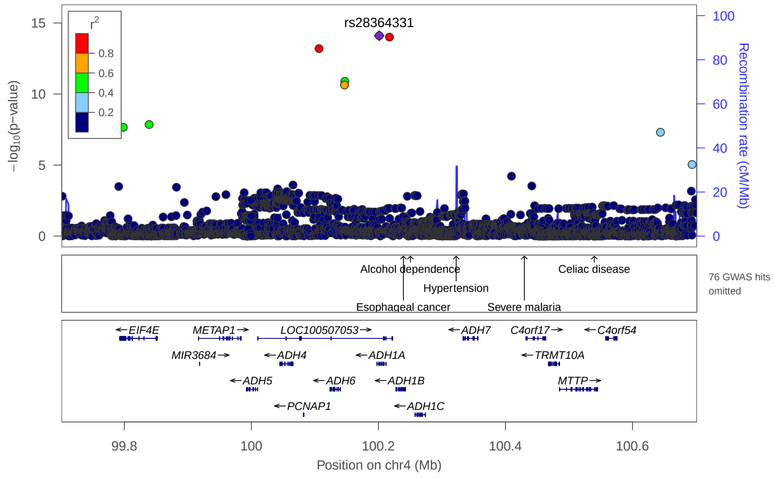

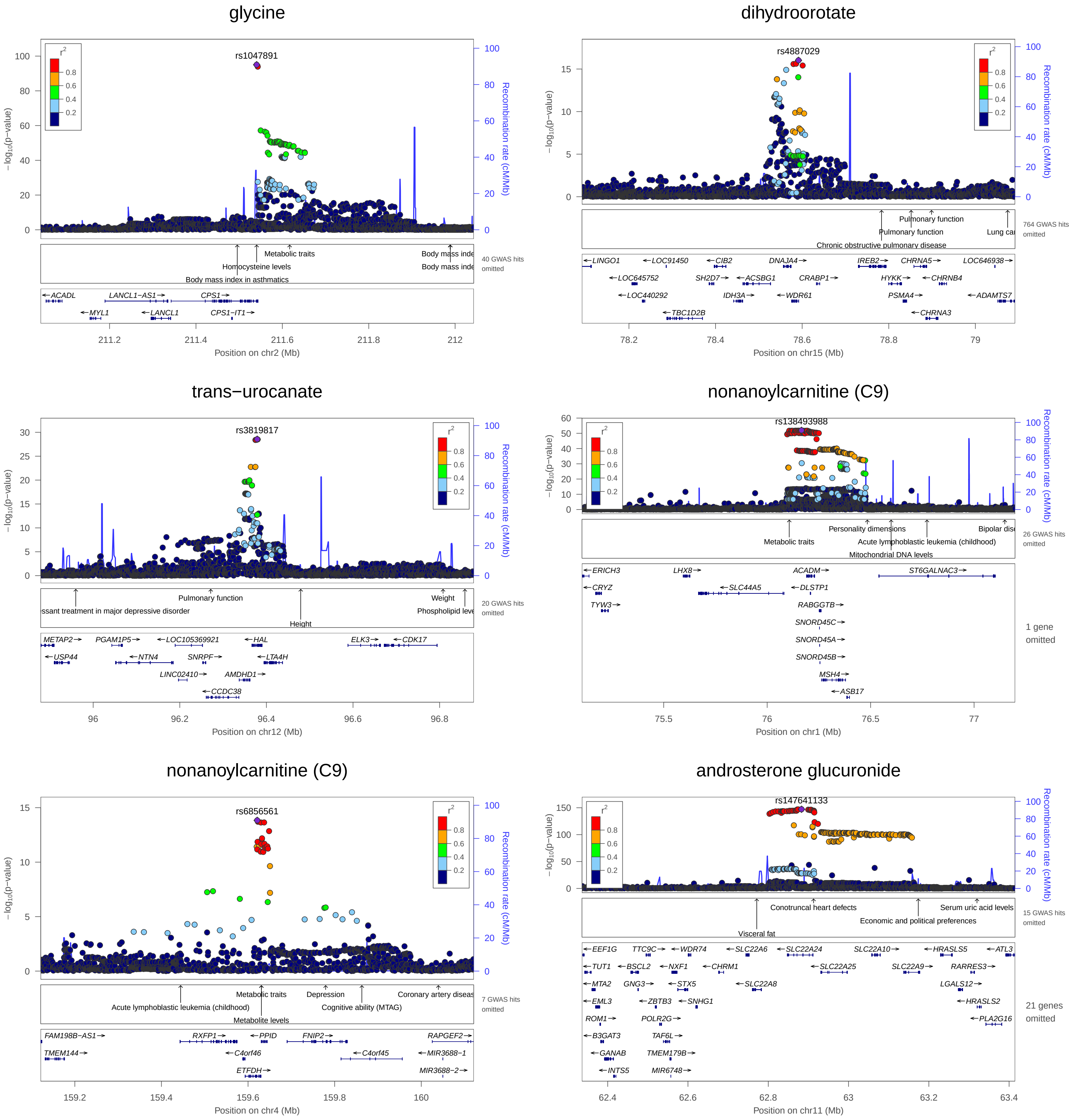

glycine
rs1047891
−log10(p−value)
Recombination rate (cM/Mb)
Metabolic traits
Homocysteine levels
Body mass index in asthmatics
Body mass inde
40 GWAS hits omitted
ACADL
LANCL1−AS1
CPS1
MYL1
LANCL1
CPS1−IT1
Position on chr2 (Mb)
dihydroorotate
rs4887029
Pulmonary function
Chronic obstructive pulmonary disease
Lung ca
764 GWAS hits omitted
LINGO1
LOC91450
CIB2
DNAJA4
IREB2
CHRNA5
LOC646938
LOC645752
SH2D7
ACSBG1
CRABP1
HYKK
CHRNB4
LOC440292
IDH3A
WDR61
PSMA4
ADAMTS7
TBC1D2B
CHRNA3
Position on chr15 (Mb)
trans−urocanate
rs3819817
Pulmonary function
ssant treatment in major depressive disorder
Height
Weight
Phospholipid leve
20 GWAS hits omitted
METAP2
PGAM1P5
LOC105369921
HAL
ELK3
CDK17
USP44
NTN4
SNRPF
LTA4H
LINC02410
AMDHD1
CCDC38
Position on chr12 (Mb)
nonanoylcarnitine (C9)
rs138493988
Personality dimensions
Metabolic traits
Acute lymphoblastic leukemia (childhood)
Mitochondrial DNA levels
Bipolar dis
26 GWAS hits omitted
ERICH3
LHX8
ACADM
ST6GALNAC3
CRYZ
SLC44A5
DLSTP1
TYW3
RABGGTB
SNORD45C
SNORD45A
SNORD45B
MSH4
ASB17
1 gene omitted
Position on chr1 (Mb)
nonanoylcarnitine (C9)
rs6856561
Metabolic traits
Depression
Coronary artery diseas
Acute lymphoblastic leukemia (childhood)
Cognitive ability (MTAG)
Metabolite levels
7 GWAS hits omitted
FAM198B−AS1
RXFP1
PPID
FNIP2
RAPGEF2
TMEM144
C4orf46
C4orf45
MIR3688−1
ETFDH
MIR3688−2
Position on chr4 (Mb)
androsterone glucuronide
rs147641133
Conotruncal heart defects
Serum uric acid levels
Economic and political preferences
Visceral fat
15 GWAS hits omitted
EEF1G
TTC9C
WDR74
SLC22A6
SLC22A24
SLC22A10
HRASLS5
ATL3
TUT1
BSCL2
NXF1
CHRM1
SLC22A25
SLC22A9
RARRES3
MTA2
GNG3
STX5
SLC22A8
LGALS12
EML3
ZBTB3
SNHG1
HRASLS2
ROM1
POLR2G
PLA2G16
B3GAT3
TAF6L
GANAB
TMEM179B
INTS5
MIR6748
21 genes omitted
Position on chr11 (Mb)

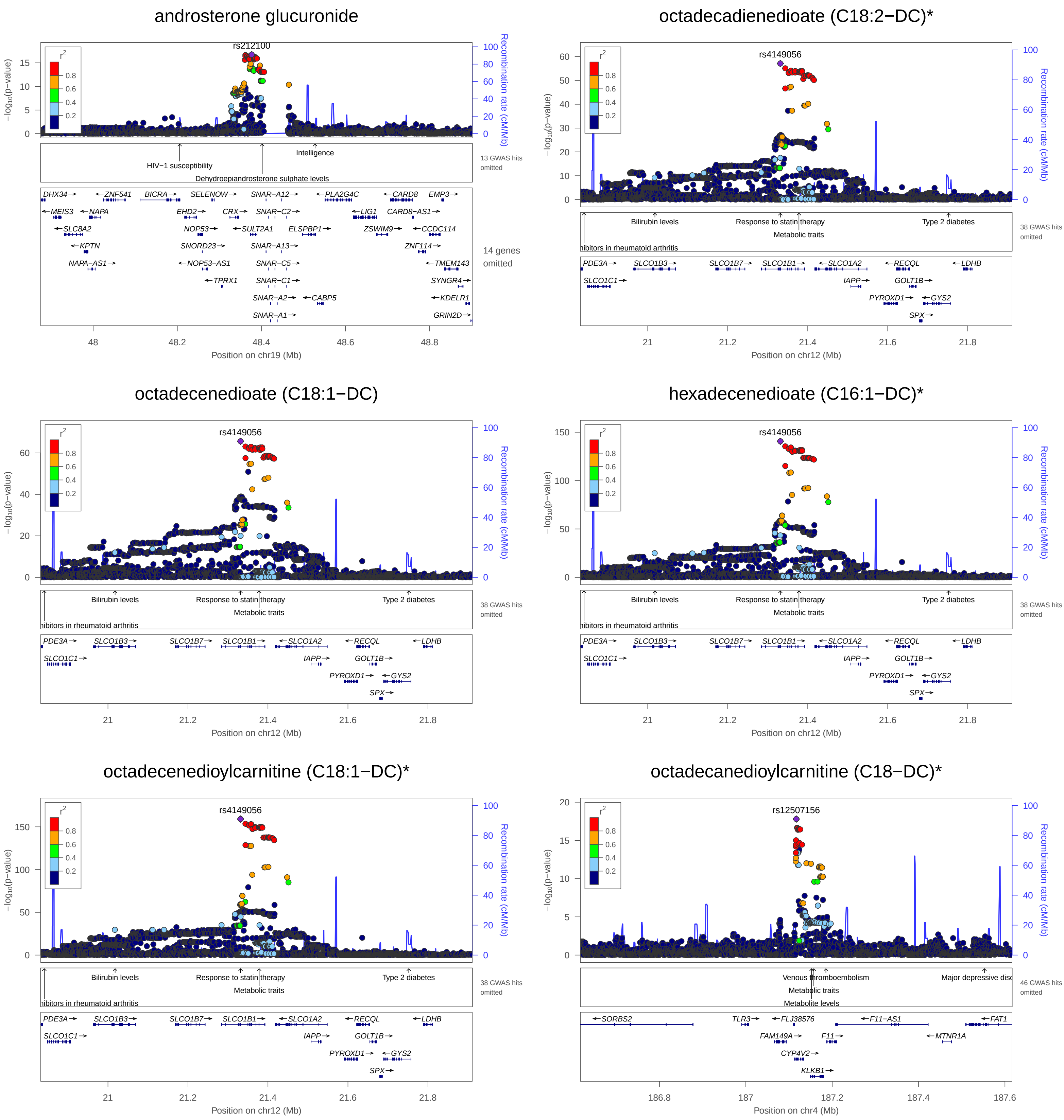

androsterone glucuronide
rs212100
−log10(p−value)
Recombination rate (cM/Mb)
HIV−1 susceptibility
Intelligence
Dehydroepiandrosterone sulphate levels
13 GWAS hits omitted
DHX34 ZNF541 BICRA SELENOW SNAR−A12 PLA2G4C CARD8 EMP3
MEIS3 NAPA EHD2 CRX SNAR−C2 LIG1 CARD8−AS1
SLC8A2 NOP53 SULT2A1 ELSPBP1 ZSWIM9 CCDC114
KPTN SNORD23 SNAR−A13 ZNF114
NAPA−AS1 NOP53−AS1 SNAR−C5 TMEM143
TPRX1 SNAR−C1 SYNGR4
SNAR−A2 CABP5 KDELR1
SNAR−A1 GRIN2D
14 genes omitted
Position on chr19 (Mb)
octadecadienedioate (C18:2−DC)*
rs4149056
Bilirubin levels
Response to statin therapy
Metabolic traits
Type 2 diabetes
nhibitors in rheumatoid arthritis
38 GWAS hits omitted
PDE3A SLCO1B3 SLCO1B7 SLCO1B1 SLCO1A2 RECQL LDHB
SLCO1C1 IAPP GOLT1B
PYROXD1 GYS2
SPX
Position on chr12 (Mb)
octadecenedioate (C18:1−DC)
rs4149056
Position on chr12 (Mb)
hexadecenedioate (C16:1−DC)*
rs4149056
Position on chr12 (Mb)
octadecenedioylcarnitine (C18:1−DC)*
rs4149056
Position on chr12 (Mb)
octadecanedioylcarnitine (C18−DC)*
rs12507156
Venous thromboembolism
Metabolic traits
Metabolite levels
Major depressive disc
46 GWAS hits omitted
SORBS2 TLR3 FLJ38576 F11−AS1 FAT1
FAM149A F11 MTNR1A
CYP4V2
KLKB1
Position on chr4 (Mb)

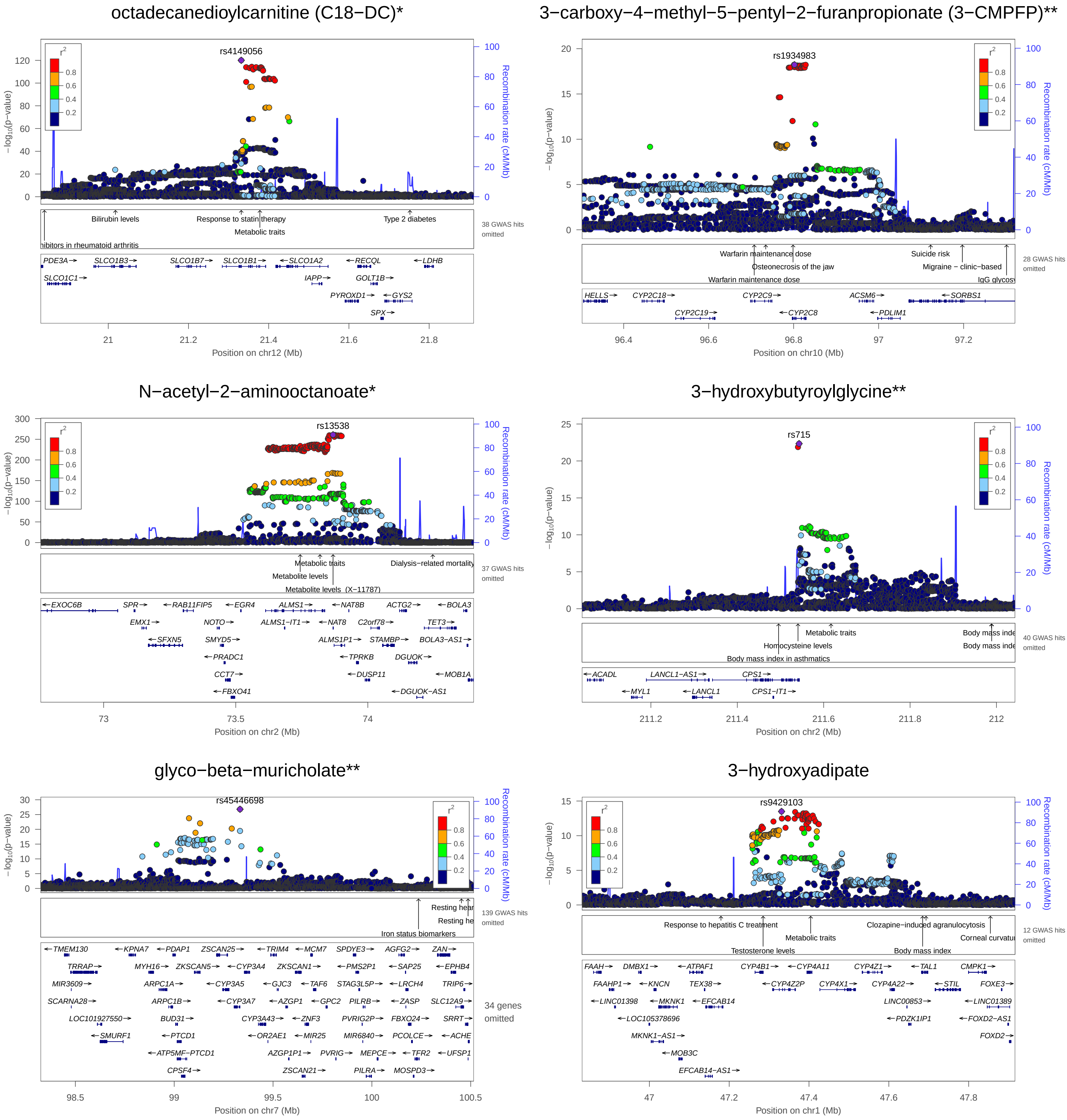

octadecanedioylcarnitine (C18−DC)*
rs4149056
Position on chr12 (Mb)
38 GWAS hits omitted
Bilirubin levels
Response to statin therapy
Metabolic traits
Type 2 diabetes
PDE3A
SLCO1B3
SLCO1B7
SLCO1B1
SLCO1A2
RECQL
LDHB
SLCO1C1
IAPP
GOLT1B
PYROXD1
GYS2
SPX
3−carboxy−4−methyl−5−pentyl−2−furanpropionate (3−CMPFP)**
rs1934983
Position on chr10 (Mb)
28 GWAS hits omitted
Warfarin maintenance dose
Osteonecrosis of the jaw
Suicide risk
Migraine − clinic−based
HELLS
CYP2C18
CYP2C19
CYP2C9
CYP2C8
ACSM6
PDLIM1
SORBS1
N−acetyl−2−aminooctanoate*
rs13538
Position on chr2 (Mb)
37 GWAS hits omitted
Metabolic traits
Metabolite levels
Metabolite levels (X−11787)
Dialysis−related mortality
EXOC6B
SPR
EMX1
SFXN5
RAB11FIP5
NOTO
SMYD5
PRADC1
CCT7
FBXO41
EGR4
ALMS1
ALMS1−IT1
ALMS1P1
NAT8
TPRKB
DUSP11
C2orf78
STAMBP
ACTG2
DGUOK
DGUOK−AS1
BOLA3
TET3
BOLA3−AS1
MOB1A
3−hydroxybutyroylglycine**
rs715
Position on chr2 (Mb)
40 GWAS hits omitted
Metabolic traits
Homocysteine levels
Body mass index in asthmatics
ACADL
MYL1
LANCL1−AS1
LANCL1
CPS1
CPS1−IT1
glyco−beta−muricholate**
rs45446698
Position on chr7 (Mb)
139 GWAS hits omitted
Iron status biomarkers
34 genes omitted
TMEM130
TRRAP
MIR3609
SCARNA28
LOC101927550
SMURF1
KPNA7
MYH16
ARPC1A
ARPC1B
BUD31
PTCD1
ATP5MF−PTCD1
CPSF4
PDAP1
ZKSCAN5
ZSCAN25
CYP3A4
CYP3A5
CYP3A7
CYP3A43
OR2AE1
AZGP1P1
ZSCAN21
TRIM4
ZKSCAN1
GJC3
AZGP1
ZNF3
MIR25
MCM7
TAF6
GPC2
PVRIG2P
MIR6840
PVRIG
SPDYE3
PMS2P1
STAG3L5P
PILRB
FBXO24
PCOLCE
MEPCE
PILRA
MOSPD3
AGFG2
SAP25
LRCH4
ZASP
TFR2
ZAN
EPHB4
TRIP6
SLC12A9
SRRT
ACHE
UFSP1
3−hydroxyadipate
rs9429103
Position on chr1 (Mb)
12 GWAS hits omitted
Response to hepatitis C treatment
Metabolic traits
Testosterone levels
Clozapine−induced agranulocytosis
Body mass index
FAAH
FAAHP1
LINC01398
LOC105378696
DMBX1
KNCN
MKNK1
MKNK1−AS1
MOB3C
ATPAF1
TEX38
EFCAB14
EFCAB14−AS1
CYP4B1
CYP4Z2P
CYP4A11
CYP4X1
CYP4Z1
CYP4A22
LINC00853
PDZK1IP1
TAL1
STIL
CMPK1
FOXE3
LINC01389
FOXD2−AS1
FOXD2
−log10(p−value)
Recombination rate (cM/Mb)
r2

alpha−ketoglutaramate*

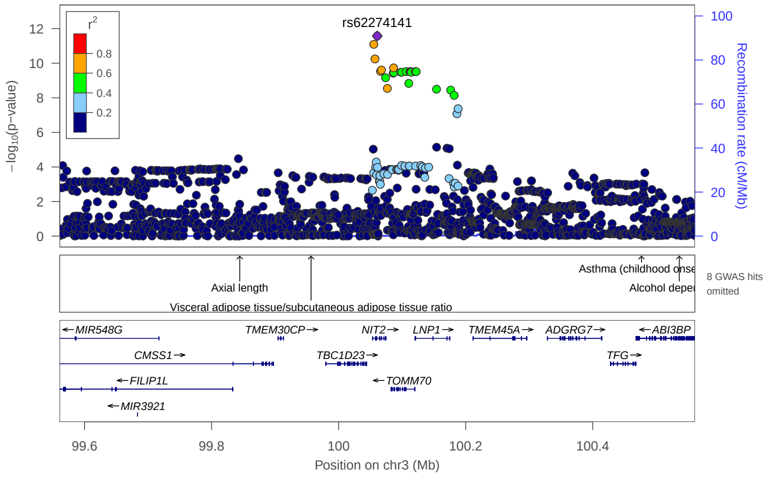

cysteinylglycine disulfide*

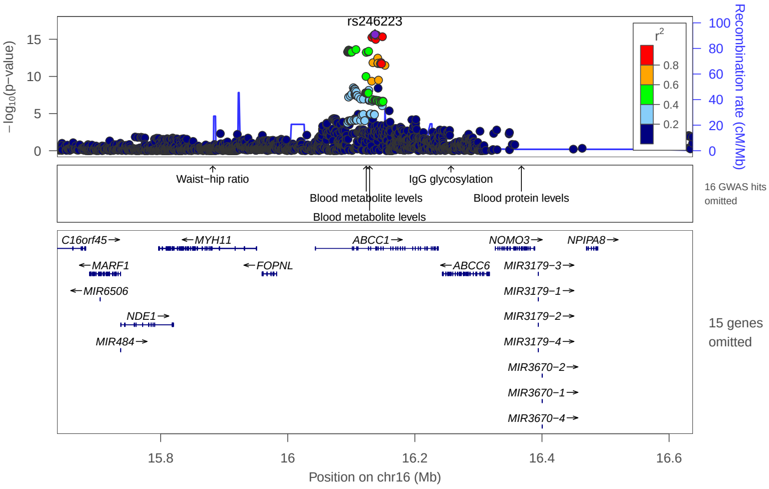

cysteinylglycine disulfide*

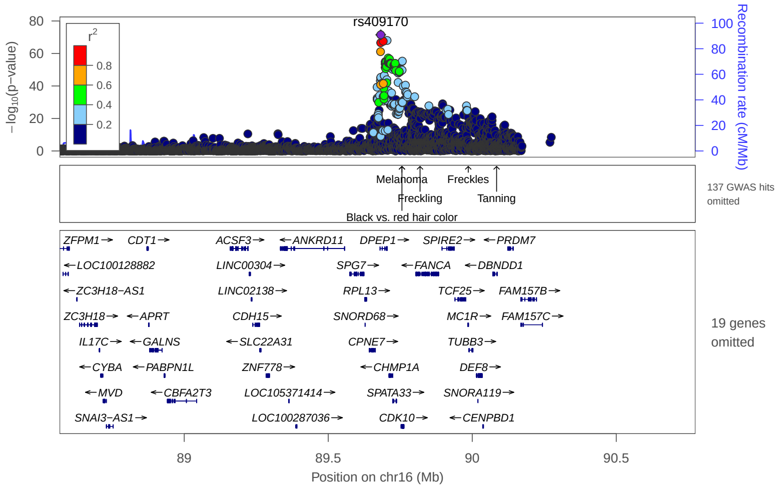

glycine conjugate of C10H14O2 (1)*

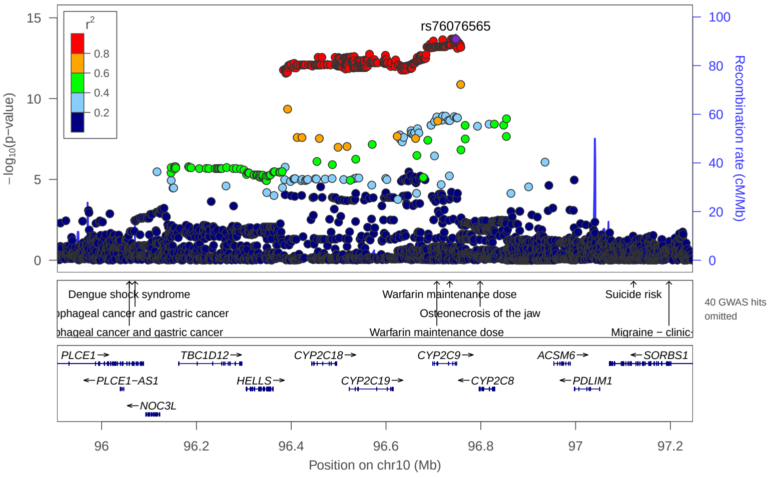

sphingomyelin (d17:1/14:0, d16:1/15:0)*

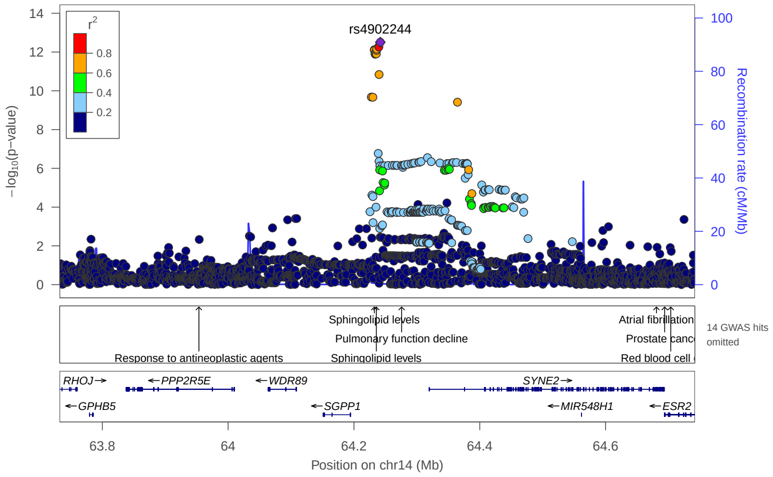

dodecadienoate (12:2)*

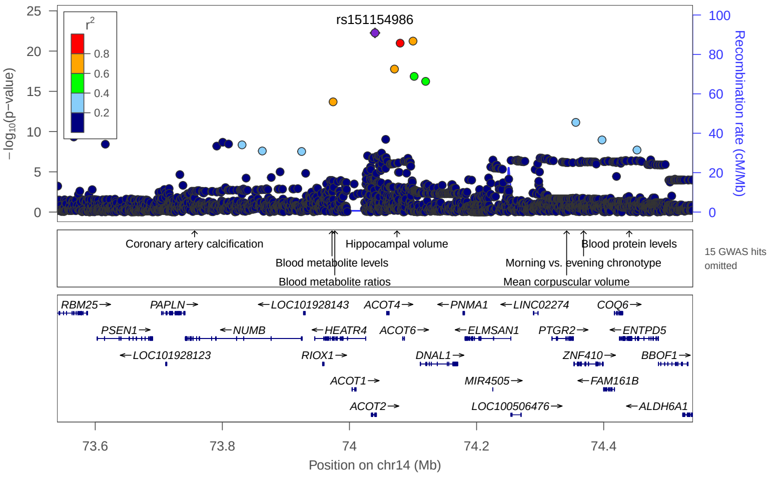

indoleacetoylcarnitine*

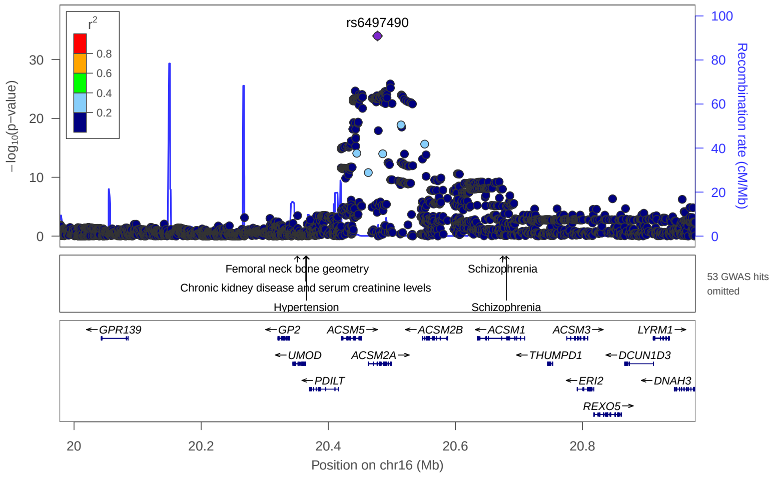

glucuronide of piperine metabolite C17H21NO3 (3)*

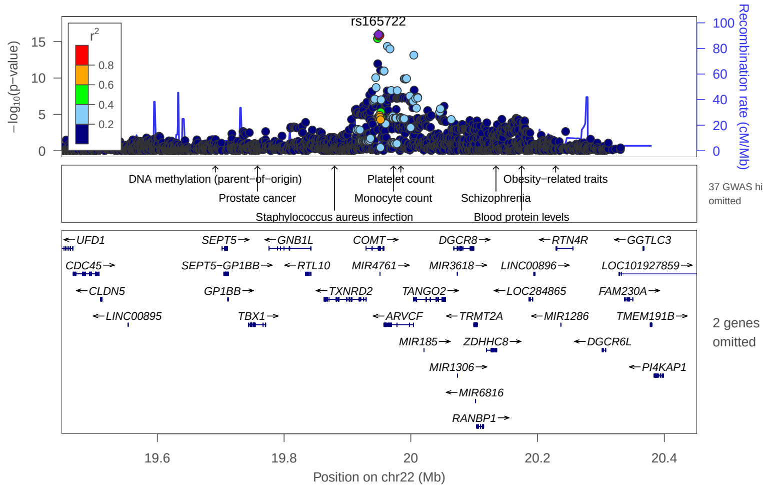

glucuronide of piperine metabolite C17H21NO3 (4)*

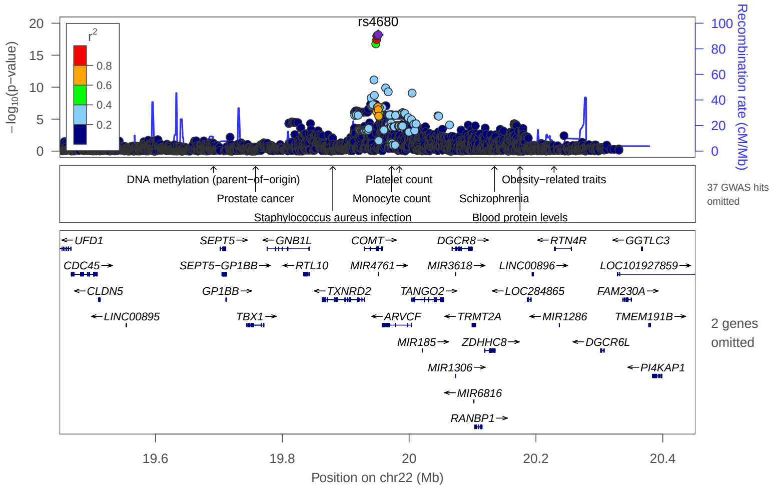

glucuronide of piperine metabolite C17H21NO3 (5)*

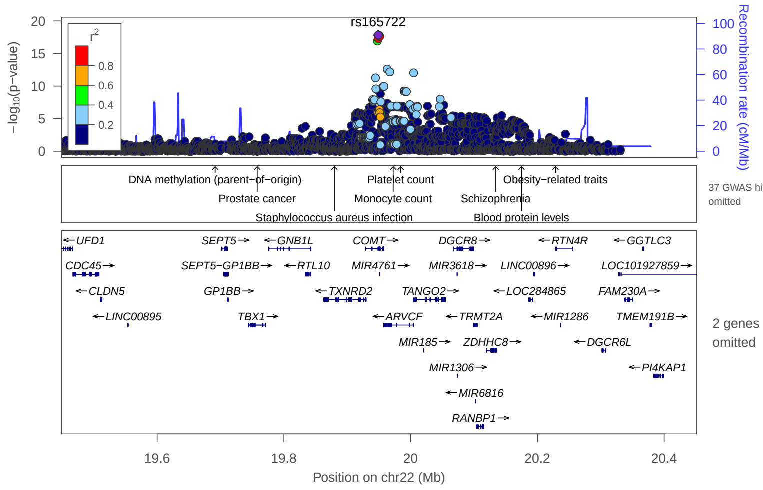

sulfate of piperine metabolite C18H21NO3 (1)*

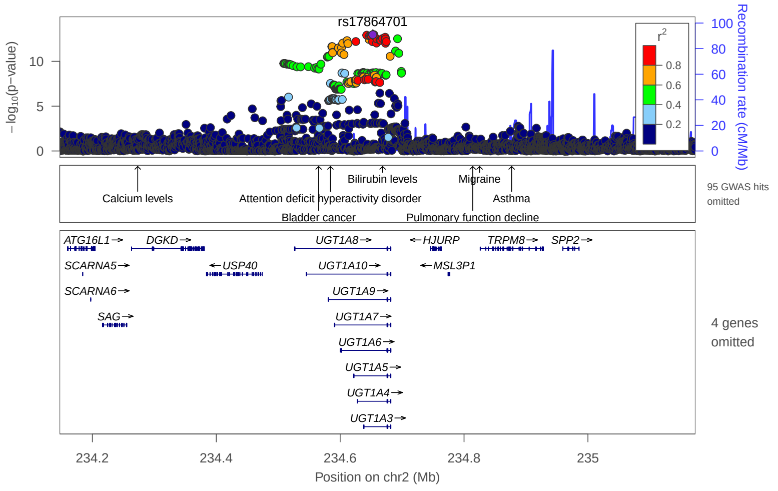

sulfate of piperine metabolite C18H21NO3 (1)*

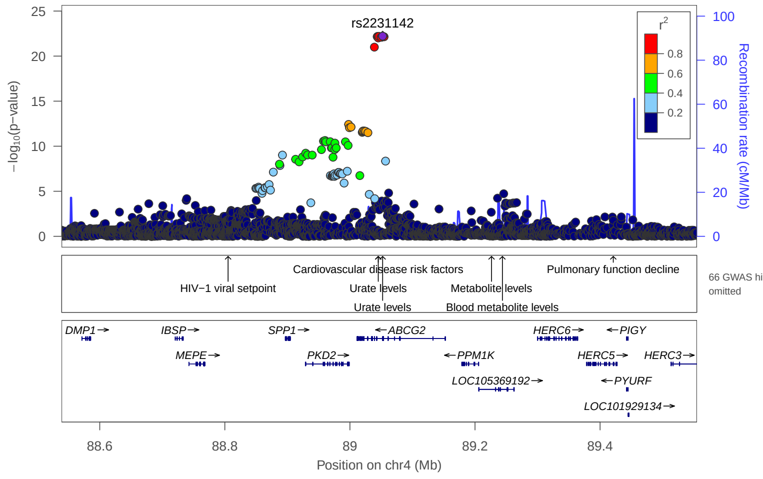

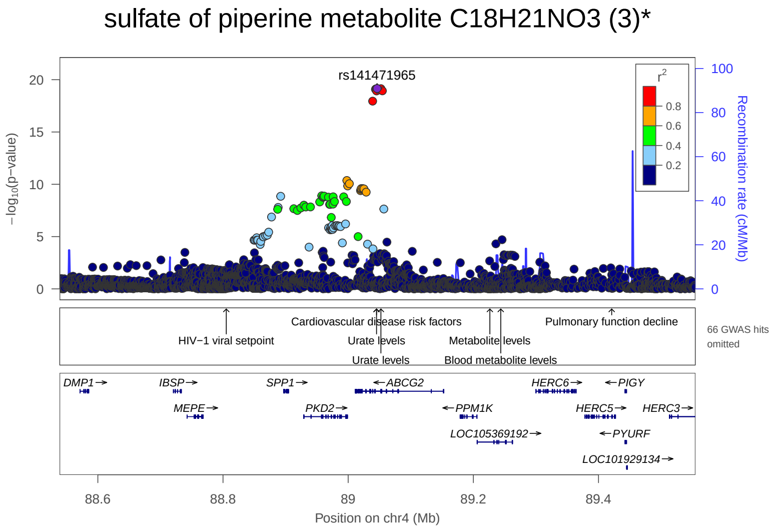
sulfate of piperine metabolite C18H21NO3 (3)*
rs141471965
−log10(p−value)
Recombination rate (cM/Mb)
r2
0.8
0.6
0.4
0.2
HIV−1 viral setpoint
Cardiovascular disease risk factors
Urate levels
Urate levels
Metabolite levels
Blood metabolite levels
Pulmonary function decline
66 GWAS hits omitted
DMP1 IBSP SPP1 ABCG2 HERC6 PIGY
MEPE PKD2 PPM1K HERC5 HERC3
LOC105369192 PYURF
LOC101929134
88.6 88.8 89 89.2 89.4
Position on chr4 (Mb)

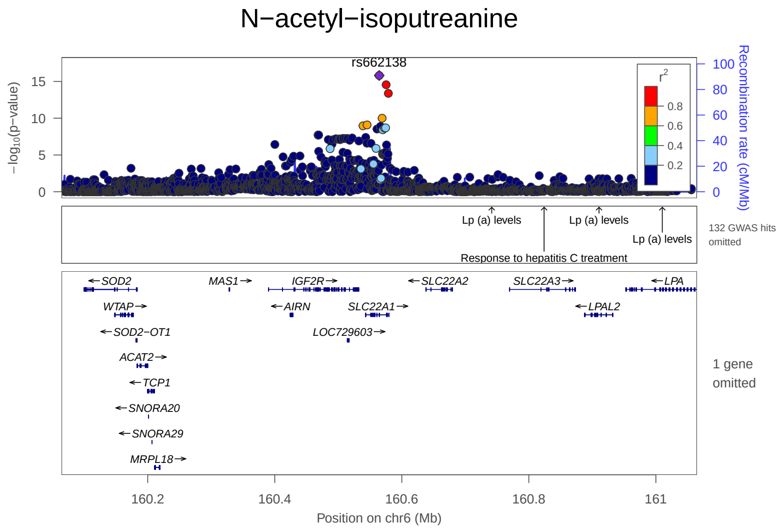
N−acetyl−isoputreanine
rs662138
−log10(p−value)
Recombination rate (cM/Mb)
r2
Lp (a) levels
Lp (a) levels
Lp (a) levels
Response to hepatitis C treatment
132 GWAS hits omitted
SOD2 MAS1 IGF2R SLC22A2 SLC22A3 LPA
WTAP AIRN SLC22A1 LPAL2
SOD2−OT1 LOC729603
ACAT2
TCP1
SNORA20
SNORA29
MRPL18
1 gene omitted
160.2 160.4 160.6 160.8 161
Position on chr6 (Mb)

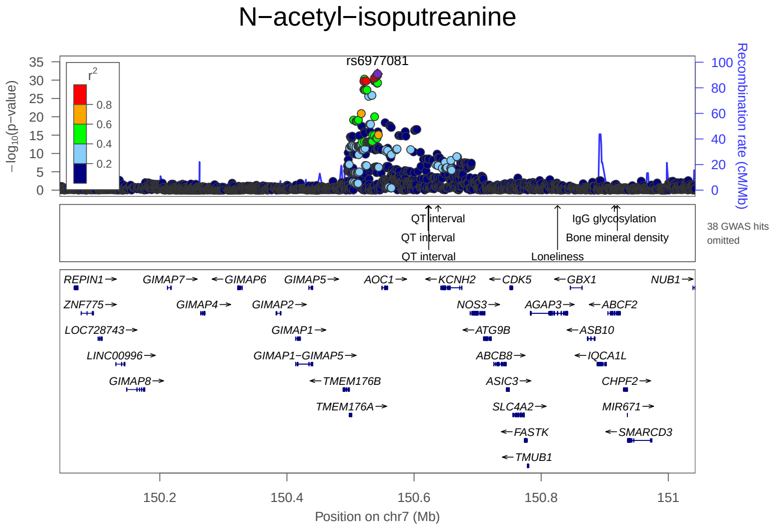
N−acetyl−isoputreanine
rs6977081
−log10(p−value)
Recombination rate (cM/Mb)
r2
QT interval
QT interval
QT interval
Loneliness
IgG glycosylation
Bone mineral density
38 GWAS hits omitted
REPIN1 GIMAP7 GIMAP6 GIMAP5 AOC1 KCNH2 CDK5 GBX1 NUB1
ZNF775 GIMAP4 GIMAP2 NOS3 AGAP3 ABCF2
LOC728743 GIMAP1 ATG9B ASB10
LINC00996 GIMAP1−GIMAP5 ABCB8 IQCA1L
GIMAP8 TMEM176B ASIC3 CHPF2
TMEM176A SLC4A2 MIR671
FASTK SMARCD3
TMUB1
150.2 150.4 150.6 150.8 151
Position on chr7 (Mb)

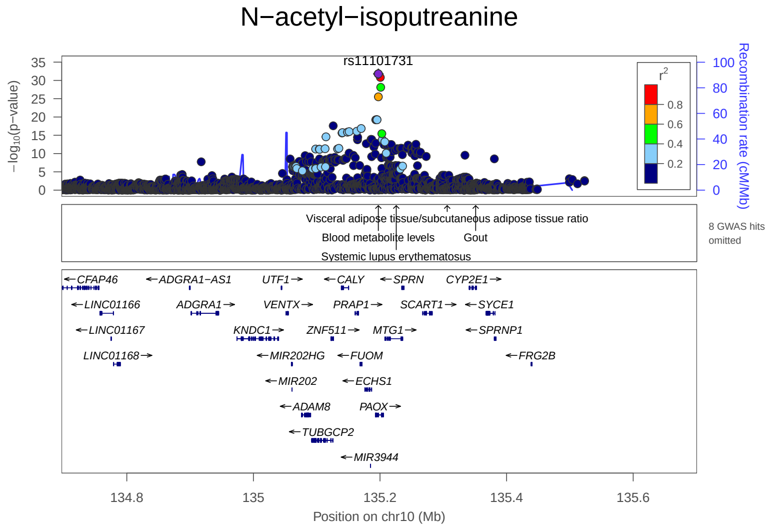
N−acetyl−isoputreanine
rs11101731
−log10(p−value)
Recombination rate (cM/Mb)
r2
Visceral adipose tissue/subcutaneous adipose tissue ratio
Blood metabolite levels
Gout
Systemic lupus erythematosus
8 GWAS hits omitted
CFAP46 ADGRA1−AS1 UTF1 CALY SPRN CYP2E1
LINC01166 ADGRA1 VENTX PRAP1 SCART1 SYCE1
LINC01167 KNDC1 ZNF511 MTG1 SPRNP1
LINC01168 MIR202HG FUOM FRG2B
MIR202 ECHS1
ADAM8 PAOX
TUBGCP2
MIR3944
134.8 135 135.2 135.4 135.6
Position on chr10 (Mb)

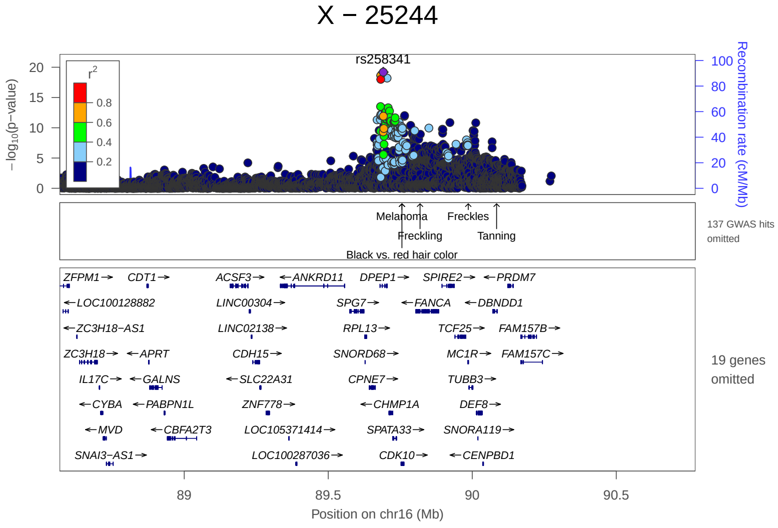
X − 25244
rs258341
−log10(p−value)
Recombination rate (cM/Mb)
r2
Melanoma
Freckles
Freckling
Tanning
Black vs. red hair color
137 GWAS hits omitted
ZFPM1 CDT1 ACSF3 ANKRD11 DPEP1 SPIRE2 PRDM7
LOC100128882 LINC00304 SPG7 FANCA DBNDD1
ZC3H18−AS1 LINC02138 RPL13 TCF25 FAM157B
ZC3H18 APRT CDH15 SNORD68 MC1R FAM157C
IL17C GALNS SLC22A31 CPNE7 TUBB3
CYBA PABPN1L ZNF778 CHMP1A DEF8
MVD CBFA2T3 LOC105371414 SPATA33 SNORA119
SNAI3−AS1 LOC100287036 CDK10 CENPBD1
19 genes omitted
89 89.5 90 90.5
Position on chr16 (Mb)

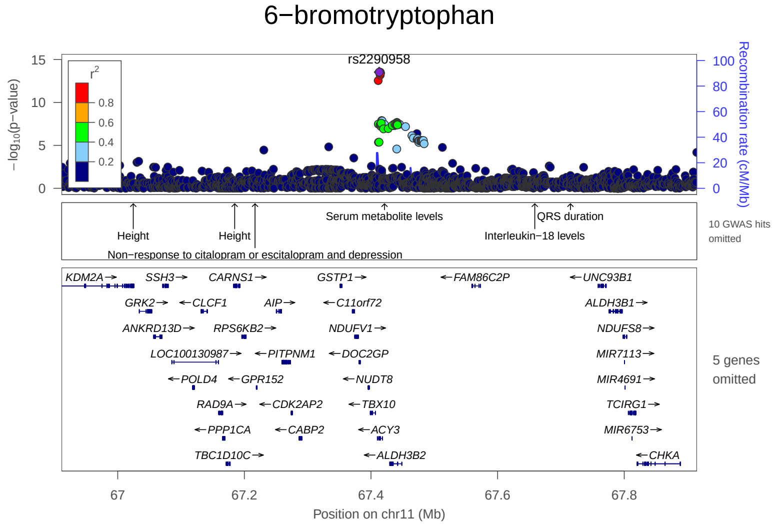
6−bromotryptophan
rs2290958
−log10(p−value)
Recombination rate (cM/Mb)
r2
Height
Height
Non−response to citalopram or escitalopram and depression
Serum metabolite levels
QRS duration
Interleukin−18 levels
10 GWAS hits omitted
KDM2A SSH3 CARNS1 GSTP1 FAM86C2P UNC93B1
GRK2 CLCF1 AIP C11orf72 ALDH3B1
ANKRD13D RPS6KB2 NDUFV1 NDUFS8
LOC100130987 PITPNM1 DOC2GP MIR7113
POLD4 GPR152 NUDT8 MIR4691
RAD9A CDK2AP2 TBX10 TCIRG1
PPP1CA CABP2 ACY3 MIR6753
TBC1D10C ALDH3B2 CHKA
5 genes omitted
67 67.2 67.4 67.6 67.8
Position on chr11 (Mb)

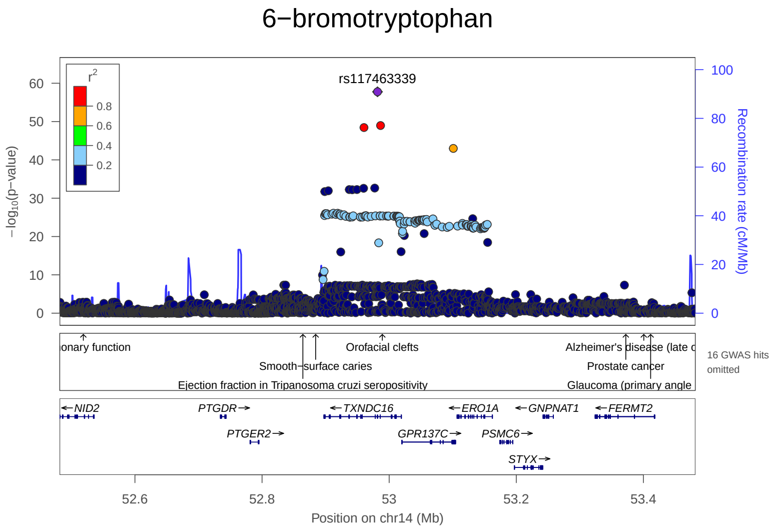
6−bromotryptophan
rs117463339
−log10(p−value)
Recombination rate (cM/Mb)
r2
onary function
Orofacial clefts
Alzheimer's disease (late o
Smooth-surface caries
Prostate cancer
Ejection fraction in Tripanosoma cruzi seropositivity
Glaucoma (primary angle
16 GWAS hits omitted
←NID2
PTGDR→
←TXNDC16
←ERO1A
←GNPNAT1
←FERMT2
PTGER2→
GPR137C→
PSMC6→
STYX→
Position on chr14 (Mb)

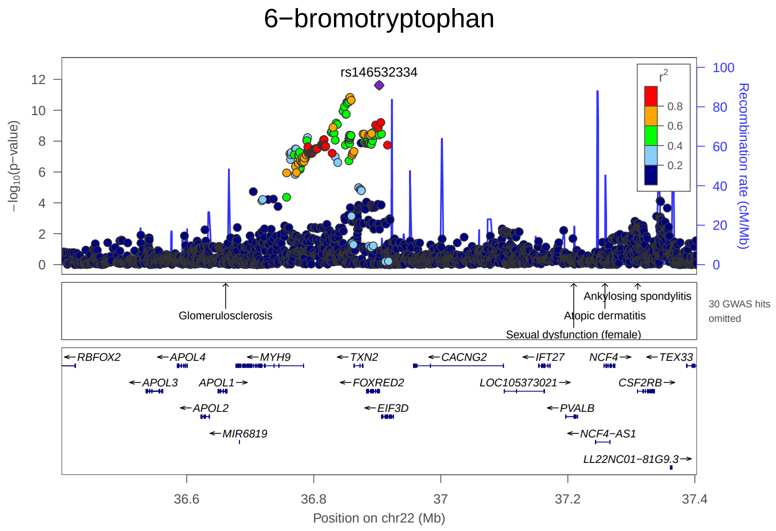
6−bromotryptophan
rs146532334
−log10(p−value)
Recombination rate (cM/Mb)
r2
Glomerulosclerosis
Ankylosing spondylitis
Atopic dermatitis
Sexual dysfunction (female)
30 GWAS hits omitted
←RBFOX2
←APOL4
←MYH9
←TXN2
←CACNG2
←IFT27
NCF4→
←TEX33
←APOL3
APOL1→
←FOXRED2
LOC105373021→
CSF2RB→
←APOL2
←EIF3D
←PVALB
←MIR6819
←NCF4−AS1
LL22NC01−81G9.3→
Position on chr22 (Mb)

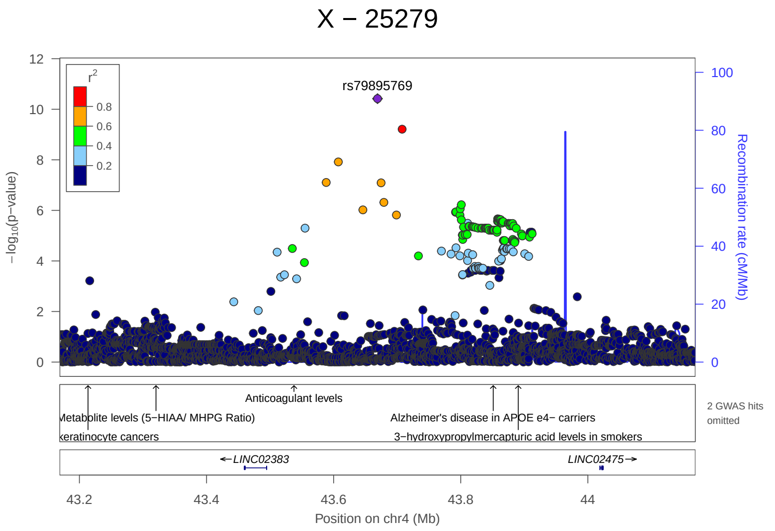
X − 25279
rs79895769
−log10(p−value)
Recombination rate (cM/Mb)
r2
Anticoagulant levels
Metabolite levels (5−HIAA/ MHPG Ratio)
Alzheimer's disease in APOE e4− carriers
keratinocyte cancers
3−hydroxypropylmercapturic acid levels in smokers
2 GWAS hits omitted
←LINC02383
LINC02475→
Position on chr4 (Mb)

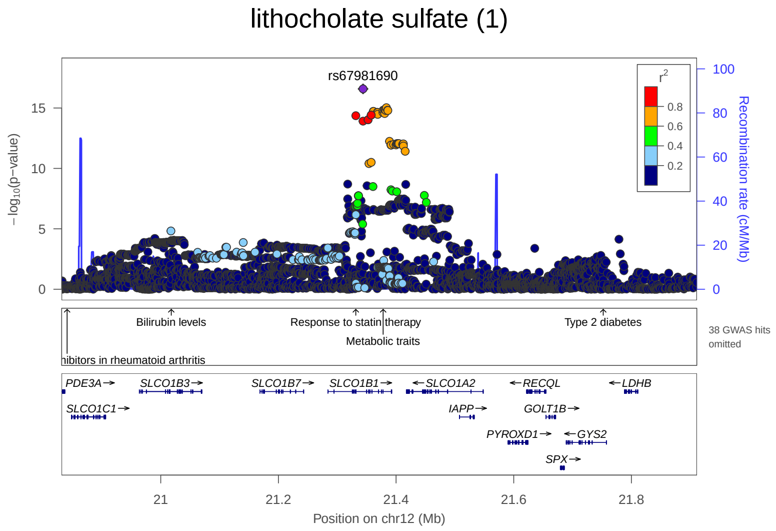
lithocholate sulfate (1)
rs67981690
−log10(p−value)
Recombination rate (cM/Mb)
r2
Bilirubin levels
Response to statin therapy
Type 2 diabetes
Metabolic traits
hibitors in rheumatoid arthritis
38 GWAS hits omitted
PDE3A→
SLCO1B3→
SLCO1B7→
SLCO1B1→
←SLCO1A2
←RECQL
←LDHB
SLCO1C1→
IAPP→
GOLT1B→
PYROXD1→
←GYS2
SPX→
Position on chr12 (Mb)

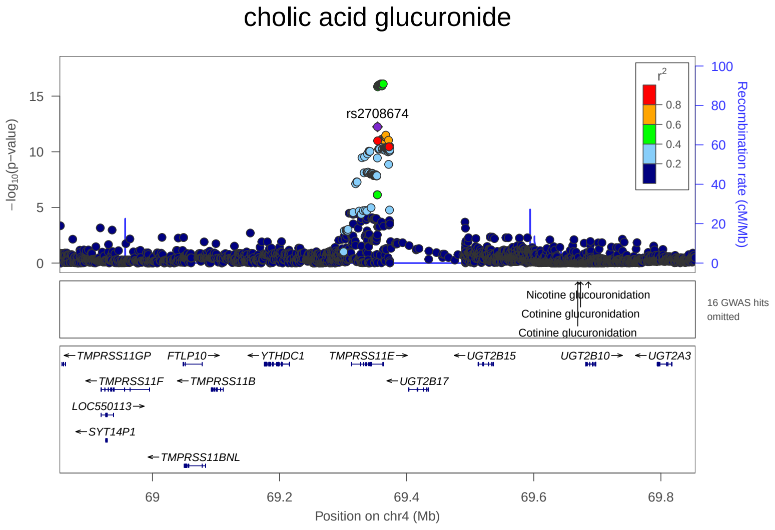
cholic acid glucuronide
rs2708674
−log10(p−value)
Recombination rate (cM/Mb)
r2
Nicotine glucouronidation
Cotinine glucuronidation
Cotinine glucuronidation
16 GWAS hits omitted
←TMPRSS11GP
FTLP10→
←YTHDC1
TMPRSS11E→
←UGT2B15
UGT2B10→
←UGT2A3
←TMPRSS11F
←TMPRSS11B
←UGT2B17
LOC550113→
←SYT14P1
←TMPRSS11BNL
Position on chr4 (Mb)

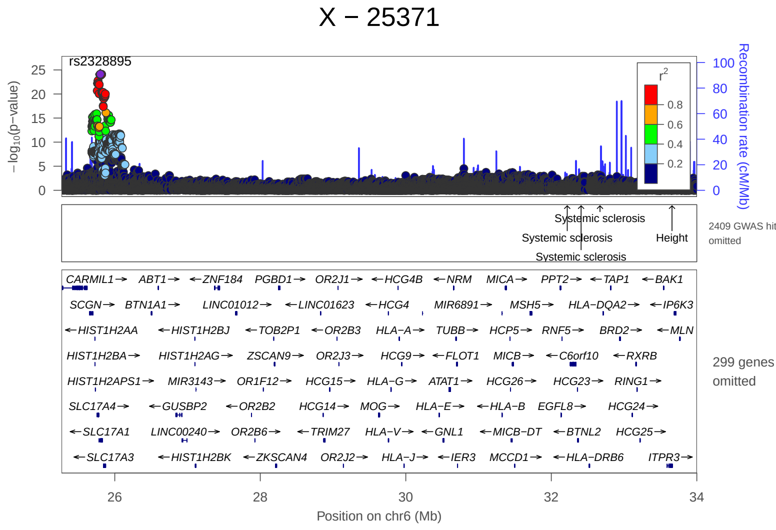
X − 25371
rs2328895
−log10(p−value)
Recombination rate (cM/Mb)
r2
Systemic sclerosis
Systemic sclerosis
Height
Systemic sclerosis
2409 GWAS hit omitted
299 genes omitted
Position on chr6 (Mb)

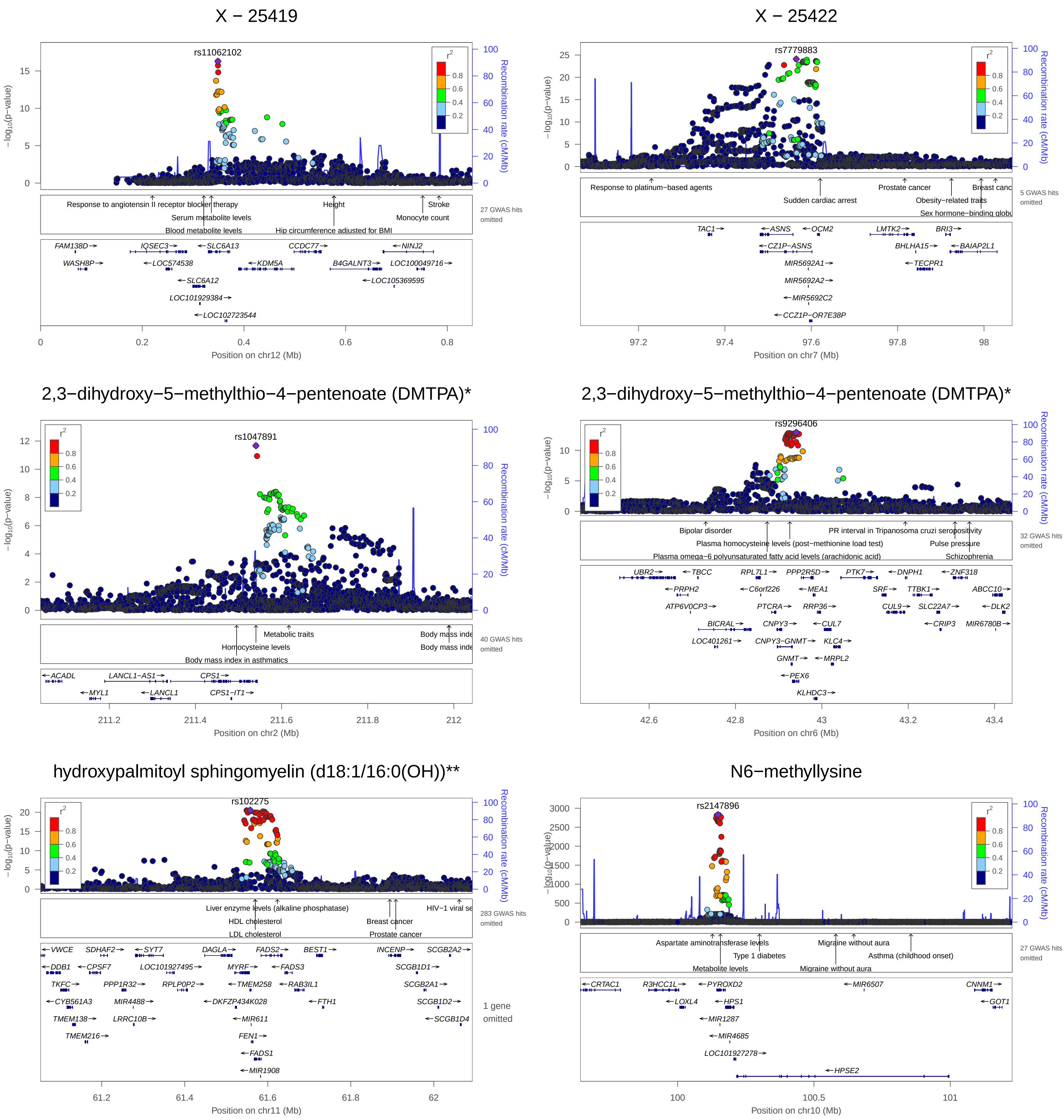
X − 25419
rs11062102
Response to angiotensin II receptor blocker therapy
Serum metabolite levels
Blood metabolite levels
Height
Hip circumference adjusted for BMI
Stroke
Monocyte count
27 GWAS hits omitted
FAM138D
WASH8P
IQSEC3
LOC574538
SLC6A13
SLC6A12
LOC101929384
LOC102723544
KDM5A
CCDC77
B4GALNT3
NINJ2
LOC100049716
LOC105369595
Position on chr12 (Mb)
X − 25422
rs7779883
Response to platinum−based agents
Sudden cardiac arrest
Prostate cancer
Obesity−related traits
Breast canc
Sex hormone−binding globu
5 GWAS hits omitted
TAC1
ASNS
OCM2
CZ1P−ASNS
MIR5692A1
MIR5692A2
MIR5692C2
CCZ1P−OR7E38P
LMTK2
BRI3
BHLHA15
BAIAP2L1
TECPR1
Position on chr7 (Mb)
2,3−dihydroxy−5−methylthio−4−pentenoate (DMTPA)*
rs1047891
Metabolic traits
Homocysteine levels
Body mass index in asthmatics
Body mass inde
40 GWAS hits omitted
ACADL
MYL1
LANCL1−AS1
LANCL1
CPS1
CPS1−IT1
Position on chr2 (Mb)
2,3−dihydroxy−5−methylthio−4−pentenoate (DMTPA)*
rs9296406
Bipolar disorder
Plasma homocysteine levels (post−methionine load test)
Plasma omega−6 polyunsaturated fatty acid levels (arachidonic acid)
PR interval in Tripanosoma cruzi seropositivity
Pulse pressure
Schizophrenia
32 GWAS hits omitted
UBR2
TBCC
RPL7L1
PPP2R5D
PTK7
DNPH1
ZNF318
PRPH2
C6orf226
MEA1
SRF
TTBK1
ABCC10
ATP6V0CP3
PTCRA
RRP36
CUL9
SLC22A7
DLK2
BICRAL
CNPY3
CUL7
CRIP3
MIR6780B
LOC401261
CNPY3−GNMT
KLC4
GNMT
MRPL2
PEX6
KLHDC3
Position on chr6 (Mb)
hydroxypalmitoyl sphingomyelin (d18:1/16:0(OH))**
rs102275
Liver enzyme levels (alkaline phosphatase)
HDL cholesterol
LDL cholesterol
Breast cancer
Prostate cancer
HIV−1 viral se
283 GWAS hits omitted
VWCE
SDHAF2
SYT7
DAGLA
FADS2
BEST1
INCENP
SCGB2A2
DDB1
CPSF7
LOC101927495
MYRF
FADS3
SCGB1D1
TKFC
PPP1R32
RPLP0P2
TMEM258
RAB3IL1
SCGB2A1
CYB561A3
MIR4488
DKFZP434K028
FTH1
SCGB1D2
TMEM138
LRRC10B
MIR611
SCGB1D4
TMEM216
FEN1
FADS1
MIR1908
1 gene omitted
Position on chr11 (Mb)
N6−methyllysine
rs2147896
Aspartate aminotransferase levels
Type 1 diabetes
Metabolite levels
Migraine without aura
Asthma (childhood onset)
27 GWAS hits omitted
CRTAC1
R3HCC1L
PYROXD2
MIR6507
CNNM1
LOXL4
HPS1
GOT1
MIR1287
MIR4685
LOC101927278
HPSE2
Position on chr10 (Mb)
−log10(p−value)
Recombination rate (cM/Mb)
r2

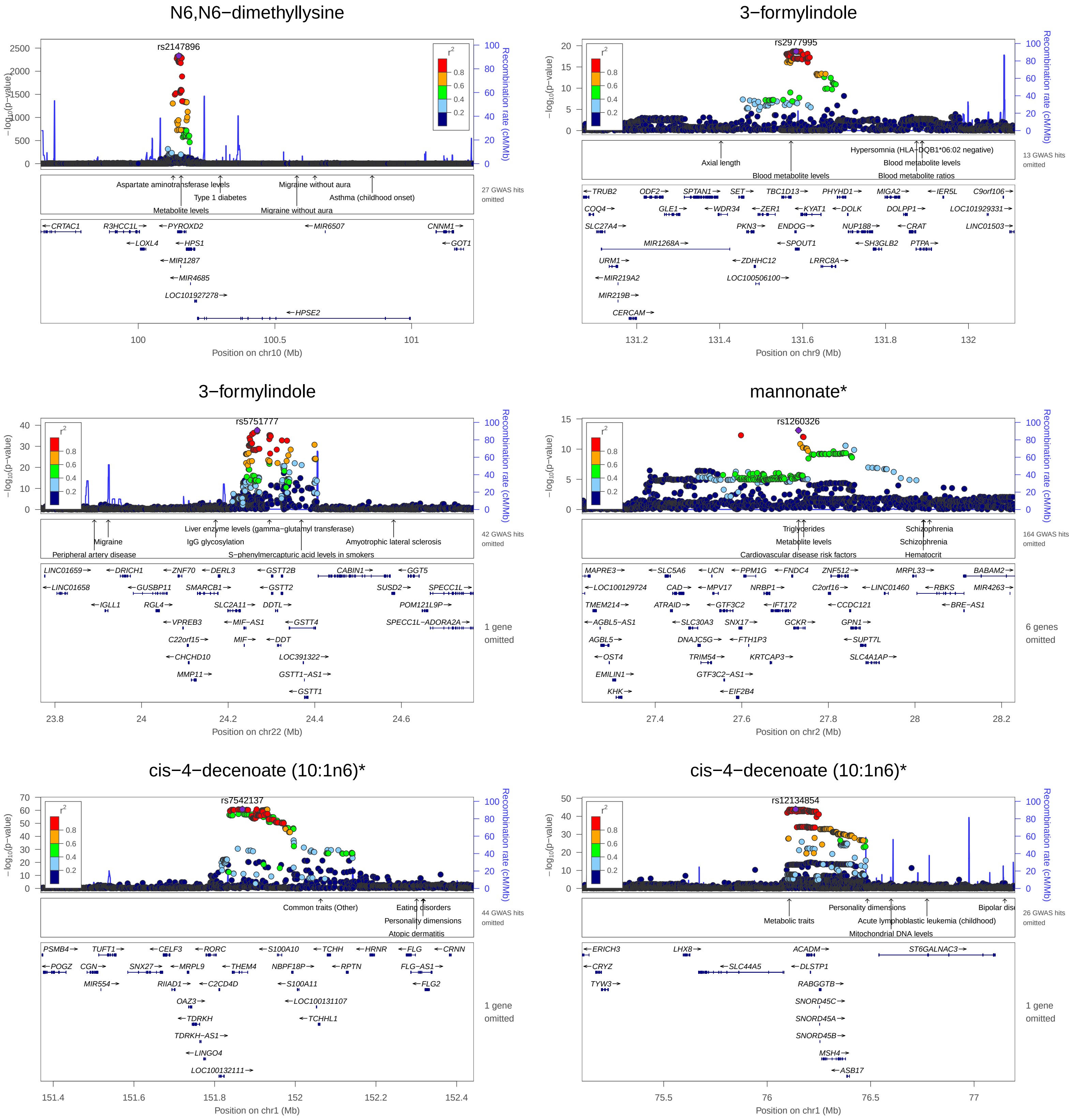

N6,N6−dimethyllysine
rs2147896
Aspartate aminotransferase levels
Type 1 diabetes
Metabolite levels
Migraine without aura
Asthma (childhood onset)
27 GWAS hits omitted
CRTAC1
R3HCC1L
PYROXD2
MIR6507
CNNM1
LOXL4
HPS1
GOT1
MIR1287
MIR4685
LOC101927278
HPSE2
Position on chr10 (Mb)
3−formylindole
rs2977995
Axial length
Blood metabolite levels
Hypersomnia (HLA−DQB1*06:02 negative)
Blood metabolite ratios
13 GWAS hits omitted
TRUB2
ODF2
SPTAN1
SET
TBC1D13
PHYHD1
MIGA2
IER5L
C9orf106
COQ4
GLE1
WDR34
ZER1
KYAT1
DOLK
DOLPP1
LOC101929331
SLC27A4
PKN3
ENDOG
NUP188
CRAT
LINC01503
MIR1268A
SPOUT1
SH3GLB2
PTPA
URM1
ZDHHC12
LRRC8A
MIR219A2
LOC100506100
MIR219B
CERCAM
Position on chr9 (Mb)
3−formylindole
rs5751777
Migraine
Peripheral artery disease
Liver enzyme levels (gamma−glutamyl transferase)
IgG glycosylation
S−phenylmercapturic acid levels in smokers
Amyotrophic lateral sclerosis
42 GWAS hits omitted
LINC01659
DRICH1
ZNF70
DERL3
GSTT2B
CABIN1
GGT5
LINC01658
GUSBP11
SMARCB1
GSTT2
SUSD2
SPECC1L
IGLL1
RGL4
SLC2A11
DDTL
POM121L9P
VPREB3
MIF−AS1
GSTT4
SPECC1L−ADORA2A
C22orf15
MIF
DDT
CHCHD10
LOC391322
MMP11
GSTT1−AS1
GSTT1
1 gene omitted
Position on chr22 (Mb)
mannonate*
rs1260326
Triglycerides
Metabolite levels
Cardiovascular disease risk factors
Schizophrenia
Hematocrit
164 GWAS hits omitted
MAPRE3
SLC5A6
UCN
PPM1G
FNDC4
ZNF512
MRPL33
BABAM2
LOC100129724
CAD
MPV17
NRBP1
C2orf16
LINC01460
RBKS
MIR4263
TMEM214
ATRAID
GTF3C2
IFT172
CCDC121
BRE−AS1
AGBL5−AS1
SLC30A3
SNX17
GCKR
GPN1
AGBL5
DNAJC5G
FTH1P3
SUPT7L
OST4
TRIM54
KRTCAP3
SLC4A1AP
EMILIN1
GTF3C2−AS1
KHK
EIF2B4
6 genes omitted
Position on chr2 (Mb)
cis−4−decenoate (10:1n6)*
rs7542137
Common traits (Other)
Eating disorders
Personality dimensions
Atopic dermatitis
44 GWAS hits omitted
PSMB4
TUFT1
CELF3
RORC
S100A10
TCHH
HRNR
FLG
CRNN
POGZ
CGN
SNX27
MRPL9
THEM4
NBPF18P
RPTN
FLG−AS1
MIR554
RIIAD1
C2CD4D
S100A11
FLG2
OAZ3
LOC100131107
TDRKH
TCHHL1
TDRKH−AS1
LINGO4
LOC100132111
1 gene omitted
Position on chr1 (Mb)
cis−4−decenoate (10:1n6)*
rs12134854
Metabolic traits
Personality dimensions
Acute lymphoblastic leukemia (childhood)
Mitochondrial DNA levels
26 GWAS hits omitted
ERICH3
LHX8
ACADM
ST6GALNAC3
CRYZ
SLC44A5
DLSTP1
TYW3
RABGGTB
SNORD45C
SNORD45A
SNORD45B
MSH4
ASB17
1 gene omitted
Position on chr1 (Mb)
−log10(p−value)
Recombination rate (cM/Mb)

taurodeoxycholic acid 3−sulfate

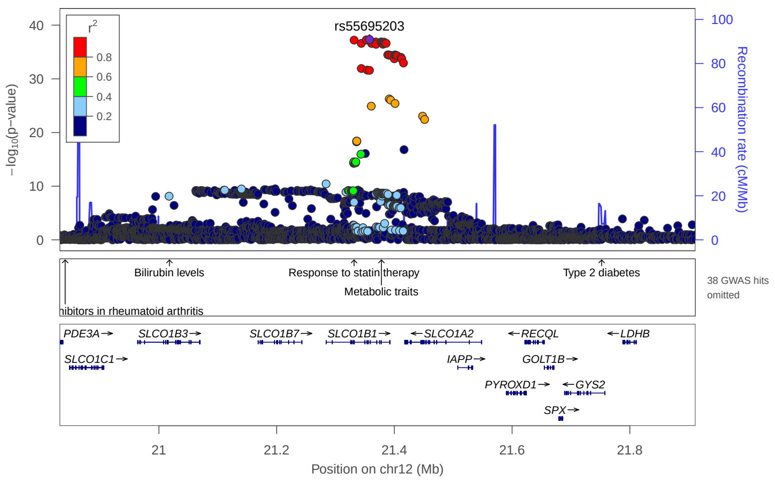

tetradecadienedioate (C14:2−DC)*

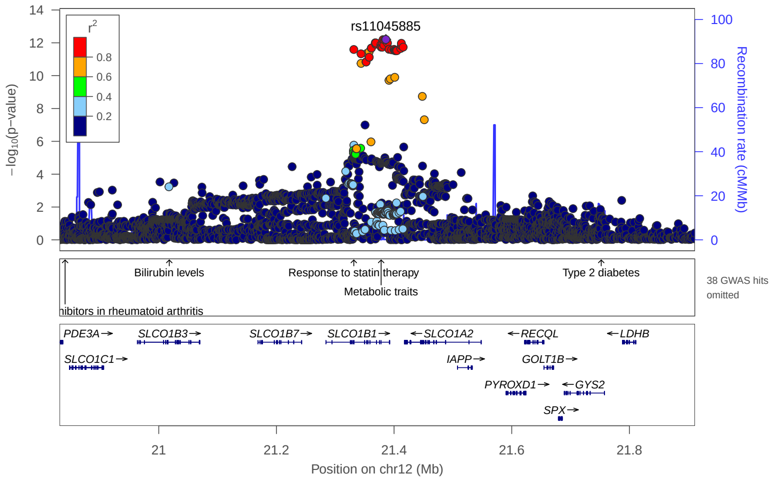

tetradecadienedioate (C14:2−DC)*

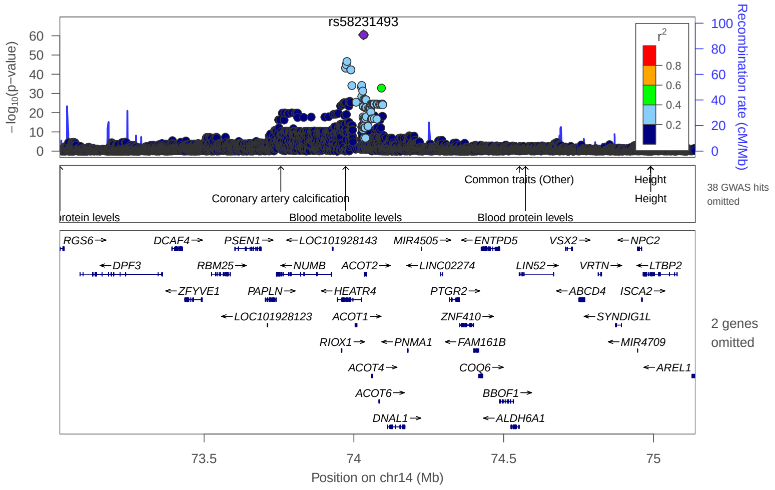

tridecenedioate (C13:1−DC)*

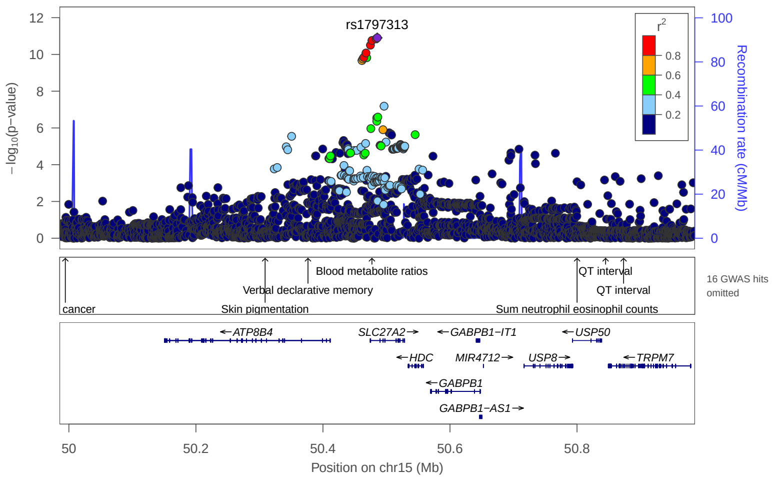

pregnenetriol disulfate*

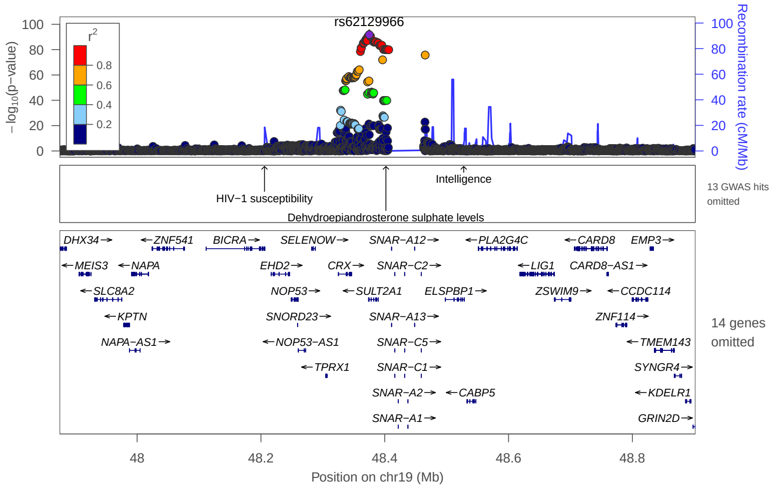

pregnenetriol sulfate*

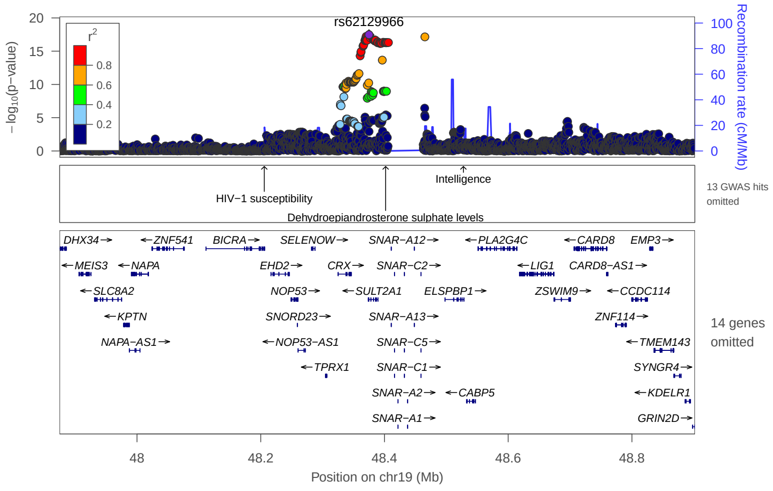

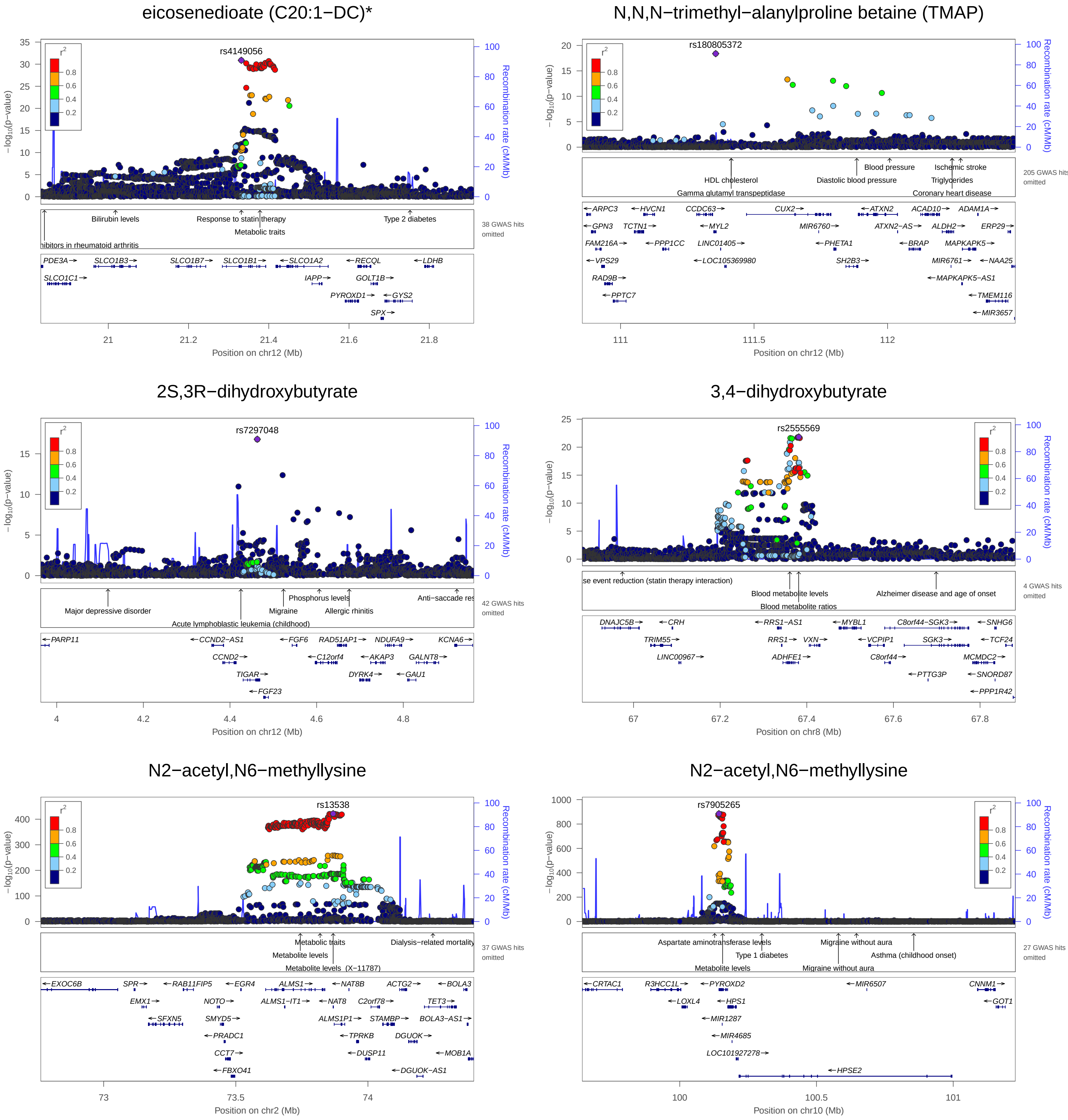

eicosenedioate (C20:1−DC)*
rs4149056
−log10(p−value)
Recombination rate (cM/Mb)
r2
Bilirubin levels
Response to statin therapy
Metabolic traits
Type 2 diabetes
hibitors in rheumatoid arthritis
38 GWAS hits omitted
PDE3A
SLCO1B3
SLCO1B7
SLCO1B1
SLCO1A2
RECQL
LDHB
SLCO1C1
IAPP
GOLT1B
PYROXD1
GYS2
SPX
Position on chr12 (Mb)
N,N,N−trimethyl−alanylproline betaine (TMAP)
rs180805372
HDL cholesterol
Gamma glutamyl transpeptidase
Diastolic blood pressure
Blood pressure
Ischemic stroke
Triglycerides
Coronary heart disease
205 GWAS hits omitted
ARPC3
HVCN1
CCDC63
CUX2
ATXN2
ACAD10
ADAM1A
GPN3
TCTN1
MYL2
MIR6760
ATXN2−AS
ALDH2
ERP29
FAM216A
PPP1CC
LINC01405
PHETA1
BRAP
MAPKAPK5
VPS29
LOC105369980
SH2B3
MIR6761
NAA25
RAD9B
MAPKAPK5−AS1
PPTC7
TMEM116
MIR3657
Position on chr12 (Mb)
2S,3R−dihydroxybutyrate
rs7297048
Major depressive disorder
Acute lymphoblastic leukemia (childhood)
Phosphorus levels
Migraine
Allergic rhinitis
Anti−saccade res
42 GWAS hits omitted
PARP11
CCND2−AS1
FGF6
RAD51AP1
NDUFA9
KCNA6
CCND2
C12orf4
AKAP3
GALNT8
TIGAR
DYRK4
GAU1
FGF23
Position on chr12 (Mb)
3,4−dihydroxybutyrate
rs2555569
se event reduction (statin therapy interaction)
Blood metabolite levels
Blood metabolite ratios
Alzheimer disease and age of onset
4 GWAS hits omitted
DNAJC5B
CRH
RRS1−AS1
MYBL1
C8orf44−SGK3
SNHG6
TRIM55
RRS1
VXN
VCPIP1
SGK3
TCF24
LINC00967
ADHFE1
C8orf44
MCMDC2
PTTG3P
SNORD87
PPP1R42
Position on chr8 (Mb)
N2−acetyl,N6−methyllysine
rs13538
Metabolic traits
Metabolite levels
Metabolite levels (X−11787)
Dialysis−related mortality
37 GWAS hits omitted
EXOC6B
SPR
RAB11FIP5
EGR4
ALMS1
NAT8B
ACTG2
BOLA3
EMX1
NOTO
ALMS1−IT1
NAT8
C2orf78
TET3
SFXN5
SMYD5
ALMS1P1
STAMBP
BOLA3−AS1
PRADC1
TPRKB
DGUOK
CCT7
DUSP11
MOB1A
FBXO41
DGUOK−AS1
Position on chr2 (Mb)
N2−acetyl,N6−methyllysine
rs7905265
Aspartate aminotransferase levels
Metabolite levels
Type 1 diabetes
Migraine without aura
Asthma (childhood onset)
27 GWAS hits omitted
CRTAC1
R3HCC1L
PYROXD2
MIR6507
CNNM1
LOXL4
HPS1
GOT1
MIR1287
MIR4685
LOC101927278
HPSE2
Position on chr10 (Mb)

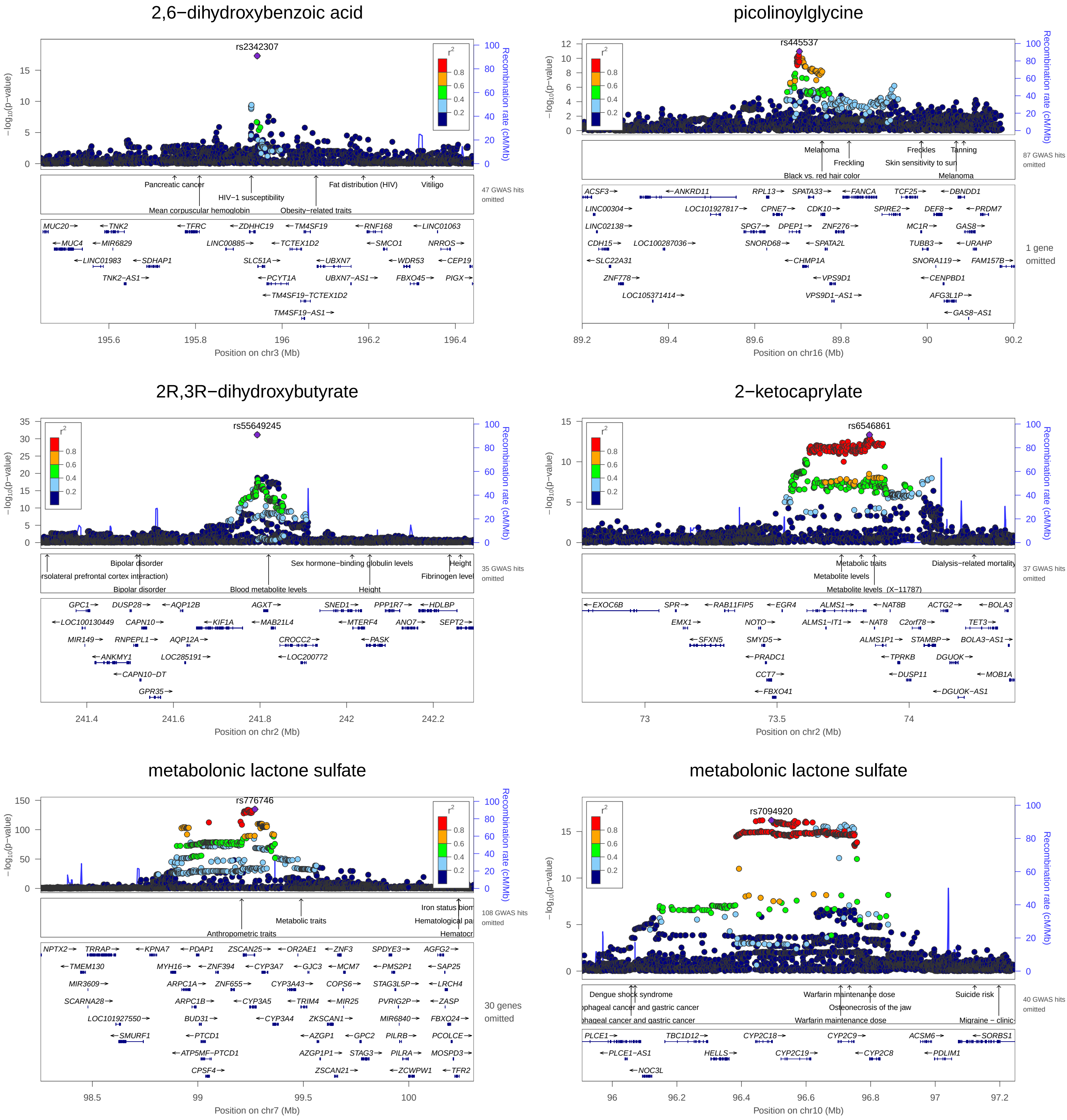

2,6−dihydroxybenzoic acid
rs2342307
−log10(p−value)
Recombination rate (cM/Mb)
r2
0.8
0.6
0.4
0.2
Pancreatic cancer
HIV−1 susceptibility
Mean corpuscular hemoglobin
Fat distribution (HIV)
Obesity−related traits
Vitiligo
47 GWAS hits omitted
MUC20 MUC4 TNK2 MIR6829 LINC01983 TNK2−AS1 SDHAP1 TFRC LINC00885 ZDHHC19 SLC51A PCYT1A TM4SF19−TCTEX1D2 TM4SF19−AS1 TM4SF19 TCTEX1D2 UBXN7 UBXN7−AS1 RNF168 SMCO1 WDR53 FBXO45 LINC01063 NRROS CEP19 PIGX
195.6 195.8 196 196.2 196.4
Position on chr3 (Mb)
picolinoylglycine
rs445537
Melanoma
Freckling
Black vs. red hair color
Freckles
Skin sensitivity to sun
Tanning
Melanoma
87 GWAS hits omitted
ACSF3 LINC00304 LINC02138 CDH15 SLC22A31 ZNF778 LOC105371414 ANKRD11 LOC101927817 LOC100287036 RPL13 CPNE7 SPG7 SNORD68 DPEP1 SPATA33 CDK10 CHMP1A FANCA ZNF276 SPATA2L VPS9D1 VPS9D1−AS1 TCF25 SPIRE2 MC1R TUBB3 SNORA119 CENPBD1 AFG3L1P DEF8 DBNDD1 PRDM7 GAS8 URAHP FAM157B GAS8−AS1
1 gene omitted
89.2 89.4 89.6 89.8 90 90.2
Position on chr16 (Mb)
2R,3R−dihydroxybutyrate
rs55649245
Bipolar disorder
rsolateral prefrontal cortex interaction)
Bipolar disorder
Blood metabolite levels
Sex hormone−binding globulin levels
Height
Height
Fibrinogen level
35 GWAS hits omitted
GPC1 LOC100130449 MIR149 ANKMY1 DUSP28 CAPN10 RNPEPL1 CAPN10−DT GPR35 AQP12B AQP12A LOC285191 KIF1A AGXT MAB21L4 CROCC2 LOC200772 SNED1 MTERF4 PPP1R7 ANO7 PASK HDLBP SEPT2
241.4 241.6 241.8 242 242.2
Position on chr2 (Mb)
2−ketocaprylate
rs6546861
Metabolic traits
Metabolite levels
Metabolite levels (X−11787)
Dialysis−related mortality
37 GWAS hits omitted
EXOC6B SPR EMX1 SFXN5 RAB11FIP5 NOTO SMYD5 PRADC1 CCT7 FBXO41 EGR4 ALMS1 ALMS1−IT1 NAT8B NAT8 ALMS1P1 TPRKB DUSP11 C2orf78 STAMBP ACTG2 DGUOK DGUOK−AS1 TET3 BOLA3 BOLA3−AS1 MOB1A
73 73.5 74
Position on chr2 (Mb)
metabolonic lactone sulfate
rs776746
Anthropometric traits
Metabolic traits
Iron status biom
Hematological pa
Hematocr
108 GWAS hits omitted
NPTX2 TMEM130 MIR3609 SCARNA28 LOC101927550 SMURF1 TRRAP KPNA7 MYH16 ARPC1A ARPC1B BUD31 PTCD1 ATP5MF−PTCD1 CPSF4 PDAP1 ZNF394 ZNF655 CYP3A5 CYP3A7 CYP3A43 CYP3A4 ZSCAN25 OR2AE1 GJC3 TRIM4 ZKSCAN1 AZGP1 AZGP1P1 ZSCAN21 ZNF3 MCM7 COPS6 MIR25 GPC2 STAG3 SPDYE3 PMS2P1 STAG3L5P PVRIG2P MIR6840 PILRB PILRA ZCWPW1 AGFG2 SAP25 LRCH4 ZASP FBXO24 PCOLCE MOSPD3 TFR2
30 genes omitted
98.5 99 99.5 100
Position on chr7 (Mb)
metabolonic lactone sulfate
rs7094920
Dengue shock syndrome
phageal cancer and gastric cancer
hageal cancer and gastric cancer
Warfarin maintenance dose
Osteonecrosis of the jaw
Warfarin maintenance dose
Suicide risk
Migraine − clinic
40 GWAS hits omitted
PLCE1 PLCE1−AS1 NOC3L TBC1D12 HELLS CYP2C18 CYP2C19 CYP2C9 CYP2C8 ACSM6 PDLIM1 SORBS1
96 96.2 96.4 96.6 96.8 97 97.2
Position on chr10 (Mb)

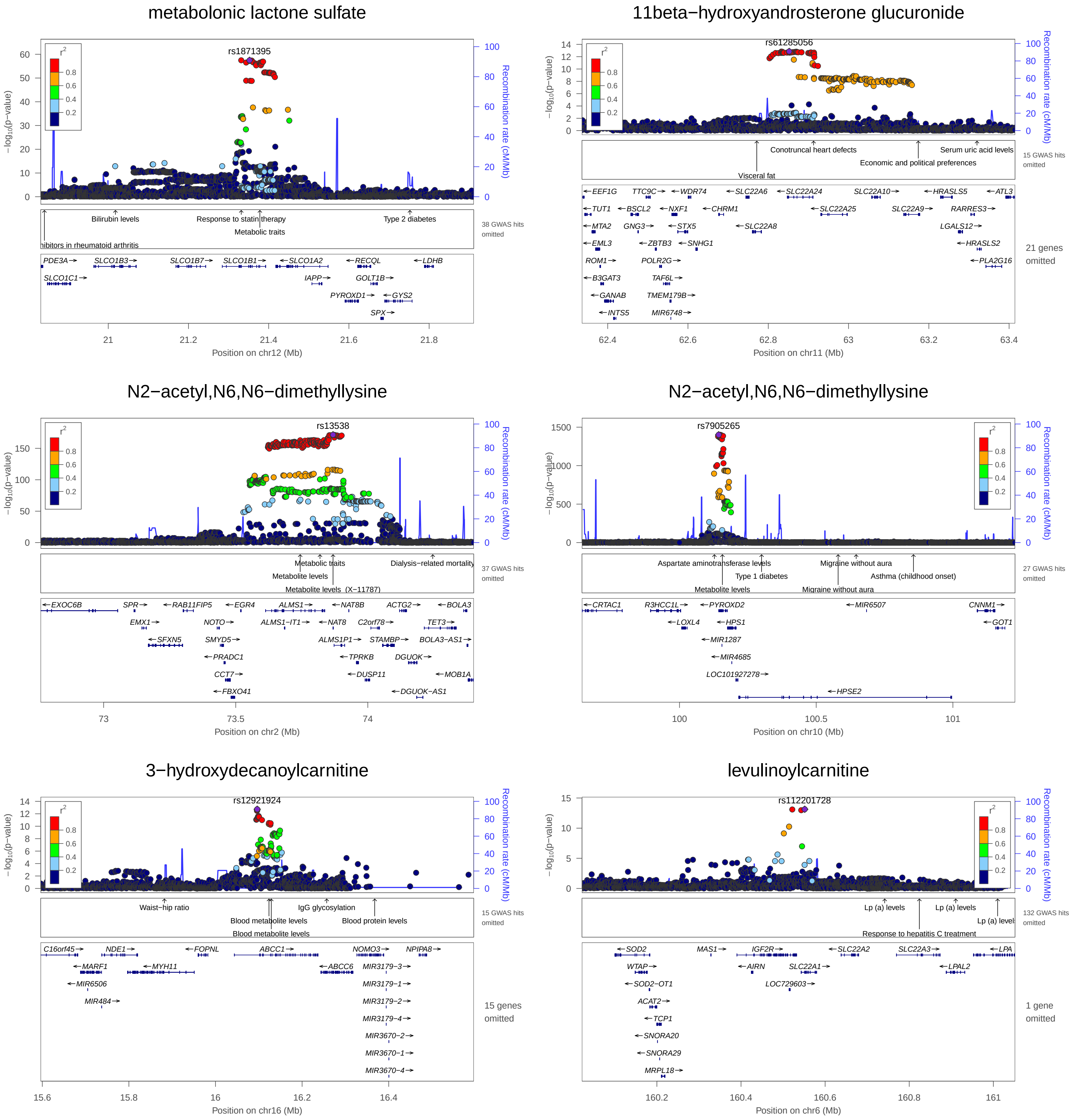
metabolonic lactone sulfate
rs1871395
Position on chr12 (Mb)
38 GWAS hits omitted
Bilirubin levels
Response to statin therapy
Metabolic traits
Type 2 diabetes
hibitors in rheumatoid arthritis
PDE3A SLCO1B3 SLCO1B7 SLCO1B1 SLCO1A2 RECQL LDHB
SLCO1C1 IAPP GOLT1B
PYROXD1 GYS2
SPX
11beta−hydroxyandrosterone glucuronide
rs61285056
Position on chr11 (Mb)
15 GWAS hits omitted
Conotruncal heart defects
Serum uric acid levels
Economic and political preferences
Visceral fat
21 genes omitted
EEF1G TTC9C WDR74 SLC22A6 SLC22A24 SLC22A10 HRASLS5 ATL3
TUT1 BSCL2 NXF1 CHRM1 SLC22A25 SLC22A9 RARRES3
MTA2 GNG3 STX5 SLC22A8 LGALS12
EML3 ZBTB3 SNHG1 HRASLS2
ROM1 POLR2G PLA2G16
B3GAT3 TAF6L
GANAB TMEM179B
INTS5 MIR6748
N2−acetyl,N6,N6−dimethyllysine
rs13538
Position on chr2 (Mb)
37 GWAS hits omitted
Metabolic traits
Dialysis−related mortality
Metabolite levels
Metabolite levels (X−11787)
EXOC6B SPR RAB11FIP5 EGR4 ALMS1 NAT8 ACTG2 BOLA3
EMX1 NOTO ALMS1−IT1 NAT8 C2orf78 TET3
SFXN5 SMYD5 ALMS1P1 STAMBP BOLA3−AS1
PRADC1 TPRKB DGUOK
CCT7 DUSP11 MOB1A
FBXO41 DGUOK−AS1
N2−acetyl,N6,N6−dimethyllysine
rs7905265
Position on chr10 (Mb)
27 GWAS hits omitted
Aspartate aminotransferase levels
Type 1 diabetes
Metabolite levels
Migraine without aura
Asthma (childhood onset)
Migraine without aura
CRTAC1 R3HCC1L PYROXD2 MIR6507 CNNM1
LOXL4 HPS1 GOT1
MIR1287
MIR4685
LOC101927278
HPSE2
3−hydroxydecanoylcarnitine
rs12921924
Position on chr16 (Mb)
15 GWAS hits omitted
Waist−hip ratio
IgG glycosylation
Blood metabolite levels
Blood protein levels
Blood metabolite levels
15 genes omitted
C16orf45 NDE1 FOPNL ABCC1 NOMO3 NPIPA8
MARF1 MYH11 ABCC6 MIR3179−3
MIR6506 MIR3179−1
MIR484 MIR3179−2
MIR3179−4
MIR3670−2
MIR3670−1
MIR3670−4
levulinoylcarnitine
rs112201728
Position on chr6 (Mb)
132 GWAS hits omitted
Lp (a) levels
Lp (a) levels
Lp (a) levels
Response to hepatitis C treatment
1 gene omitted
SOD2 MAS1 IGF2R SLC22A2 SLC22A3 LPA
WTAP AIRN SLC22A1 LPAL2
SOD2−OT1 LOC729603
ACAT2
TCP1
SNORA20
SNORA29
MRPL18
−log10(p−value)
Recombination rate (cM/Mb)
r2

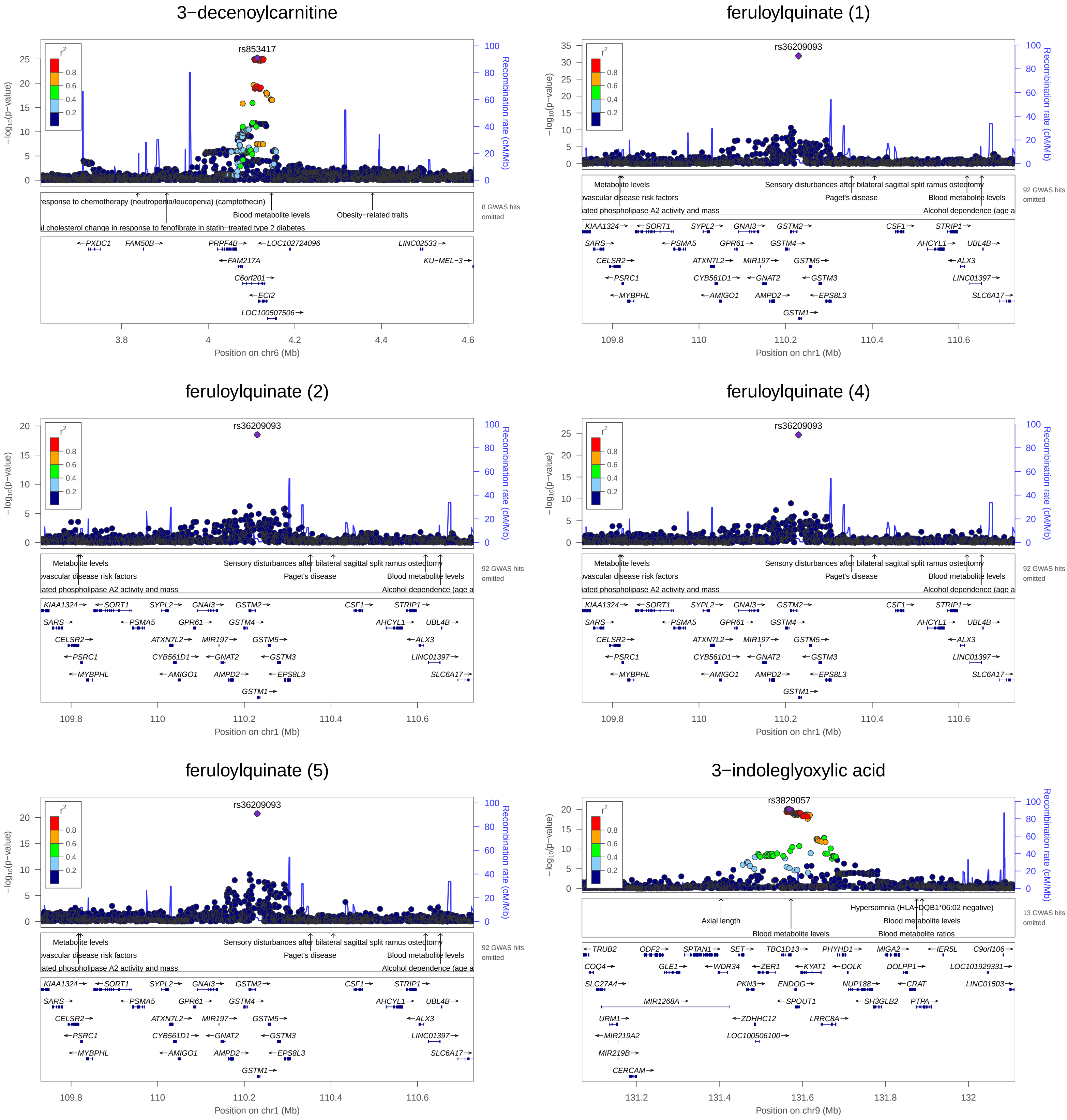

3−decenoylcarnitine
rs853417
Recombination rate (cM/Mb)
Position on chr6 (Mb)
8 GWAS hits omitted
feruloylquinate (1)
rs36209093
Position on chr1 (Mb)
92 GWAS hits omitted
feruloylquinate (2)
rs36209093
Position on chr1 (Mb)
92 GWAS hits omitted
feruloylquinate (4)
rs36209093
Position on chr1 (Mb)
92 GWAS hits omitted
feruloylquinate (5)
rs36209093
Position on chr1 (Mb)
92 GWAS hits omitted
3−indoleglyoxylic acid
rs3829057
Position on chr9 (Mb)
13 GWAS hits omitted

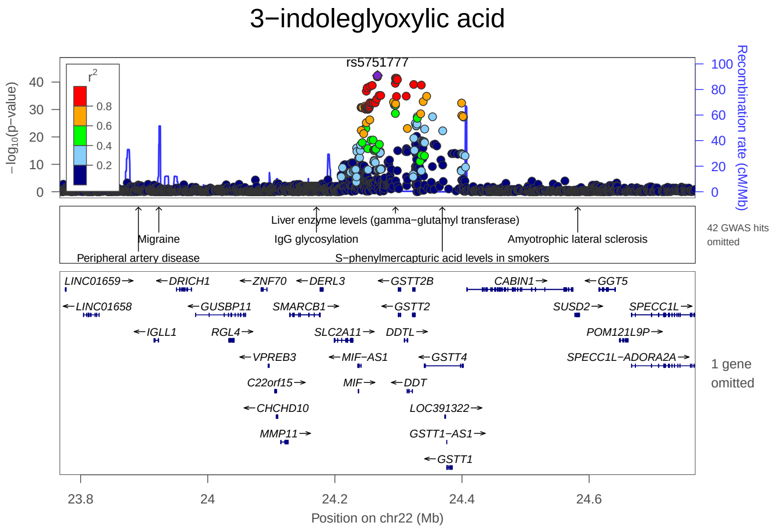
3−indoleglyoxylic acid
rs5751777
Liver enzyme levels (gamma−glutamyl transferase)
Migraine
IgG glycosylation
Amyotrophic lateral sclerosis
Peripheral artery disease
S−phenylmercapturic acid levels in smokers
42 GWAS hits omitted
1 gene omitted
Position on chr22 (Mb)
Recombination rate (cM/Mb)

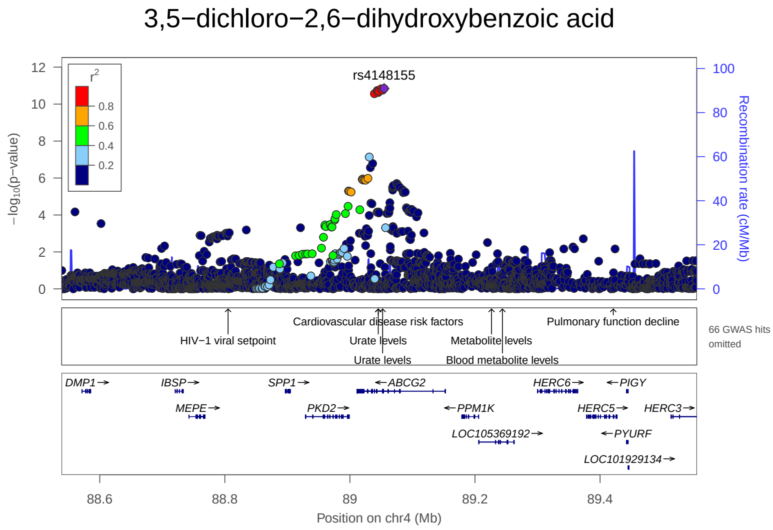
3,5−dichloro−2,6−dihydroxybenzoic acid
rs4148155
Cardiovascular disease risk factors
Pulmonary function decline
HIV−1 viral setpoint
Urate levels
Metabolite levels
Blood metabolite levels
66 GWAS hits omitted
Position on chr4 (Mb)
Recombination rate (cM/Mb)

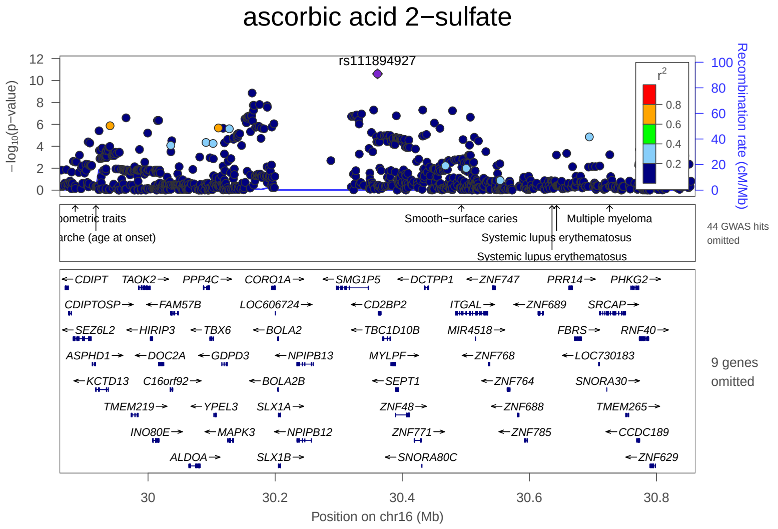
ascorbic acid 2−sulfate
rs111894927
Smooth−surface caries
Multiple myeloma
Systemic lupus erythematosus
44 GWAS hits omitted
9 genes omitted
Position on chr16 (Mb)
Recombination rate (cM/Mb)

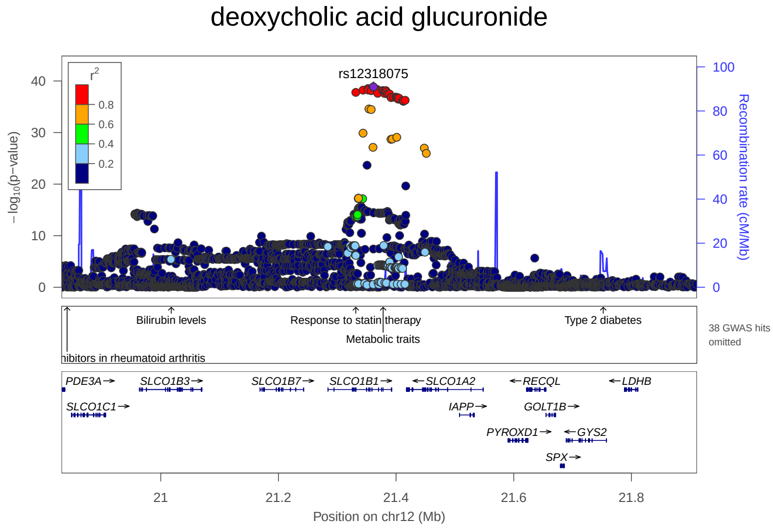
deoxycholic acid glucuronide
rs12318075
Bilirubin levels
Response to statin therapy
Type 2 diabetes
Metabolic traits
38 GWAS hits omitted
Position on chr12 (Mb)
Recombination rate (cM/Mb)

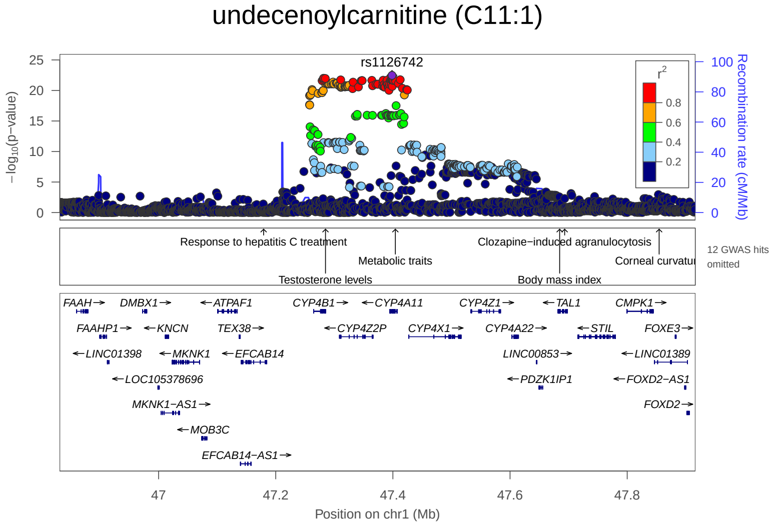
undecenoylcarnitine (C11:1)
rs1126742
Response to hepatitis C treatment
Clozapine−induced agranulocytosis
Metabolic traits
Testosterone levels
Body mass index
12 GWAS hits omitted
Position on chr1 (Mb)
Recombination rate (cM/Mb)

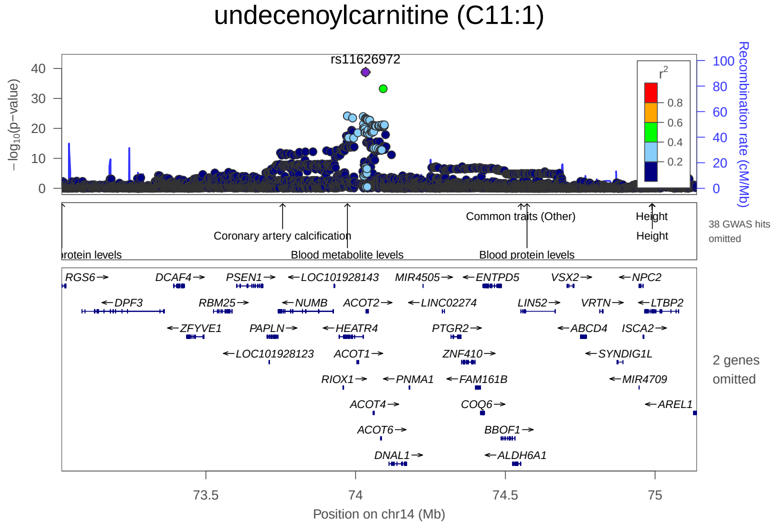
undecenoylcarnitine (C11:1)
rs11626972
Common traits (Other)
Height
Coronary artery calcification
Blood metabolite levels
Blood protein levels
38 GWAS hits omitted
2 genes omitted
Position on chr14 (Mb)
Recombination rate (cM/Mb)

branched−chain, straight−chain, or cyclopropyl 10:1 fatty acid (2)*

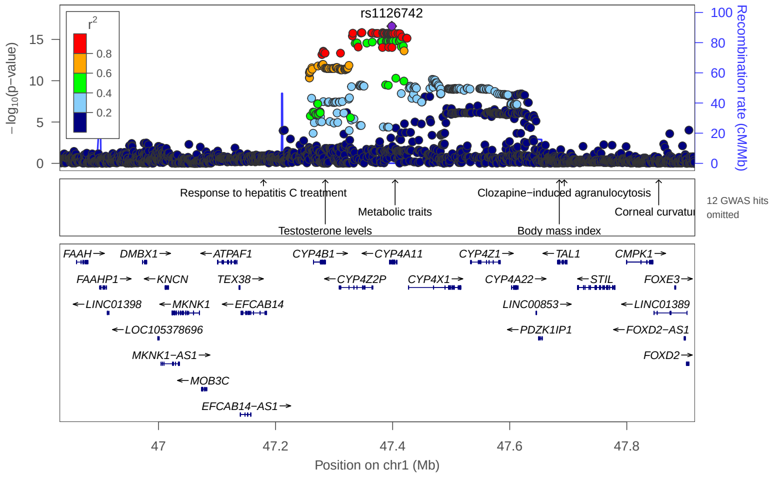

succinoyltaurine

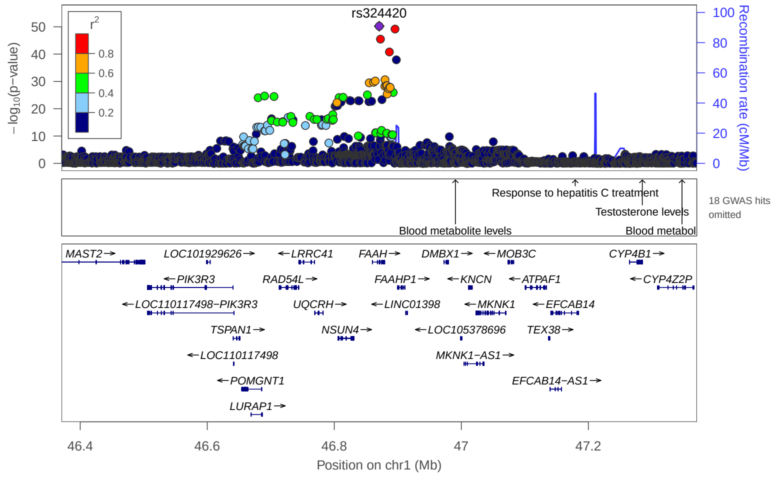

succinoyltaurine

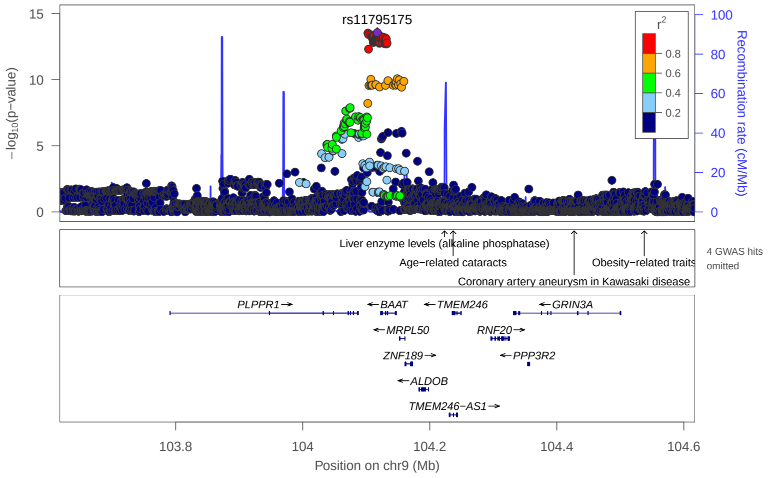

succinoyltaurine

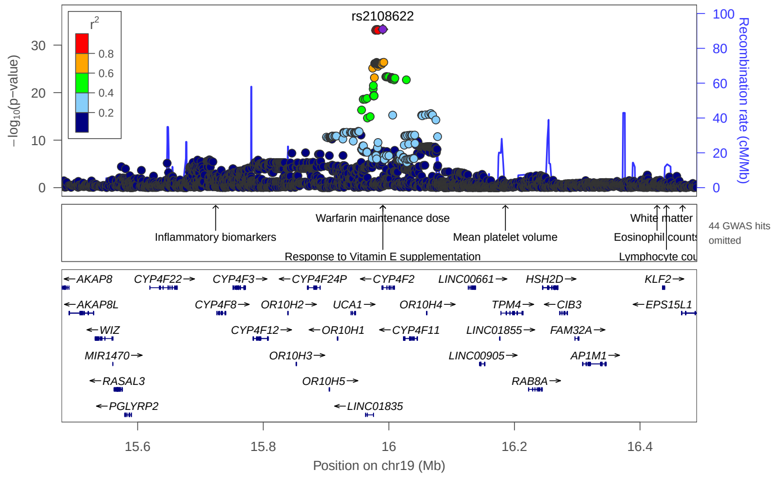

3−bromo−5−chloro−2,6−dihydroxybenzoic acid*

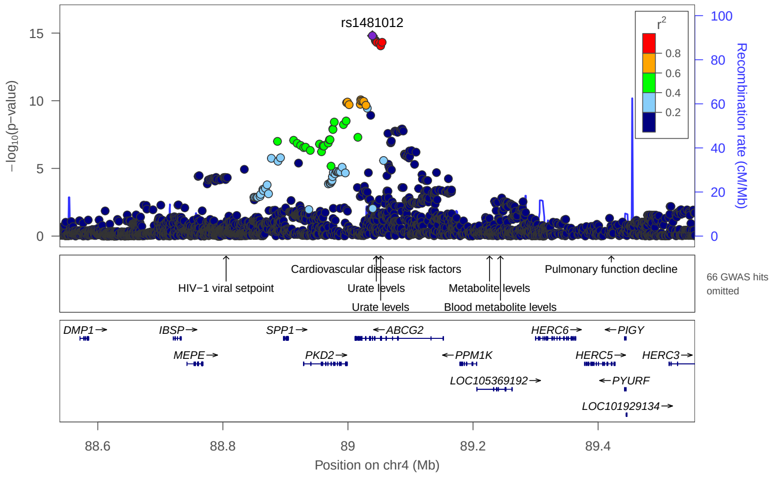

branched−chain, straight−chain, or cyclopropyl 12:1 fatty acid*

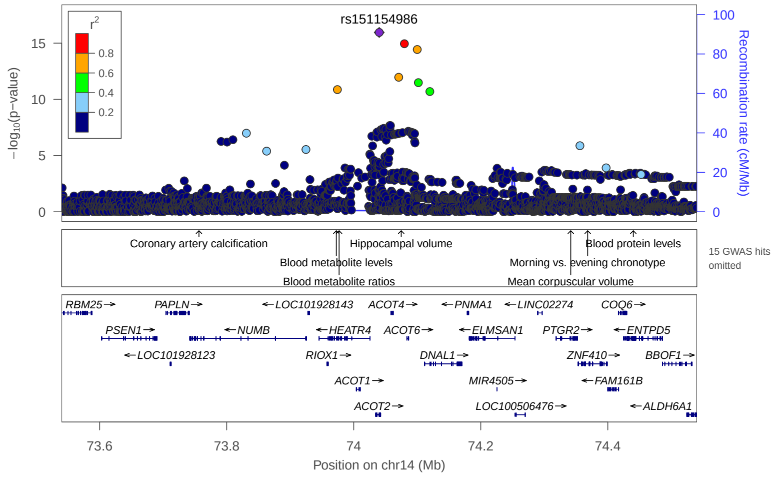

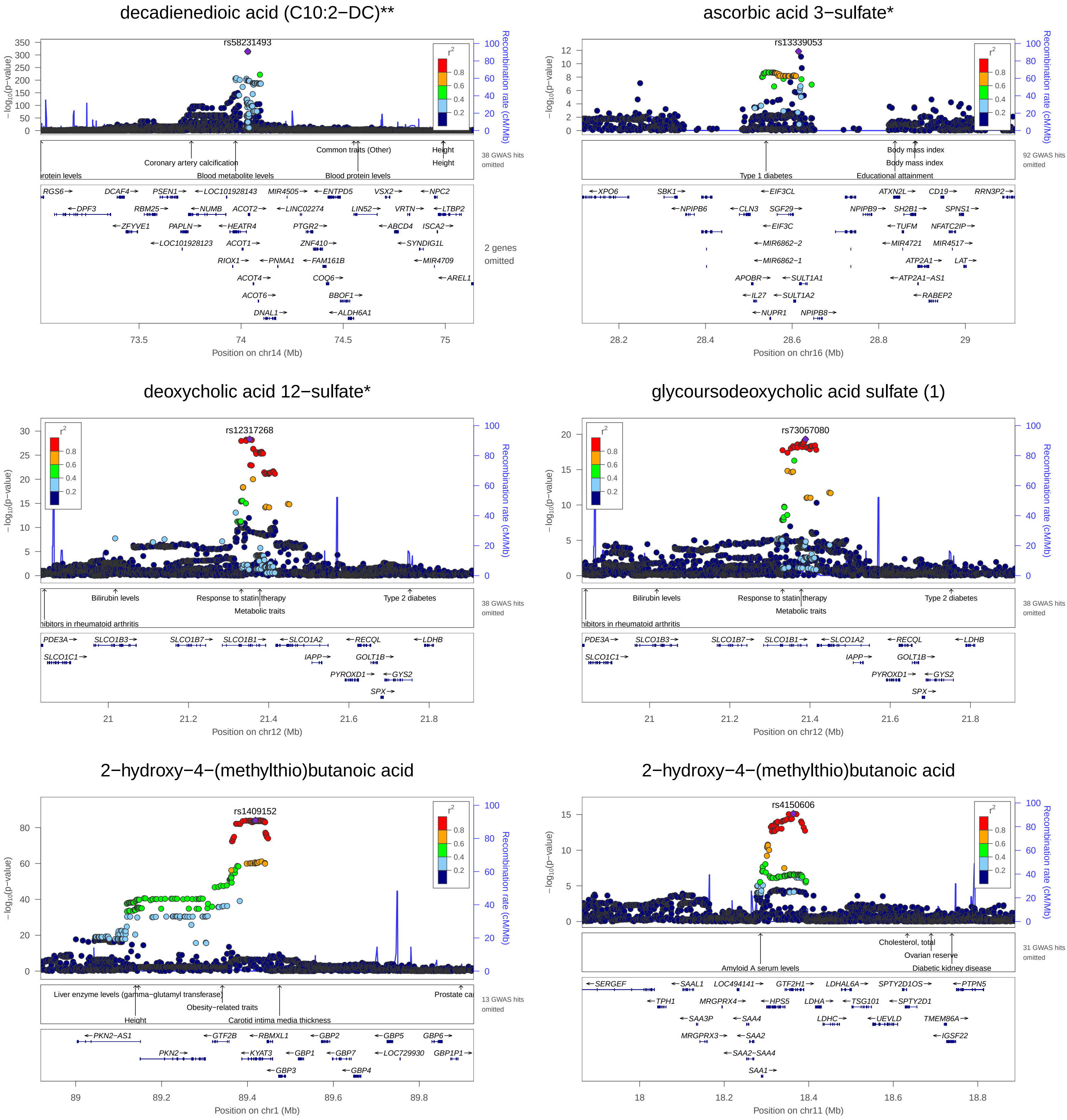
decadienedioic acid (C10:2−DC)**
rs58231493
Recombination rate (cM/Mb)
Position on chr14 (Mb)
38 GWAS hits omitted
2 genes omitted
Coronary artery calcification
Blood metabolite levels
Common traits (Other)
Height
Blood protein levels
RGS6 DCAF4 PSEN1 LOC101928143 MIR4505 ENTPD5 VSX2 NPC2
DPF3 RBM25 NUMB ACOT2 LINC02274 LIN52 VRTN LTBP2
ZFYVE1 PAPLN HEATR4 PTGR2 ABCD4 ISCA2
LOC101928123 ACOT1 ZNF410 SYNDIG1L
RIOX1 PNMA1 FAM161B MIR4709
ACOT4 COQ6 AREL1
ACOT6 BBOF1
DNAL1 ALDH6A1
ascorbic acid 3−sulfate*
rs13339053
Position on chr16 (Mb)
92 GWAS hits omitted
Type 1 diabetes
Body mass index
Educational attainment
XPO6 SBK1 EIF3CL ATXN2L CD19 RRN3P2
NPIPB6 CLN3 SGF29 NPIPB9 SH2B1 SPNS1
EIF3C TUFM NFATC2IP
MIR6862−2 MIR4721 MIR4517
MIR6862−1 ATP2A1 LAT
APOBR SULT1A1 ATP2A1−AS1
IL27 SULT1A2 RABEP2
NUPR1 NPIPB8
deoxycholic acid 12−sulfate*
rs12317268
Position on chr12 (Mb)
38 GWAS hits omitted
Bilirubin levels
Response to statin therapy
Metabolic traits
Type 2 diabetes
PDE3A SLCO1B3 SLCO1B7 SLCO1B1 SLCO1A2 RECQL LDHB
SLCO1C1 IAPP GOLT1B
PYROXD1 GYS2
SPX
glycoursodeoxycholic acid sulfate (1)
rs73067080
Position on chr12 (Mb)
38 GWAS hits omitted
2−hydroxy−4−(methylthio)butanoic acid
rs1409152
Position on chr1 (Mb)
13 GWAS hits omitted
Liver enzyme levels (gamma−glutamyl transferase)
Obesity−related traits
Height
Carotid intima media thickness
PKN2−AS1 GTF2B RBMXL1 GBP2 GBP5 GBP6
PKN2 KYAT3 GBP1 GBP7 LOC729930 GBP1P1
GBP3 GBP4
2−hydroxy−4−(methylthio)butanoic acid
rs4150606
Position on chr11 (Mb)
31 GWAS hits omitted
Cholesterol, total
Ovarian reserve
Amyloid A serum levels
Diabetic kidney disease
SERGEF SAAL1 LOC494141 GTF2H1 LDHAL6A SPTY2D1OS PTPN5
TPH1 MRGPRX4 HPS5 LDHA TSG101 SPTY2D1
SAA3P SAA4 LDHC UEVLD TMEM86A
MRGPRX3 SAA2 IGSF22
SAA2−SAA4
SAA1

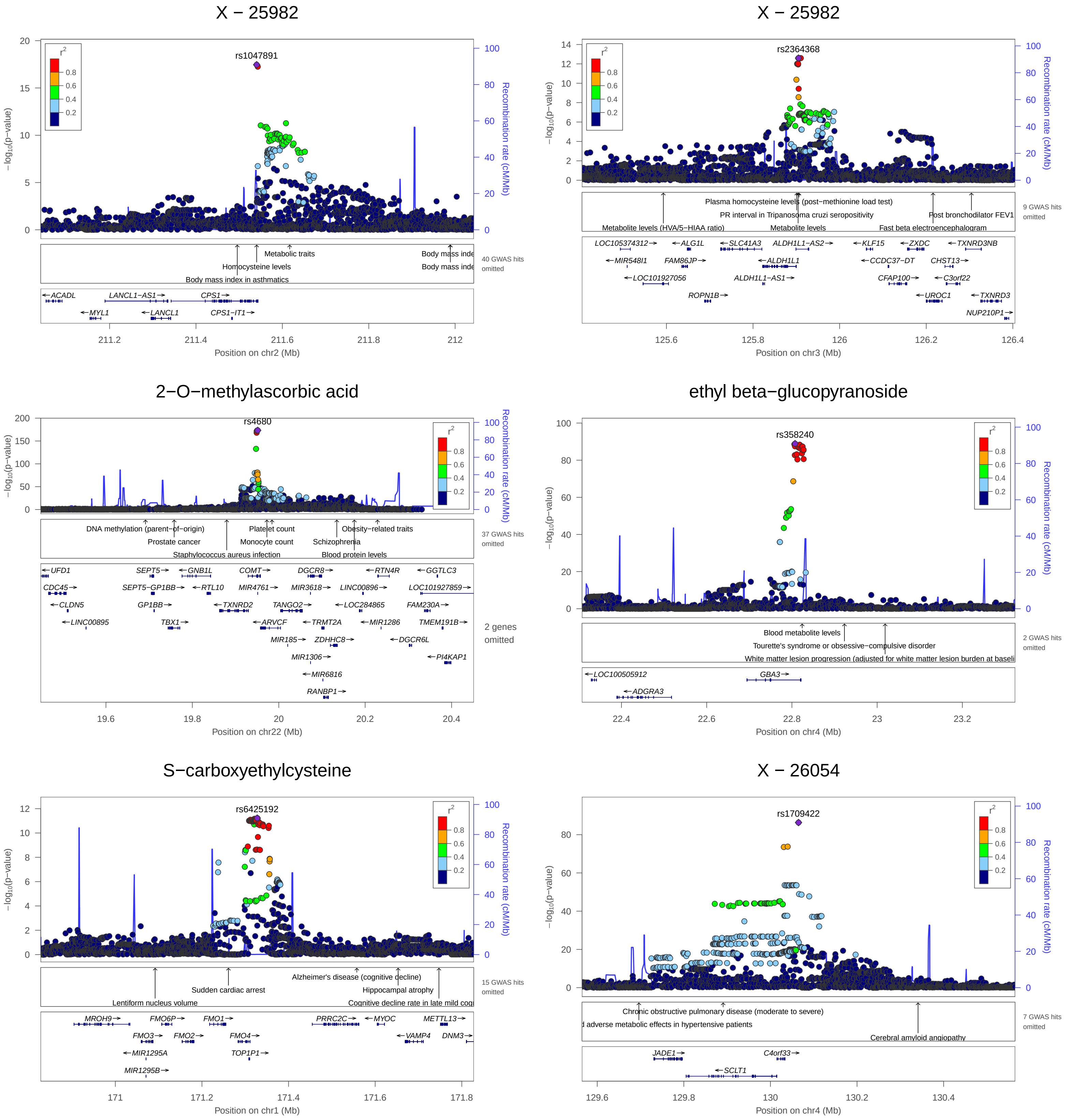

X − 25982
rs1047891
Position on chr2 (Mb)
40 GWAS hits omitted
Metabolic traits
Homocysteine levels
Body mass index in asthmatics
X − 25982
rs2364368
Position on chr3 (Mb)
9 GWAS hits omitted
Plasma homocysteine levels (post−methionine load test)
PR interval in Tripanosoma cruzi seropositivity
Metabolite levels (HVA/5−HIAA ratio)
Metabolite levels
Post bronchodilator FEV1
Fast beta electroencephalogram
2−O−methylascorbic acid
rs4680
Position on chr22 (Mb)
37 GWAS hits omitted
2 genes omitted
DNA methylation (parent−of−origin)
Prostate cancer
Staphylococcus aureus infection
Platelet count
Monocyte count
Schizophrenia
Obesity−related traits
Blood protein levels
ethyl beta−glucopyranoside
rs358240
Position on chr4 (Mb)
2 GWAS hits omitted
Blood metabolite levels
Tourette's syndrome or obsessive−compulsive disorder
S−carboxyethylcysteine
rs6425192
Position on chr1 (Mb)
15 GWAS hits omitted
Sudden cardiac arrest
Lentiform nucleus volume
Alzheimer's disease (cognitive decline)
Hippocampal atrophy
X − 26054
rs1709422
Position on chr4 (Mb)
7 GWAS hits omitted
Chronic obstructive pulmonary disease (moderate to severe)
Cerebral amyloid angiopathy
−log10(p−value)
Recombination rate (cM/Mb)

X − 26054

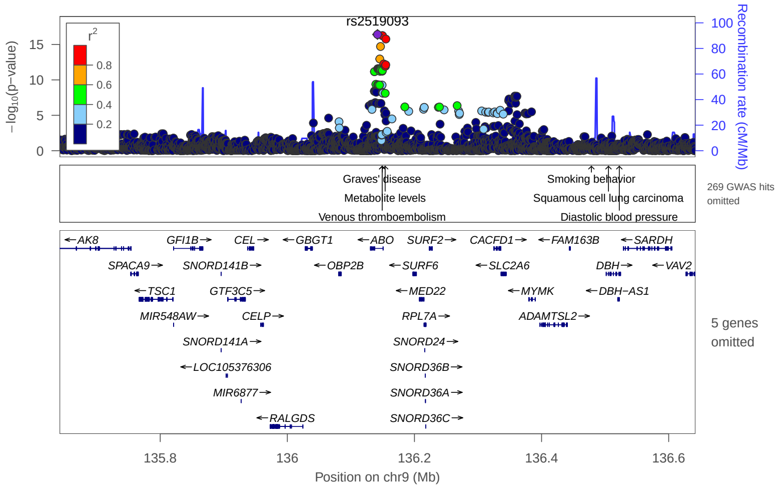

cis−3,4−methyleneheptanoylglycine

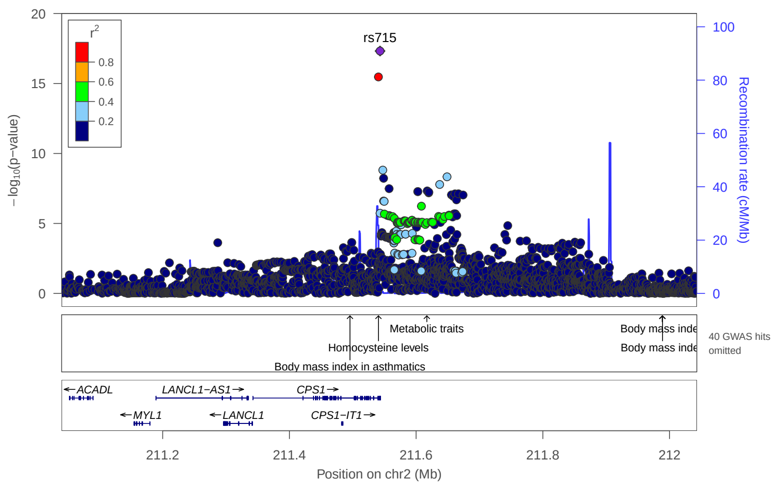

3−hydroxyoctanoylcarnitine (1)

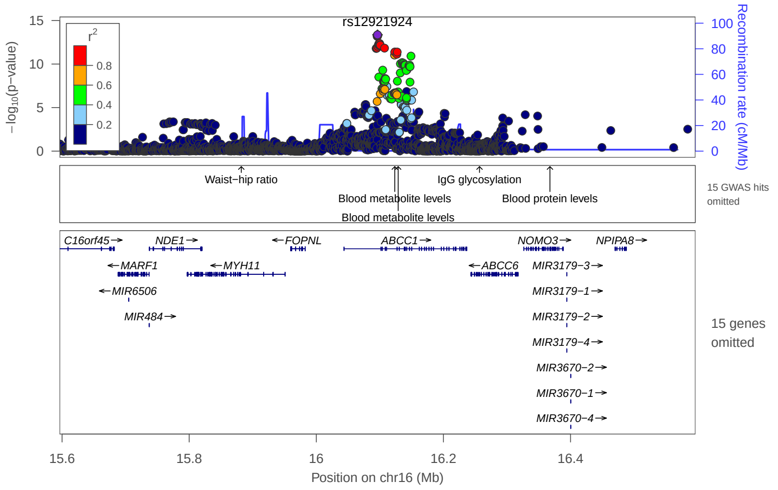

3−hydroxyoctanoylcarnitine (2)

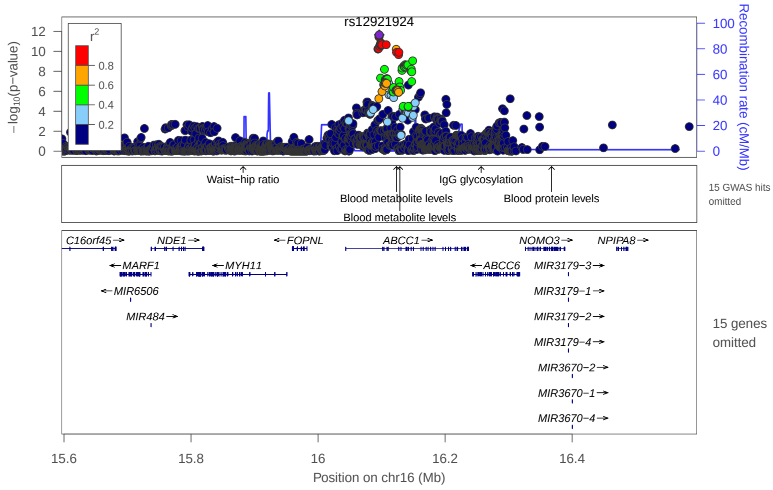

X − 26109

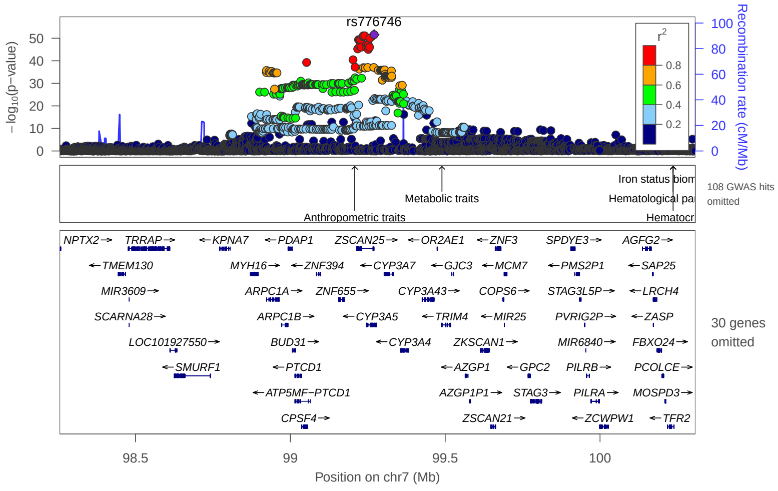

X − 26109

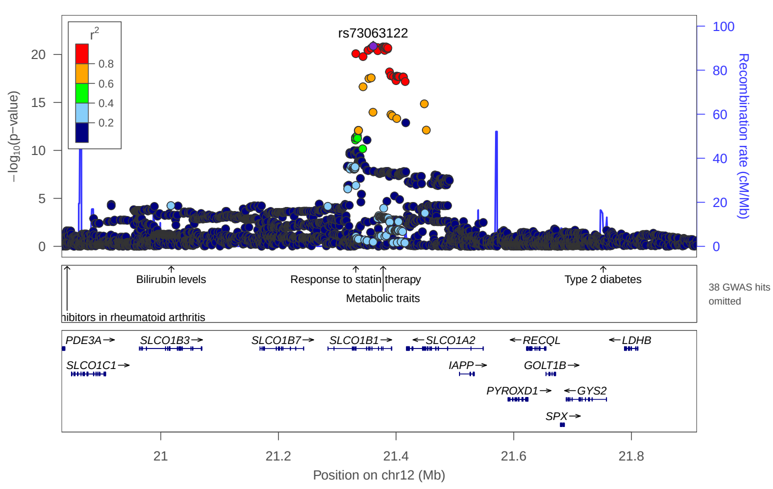

X − 26111

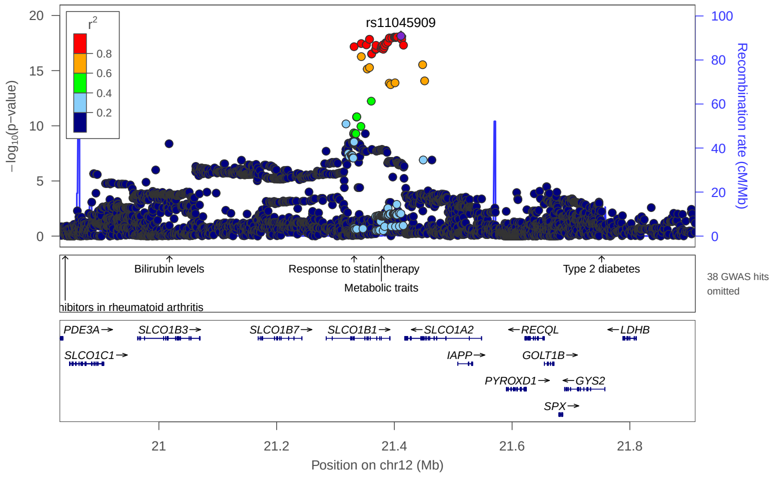

bilirubin degradation product, C16H18N2O5 (1)**

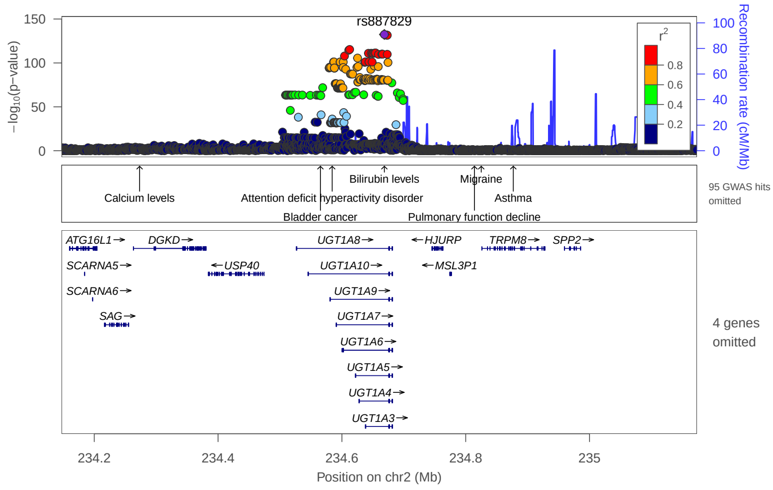

bilirubin degradation product, C16H18N2O5 (2)**

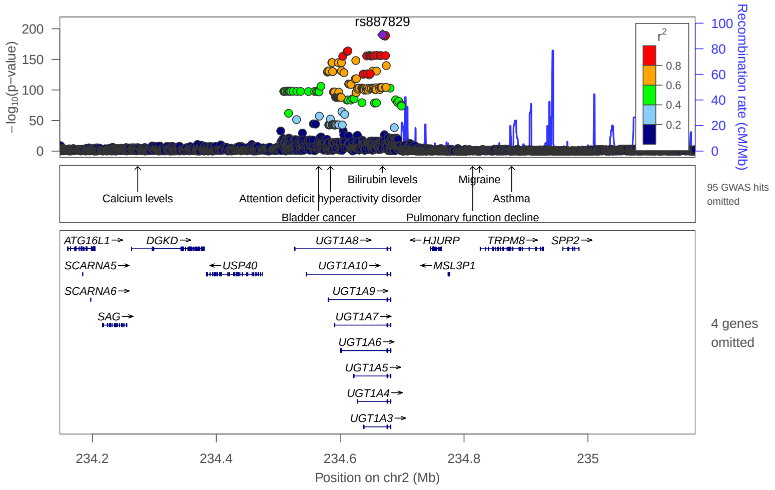

bilirubin degradation product, C17H20N2O5 (1)**

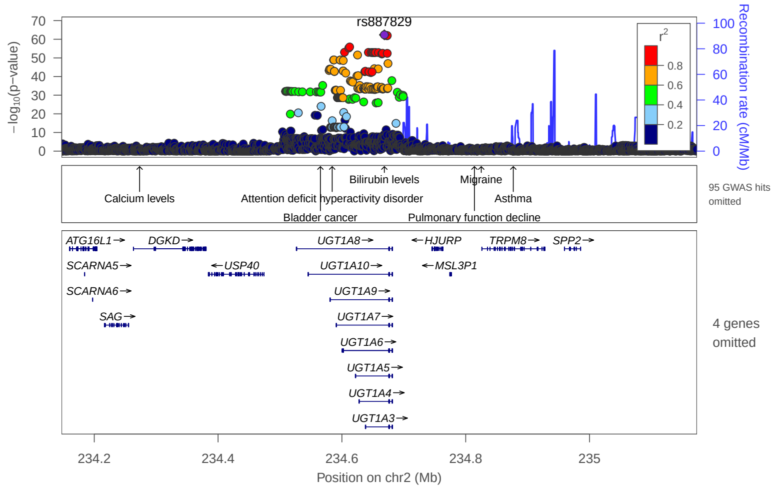

bilirubin degradation product, C17H20N2O5 (2)**

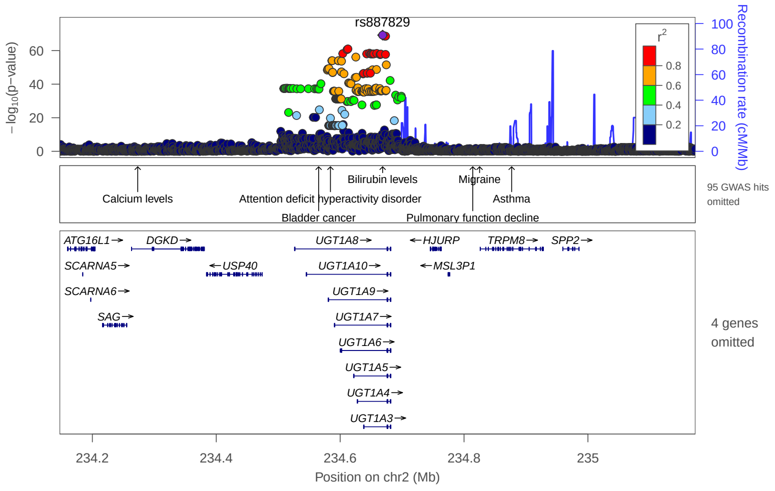

bilirubin degradation product, C17H18N2O4 (2)**

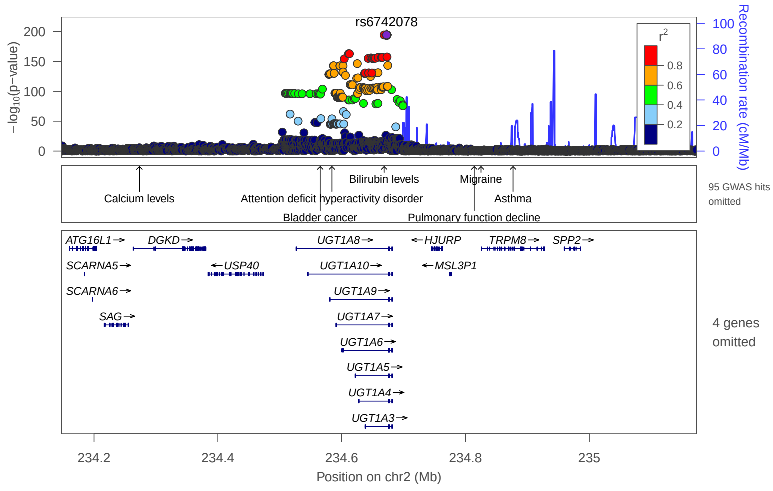

bilirubin degradation product, C17H18N2O4 (3)**

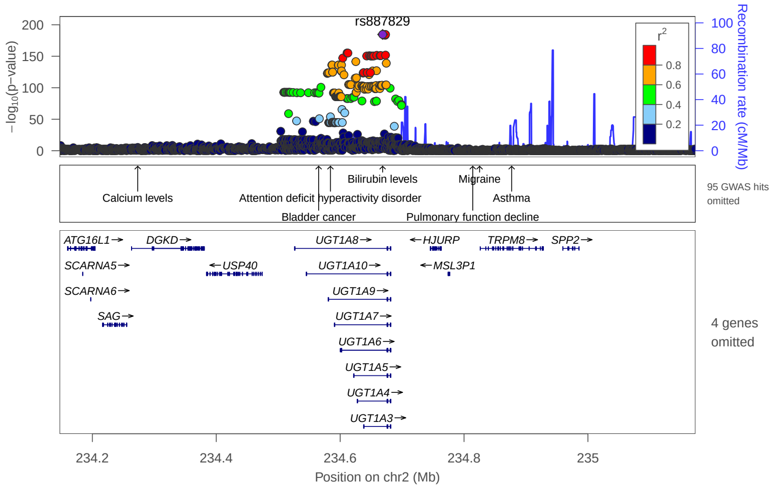

bilirubin degradation product, C17H18N2O4 (1)**

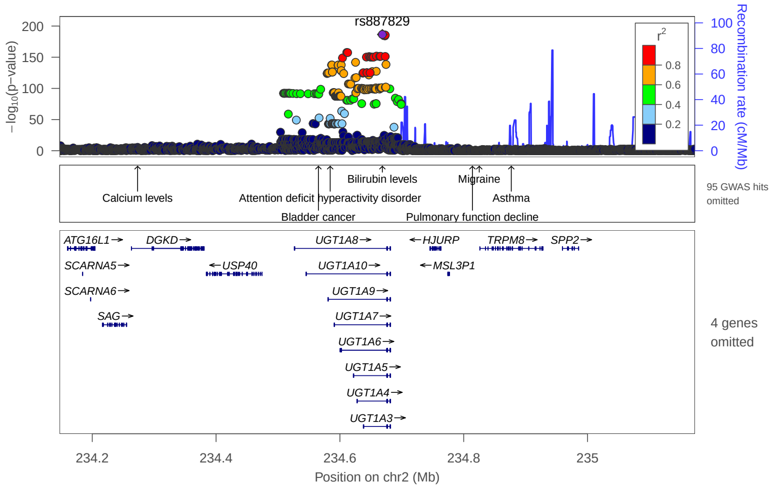

tetrahydrocortisol glucuronide

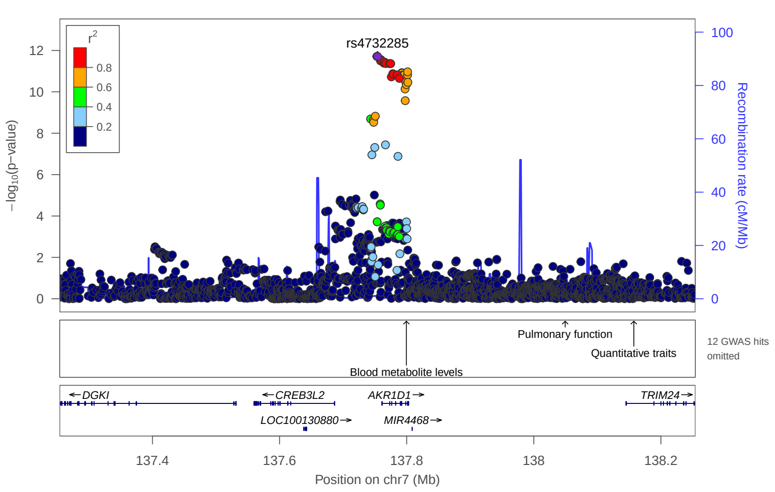

bilirubin degradation product, C16H18N2O5 (4)**

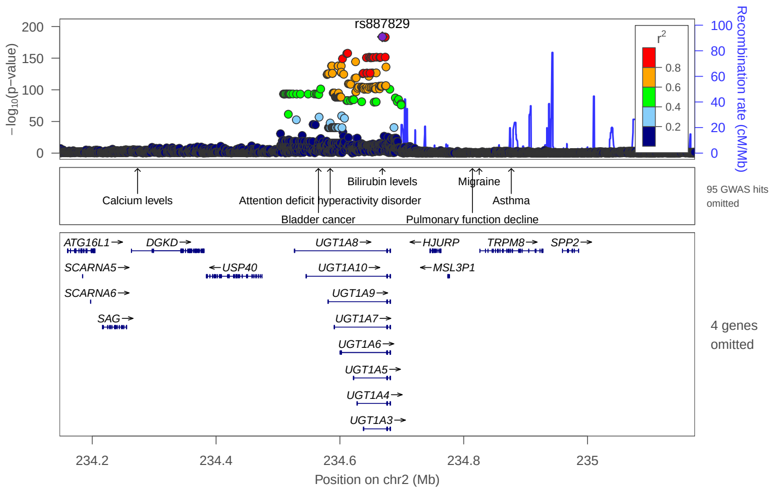

tetrahydrocortisone glucuronide (5)

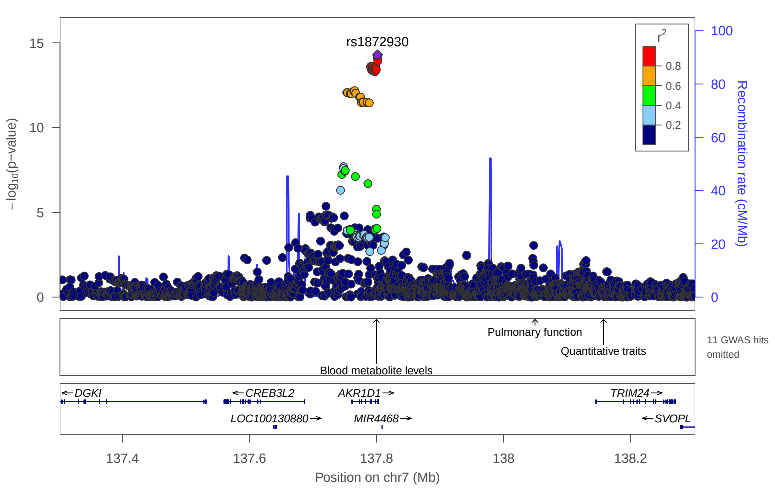

Supplement: Supplementary file 4 — Regional association plots for mQTL identified in mGWAS of plasma metabolite levels. [file 41588_2023_1409_MOESM4_ESM.pdf]
