# Supplementary material for: Genetic studies of paired metabolomes reveal enzymatic and transport processes at the interface of plasma and urine
Source: Nat Genet. 2023 Jun 5;55(6):995–1008. doi: 10.1038/s41588-023-01409-8 (PMC10260405; doi:10.1038/s41588-023-01409-8)
Supplement: Supplementary file 5 — Regional association plots for mQTL identified in mGWAS of urine metabolite levels. [file 41588_2023_1409_MOESM5_ESM.pdf]

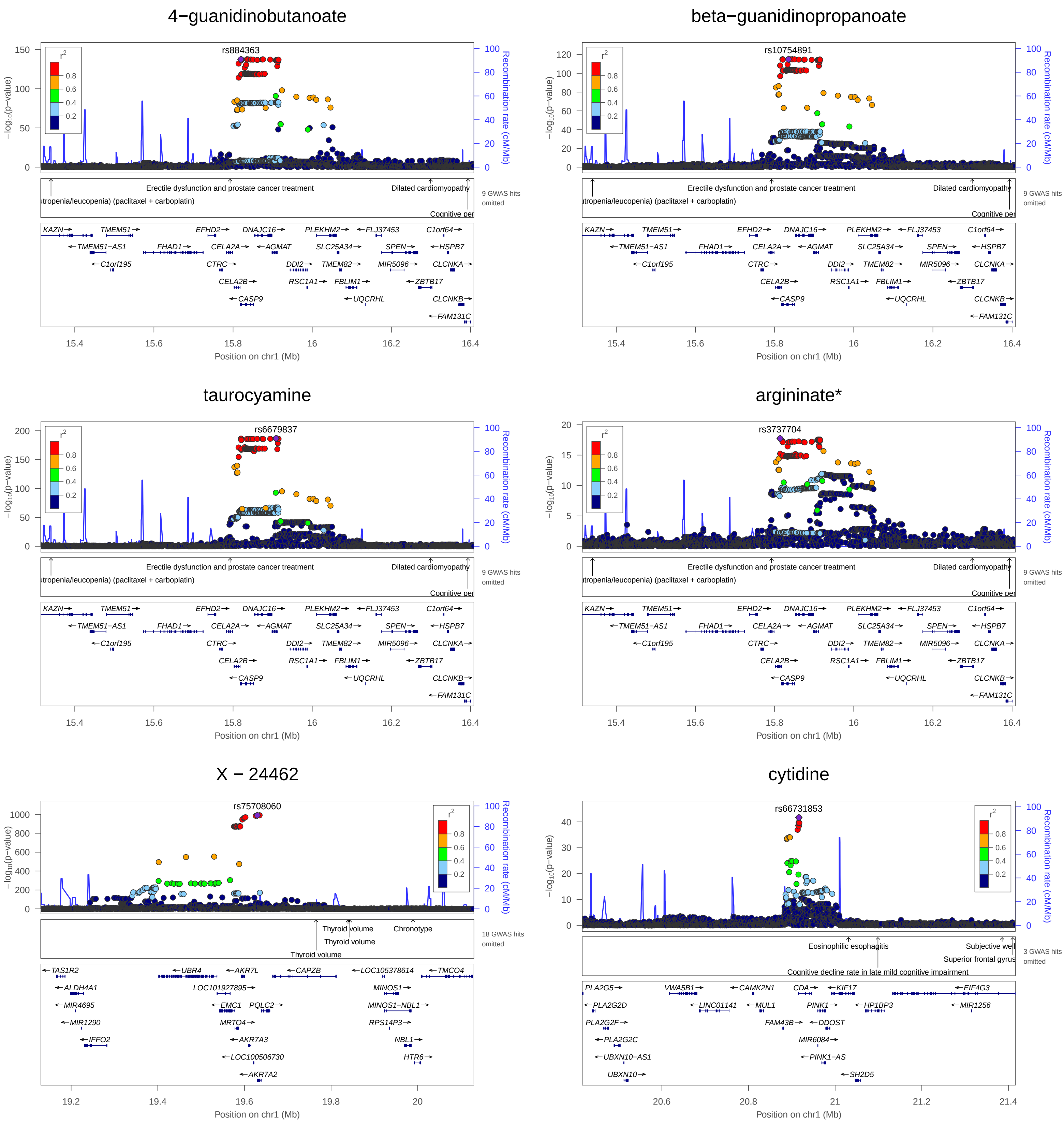
4−guanidinobutanoate
rs884363
beta−guanidinopropanoate
rs10754891
taurocyamine
rs6679837
argininate*
rs3737704
X − 24462
rs75708060
cytidine
rs66731853
−log10(p−value)
Recombination rate (cM/Mb)
r2
Position on chr1 (Mb)
9 GWAS hits omitted
18 GWAS hits omitted
3 GWAS hits omitted
Erectile dysfunction and prostate cancer treatment
Dilated cardiomyopathy
utropenia/leucopenia) (paclitaxel + carboplatin)
Cognitive per
Thyroid volume
Chronotype
Eosinophilic esophagitis
Subjective well
Superior frontal gyrus
Cognitive decline rate in late mild cognitive impairment
KAZN
TMEM51
TMEM51−AS1
C1orf195
FHAD1
EFHD2
CTRC
CELA2A
CELA2B
CASP9
DNAJC16
AGMAT
DDI2
RSC1A1
PLEKHM2
SLC25A34
TMEM82
FBLIM1
UQCRHL
FLJ37453
SPEN
MIR5096
ZBTB17
C1orf64
HSPB7
CLCNKA
CLCNKB
FAM131C
TAS1R2
ALDH4A1
MIR4695
MIR1290
IFFO2
UBR4
LOC101927895
EMC1
MRTO4
AKR7A3
LOC100506730
AKR7A2
AKR7L
PQLC2
CAPZB
LOC105378614
MINOS1
MINOS1−NBL1
RPS14P3
NBL1
HTR6
TMCO4
PLA2G5
PLA2G2D
PLA2G2F
PLA2G2C
UBXN10−AS1
UBXN10
VWA5B1
LINC01141
CAMK2N1
MUL1
FAM43B
CDA
PINK1
DDOST
MIR6084
PINK1−AS
KIF17
HP1BP3
SH2D5
EIF4G3
MIR1256

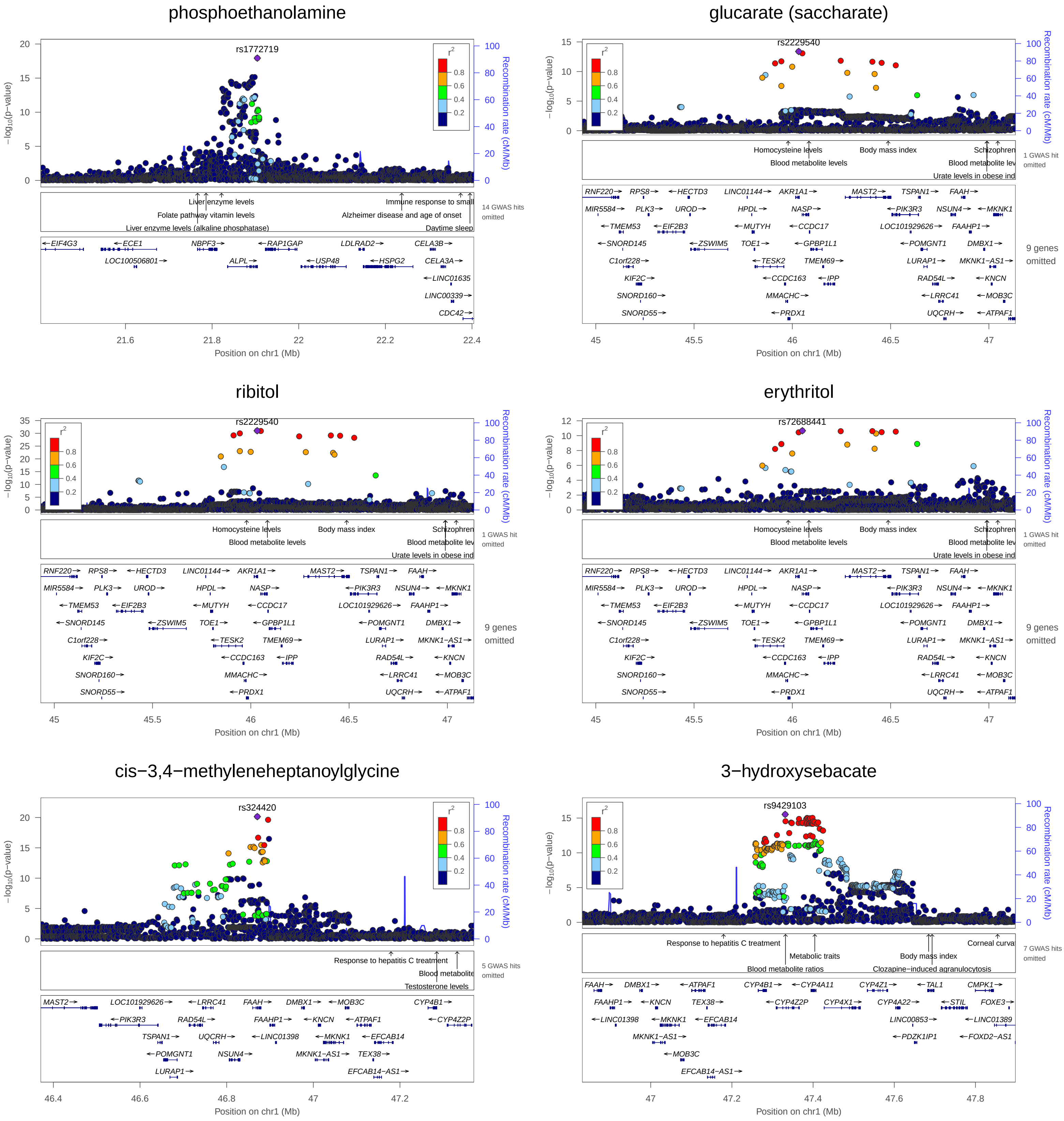

phosphoethanolamine
rs1772719
glucarate (saccharate)
rs2229540
ribitol
rs2229540
erythritol
rs72688441
cis−3,4−methyleneheptanoylglycine
rs324420
3−hydroxysebacate
rs9429103
−log10(p−value)
Recombination rate (cM/Mb)
r2
0.8
0.6
0.4
0.2
Liver enzyme levels
Folate pathway vitamin levels
Liver enzyme levels (alkaline phosphatase)
Immune response to small
Alzheimer disease and age of onset
Daytime sleep
14 GWAS hits omitted
Homocysteine levels
Blood metabolite levels
Body mass index
Schizophren
Blood metabolite lev
Urate levels in obese ind
1 GWAS hit omitted
9 genes omitted
Response to hepatitis C treatment
Blood metabolite
Testosterone levels
5 GWAS hits omitted
Metabolic traits
Blood metabolite ratios
Body mass index
Clozapine−induced agranulocytosis
Corneal curva
7 GWAS hits omitted
Position on chr1 (Mb)

X − 21827

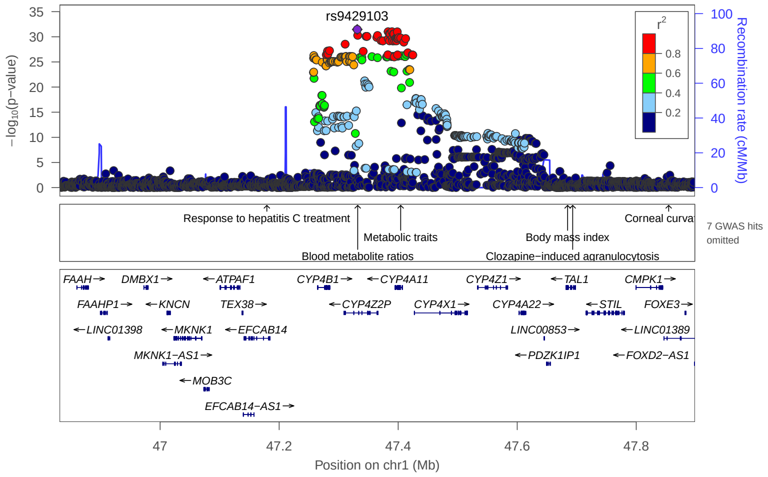

X − 21829

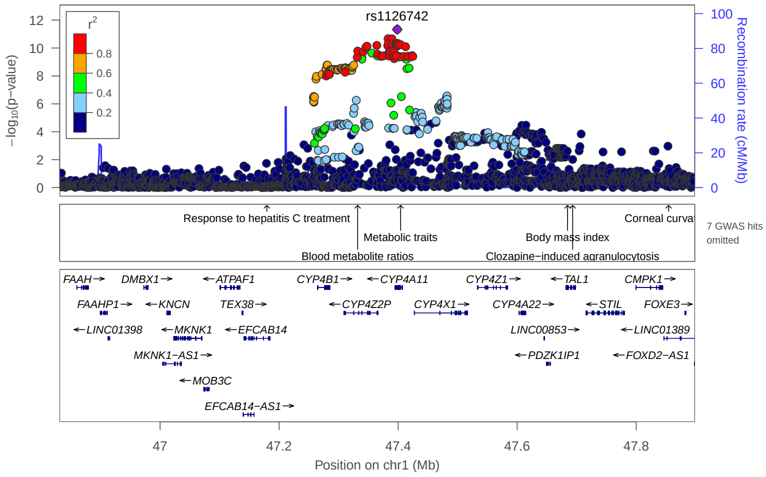

X − 12839

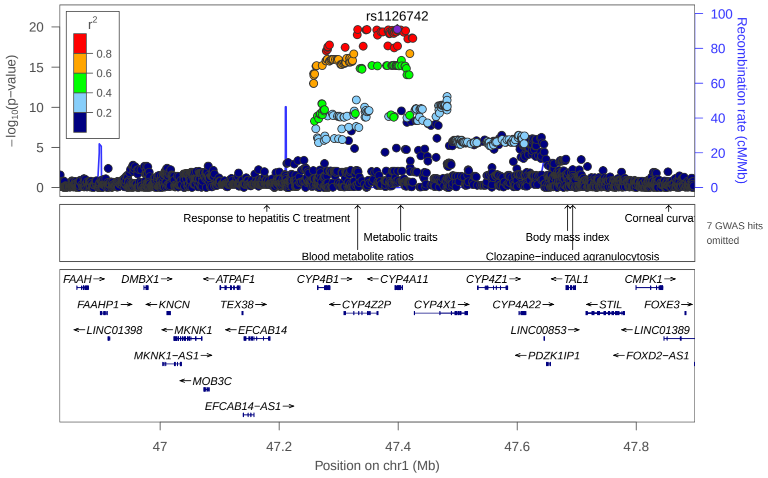

X − 17324

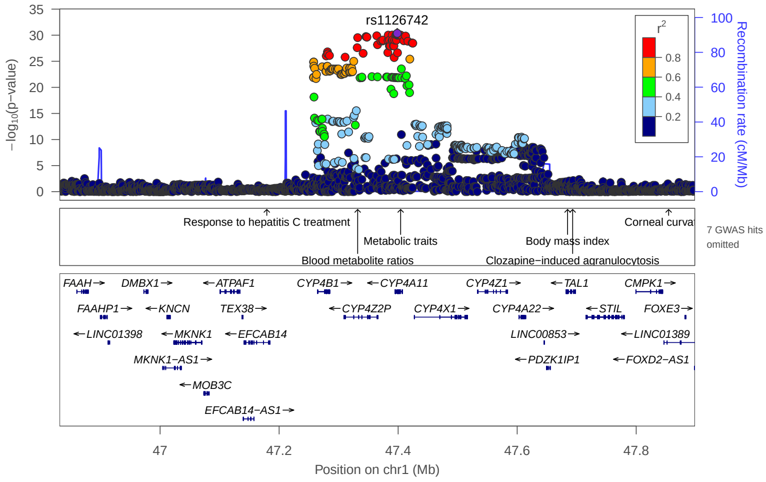

X − 13874

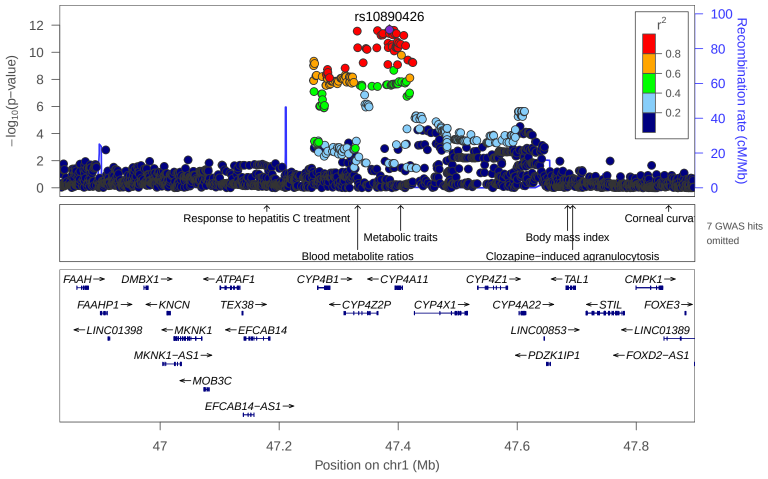

3−hydroxyoctanoyl glycine

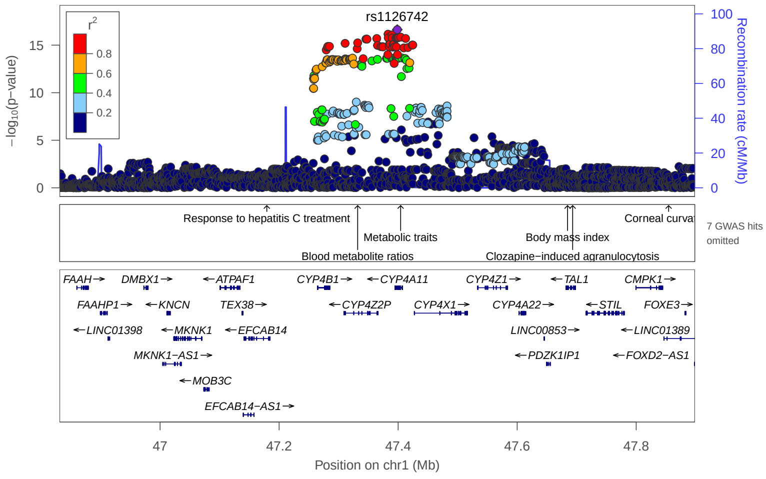

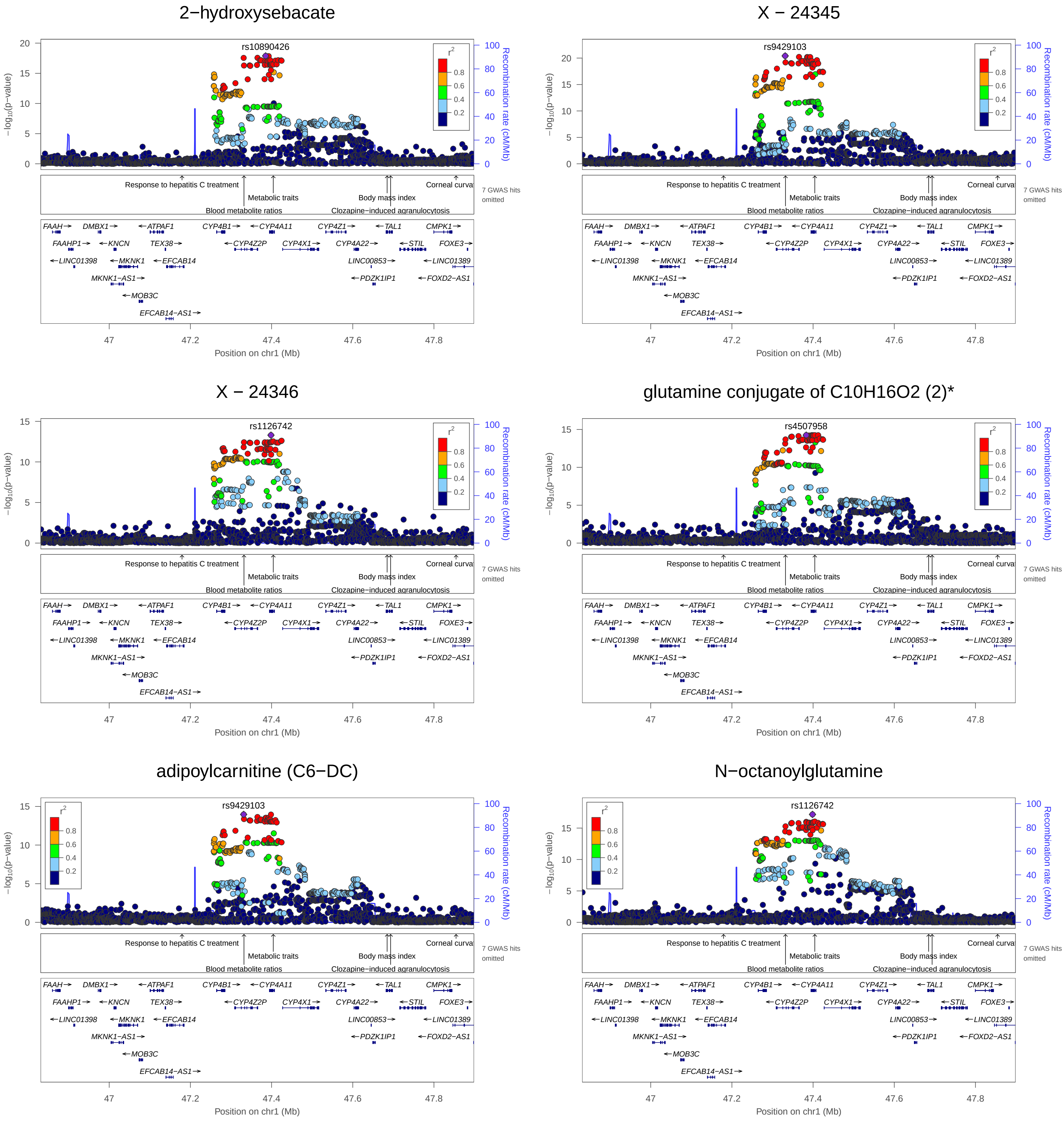

2−hydroxysebacate
rs10890426
X − 24345
rs9429103
X − 24346
rs1126742
glutamine conjugate of C10H16O2 (2)*
rs4507958
adipoylcarnitine (C6−DC)
rs9429103
N−octanoylglutamine
rs1126742
−log10(p−value)
Recombination rate (cM/Mb)
r2
0.8
0.6
0.4
0.2
Response to hepatitis C treatment
Metabolic traits
Body mass index
Corneal curva
Blood metabolite ratios
Clozapine−induced agranulocytosis
7 GWAS hits omitted
FAAH
DMBX1
ATPAF1
CYP4B1
CYP4A11
CYP4Z1
TAL1
CMPK1
FAAHP1
KNCN
TEX38
CYP4Z2P
CYP4X1
CYP4A22
STIL
FOXE3
LINC01398
MKNK1
EFCAB14
LINC00853
LINC01389
MKNK1−AS1
PDZK1IP1
FOXD2−AS1
MOB3C
EFCAB14−AS1
47
47.2
47.4
47.6
47.8
Position on chr1 (Mb)

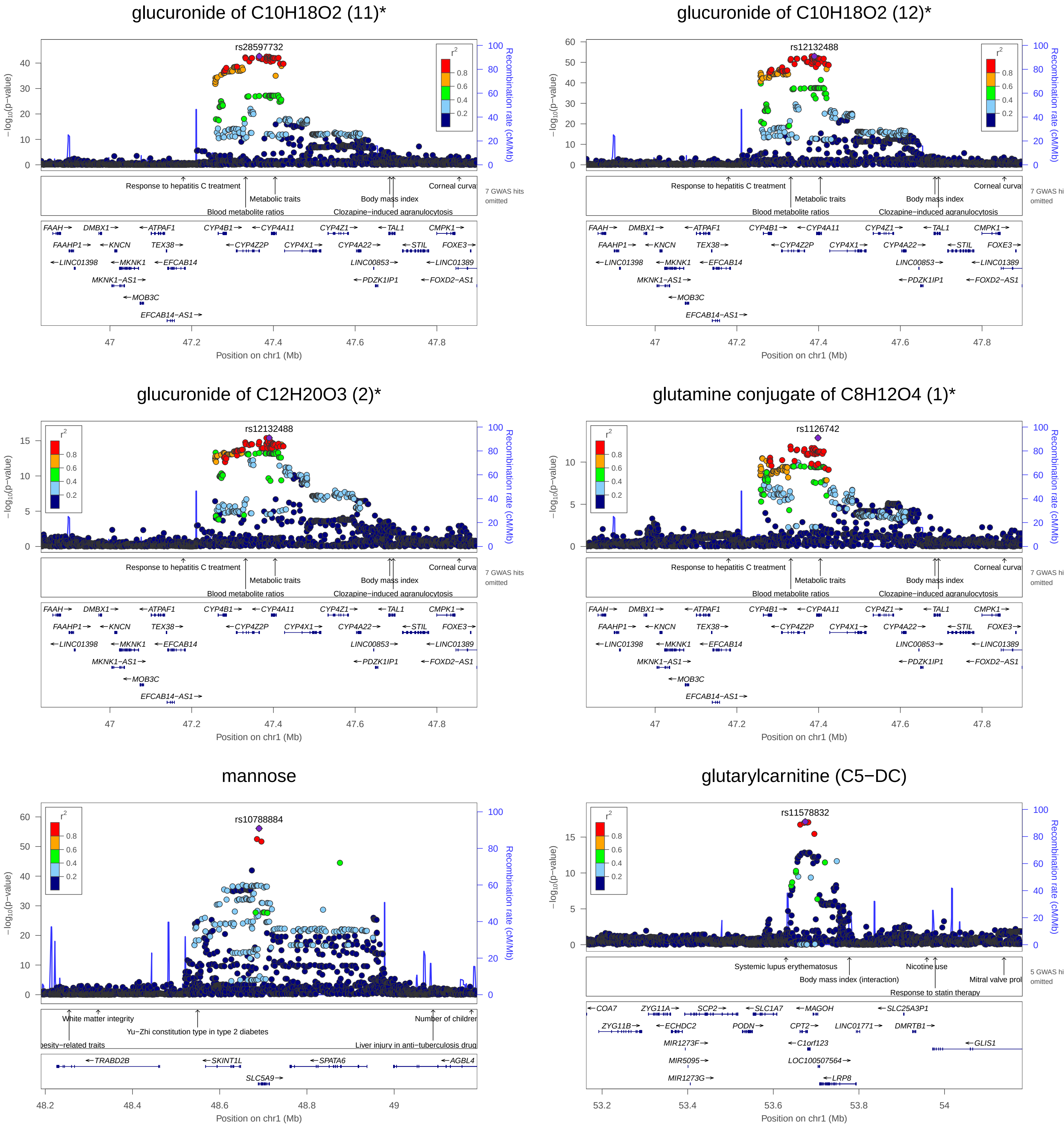
glucuronide of C10H18O2 (11)*
rs28597732
glucuronide of C10H18O2 (12)*
rs12132488
glucuronide of C12H20O3 (2)*
rs12132488
glutamine conjugate of C8H12O4 (1)*
rs1126742
mannose
rs10788884
glutarylcarnitine (C5−DC)
rs11578832
−log10(p−value)
Recombination rate (cM/Mb)
r2
0.8
0.6
0.4
0.2
Response to hepatitis C treatment
Metabolic traits
Body mass index
Corneal curva
Blood metabolite ratios
Clozapine−induced agranulocytosis
7 GWAS hits omitted
FAAH
DMBX1
ATPAF1
CYP4B1
CYP4A11
CYP4Z1
TAL1
CMPK1
FAAHP1
KNCN
TEX38
CYP4Z2P
CYP4X1
CYP4A22
STIL
FOXE3
LINC01398
MKNK1
EFCAB14
LINC00853
LINC01389
MKNK1−AS1
PDZK1IP1
FOXD2−AS1
MOB3C
EFCAB14−AS1
Position on chr1 (Mb)
White matter integrity
Yu−Zhi constitution type in type 2 diabetes
Number of childre
besity−related traits
Liver injury in anti−tuberculosis drug
TRABD2B
SKINT1L
SPATA6
AGBL4
SLC5A9
Systemic lupus erythematosus
Nicotine use
Body mass index (interaction)
Mitral valve prol
Response to statin therapy
5 GWAS hits omitted
COA7
ZYG11A
SCP2
SLC1A7
MAGOH
SLC25A3P1
ZYG11B
ECHDC2
PODN
CPT2
LINC01771
DMRTB1
MIR1273F
C1orf123
GLIS1
MIR5095
LOC100507564
MIR1273G
LRP8

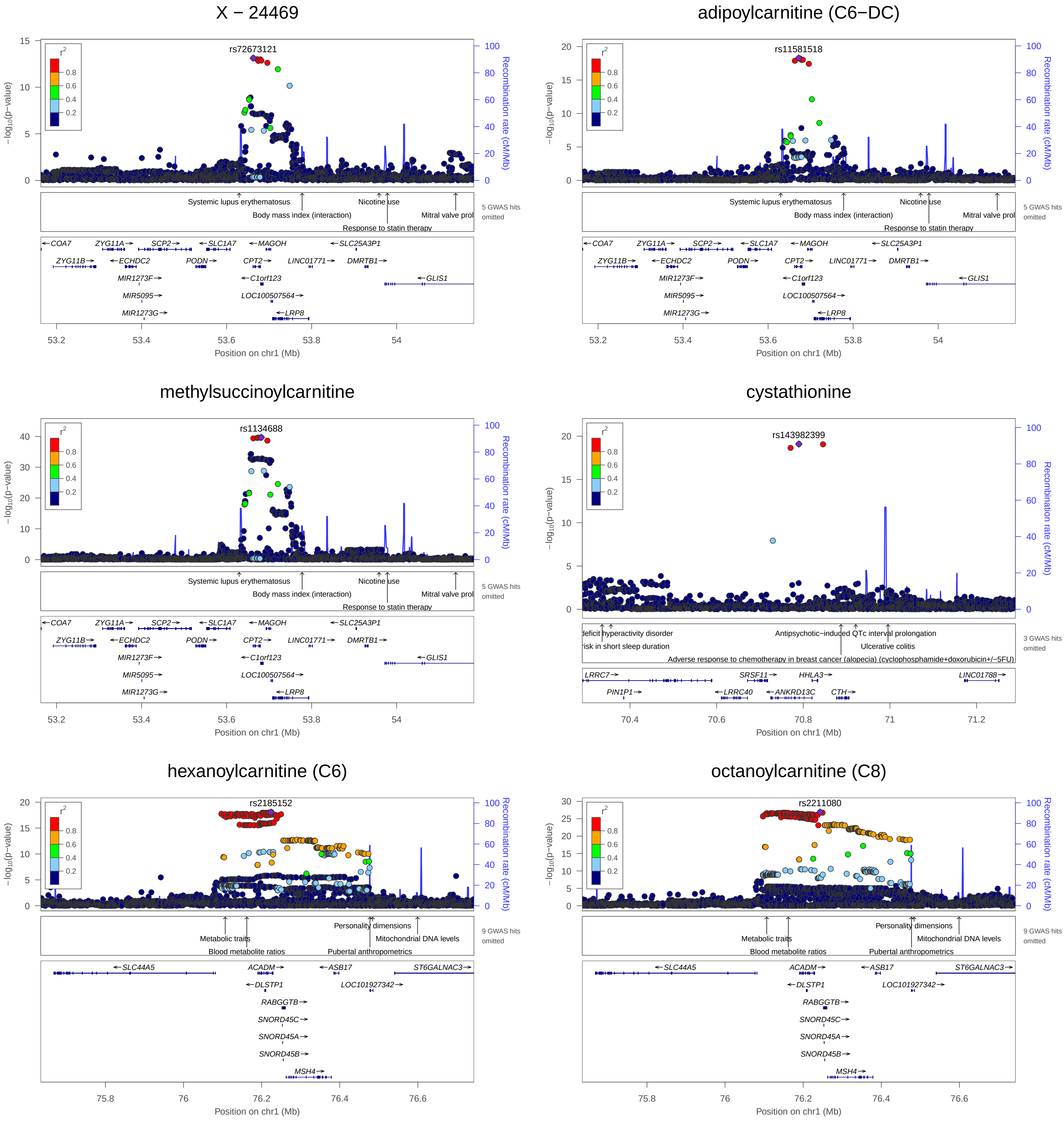
X − 24469
rs72673121
adipoylcarnitine (C6−DC)
rs11581518
methylsuccinoylcarnitine
rs1134688
cystathionine
rs143982399
hexanoylcarnitine (C6)
rs2185152
octanoylcarnitine (C8)
rs2211080
r2
0.8
0.6
0.4
0.2
−log10(p−value)
Recombination rate (cM/Mb)
Systemic lupus erythematosus
Nicotine use
Body mass index (interaction)
Mitral valve prol
Response to statin therapy
5 GWAS hits omitted
COA7
ZYG11A
SCP2
SLC1A7
MAGOH
SLC25A3P1
ZYG11B
ECHDC2
PODN
CPT2
LINC01771
DMRTB1
MIR1273F
C1orf123
GLIS1
MIR5095
LOC100507564
MIR1273G
LRP8
Position on chr1 (Mb)
deficit hyperactivity disorder
risk in short sleep duration
Antipsychotic−induced QTc interval prolongation
Ulcerative colitis
Adverse response to chemotherapy in breast cancer (alopecia) (cyclophosphamide+doxorubicin+/−5FU)
3 GWAS hits omitted
LRRC7
SRSF11
HHLA3
LINC01788
PIN1P1
LRRC40
ANKRD13C
CTH
Personality dimensions
Metabolic traits
Mitochondrial DNA levels
Blood metabolite ratios
Pubertal anthropometrics
9 GWAS hits omitted
SLC44A5
ACADM
ASB17
ST6GALNAC3
DLSTP1
LOC101927342
RABGGTB
SNORD45C
SNORD45A
SNORD45B
MSH4

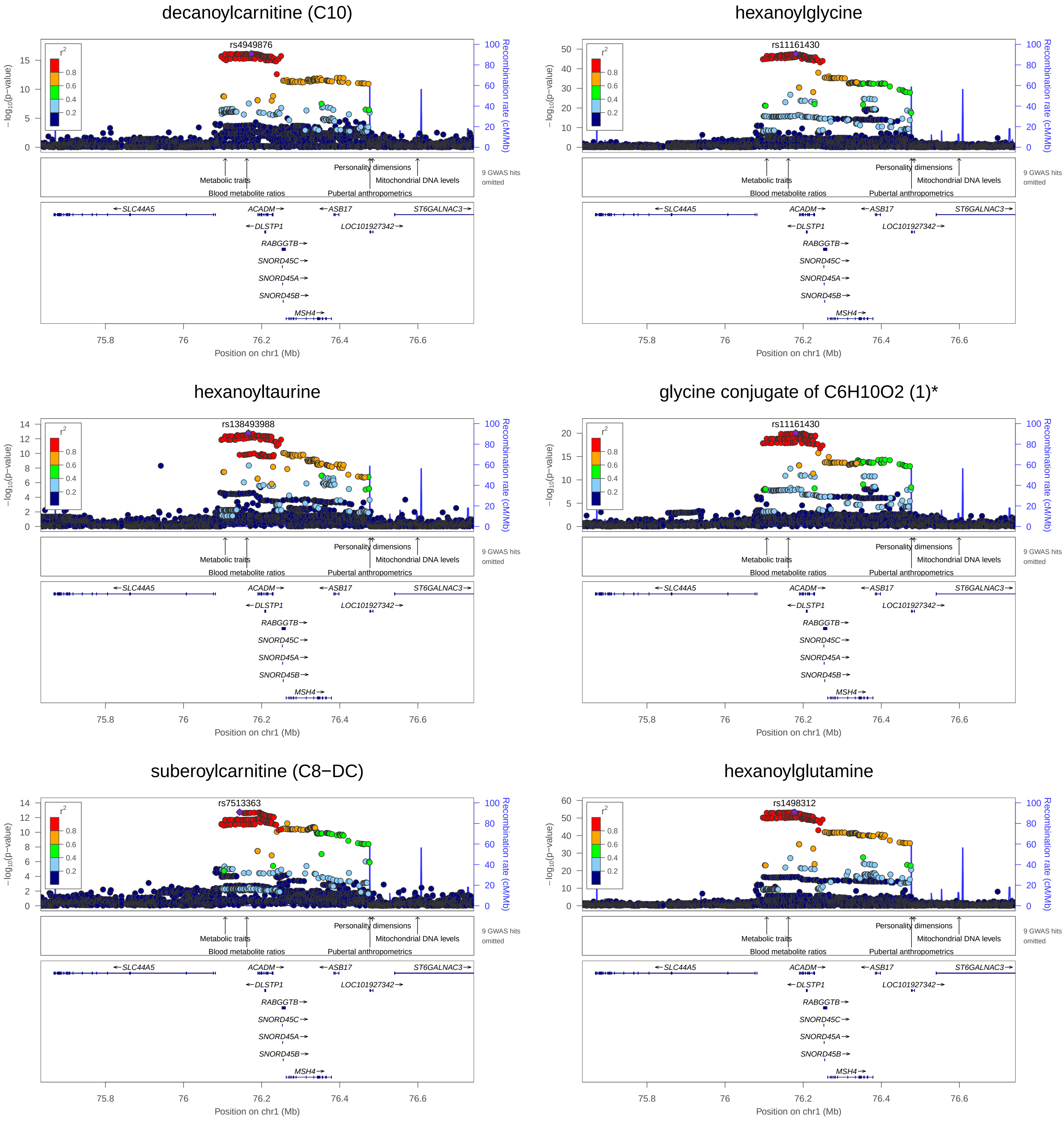
decanoylcarnitine (C10)
rs4949876
hexanoylglycine
rs11161430
hexanoyltaurine
rs138493988
glycine conjugate of C6H10O2 (1)*
rs11161430
suberoylcarnitine (C8−DC)
rs7513363
hexanoylglutamine
rs1498312
−log10(p−value)
Recombination rate (cM/Mb)
r2
Metabolic traits
Blood metabolite ratios
Personality dimensions
Pubertal anthropometrics
Mitochondrial DNA levels
9 GWAS hits omitted
SLC44A5
ACADM
DLSTP1
RABGGTB
SNORD45C
SNORD45A
SNORD45B
MSH4
ASB17
LOC101927342
ST6GALNAC3
Position on chr1 (Mb)

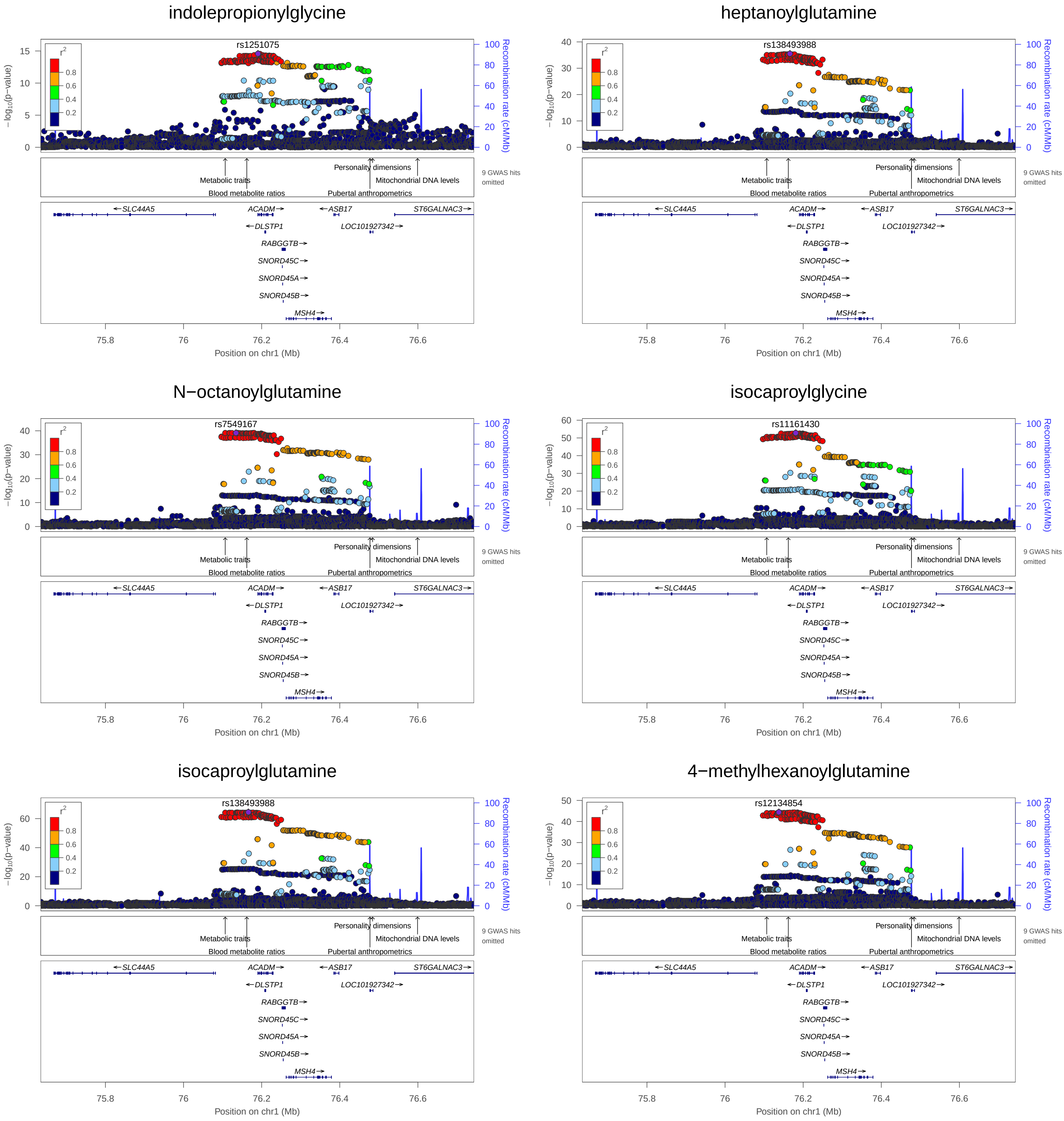
indolepropionylglycine
rs1251075
heptanoylglutamine
rs138493988
N−octanoylglutamine
rs7549167
isocaproylglycine
rs11161430
isocaproylglutamine
rs138493988
4−methylhexanoylglutamine
rs12134854
−log10(p−value)
Recombination rate (cM/Mb)
r2
0.8
0.6
0.4
0.2
Metabolic traits
Blood metabolite ratios
Personality dimensions
Pubertal anthropometrics
Mitochondrial DNA levels
9 GWAS hits omitted
SLC44A5
ACADM
ASB17
ST6GALNAC3
DLSTP1
LOC101927342
RABGGTB
SNORD45C
SNORD45A
SNORD45B
MSH4
75.8
76
76.2
76.4
76.6
Position on chr1 (Mb)

4−methylhexanoylglycine

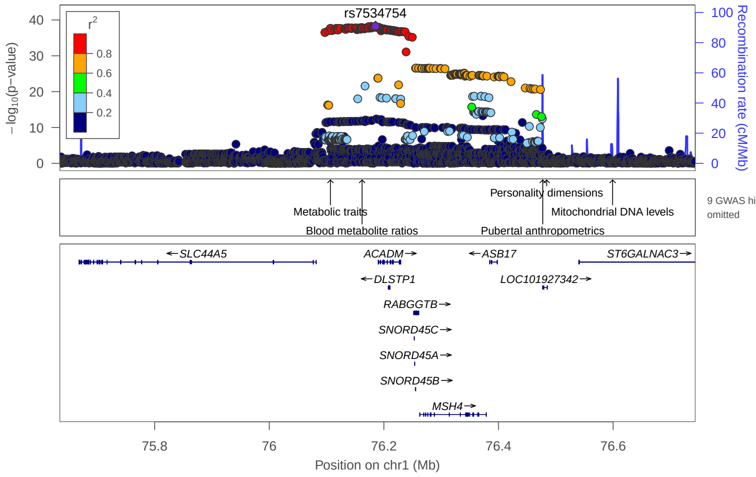

X − 12097

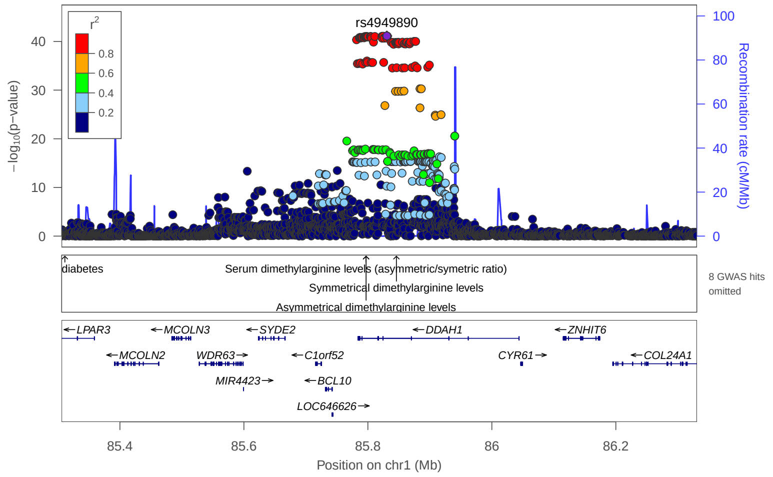

X − 24518

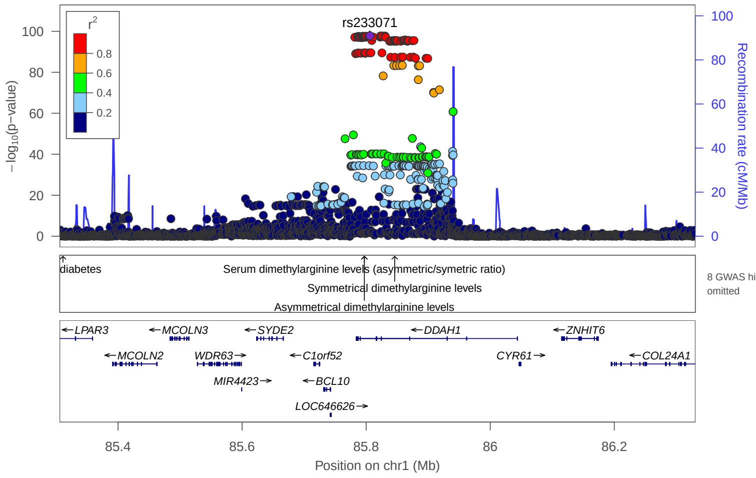

imidazole lactate

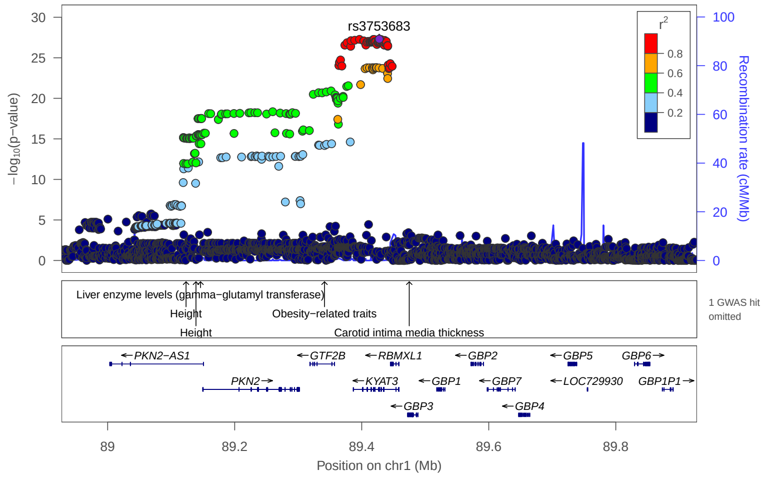

3,7−dimethylurate

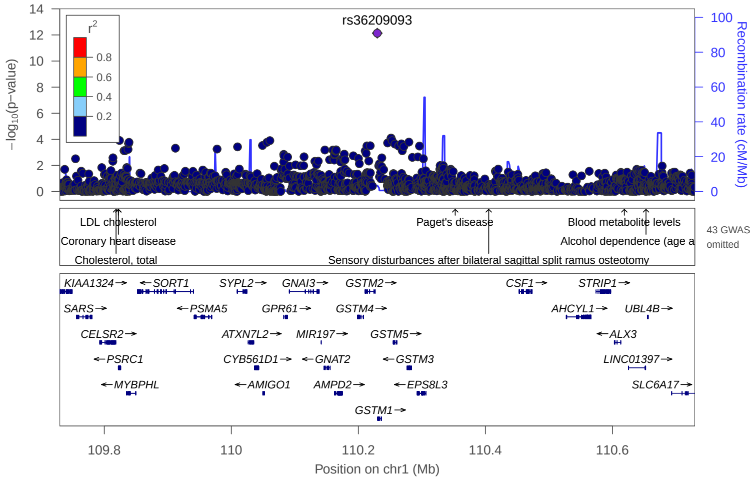

X − 21796

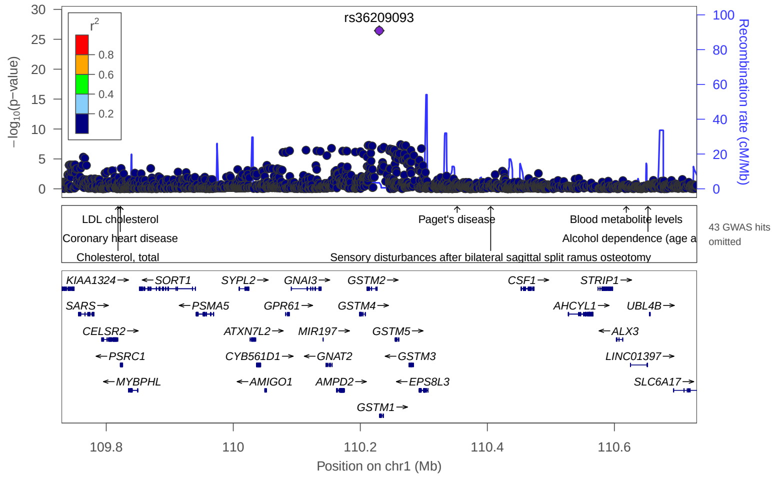

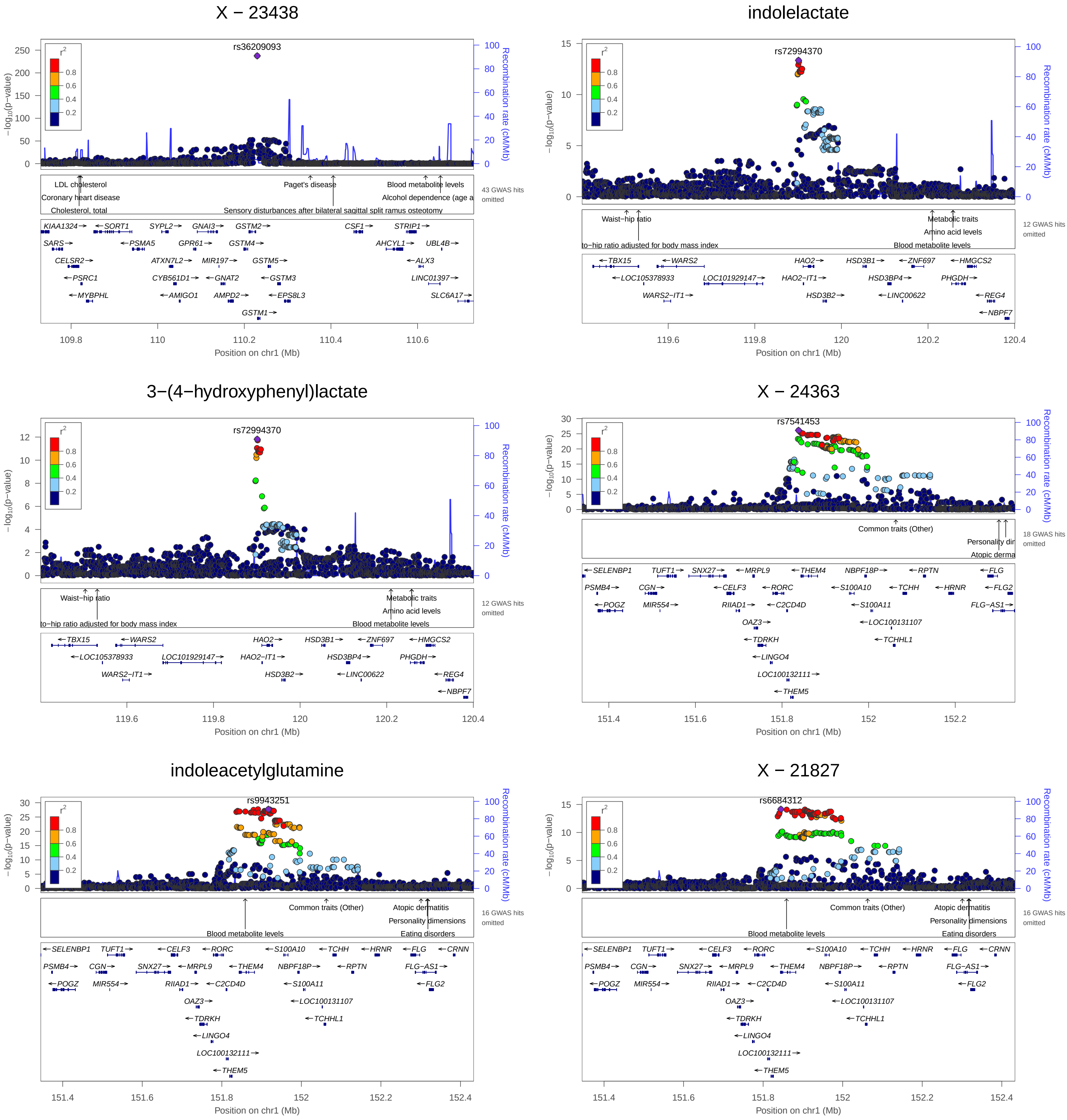

X − 23438
rs36209093
indolelactate
rs72994370
3−(4−hydroxyphenyl)lactate
rs72994370
X − 24363
rs7541453
indoleacetylglutamine
rs9943251
X − 21827
rs6684312
−log10(p−value)
Recombination rate (cM/Mb)
Position on chr1 (Mb)
43 GWAS hits omitted
12 GWAS hits omitted
18 GWAS hits omitted
16 GWAS hits omitted
LDL cholesterol
Coronary heart disease
Cholesterol, total
Paget's disease
Sensory disturbances after bilateral sagittal split ramus osteotomy
Blood metabolite levels
Waist−hip ratio
to−hip ratio adjusted for body mass index
Metabolic traits
Amino acid levels
Common traits (Other)
Atopic dermatitis
Personality dimensions
Eating disorders
KIAA1324 SORT1 SYPL2 GNAI3 GSTM2 CSF1 STRIP1
SARS PSMA5 GPR61 GSTM4 AHCYL1 UBL4B
CELSR2 ATXN7L2 MIR197 GSTM5 ALX3
PSRC1 CYB561D1 GNAT2 GSTM3 LINC01397
MYBPHL AMIGO1 AMPD2 EPS8L3 SLC6A17
GSTM1
TBX15 WARS2 HAO2 HSD3B1 ZNF697 HMGCS2
LOC105378933 LOC101929147 HAO2−IT1 HSD3BP4 PHGDH
WARS2−IT1 HSD3B2 LINC00622 REG4
NBPF7
SELENBP1 TUFT1 SNX27 MRPL9 THEM4 NBPF18P RPTN FLG
PSMB4 CGN CELF3 RORC S100A10 TCHH HRNR FLG2
POGZ MIR554 RIIAD1 C2CD4D S100A11 FLG−AS1
OAZ3 LOC100131107
TDRKH TCHHL1
LINGO4
LOC100132111
THEM5
CRNN

X − 13431

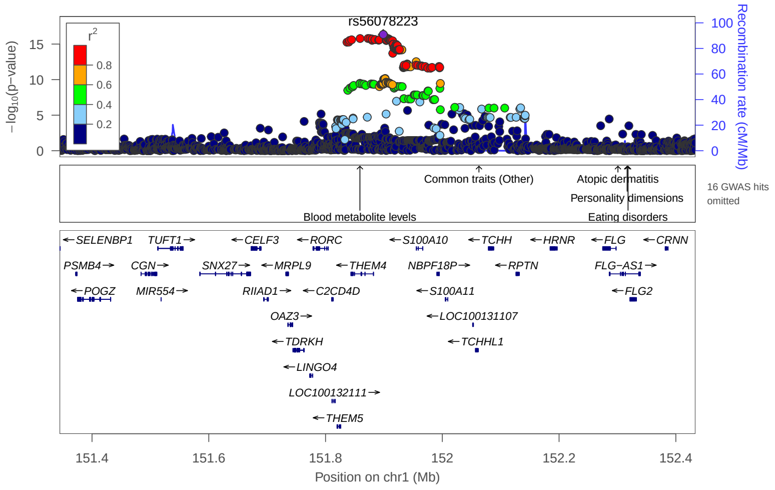

X − 13874

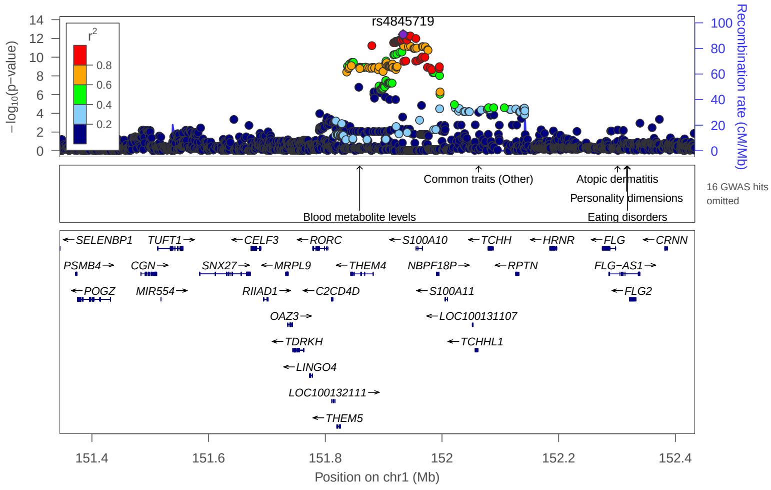

X − 23680

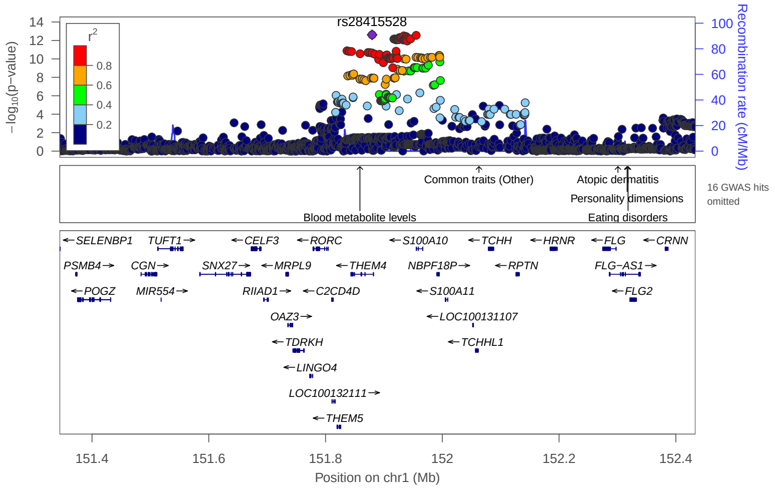

4−hydroxyphenylacetoylcarnitine

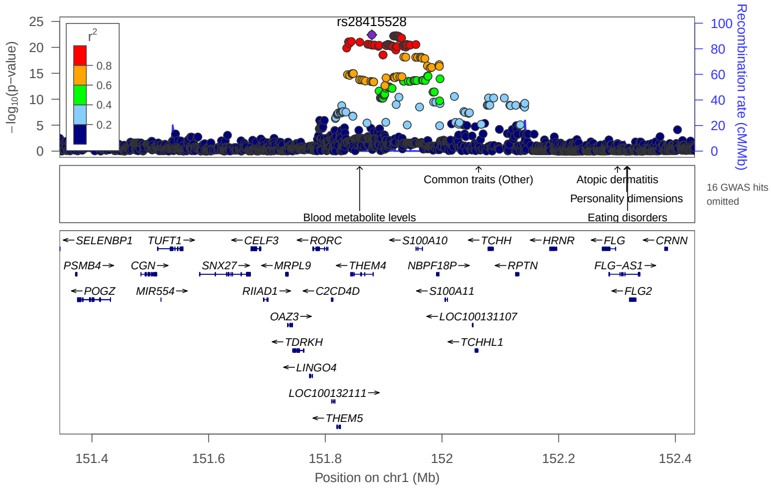

indoleacetylglycine

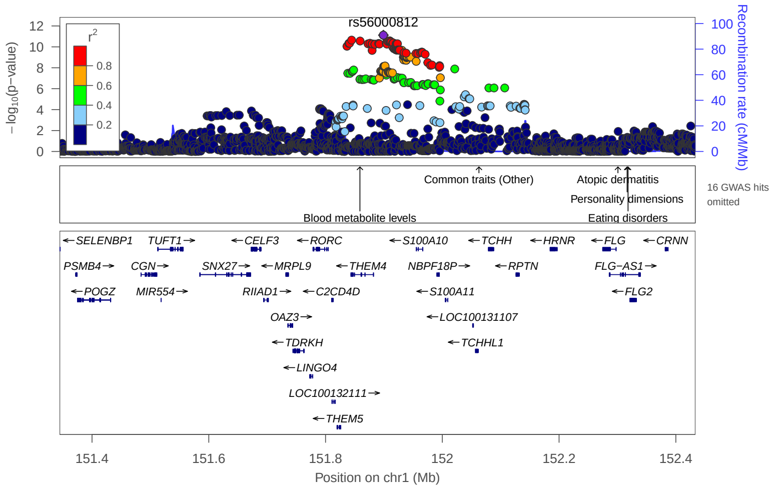

X − 24798

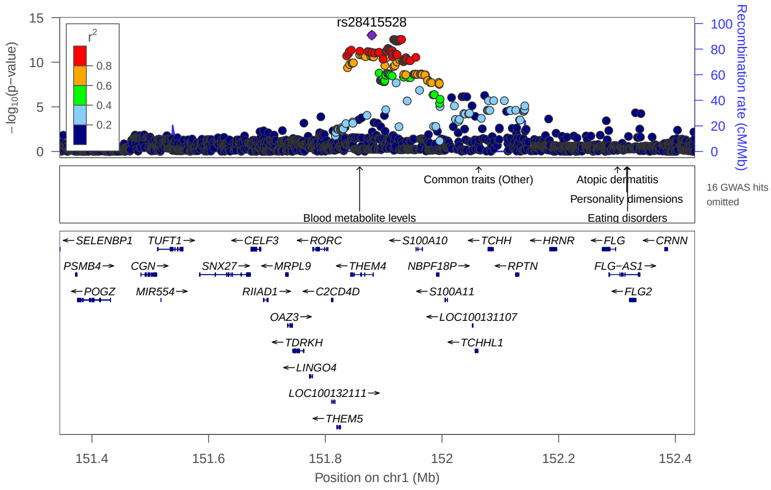

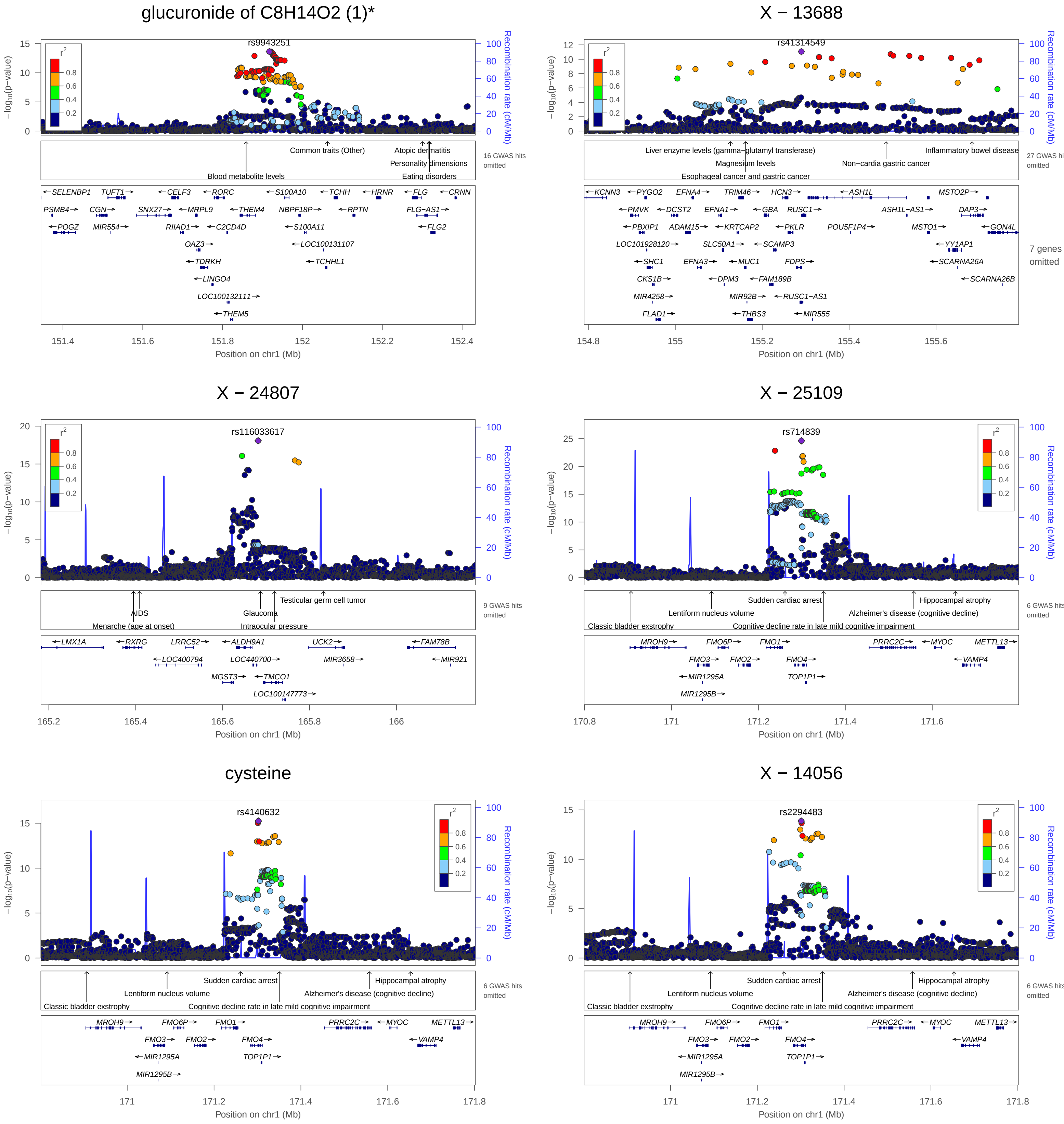
glucuronide of C8H14O2 (1)*
rs9943251
Common traits (Other)
Atopic dermatitis
Personality dimensions
Blood metabolite levels
Eating disorders
16 GWAS hits omitted
Position on chr1 (Mb)
X − 13688
rs41314549
Liver enzyme levels (gamma−glutamyl transferase)
Inflammatory bowel disease
Magnesium levels
Non−cardia gastric cancer
Esophageal cancer and gastric cancer
27 GWAS hits omitted
7 genes omitted
Position on chr1 (Mb)
X − 24807
rs116033617
Testicular germ cell tumor
AIDS
Glaucoma
Menarche (age at onset)
Intraocular pressure
9 GWAS hits omitted
Position on chr1 (Mb)
X − 25109
rs714839
Sudden cardiac arrest
Hippocampal atrophy
Lentiform nucleus volume
Alzheimer's disease (cognitive decline)
Classic bladder exstrophy
Cognitive decline rate in late mild cognitive impairment
6 GWAS hits omitted
Position on chr1 (Mb)
cysteine
rs4140632
Sudden cardiac arrest
Hippocampal atrophy
Lentiform nucleus volume
Alzheimer's disease (cognitive decline)
Classic bladder exstrophy
Cognitive decline rate in late mild cognitive impairment
6 GWAS hits omitted
Position on chr1 (Mb)
X − 14056
rs2294483
Sudden cardiac arrest
Hippocampal atrophy
Lentiform nucleus volume
Alzheimer's disease (cognitive decline)
Classic bladder exstrophy
Cognitive decline rate in late mild cognitive impairment
6 GWAS hits omitted
Position on chr1 (Mb)
−log10(p−value)
Recombination rate (cM/Mb)

N−acetylmethionine sulfoxide

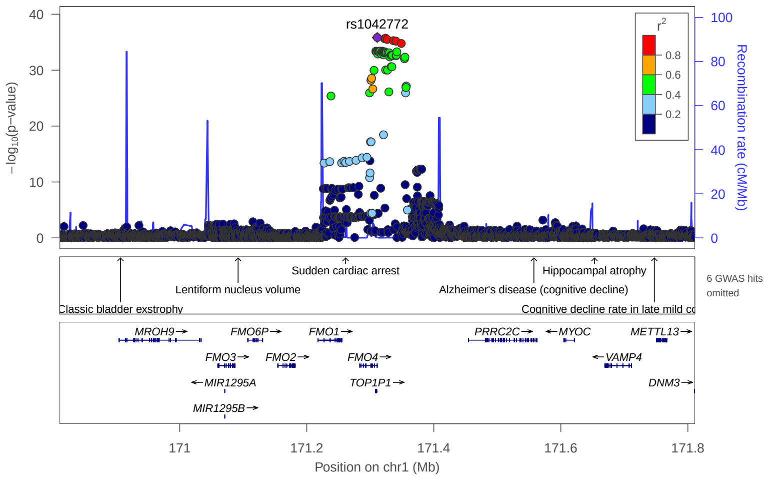

cysteinylglycine

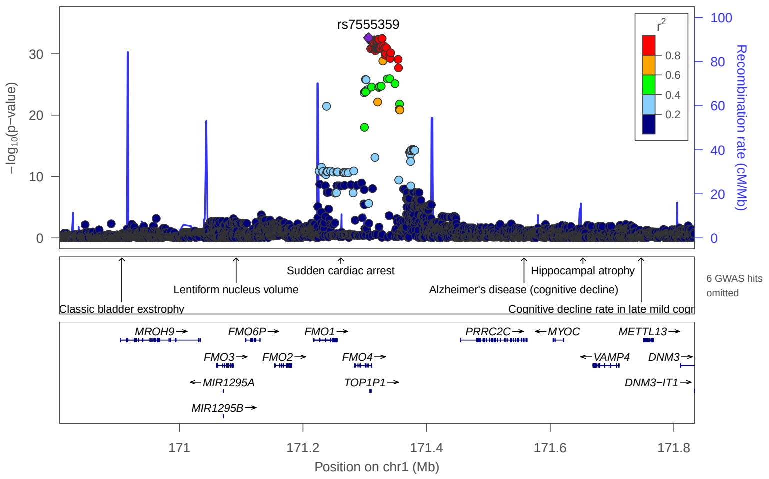

S−(3−hydroxypropyl)mercapturic acid (HPMA)

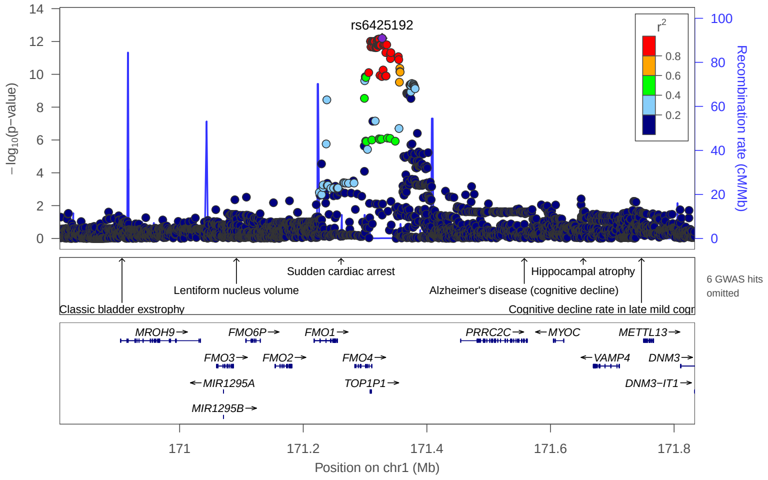

X − 21842

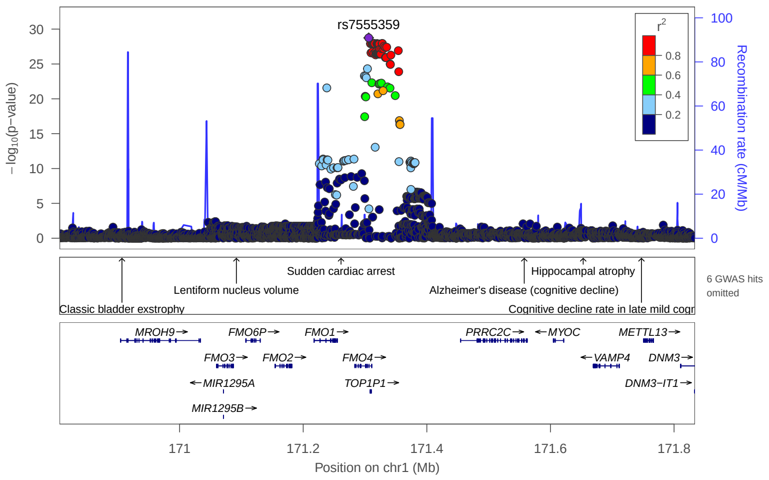

X − 24403

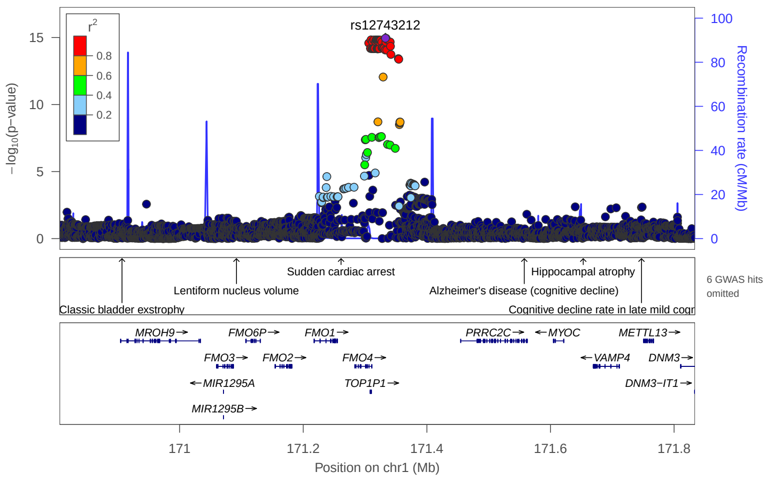

X − 24414

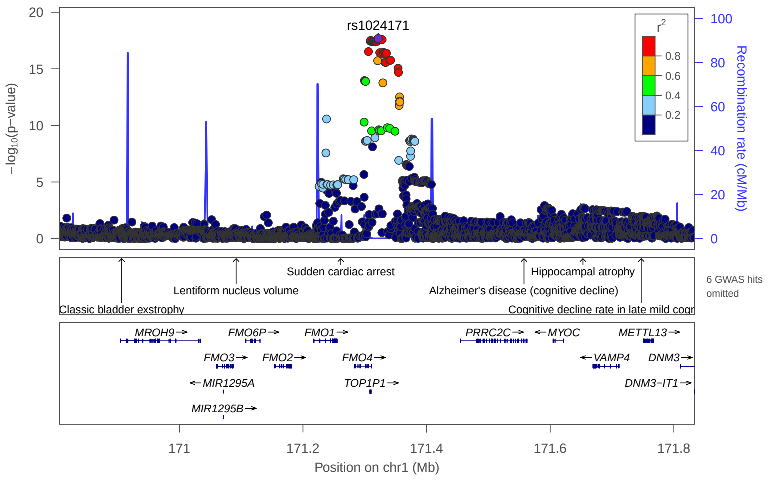

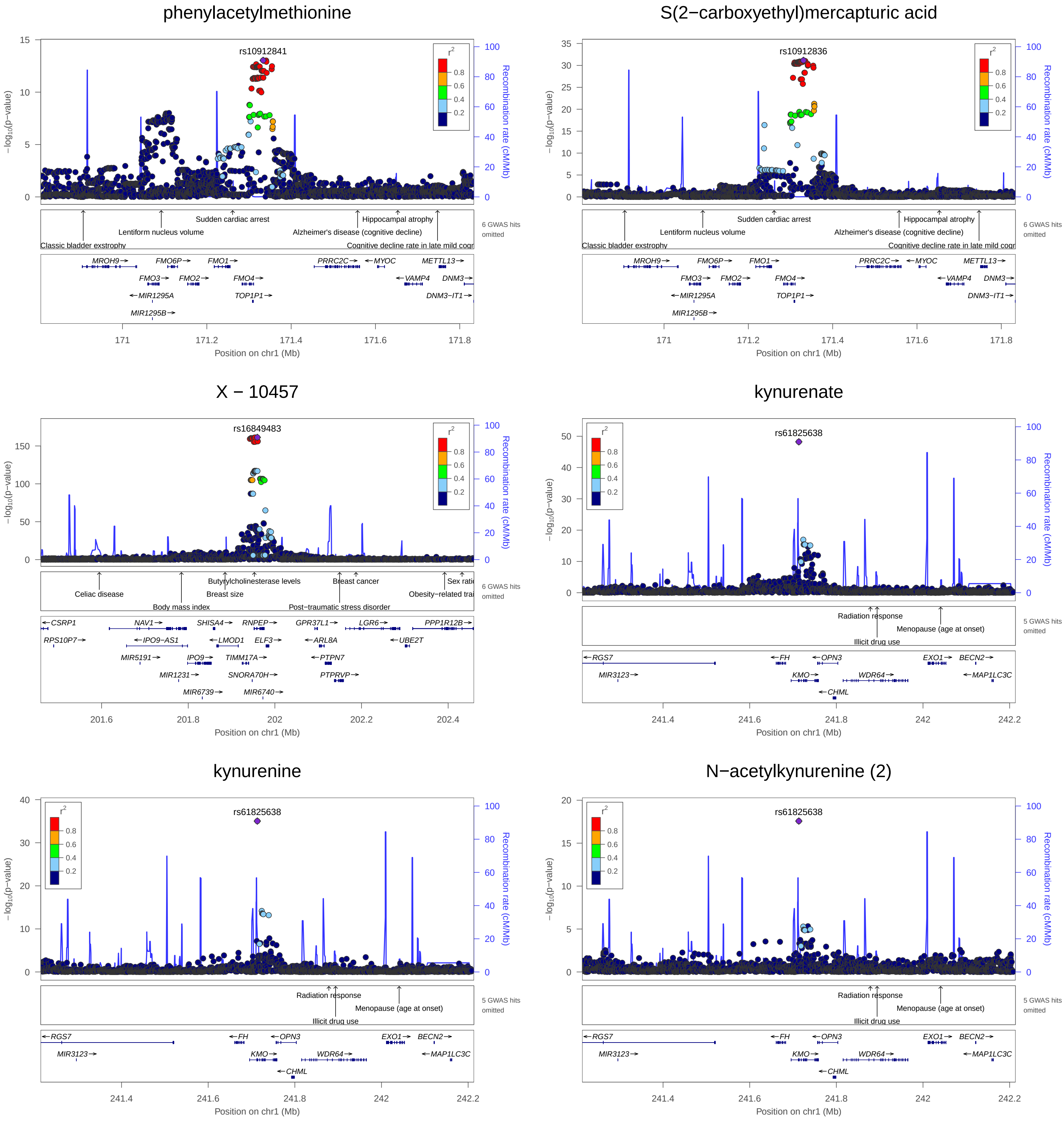

phenylacetylmethionine
rs10912841
S(2−carboxyethyl)mercapturic acid
rs10912836
X − 10457
rs16849483
kynurenate
rs61825638
kynurenine
rs61825638
N−acetylkynurenine (2)
rs61825638
−log10(p−value)
Recombination rate (cM/Mb)
r2
0.8
0.6
0.4
0.2
Sudden cardiac arrest
Hippocampal atrophy
Lentiform nucleus volume
Alzheimer's disease (cognitive decline)
Classic bladder exstrophy
Cognitive decline rate in late mild cogr
6 GWAS hits omitted
MROH9
FMO6P
FMO1
PRRC2C
MYOC
METTL13
FMO3
FMO2
FMO4
VAMP4
DNM3
MIR1295A
TOP1P1
DNM3−IT1
MIR1295B
Position on chr1 (Mb)
Butyrylcholinesterase levels
Breast cancer
Sex ratio
Celiac disease
Breast size
Obesity−related trai
Body mass index
Post−traumatic stress disorder
CSRP1
NAV1
SHISA4
RNPEP
GPR37L1
LGR6
PPP1R12B
RPS10P7
IPO9−AS1
LMOD1
ELF3
ARL8A
UBE2T
MIR5191
IPO9
TIMM17A
PTPN7
MIR1231
SNORA70H
PTPRVP
MIR6739
MIR6740
Radiation response
Menopause (age at onset)
Illicit drug use
5 GWAS hits omitted
RGS7
FH
OPN3
EXO1
BECN2
MIR3123
KMO
WDR64
MAP1LC3C
CHML

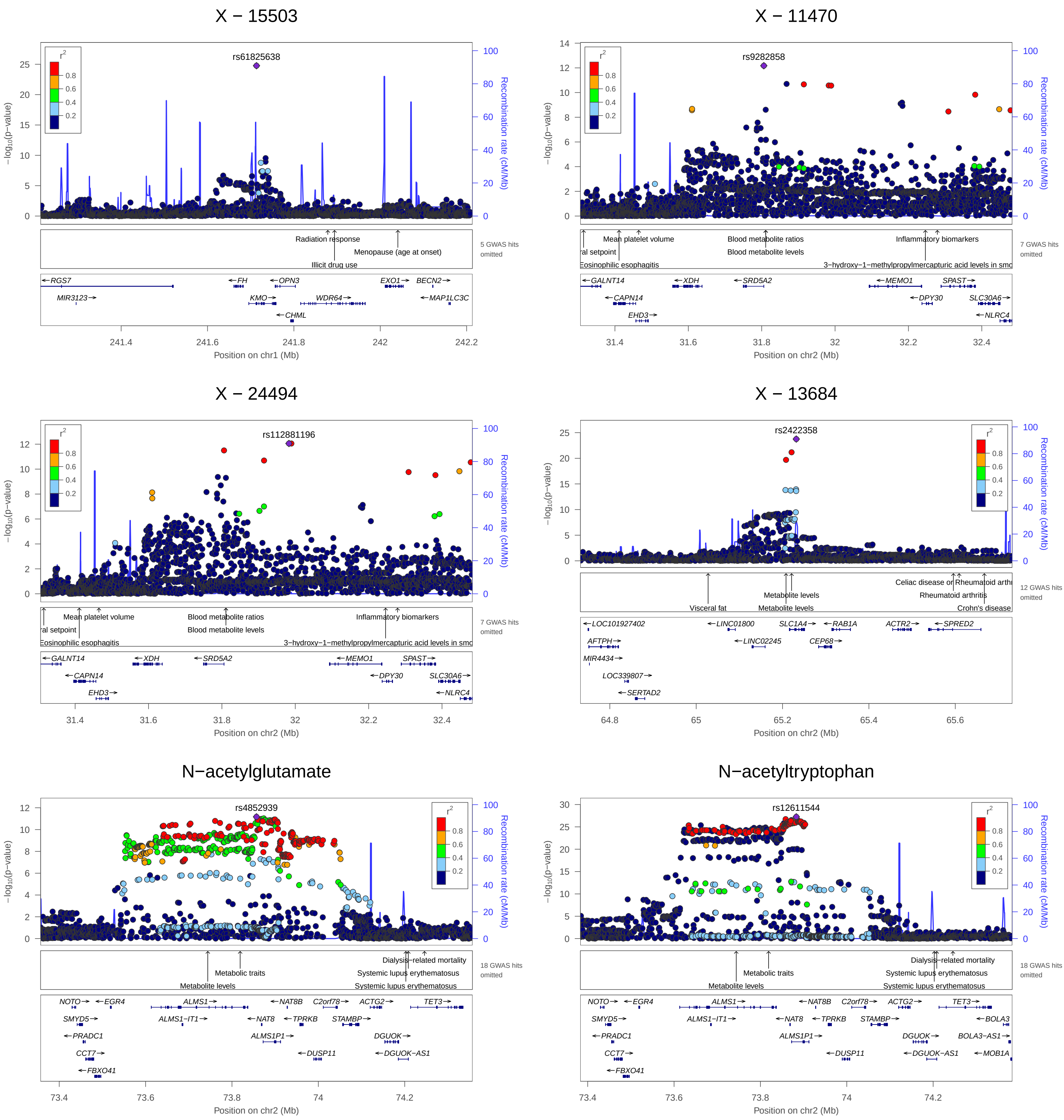
X − 15503
rs61825638
−log10(p−value)
Recombination rate (cM/Mb)
Radiation response
Menopause (age at onset)
Illicit drug use
5 GWAS hits omitted
RGS7
MIR3123
FH
KMO
OPN3
CHML
WDR64
EXO1
BECN2
MAP1LC3C
Position on chr1 (Mb)
X − 11470
rs9282858
Mean platelet volume
Blood metabolite ratios
Blood metabolite levels
Inflammatory biomarkers
Eosinophilic esophagitis
3−hydroxy−1−methylpropylmercapturic acid levels in smo
7 GWAS hits omitted
GALNT14
CAPN14
EHD3
XDH
SRD5A2
MEMO1
DPY30
SPAST
SLC30A6
NLRC4
Position on chr2 (Mb)
X − 24494
rs112881196
Mean platelet volume
Blood metabolite ratios
Blood metabolite levels
Inflammatory biomarkers
Eosinophilic esophagitis
3−hydroxy−1−methylpropylmercapturic acid levels in smo
7 GWAS hits omitted
GALNT14
CAPN14
EHD3
XDH
SRD5A2
MEMO1
DPY30
SPAST
SLC30A6
NLRC4
Position on chr2 (Mb)
X − 13684
rs2422358
Celiac disease or Rheumatoid arthri
Metabolite levels
Rheumatoid arthritis
Visceral fat
Crohn's disease
12 GWAS hits omitted
LOC101927402
AFTPH
MIR4434
LOC339807
SERTAD2
LINC01800
LINC02245
SLC1A4
CEP68
RAB1A
ACTR2
SPRED2
Position on chr2 (Mb)
N−acetylglutamate
rs4852939
Dialysis−related mortality
Metabolic traits
Systemic lupus erythematosus
Metabolite levels
18 GWAS hits omitted
NOTO
SMYD5
PRADC1
CCT7
FBXO41
EGR4
ALMS1
ALMS1−IT1
NAT8
ALMS1P1
TPRKB
DUSP11
C2orf78
STAMBP
ACTG2
DGUOK
DGUOK−AS1
TET3
Position on chr2 (Mb)
N−acetyltryptophan
rs12611544
Dialysis−related mortality
Metabolic traits
Systemic lupus erythematosus
Metabolite levels
18 GWAS hits omitted
NOTO
SMYD5
PRADC1
CCT7
FBXO41
EGR4
ALMS1
ALMS1−IT1
NAT8
ALMS1P1
TPRKB
DUSP11
C2orf78
STAMBP
ACTG2
DGUOK
DGUOK−AS1
TET3
BOLA3
BOLA3−AS1
MOB1A
Position on chr2 (Mb)

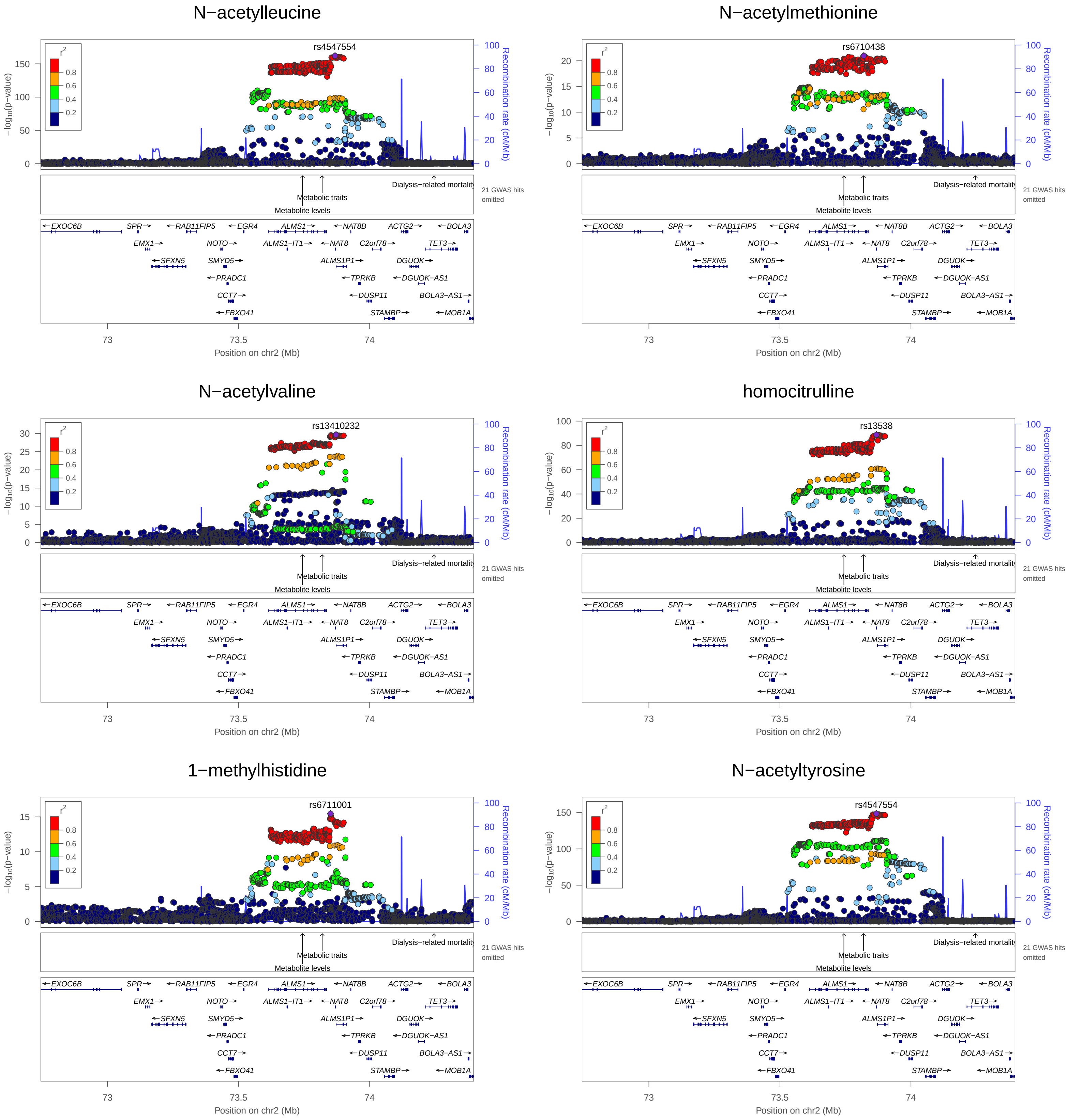
N−acetylleucine
rs4547554
N−acetylmethionine
rs6710438
N−acetylvaline
rs13410232
homocitrulline
rs13538
1−methylhistidine
rs6711001
N−acetyltyrosine
rs4547554
r2
0.8
0.6
0.4
0.2
−log10(p−value)
Recombination rate (cM/Mb)
Dialysis−related mortality
Metabolic traits
Metabolite levels
21 GWAS hits omitted
EXOC6B
SPR
RAB11FIP5
EGR4
ALMS1
NAT8B
ACTG2
BOLA3
EMX1
NOTO
ALMS1−IT1
NAT8
C2orf78
TET3
SFXN5
SMYD5
ALMS1P1
DGUOK
PRADC1
TPRKB
DGUOK−AS1
CCT7
DUSP11
BOLA3−AS1
FBXO41
STAMBP
MOB1A
73
73.5
74
Position on chr2 (Mb)

N−alpha−acetylornithine

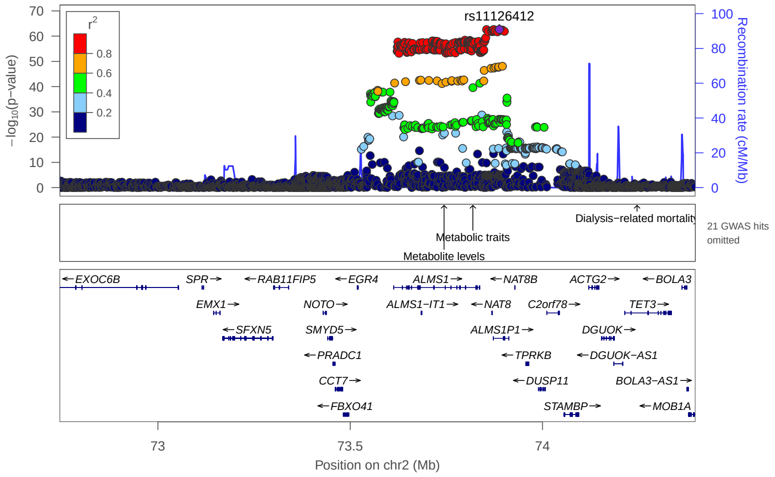

N−acetylasparagine

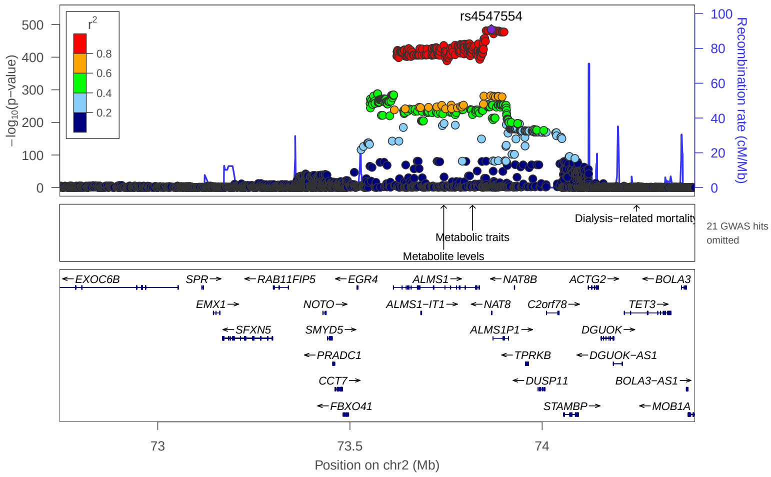

N−acetylglutamine

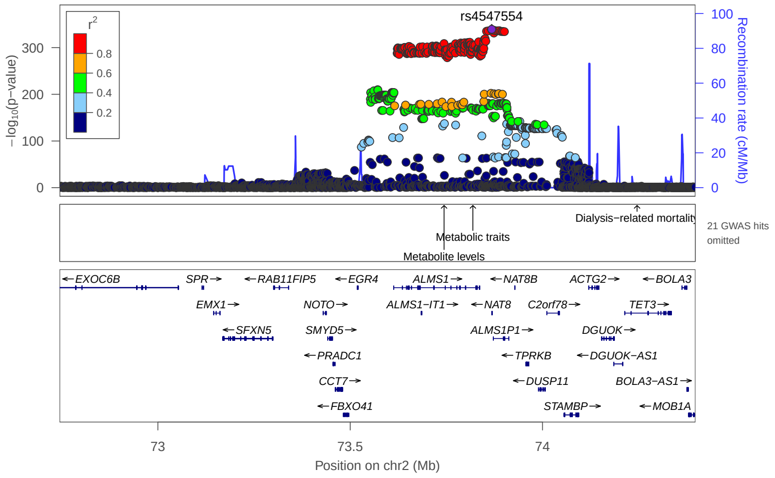

N−acetylhistidine

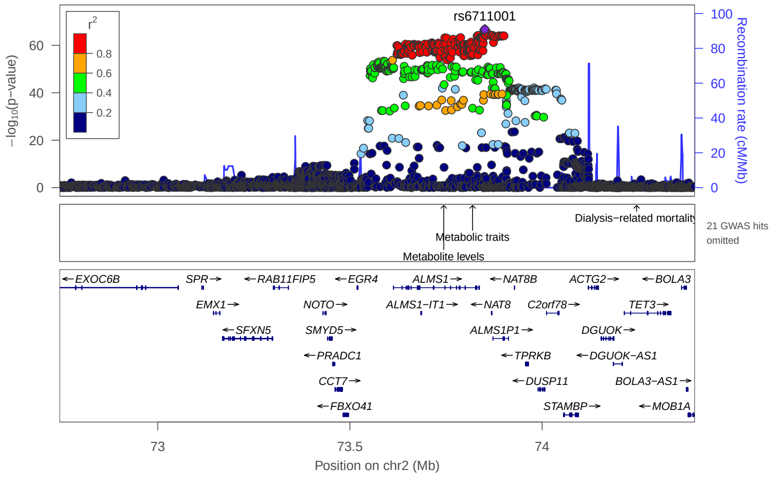

N−acetylphenylalanine

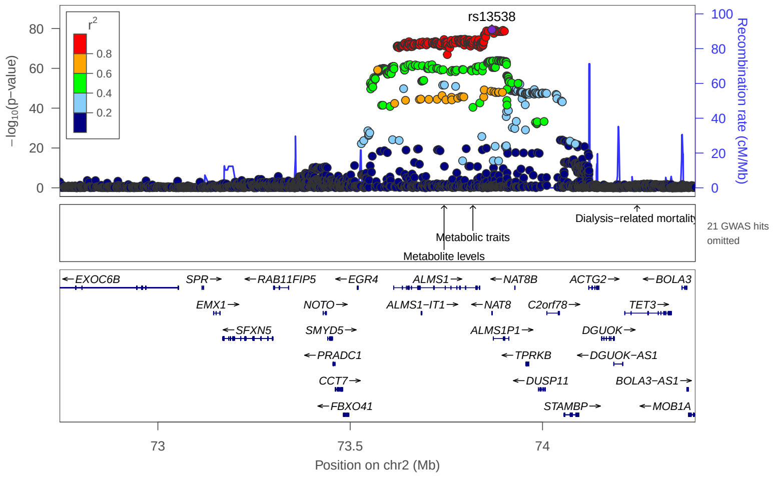

N−acetylarginine

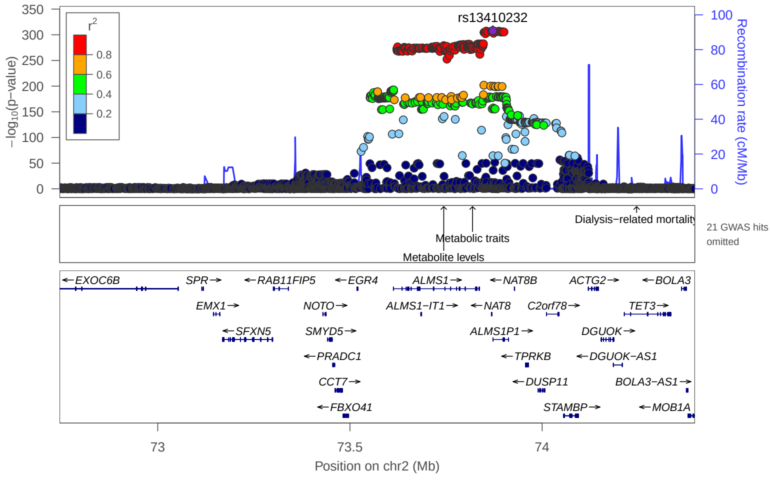

N2,N2−dimethylguanosine

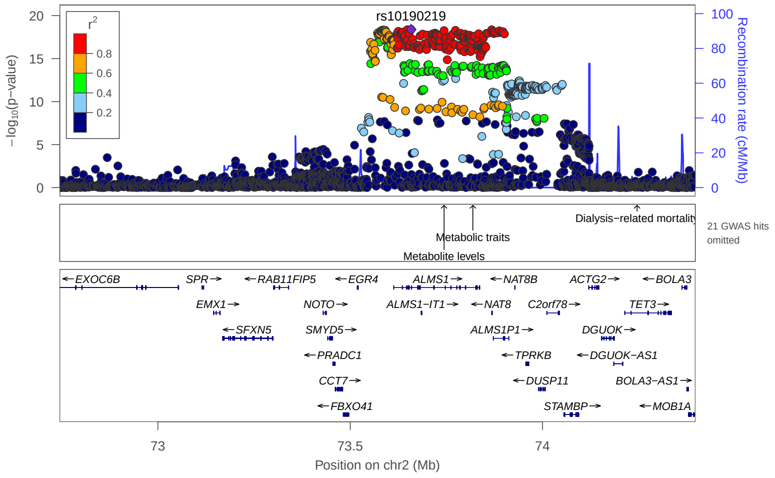

N2−acetyllysine

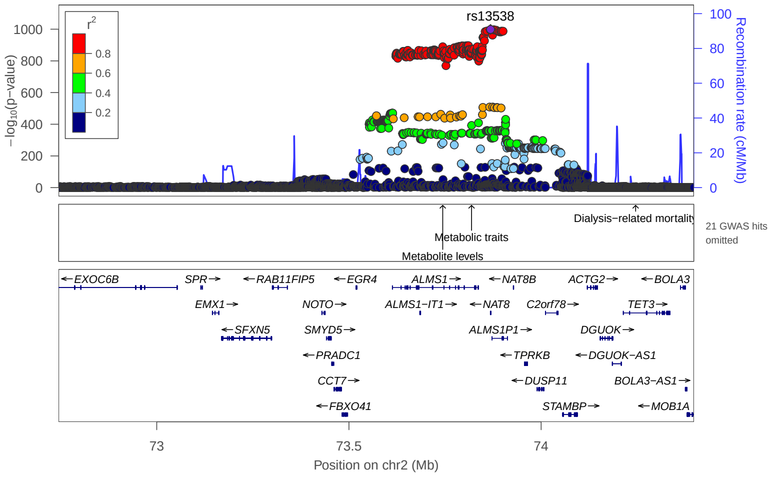

N6−acetyllysine

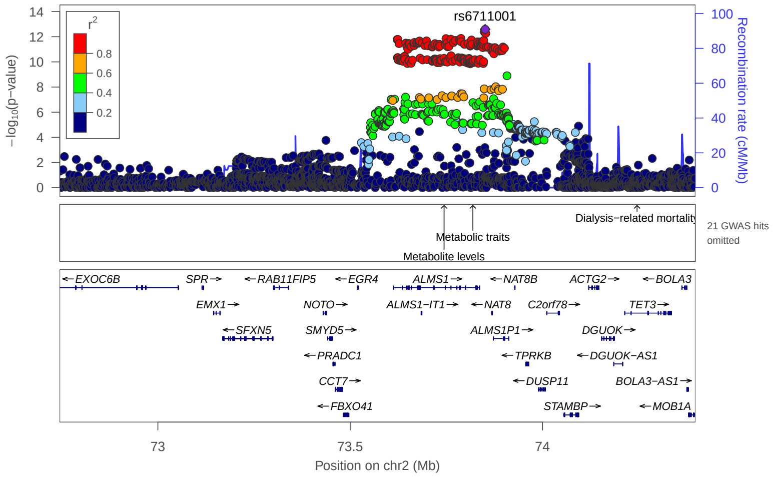

N6−carboxyethyllysine

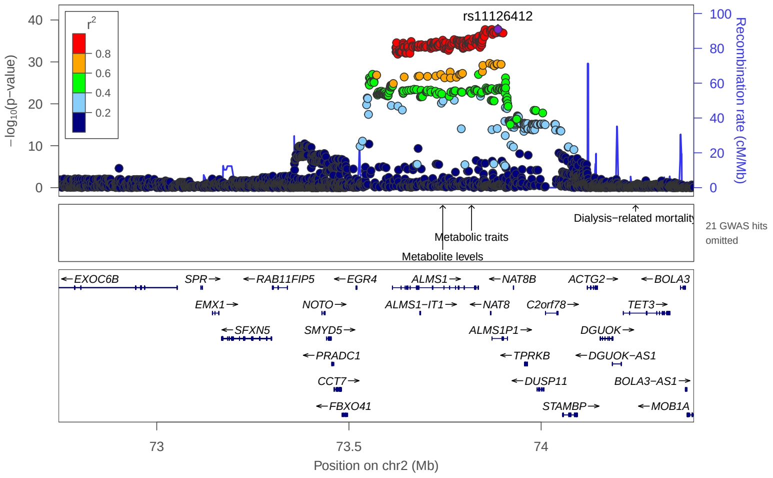

N−delta−acetylornithine

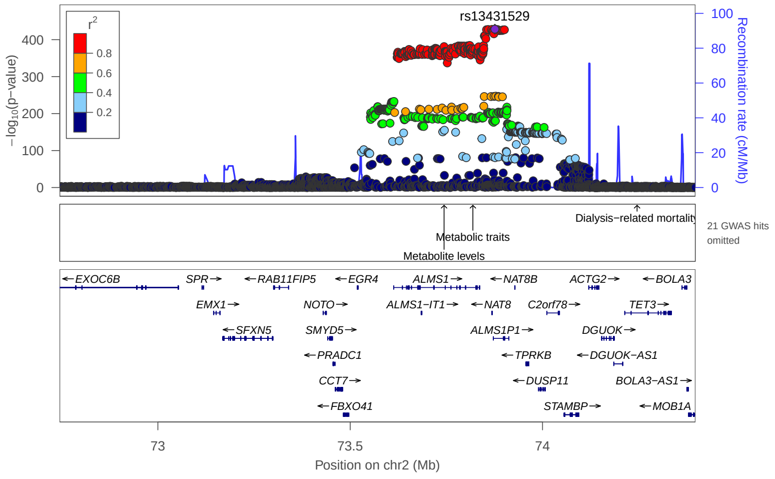

N−acetyl−1−methylhistidine*

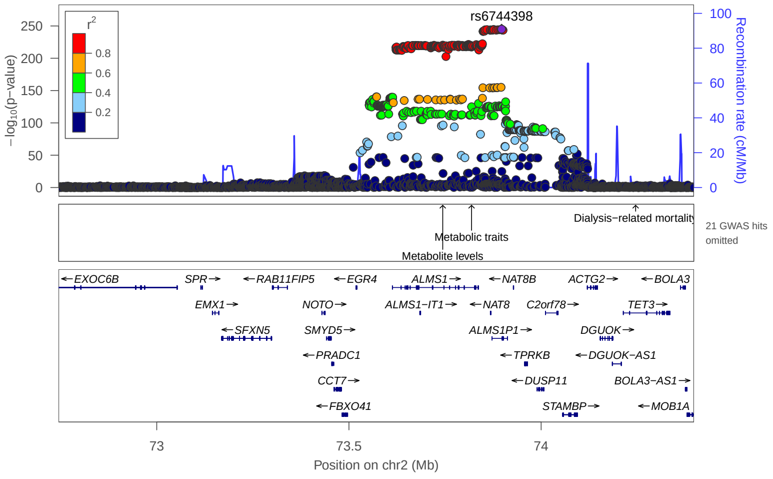

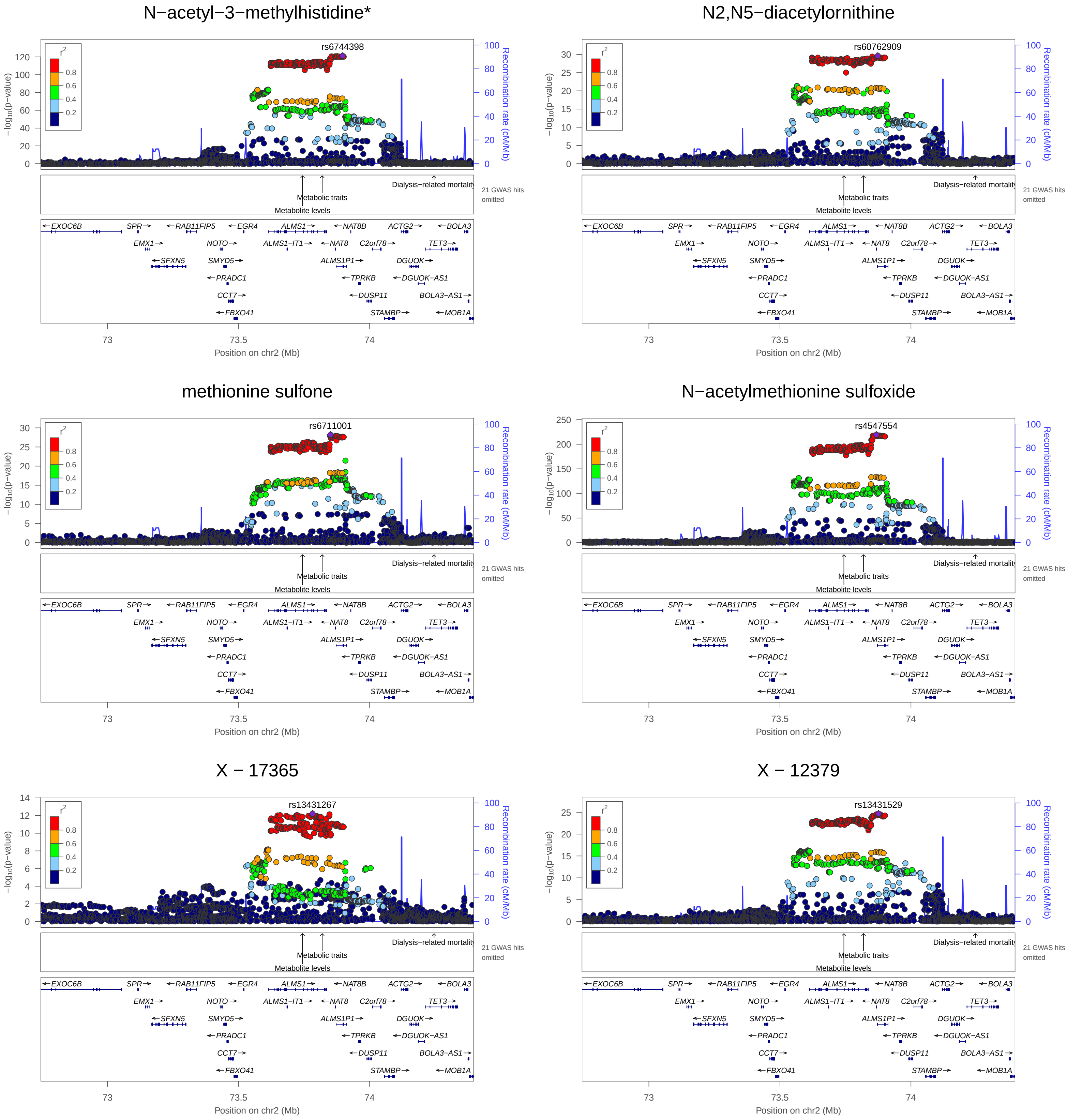

N−acetyl−3−methylhistidine*
rs6744398
N2,N5−diacetylornithine
rs60762909
methionine sulfone
rs6711001
N−acetylmethionine sulfoxide
rs4547554
X − 17365
rs13431267
X − 12379
rs13431529
−log10(p−value)
Recombination rate (cM/Mb)
r2
0.8
0.6
0.4
0.2
Dialysis−related mortality
Metabolic traits
Metabolite levels
21 GWAS hits omitted
EXOC6B
SPR
RAB11FIP5
EGR4
ALMS1
NAT8B
ACTG2
BOLA3
EMX1
NOTO
ALMS1−IT1
NAT8
C2orf78
TET3
SFXN5
SMYD5
ALMS1P1
DGUOK
PRADC1
TPRKB
DGUOK−AS1
CCT7
DUSP11
BOLA3−AS1
FBXO41
STAMBP
MOB1A
73
73.5
74
Position on chr2 (Mb)

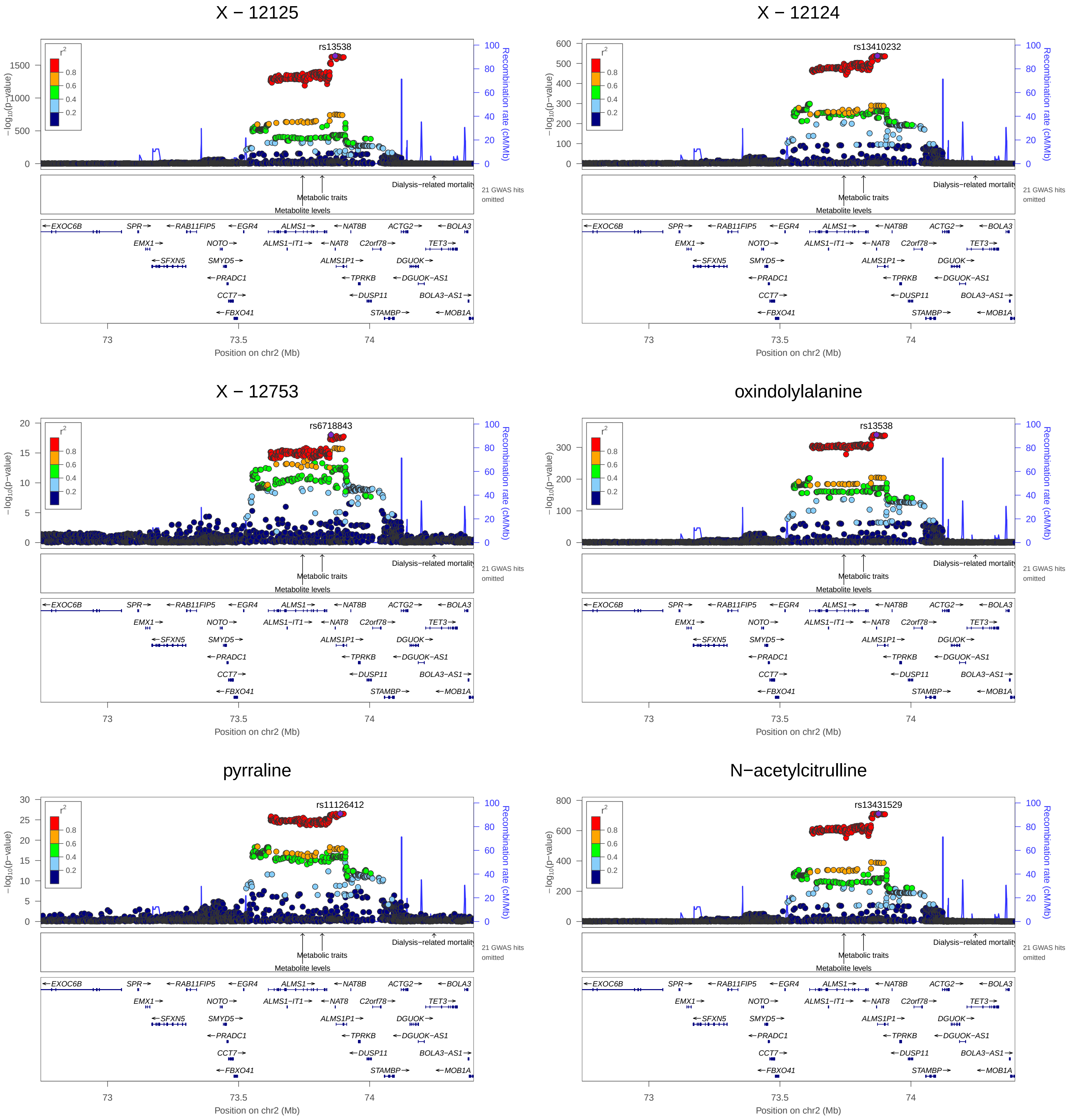

X − 12125
rs13538
X − 12124
rs13410232
X − 12753
rs6718843
oxindolylalanine
rs13538
pyrraline
rs11126412
N−acetylcitrulline
rs13431529
−log10(p−value)
Recombination rate (cM/Mb)
r2
0.8
0.6
0.4
0.2
Dialysis−related mortality
Metabolic traits
Metabolite levels
21 GWAS hits omitted
EXOC6B
SPR
RAB11FIP5
EGR4
ALMS1
NAT8B
ACTG2
BOLA3
EMX1
NOTO
ALMS1−IT1
NAT8
C2orf78
TET3
SFXN5
SMYD5
ALMS1P1
DGUOK
PRADC1
TPRKB
DGUOK−AS1
CCT7
DUSP11
BOLA3−AS1
FBXO41
STAMBP
MOB1A
73
73.5
74
Position on chr2 (Mb)

N−acetylpyrraline

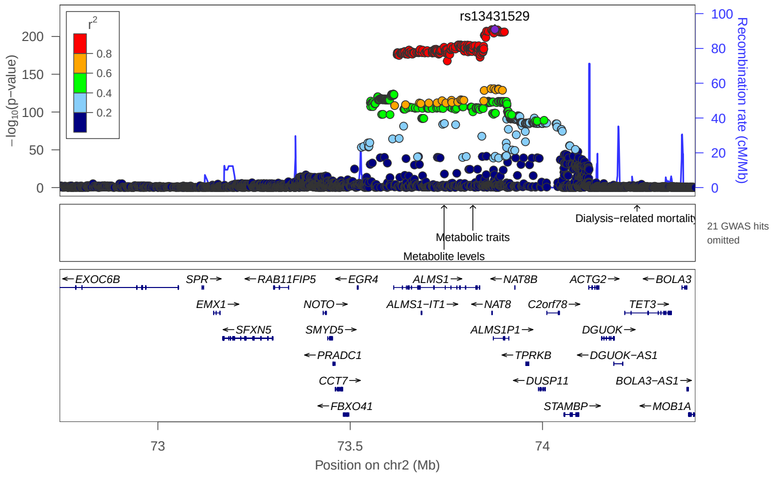

N2,N6−diacetyllysine

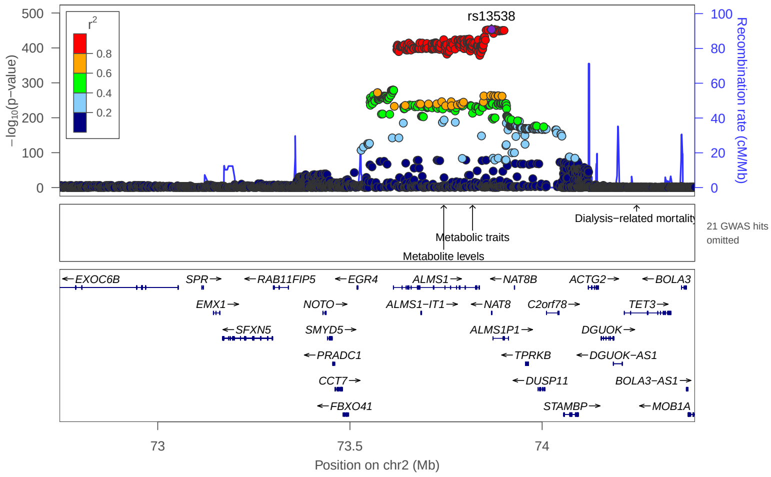

N−acetylkynurenine (2)

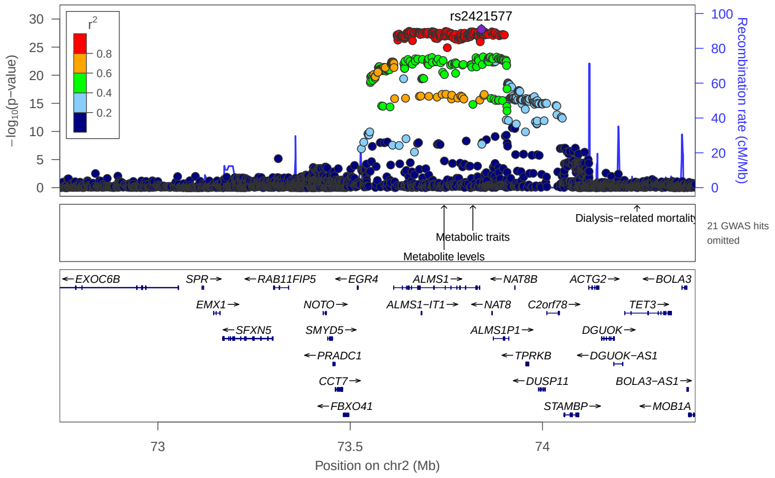

X − 23423

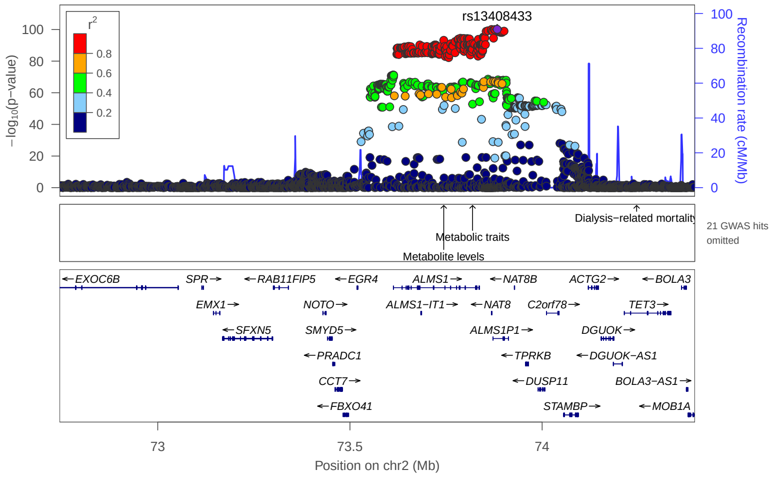

N−acetyl−L−2−aminoadipic acid

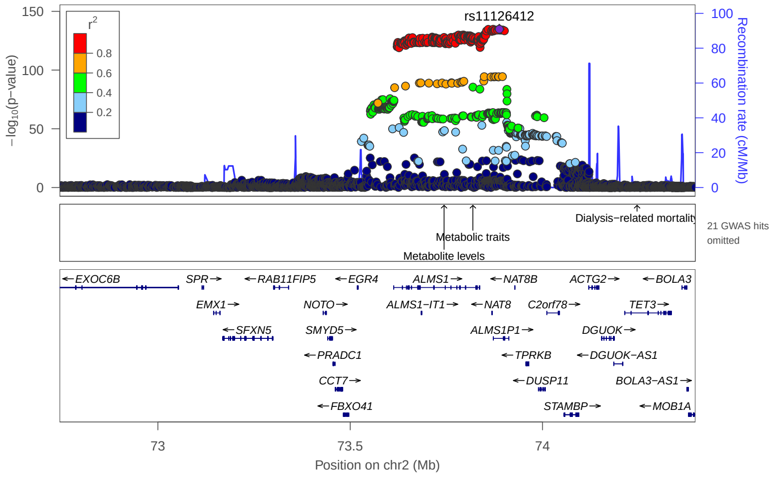

X − 13698

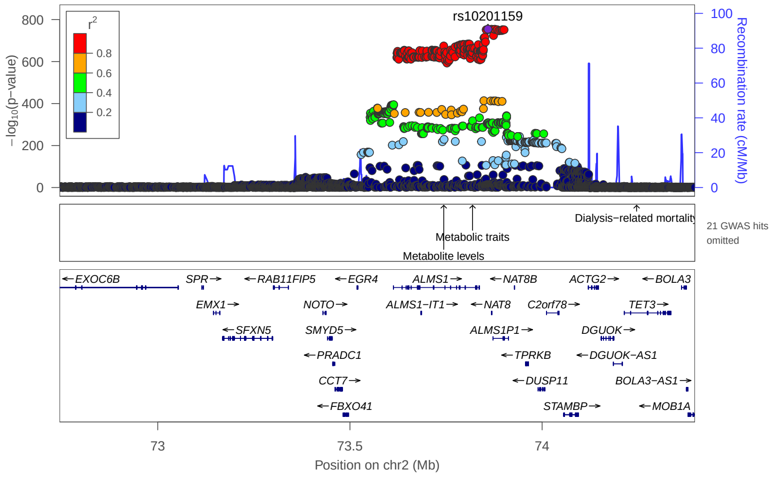

X − 24519

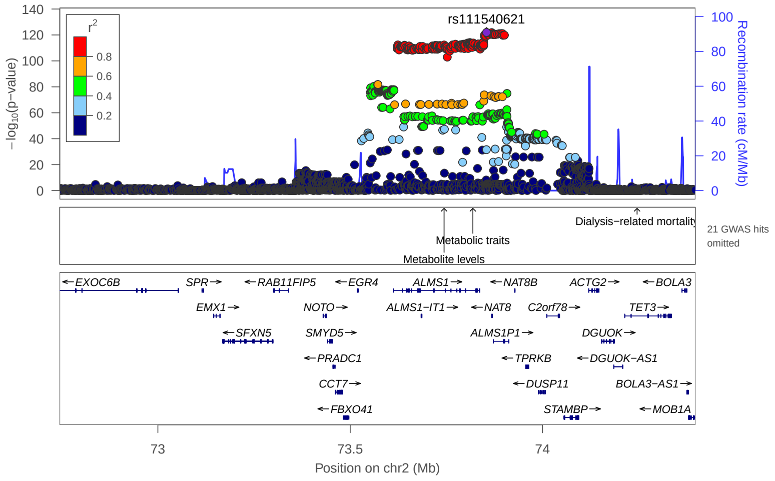

N2−acetyl,N6,N6−dimethyllysine

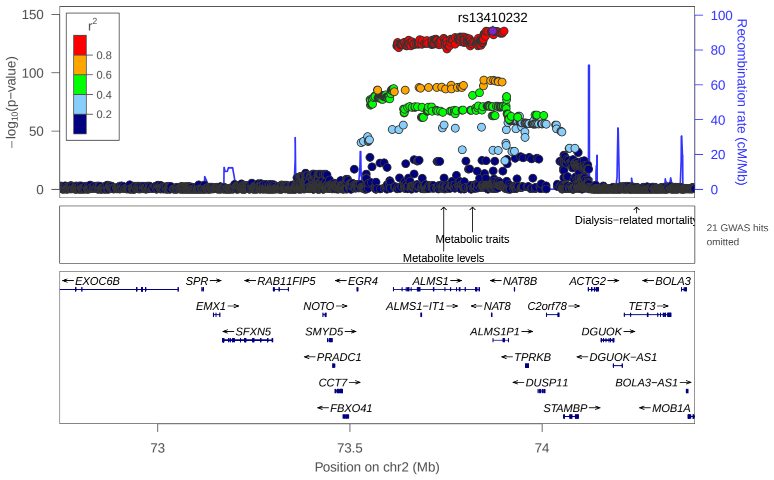

S(2−carboxyethyl)mercapturic acid

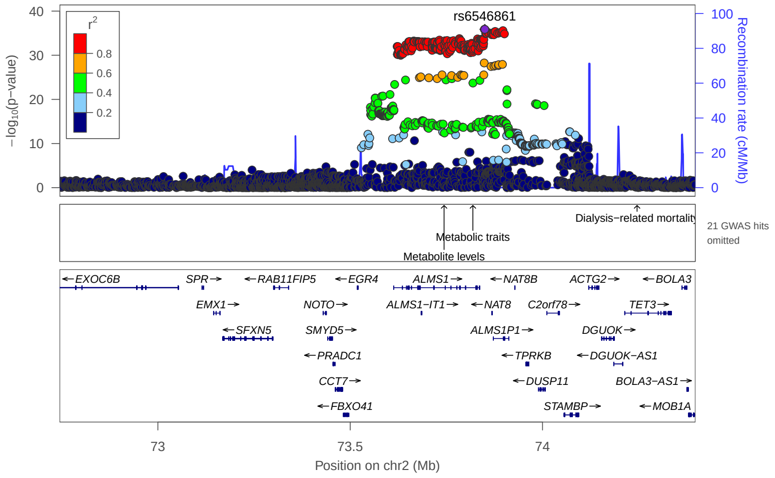

N−acetyl−2−aminooctanoate*

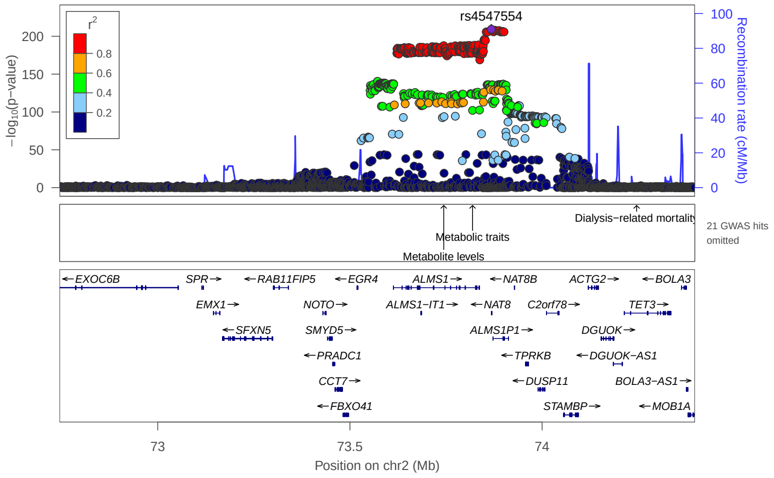

3−S−cysteinyl−2−methylpropanoate*

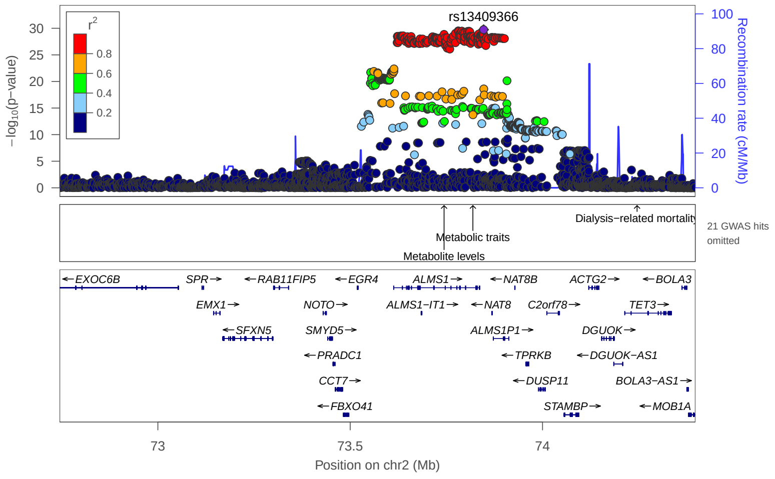

N−acetylhomocitrulline

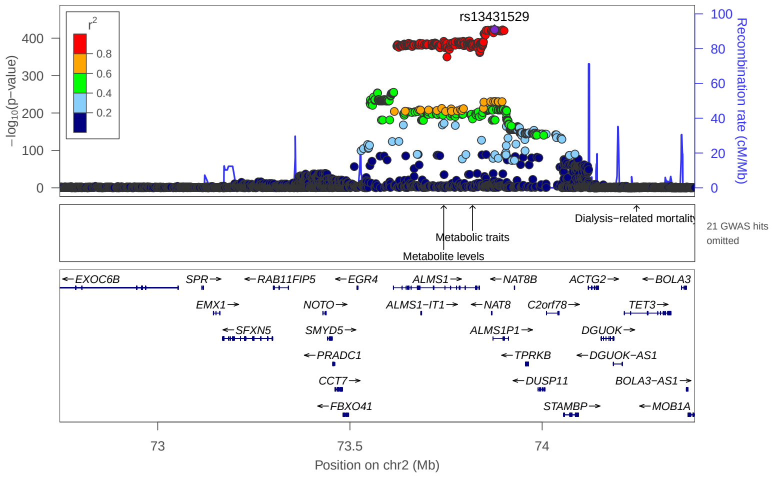

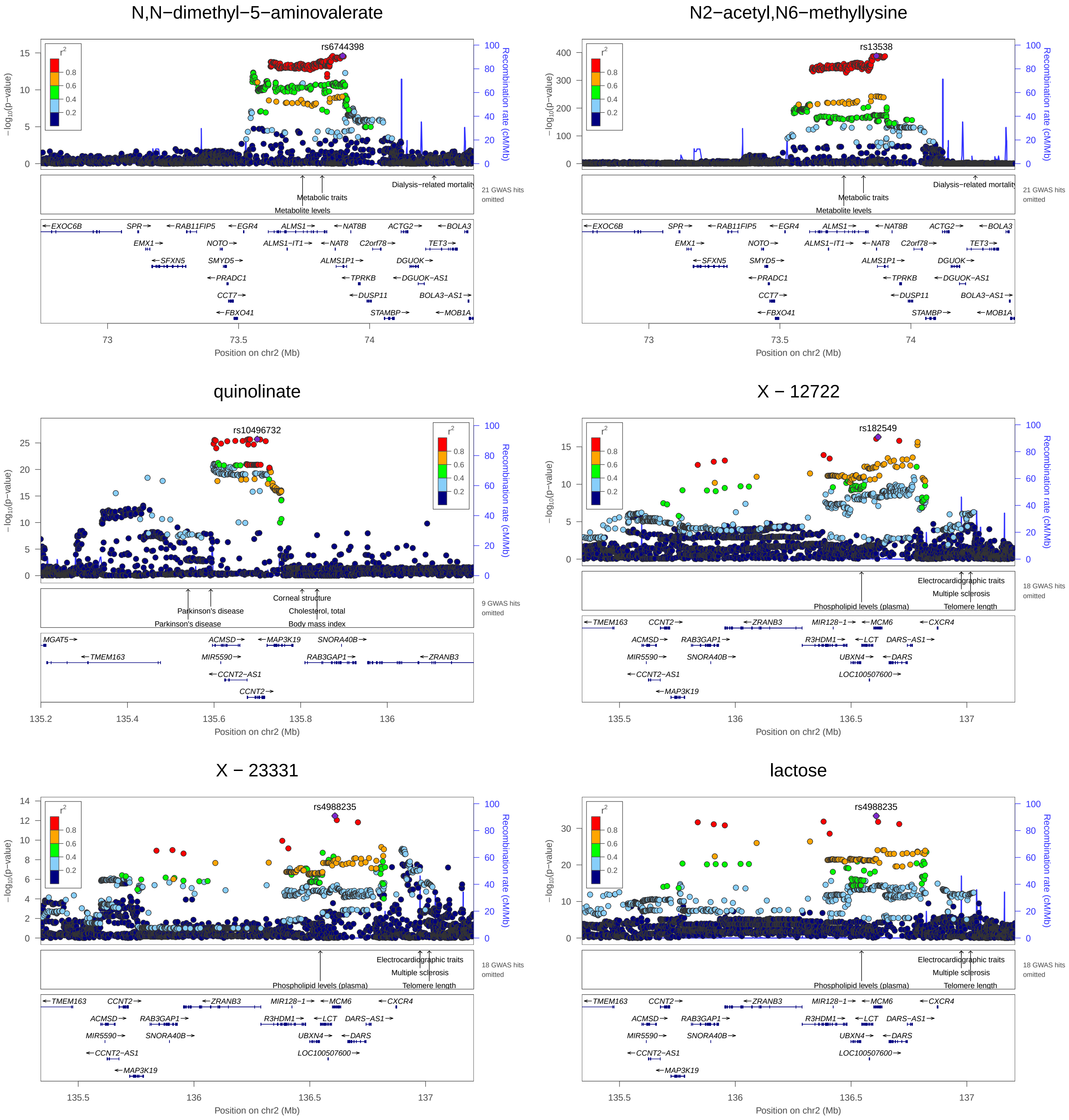
N,N−dimethyl−5−aminovalerate
rs6744398
N2−acetyl,N6−methyllysine
rs13538
Dialysis−related mortality
Metabolic traits
Metabolite levels
21 GWAS hits omitted
EXOC6B
SPR
RAB11FIP5
EGR4
ALMS1
NAT8B
ACTG2
BOLA3
EMX1
NOTO
ALMS1−IT1
NAT8
C2orf78
TET3
SFXN5
SMYD5
ALMS1P1
DGUOK
PRADC1
TPRKB
DGUOK−AS1
CCT7
DUSP11
BOLA3−AS1
FBXO41
STAMBP
MOB1A
Position on chr2 (Mb)
quinolinate
rs10496732
Parkinson's disease
Corneal structure
Cholesterol, total
Body mass index
9 GWAS hits omitted
MGAT5
TMEM163
ACMSD
MAP3K19
SNORA40B
MIR5590
RAB3GAP1
ZRANB3
CCNT2−AS1
CCNT2
X − 12722
rs182549
X − 23331
rs4988235
lactose
Electrocardiographic traits
Multiple sclerosis
Phospholipid levels (plasma)
Telomere length
18 GWAS hits omitted
MIR128−1
MCM6
CXCR4
R3HDM1
LCT
DARS−AS1
UBXN4
DARS
LOC100507600
−log10(p−value)
Recombination rate (cM/Mb)
r2

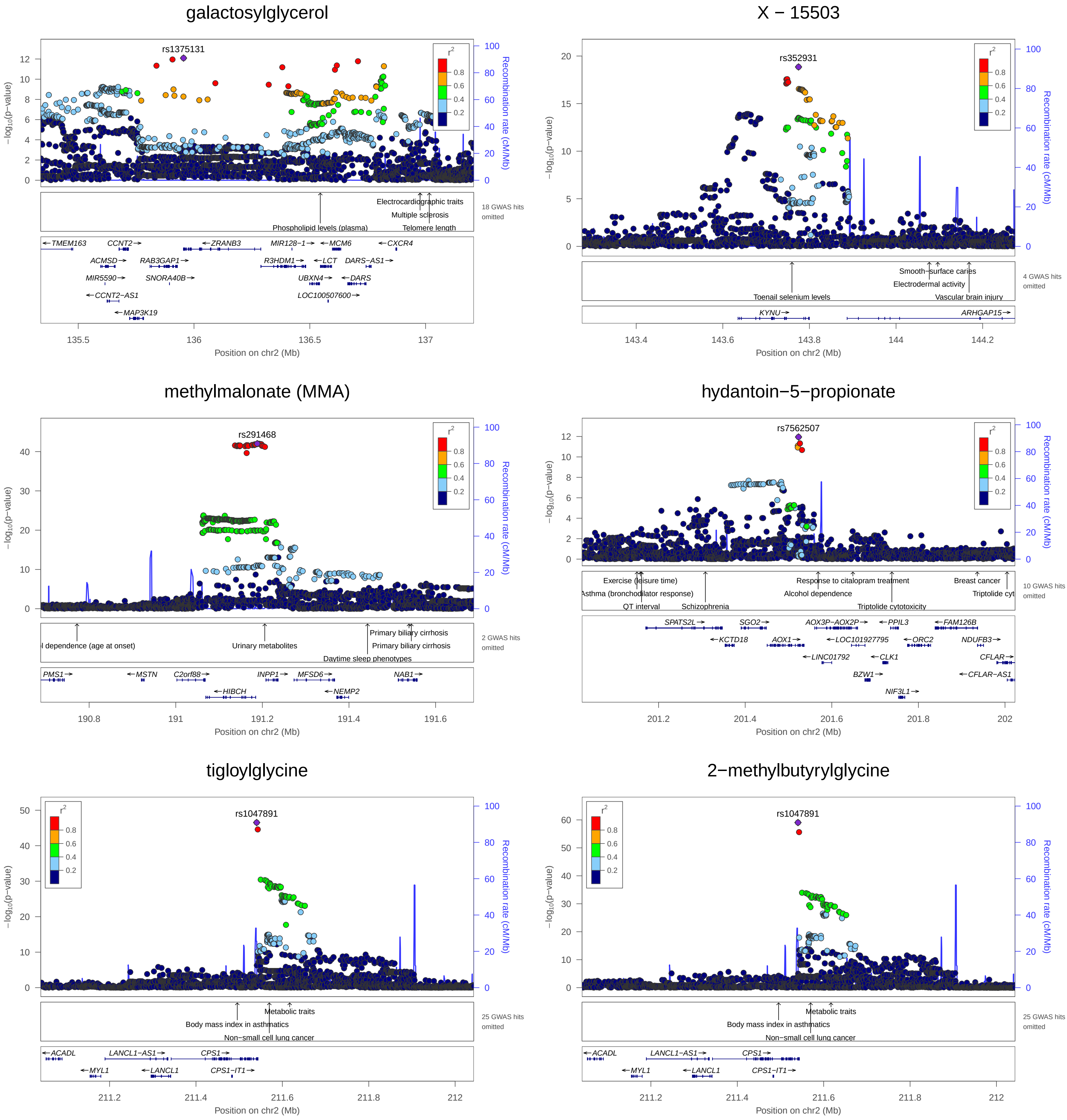

galactosylglycerol
rs1375131
−log10(p−value)
Recombination rate (cM/Mb)
Electrocardiographic traits
Multiple sclerosis
Phospholipid levels (plasma)
Telomere length
18 GWAS hits omitted
TMEM163
CCNT2
ZRANB3
MIR128−1
MCM6
CXCR4
ACMSD
RAB3GAP1
R3HDM1
LCT
DARS−AS1
MIR5590
SNORA40B
UBXN4
DARS
CCNT2−AS1
LOC100507600
MAP3K19
Position on chr2 (Mb)
X − 15503
rs352931
Smooth−surface caries
Electrodermal activity
Toenail selenium levels
Vascular brain injury
4 GWAS hits omitted
KYNU
ARHGAP15
methylmalonate (MMA)
rs291468
Primary biliary cirrhosis
Urinary metabolites
Daytime sleep phenotypes
2 GWAS hits omitted
PMS1
MSTN
C2orf88
INPP1
MFSD6
NAB1
HIBCH
NEMP2
hydantoin−5−propionate
rs7562507
Exercise (leisure time)
Asthma (bronchodilator response)
QT interval
Schizophrenia
Response to citalopram treatment
Alcohol dependence
Triptolide cytotoxicity
Breast cancer
10 GWAS hits omitted
SPATS2L
SGO2
AOX3P−AOX2P
PPIL3
FAM126B
KCTD18
AOX1
LOC101927795
ORC2
NDUFB3
LINC01792
CLK1
CFLAR
BZW1
CFLAR−AS1
NIF3L1
tigloylglycine
rs1047891
Metabolic traits
Body mass index in asthmatics
Non−small cell lung cancer
25 GWAS hits omitted
ACADL
LANCL1−AS1
CPS1
MYL1
LANCL1
CPS1−IT1
2−methylbutyrylglycine

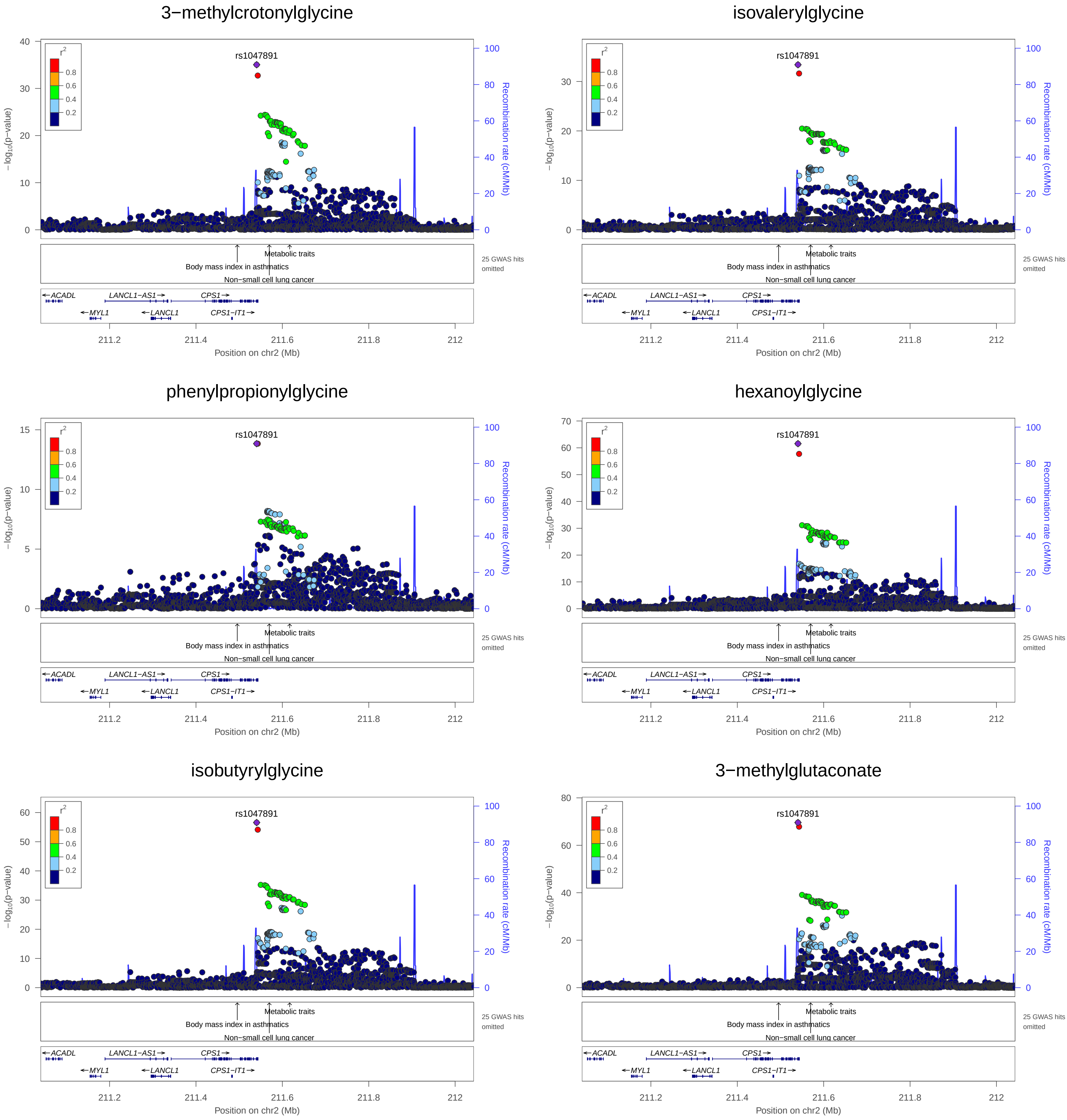
3−methylcrotonylglycine
isovalerylglycine
phenylpropionylglycine
hexanoylglycine
isobutyrylglycine
3−methylglutaconate
rs1047891
r2
0.8
0.6
0.4
0.2
−log10(p−value)
Recombination rate (cM/Mb)
Metabolic traits
Body mass index in asthmatics
Non−small cell lung cancer
25 GWAS hits omitted
ACADL
LANCL1−AS1
CPS1
MYL1
LANCL1
CPS1−IT1
211.2
211.4
211.6
211.8
212
Position on chr2 (Mb)

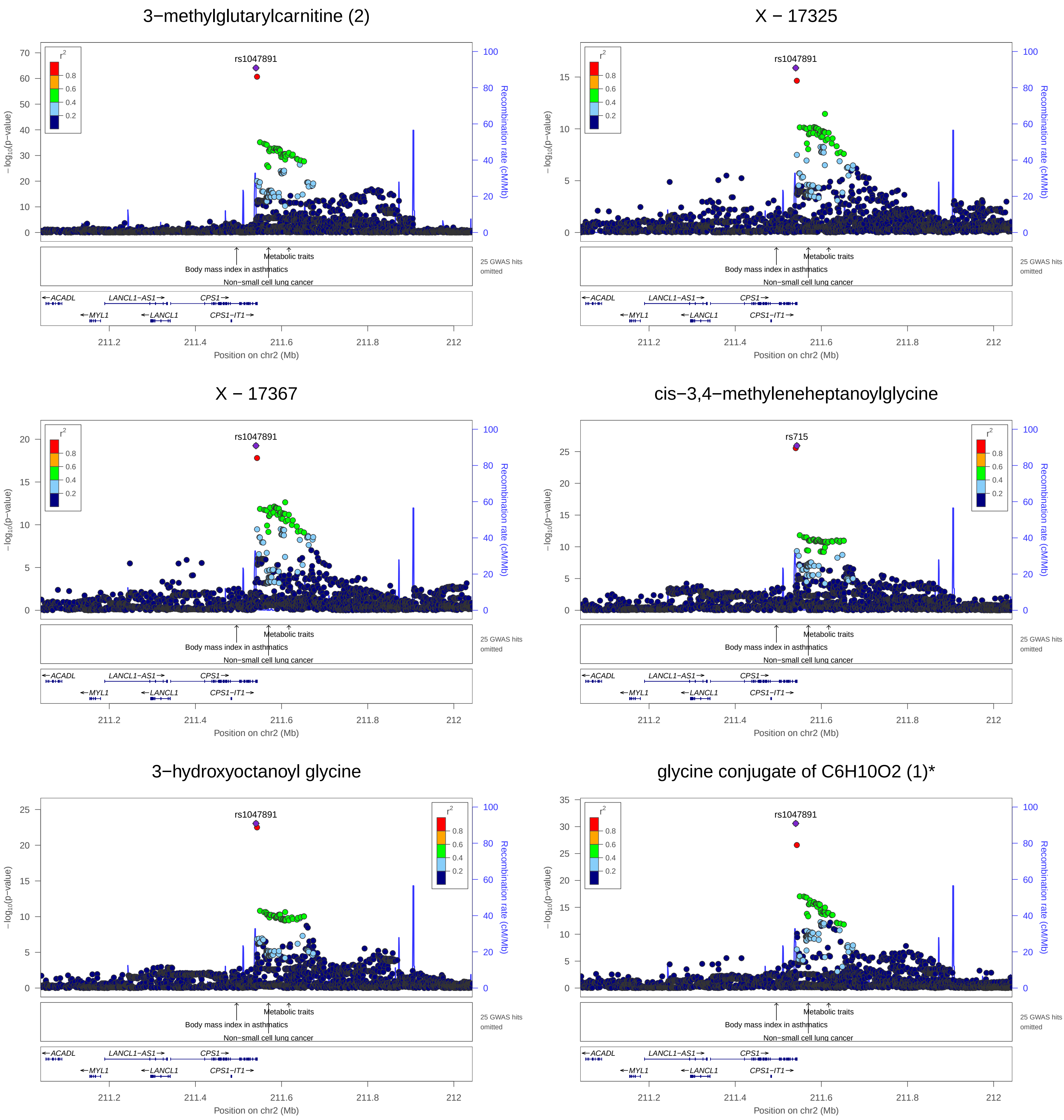
3−methylglutarylcarnitine (2)
X − 17325
X − 17367
cis−3,4−methyleneheptanoylglycine
3−hydroxyoctanoyl glycine
glycine conjugate of C6H10O2 (1)*
rs1047891
rs715
r^2
0.8
0.6
0.4
0.2
$-\log_{10}$(p−value)
Recombination rate (cM/Mb)
Metabolic traits
Body mass index in asthmatics
Non−small cell lung cancer
25 GWAS hits omitted
ACADL
LANCL1−AS1
CPS1
MYL1
LANCL1
CPS1−IT1
211.2
211.4
211.6
211.8
212
Position on chr2 (Mb)

X − 24406

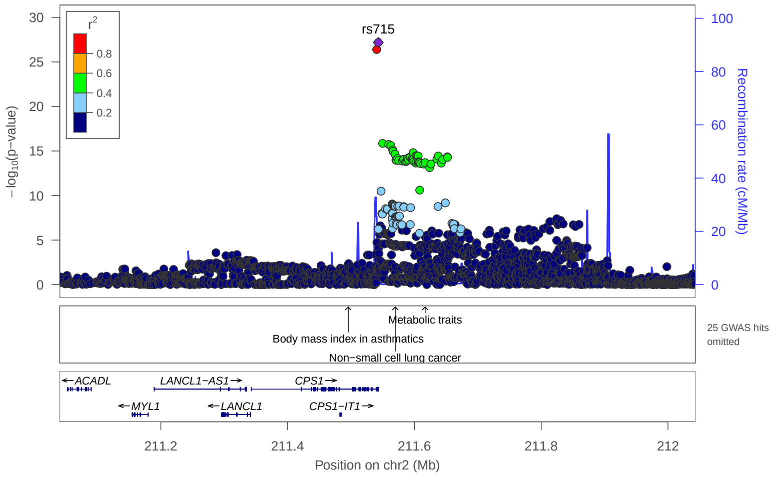

X − 24465

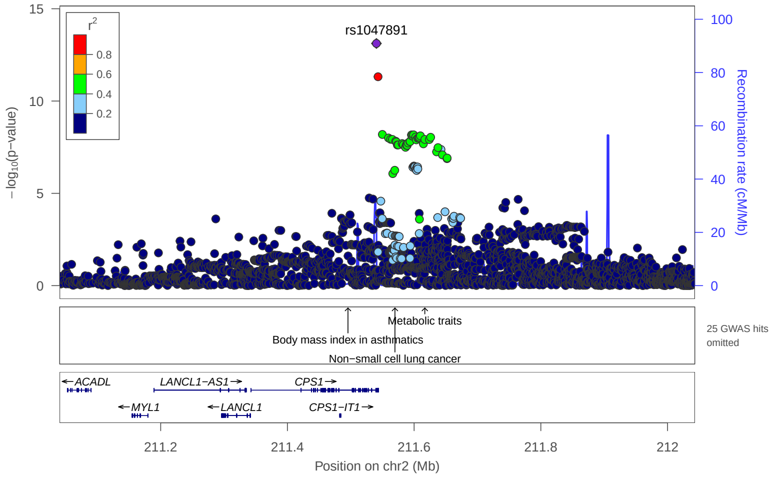

2−methylserine

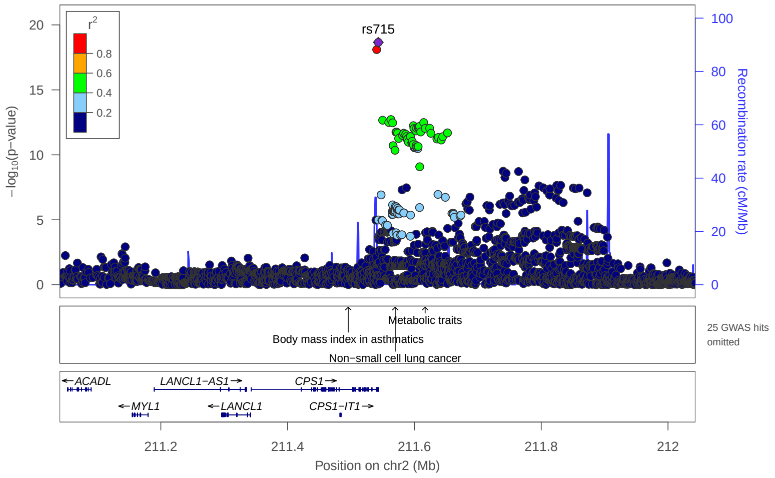

3−methylglutarate/2−methylglutarate

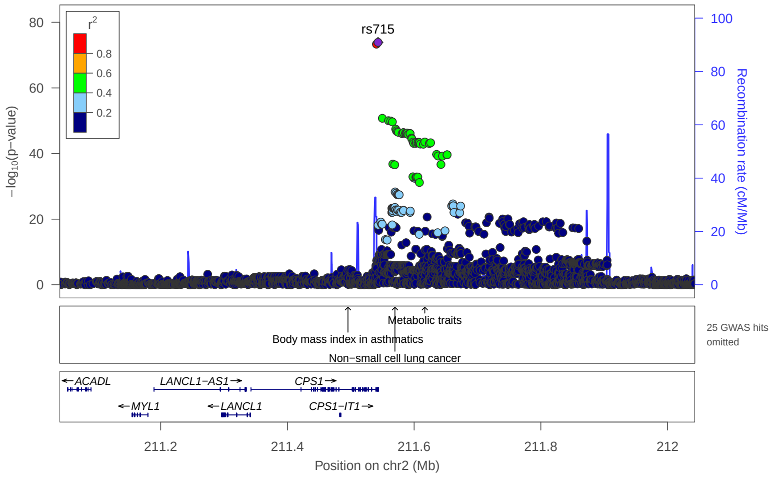

indolepropionylglycine

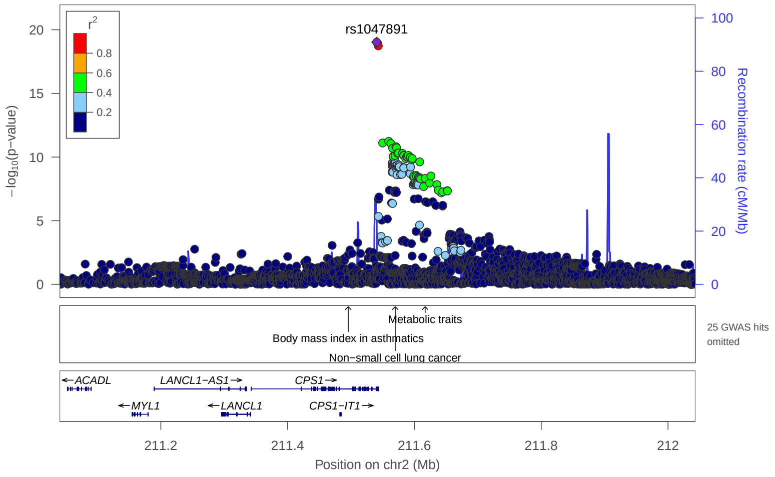

trans−3,4−methyleneheptanoylglycine

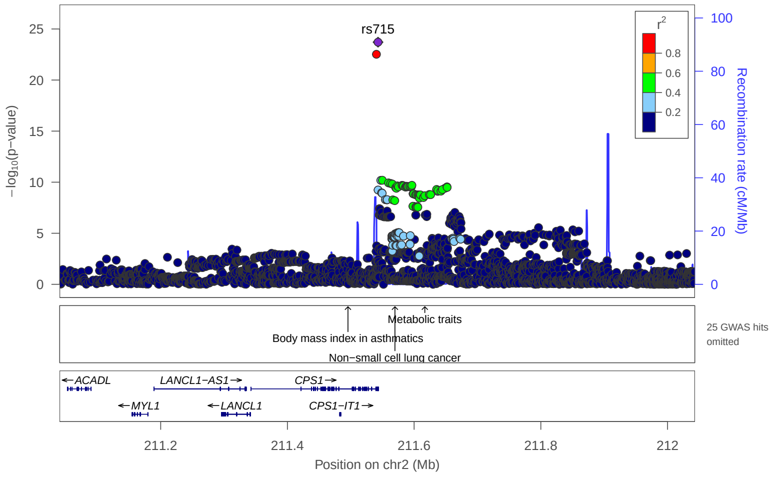

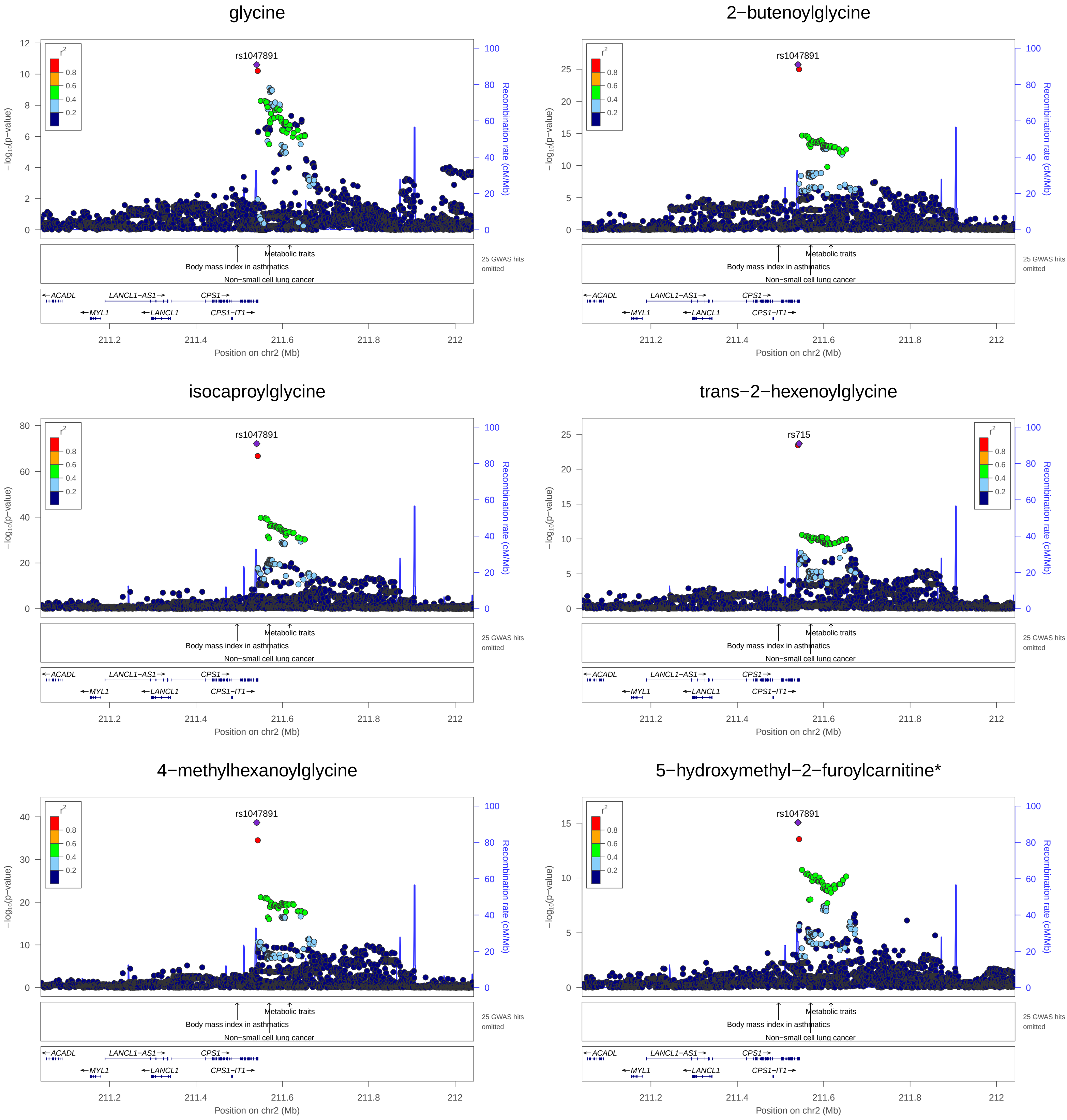
glycine
rs1047891
2−butenoylglycine
rs1047891
isocaproylglycine
rs1047891
trans−2−hexenoylglycine
rs715
4−methylhexanoylglycine
rs1047891
5−hydroxymethyl−2−furoylcarnitine*
rs1047891
r²
0.8
0.6
0.4
0.2
−log10(p−value)
Recombination rate (cM/Mb)
Metabolic traits
Body mass index in asthmatics
Non−small cell lung cancer
25 GWAS hits omitted
ACADL
LANCL1−AS1
CPS1
MYL1
LANCL1
CPS1−IT1
211.2
211.4
211.6
211.8
212
Position on chr2 (Mb)

X − 13431

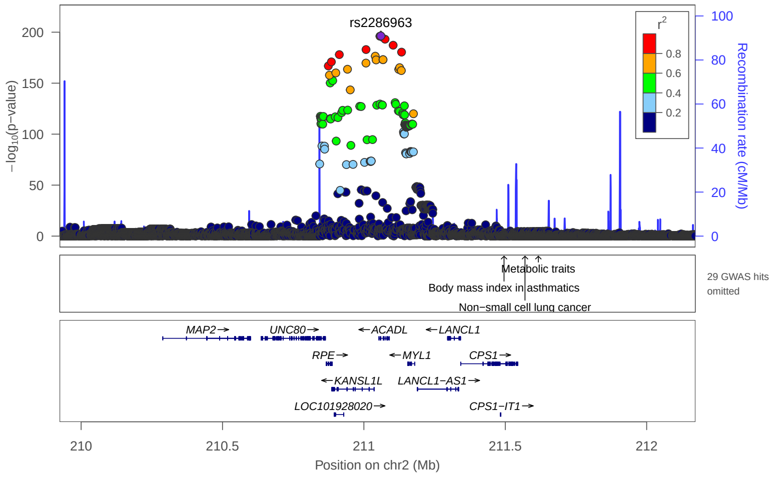

X − 23641

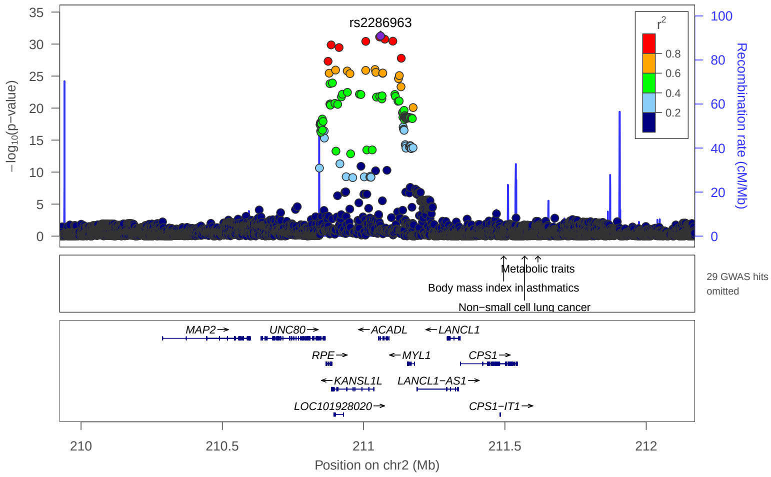

X − 24798

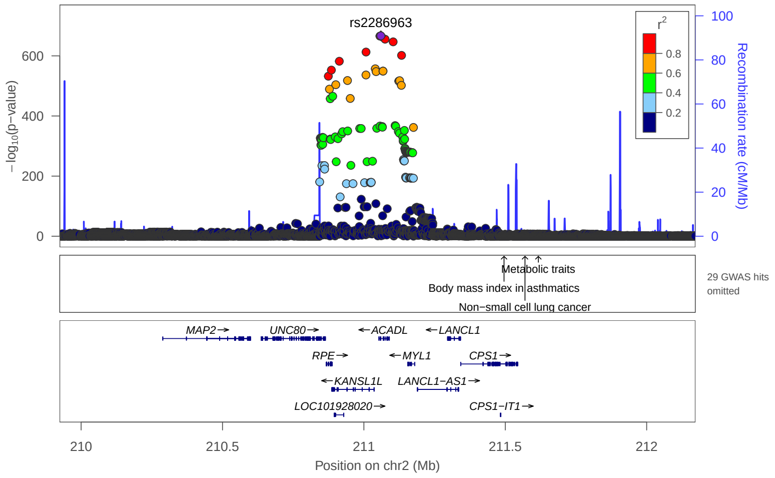

glycine conjugate of C9H16O2*

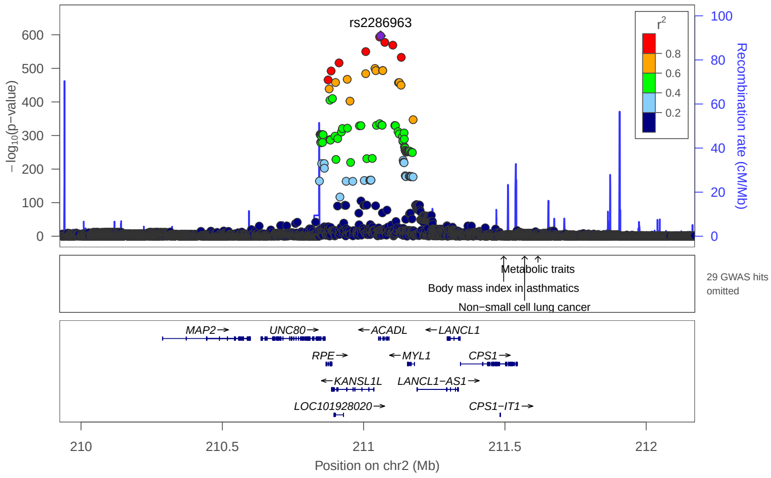

3−methylglutarate/2−methylglutarate

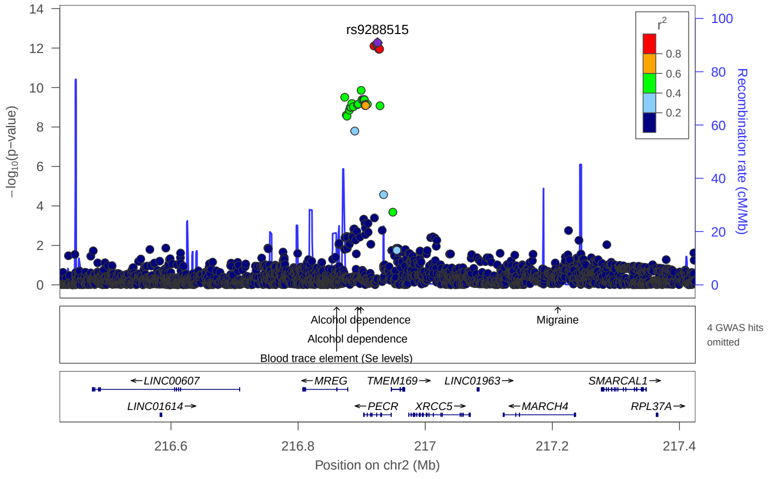

5−(galactosylhydroxy)−L−lysine

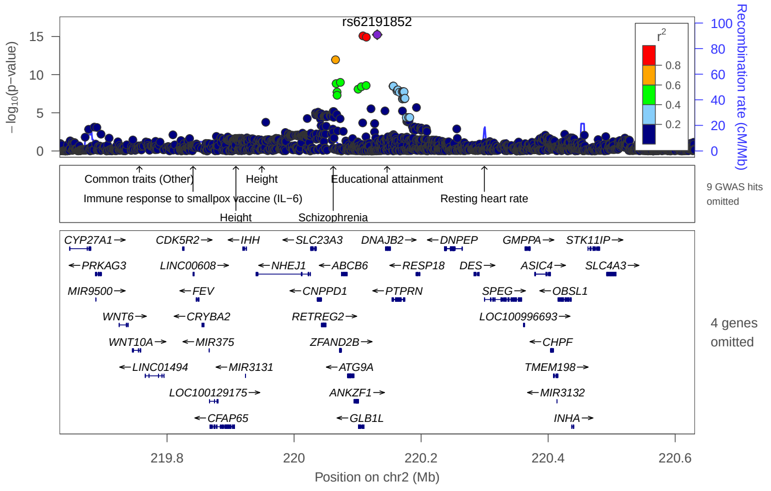

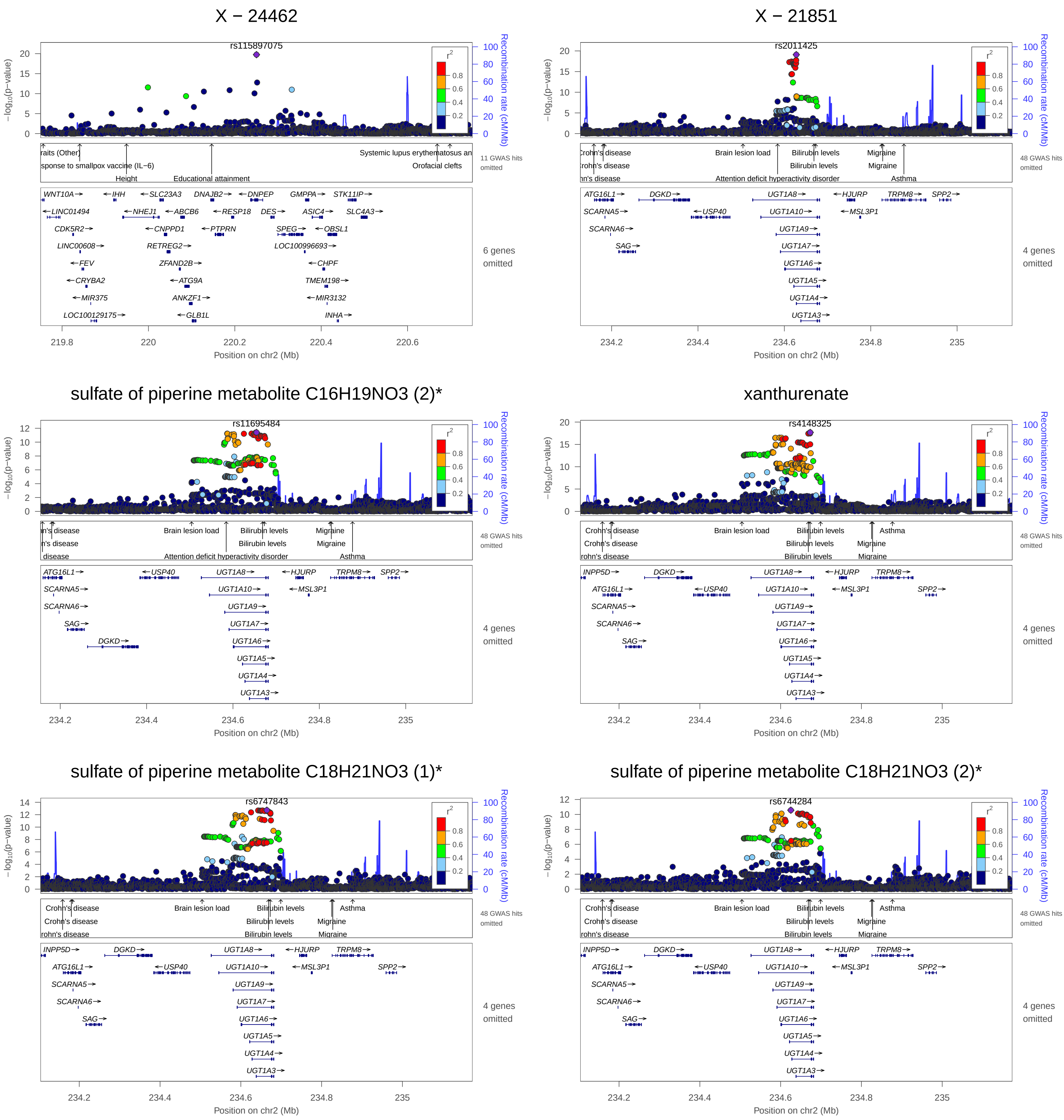
X − 24462
rs115897075
X − 21851
rs2011425
sulfate of piperine metabolite C16H19NO3 (2)*
rs11695484
xanthurenate
rs4148325
sulfate of piperine metabolite C18H21NO3 (1)*
rs6747843
sulfate of piperine metabolite C18H21NO3 (2)*
rs6744284
Position on chr2 (Mb)
Recombination rate (cM/Mb)
11 GWAS hits omitted
48 GWAS hits omitted
6 genes omitted
4 genes omitted

sulfate of piperine metabolite C18H21NO3 (3)*

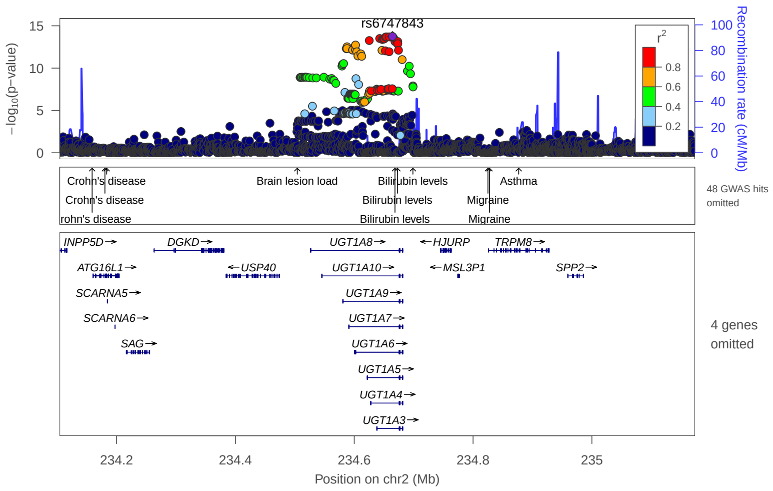

sulfate of piperine metabolite C16H17NO3 (1)*

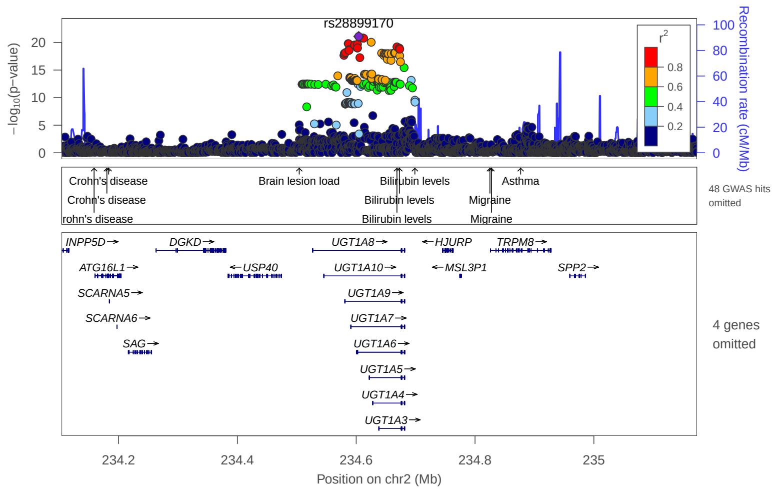

2−hydroxysebacate

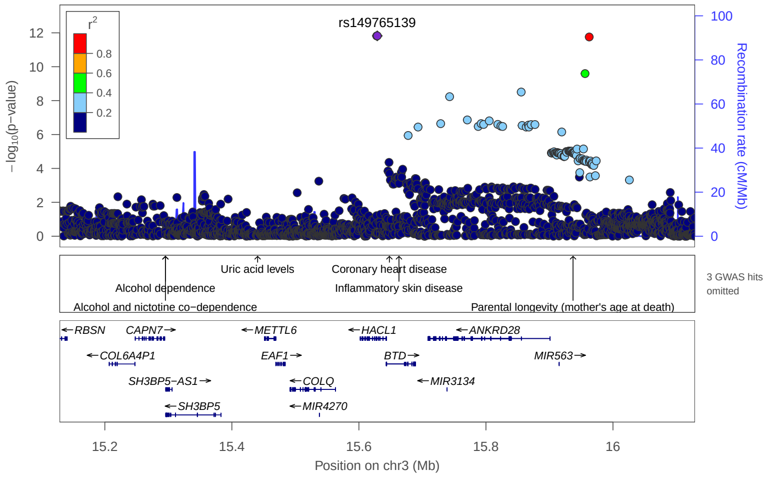

N−acetylcarnosine

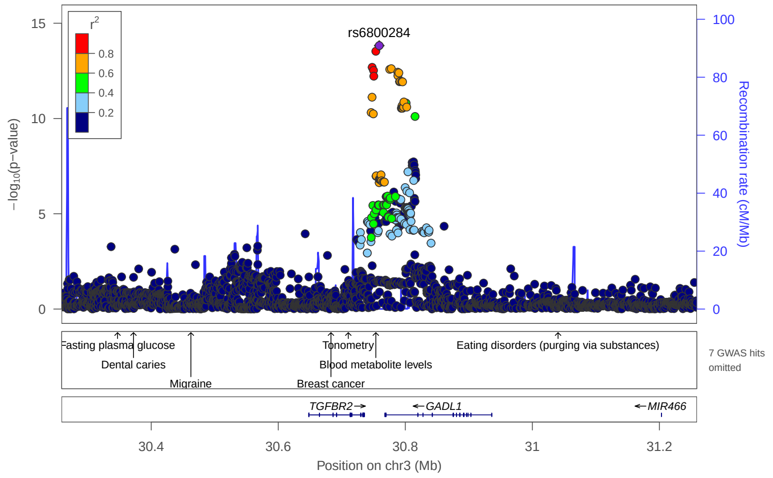

dimethylglycine

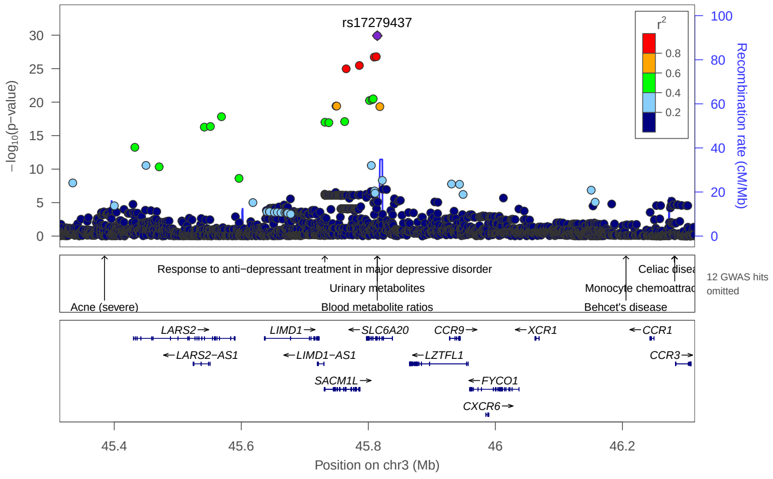

succinylcarnitine (C4−DC)

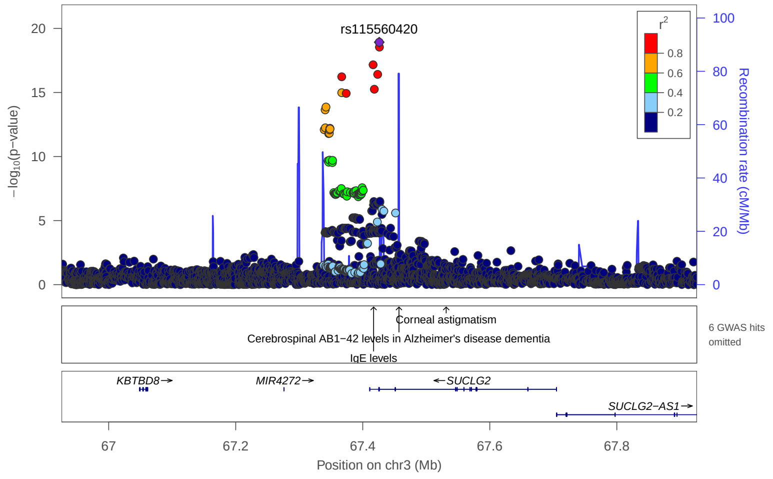

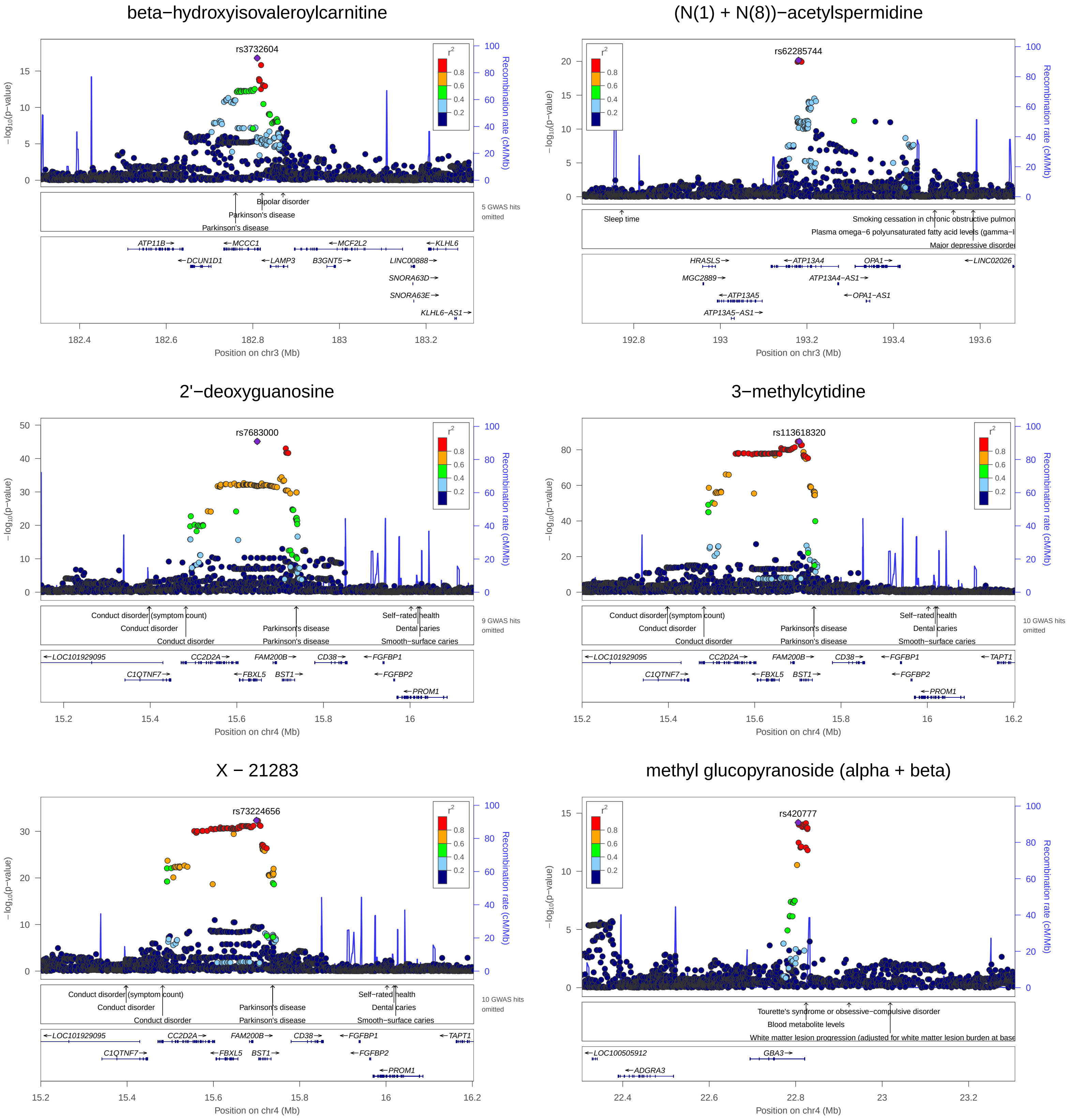
beta−hydroxyisovaleroylcarnitine
rs3732604
Bipolar disorder
Parkinson's disease
Parkinson's disease
5 GWAS hits omitted
ATP11B
MCCC1
MCF2L2
KLHL6
DCUN1D1
LAMP3
B3GNT5
LINC00888
SNORA63D
SNORA63E
KLHL6−AS1
182.4
182.6
182.8
183
183.2
Position on chr3 (Mb)
(N(1) + N(8))−acetylspermidine
rs62285744
Sleep time
Smoking cessation in chronic obstructive pulmon
Plasma omega−6 polyunsaturated fatty acid levels (gamma−li
Major depressive disorder
HRASLS
ATP13A4
OPA1
LINC02026
MGC2889
ATP13A4−AS1
ATP13A5
OPA1−AS1
ATP13A5−AS1
192.8
193
193.2
193.4
193.6
Position on chr3 (Mb)
2'−deoxyguanosine
rs7683000
Conduct disorder (symptom count)
Conduct disorder
Conduct disorder
Parkinson's disease
Parkinson's disease
Self−rated health
Dental caries
Smooth−surface caries
9 GWAS hits omitted
LOC101929095
CC2D2A
FAM200B
CD38
FGFBP1
C1QTNF7
FBXL5
BST1
FGFBP2
PROM1
15.2
15.4
15.6
15.8
16
Position on chr4 (Mb)
3−methylcytidine
rs113618320
10 GWAS hits omitted
TAPT1
16.2
Position on chr4 (Mb)
X − 21283
rs73224656
10 GWAS hits omitted
Position on chr4 (Mb)
methyl glucopyranoside (alpha + beta)
rs420777
Tourette's syndrome or obsessive−compulsive disorder
Blood metabolite levels
White matter lesion progression (adjusted for white matter lesion burden at base
LOC100505912
GBA3
ADGRA3
22.4
22.6
22.8
23
23.2
Position on chr4 (Mb)
−log10(p−value)
Recombination rate (cM/Mb)
r2

X − 13847

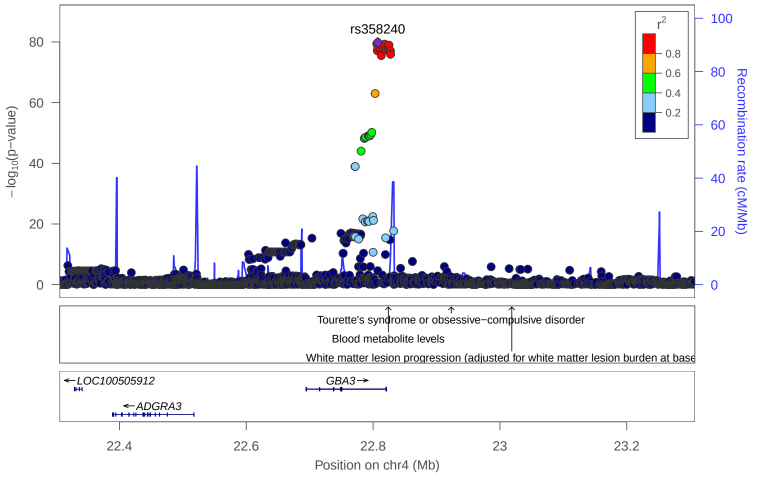

X − 23299

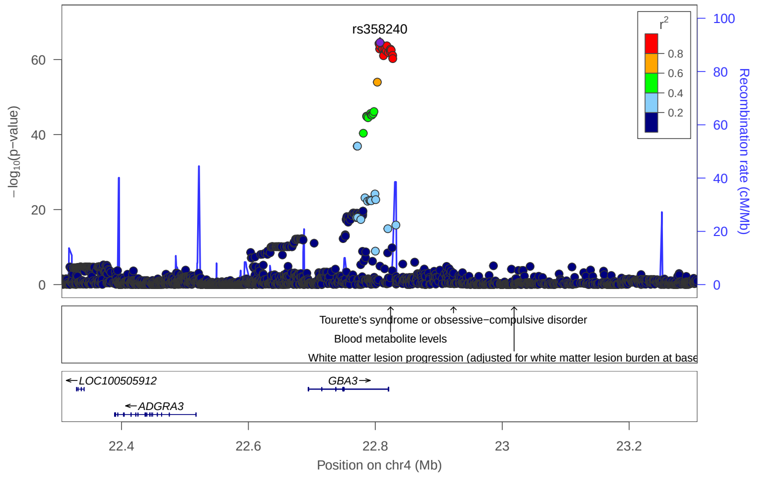

X − 23311

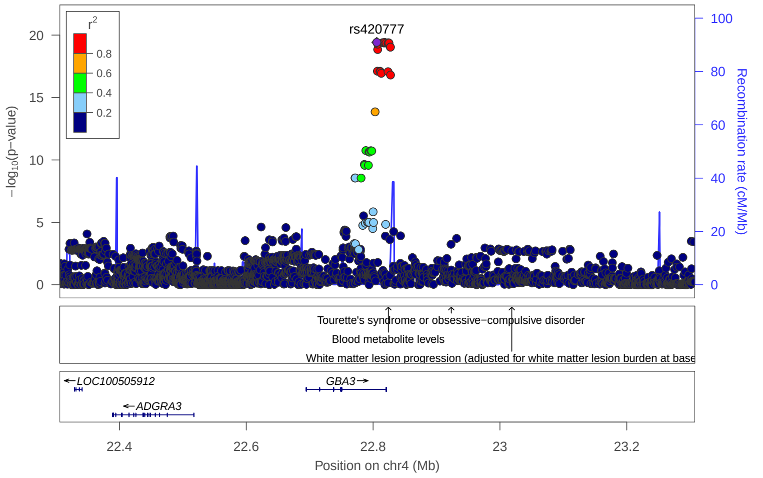

ethyl beta−glucopyranoside

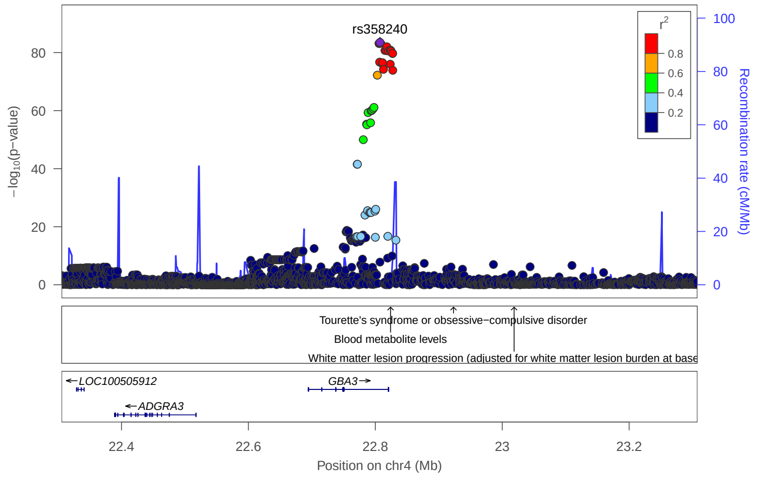

X − 24838

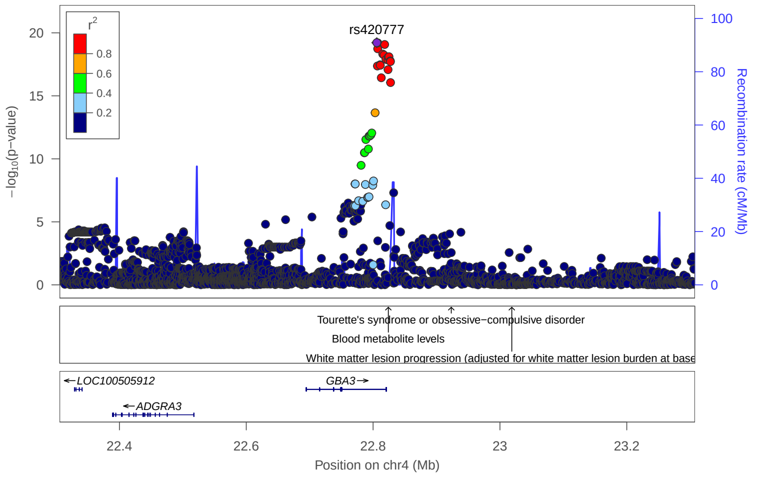

X − 24417

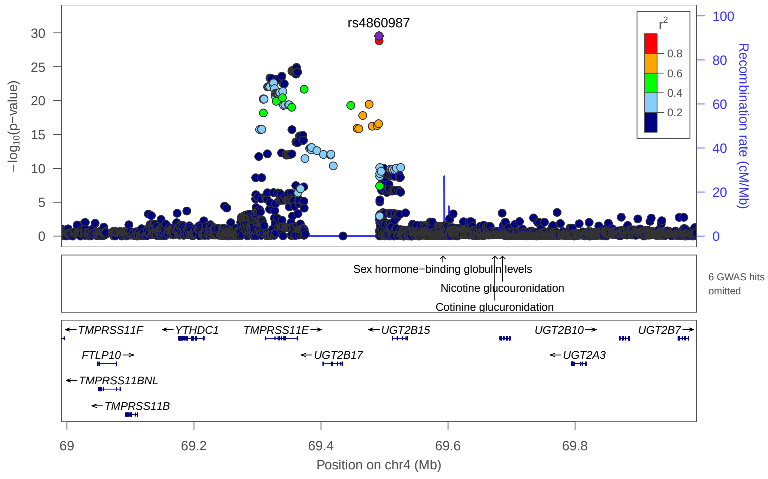

vanillylmandelate (VMA)

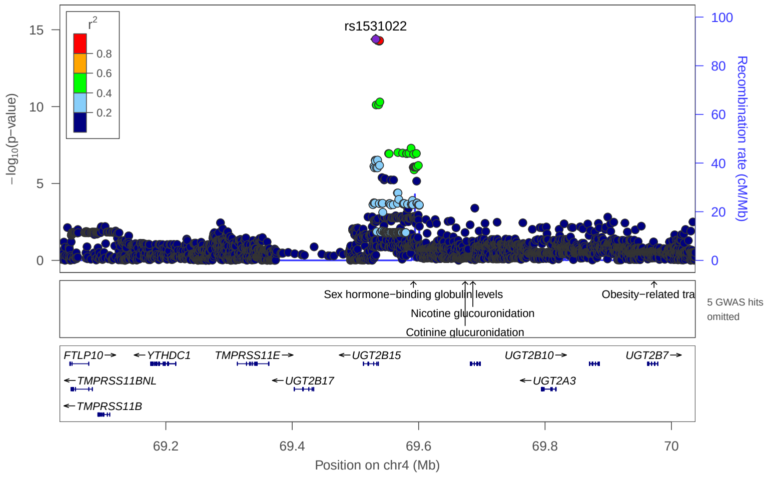

X − 12701

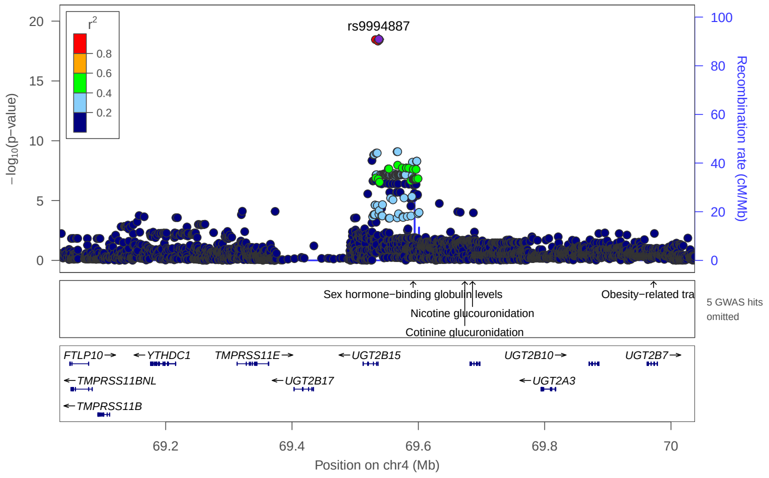

3alpha,21−dihydroxy−5beta−pregnane−11,20−dione 21−glucuronide

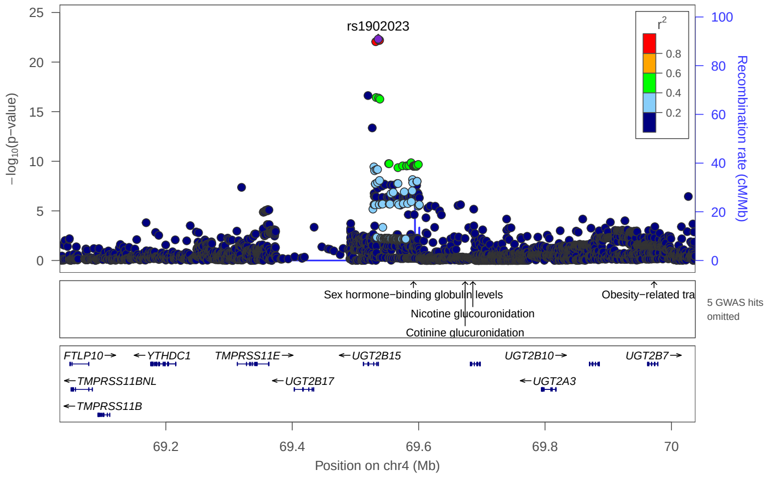

X − 17328

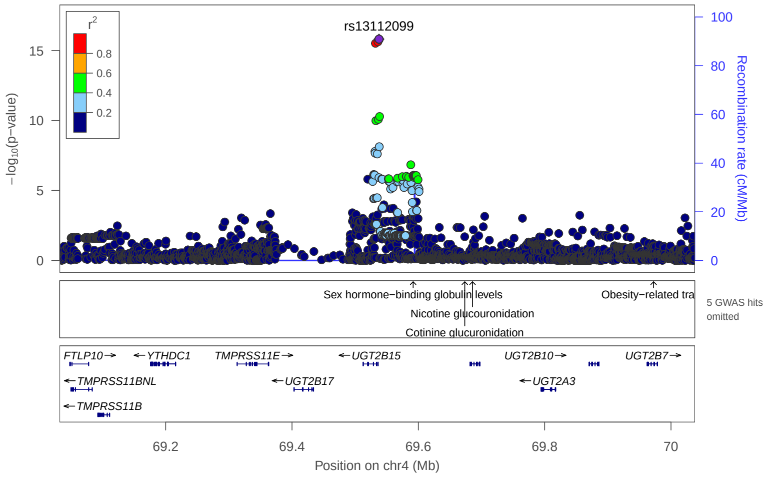

X − 13688

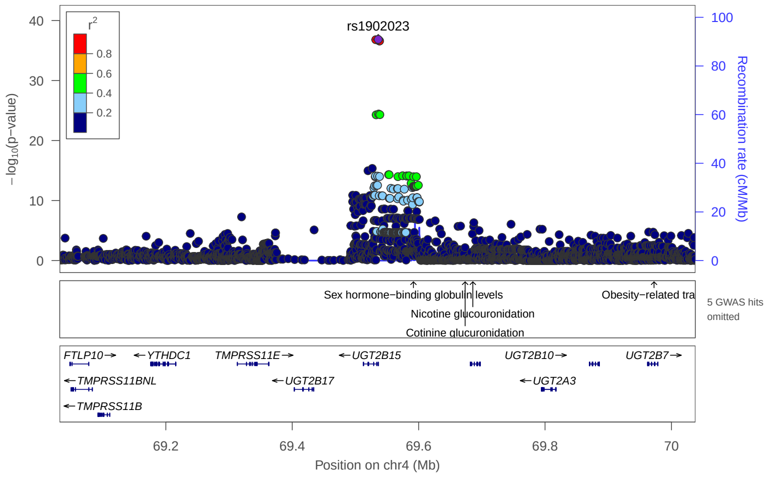

X − 24418

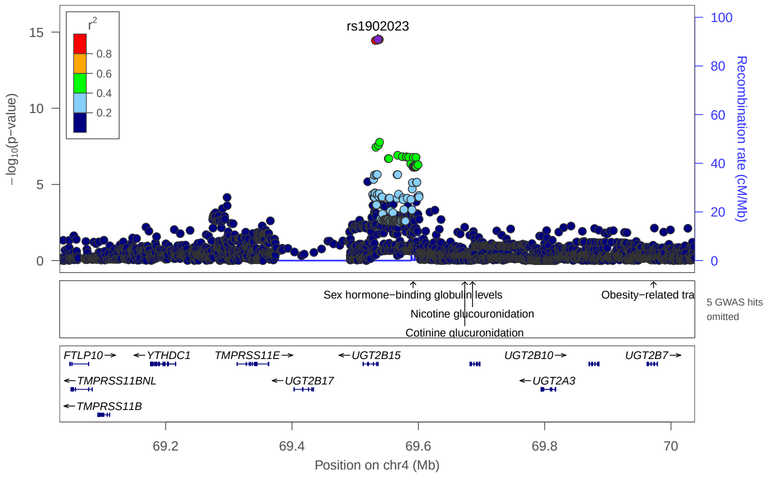

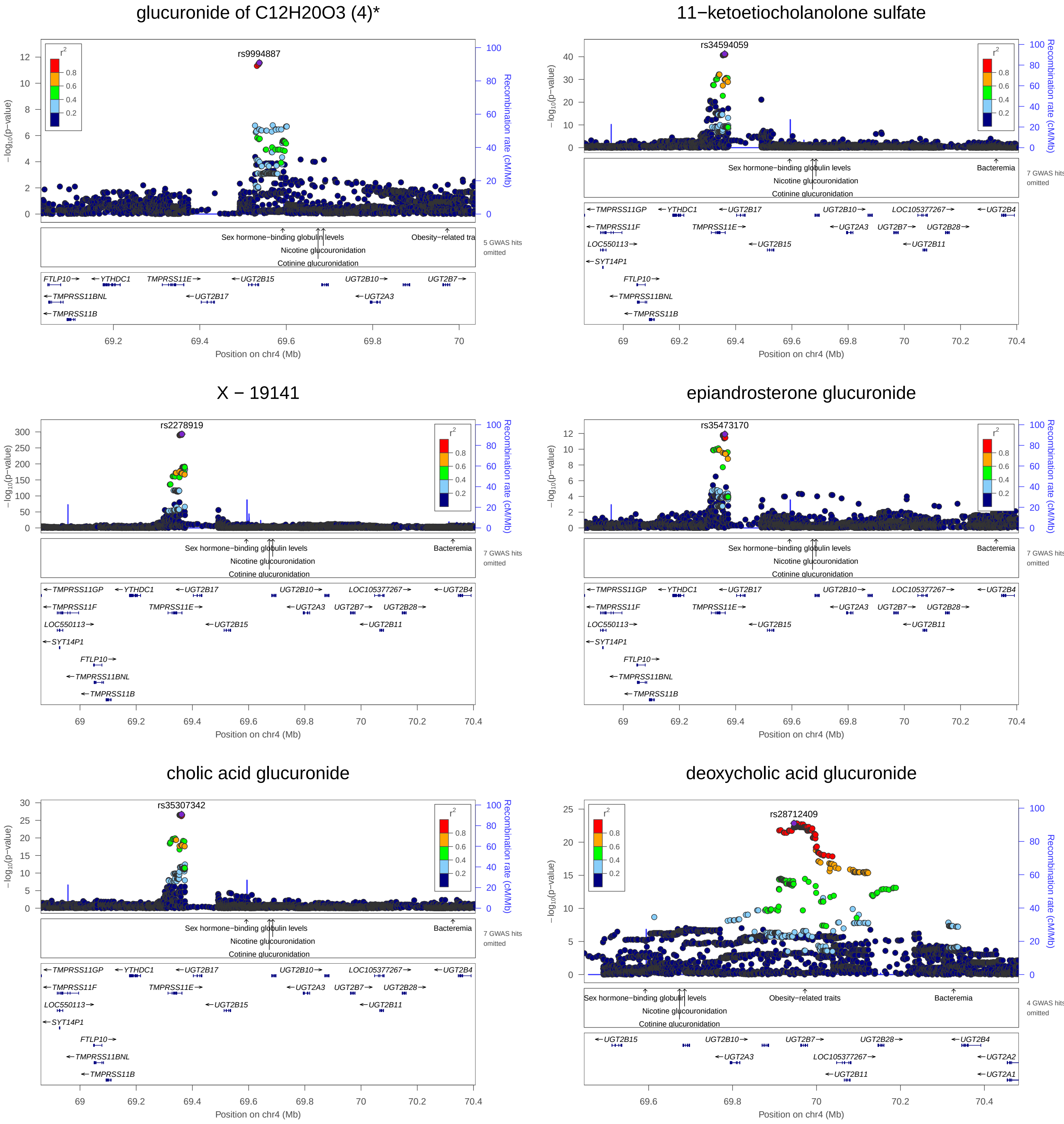
glucuronide of C12H20O3 (4)*
rs9994887
−log10(p−value)
Recombination rate (cM/Mb)
Sex hormone−binding globulin levels
Nicotine glucuronidation
Cotinine glucuronidation
Obesity−related tra
5 GWAS hits omitted
FTLP10
YTHDC1
TMPRSS11E
UGT2B15
UGT2B10
UGT2B7
TMPRSS11BNL
UGT2B17
UGT2A3
TMPRSS11B
Position on chr4 (Mb)
11−ketoetiocholanolone sulfate
rs34594059
Bacteremia
7 GWAS hits omitted
TMPRSS11GP
TMPRSS11F
LOC550113
SYT14P1
UGT2B28
UGT2B11
UGT2B4
LOC105377267
X − 19141
rs2278919
epiandrosterone glucuronide
rs35473170
cholic acid glucuronide
rs35307342
deoxycholic acid glucuronide
rs28712409
Obesity−related traits
4 GWAS hits omitted
UGT2A2
UGT2A1

glucuronide of C12H22O4 (2)*

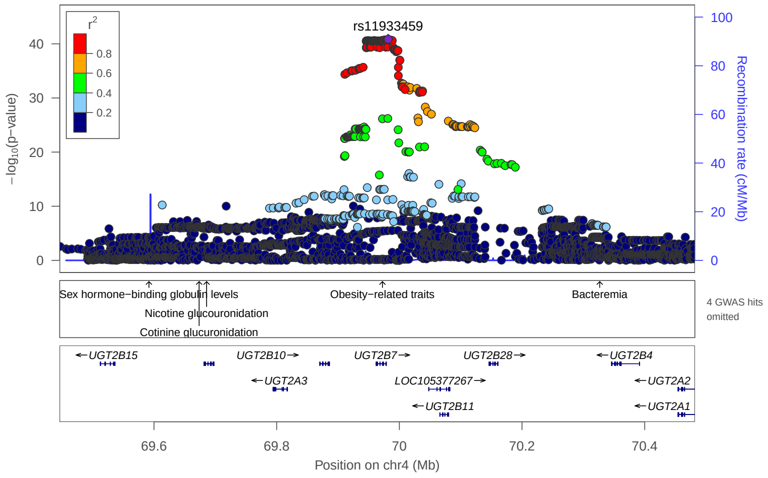

glucuronide of C14H26O4 (2)*

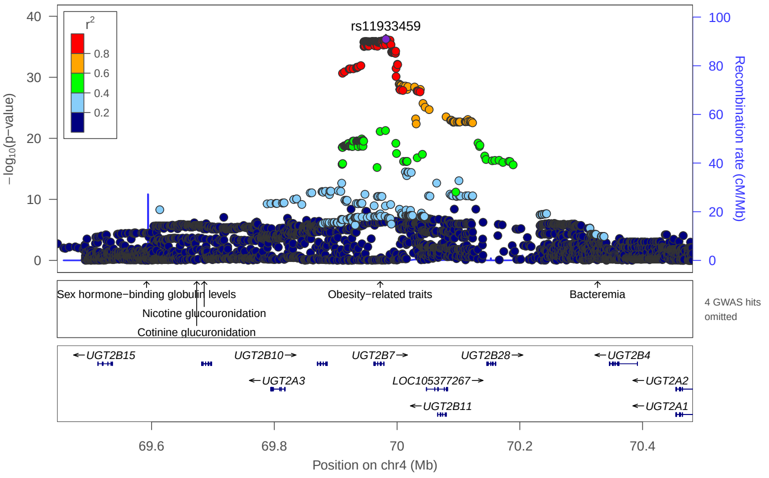

tetrahydrocortisone glucuronide (5)

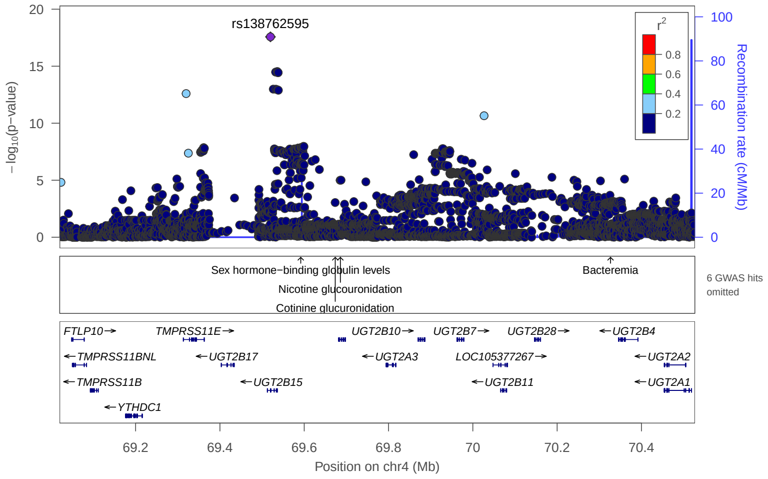

orotidine

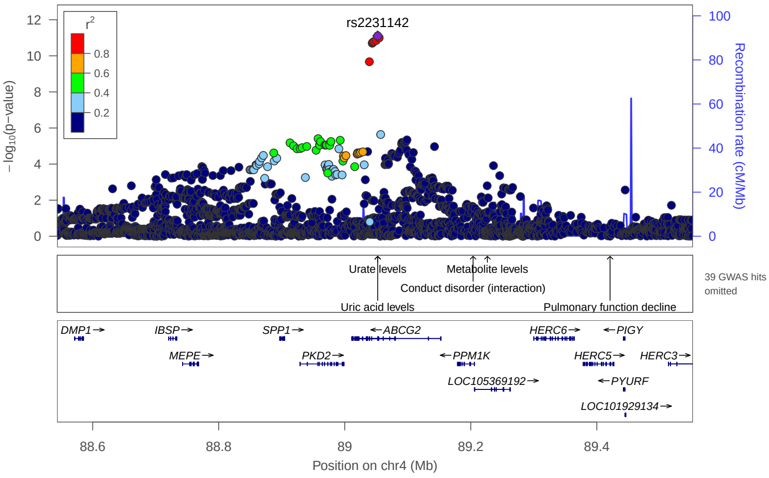

X − 11612

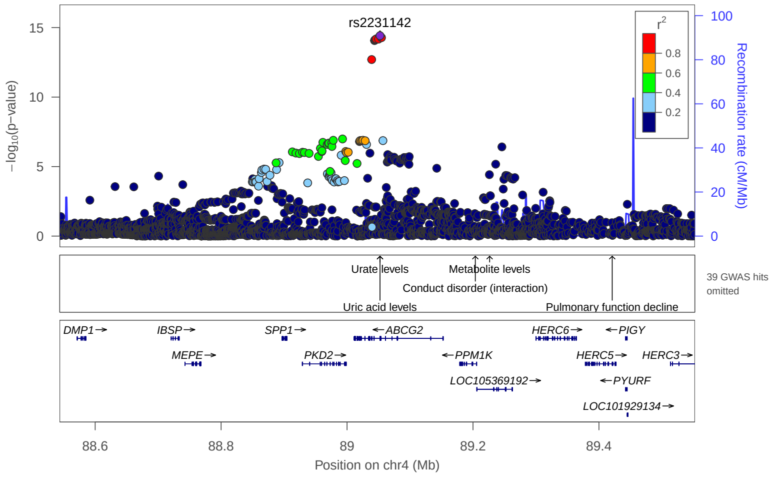

sulfate of piperine metabolite C18H21NO3 (1)*

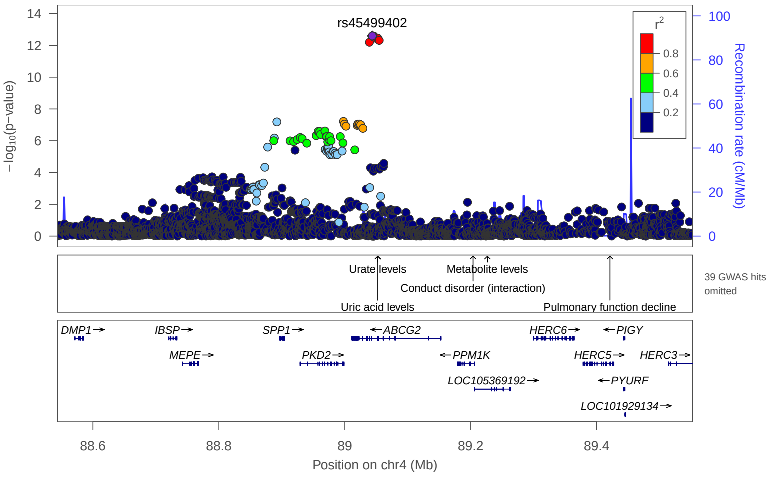

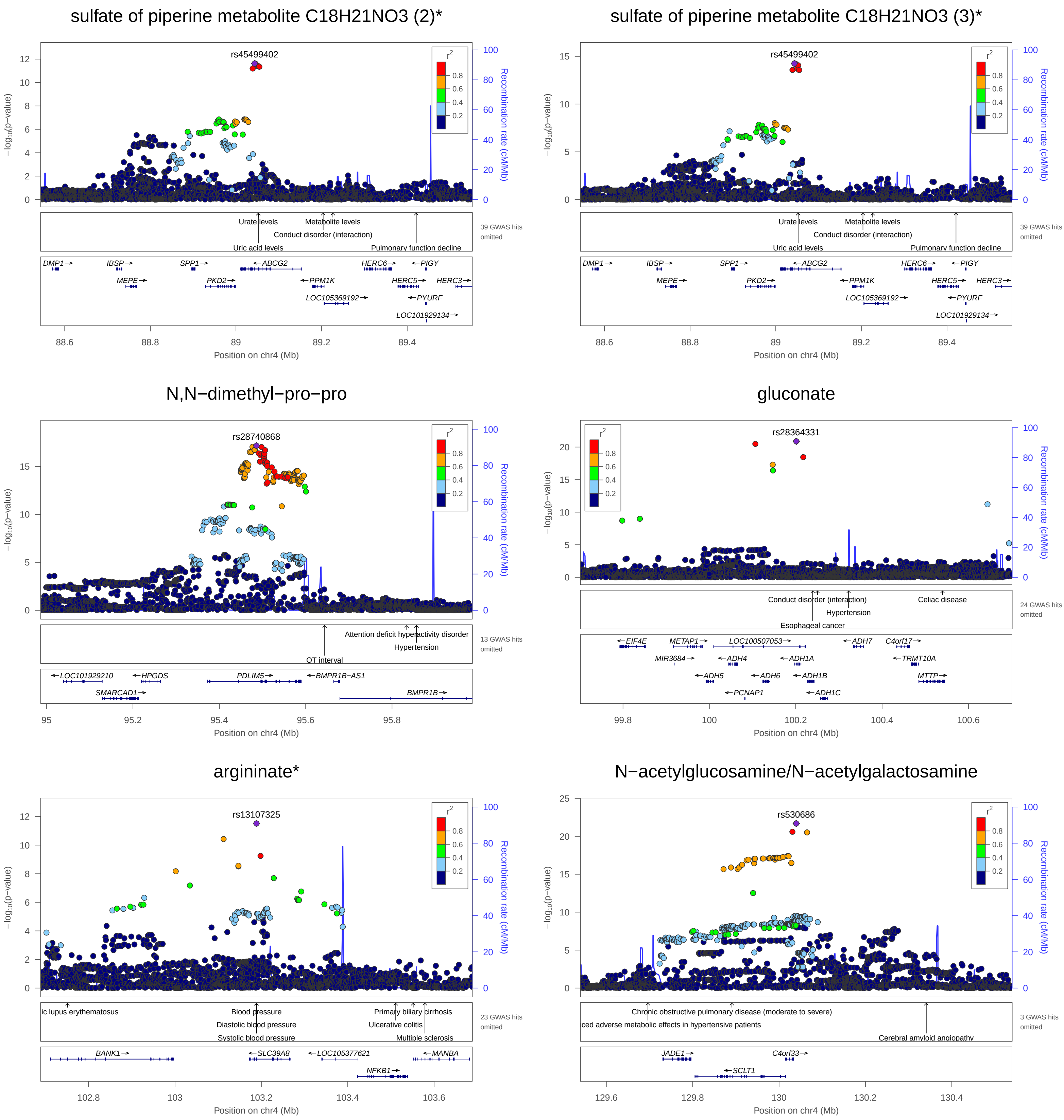

sulfate of piperine metabolite C18H21NO3 (2)*
rs45499402
39 GWAS hits omitted
Position on chr4 (Mb)
sulfate of piperine metabolite C18H21NO3 (3)*
rs45499402
39 GWAS hits omitted
Position on chr4 (Mb)
N,N−dimethyl−pro−pro
rs28740868
13 GWAS hits omitted
Position on chr4 (Mb)
gluconate
rs28364331
24 GWAS hits omitted
Position on chr4 (Mb)
argininate*
rs13107325
23 GWAS hits omitted
Position on chr4 (Mb)
N−acetylglucosamine/N−acetylgalactosamine
rs530686
3 GWAS hits omitted
Position on chr4 (Mb)

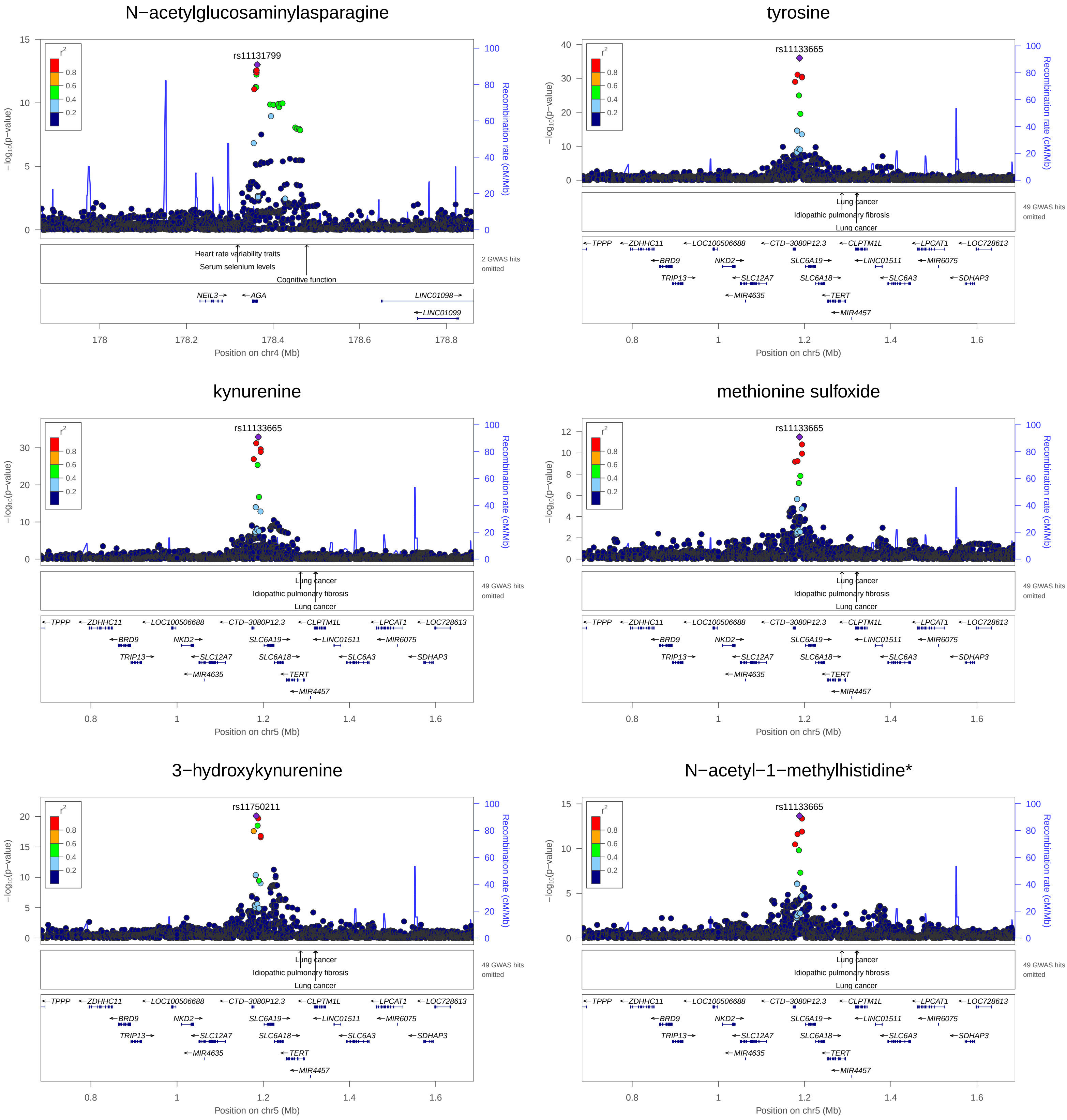

N−acetylglucosaminylasparagine
rs11131799
Heart rate variability traits
Serum selenium levels
Cognitive function
2 GWAS hits omitted
NEIL3
AGA
LINC01098
LINC01099
Position on chr4 (Mb)
tyrosine
rs11133665
kynurenine
rs11133665
methionine sulfoxide
rs11133665
3−hydroxykynurenine
rs11750211
N−acetyl−1−methylhistidine*
rs11133665
Lung cancer
Idiopathic pulmonary fibrosis
49 GWAS hits omitted
TPPP
ZDHHC11
LOC100506688
CTD−3080P12.3
CLPTM1L
LPCAT1
LOC728613
BRD9
NKD2
SLC6A19
LINC01511
MIR6075
TRIP13
SLC12A7
SLC6A18
SLC6A3
SDHAP3
MIR4635
TERT
MIR4457
Position on chr5 (Mb)
r2
−log10(p−value)
Recombination rate (cM/Mb)

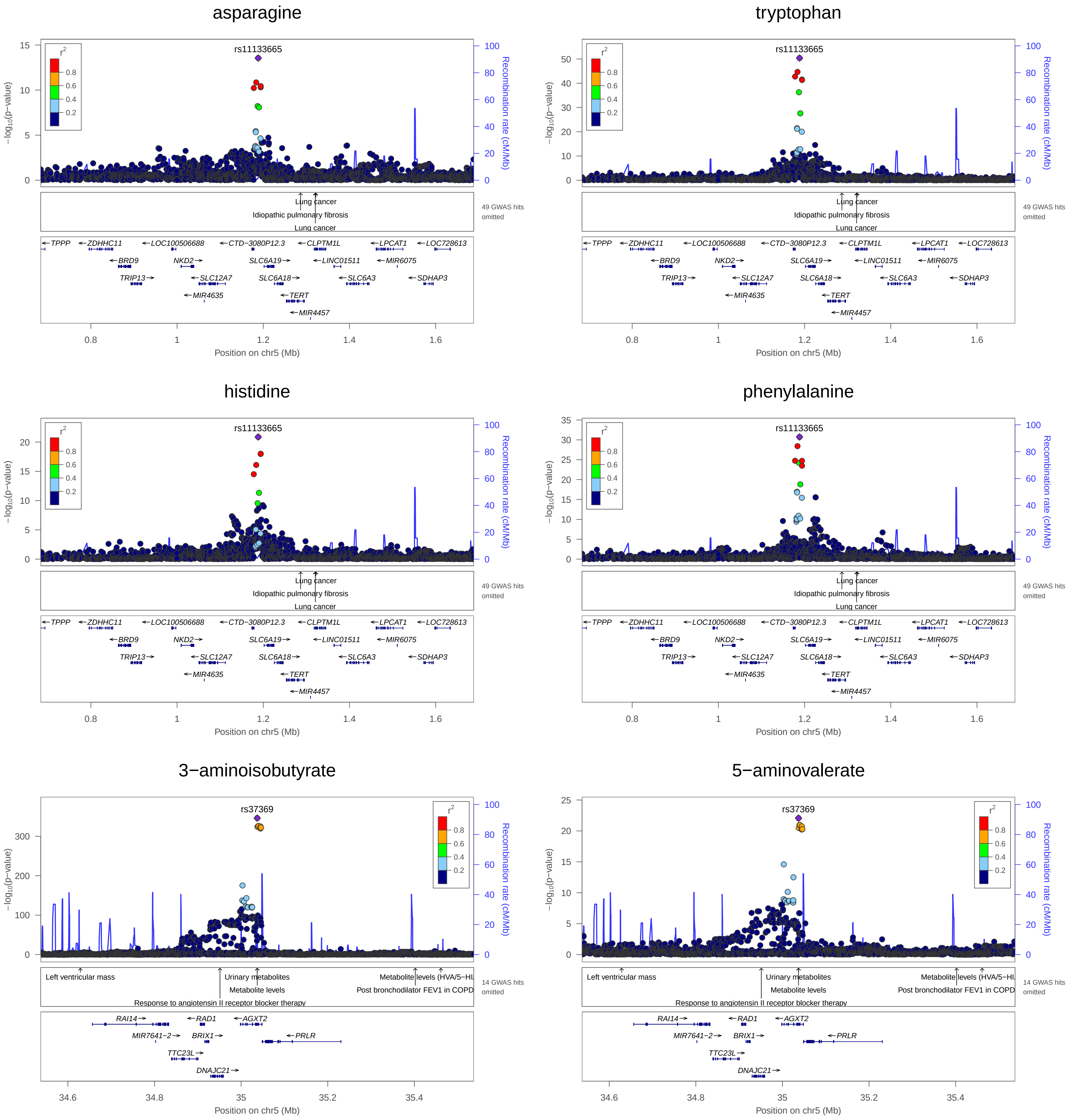

asparagine
tryptophan
histidine
phenylalanine
3−aminoisobutyrate
5−aminovalerate
rs11133665
rs37369
r2
0.8
0.6
0.4
0.2
−log10(p−value)
Recombination rate (cM/Mb)
Position on chr5 (Mb)
Lung cancer
Idiopathic pulmonary fibrosis
49 GWAS hits omitted
14 GWAS hits omitted
Left ventricular mass
Urinary metabolites
Metabolite levels
Metabolite levels (HVA/5−HI...
Post bronchodilator FEV1 in COPD
Response to angiotensin II receptor blocker therapy
TPPP
ZDHHC11
LOC100506688
CTD−3080P12.3
CLPTM1L
LPCAT1
LOC728613
BRD9
NKD2
SLC6A19
LINC01511
MIR6075
TRIP13
SLC12A7
SLC6A18
SLC6A3
SDHAP3
MIR4635
TERT
MIR4457
RAI14
RAD1
AGXT2
MIR7641−2
BRIX1
PRLR
TTC23L
DNAJC21

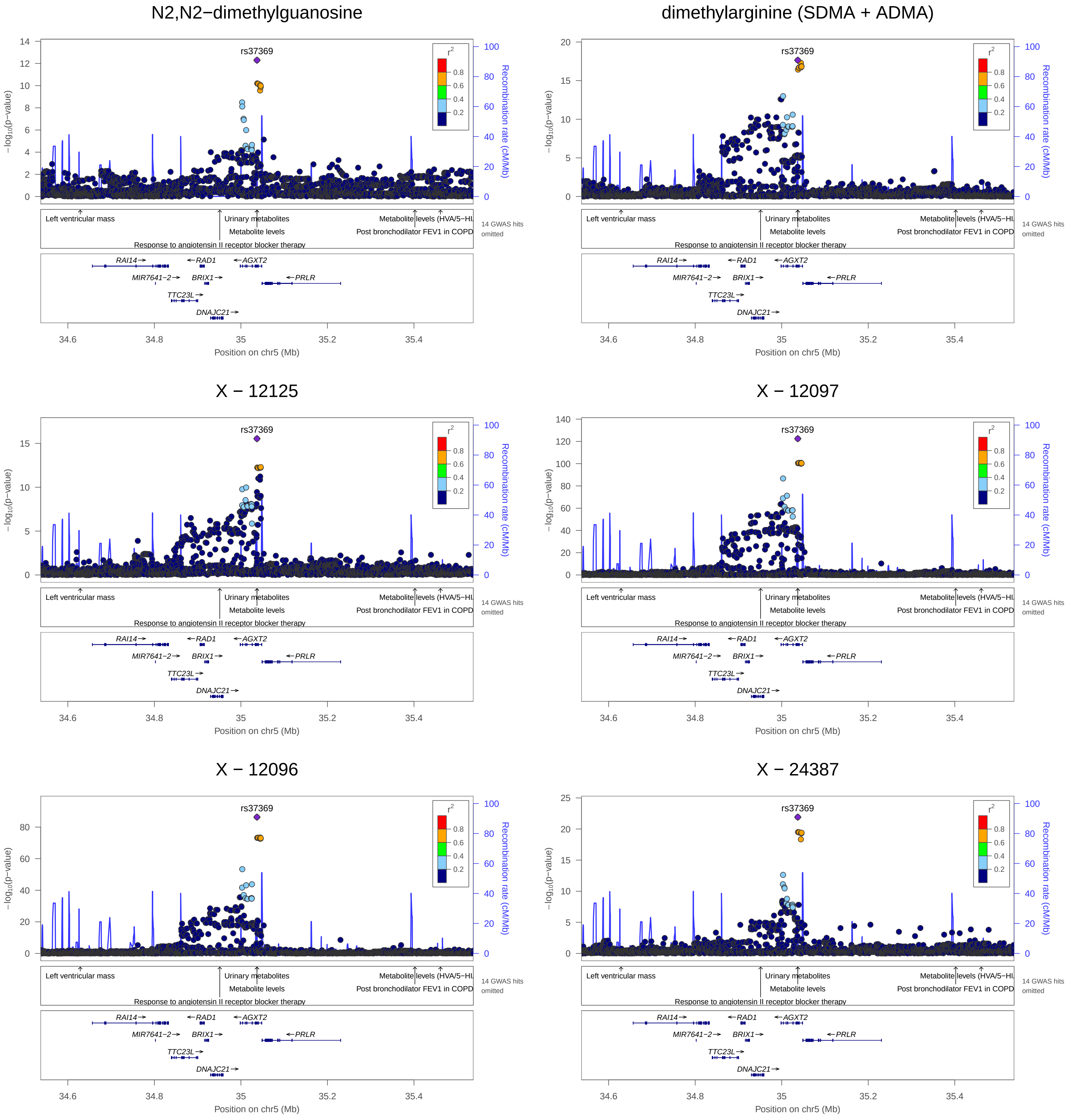
N2,N2−dimethylguanosine
dimethylarginine (SDMA + ADMA)
X − 12125
X − 12097
X − 12096
X − 24387
rs37369
r2
0.8
0.6
0.4
0.2
−log10(p−value)
Recombination rate (cM/Mb)
Left ventricular mass
Urinary metabolites
Metabolite levels
Metabolite levels (HVA/5−HI
Post bronchodilator FEV1 in COPD
Response to angiotensin II receptor blocker therapy
14 GWAS hits omitted
RAI14
RAD1
AGXT2
MIR7641−2
BRIX1
PRLR
TTC23L
DNAJC21
34.6
34.8
35
35.2
35.4
Position on chr5 (Mb)

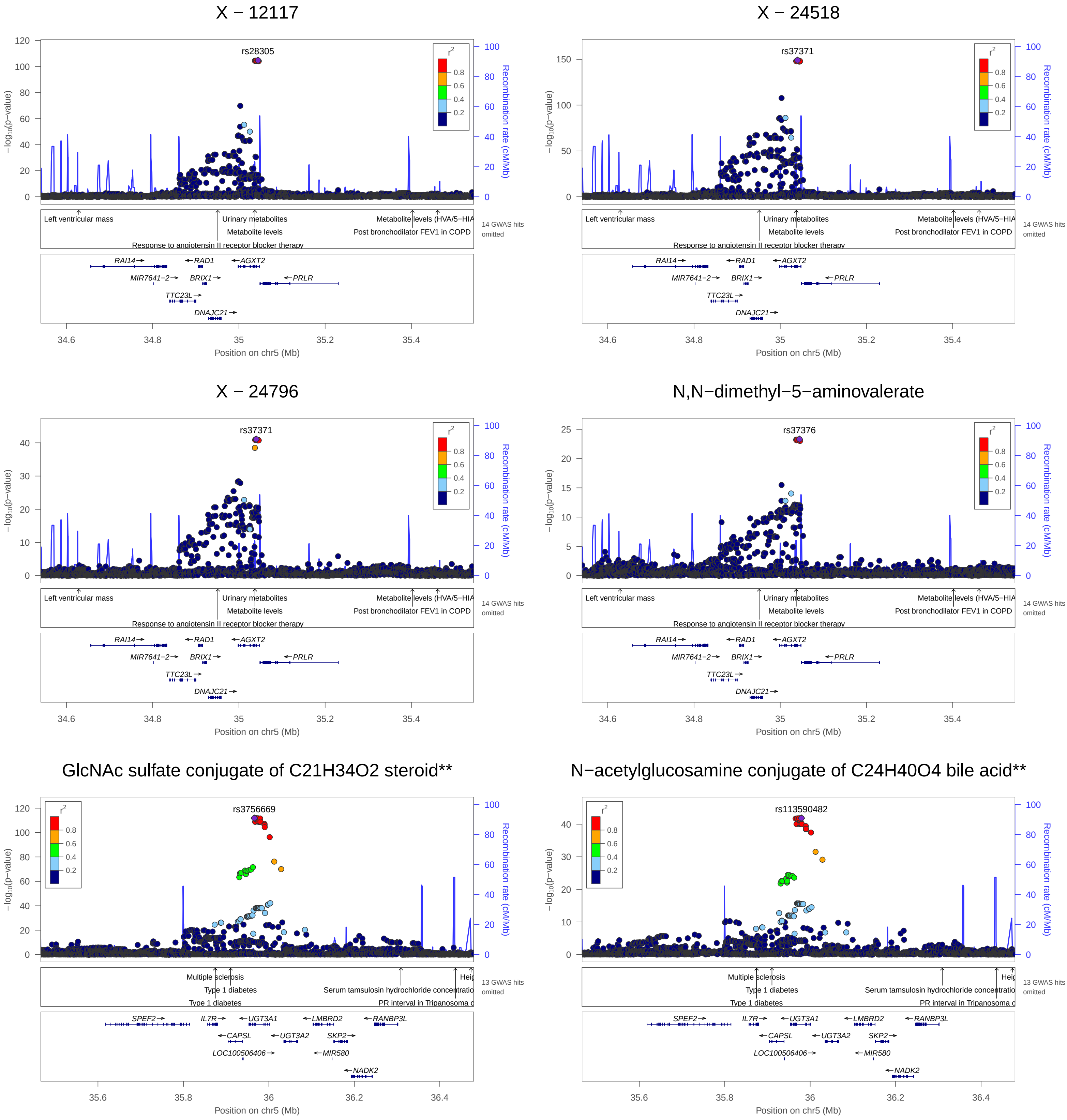

X − 12117
X − 24518
X − 24796
N,N−dimethyl−5−aminovalerate
GlcNAc sulfate conjugate of C21H34O2 steroid**
N−acetylglucosamine conjugate of C24H40O4 bile acid**
rs28305
rs37371
rs37376
rs3756669
rs113590482
−log10(p−value)
Recombination rate (cM/Mb)
r2
0.8
0.6
0.4
0.2
Left ventricular mass
Urinary metabolites
Metabolite levels
Metabolite levels (HVA/5−HIA
Post bronchodilator FEV1 in COPD
Response to angiotensin II receptor blocker therapy
14 GWAS hits omitted
Multiple sclerosis
Type 1 diabetes
Serum tamsulosin hydrochloride concentratio
PR interval in Tripanosoma c
Heig
13 GWAS hits omitted
RAI14
RAD1
AGXT2
MIR7641−2
BRIX1
PRLR
TTC23L
DNAJC21
SPEF2
IL7R
UGT3A1
LMBRD2
RANBP3L
CAPSL
UGT3A2
SKP2
LOC100506406
MIR580
NADK2
Position on chr5 (Mb)

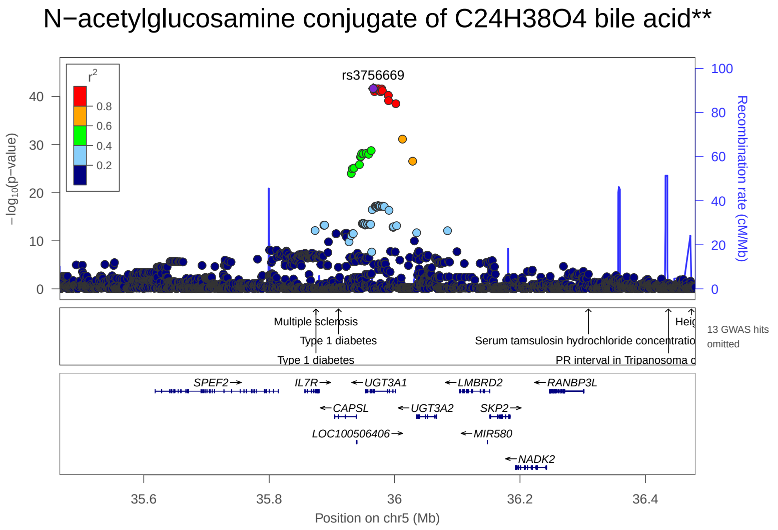
N−acetylglucosamine conjugate of C24H38O4 bile acid**
rs3756669
−log10(p−value)
Recombination rate (cM/Mb)
Multiple sclerosis
Type 1 diabetes
Serum tamsulosin hydrochloride concentratio
PR interval in Tripanosoma c
13 GWAS hits omitted
SPEF2
IL7R
UGT3A1
LMBRD2
RANBP3L
CAPSL
UGT3A2
SKP2
LOC100506406
MIR580
NADK2
Position on chr5 (Mb)

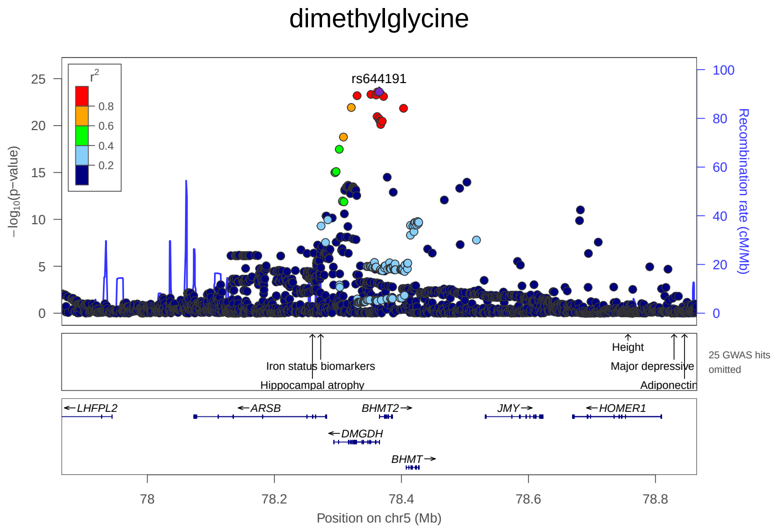
dimethylglycine
rs644191
−log10(p−value)
Recombination rate (cM/Mb)
Iron status biomarkers
Hippocampal atrophy
Height
Major depressive
Adiponectin
25 GWAS hits omitted
LHFPL2
ARSB
BHMT2
JMY
HOMER1
DMGDH
BHMT
Position on chr5 (Mb)

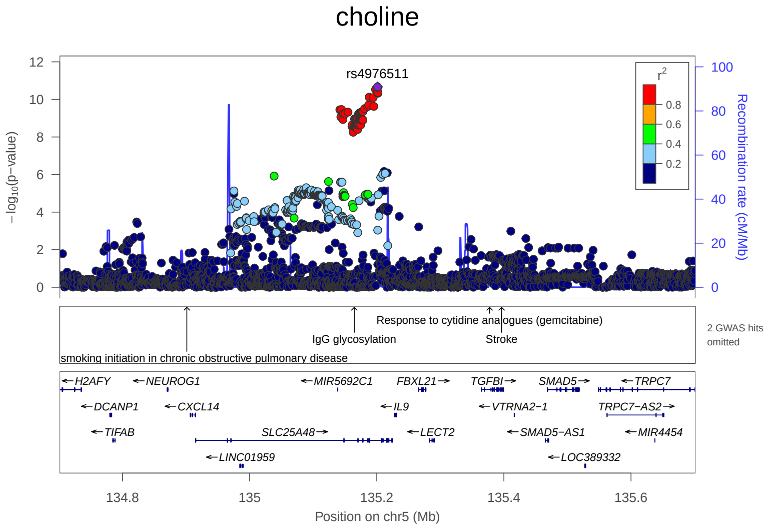
choline
rs4976511
−log10(p−value)
Recombination rate (cM/Mb)
Response to cytidine analogues (gemcitabine)
IgG glycosylation
Stroke
smoking initiation in chronic obstructive pulmonary disease
2 GWAS hits omitted
H2AFY
NEUROG1
MIR5692C1
FBXL21
TGFBI
SMAD5
TRPC7
DCANP1
CXCL14
IL9
VTRNA2−1
TRPC7−AS2
TIFAB
SLC25A48
LECT2
SMAD5−AS1
MIR4454
LINC01959
LOC389332
Position on chr5 (Mb)

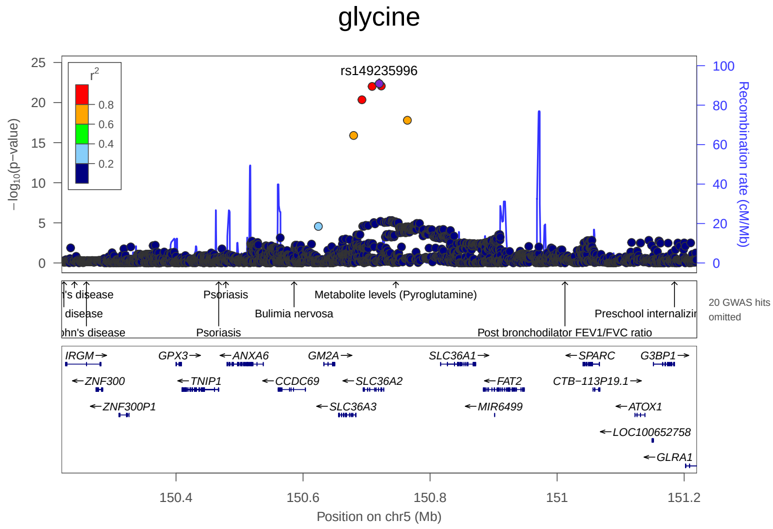
glycine
rs149235996
−log10(p−value)
Recombination rate (cM/Mb)
Psoriasis
Metabolite levels (Pyroglutamine)
Bulimia nervosa
Preschool internalizi
Post bronchodilator FEV1/FVC ratio
20 GWAS hits omitted
IRGM
GPX3
ANXA6
GM2A
SLC36A1
SPARC
G3BP1
ZNF300
TNIP1
CCDC69
SLC36A2
FAT2
CTB−113P19.1
ZNF300P1
SLC36A3
MIR6499
ATOX1
LOC100652758
GLRA1
Position on chr5 (Mb)

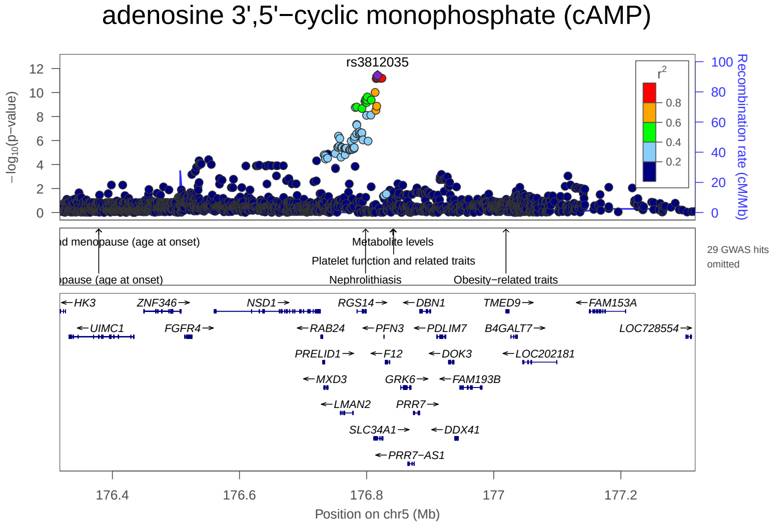
adenosine 3',5'−cyclic monophosphate (cAMP)
rs3812035
−log10(p−value)
Recombination rate (cM/Mb)
Metabolite levels
Platelet function and related traits
Nephrolithiasis
Obesity−related traits
29 GWAS hits omitted
HK3
ZNF346
NSD1
RGS14
DBN1
TMED9
FAM153A
UIMC1
FGFR4
RAB24
PFN3
PDLIM7
B4GALT7
LOC728554
PRELID1
F12
DOK3
LOC202181
MXD3
GRK6
FAM193B
LMAN2
PRR7
SLC34A1
DDX41
PRR7−AS1
Position on chr5 (Mb)

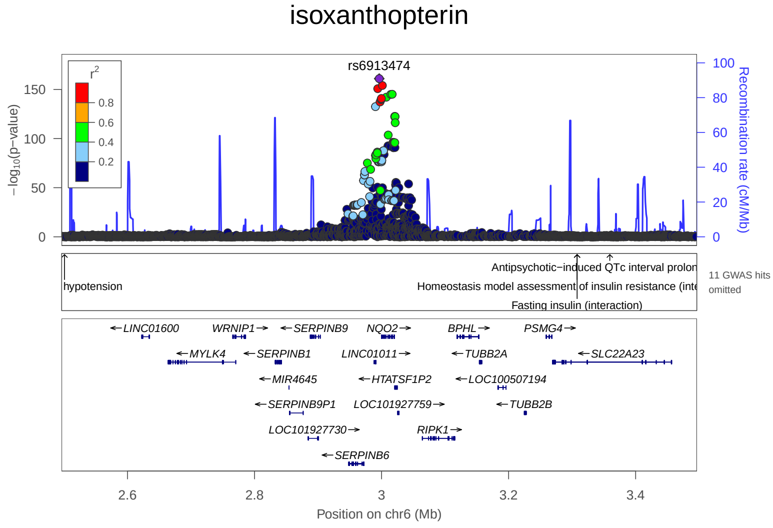
isoxanthopterin
rs6913474
−log10(p−value)
Recombination rate (cM/Mb)
hypotension
Homeostasis model assessment of insulin resistance (inte
Fasting insulin (interaction)
11 GWAS hits omitted
LINC01600
WRNIP1
SERPINB9
NQO2
BPHL
PSMG4
MYLK4
SERPINB1
LINC01011
TUBB2A
SLC22A23
MIR4645
HTATSF1P2
LOC100507194
SERPINB9P1
LOC101927759
TUBB2B
LOC101927730
RIPK1
SERPINB6
Position on chr6 (Mb)

pterin

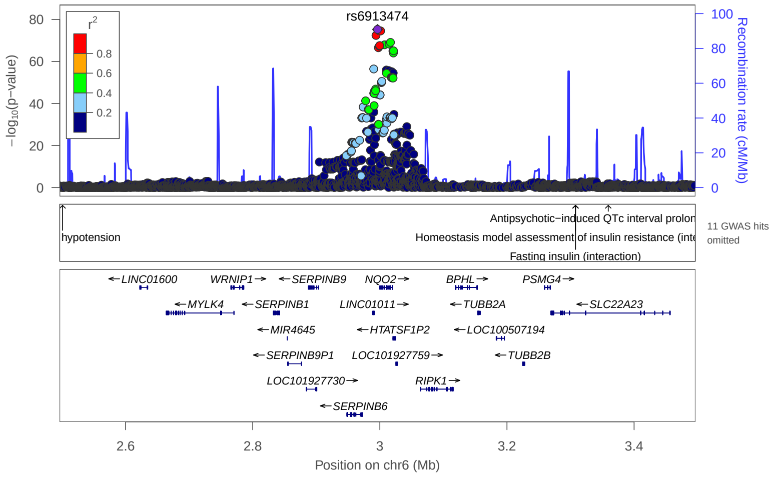

kynurenine

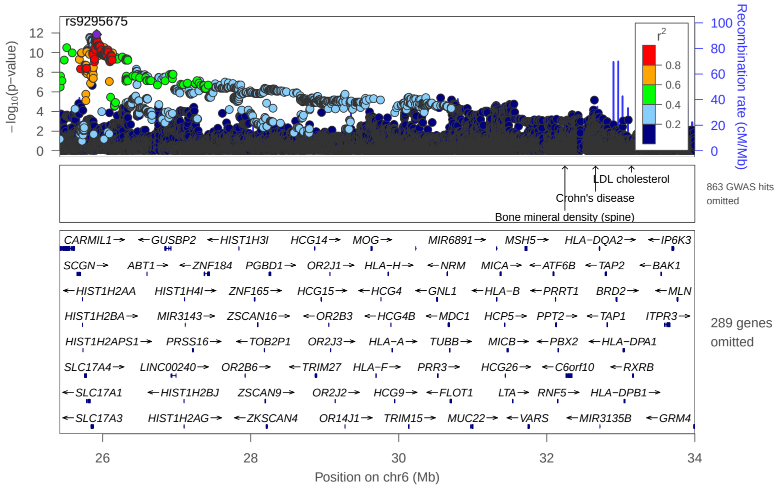

suberate (C8−DC)

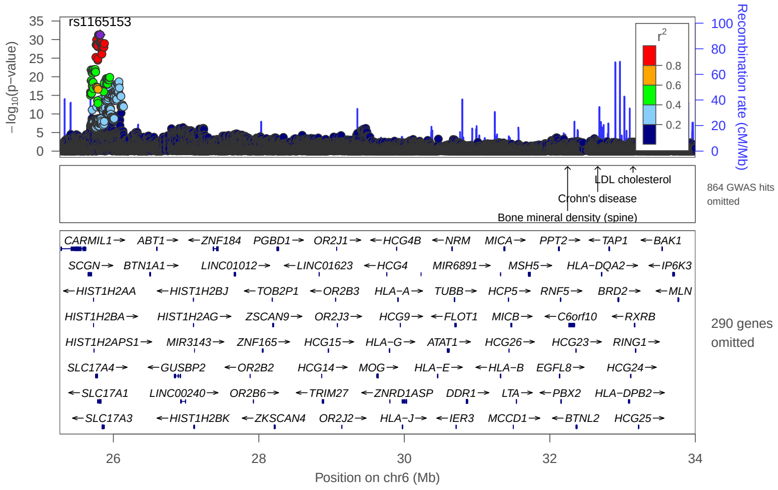

pyridoxal

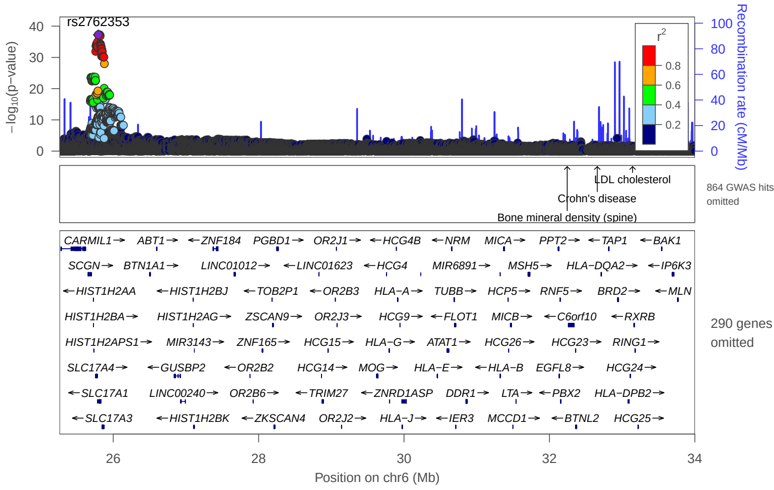

4−hydroxyphenylpyruvate

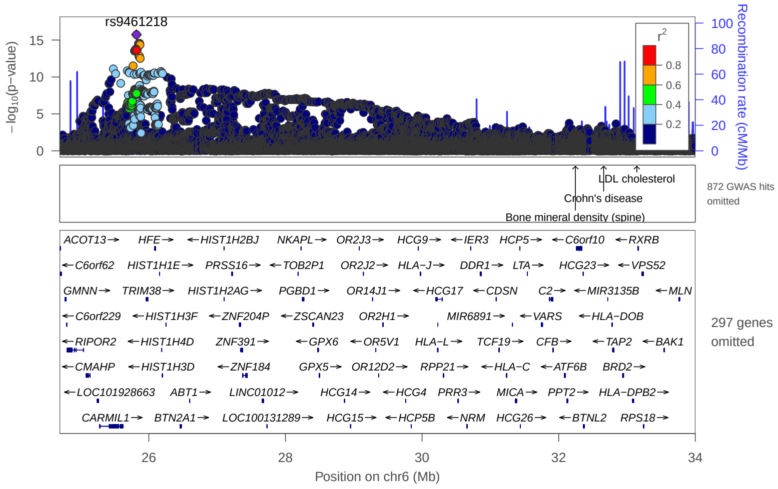

indolelactate

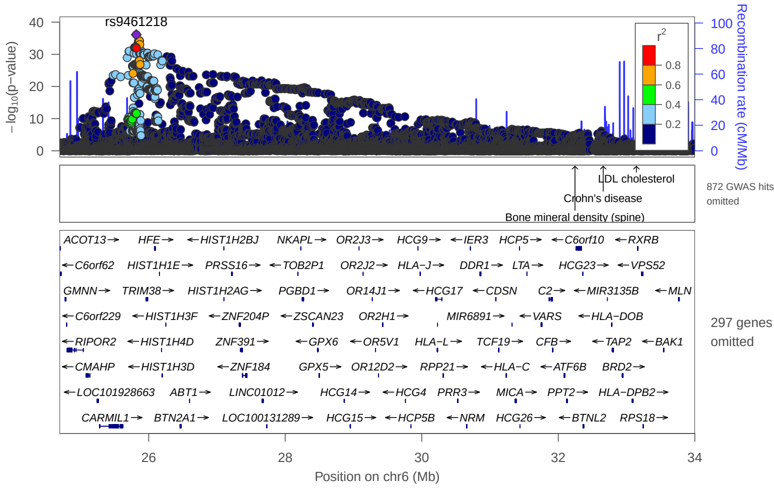

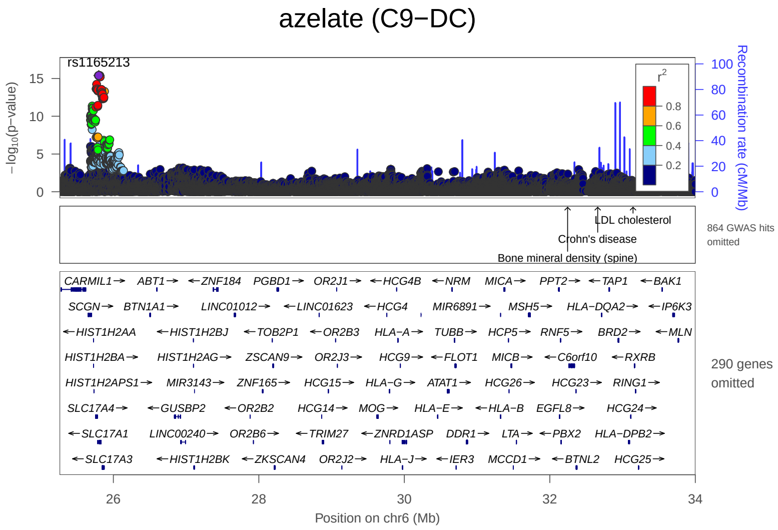
azelate (C9−DC)
rs1165213
−log10(p−value)
Recombination rate (cM/Mb)
r2
LDL cholesterol
Crohn's disease
Bone mineral density (spine)
864 GWAS hits omitted
290 genes omitted
Position on chr6 (Mb)

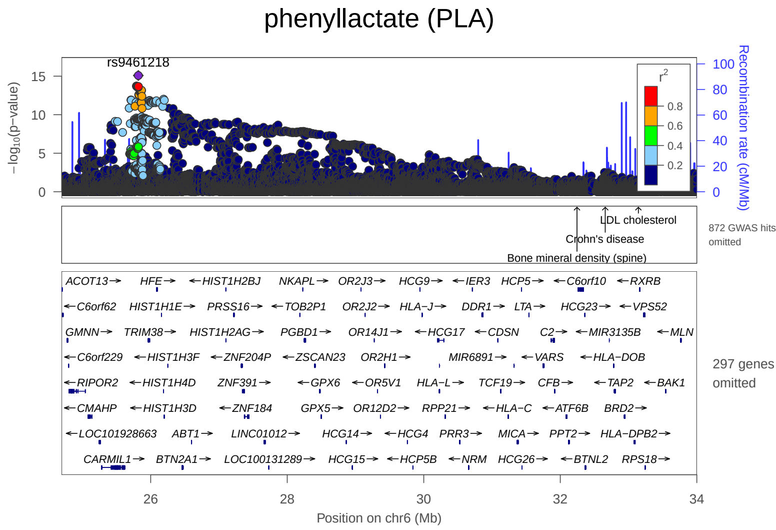
phenyllactate (PLA)
rs9461218
−log10(p−value)
Recombination rate (cM/Mb)
r2
LDL cholesterol
Crohn's disease
Bone mineral density (spine)
872 GWAS hits omitted
297 genes omitted
Position on chr6 (Mb)

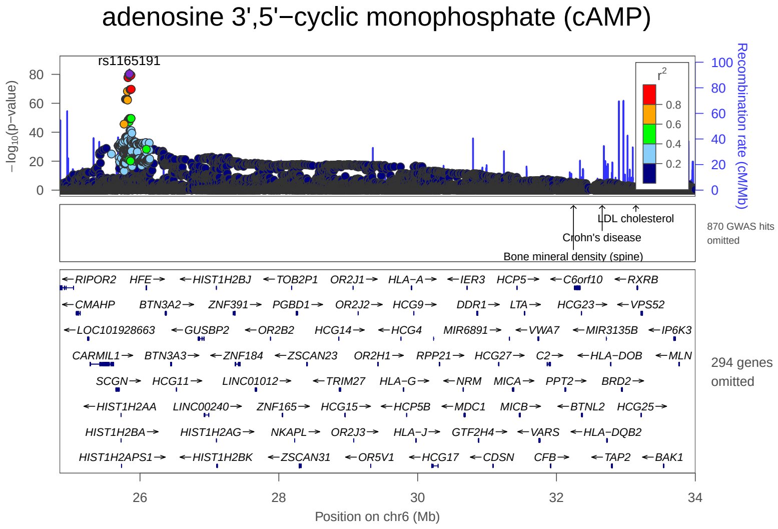
adenosine 3',5'−cyclic monophosphate (cAMP)
rs1165191
−log10(p−value)
Recombination rate (cM/Mb)
r2
LDL cholesterol
Crohn's disease
Bone mineral density (spine)
870 GWAS hits omitted
294 genes omitted
Position on chr6 (Mb)

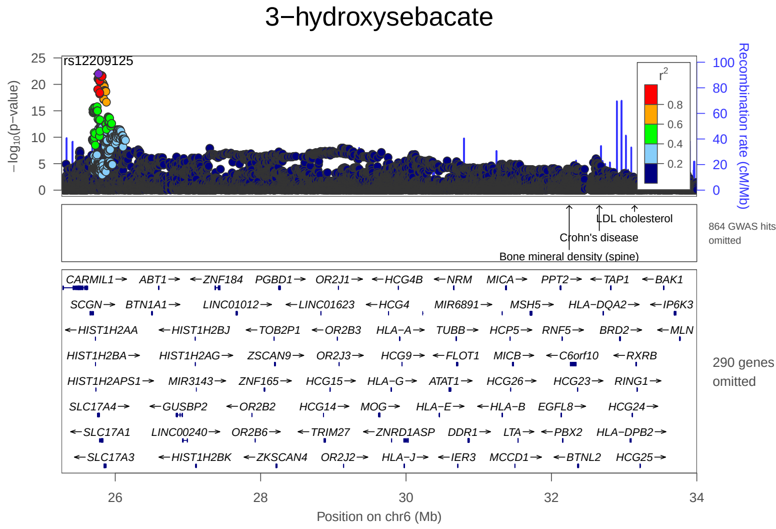
3−hydroxysebacate
rs12209125
−log10(p−value)
Recombination rate (cM/Mb)
r2
LDL cholesterol
Crohn's disease
Bone mineral density (spine)
864 GWAS hits omitted
290 genes omitted
Position on chr6 (Mb)

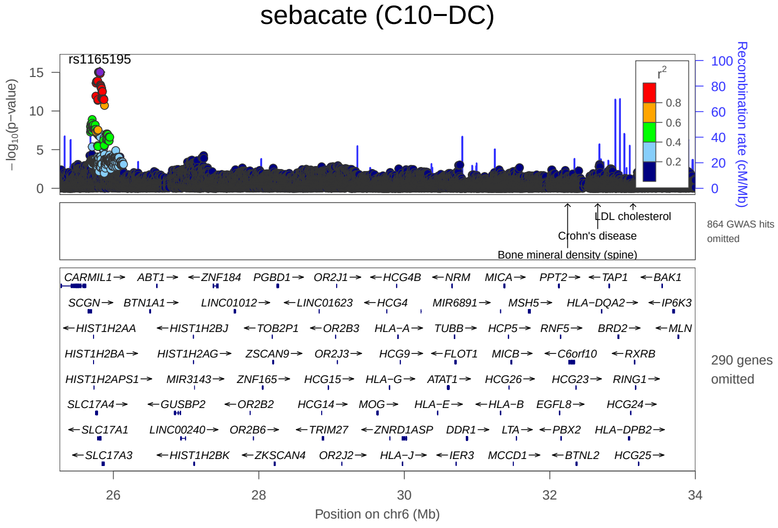
sebacate (C10−DC)
rs1165195
−log10(p−value)
Recombination rate (cM/Mb)
r2
LDL cholesterol
Crohn's disease
Bone mineral density (spine)
864 GWAS hits omitted
290 genes omitted
Position on chr6 (Mb)

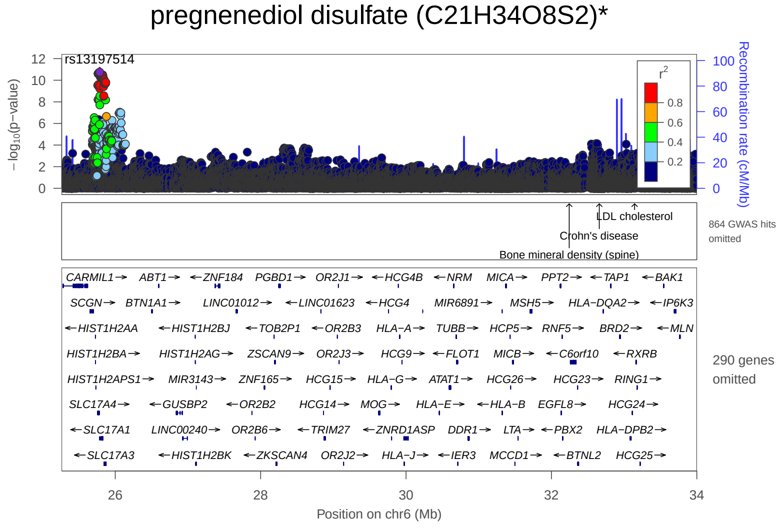
pregnenediol disulfate (C21H34O8S2)*
rs13197514
−log10(p−value)
Recombination rate (cM/Mb)
r2
LDL cholesterol
Crohn's disease
Bone mineral density (spine)
864 GWAS hits omitted
290 genes omitted
Position on chr6 (Mb)

21−hydroxypregnenolone disulfate

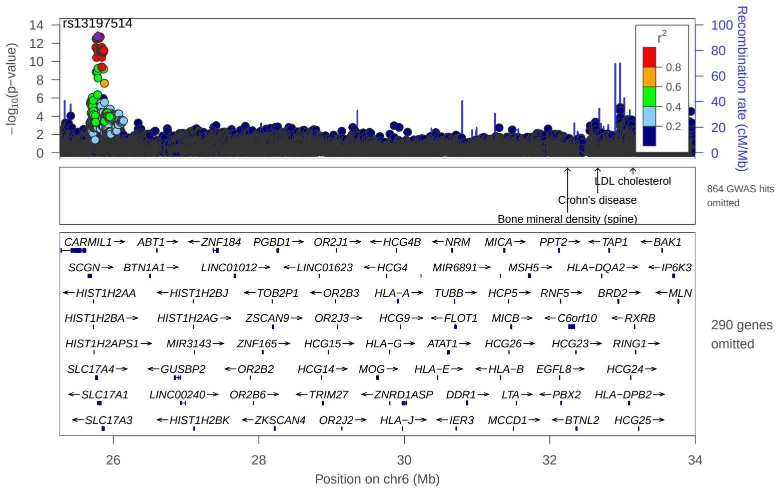

X − 13866

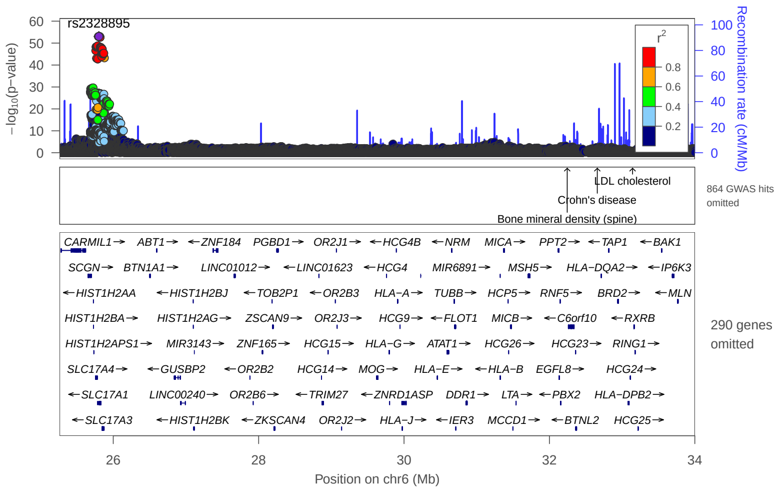

X − 11470

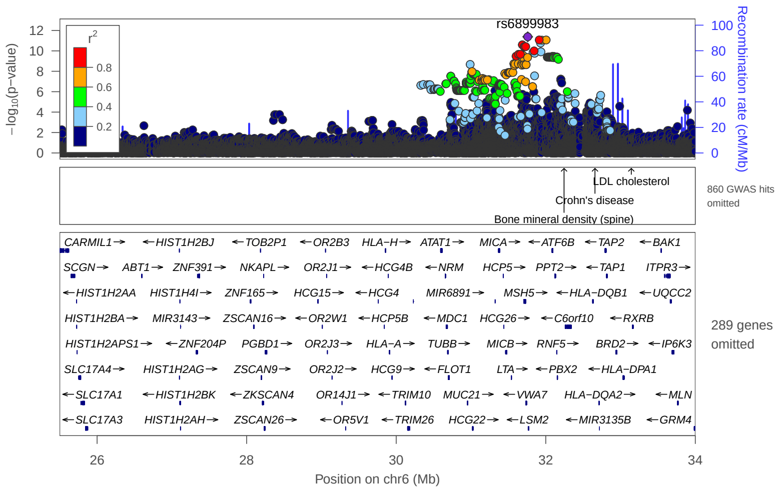

X − 12822

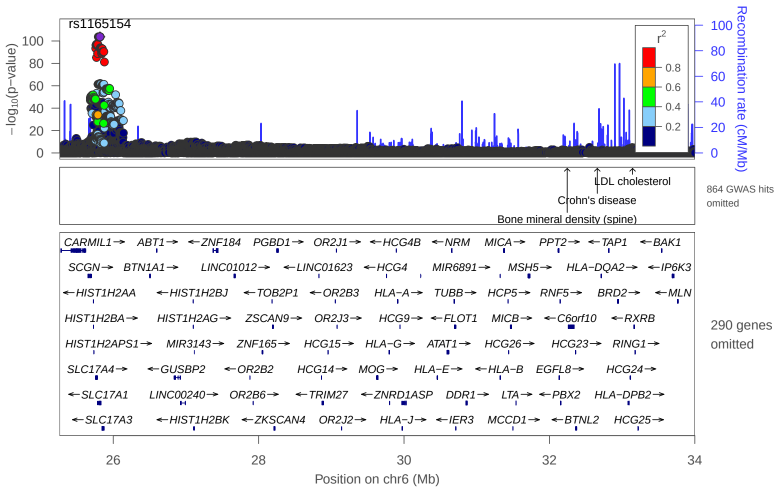

X − 21829

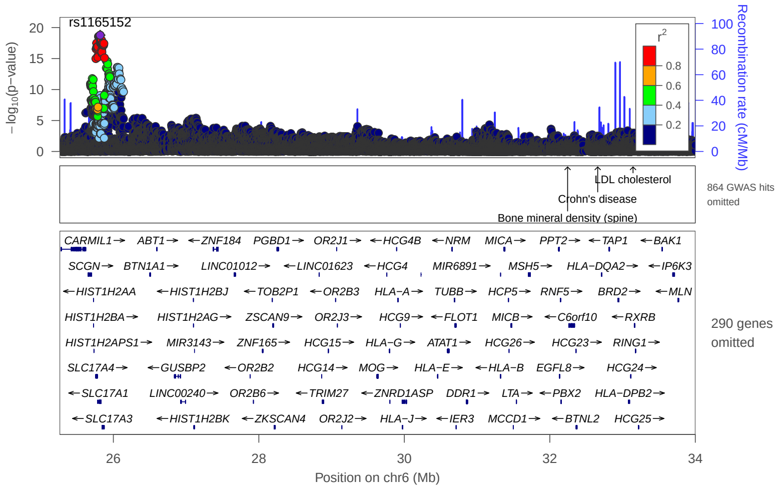

X − 12839

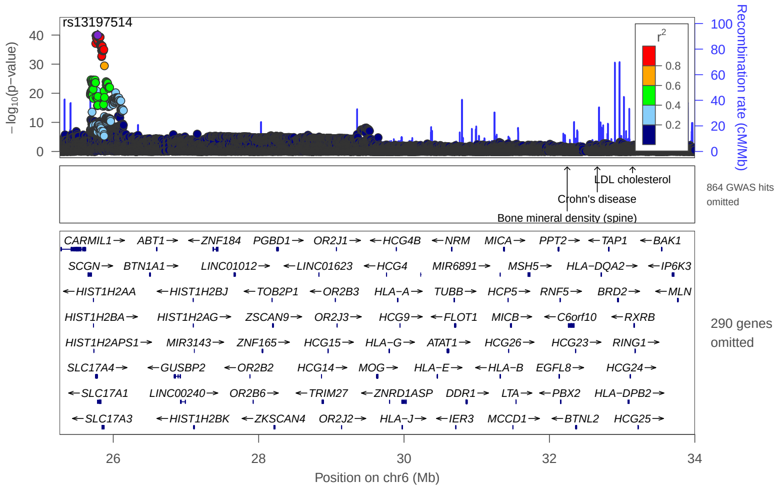

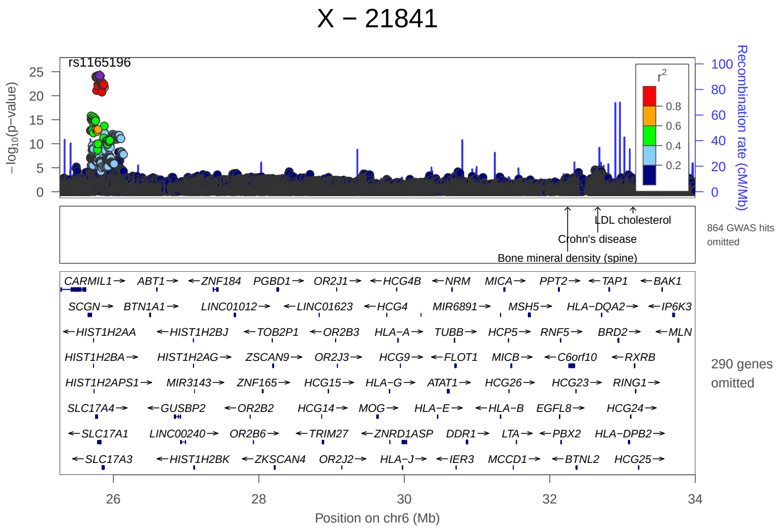
X − 21841
rs1165196
−log10(p−value)
Recombination rate (cM/Mb)
LDL cholesterol
Crohn's disease
Bone mineral density (spine)
864 GWAS hits omitted
290 genes omitted
Position on chr6 (Mb)

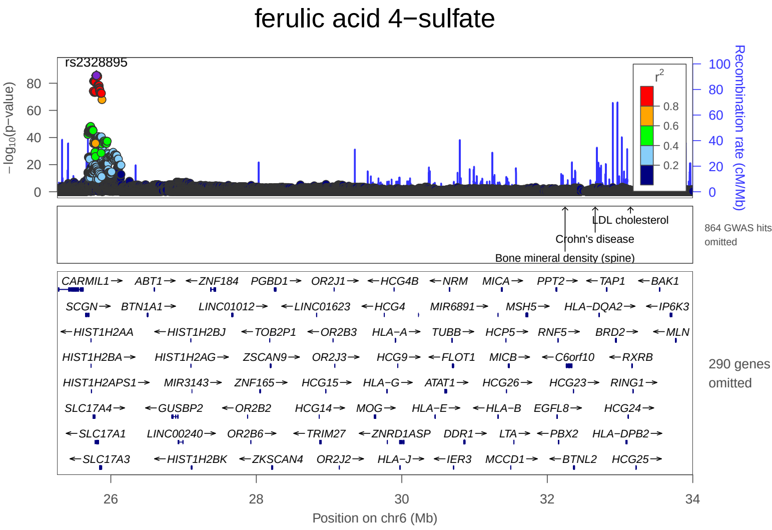
ferulic acid 4−sulfate
rs2328895
−log10(p−value)
Recombination rate (cM/Mb)
LDL cholesterol
Crohn's disease
Bone mineral density (spine)
864 GWAS hits omitted
290 genes omitted
Position on chr6 (Mb)

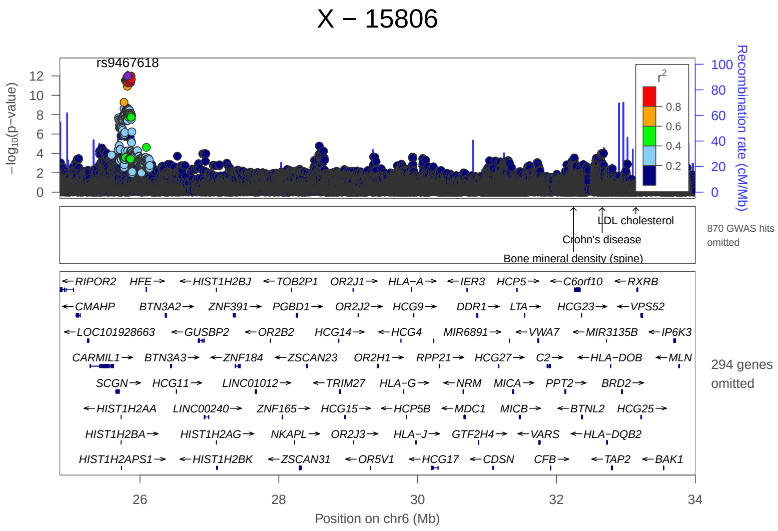
X − 15806
rs9467618
−log10(p−value)
Recombination rate (cM/Mb)
LDL cholesterol
Crohn's disease
Bone mineral density (spine)
870 GWAS hits omitted
294 genes omitted
Position on chr6 (Mb)

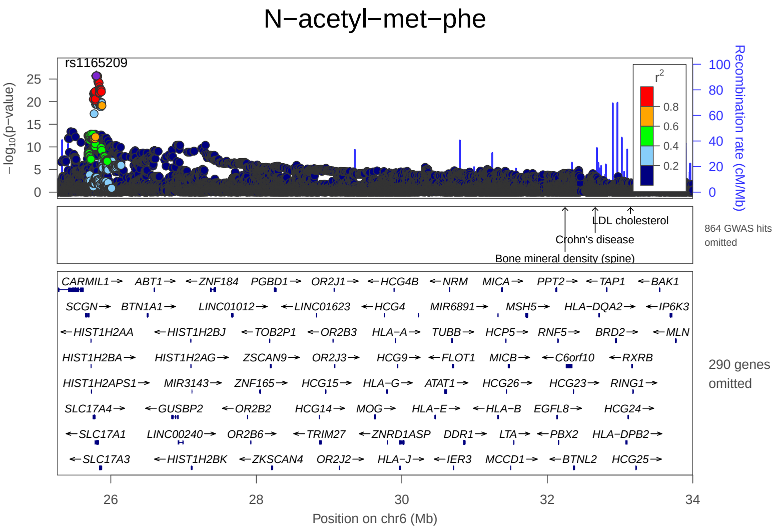
N−acetyl−met−phe
rs1165209
−log10(p−value)
Recombination rate (cM/Mb)
LDL cholesterol
Crohn's disease
Bone mineral density (spine)
864 GWAS hits omitted
290 genes omitted
Position on chr6 (Mb)

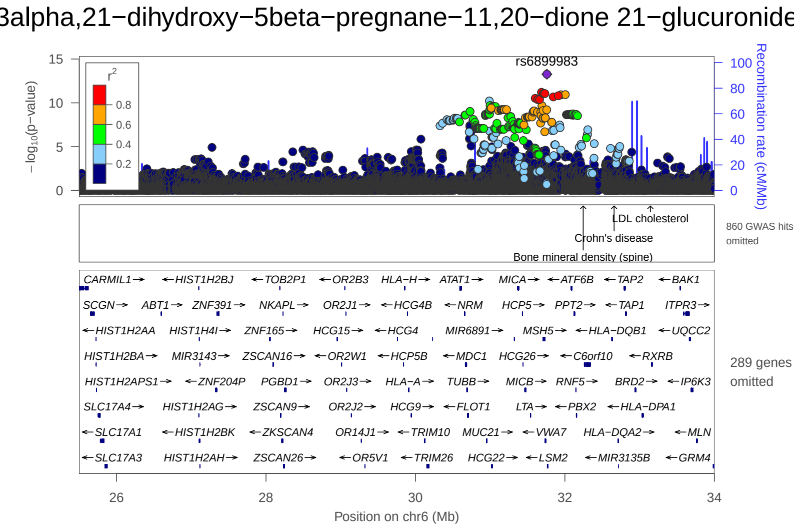
3alpha,21−dihydroxy−5beta−pregnane−11,20−dione 21−glucuronide
rs6899983
−log10(p−value)
Recombination rate (cM/Mb)
LDL cholesterol
Crohn's disease
Bone mineral density (spine)
860 GWAS hits omitted
289 genes omitted
Position on chr6 (Mb)

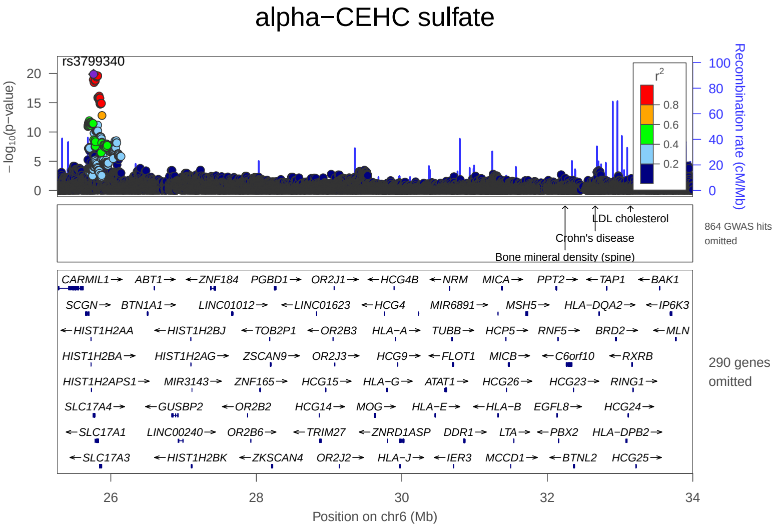
alpha−CEHC sulfate
rs3799340
−log10(p−value)
Recombination rate (cM/Mb)
LDL cholesterol
Crohn's disease
Bone mineral density (spine)
864 GWAS hits omitted
290 genes omitted
Position on chr6 (Mb)

X − 12124

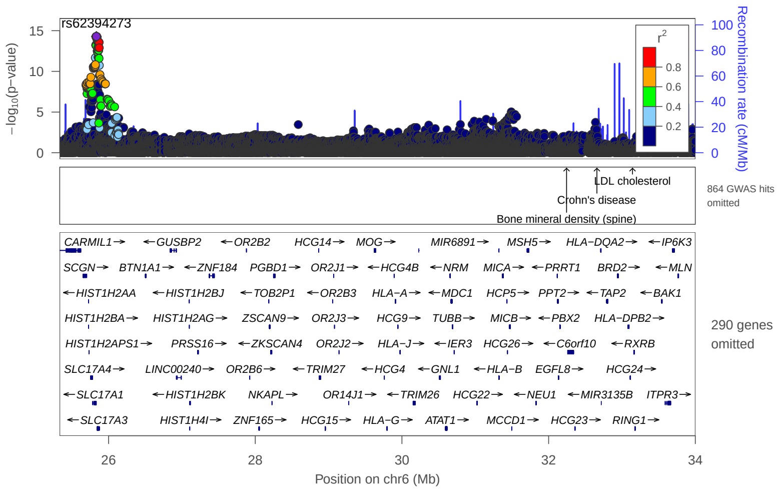

X − 13837

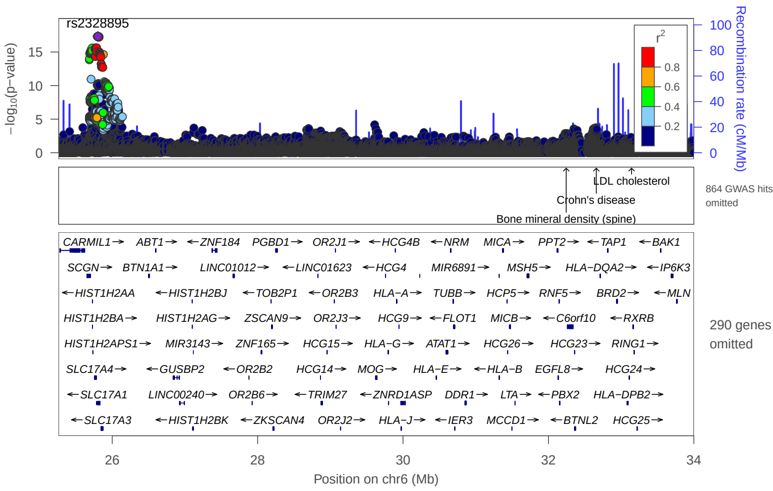

X − 12704

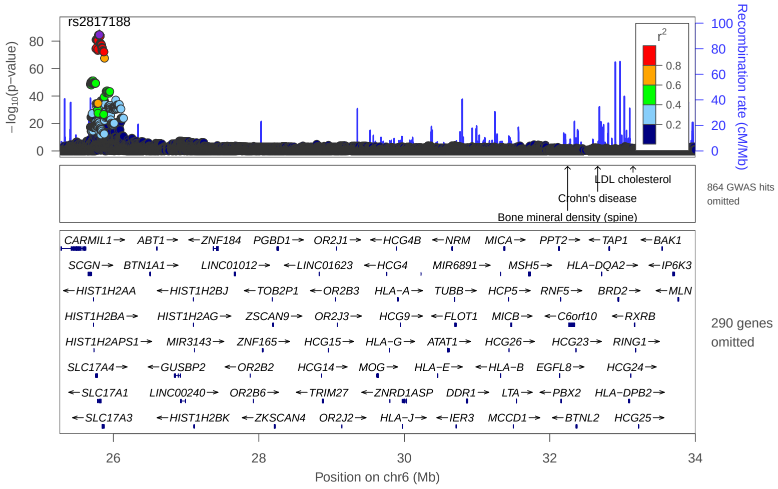

X − 13874

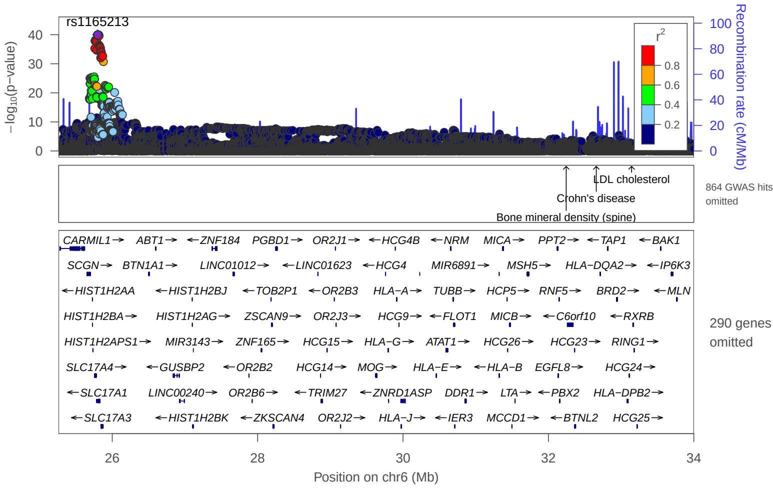

X − 18888

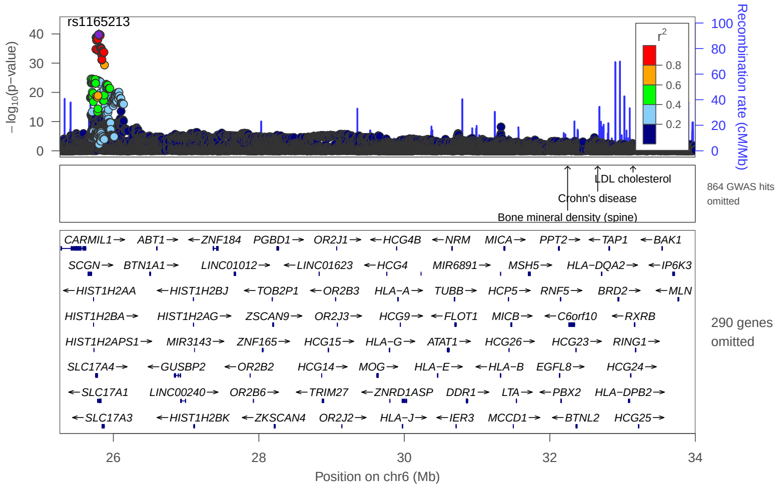

3−hydroxycinnamate sulfate

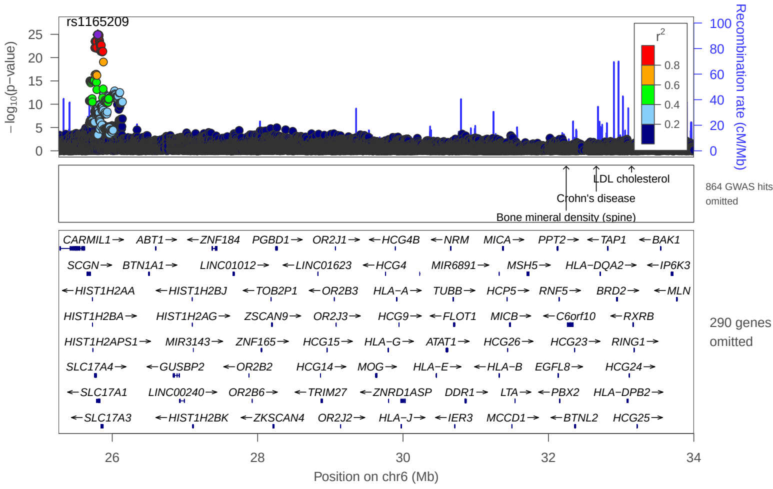

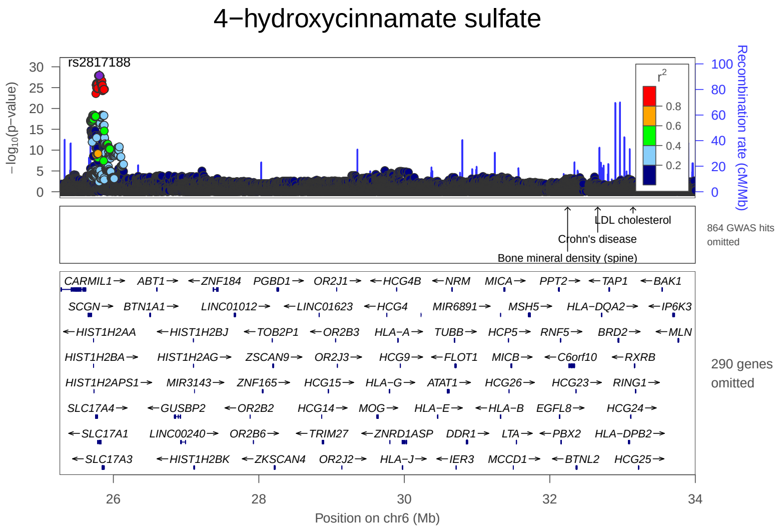
4−hydroxycinnamate sulfate
rs2817188
−log10(p−value)
Recombination rate (cM/Mb)
r2
LDL cholesterol
Crohn's disease
Bone mineral density (spine)
864 GWAS hits omitted
290 genes omitted
Position on chr6 (Mb)

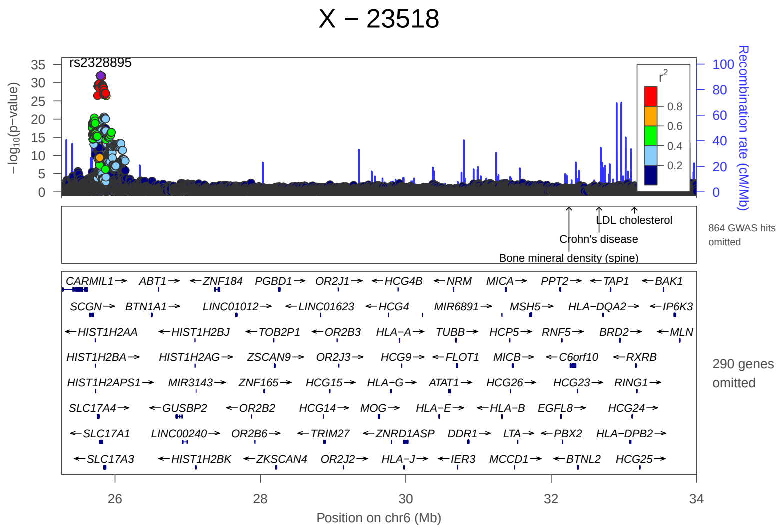
X − 23518
rs2328895
−log10(p−value)
Recombination rate (cM/Mb)
r2
LDL cholesterol
Crohn's disease
Bone mineral density (spine)
864 GWAS hits omitted
290 genes omitted
Position on chr6 (Mb)

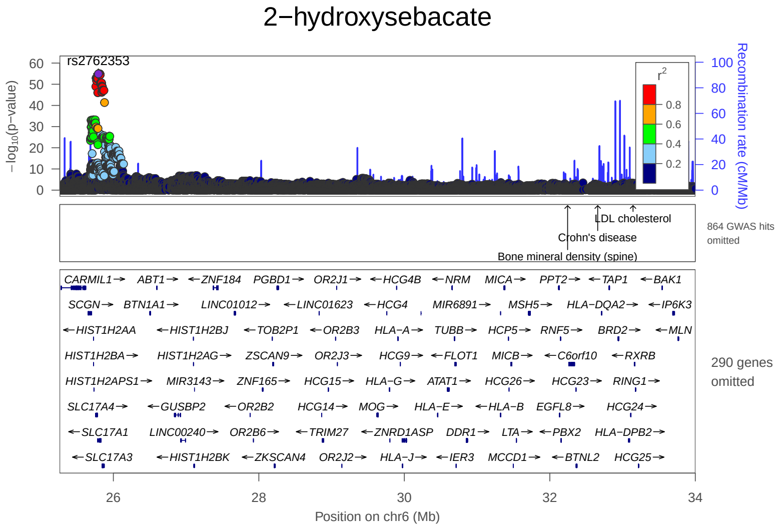
2−hydroxysebacate
rs2762353
−log10(p−value)
Recombination rate (cM/Mb)
r2
LDL cholesterol
Crohn's disease
Bone mineral density (spine)
864 GWAS hits omitted
290 genes omitted
Position on chr6 (Mb)

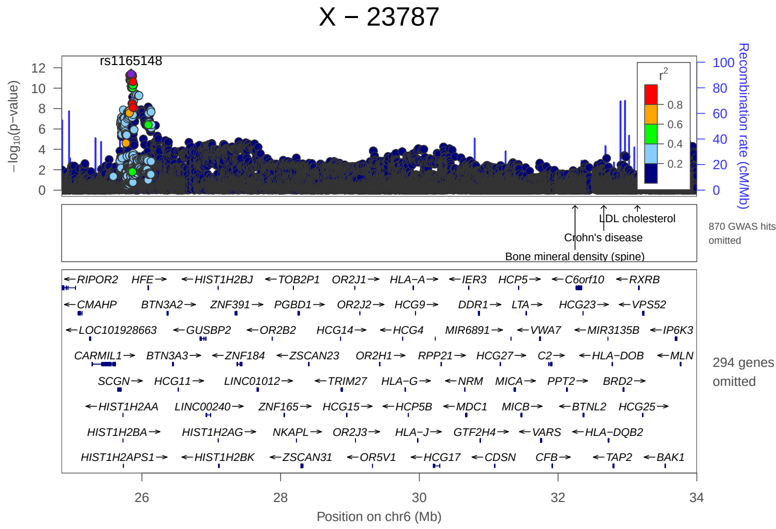
X − 23787
rs1165148
−log10(p−value)
Recombination rate (cM/Mb)
r2
LDL cholesterol
Crohn's disease
Bone mineral density (spine)
870 GWAS hits omitted
294 genes omitted
Position on chr6 (Mb)

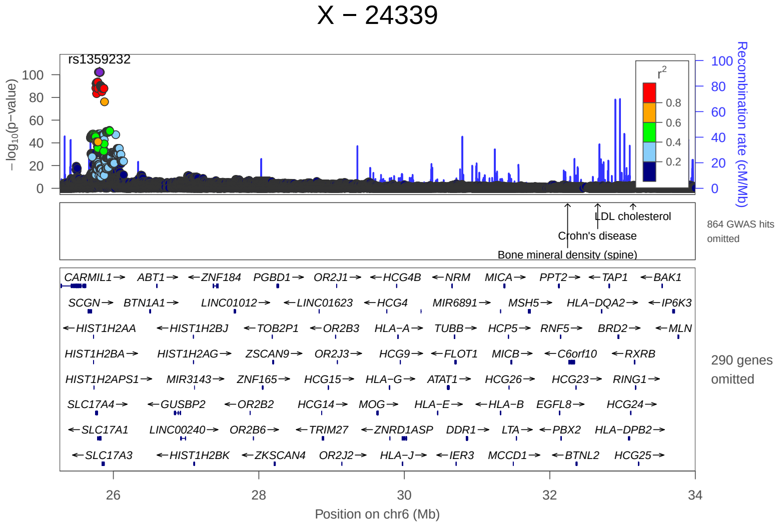
X − 24339
rs1359232
−log10(p−value)
Recombination rate (cM/Mb)
r2
LDL cholesterol
Crohn's disease
Bone mineral density (spine)
864 GWAS hits omitted
290 genes omitted
Position on chr6 (Mb)

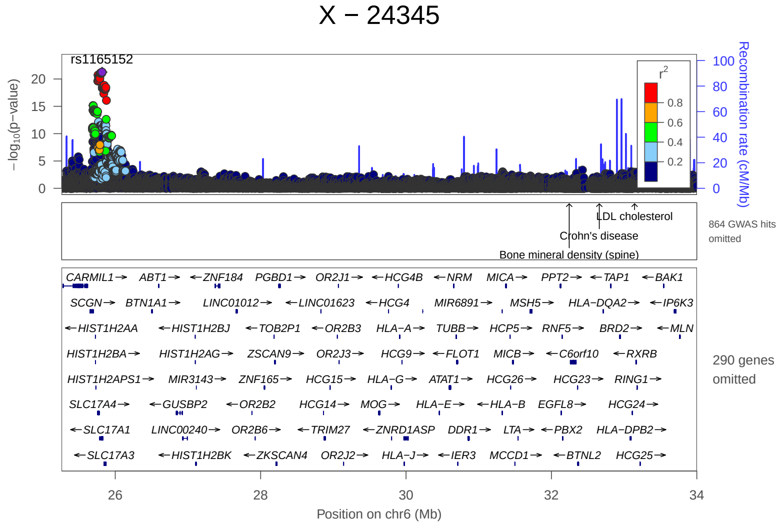
X − 24345
rs1165152
−log10(p−value)
Recombination rate (cM/Mb)
r2
LDL cholesterol
Crohn's disease
Bone mineral density (spine)
864 GWAS hits omitted
290 genes omitted
Position on chr6 (Mb)

glutamine conjugate of C9H16O2 (1)*

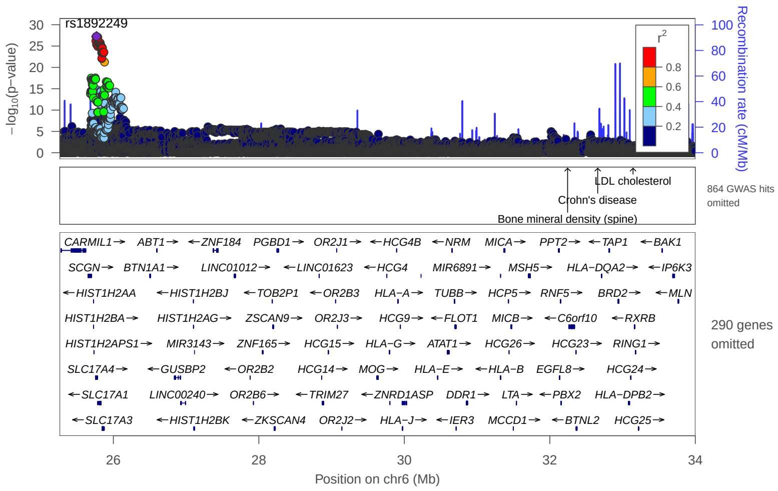

glutamine conjugate of C10H16O2 (2)*

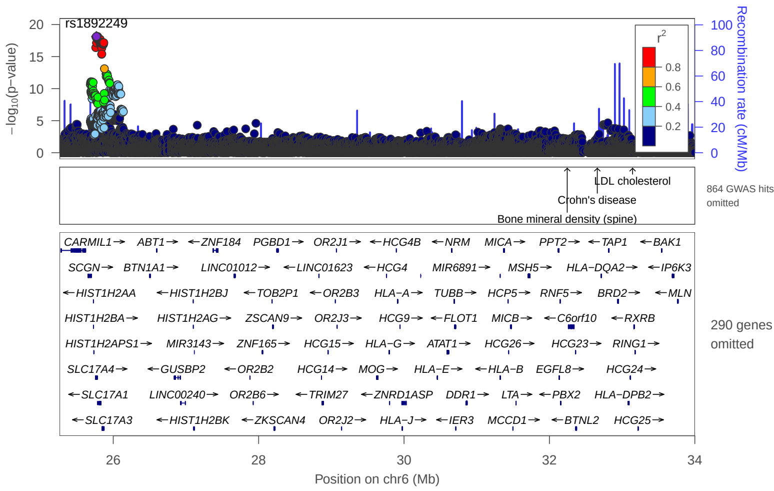

phenylacetylglutamate

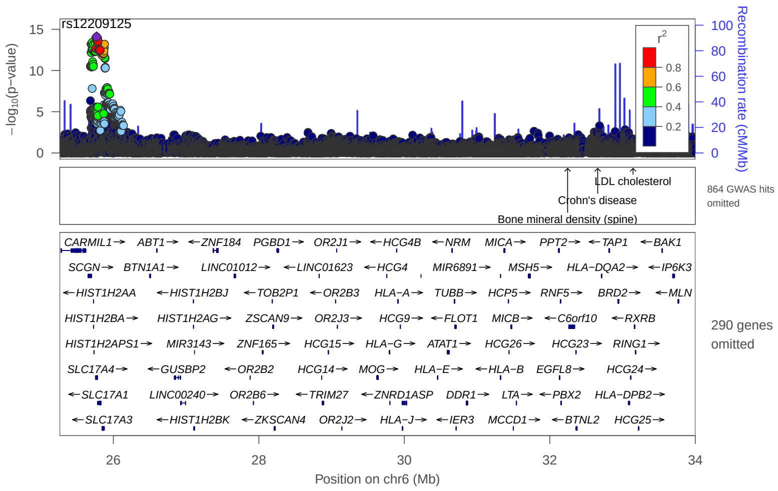

suberoylcarnitine (C8−DC)

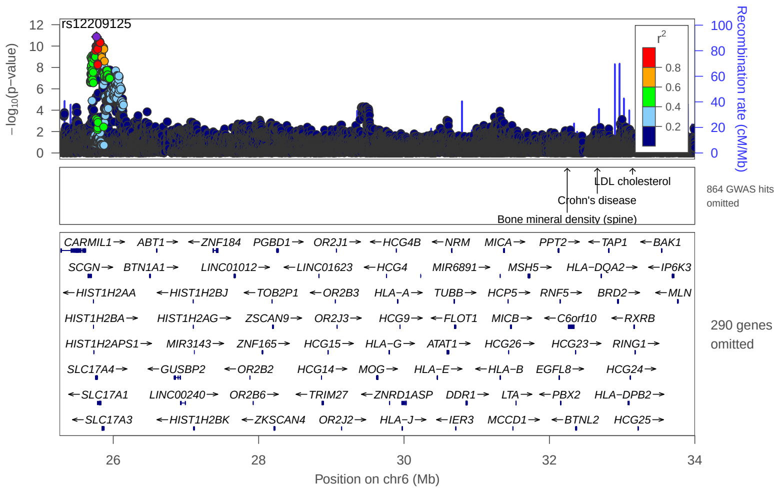

hexanoylglutamine

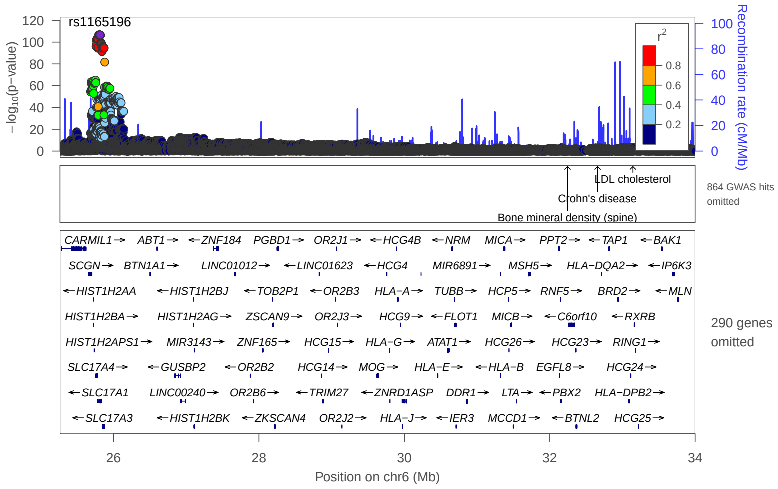

heptanoylglutamine

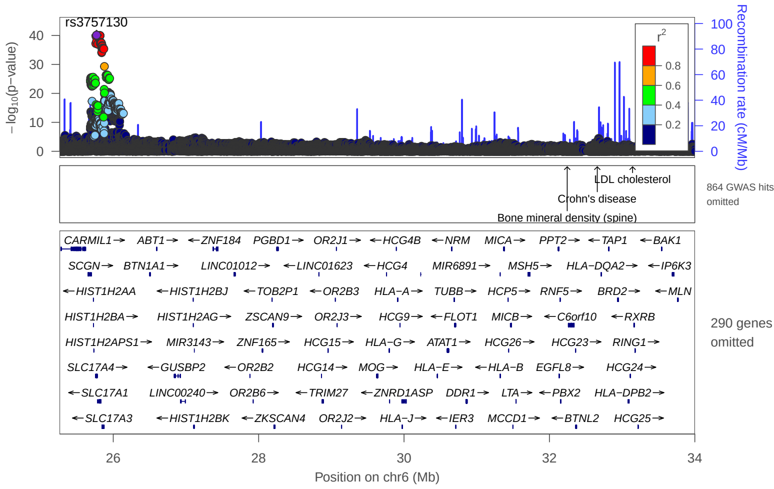

N−octanoylglutamine

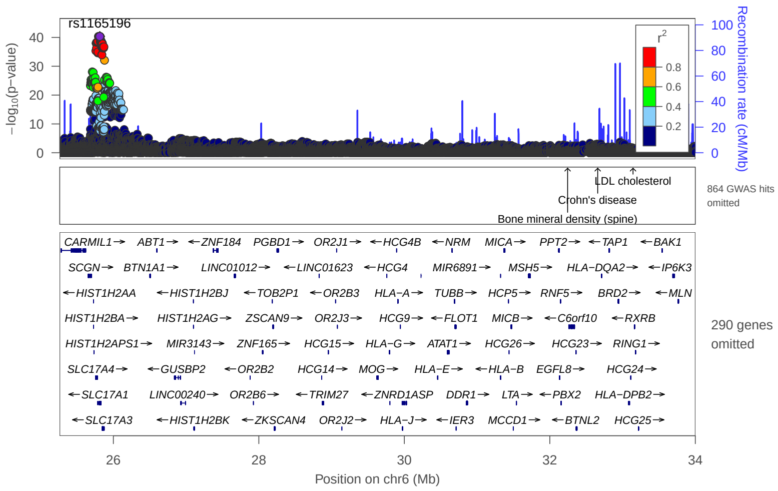

adenosine

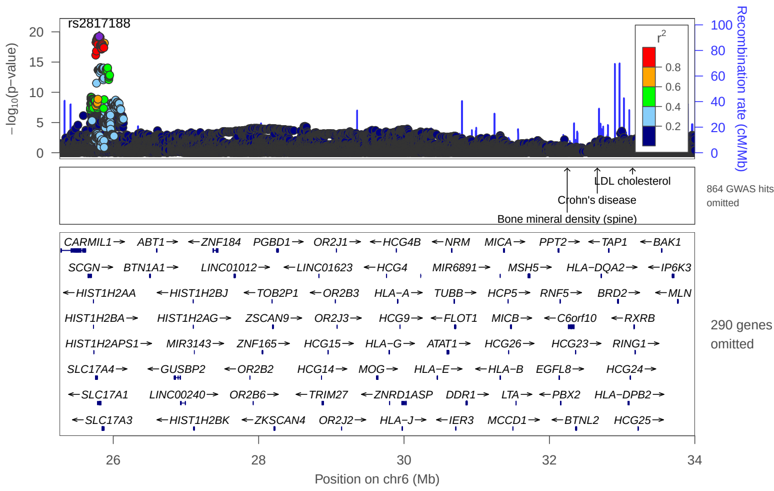

isocaproylglutamine

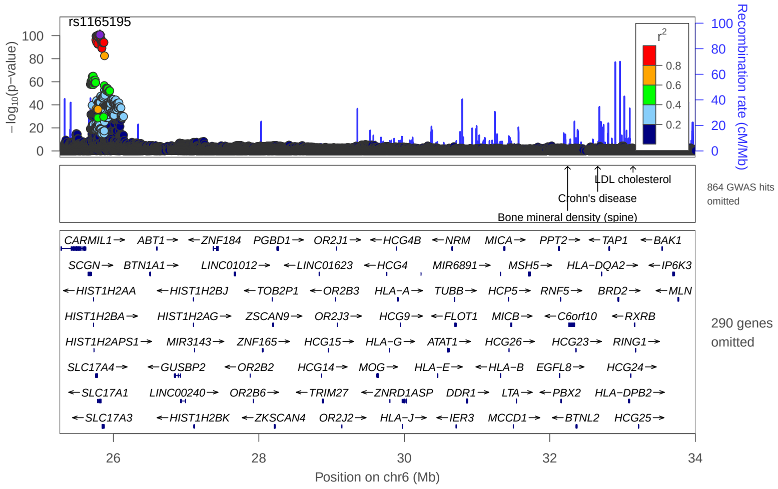

glutamine conjugate of C8H12O4 (1)*

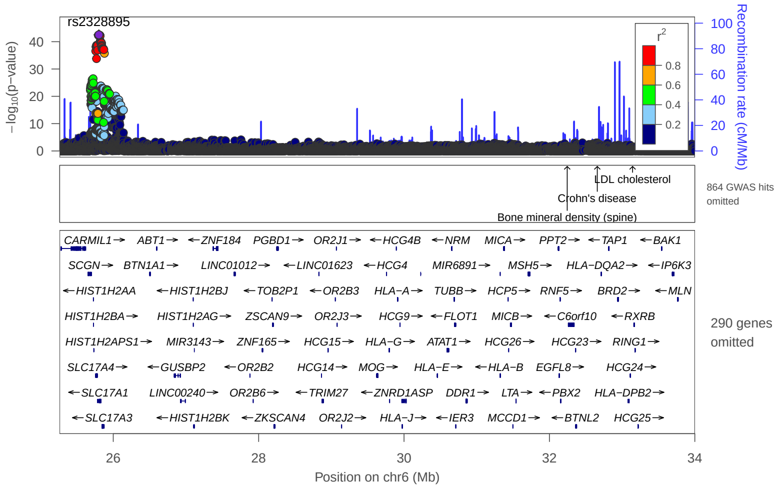

glutamine conjugate of C8H12O2 (3)*

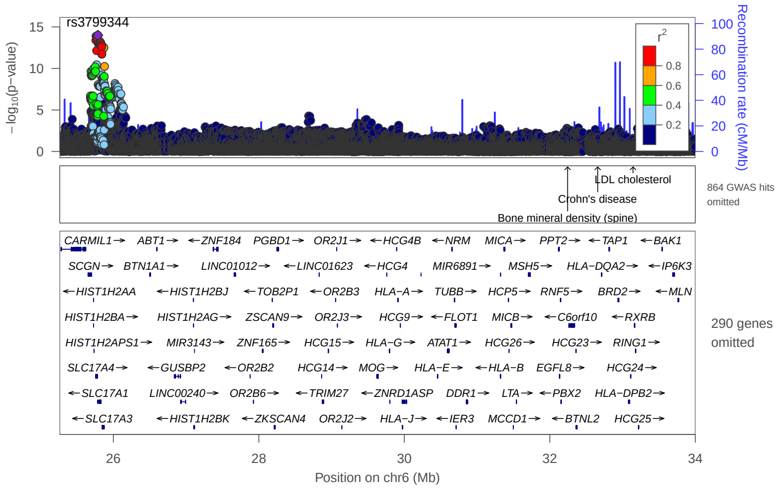

gamma−CEHC sulfate

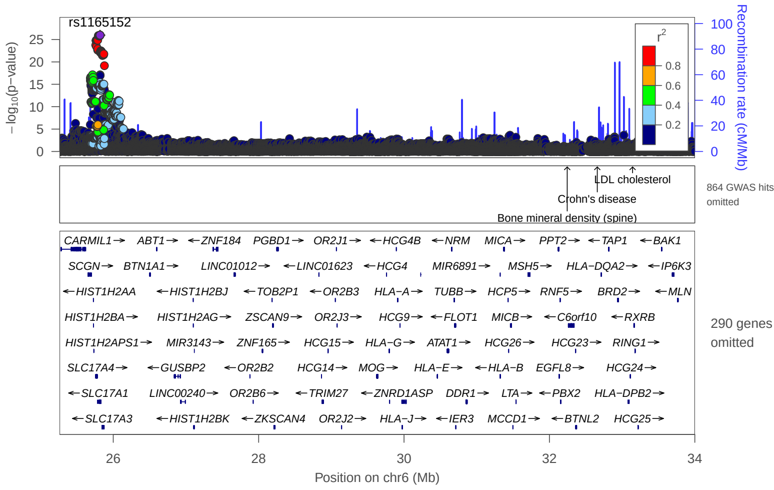

N−lactoyl leucine

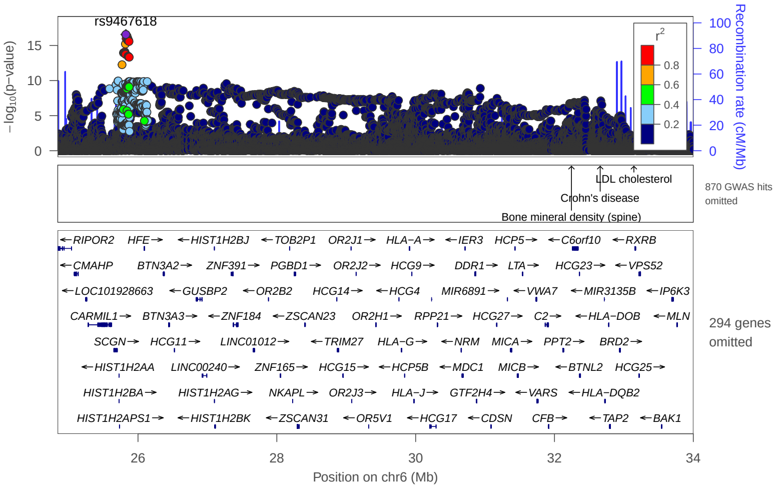

N−lactoyl valine

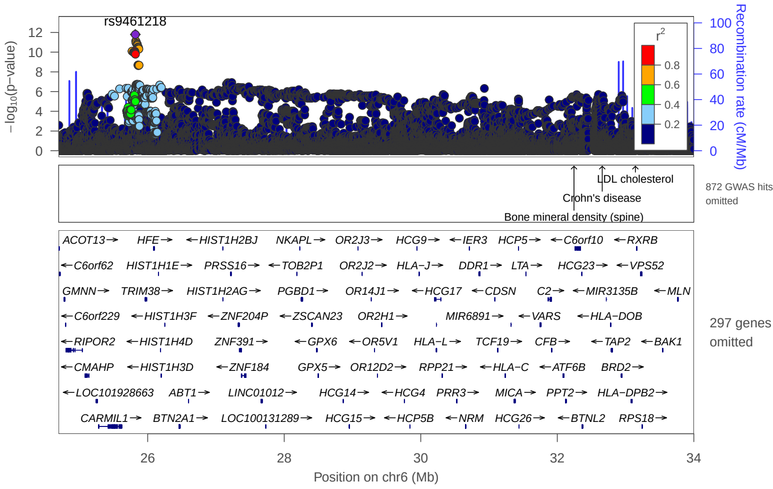

N−lactoyl tyrosine

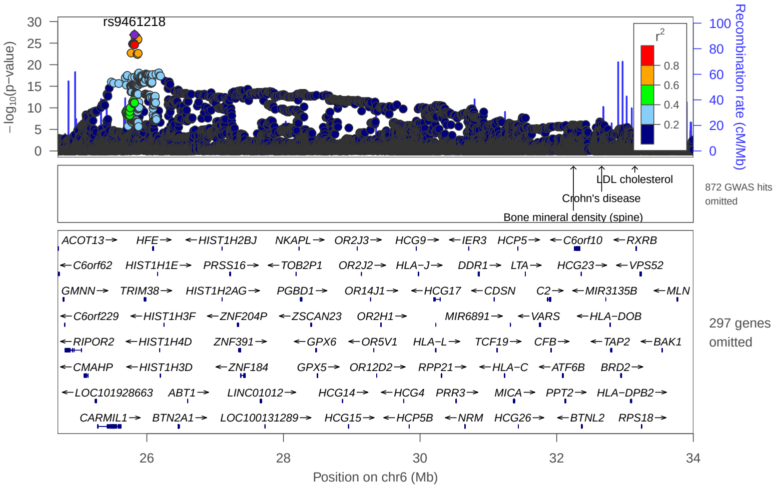

N−lactoyl phenylalanine

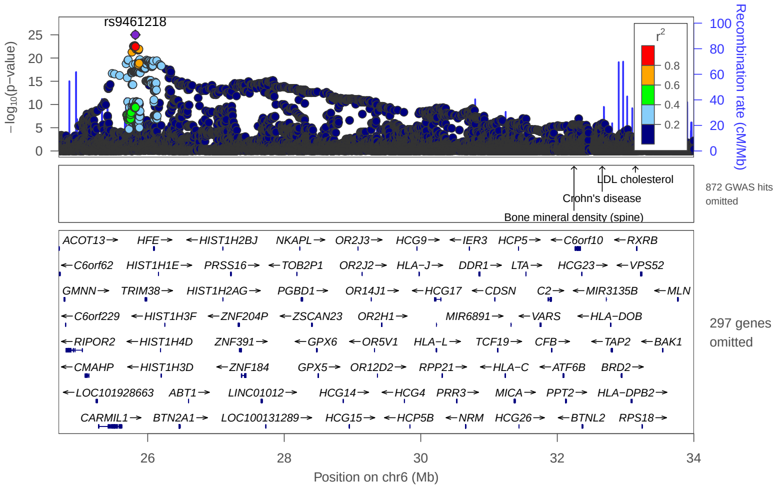

4−methylhexanoylglutamine

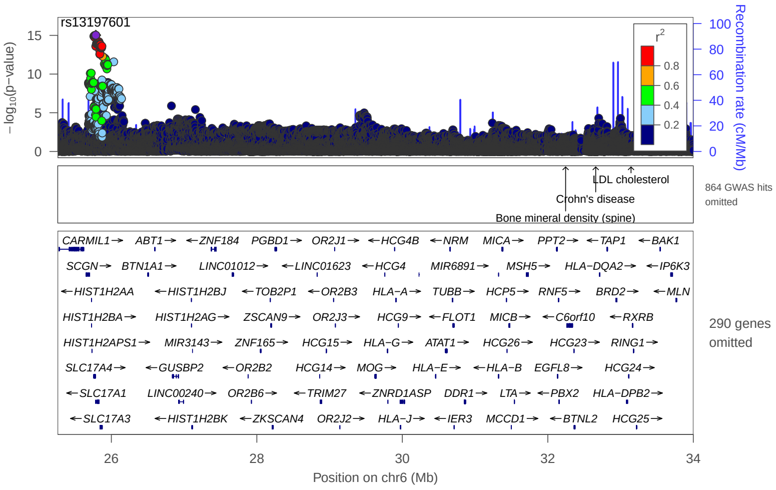

cyclo(leu−asp)*

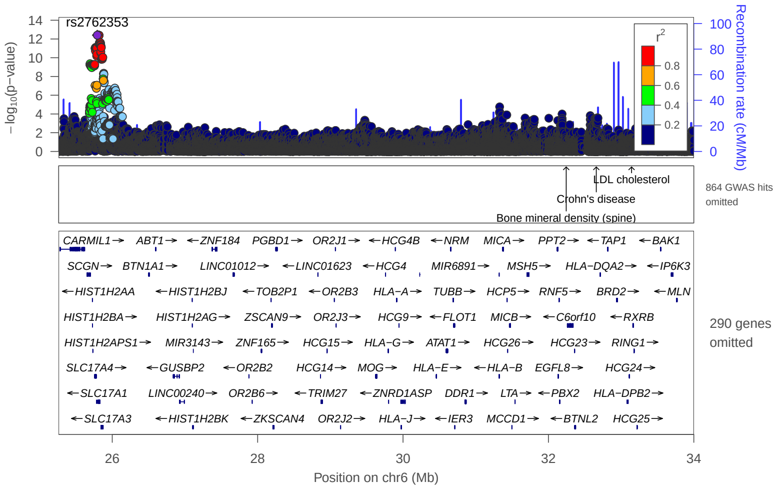

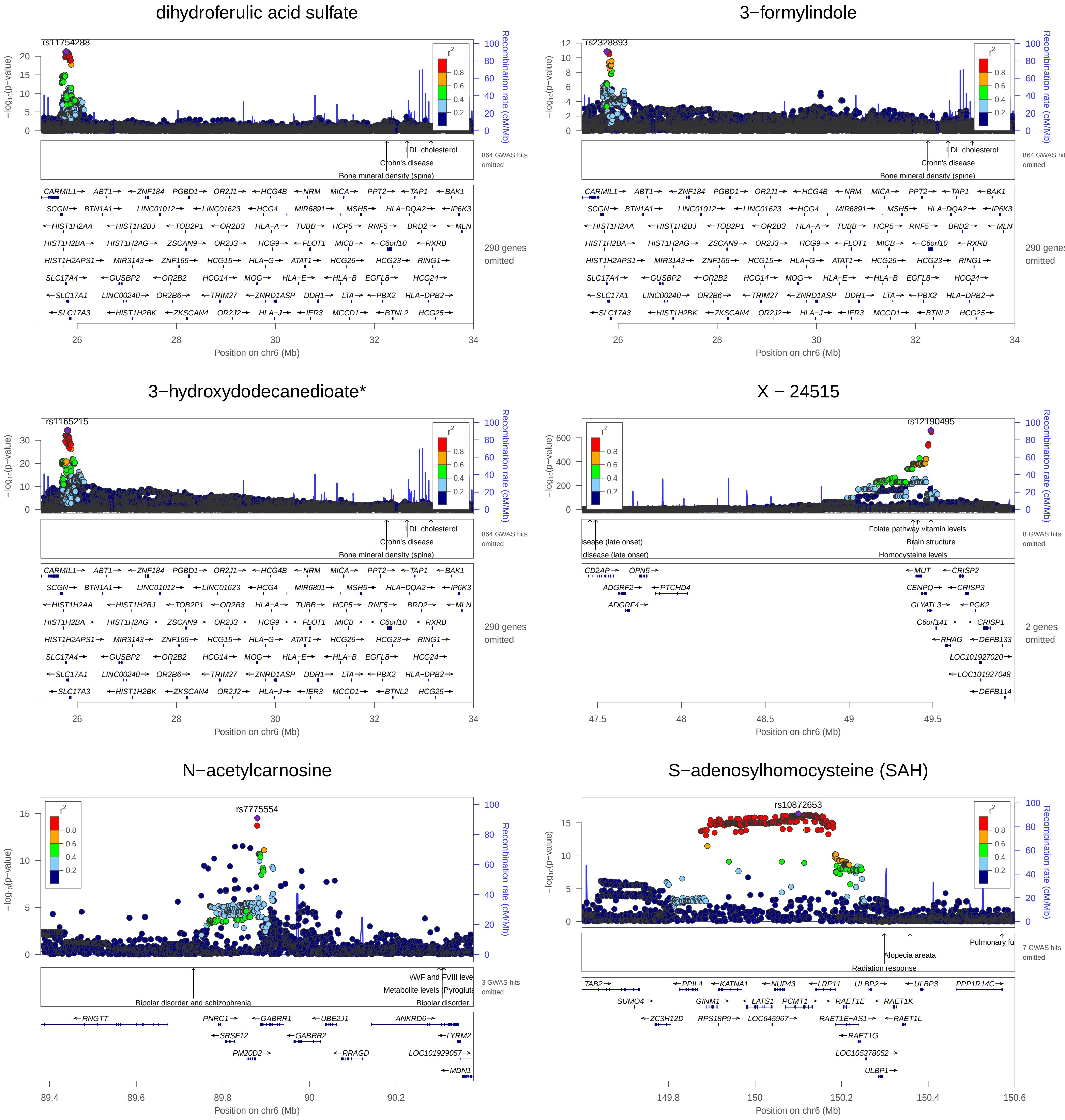

dihydroferulic acid sulfate
rs11754288
3−formylindole
rs2328893
3−hydroxydodecanedioate*
rs1165215
X − 24515
rs12190495
N−acetylcarnosine
rs7775554
S−adenosylhomocysteine (SAH)
rs10872653
−log10(p−value)
Recombination rate (cM/Mb)
r2
LDL cholesterol
Crohn's disease
Bone mineral density (spine)
864 GWAS hits omitted
290 genes omitted
Folate pathway vitamin levels
Brain structure
Homocysteine levels
8 GWAS hits omitted
2 genes omitted
vWF and FVIII leve
Metabolite levels (Pyrogluta
Bipolar disorder and schizophrenia
Bipolar disorder
3 GWAS hits omitted
Pulmonary fu
Alopecia areata
Radiation response
7 GWAS hits omitted
CARMIL1 ABT1 ZNF184 PGBD1 OR2J1 HCG4B NRM MICA PPT2 TAP1 BAK1
SCGN BTN1A1 LINC01012 LINC01623 HCG4 MIR6891 MSH5 HLA−DQA2 IP6K3
HIST1H2AA HIST1H2BJ TOB2P1 OR2B3 HLA−A TUBB HCP5 RNF5 BRD2 MLN
HIST1H2BA HIST1H2AG ZSCAN9 OR2J3 HCG9 FLOT1 MICB C6orf10 RXRB
HIST1H2APS1 MIR3143 ZNF165 HCG15 HLA−G ATAT1 HCG26 HCG23 RING1
SLC17A4 GUSBP2 OR2B2 HCG14 MOG HLA−E HLA−B EGFL8 HCG24
SLC17A1 LINC00240 OR2B6 TRIM27 ZNRD1ASP DDR1 LTA PBX2 HLA−DPB2
SLC17A3 HIST1H2BK ZKSCAN4 OR2J2 HLA−J IER3 MCCD1 BTNL2 HCG25
CD2AP OPN5 ADGRF2 PTCHD4 ADGRF4
MUT CRISP2 CENPQ CRISP3 GLYATL3 PGK2 C6orf141 CRISP1 RHAG DEFB133 LOC101927020 LOC101927048 DEFB114
RNGTT PNRC1 GABRR1 UBE2J1 ANKRD6 SRSF12 GABRR2 LYRM2 PM20D2 RRAGD LOC101929057 MDN1
TAB2 PPIL4 KATNA1 NUP43 LRP11 ULBP2 ULBP3 PPP1R14C SUMO4 GINM1 LATS1 PCMT1 RAET1E RAET1K ZC3H12D RPS18P9 LOC645967 RAET1E−AS1 RAET1L RAET1G LOC105378052 ULBP1
Position on chr6 (Mb)

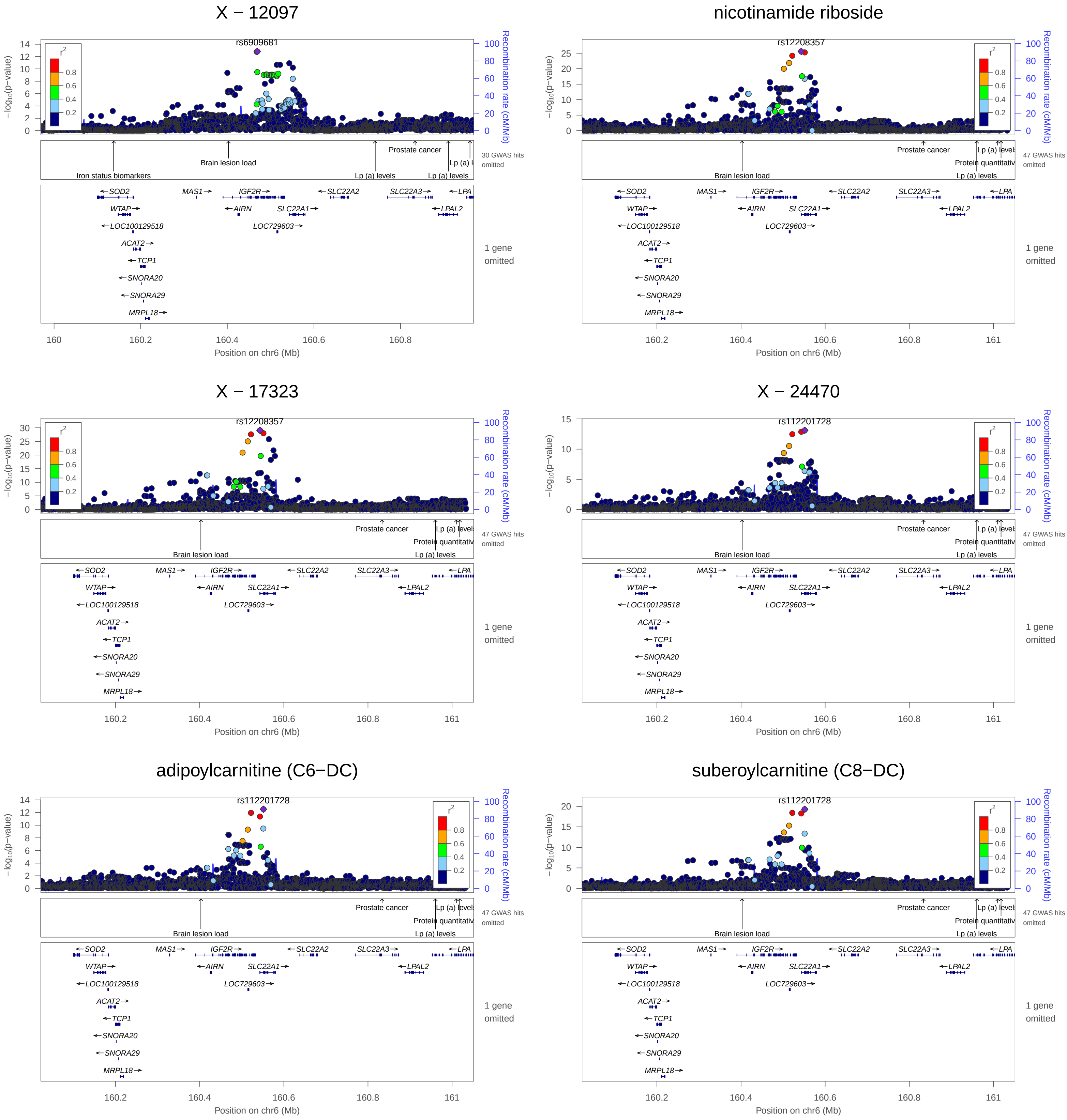
X − 12097
nicotinamide riboside
X − 17323
X − 24470
adipoylcarnitine (C6−DC)
suberoylcarnitine (C8−DC)
rs6909681
rs12208357
rs112201728
−log10(p−value)
Recombination rate (cM/Mb)
r2
Iron status biomarkers
Brain lesion load
Prostate cancer
Lp (a) levels
Protein quantitativ
30 GWAS hits omitted
47 GWAS hits omitted
SOD2
WTAP
LOC100129518
ACAT2
TCP1
SNORA20
SNORA29
MRPL18
MAS1
IGF2R
AIRN
SLC22A1
LOC729603
SLC22A2
SLC22A3
LPAL2
LPA
1 gene omitted
Position on chr6 (Mb)

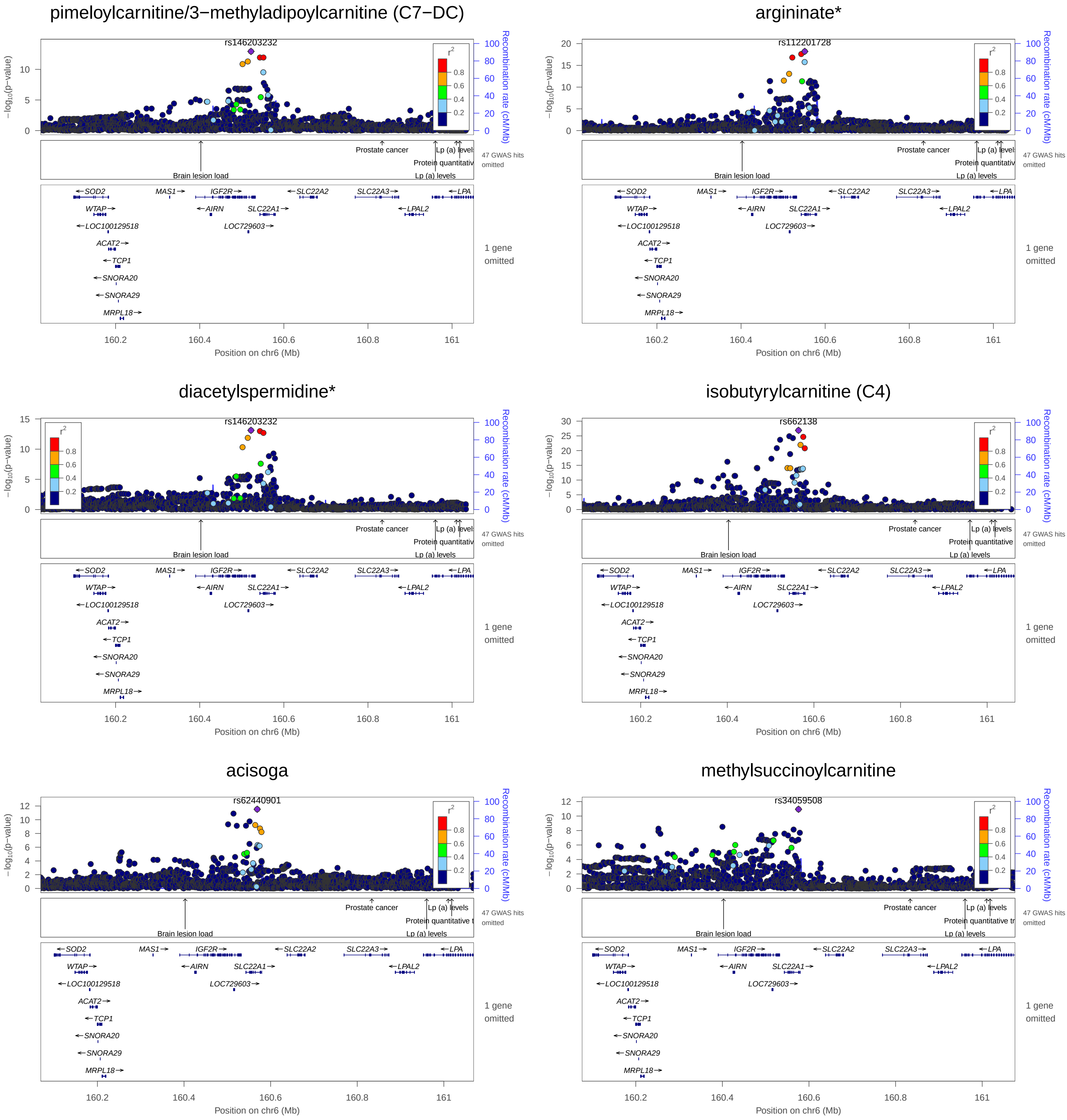
pimeloylcarnitine/3−methyladipoylcarnitine (C7−DC)
rs146203232
argininate*
rs112201728
diacetylspermidine*
rs146203232
isobutyrylcarnitine (C4)
rs662138
acisoga
rs62440901
methylsuccinoylcarnitine
rs34059508
−log10(p−value)
Recombination rate (cM/Mb)
r2
0.8
0.6
0.4
0.2
Prostate cancer
Lp (a) levels
Protein quantitative
Brain lesion load
47 GWAS hits omitted
SOD2
WTAP
LOC100129518
ACAT2
TCP1
SNORA20
SNORA29
MRPL18
MAS1
IGF2R
AIRN
LOC729603
SLC22A2
SLC22A1
SLC22A3
LPAL2
LPA
1 gene omitted
160.2
160.4
160.6
160.8
161
Position on chr6 (Mb)

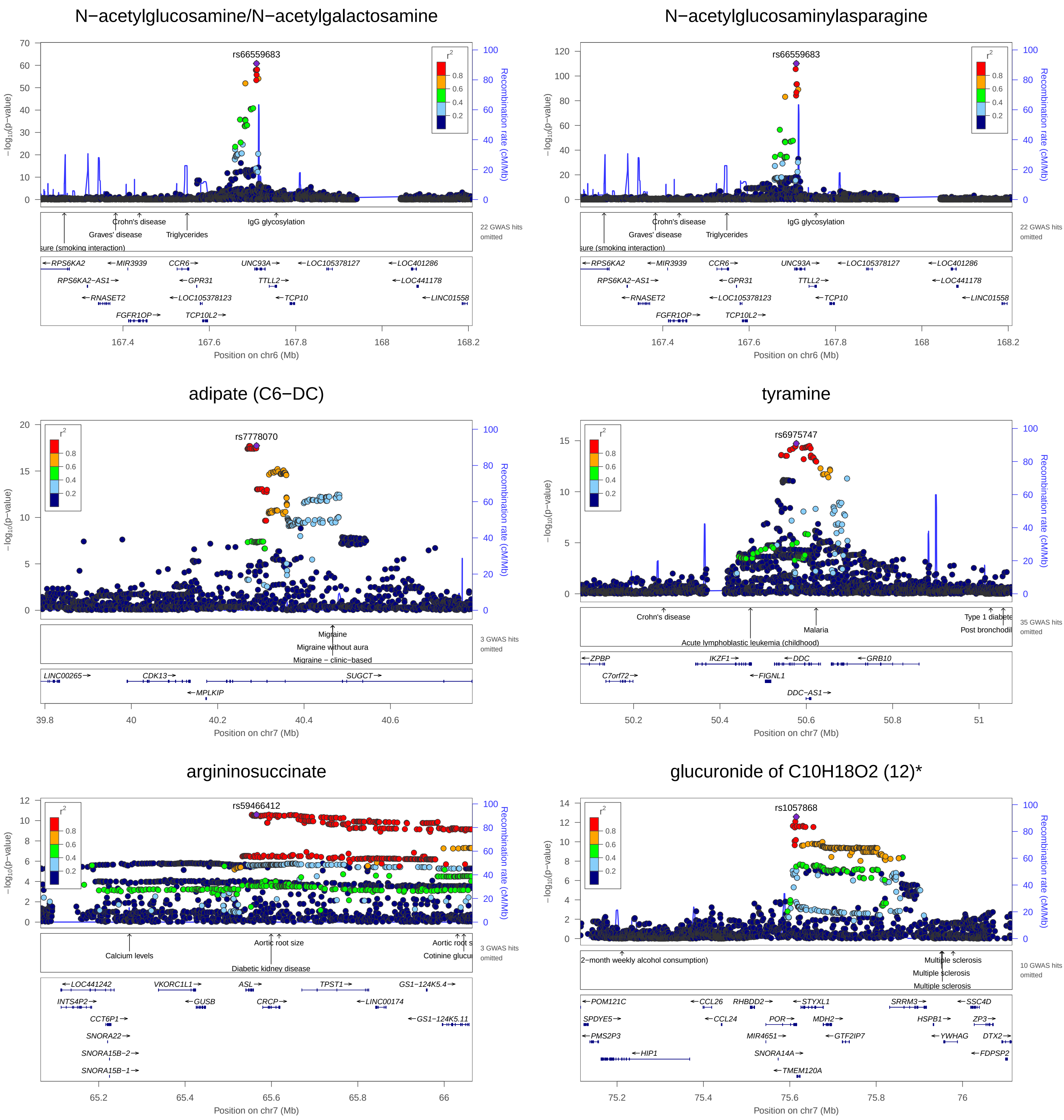
N−acetylglucosamine/N−acetylgalactosamine
rs66559683
−log10(p−value)
Recombination rate (cM/Mb)
r2
0.8
0.6
0.4
0.2
Crohn's disease
Graves' disease
Triglycerides
IgG glycosylation
sure (smoking interaction)
22 GWAS hits omitted
RPS6KA2
MIR3939
CCR6
UNC93A
LOC105378127
LOC401286
RPS6KA2−AS1
GPR31
TTLL2
LOC441178
RNASET2
LOC105378123
TCP10
LINC01558
FGFR1OP
TCP10L2
Position on chr6 (Mb)
N−acetylglucosaminylasparagine
rs66559683
adipate (C6−DC)
rs7778070
Migraine
Migraine without aura
Migraine − clinic−based
3 GWAS hits omitted
LINC00265
CDK13
SUGCT
MPLKIP
Position on chr7 (Mb)
tyramine
rs6975747
Crohn's disease
Malaria
Type 1 diabete
Post bronchodil
Acute lymphoblastic leukemia (childhood)
35 GWAS hits omitted
ZPBP
IKZF1
DDC
GRB10
C7orf72
FIGNL1
DDC−AS1
argininosuccinate
rs59466412
Calcium levels
Aortic root size
Aortic root s
Cotinine glucu
Diabetic kidney disease
3 GWAS hits omitted
LOC441242
VKORC1L1
ASL
TPST1
GS1−124K5.4
INTS4P2
GUSB
CRCP
LINC00174
CCT6P1
GS1−124K5.11
SNORA22
SNORA15B−2
SNORA15B−1
glucuronide of C10H18O2 (12)*
rs1057868
2−month weekly alcohol consumption)
Multiple sclerosis
10 GWAS hits omitted
POM121C
CCL26
RHBDD2
STYXL1
SRRM3
SSC4D
SPDYE5
CCL24
POR
MDH2
HSPB1
ZP3
PMS2P3
MIR4651
GTF2IP7
YWHAG
DTX2
HIP1
SNORA14A
FDPSP2
TMEM120A

laurylcarnitine (C12)

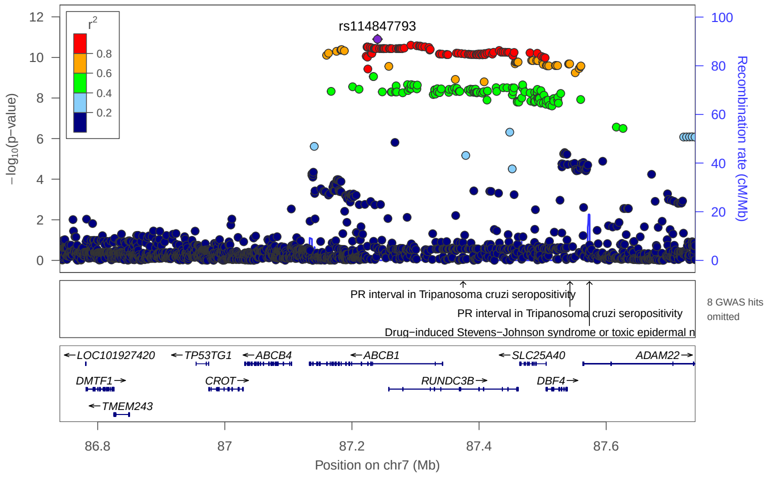

X − 21286

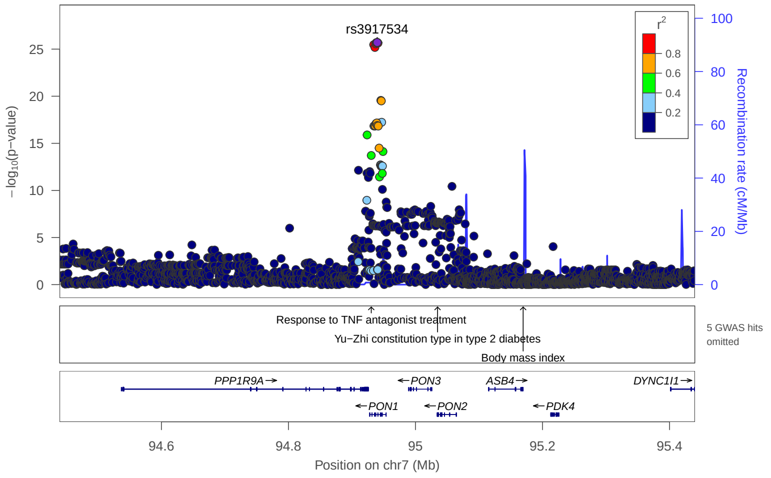

5alpha−androstan−3beta,17beta−diol disulfate

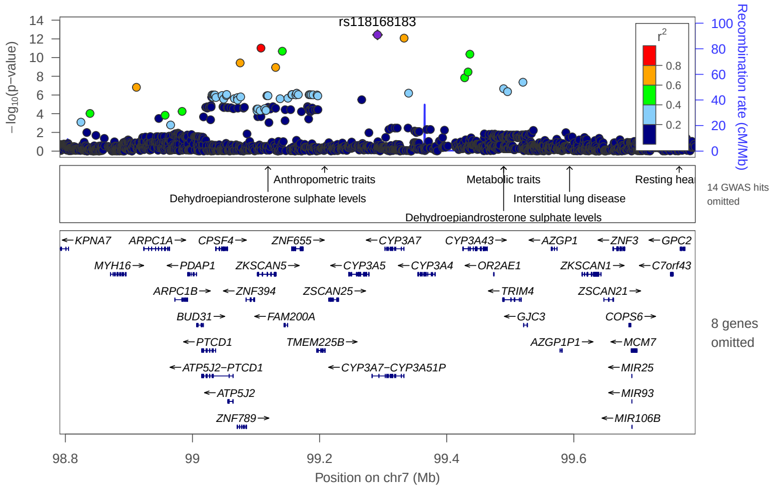

etiocholanolone glucuronide

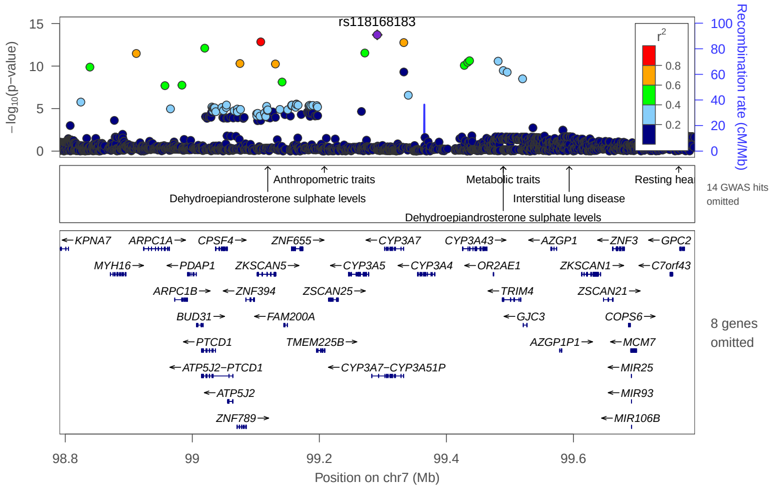

X − 23787

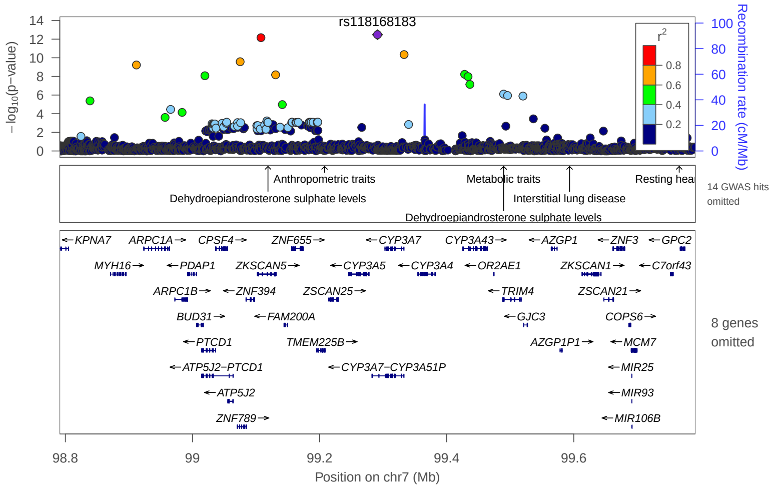

andro steroid monosulfate C19H28O6S (1)*

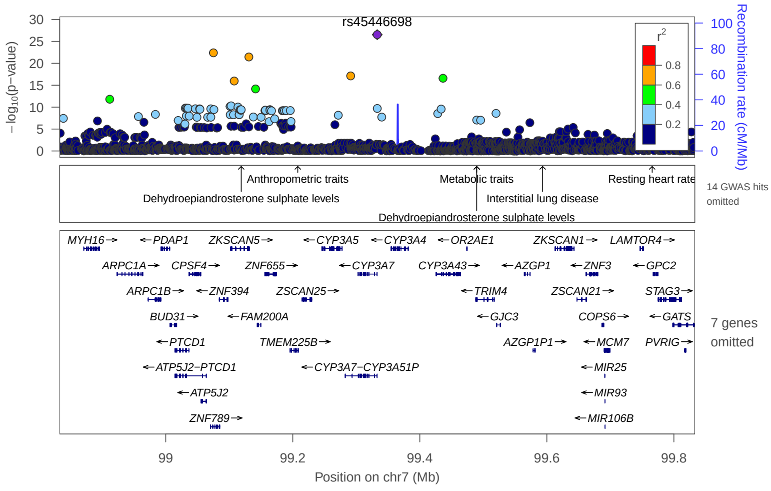

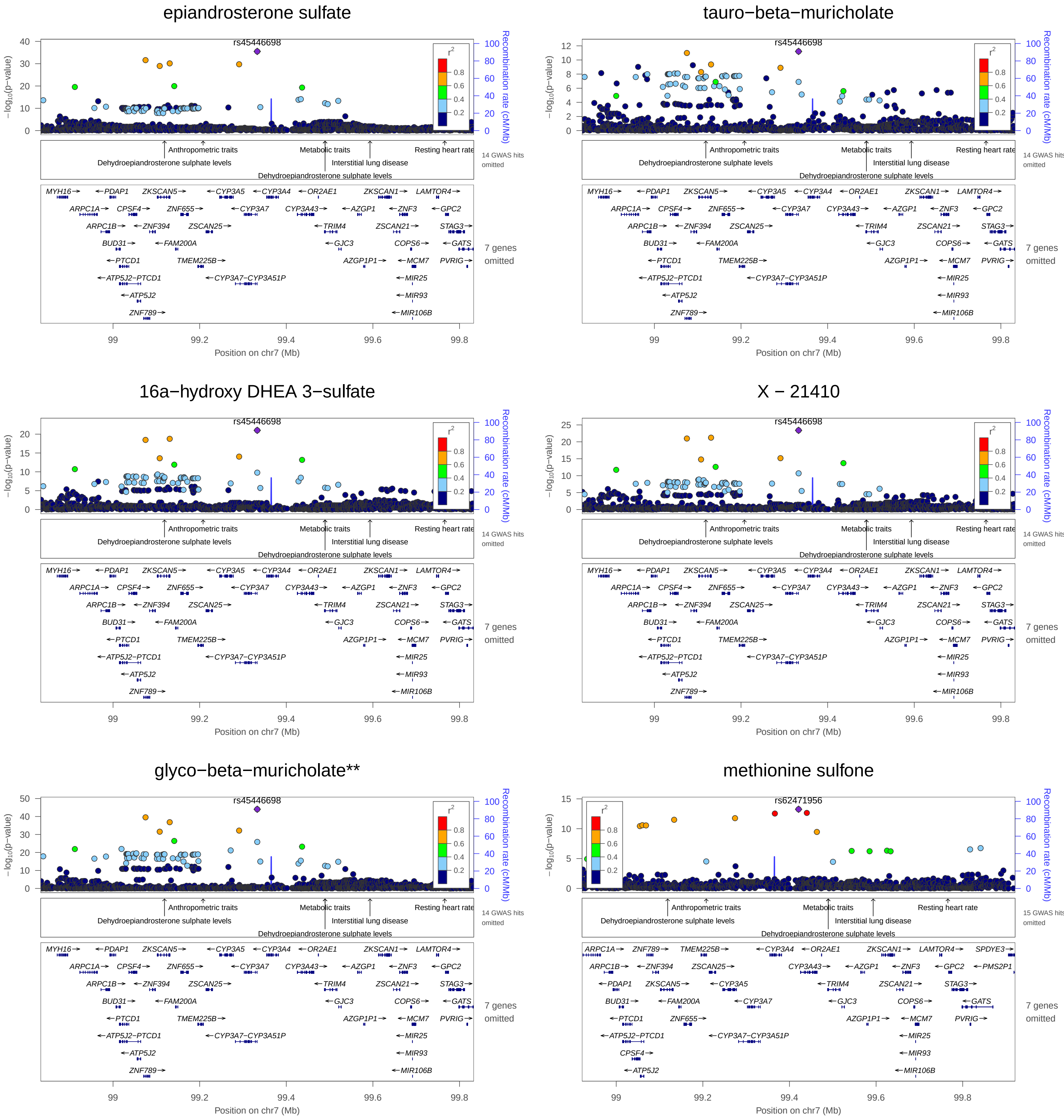

epiandrosterone sulfate
tauro−beta−muricholate
16a−hydroxy DHEA 3−sulfate
X − 21410
glyco−beta−muricholate**
methionine sulfone
rs45446698
rs62471956
−log10(p−value)
Recombination rate (cM/Mb)
r2
Anthropometric traits
Dehydroepiandrosterone sulphate levels
Metabolic traits
Interstitial lung disease
Resting heart rate
14 GWAS hits omitted
15 GWAS hits omitted
MYH16 PDAP1 ZKSCAN5 CYP3A5 CYP3A4 OR2AE1 ZKSCAN1 LAMTOR4
ARPC1A CPSF4 ZNF655 CYP3A7 CYP3A43 AZGP1 ZNF3 GPC2
ARPC1B ZNF394 ZSCAN25 TRIM4 ZSCAN21 STAG3
BUD31 FAM200A GJC3 COPS6 GATS
PTCD1 TMEM225B AZGP1P1 MCM7 PVRIG
ATP5J2−PTCD1 CYP3A7−CYP3A51P MIR25
ATP5J MIR93
ZNF789 MIR106B
SPDYE3 PMS2P1
7 genes omitted
Position on chr7 (Mb)

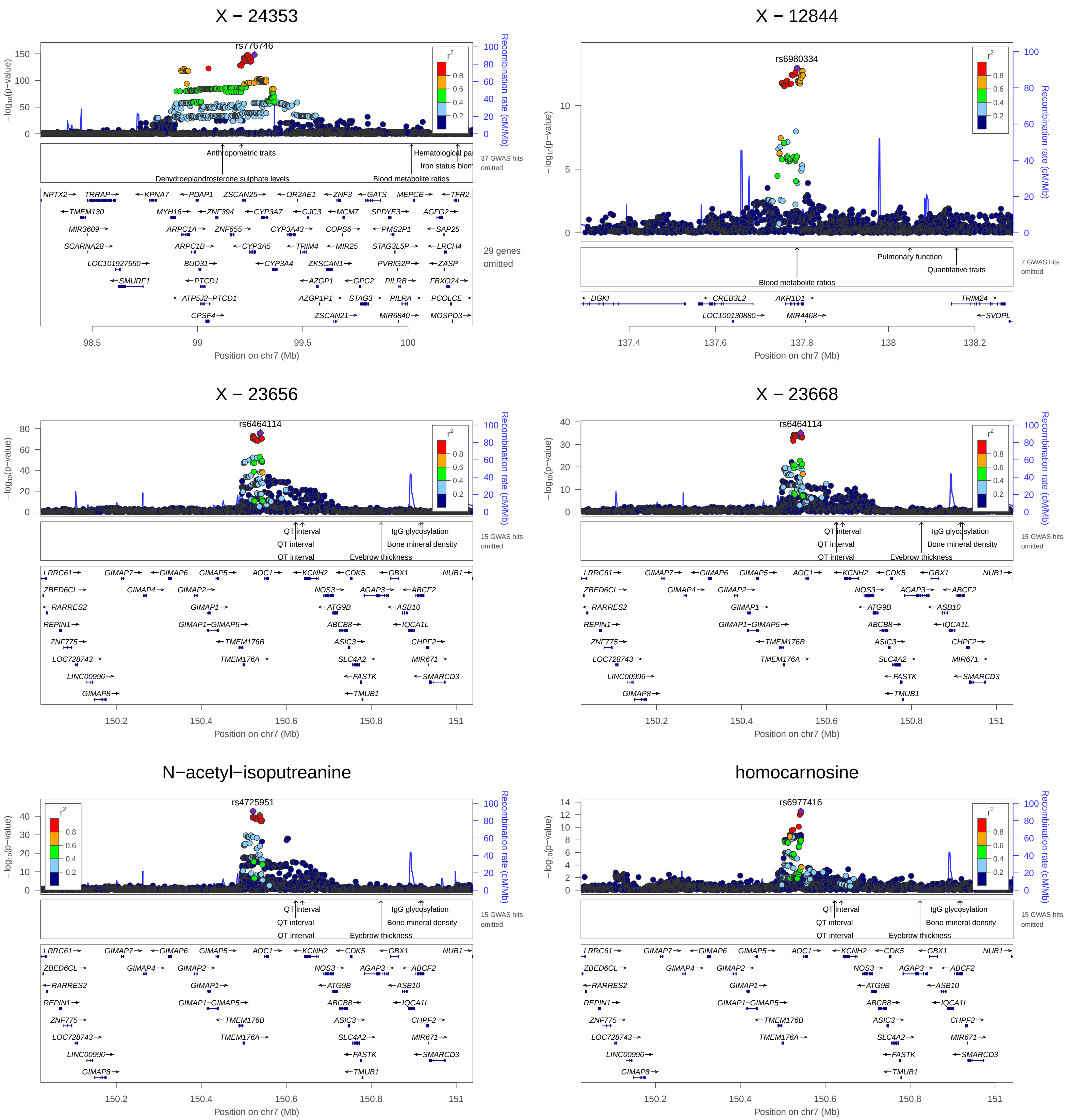

X − 24353
rs776746
−log10(p−value)
Recombination rate (cM/Mb)
Anthropometric traits
Dehydroepiandrosterone sulphate levels
Hematological pa
Iron status biom
Blood metabolite ratios
37 GWAS hits omitted
29 genes omitted
Position on chr7 (Mb)
X − 12844
rs6980334
Pulmonary function
Quantitative traits
Blood metabolite ratios
7 GWAS hits omitted
DGKI
CREB3L2
LOC100130880
AKR1D1
MIR4468
TRIM24
SVOPL
X − 23656
rs6464114
X − 23668
rs6464114
QT interval
IgG glycosylation
Bone mineral density
Eyebrow thickness
15 GWAS hits omitted
N−acetyl−isoputreanine
rs4725951
homocarnosine
rs6977416

1−methylhistamine

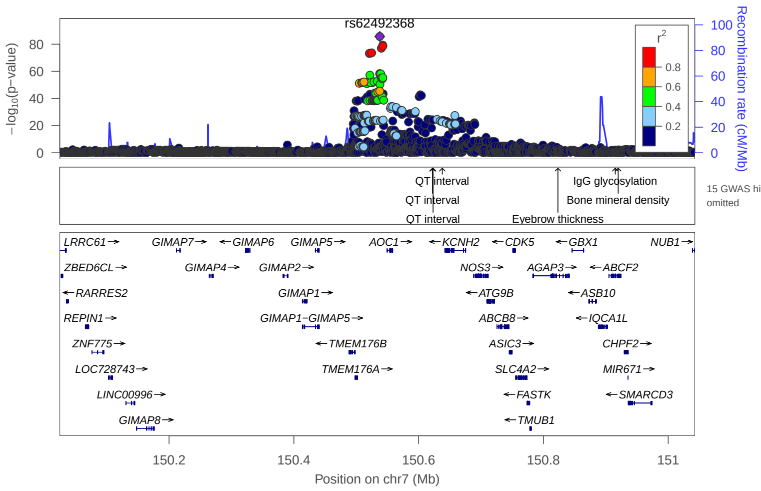

(N(1) + N(8))−acetylspermidine

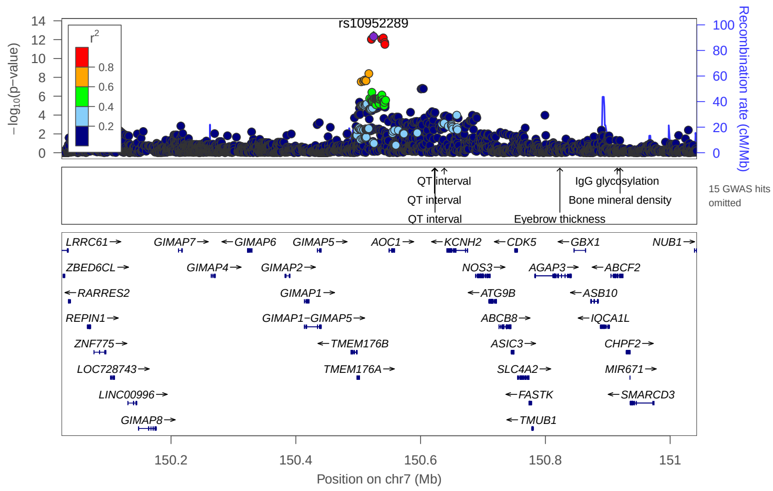

acisoga

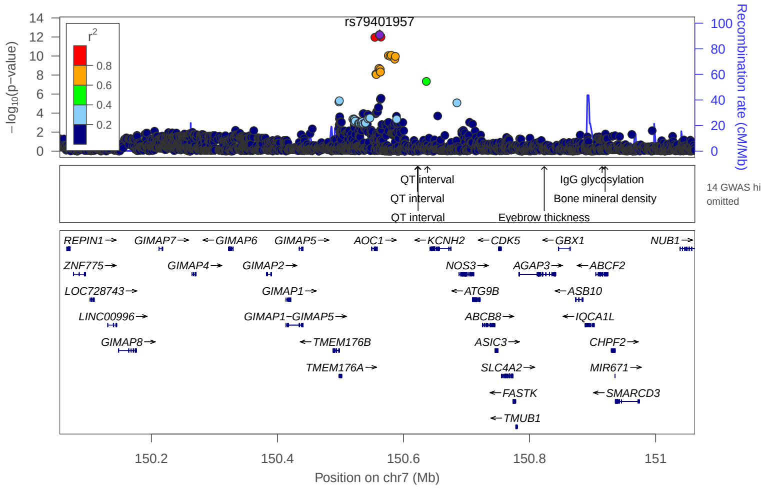

X − 23659

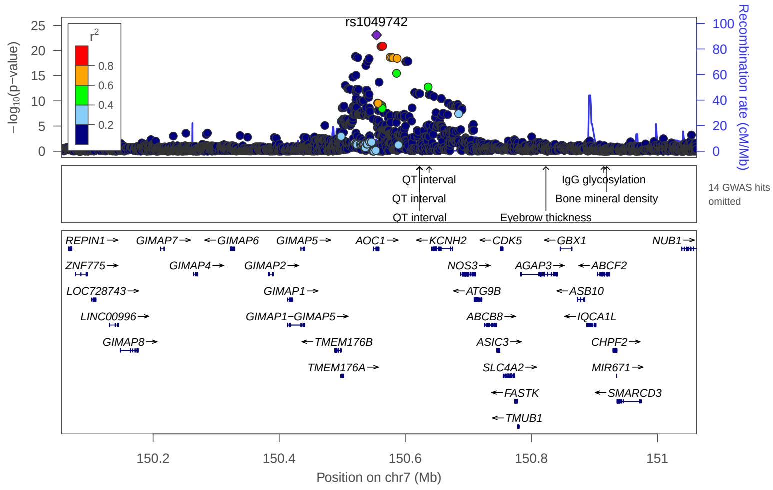

4−acetamidobutanoate

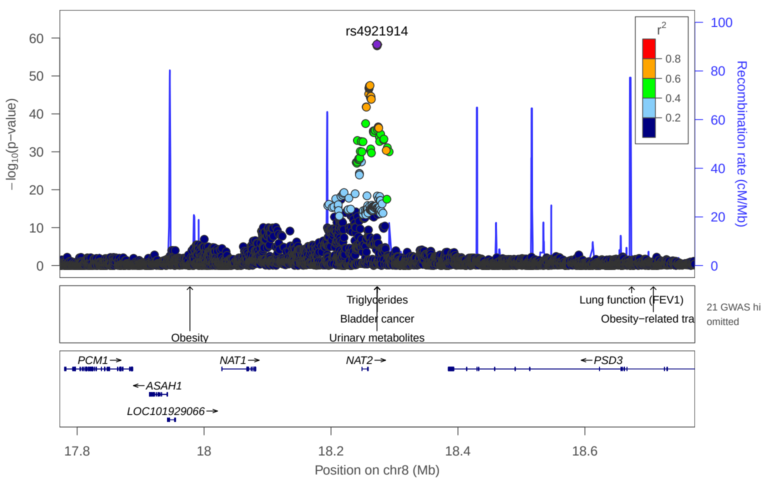

1−methylxanthine

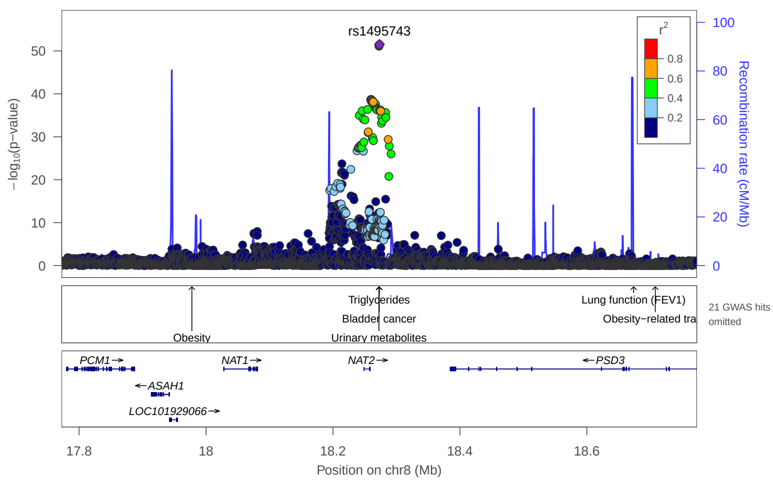

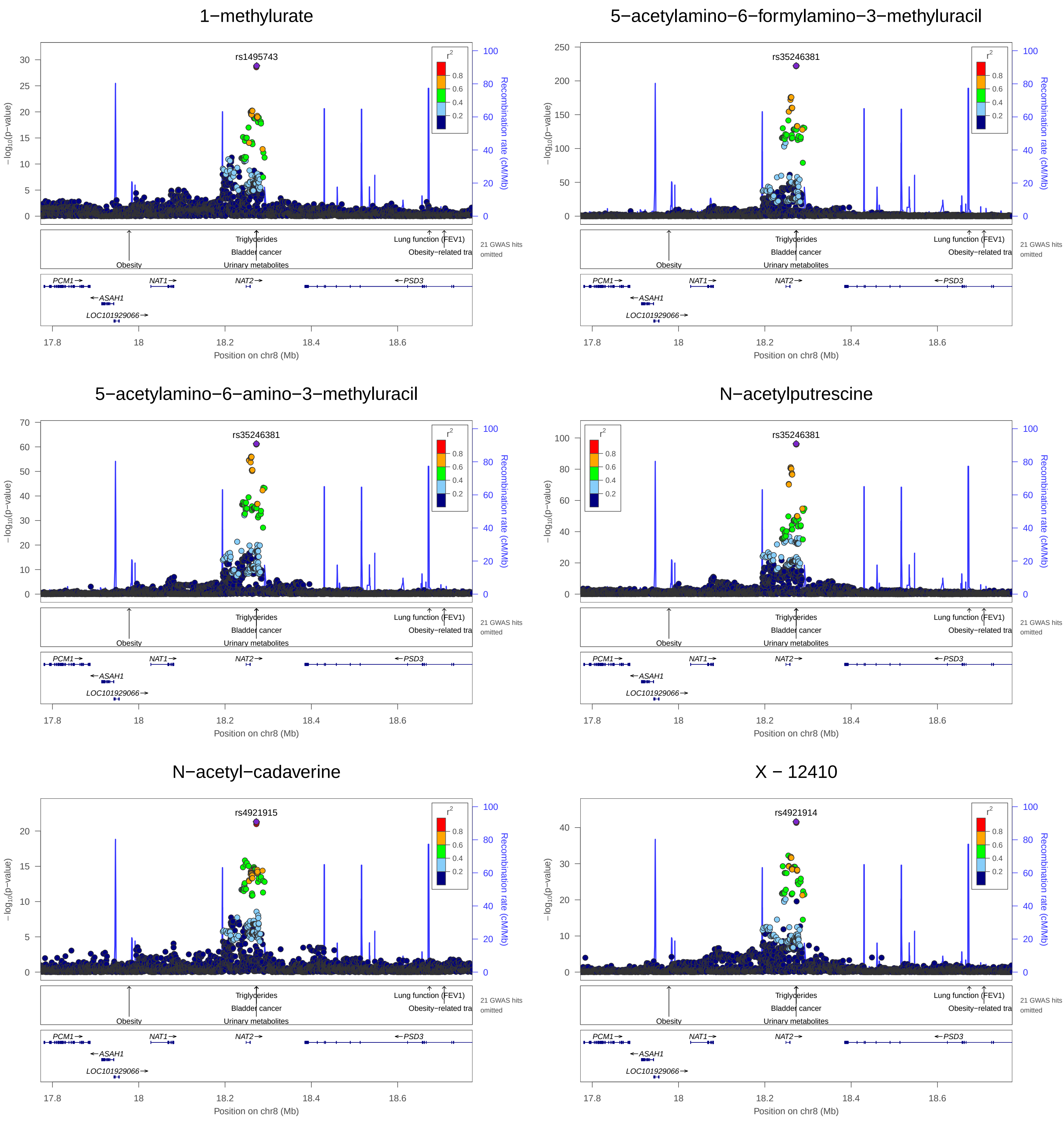
1−methylurate
rs1495743
5−acetylamino−6−formylamino−3−methyluracil
rs35246381
5−acetylamino−6−amino−3−methyluracil
rs35246381
N−acetylputrescine
rs35246381
N−acetyl−cadaverine
rs4921915
X − 12410
rs4921914
r2
0.8
0.6
0.4
0.2
−log10(p−value)
Recombination rate (cM/Mb)
Triglycerides
Bladder cancer
Urinary metabolites
Obesity
Lung function (FEV1)
Obesity−related tra
21 GWAS hits omitted
PCM1
NAT1
NAT2
PSD3
ASAH1
LOC101929066
17.8
18
18.2
18.4
18.6
Position on chr8 (Mb)

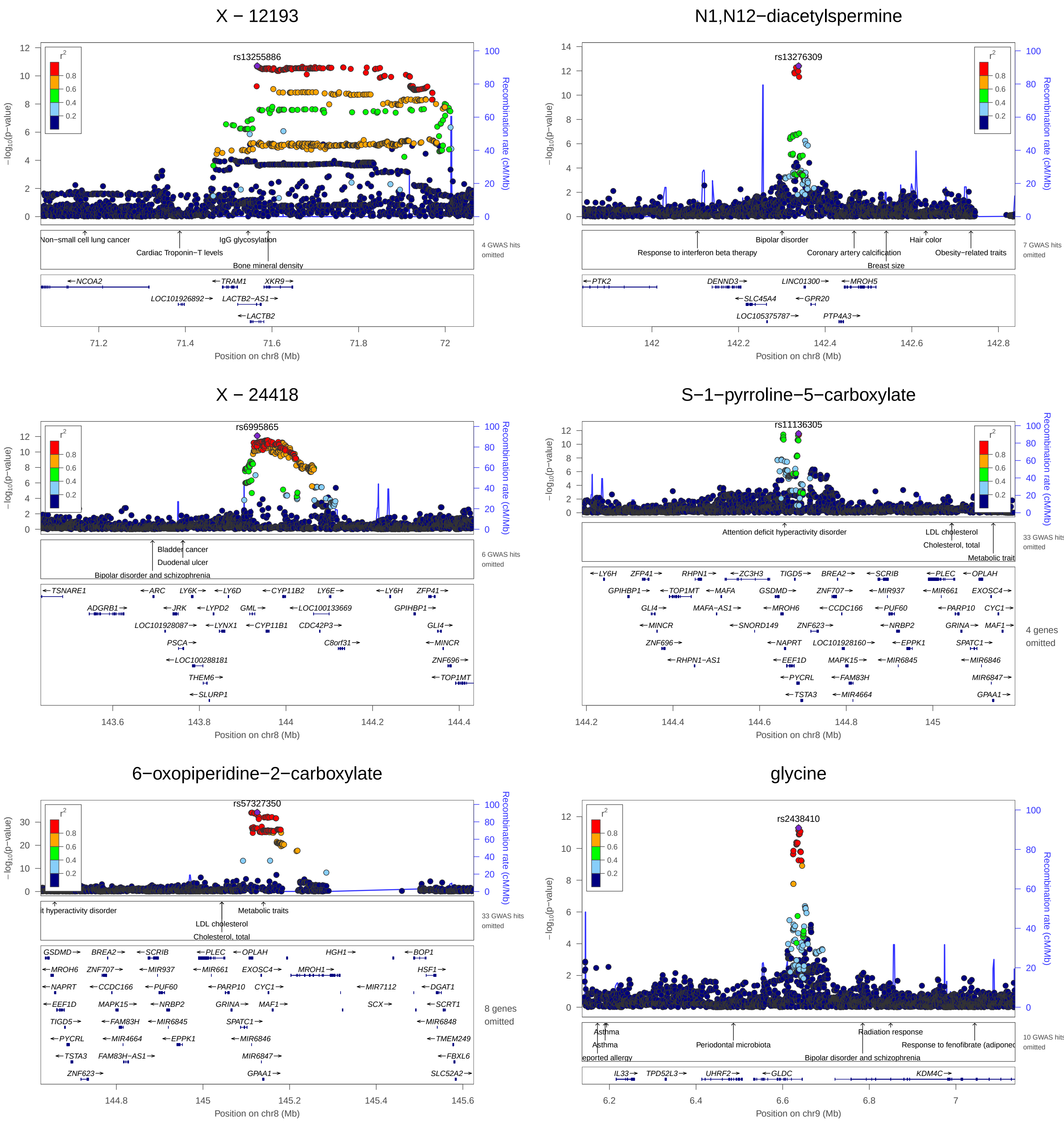
X − 12193
rs13255886
Non−small cell lung cancer
IgG glycosylation
Cardiac Troponin−T levels
Bone mineral density
4 GWAS hits omitted
NCOA2
TRAM1
XKR9
LOC101926892
LACTB2−AS1
LACTB2
Position on chr8 (Mb)
N1,N12−diacetylspermine
rs13276309
Bipolar disorder
Hair color
Response to interferon beta therapy
Coronary artery calcification
Obesity−related traits
Breast size
7 GWAS hits omitted
PTK2
DENND3
LINC01300
MROH5
SLC45A4
GPR20
LOC105375787
PTP4A3
Position on chr8 (Mb)
X − 24418
rs6995865
Bladder cancer
Duodenal ulcer
Bipolar disorder and schizophrenia
6 GWAS hits omitted
Position on chr8 (Mb)
S−1−pyrroline−5−carboxylate
rs11136305
Attention deficit hyperactivity disorder
LDL cholesterol
Cholesterol, total
Metabolic trait
33 GWAS hits omitted
4 genes omitted
Position on chr8 (Mb)
6−oxopiperidine−2−carboxylate
rs57327350
Metabolic traits
LDL cholesterol
Cholesterol, total
33 GWAS hits omitted
8 genes omitted
Position on chr8 (Mb)
glycine
rs2438410
Asthma
Periodontal microbiota
Radiation response
Response to fenofibrate (adiponec
Bipolar disorder and schizophrenia
10 GWAS hits omitted
IL33
TPD52L3
UHRF2
GLDC
KDM4C
Position on chr9 (Mb)
−log10(p−value)
Recombination rate (cM/Mb)

3−methylglutaconate

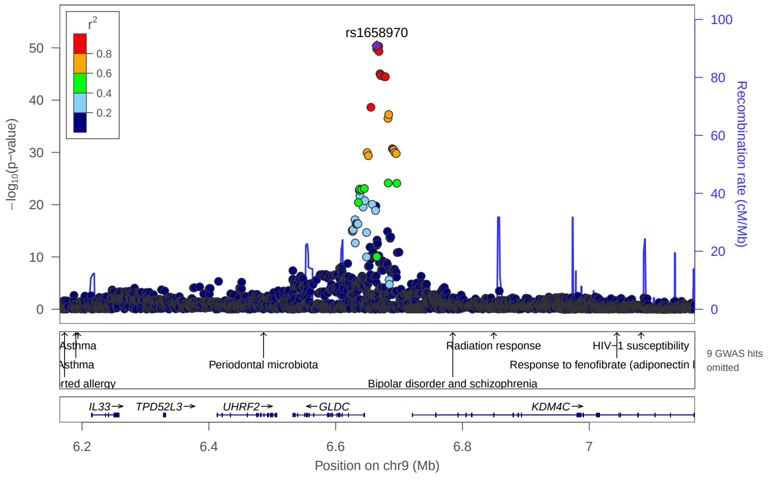

3−methylglutarylcarnitine (2)

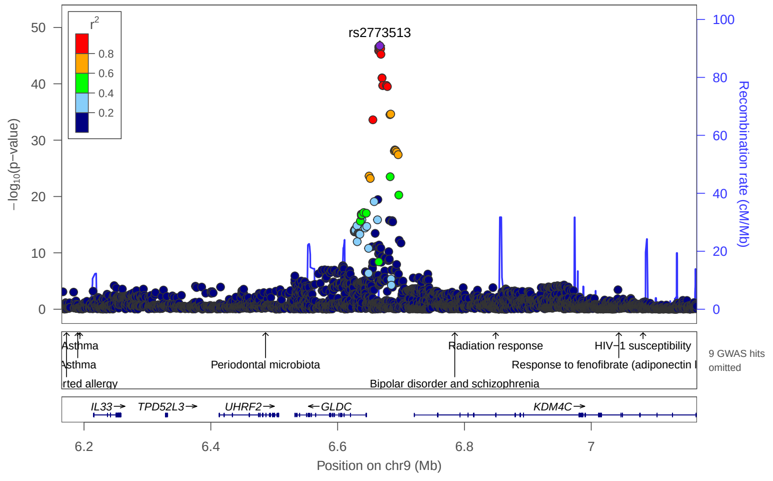

3−methylglutarate/2−methylglutarate

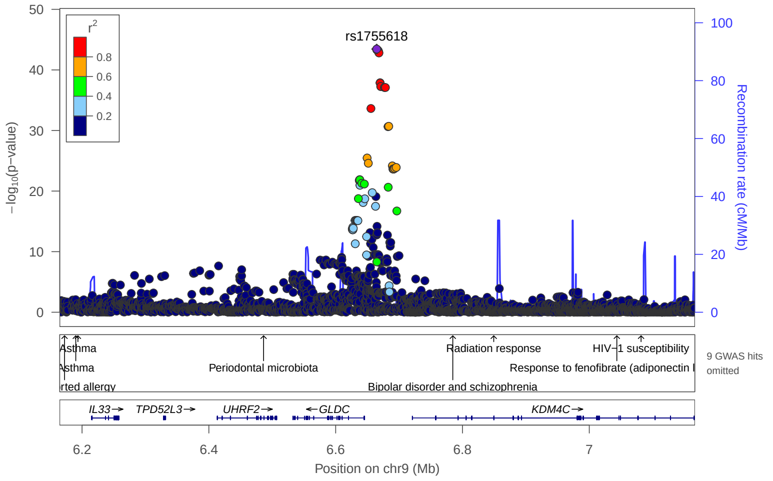

glycerol

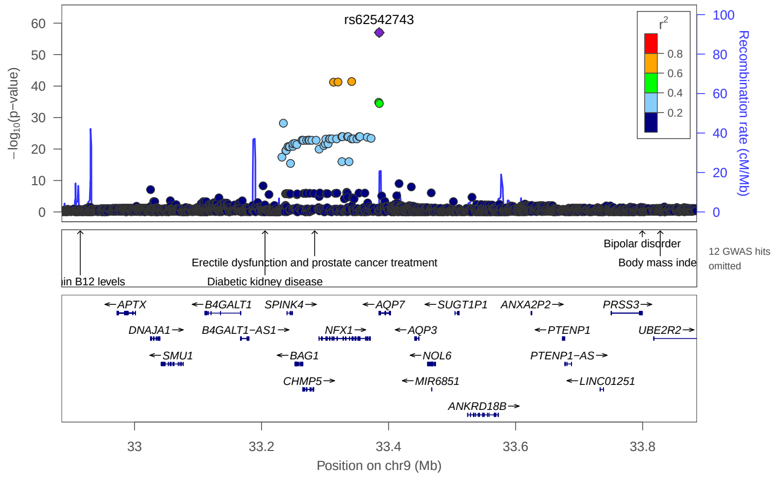

2−hydroxyphenylacetate

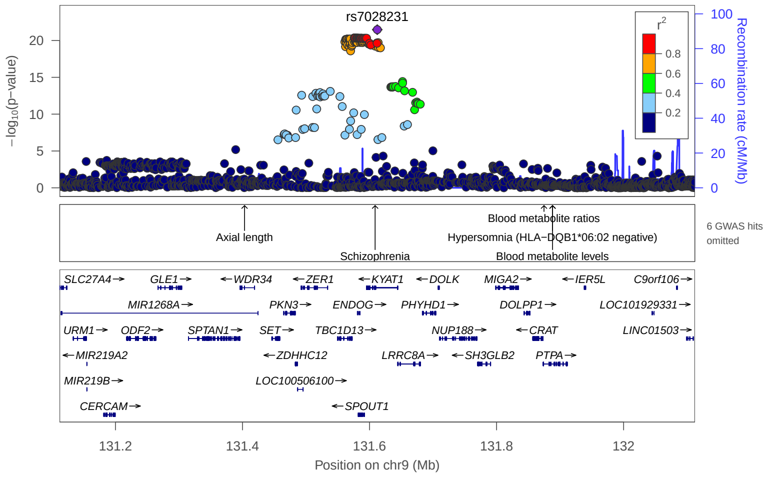

4−imidazoleacetate

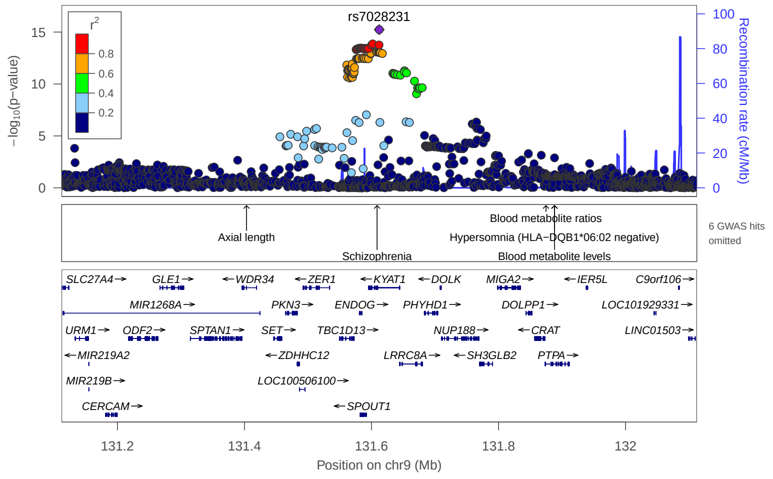

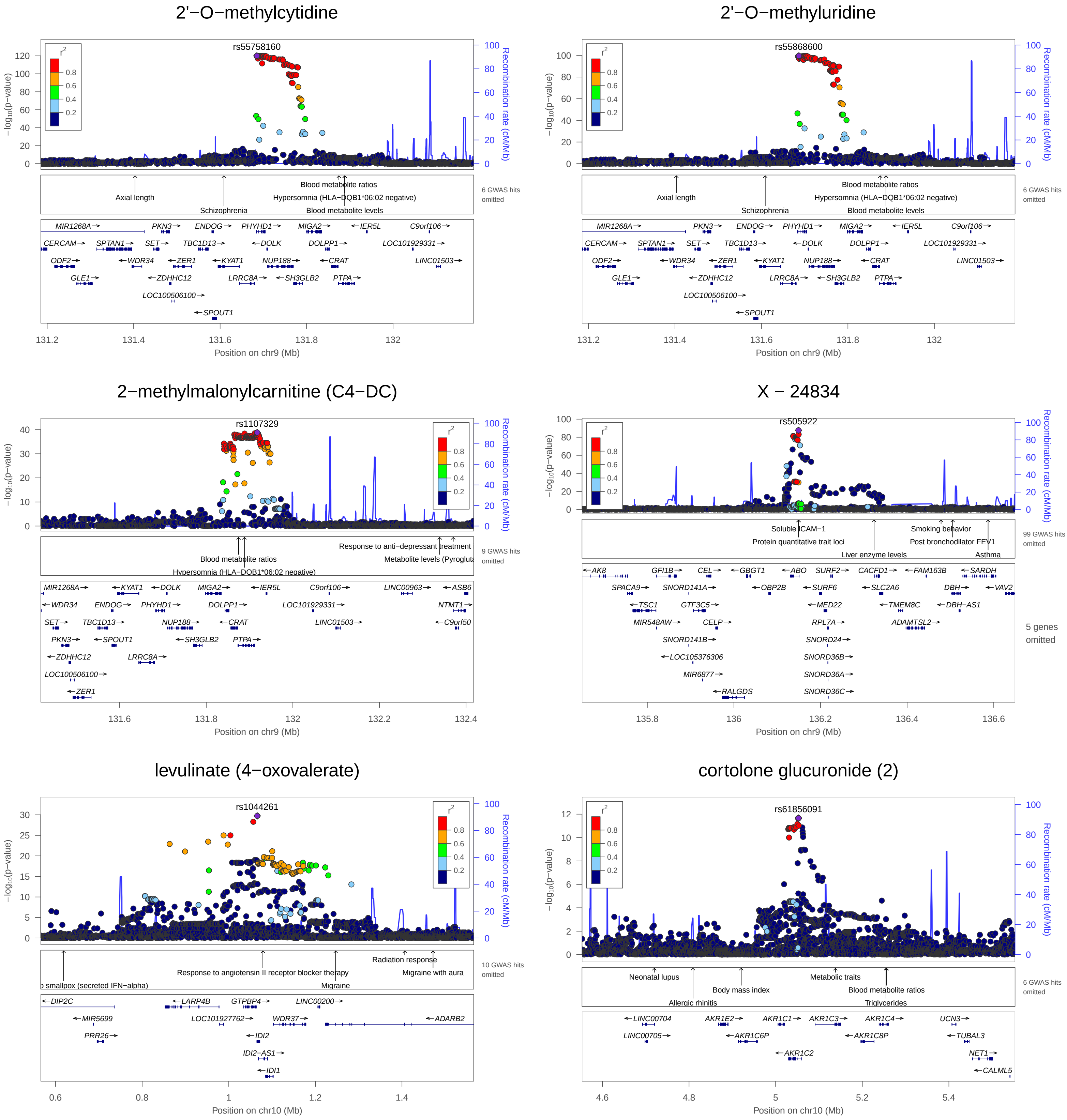
2'−O−methylcytidine
rs55758160
−log10(p−value)
Recombination rate (cM/Mb)
r2
Axial length
Schizophrenia
Blood metabolite ratios
Hypersomnia (HLA−DQB1*06:02 negative)
Blood metabolite levels
6 GWAS hits omitted
MIR1268A PKN3 ENDOG PHYHD1 MIGA2 IER5L C9orf106
CERCAM SPTAN1 SET TBC1D13 DOLK DOLPP1 LOC101929331
ODF2 WDR34 ZER1 KYAT1 NUP188 CRAT LINC01503
GLE1 ZDHHC12 LRRC8A SH3GLB2 PTPA
LOC100506100
SPOUT1
Position on chr9 (Mb)
2'−O−methyluridine
rs55868600
2−methylmalonylcarnitine (C4−DC)
rs1107329
Response to anti−depressant treatment
Metabolite levels (Pyroglut
Blood metabolite ratios
Hypersomnia (HLA−DQB1*06:02 negative)
9 GWAS hits omitted
MIR1268A KYAT1 DOLK MIGA2 IER5L C9orf106 LINC00963 ASB6
WDR34 ENDOG PHYHD1 DOLPP1 LOC101929331 NTMT1
SET TBC1D13 NUP188 CRAT LINC01503 C9orf50
PKN3 SPOUT1 SH3GLB2 PTPA
ZDHHC12 LRRC8A
LOC100506100
ZER1
X − 24834
rs505922
Soluble ICAM−1
Protein quantitative trait loci
Smoking behavior
Post bronchodilator FEV1
Liver enzyme levels
Asthma
99 GWAS hits omitted
AK8 GFI1B CEL GBGT1 ABO SURF2 CACFD1 FAM163B SARDH
SPACA9 SNORD141A OBP2B SURF6 SLC2A6 DBH VAV2
TSC1 GTF3C5 MED22 TMEM8C DBH−AS1
MIR548AW CELP RPL7A ADAMTSL2
SNORD141B SNORD24
LOC105376306 SNORD36B
MIR6877 SNORD36A
RALGDS SNORD36C
5 genes omitted
levulinate (4−oxovalerate)
rs1044261
Radiation response
Response to angiotensin II receptor blocker therapy
Migraine with aura
smallpox (secreted IFN−alpha)
Migraine
10 GWAS hits omitted
DIP2C LARP4B GTPBP4 LINC00200
MIR5699 LOC101927762 WDR37 ADARB2
PRR26 IDI2
IDI2−AS1
IDI1
Position on chr10 (Mb)
cortolone glucuronide (2)
rs61856091
Neonatal lupus
Body mass index
Metabolic traits
Blood metabolite ratios
Allergic rhinitis
Triglycerides
6 GWAS hits omitted
LINC00704 AKR1E2 AKR1C1 AKR1C3 AKR1C4 UCN3
LINC00705 AKR1C6P AKR1C8P TUBAL3
AKR1C2 NET1
CALML5

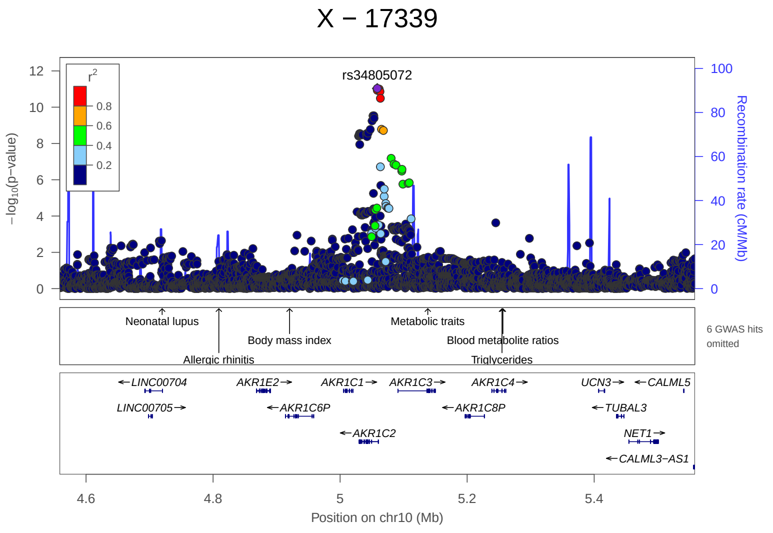
X − 17339
rs34805072
−log10(p−value)
Recombination rate (cM/Mb)
r2
Neonatal lupus
Allergic rhinitis
Body mass index
Metabolic traits
Blood metabolite ratios
Triglycerides
6 GWAS hits omitted
LINC00704
LINC00705
AKR1E2
AKR1C6P
AKR1C1
AKR1C2
AKR1C3
AKR1C4
AKR1C8P
UCN3
CALML5
TUBAL3
NET1
CALML3−AS1
Position on chr10 (Mb)

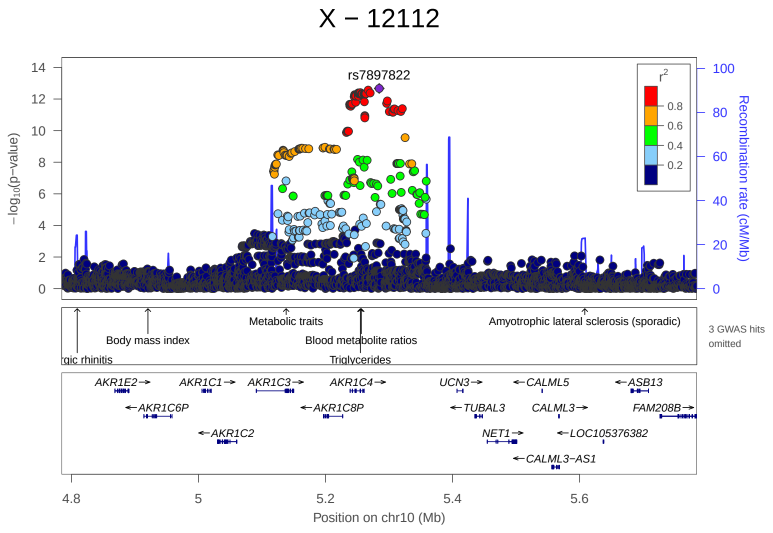
X − 12112
rs7897822
−log10(p−value)
Recombination rate (cM/Mb)
r2
Body mass index
Metabolic traits
Blood metabolite ratios
Triglycerides
Amyotrophic lateral sclerosis (sporadic)
3 GWAS hits omitted
AKR1E2
AKR1C6P
AKR1C1
AKR1C2
AKR1C3
AKR1C4
AKR1C8P
UCN3
TUBAL3
NET1
CALML5
CALML3
CALML3−AS1
LOC105376382
ASB13
FAM208B
Position on chr10 (Mb)

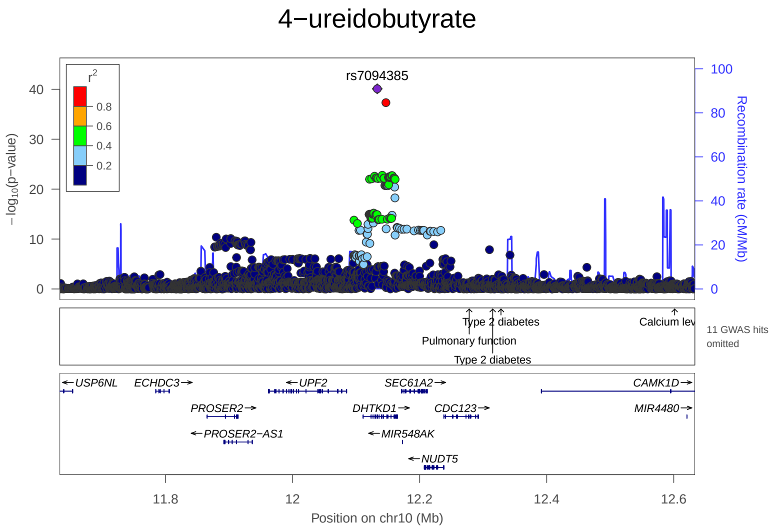
4−ureidobutyrate
rs7094385
−log10(p−value)
Recombination rate (cM/Mb)
r2
Type 2 diabetes
Pulmonary function
Calcium lev
11 GWAS hits omitted
USP6NL
ECHDC3
PROSER2
PROSER2−AS1
UPF2
DHTKD1
SEC61A2
MIR548AK
NUDT5
CDC123
CAMK1D
MIR4480
Position on chr10 (Mb)

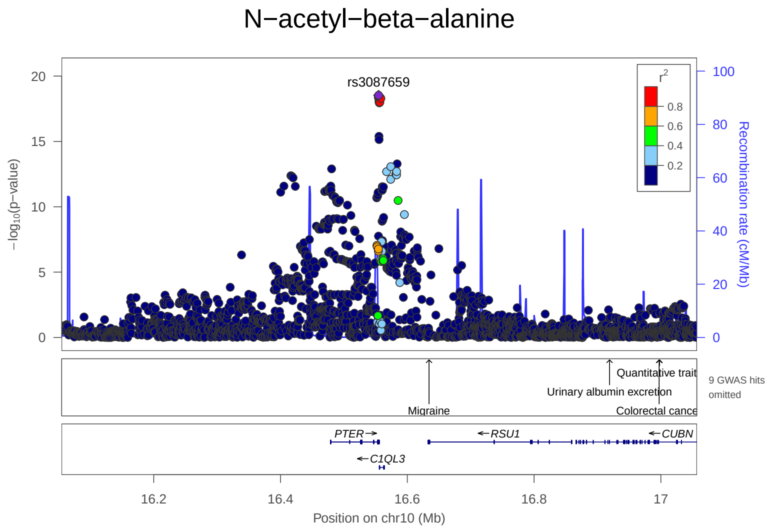
N−acetyl−beta−alanine
rs3087659
−log10(p−value)
Recombination rate (cM/Mb)
r2
Quantitative trait
Urinary albumin excretion
Migraine
Colorectal cance
9 GWAS hits omitted
PTER
C1QL3
RSU1
CUBN
Position on chr10 (Mb)

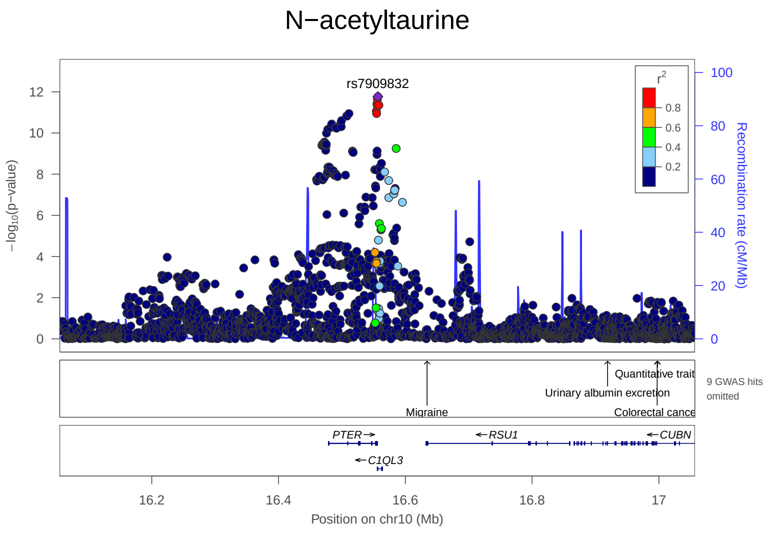
N−acetyltaurine
rs7909832
−log10(p−value)
Recombination rate (cM/Mb)
r2
Quantitative trait
Urinary albumin excretion
Migraine
Colorectal cance
9 GWAS hits omitted
PTER
C1QL3
RSU1
CUBN
Position on chr10 (Mb)

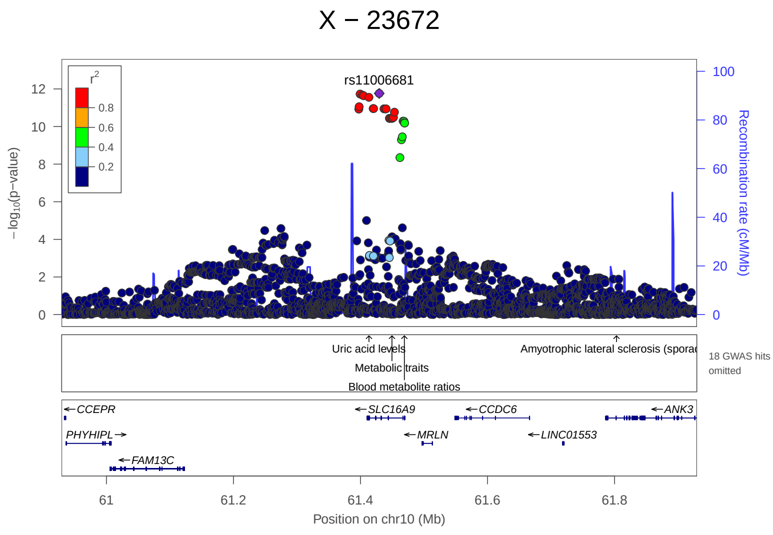
X − 23672
rs11006681
−log10(p−value)
Recombination rate (cM/Mb)
r2
Uric acid levels
Metabolic traits
Blood metabolite ratios
Amyotrophic lateral sclerosis (sporad
18 GWAS hits omitted
CCEPR
PHYHIPL
FAM13C
SLC16A9
MRLN
CCDC6
LINC01553
ANK3
Position on chr10 (Mb)

betaine

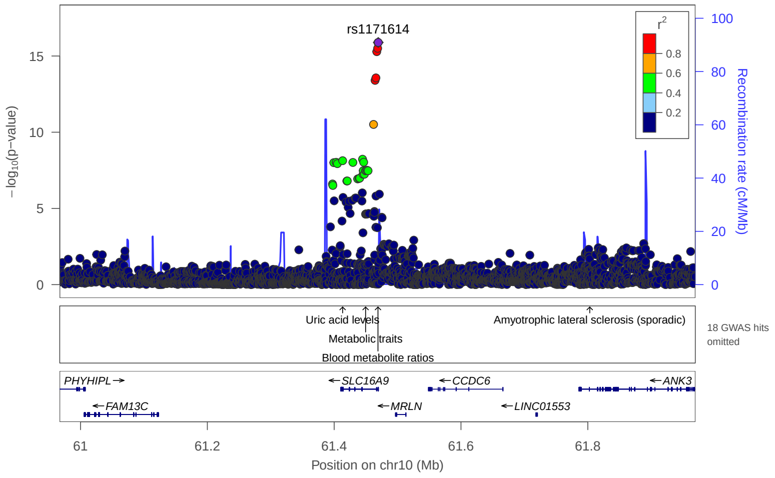

X − 17324

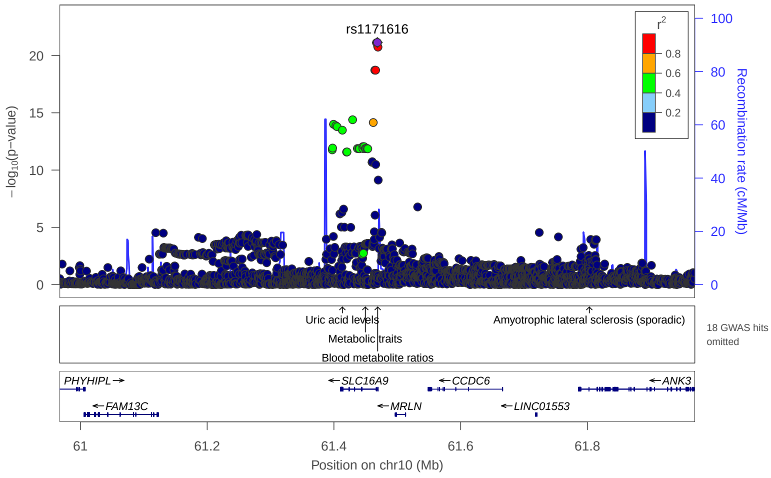

undecenoylcarnitine (C11:1)

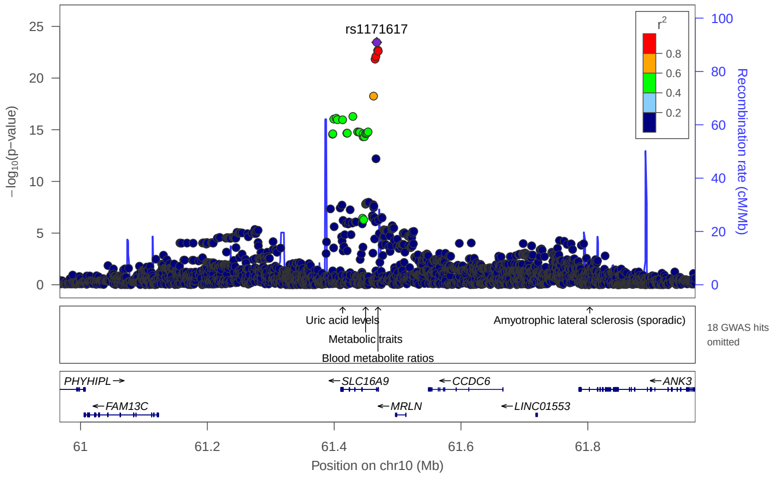

3−hydroxyphenylacetoylcarnitine

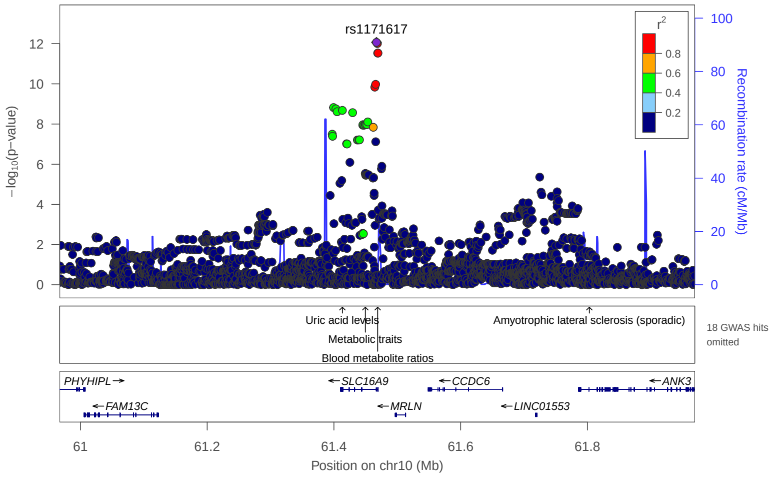

indoleacetoylcarnitine*

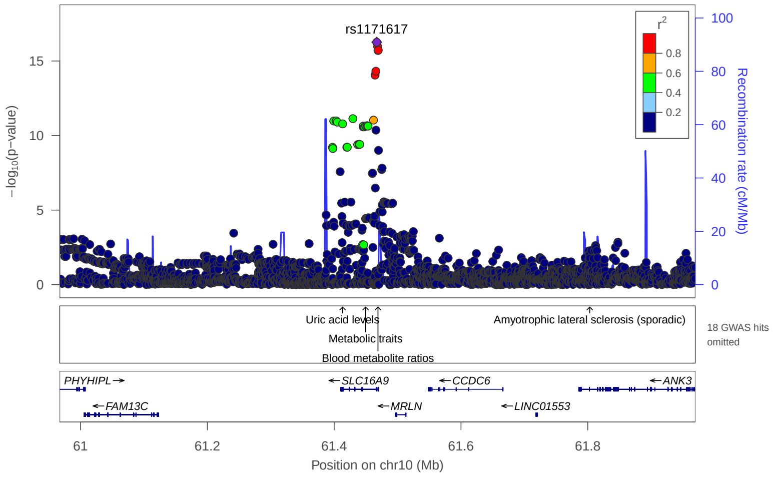

phenylalanylglycine

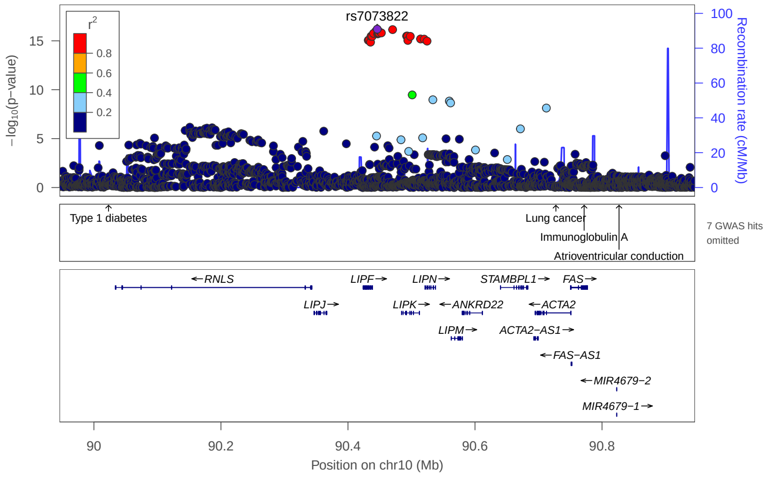

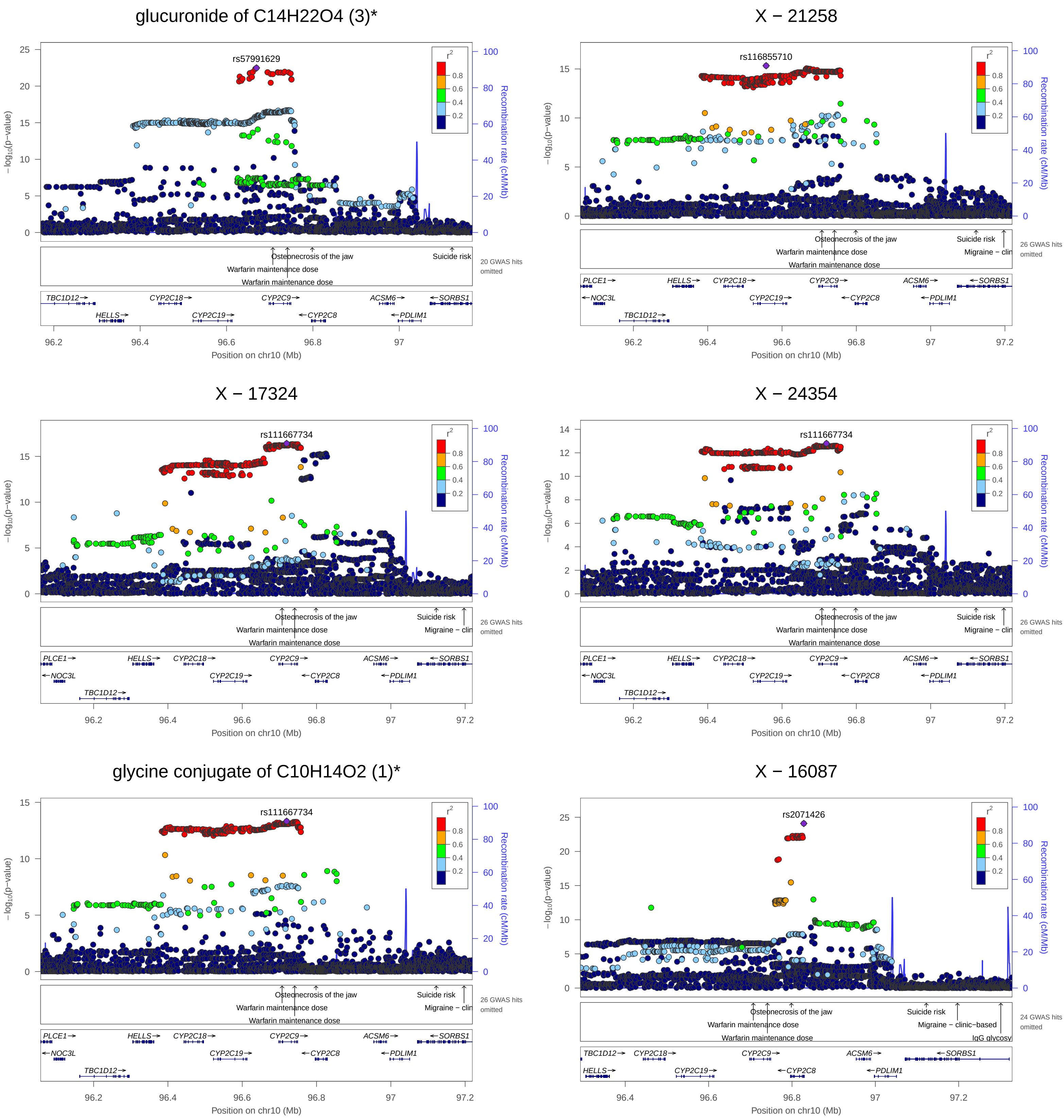
glucuronide of C14H22O4 (3)*
rs57991629
X − 21258
rs116855710
X − 17324
rs111667734
X − 24354
rs111667734
glycine conjugate of C10H14O2 (1)*
rs111667734
X − 16087
rs2071426
r2
−log10(p−value)
Recombination rate (cM/Mb)
Position on chr10 (Mb)
Osteonecrosis of the jaw
Warfarin maintenance dose
Suicide risk
Migraine − clin
Migraine − clinic−based
IgG glycosy
20 GWAS hits omitted
26 GWAS hits omitted
24 GWAS hits omitted
PLCE1
NOC3L
TBC1D12
HELLS
CYP2C18
CYP2C19
CYP2C9
CYP2C8
ACSM6
PDLIM1
SORBS1

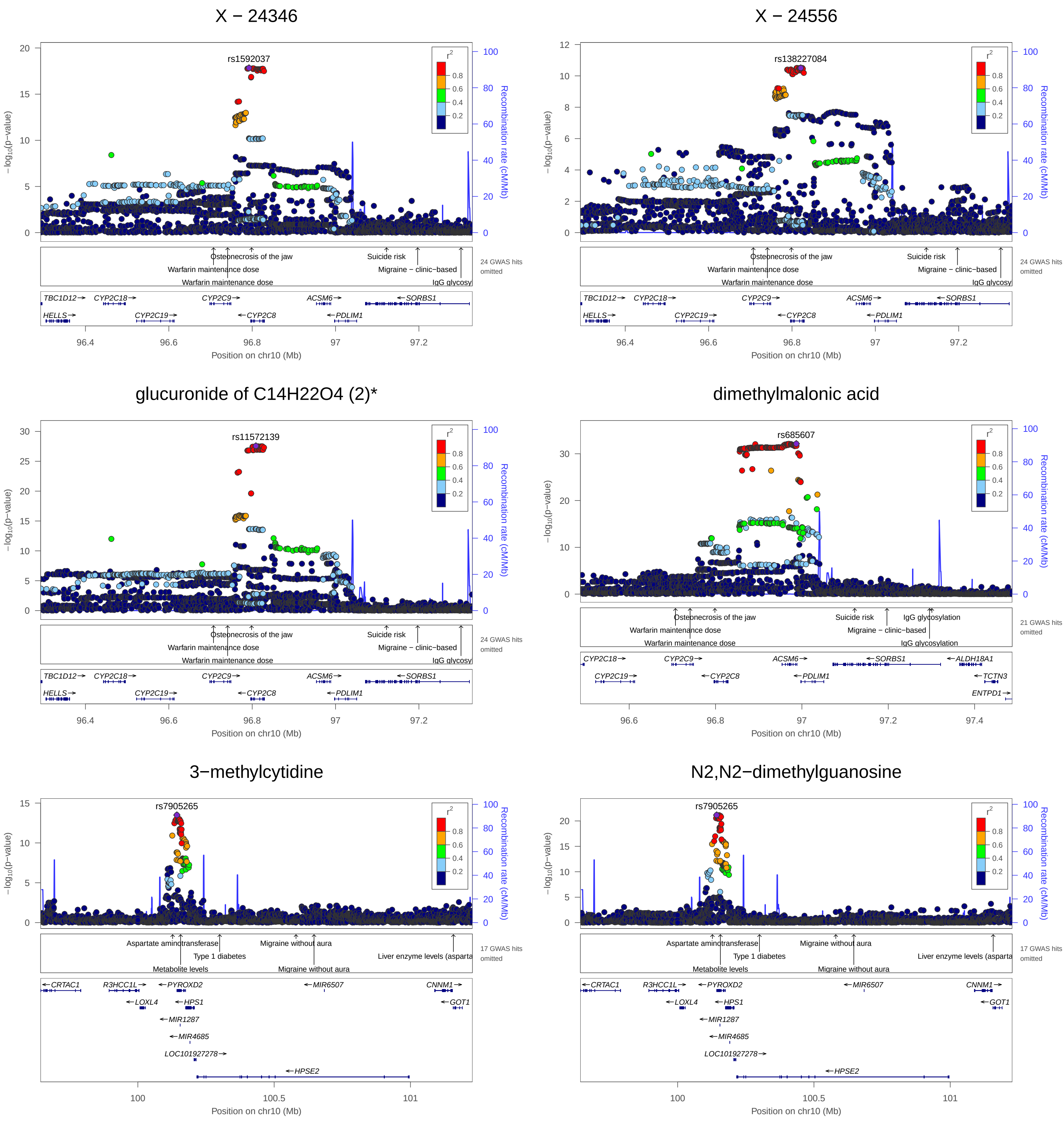
X − 24346
rs1592037
X − 24556
rs138227084
glucuronide of C14H22O4 (2)*
rs11572139
dimethylmalonic acid
rs685607
3−methylcytidine
rs7905265
N2,N2−dimethylguanosine
rs7905265
−log10(p−value)
Recombination rate (cM/Mb)
r2
0.8
0.6
0.4
0.2
Osteonecrosis of the jaw
Warfarin maintenance dose
Suicide risk
Migraine − clinic−based
IgG glycosylation
24 GWAS hits omitted
21 GWAS hits omitted
Aspartate aminotransferase
Type 1 diabetes
Metabolite levels
Migraine without aura
Liver enzyme levels (asparta
17 GWAS hits omitted
TBC1D12
CYP2C18
CYP2C9
ACSM6
SORBS1
HELLS
CYP2C19
CYP2C8
PDLIM1
ALDH18A1
TCTN3
ENTPD1
CRTAC1
R3HCC1L
PYROXD2
MIR6507
CNNM1
LOXL4
HPS1
GOT1
MIR1287
MIR4685
LOC101927278
HPSE2
Position on chr10 (Mb)

N−methylpipecolate

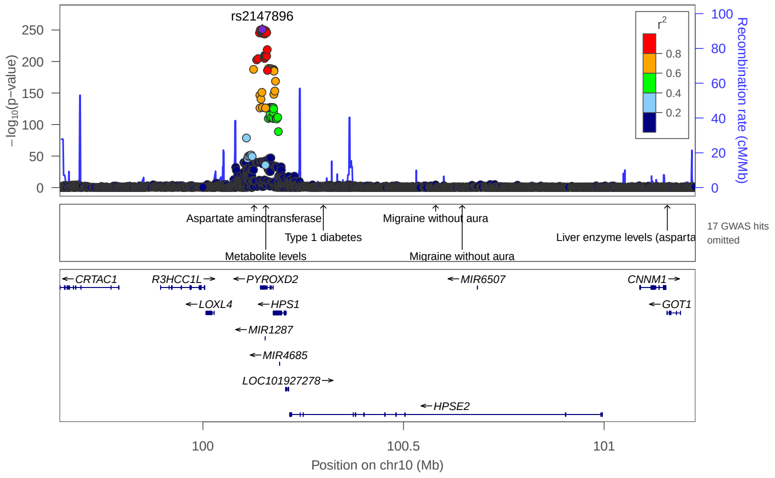

X − 22162

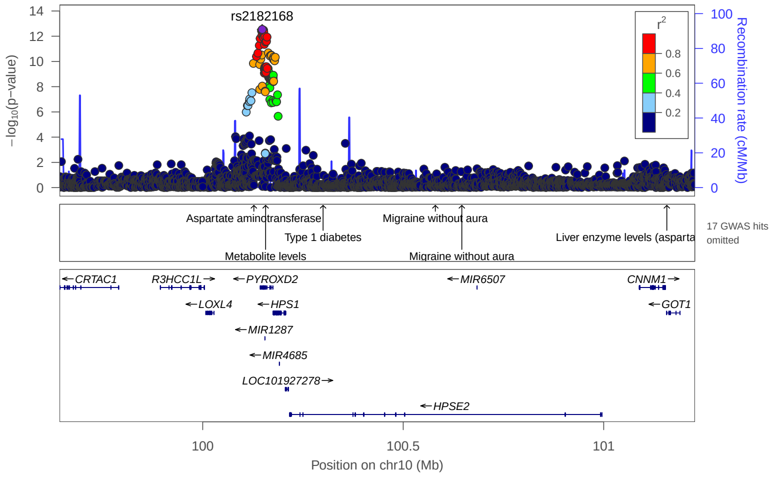

X − 12112

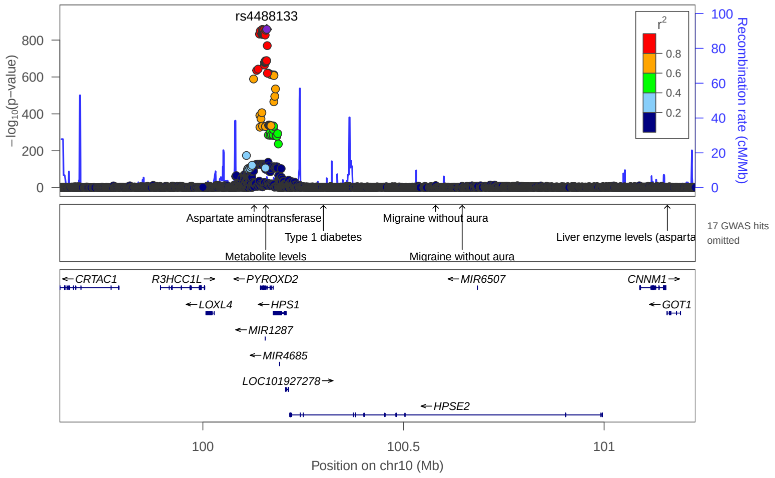

N2−acetyl,N6,N6−dimethyllysine

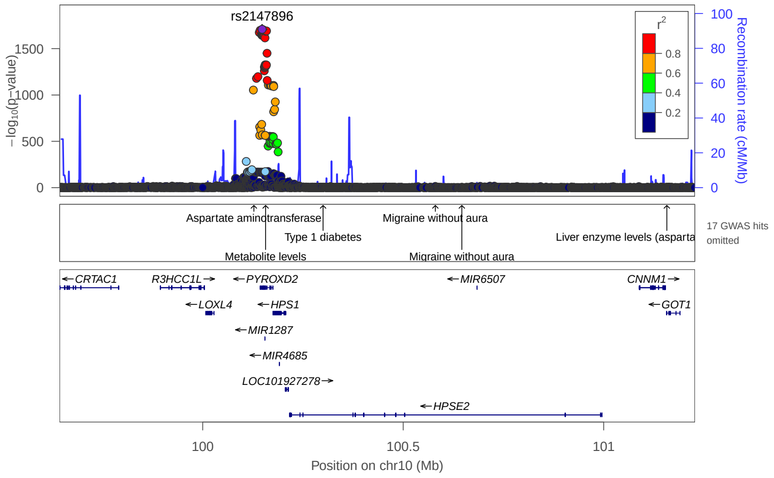

N,N−dimethyl−5−aminovalerate

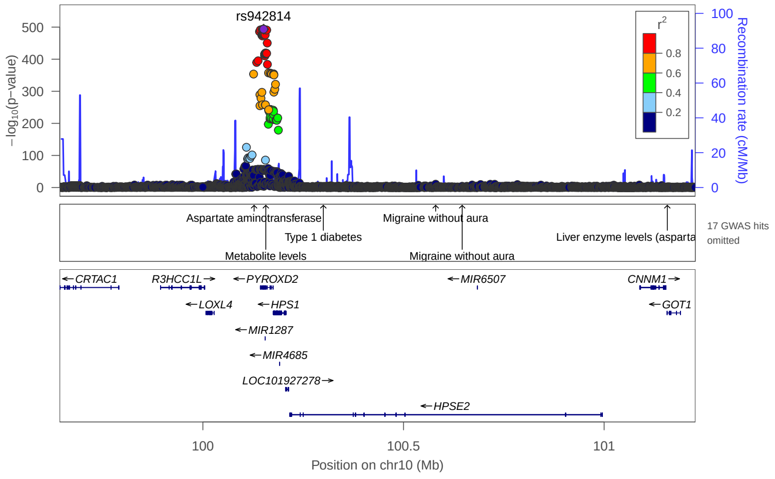

N6−methyllysine

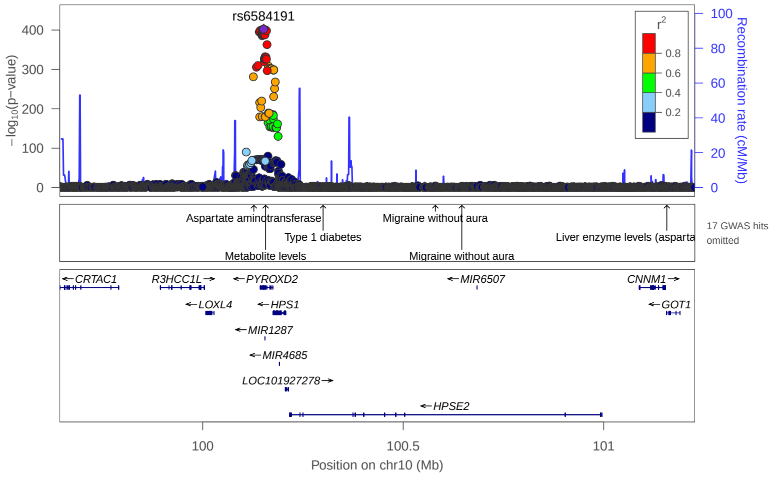

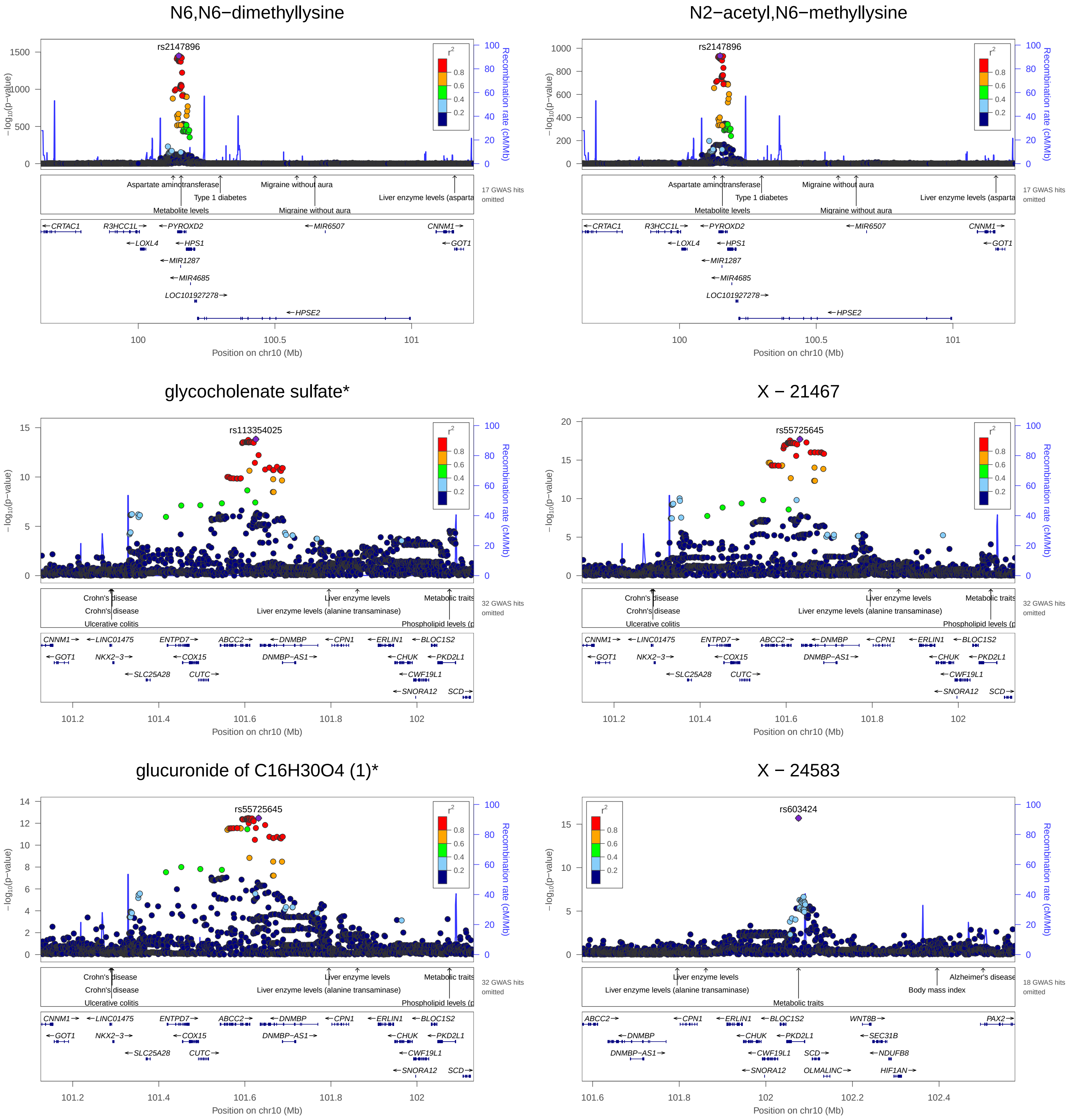
N6,N6−dimethyllysine
N2−acetyl,N6−methyllysine
glycocholenate sulfate*
X − 21467
glucuronide of C16H30O4 (1)*
X − 24583
rs2147896
rs2147896
rs113354025
rs55725645
rs55725645
rs603424
−log10(p−value)
Recombination rate (cM/Mb)
r2
0.8
0.6
0.4
0.2
Position on chr10 (Mb)
17 GWAS hits omitted
32 GWAS hits omitted
18 GWAS hits omitted
Aspartate aminotransferase
Type 1 diabetes
Metabolite levels
Migraine without aura
Liver enzyme levels (asparta
Crohn's disease
Ulcerative colitis
Liver enzyme levels
Liver enzyme levels (alanine transaminase)
Metabolic traits
Phospholipid levels (p
Alzheimer's disease
Body mass index
CRTAC1
R3HCC1L
PYROXD2
LOXL4
HPS1
MIR1287
MIR4685
LOC101927278
HPSE2
MIR6507
CNNM1
GOT1
LINC01475
NKX2−3
SLC25A28
ENTPD7
COX15
CUTC
ABCC2
DNMBP
DNMBP−AS1
CPN1
ERLIN1
CHUK
CWF19L1
SNORA12
BLOC1S2
PKD2L1
SCD
OLMALINC
WNT8B
SEC31B
NDUFB8
HIF1AN
PAX2

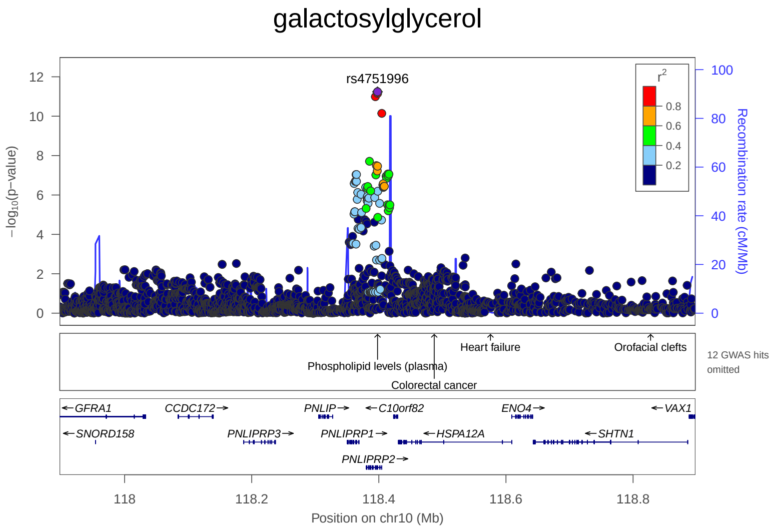
galactosylglycerol
rs4751996
Recombination rate (cM/Mb)
12 GWAS hits omitted
Phospholipid levels (plasma)
Colorectal cancer
Heart failure
Orofacial clefts
GFRA1
CCDC172
PNLIP
C10orf82
ENO4
VAX1
SNORD158
PNLIPRP3
PNLIPRP1
HSPA12A
SHTN1
PNLIPRP2
Position on chr10 (Mb)

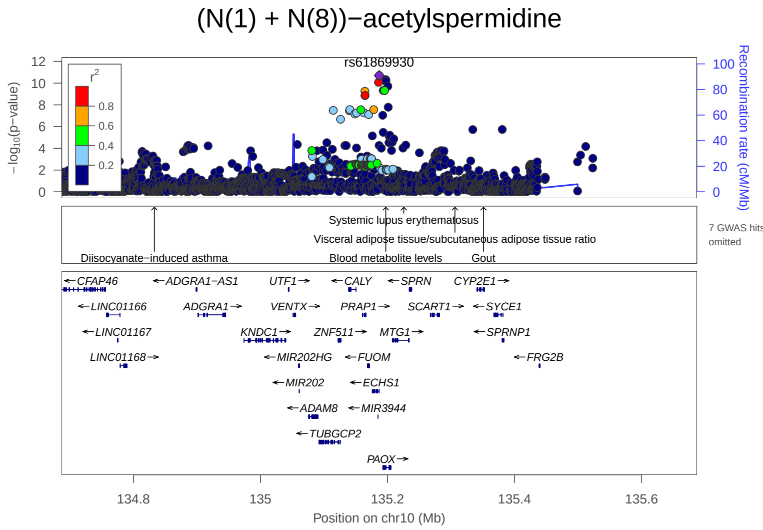
(N(1) + N(8))−acetylspermidine
rs61869930
Recombination rate (cM/Mb)
7 GWAS hits omitted
Systemic lupus erythematosus
Visceral adipose tissue/subcutaneous adipose tissue ratio
Diisocyanate−induced asthma
Blood metabolite levels
Gout
Position on chr10 (Mb)

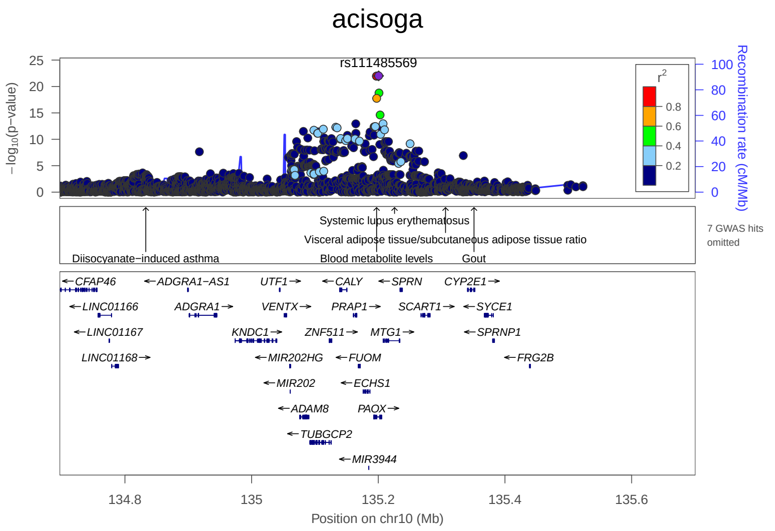
acisoga
rs111485569
Recombination rate (cM/Mb)
7 GWAS hits omitted
Systemic lupus erythematosus
Visceral adipose tissue/subcutaneous adipose tissue ratio
Diisocyanate−induced asthma
Blood metabolite levels
Gout
Position on chr10 (Mb)

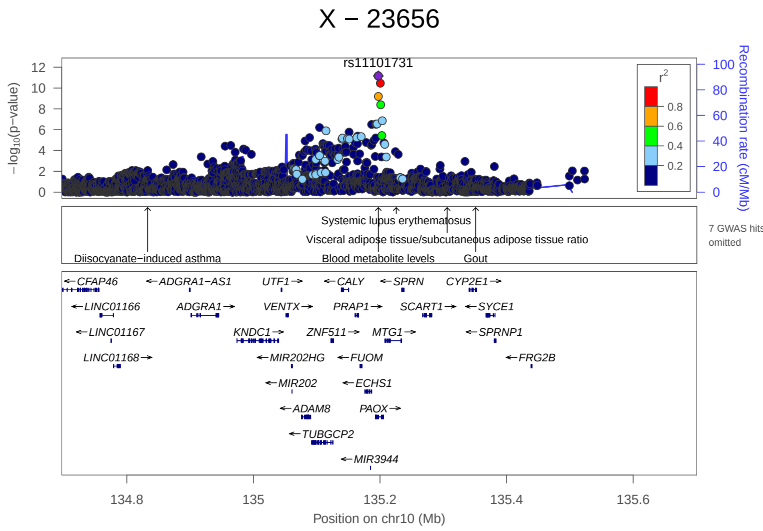
X − 23656
rs11101731
Recombination rate (cM/Mb)
7 GWAS hits omitted
Systemic lupus erythematosus
Visceral adipose tissue/subcutaneous adipose tissue ratio
Diisocyanate−induced asthma
Blood metabolite levels
Gout
Position on chr10 (Mb)

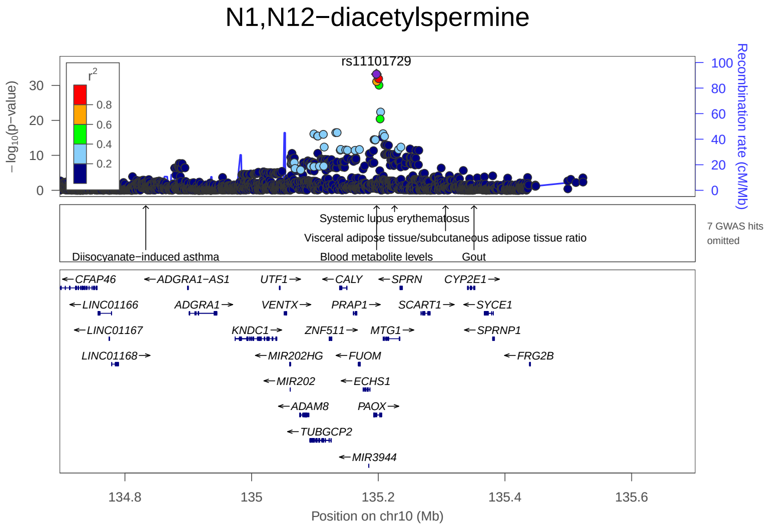
N1,N12−diacetylspermine
rs11101729
Recombination rate (cM/Mb)
7 GWAS hits omitted
Systemic lupus erythematosus
Visceral adipose tissue/subcutaneous adipose tissue ratio
Diisocyanate−induced asthma
Blood metabolite levels
Gout
Position on chr10 (Mb)

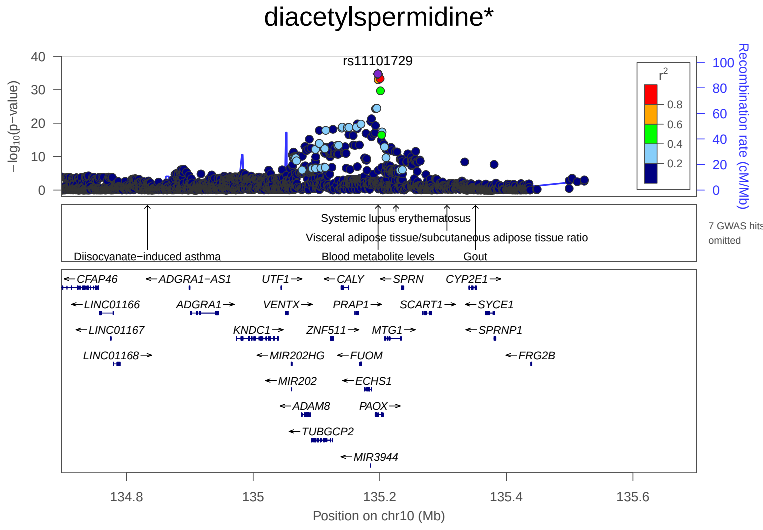
diacetylspermidine*
rs11101729
Recombination rate (cM/Mb)
7 GWAS hits omitted
Systemic lupus erythematosus
Visceral adipose tissue/subcutaneous adipose tissue ratio
Diisocyanate−induced asthma
Blood metabolite levels
Gout
Position on chr10 (Mb)

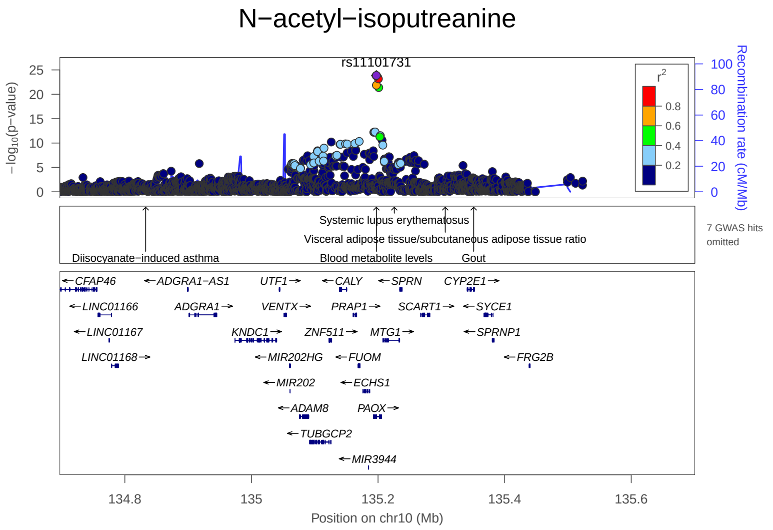
N−acetyl−isoputreanine
rs11101731
7 GWAS hits omitted
Systemic lupus erythematosus
Visceral adipose tissue/subcutaneous adipose tissue ratio
Diisocyanate−induced asthma
Blood metabolite levels
Gout
Position on chr10 (Mb)
Recombination rate (cM/Mb)

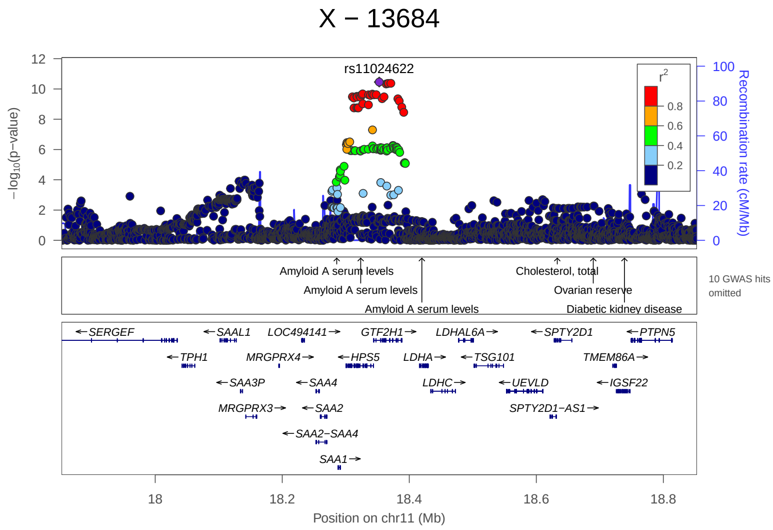
X − 13684
rs11024622
10 GWAS hits omitted
Amyloid A serum levels
Cholesterol, total
Ovarian reserve
Diabetic kidney disease
Position on chr11 (Mb)
Recombination rate (cM/Mb)

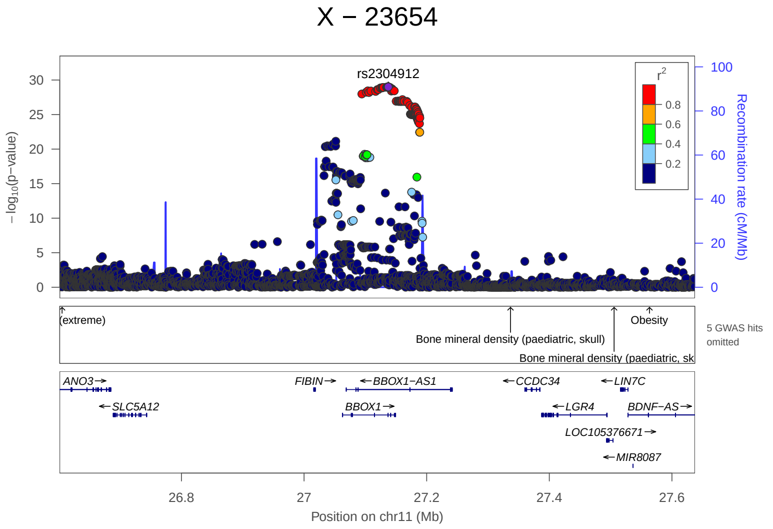
X − 23654
rs2304912
5 GWAS hits omitted
(extreme)
Obesity
Bone mineral density (paediatric, skull)
Position on chr11 (Mb)
Recombination rate (cM/Mb)

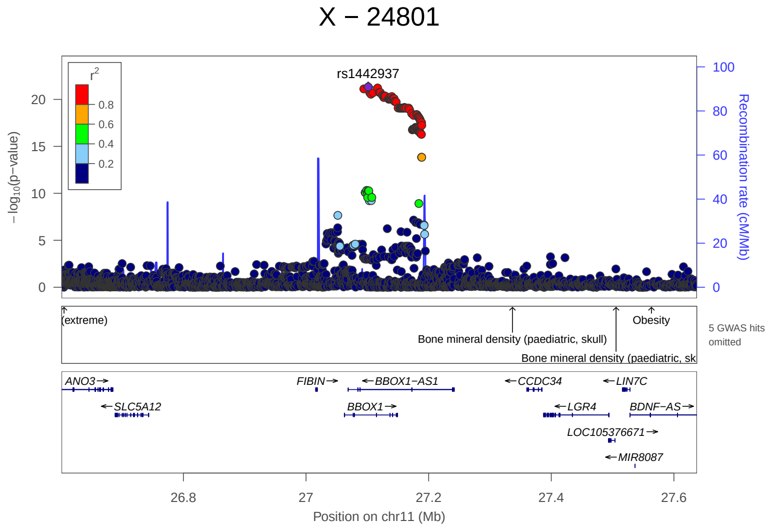
X − 24801
rs1442937
5 GWAS hits omitted
(extreme)
Obesity
Bone mineral density (paediatric, skull)
Position on chr11 (Mb)
Recombination rate (cM/Mb)

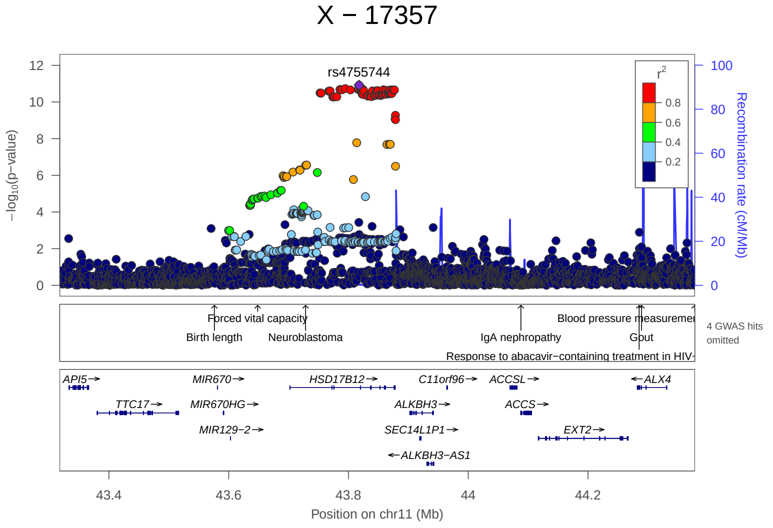
X − 17357
rs4755744
4 GWAS hits omitted
Forced vital capacity
Birth length
Neuroblastoma
IgA nephropathy
Gout
Blood pressure measurement
Response to abacavir−containing treatment in HIV−
Position on chr11 (Mb)
Recombination rate (cM/Mb)

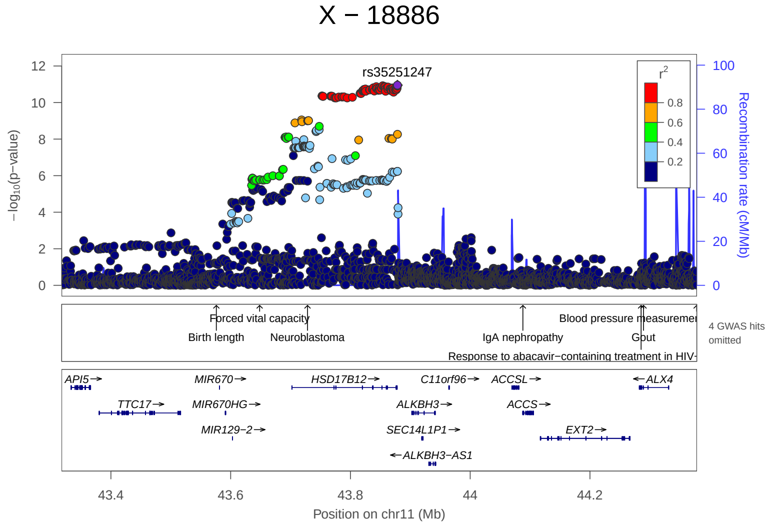
X − 18886
rs35251247
4 GWAS hits omitted
Forced vital capacity
Birth length
Neuroblastoma
IgA nephropathy
Gout
Blood pressure measurement
Response to abacavir−containing treatment in HIV−
Position on chr11 (Mb)
Recombination rate (cM/Mb)

N−acetyl−aspartyl−glutamate (NAAG)

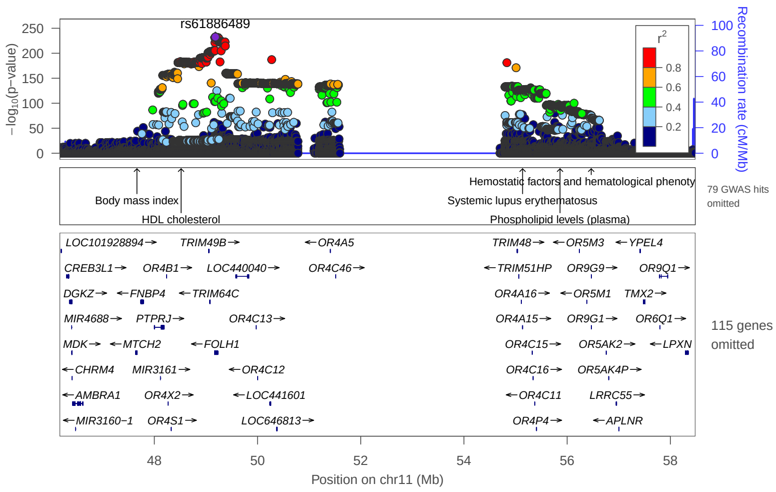

N−acetyl−aspartyl−glutamate (NAAG)

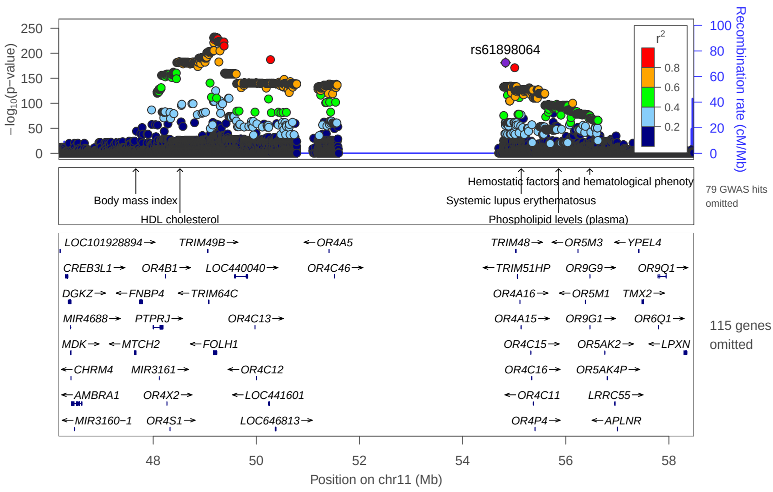

X − 24680

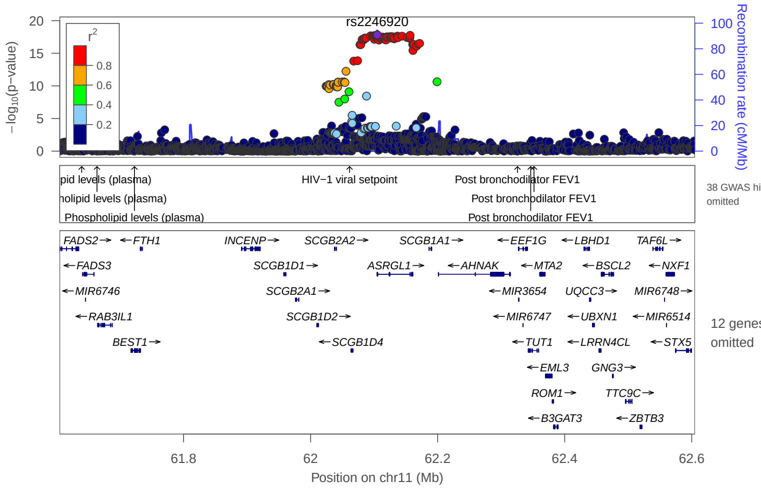

pregnanediol−3−glucuronide

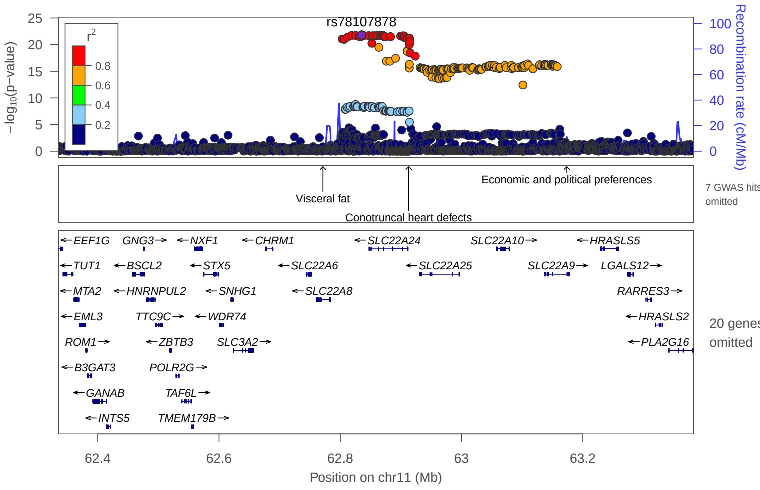

17alpha−hydroxypregnanolone glucuronide

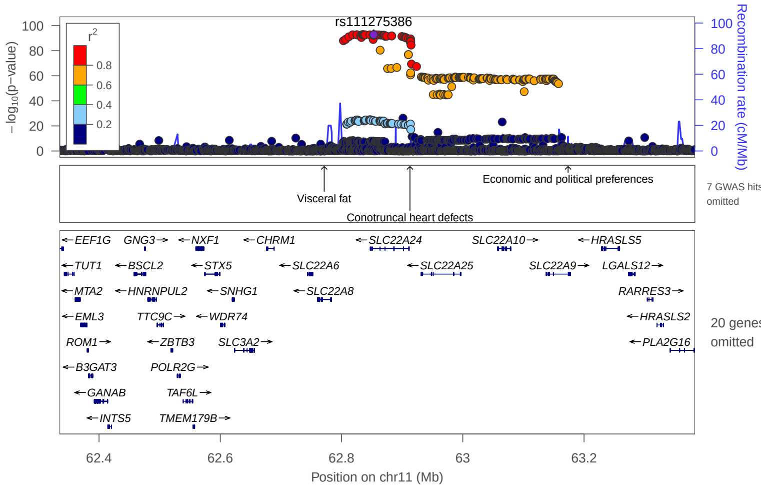

X − 22834

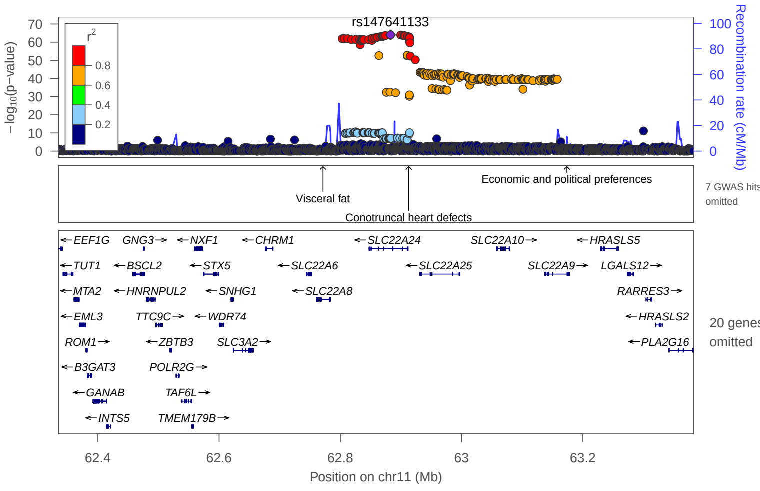

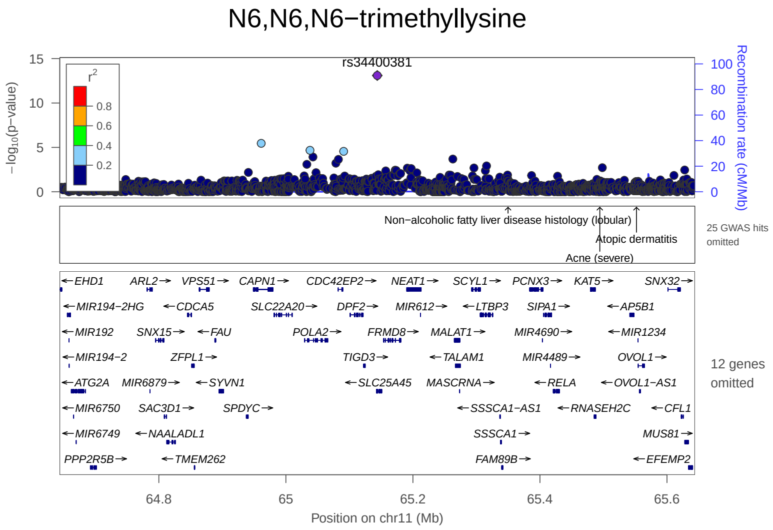
N6,N6,N6−trimethyllysine
rs34400381
−log10(p−value)
Recombination rate (cM/Mb)
Non−alcoholic fatty liver disease histology (lobular)
Atopic dermatitis
Acne (severe)
25 GWAS hits omitted
12 genes omitted
Position on chr11 (Mb)

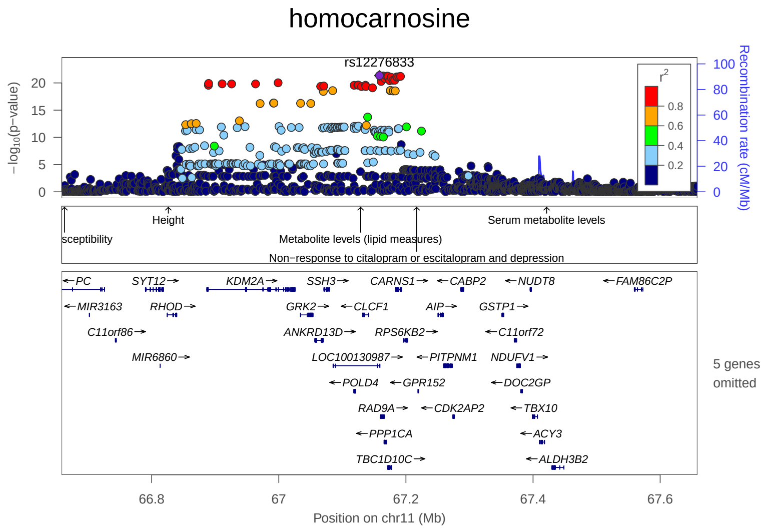
homocarnosine
rs12276833
−log10(p−value)
Recombination rate (cM/Mb)
Height
Serum metabolite levels
Metabolite levels (lipid measures)
Non−response to citalopram or escitalopram and depression
5 genes omitted
Position on chr11 (Mb)

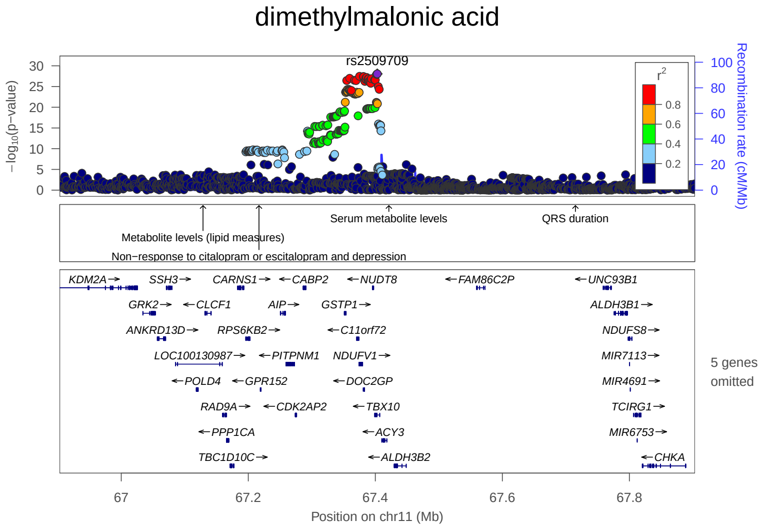
dimethylmalonic acid
rs2509709
−log10(p−value)
Recombination rate (cM/Mb)
Serum metabolite levels
QRS duration
Metabolite levels (lipid measures)
Non−response to citalopram or escitalopram and depression
5 genes omitted
Position on chr11 (Mb)

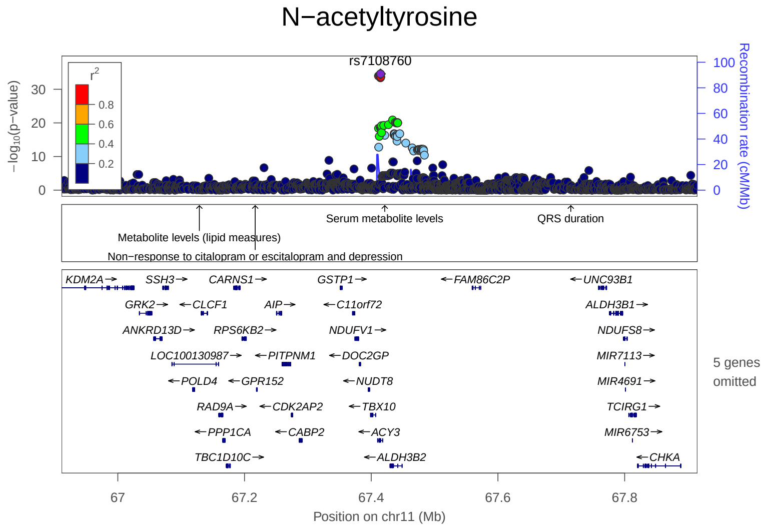
N−acetyltyrosine
rs7108760
−log10(p−value)
Recombination rate (cM/Mb)
Serum metabolite levels
QRS duration
Metabolite levels (lipid measures)
Non−response to citalopram or escitalopram and depression
5 genes omitted
Position on chr11 (Mb)

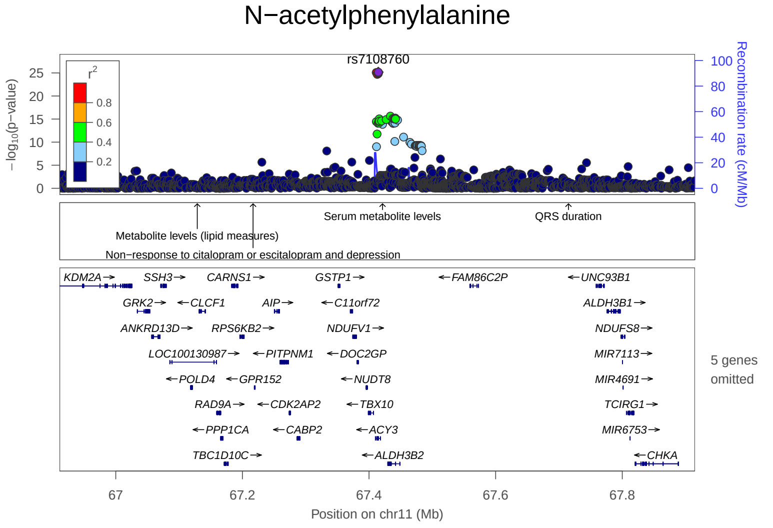
N−acetylphenylalanine
rs7108760
−log10(p−value)
Recombination rate (cM/Mb)
Serum metabolite levels
QRS duration
Metabolite levels (lipid measures)
Non−response to citalopram or escitalopram and depression
5 genes omitted
Position on chr11 (Mb)

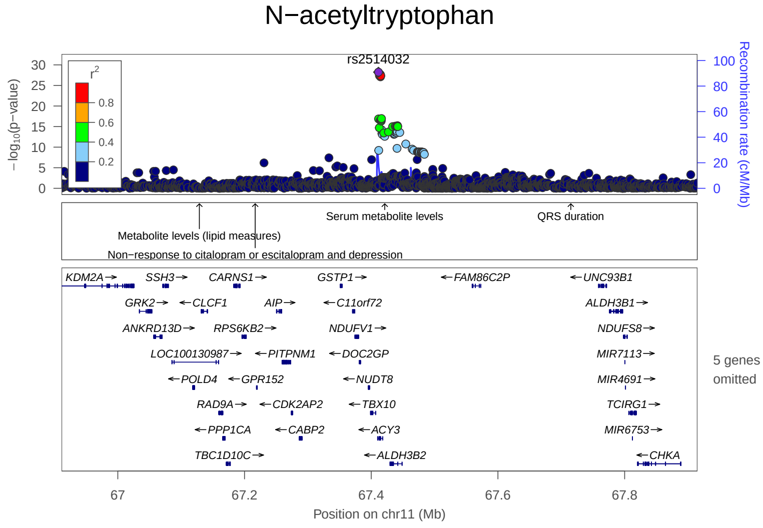
N−acetyltryptophan
rs2514032
−log10(p−value)
Recombination rate (cM/Mb)
Serum metabolite levels
QRS duration
Metabolite levels (lipid measures)
Non−response to citalopram or escitalopram and depression
5 genes omitted
Position on chr11 (Mb)

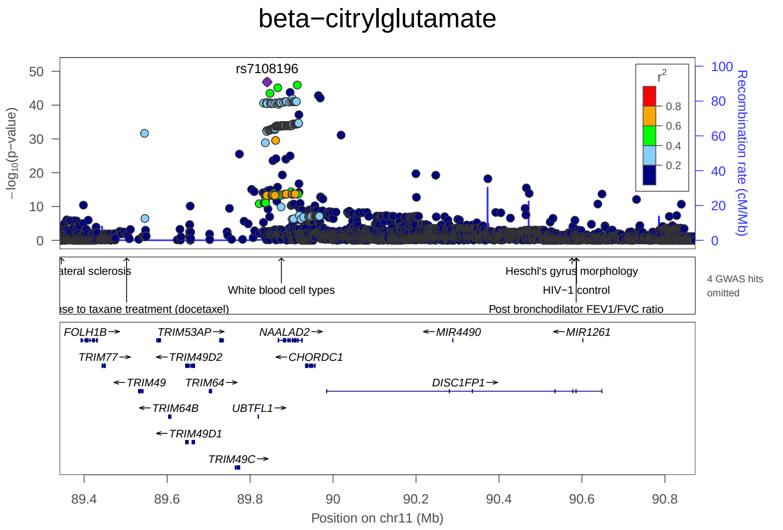
beta−citrylglutamate
rs7108196
−log10(p−value)
Recombination rate (cM/Mb)
White blood cell types
Heschl's gyrus morphology
HIV−1 control
Post bronchodilator FEV1/FVC ratio
4 GWAS hits omitted
FOLH1B
TRIM53AP
NAALAD2
MIR4490
MIR1261
TRIM77
TRIM49D2
CHORDC1
TRIM49
TRIM64
DISC1FP1
TRIM64B
UBTFL1
TRIM49D1
TRIM49C
Position on chr11 (Mb)

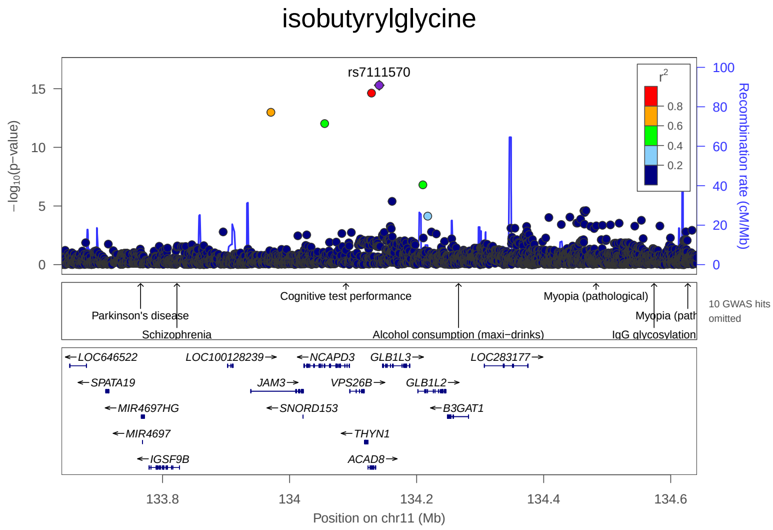
isobutyrylglycine
rs7111570
−log10(p−value)
Recombination rate (cM/Mb)
Parkinson's disease
Schizophrenia
Cognitive test performance
Alcohol consumption (maxi−drinks)
Myopia (pathological)
IgG glycosylation
10 GWAS hits omitted
LOC646522
LOC100128239
NCAPD3
GLB1L3
LOC283177
SPATA19
JAM3
VPS26B
GLB1L2
MIR4697HG
SNORD153
B3GAT1
MIR4697
THYN1
IGSF9B
ACAD8
Position on chr11 (Mb)

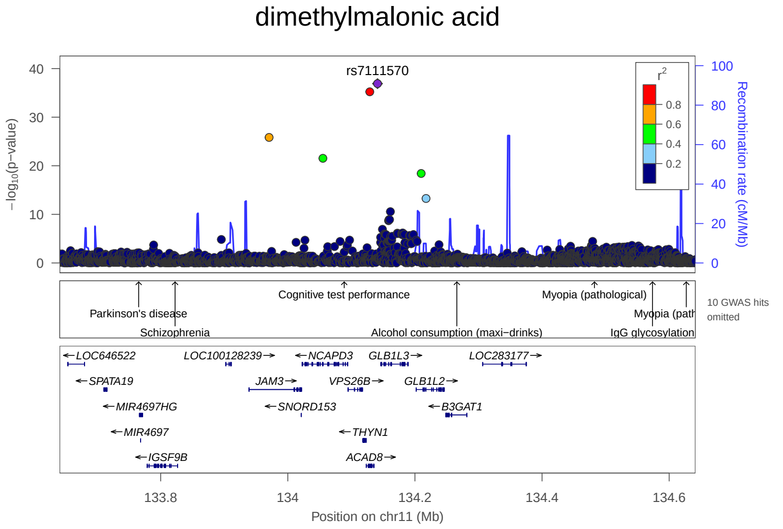
dimethylmalonic acid
rs7111570
−log10(p−value)
Recombination rate (cM/Mb)
Parkinson's disease
Schizophrenia
Cognitive test performance
Alcohol consumption (maxi−drinks)
Myopia (pathological)
IgG glycosylation
10 GWAS hits omitted
LOC646522
LOC100128239
NCAPD3
GLB1L3
LOC283177
SPATA19
JAM3
VPS26B
GLB1L2
MIR4697HG
SNORD153
B3GAT1
MIR4697
THYN1
IGSF9B
ACAD8
Position on chr11 (Mb)

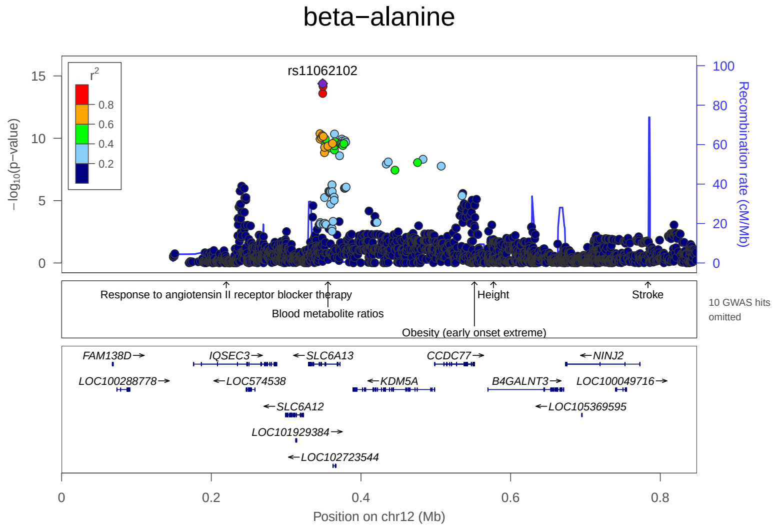
beta−alanine
rs11062102
−log10(p−value)
Recombination rate (cM/Mb)
Response to angiotensin II receptor blocker therapy
Blood metabolite ratios
Height
Obesity (early onset extreme)
Stroke
10 GWAS hits omitted
FAM138D
IQSEC3
SLC6A13
CCDC77
NINJ2
LOC100288778
LOC574538
KDM5A
B4GALNT3
LOC100049716
SLC6A12
LOC105369595
LOC101929384
LOC102723544
Position on chr12 (Mb)

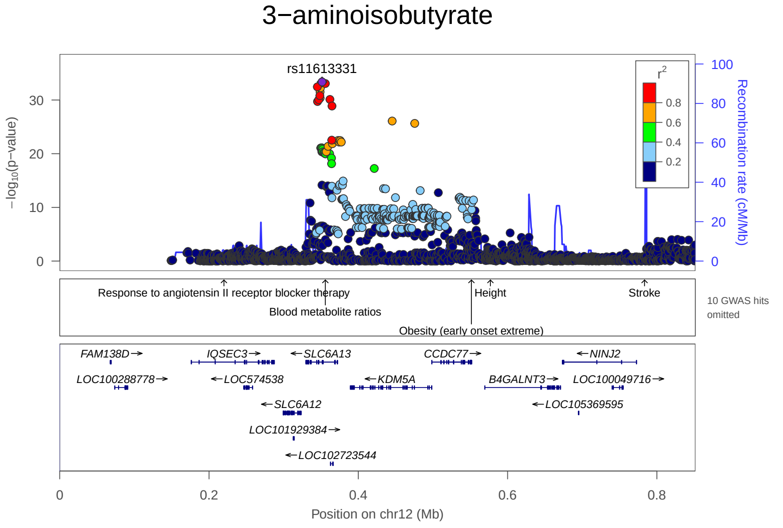
3−aminoisobutyrate
rs11613331
−log10(p−value)
Recombination rate (cM/Mb)
Response to angiotensin II receptor blocker therapy
Blood metabolite ratios
Height
Obesity (early onset extreme)
Stroke
10 GWAS hits omitted
FAM138D
IQSEC3
SLC6A13
CCDC77
NINJ2
LOC100288778
LOC574538
KDM5A
B4GALNT3
LOC100049716
SLC6A12
LOC105369595
LOC101929384
LOC102723544
Position on chr12 (Mb)

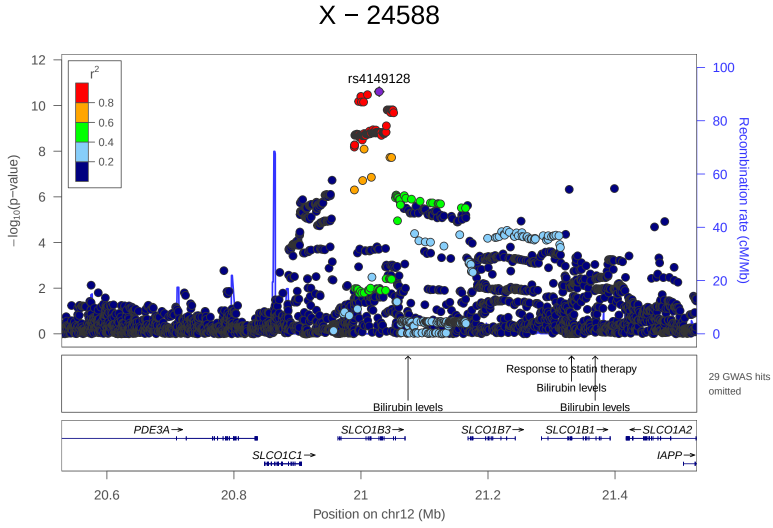
X − 24588
rs4149128
−log10(p−value)
Recombination rate (cM/Mb)
Response to statin therapy
Bilirubin levels
29 GWAS hits omitted
PDE3A
SLCO1B3
SLCO1B7
SLCO1B1
SLCO1A2
SLCO1C1
IAPP
Position on chr12 (Mb)

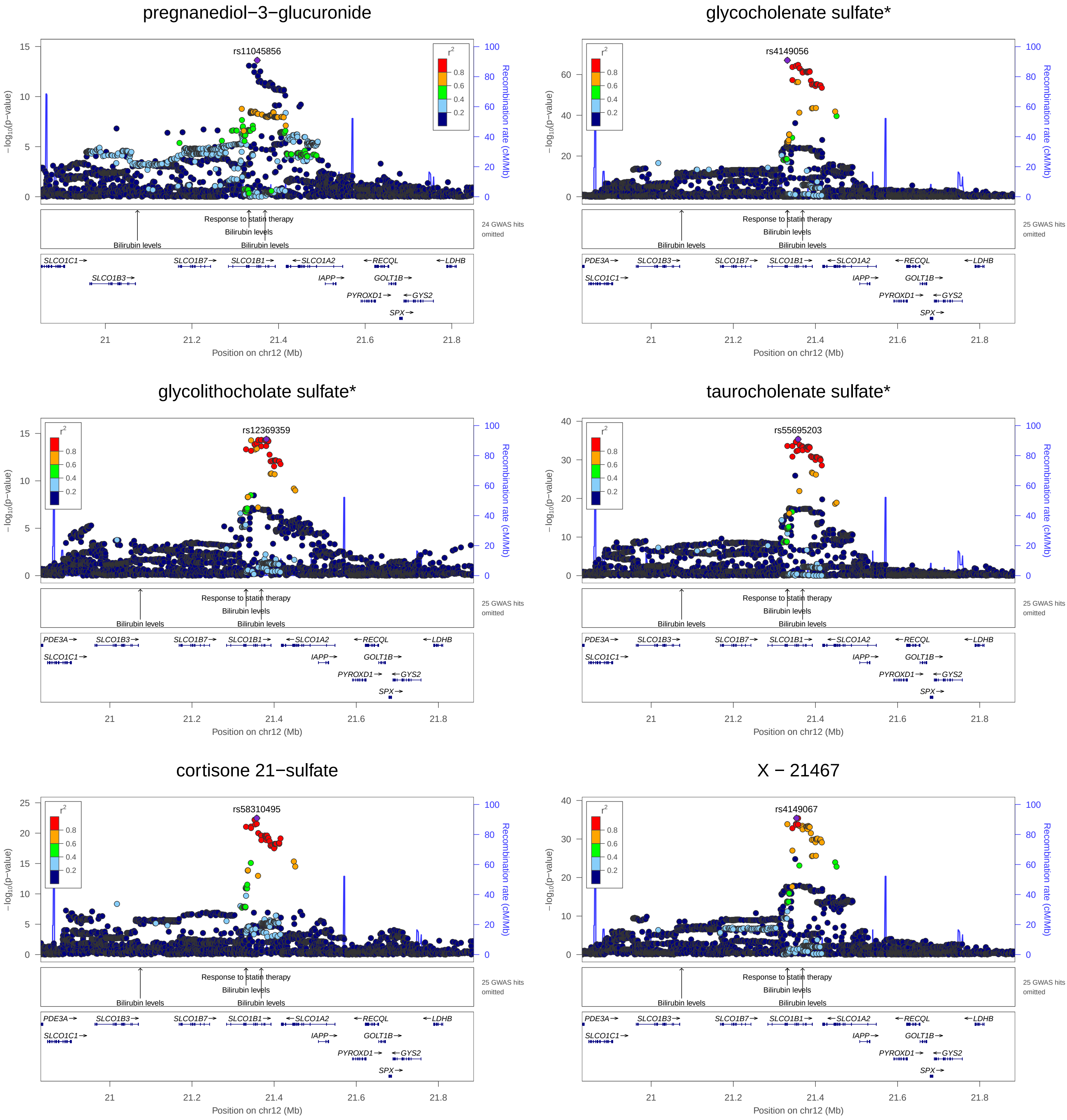
pregnanediol−3−glucuronide
rs11045856
24 GWAS hits omitted
glycocholenate sulfate*
rs4149056
25 GWAS hits omitted
glycolithocholate sulfate*
rs12369359
25 GWAS hits omitted
taurocholenate sulfate*
rs55695203
25 GWAS hits omitted
cortisone 21−sulfate
rs58310495
25 GWAS hits omitted
X − 21467
rs4149067
25 GWAS hits omitted
−log10(p−value)
Recombination rate (cM/Mb)
r2
Response to statin therapy
Bilirubin levels
PDE3A
SLCO1C1
SLCO1B3
SLCO1B7
SLCO1B1
SLCO1A2
IAPP
PYROXD1
RECQL
GOLT1B
GYS2
SPX
LDHB
Position on chr12 (Mb)

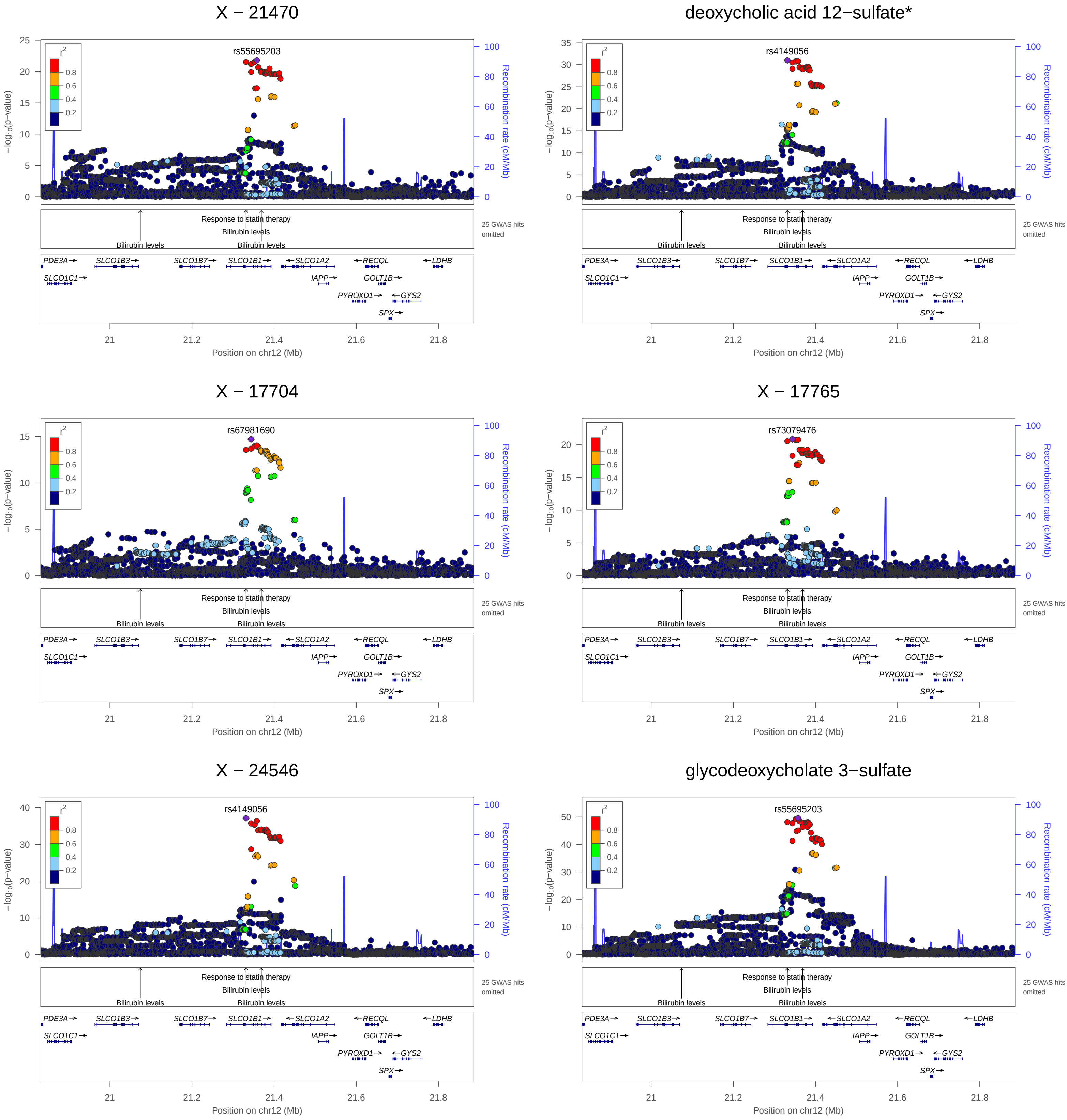
X − 21470
rs55695203
deoxycholic acid 12−sulfate*
rs4149056
X − 17704
rs67981690
X − 17765
rs73079476
X − 24546
rs4149056
glycodeoxycholate 3−sulfate
rs55695203
r2
0.8
0.6
0.4
0.2
−log10(p−value)
Recombination rate (cM/Mb)
Response to statin therapy
Bilirubin levels
Bilirubin levels
Bilirubin levels
25 GWAS hits omitted
PDE3A→
SLCO1B3→
SLCO1B7→
SLCO1B1→
←SLCO1A2
←RECQL
←LDHB
SLCO1C1→
IAPP→
GOLT1B→
PYROXD1→
←GYS2
SPX→
21
21.2
21.4
21.6
21.8
Position on chr12 (Mb)

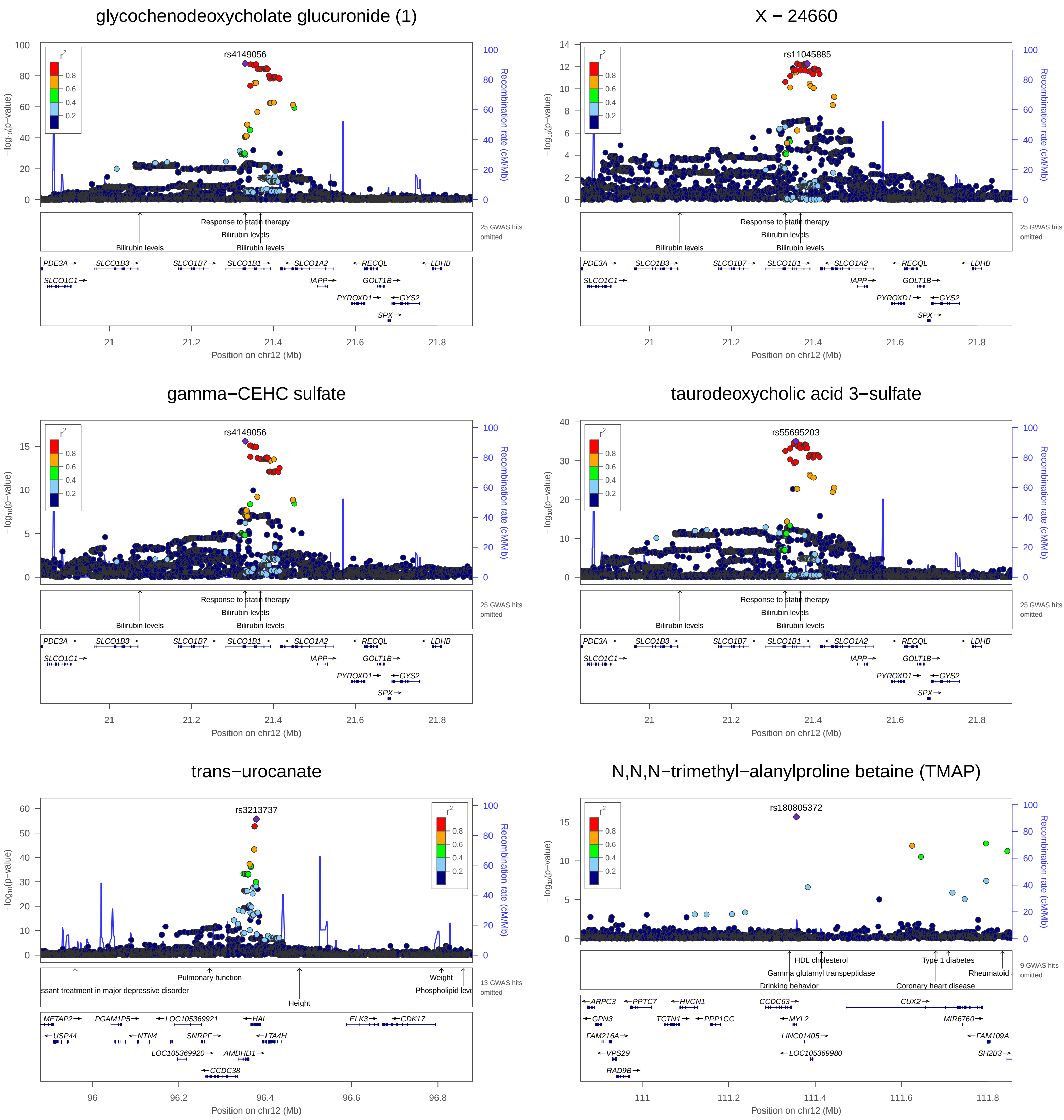
glycochenodeoxycholate glucuronide (1)
rs4149056
X − 24660
rs11045885
gamma−CEHC sulfate
rs4149056
taurodeoxycholic acid 3−sulfate
rs55695203
trans−urocanate
rs3213737
N,N,N−trimethyl−alanylproline betaine (TMAP)
rs180805372
r2
0.8
0.6
0.4
0.2
−log10(p−value)
Recombination rate (cM/Mb)
Response to statin therapy
Bilirubin levels
25 GWAS hits omitted
PDE3A→
SLCO1B3→
SLCO1B7→
SLCO1B1→
←SLCO1A2
←RECQL
←LDHB
SLCO1C1→
IAPP→
GOLT1B→
PYROXD1→
←GYS2
SPX→
Position on chr12 (Mb)
Pulmonary function
ssant treatment in major depressive disorder
Height
Weight
Phospholipid leve
13 GWAS hits omitted
METAP2→
PGAM1P5→
←LOC105369921
←HAL
ELK3→
←CDK17
←USP44
←NTN4
SNRPF→
←LTA4H
LOC105369920→
AMDHD1→
←CCDC38
HDL cholesterol
Gamma glutamyl transpeptidase
Drinking behavior
Type 1 diabetes
Rheumatoid
Coronary heart disease
9 GWAS hits omitted
←ARPC3
←PPTC7
←HVCN1
CCDC63→
CUX2→
←GPN3
TCTN1→
←PPP1CC
←MYL2
MIR6760→
FAM216A→
LINC01405→
←FAM109A
←VPS29
←LOC105369980
SH2B3→
RAD9B→

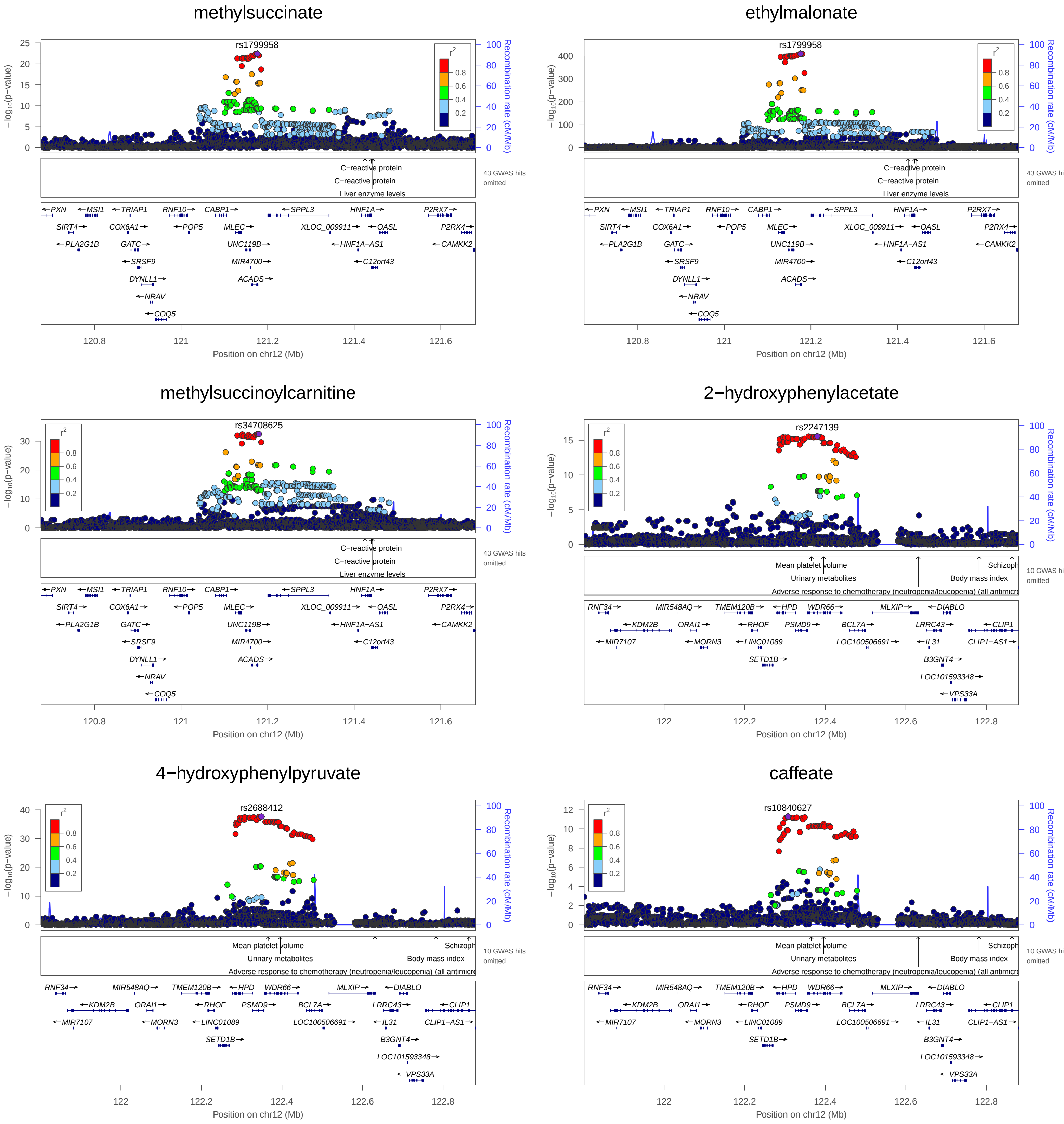

methylsuccinate
ethylmalonate
methylsuccinoylcarnitine
2−hydroxyphenylacetate
4−hydroxyphenylpyruvate
caffeate
rs1799958
rs34708625
rs2247139
rs2688412
rs10840627
−log10(p−value)
Recombination rate (cM/Mb)
r2
0.8
0.6
0.4
0.2
C−reactive protein
C−reactive protein
Liver enzyme levels
43 GWAS hits omitted
Mean platelet volume
Urinary metabolites
Adverse response to chemotherapy (neutropenia/leucopenia) (all antimicro
Body mass index
Schizoph
10 GWAS hits omitted
PXN
MSI1
TRIAP1
RNF10
CABP1
SPPL3
HNF1A
P2RX7
SIRT4
COX6A1
POP5
MLEC
XLOC_009911
OASL
P2RX4
PLA2G1B
GATC
UNC119B
HNF1A−AS1
CAMKK2
SRSF9
MIR4700
C12orf43
DYNLL1
ACADS
NRAV
COQ5
RNF34
MIR548AQ
TMEM120B
HPD
WDR66
MLXIP
DIABLO
KDM2B
ORAI1
RHOF
PSMD9
BCL7A
LRRC43
CLIP1
MIR7107
MORN3
LINC01089
LOC100506691
IL31
CLIP1−AS1
SETD1B
B3GNT4
LOC101593348
VPS33A
Position on chr12 (Mb)

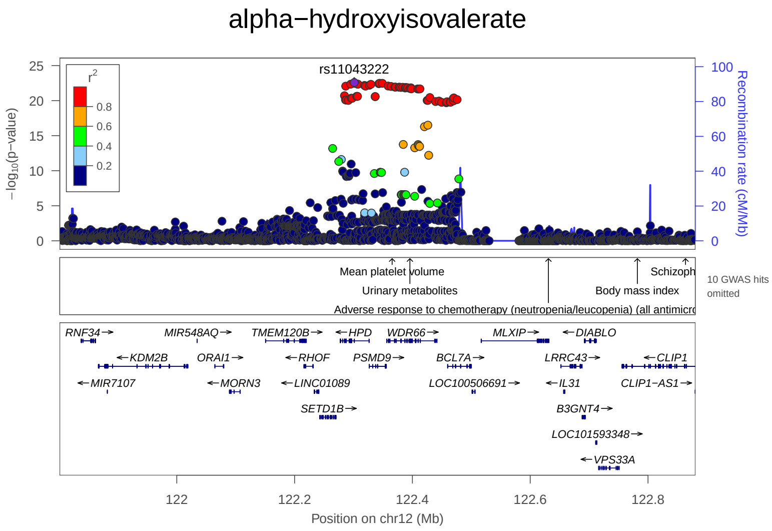

alpha−hydroxyisovalerate
rs11043222
−log10(p−value)
Recombination rate (cM/Mb)
r2
0.8
0.6
0.4
0.2
Mean platelet volume
Urinary metabolites
Body mass index
Schizoph
Adverse response to chemotherapy (neutropenia/leucopenia) (all antimicro
10 GWAS hits omitted
RNF34
MIR548AQ
TMEM120B
HPD
WDR66
MLXIP
DIABLO
KDM2B
ORAI1
RHOF
PSMD9
BCL7A
LRRC43
CLIP1
MIR7107
MORN3
LINC01089
LOC100506691
IL31
CLIP1−AS1
SETD1B
B3GNT4
LOC101593348
VPS33A
Position on chr12 (Mb)

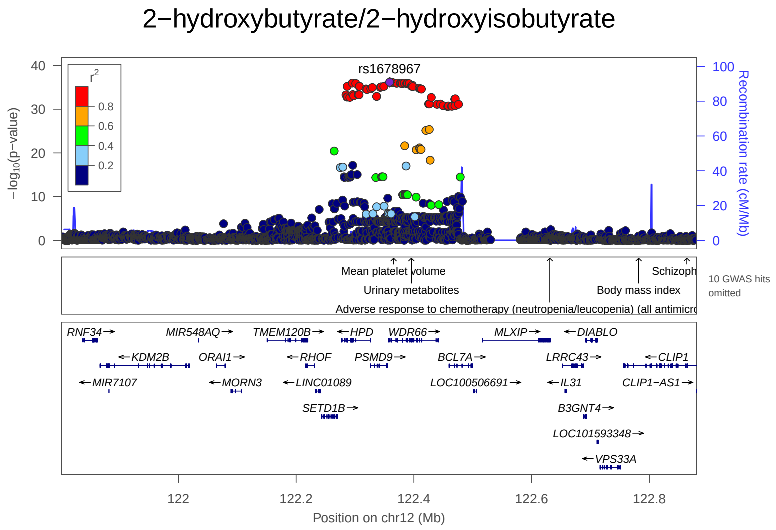

2−hydroxybutyrate/2−hydroxyisobutyrate
rs1678967
−log10(p−value)
Recombination rate (cM/Mb)
r2
Mean platelet volume
Urinary metabolites
Body mass index
Schizoph
Adverse response to chemotherapy (neutropenia/leucopenia) (all antimicro
10 GWAS hits omitted
RNF34
MIR548AQ
TMEM120B
HPD
WDR66
MLXIP
DIABLO
KDM2B
ORAI1
RHOF
PSMD9
BCL7A
LRRC43
CLIP1
MIR7107
MORN3
LINC01089
LOC100506691
IL31
CLIP1−AS1
SETD1B
B3GNT4
LOC101593348
VPS33A
Position on chr12 (Mb)

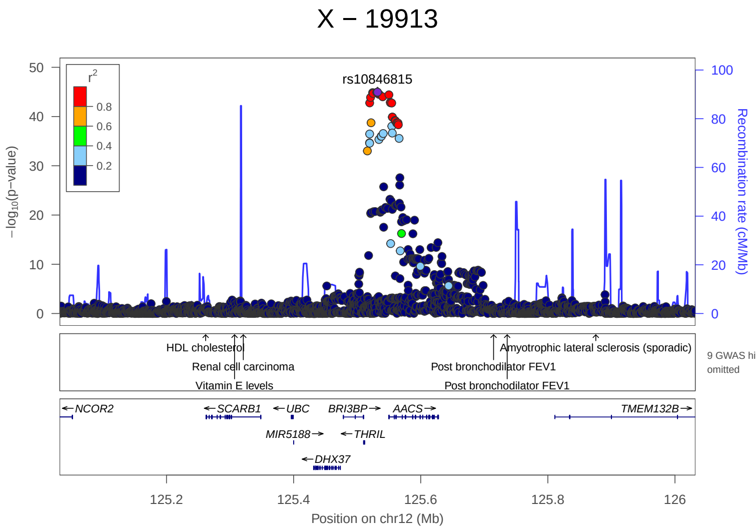

X − 19913
rs10846815
−log10(p−value)
Recombination rate (cM/Mb)
r2
HDL cholesterol
Renal cell carcinoma
Vitamin E levels
Amyotrophic lateral sclerosis (sporadic)
Post bronchodilator FEV1
Post bronchodilator FEV1
9 GWAS hits omitted
NCOR2
SCARB1
UBC
BRI3BP
AACS
TMEM132B
MIR5188
THRIL
DHX37
Position on chr12 (Mb)

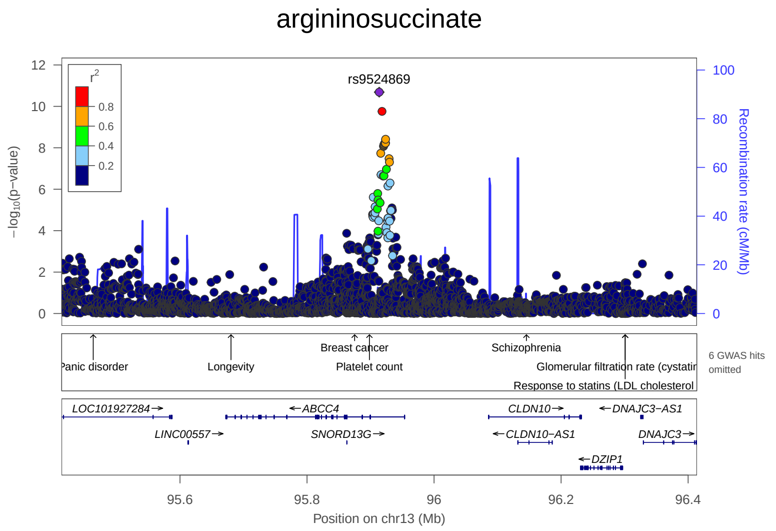

argininosuccinate
rs9524869
−log10(p−value)
Recombination rate (cM/Mb)
r2
Panic disorder
Longevity
Breast cancer
Platelet count
Schizophrenia
Glomerular filtration rate (cystatin
Response to statins (LDL cholesterol
6 GWAS hits omitted
LOC101927284
ABCC4
CLDN10
DNAJC3−AS1
LINC00557
SNORD13G
CLDN10−AS1
DNAJC3
DZIP1
Position on chr13 (Mb)

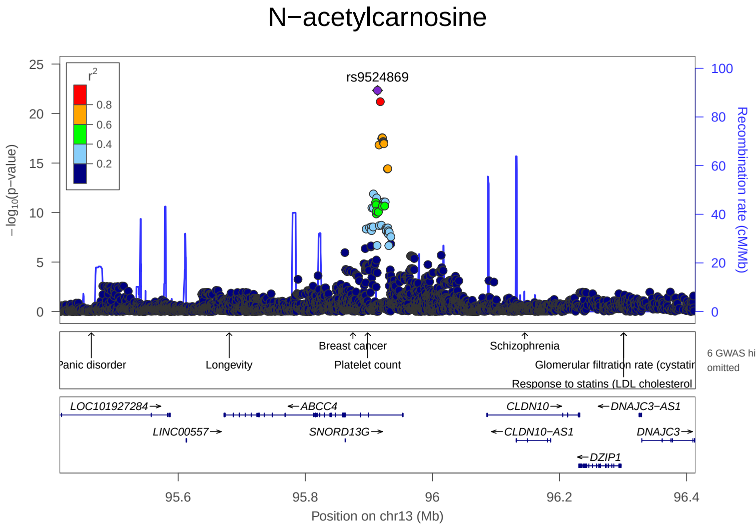

N−acetylcarnosine
rs9524869
−log10(p−value)
Recombination rate (cM/Mb)
r2
Panic disorder
Longevity
Breast cancer
Platelet count
Schizophrenia
Glomerular filtration rate (cystatin
Response to statins (LDL cholesterol
6 GWAS hits omitted
LOC101927284
ABCC4
CLDN10
DNAJC3−AS1
LINC00557
SNORD13G
CLDN10−AS1
DNAJC3
DZIP1
Position on chr13 (Mb)

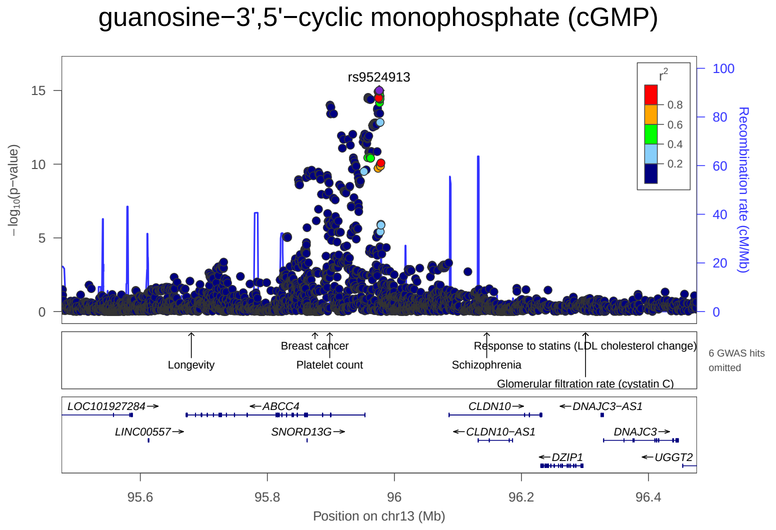

guanosine−3',5'−cyclic monophosphate (cGMP)
rs9524913
−log10(p−value)
Recombination rate (cM/Mb)
r2
Longevity
Breast cancer
Platelet count
Response to statins (LDL cholesterol change)
Schizophrenia
Glomerular filtration rate (cystatin C)
6 GWAS hits omitted
LOC101927284
ABCC4
CLDN10
DNAJC3−AS1
LINC00557
SNORD13G
CLDN10−AS1
DNAJC3
DZIP1
UGGT2
Position on chr13 (Mb)

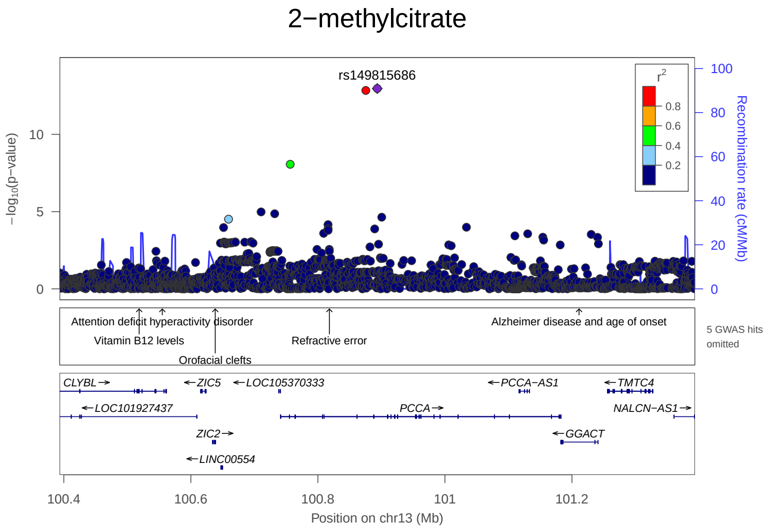
2−methylcitrate
rs149815686
Position on chr13 (Mb)
Attention deficit hyperactivity disorder
Vitamin B12 levels
Orofacial clefts
Refractive error
Alzheimer disease and age of onset
5 GWAS hits omitted

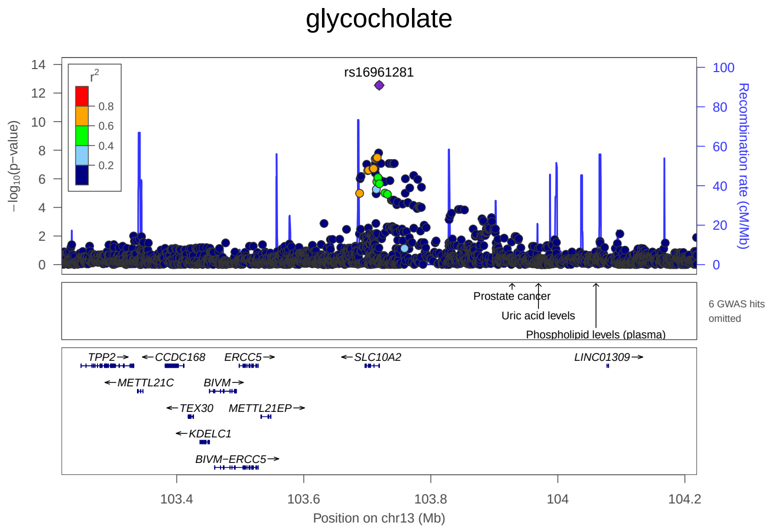
glycocholate
rs16961281
Position on chr13 (Mb)
Prostate cancer
Uric acid levels
Phospholipid levels (plasma)
6 GWAS hits omitted

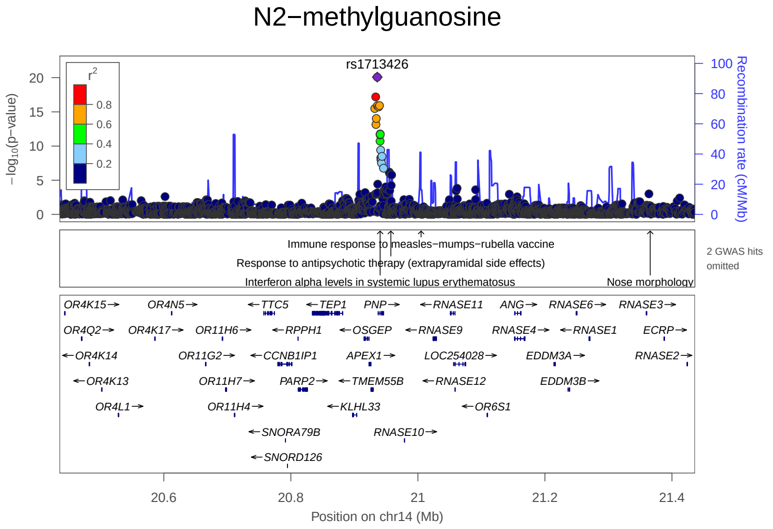
N2−methylguanosine
rs1713426
Position on chr14 (Mb)
Immune response to measles−mumps−rubella vaccine
Response to antipsychotic therapy (extrapyramidal side effects)
Interferon alpha levels in systemic lupus erythematosus
Nose morphology
2 GWAS hits omitted

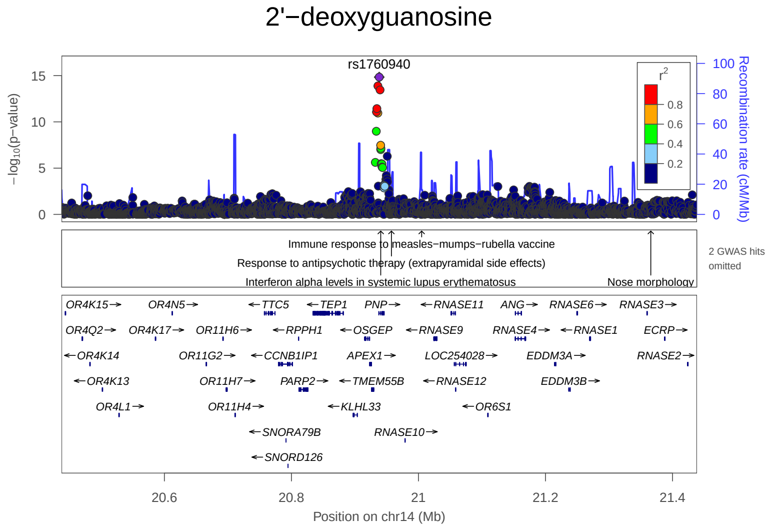
2'−deoxyguanosine
rs1760940
Position on chr14 (Mb)
Immune response to measles−mumps−rubella vaccine
Response to antipsychotic therapy (extrapyramidal side effects)
Interferon alpha levels in systemic lupus erythematosus
Nose morphology
2 GWAS hits omitted

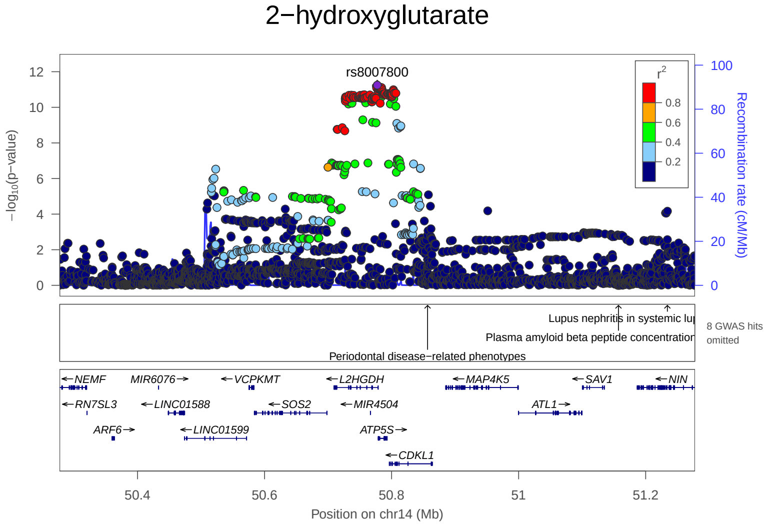
2−hydroxyglutarate
rs8007800
Position on chr14 (Mb)
Periodontal disease−related phenotypes
8 GWAS hits omitted

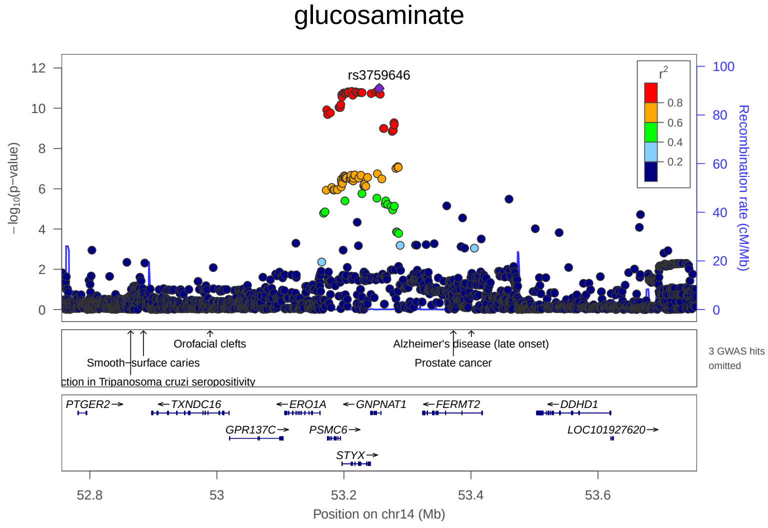
glucosaminate
rs3759646
Position on chr14 (Mb)
Orofacial clefts
Alzheimer's disease (late onset)
Prostate cancer
3 GWAS hits omitted

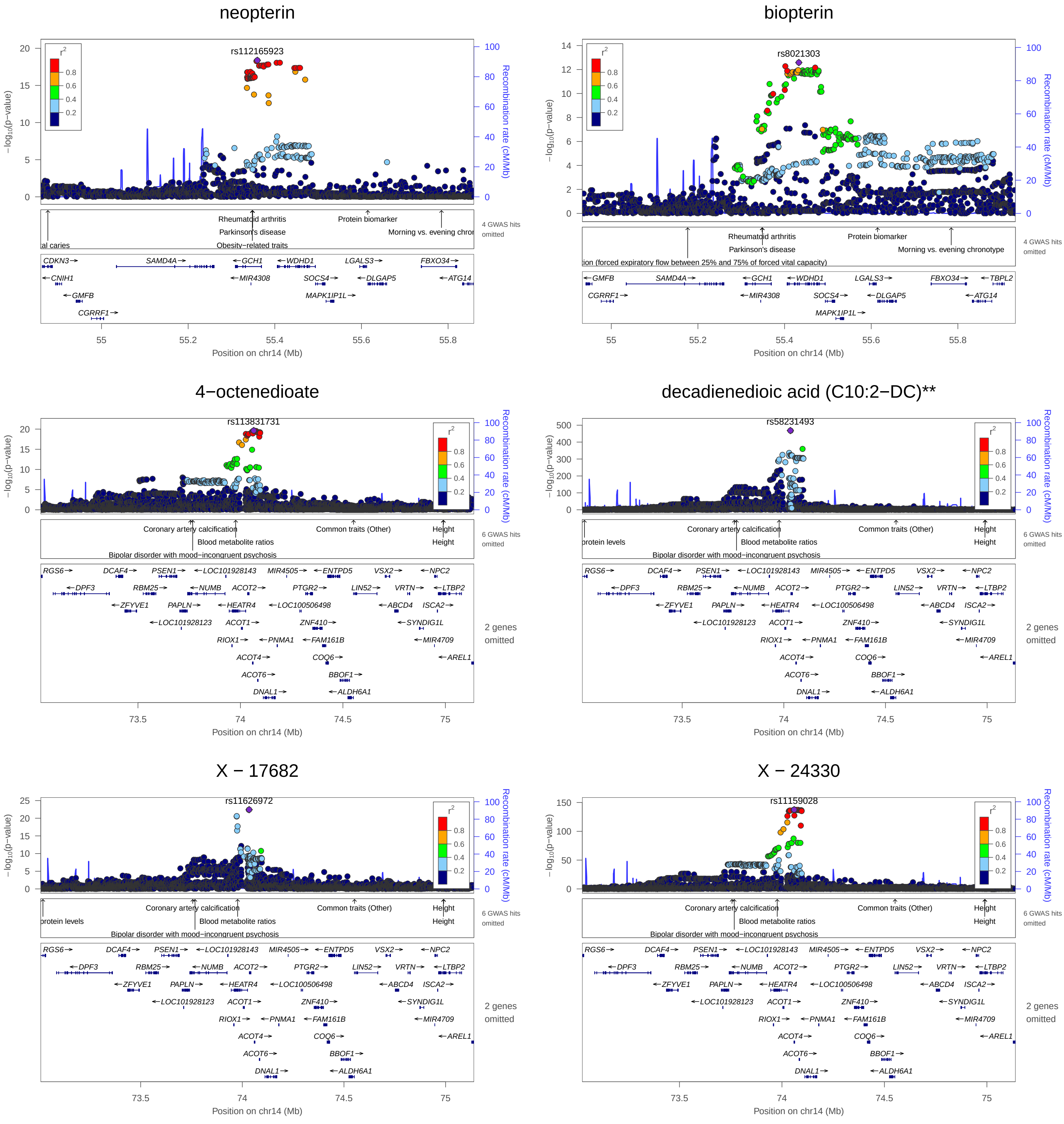

neopterin
rs112165923
biopterin
rs8021303
4−octenedioate
rs113831731
decadienedioic acid (C10:2−DC)**
rs58231493
X − 17682
rs11626972
X − 24330
rs11159028
−log10(p−value)
Recombination rate (cM/Mb)
r2
Position on chr14 (Mb)
4 GWAS hits omitted
6 GWAS hits omitted
2 genes omitted
Rheumatoid arthritis
Parkinson's disease
Obesity−related traits
Protein biomarker
Morning vs. evening chronotype
Coronary artery calcification
Blood metabolite ratios
Common traits (Other)
Height
Bipolar disorder with mood−incongruent psychosis
CDKN3 CNIH1 GMFB CGRRF1 SAMD4A GCH1 MIR4308 WDHD1 SOCS4 MAPK1IP1L LGALS3 DLGAP5 FBXO34 ATG14 TBPL2
RGS6 DPF3 DCAF4 ZFYVE1 RBM25 PSEN1 PAPLN LOC101928123 NUMB HEATR4 ACOT1 ACOT2 RIOX1 ACOT4 ACOT6 DNAL1 PNMA1 MIR4505 LOC100506498 PTGR2 ZNF410 FAM161B COQ6 BBOF1 ALDH6A1 ENTPD5 LIN52 VSX2 ABCD4 VRTN SYNDIG1L ISCA2 NPC2 LTBP2 MIR4709 AREL1

X − 24588

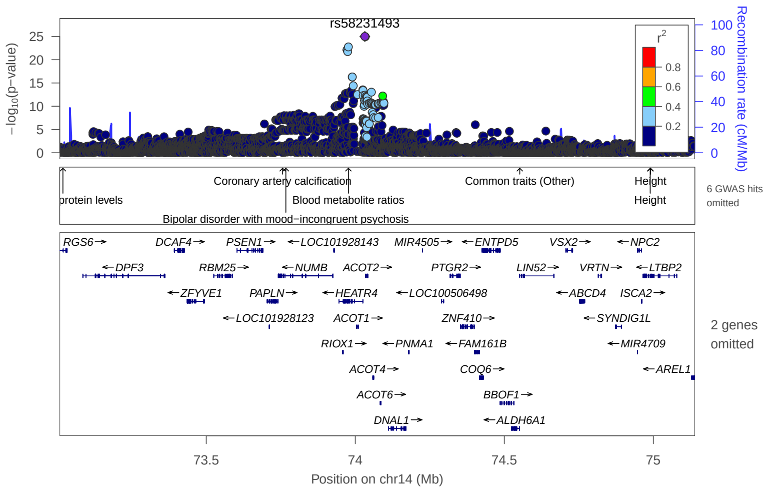

X − 24799

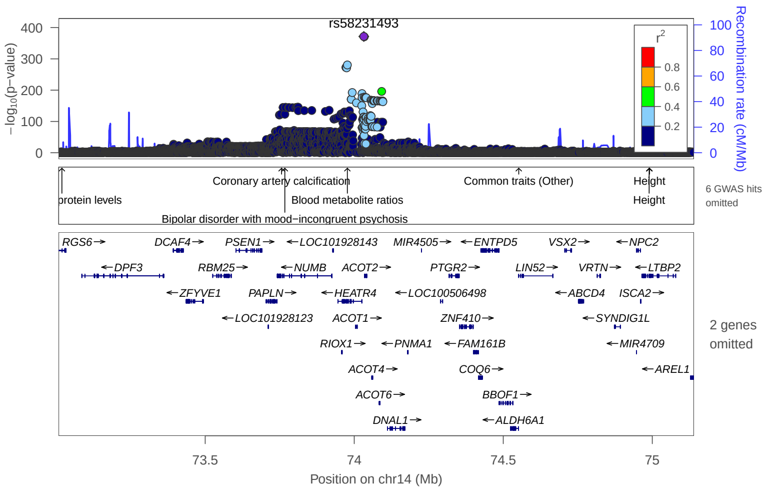

isocaproylglutamine

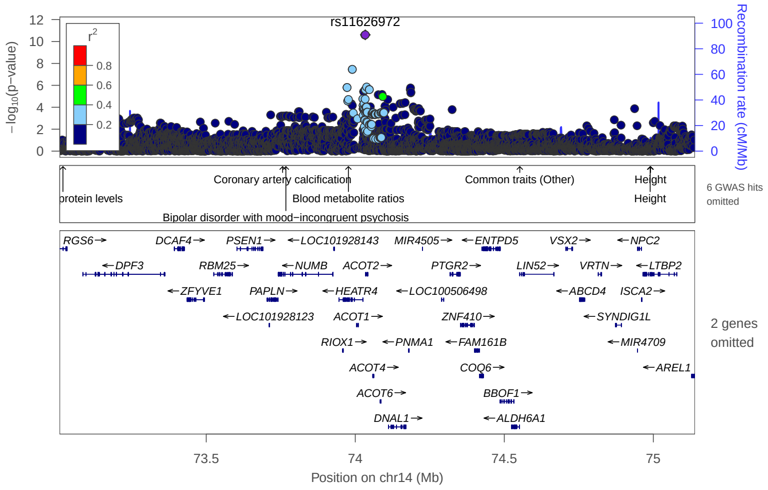

nonenedioate (C9:1−DC)*

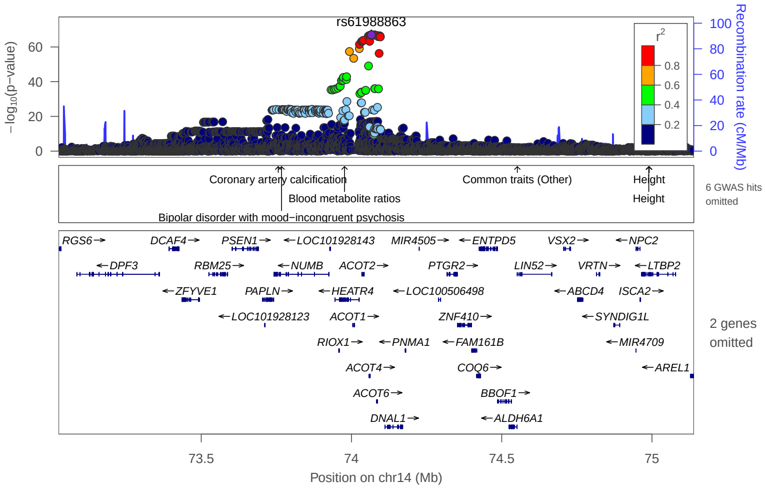

glucuronide of C10H18O2 (12)*

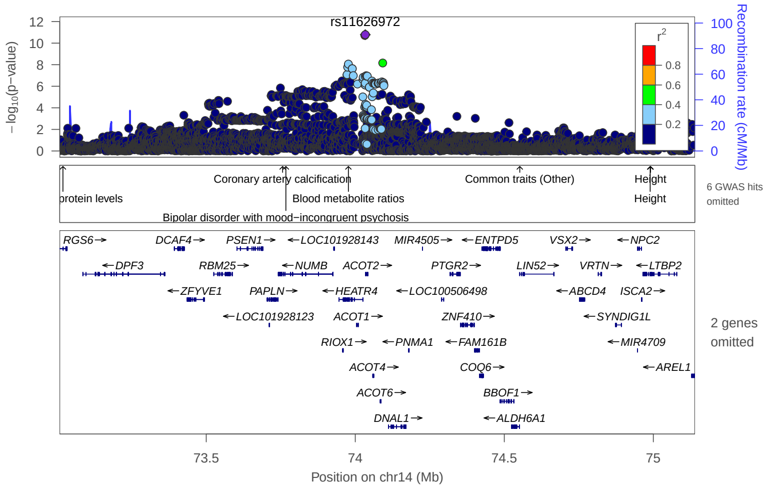

X − 24519

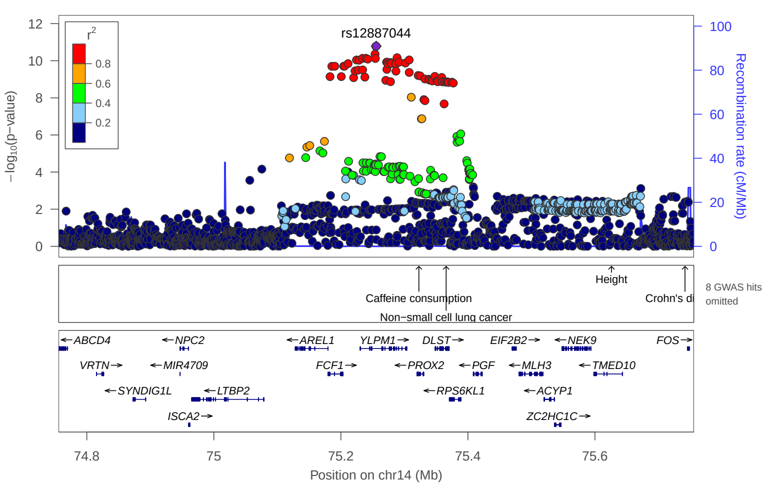

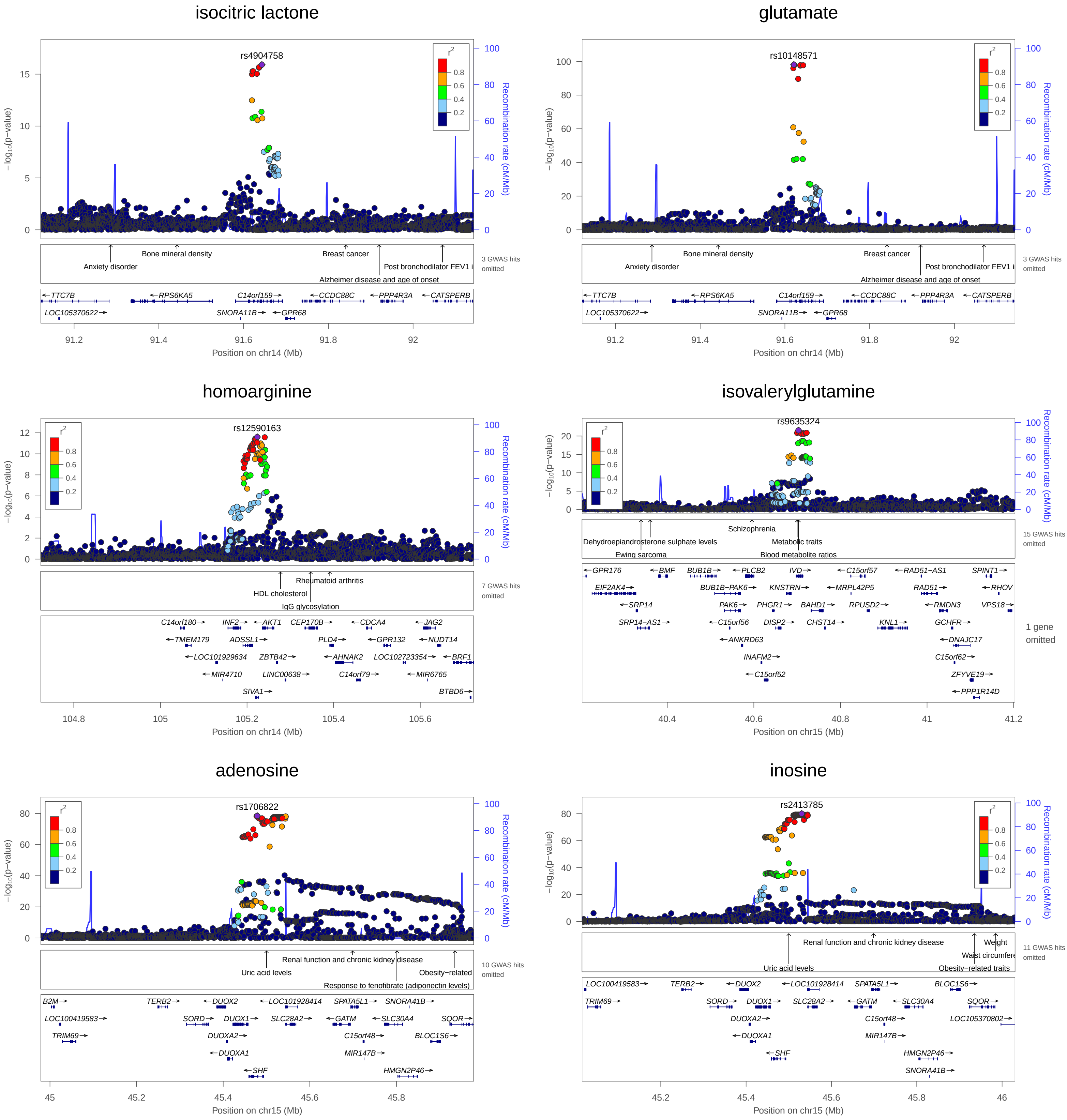

isocitric lactone
rs4904758
Anxiety disorder
Bone mineral density
Breast cancer
Post bronchodilator FEV1
Alzheimer disease and age of onset
3 GWAS hits omitted
TTC7B
RPS6KA5
C14orf159
CCDC88C
PPP4R3A
CATSPERB
LOC105370622
SNORA11B
GPR68
Position on chr14 (Mb)
glutamate
rs10148571
Position on chr14 (Mb)
homoarginine
rs12590163
Rheumatoid arthritis
HDL cholesterol
IgG glycosylation
7 GWAS hits omitted
C14orf180
INF2
AKT1
CEP170B
CDCA4
JAG2
TMEM179
ADSSL1
PLD4
GPR132
NUDT14
LOC101929634
ZBTB42
AHNAK2
LOC102723354
BRF1
MIR4710
LINC00638
C14orf79
MIR6765
SIVA1
BTBD6
Position on chr14 (Mb)
isovalerylglutamine
rs9635324
Schizophrenia
Dehydroepiandrosterone sulphate levels
Metabolic traits
Ewing sarcoma
Blood metabolite ratios
15 GWAS hits omitted
1 gene omitted
Position on chr15 (Mb)
adenosine
rs1706822
Renal function and chronic kidney disease
Uric acid levels
Obesity−related
Response to fenofibrate (adiponectin levels)
10 GWAS hits omitted
Position on chr15 (Mb)
inosine
rs2413785
Renal function and chronic kidney disease
Weight
Waist circumference
Uric acid levels
Obesity−related traits
11 GWAS hits omitted
Position on chr15 (Mb)
−log10(p−value)
Recombination rate (cM/Mb)
r2

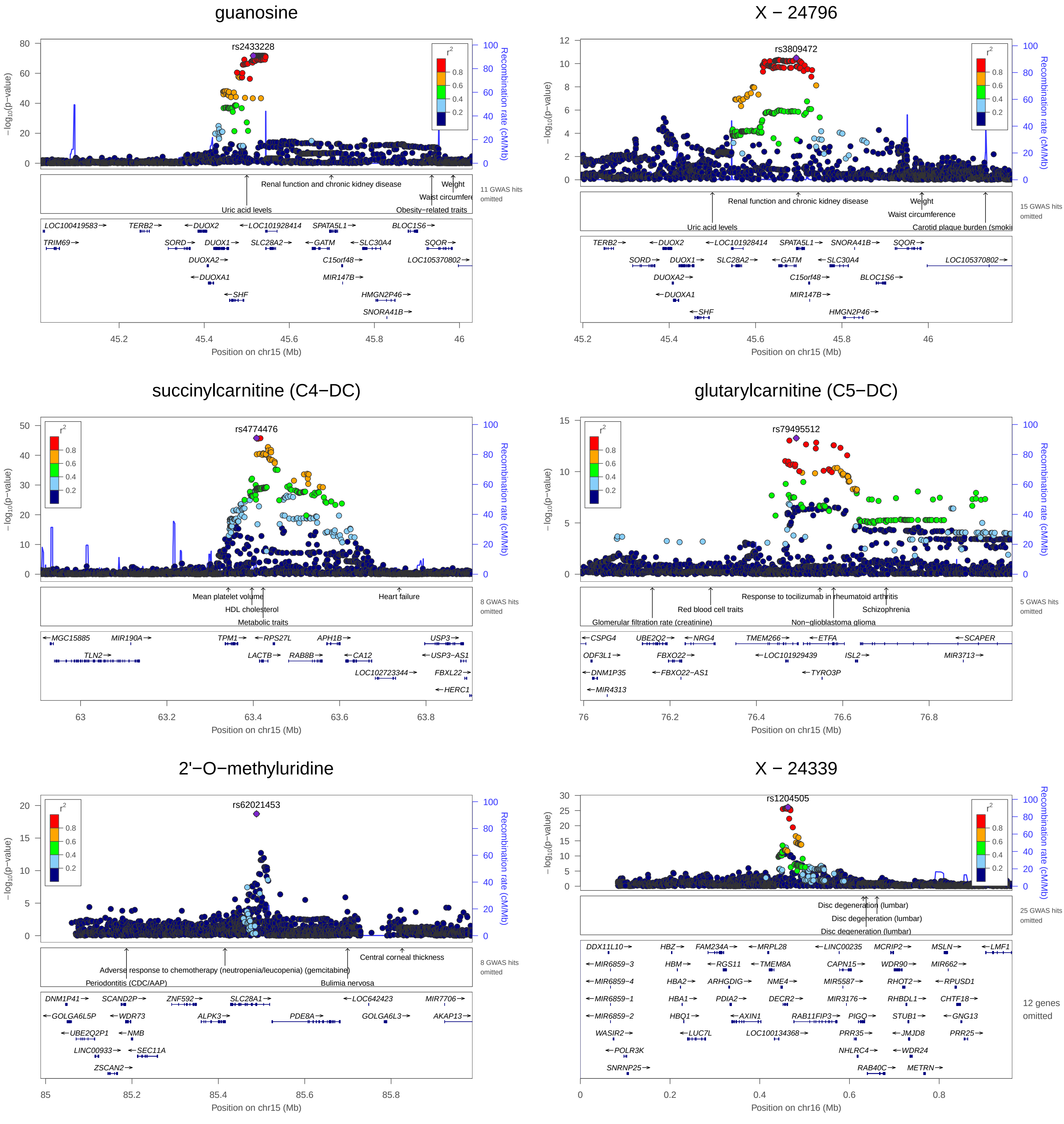
guanosine
rs2433228
Renal function and chronic kidney disease
Weight
Waist circumference
Uric acid levels
Obesity−related traits
11 GWAS hits omitted
LOC100419583 TERB2 DUOX2 LOC101928414 SPATA5L1 BLOC1S6
TRIM69 SORD DUOX1 SLC28A2 GATM SLC30A4 SQOR
DUOXA2 C15orf48 LOC105370802
DUOXA1 MIR147B
SHF HMGN2P46
SNORA41B
Position on chr15 (Mb)
X − 24796
rs3809472
Renal function and chronic kidney disease
Weight
Waist circumference
Uric acid levels
Carotid plaque burden (smoki
15 GWAS hits omitted
TERB2 DUOX2 LOC101928414 SPATA5L1 SNORA41B SQOR
SORD DUOX1 SLC28A2 GATM SLC30A4 LOC105370802
DUOXA2 C15orf48 BLOC1S6
DUOXA1 MIR147B
SHF HMGN2P46
Position on chr15 (Mb)
succinylcarnitine (C4−DC)
rs4774476
Mean platelet volume
HDL cholesterol
Metabolic traits
Heart failure
8 GWAS hits omitted
MGC15885 MIR190A TPM1 RPS27L APH1B USP3
TLN2 LACTB RAB8B CA12 USP3−AS1
LOC102723344 FBXL22
HERC1
Position on chr15 (Mb)
glutarylcarnitine (C5−DC)
rs79495512
Response to tocilizumab in rheumatoid arthritis
Red blood cell traits
Schizophrenia
Glomerular filtration rate (creatinine)
Non−glioblastoma glioma
5 GWAS hits omitted
CSPG4 UBE2Q2 NRG4 TMEM266 ETFA SCAPER
ODF3L1 FBXO22 LOC101929439 ISL2 MIR3713
DNM1P35 FBXO22−AS1 TYRO3P
MIR4313
Position on chr15 (Mb)
2'−O−methyluridine
rs62021453
Central corneal thickness
Adverse response to chemotherapy (neutropenia/leucopenia) (gemcitabine)
Periodontitis (CDC/AAP)
Bulimia nervosa
8 GWAS hits omitted
DNM1P41 SCAND2P ZNF592 SLC28A1 LOC642423 MIR7706
GOLGA6L5P WDR73 ALPK3 PDE8A GOLGA6L3 AKAP13
UBE2Q2P1 NMB
LINC00933 SEC11A
ZSCAN2
Position on chr15 (Mb)
X − 24339
rs1204505
Disc degeneration (lumbar)
Disc degeneration (lumbar)
Disc degeneration (lumbar)
25 GWAS hits omitted
DDX11L10 HBZ FAM234A MRPL28 LINC00235 MCRIP2 MSLN LMF1
MIR6859−3 HBM RGS11 TMEM8A CAPN15 WDR90 MIR662
MIR6859−4 HBA2 ARHGDIG NME4 MIR5587 RHOT2 RPUSD1
MIR6859−1 HBA1 PDIA2 DECR2 MIR3176 RHBDL1 CHTF18
MIR6859−2 HBQ1 AXIN1 RAB11FIP3 PIGQ STUB1 GNG13
WASIR2 LUC7L LOC100134368 PRR35 JMJD8 PRR25
POLR3K NHLRC4 WDR24
SNRNP25 RAB40C METRN
12 genes omitted
Position on chr16 (Mb)
−log10(p−value)
Recombination rate (cM/Mb)
r2

3−(3−hydroxyphenyl)propionate

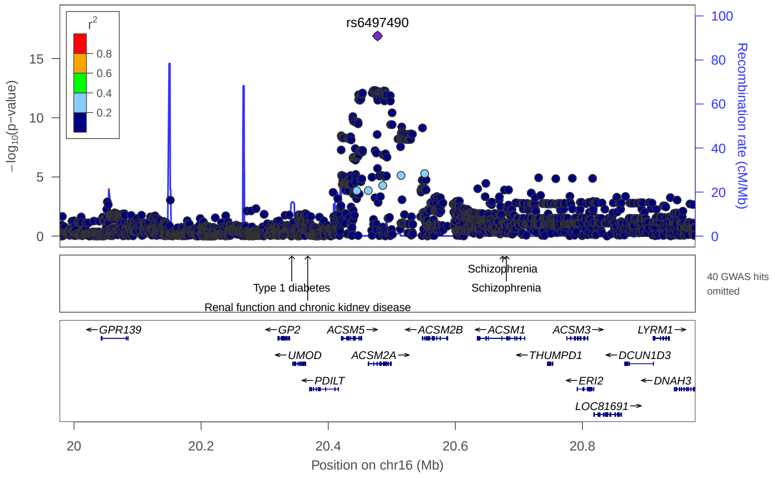

indoleacetylglutamine

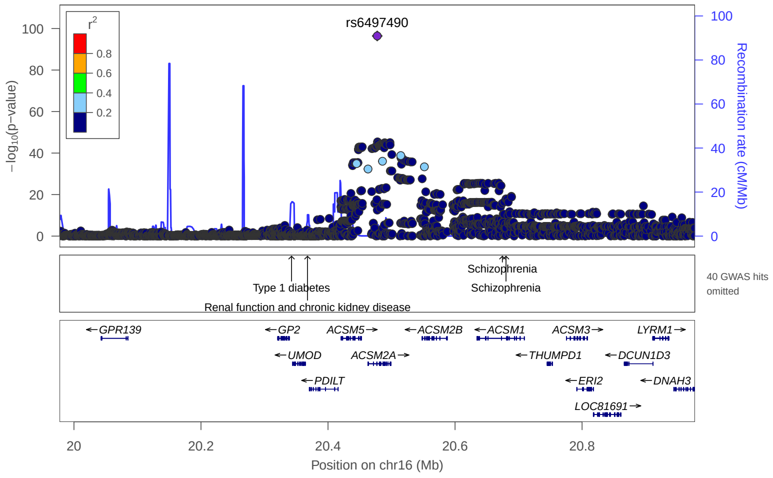

X − 17328

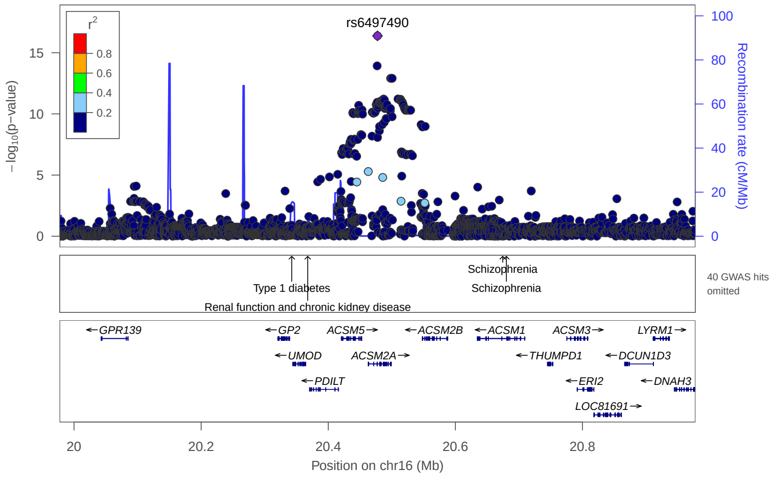

indoleacetylglycine

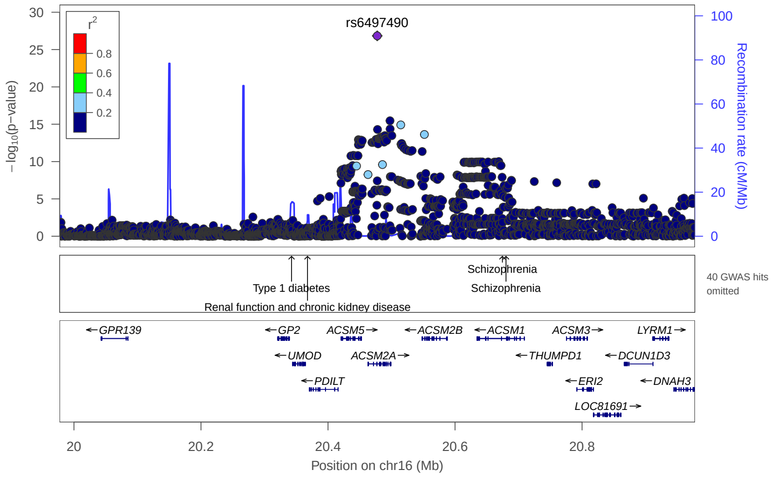

indoleacetoylcarnitine*

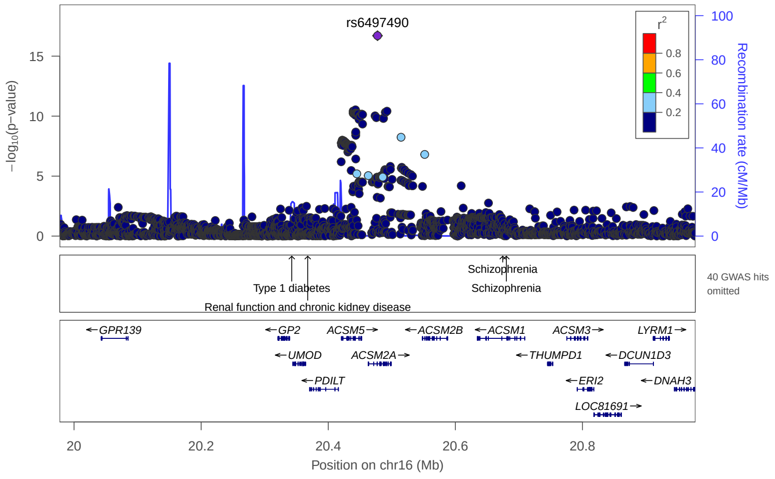

glucuronide of C10H18O2 (10)*

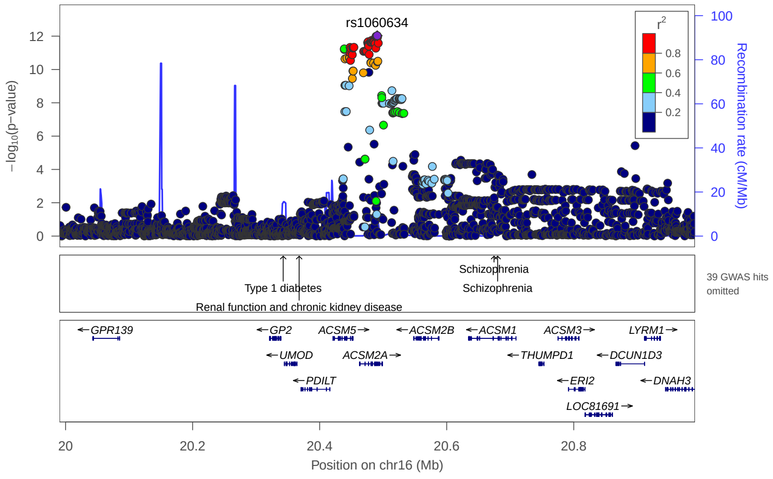

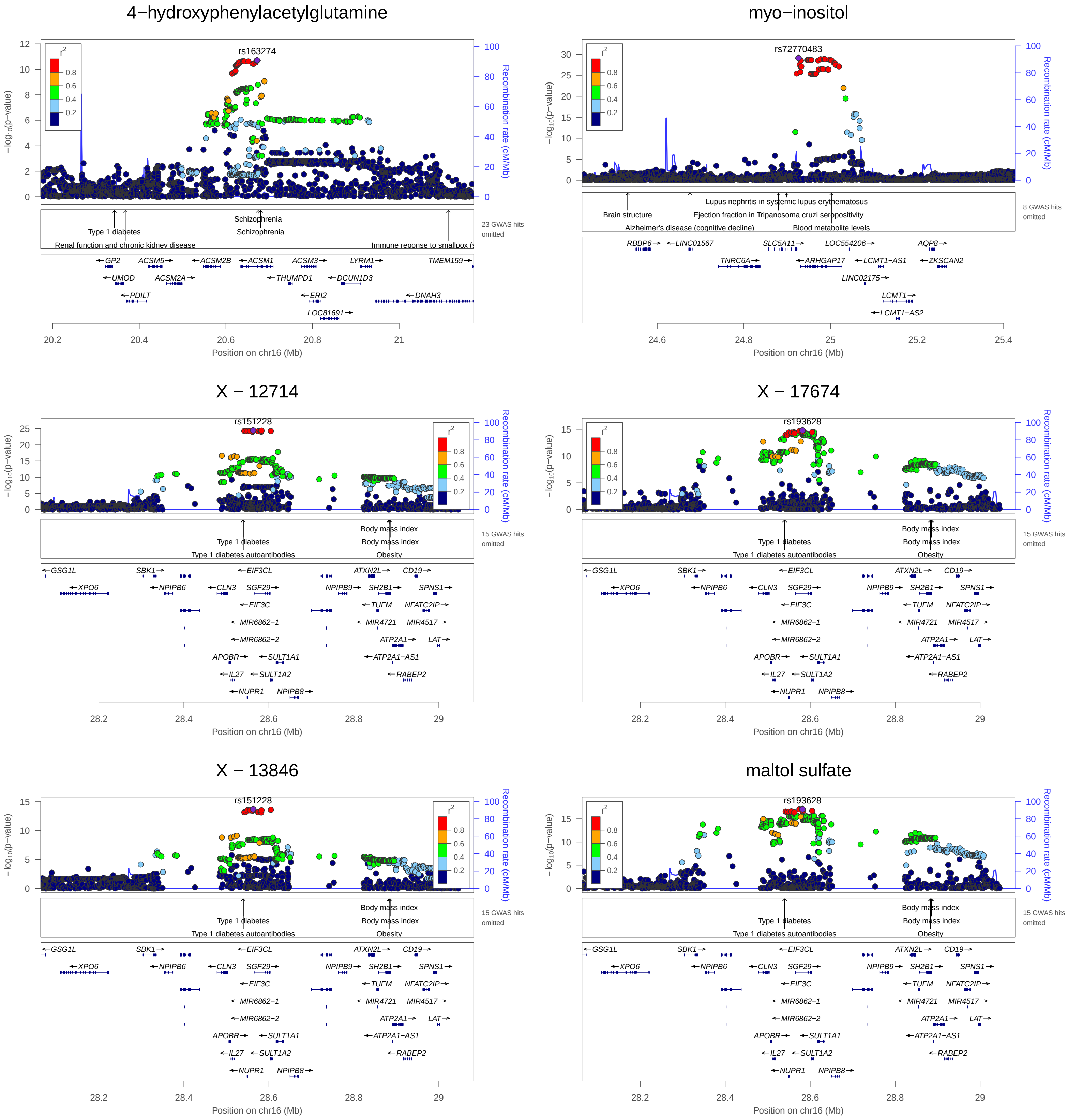

4−hydroxyphenylacetylglutamine
rs163274
−log10(p−value)
Recombination rate (cM/Mb)
r2
Type 1 diabetes
Renal function and chronic kidney disease
Schizophrenia
Schizophrenia
Immune reponse to smallpox (s
23 GWAS hits omitted
GP2
ACSM5
ACSM2B
ACSM1
ACSM3
LYRM1
TMEM159
UMOD
ACSM2A
THUMPD1
DCUN1D3
PDILT
ERI2
DNAH3
LOC81691
Position on chr16 (Mb)
myo−inositol
rs72770483
Brain structure
Lupus nephritis in systemic lupus erythematosus
Ejection fraction in Tripanosoma cruzi seropositivity
Alzheimer's disease (cognitive decline)
Blood metabolite levels
8 GWAS hits omitted
RBBP6
LINC01567
SLC5A11
LOC554206
AQP8
TNRC6A
ARHGAP17
LCMT1−AS1
ZKSCAN2
LINC02175
LCMT1
LCMT1−AS2
X − 12714
rs151228
X − 17674
rs193628
X − 13846
rs151228
maltol sulfate
rs193628
Body mass index
Type 1 diabetes
Body mass index
Type 1 diabetes autoantibodies
Obesity
15 GWAS hits omitted
GSG1L
SBK1
EIF3CL
ATXN2L
CD19
XPO6
NPIPB6
CLN3
SGF29
NPIPB9
SH2B1
SPNS1
EIF3C
TUFM
NFATC2IP
MIR6862−1
MIR4721
MIR4517
MIR6862−2
ATP2A1
LAT
APOBR
SULT1A1
ATP2A1−AS1
IL27
SULT1A2
RABEP2
NUPR1
NPIPB8

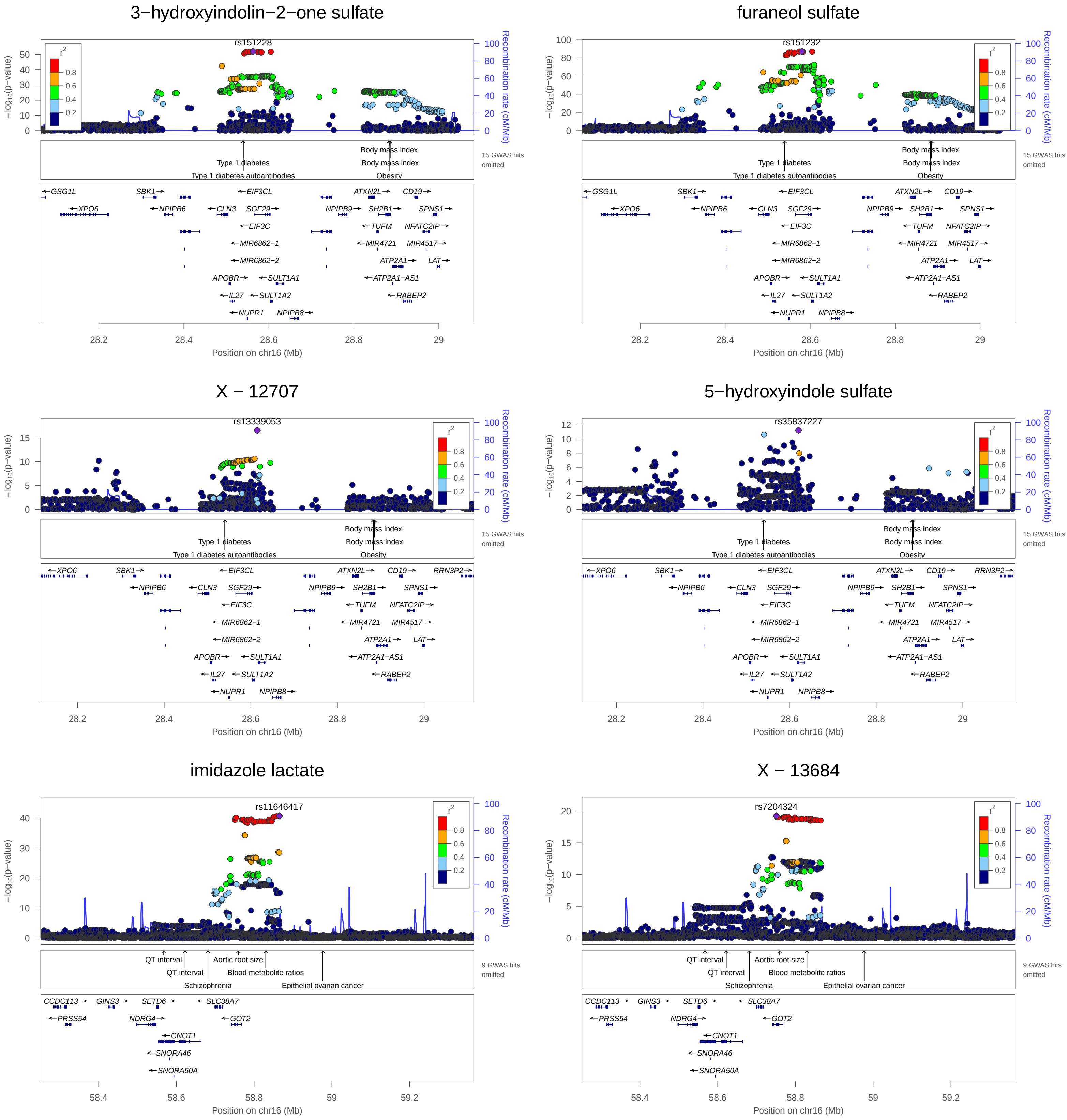

3−hydroxyindolin−2−one sulfate
rs151228
furaneol sulfate
rs151232
X − 12707
rs13339053
5−hydroxyindole sulfate
rs35837227
imidazole lactate
rs11646417
X − 13684
rs7204324
−log10(p−value)
Recombination rate (cM/Mb)
r2
0.8
0.6
0.4
0.2
Body mass index
Type 1 diabetes
Type 1 diabetes autoantibodies
Obesity
15 GWAS hits omitted
QT interval
Aortic root size
Blood metabolite ratios
Schizophrenia
Epithelial ovarian cancer
9 GWAS hits omitted
GSG1L
XPO6
SBK1
NPIPB6
CLN3
SGF29
EIF3CL
EIF3C
MIR6862−1
MIR6862−2
APOBR
SULT1A1
IL27
SULT1A2
NUPR1
NPIPB8
NPIPB9
ATXN2L
SH2B1
TUFM
MIR4721
ATP2A1
ATP2A1−AS1
RABEP2
CD19
SPNS1
NFATC2IP
MIR4517
LAT
RRN3P2
CCDC113
PRSS54
GINS3
SETD6
NDRG4
CNOT1
SNORA46
SNORA50A
SLC38A7
GOT2
Position on chr16 (Mb)

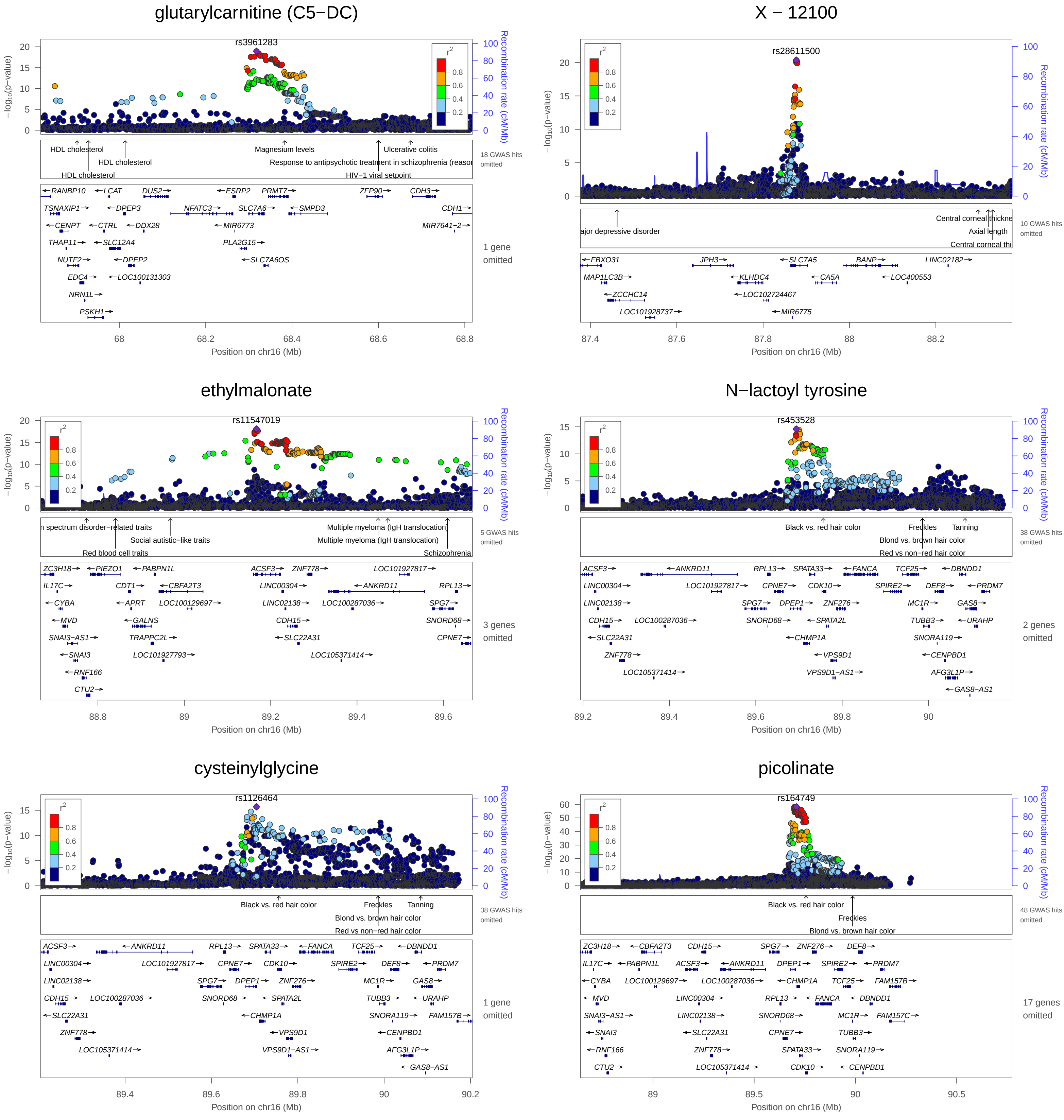

glutarylcarnitine (C5−DC)
rs3961283
−log10(p−value)
Recombination rate (cM/Mb)
HDL cholesterol
HDL cholesterol
HDL cholesterol
Magnesium levels
Ulcerative colitis
Response to antipsychotic treatment in schizophrenia (reasor
HIV−1 viral setpoint
18 GWAS hits omitted
RANBP10 LCAT DUS2 ESRP2 PRMT7 ZFP90 CDH3
TSNAXIP1 DPEP3 NFATC3 SLC7A6 SMPD3 CDH1
CENPT CTRL DDX28 MIR6773 MIR7641−2
THAP11 SLC12A4 PLA2G15
NUTF2 DPEP2 SLC7A6OS
EDC4 LOC100131303
NRN1L
PSKH1
1 gene omitted
Position on chr16 (Mb)
X − 12100
rs28611500
Central corneal thickne
Axial length
Central corneal thi
ajor depressive disorder
10 GWAS hits omitted
FBXO31 JPH3 SLC7A5 BANP LINC02182
MAP1LC3B KLHDC4 CA5A LOC400553
ZCCHC14 LOC102724467
LOC101928737 MIR6775
Position on chr16 (Mb)
ethylmalonate
rs11547019
n spectrum disorder−related traits
Social autistic−like traits
Red blood cell traits
Multiple myeloma (IgH translocation)
Multiple myeloma (IgH translocation)
Schizophrenia
5 GWAS hits omitted
3 genes omitted
Position on chr16 (Mb)
N−lactoyl tyrosine
rs453528
Black vs. red hair color
Freckles
Tanning
Blond vs. brown hair color
Red vs non−red hair color
38 GWAS hits omitted
2 genes omitted
Position on chr16 (Mb)
cysteinylglycine
rs1126464
Black vs. red hair color
Freckles
Tanning
Blond vs. brown hair color
Red vs non−red hair color
38 GWAS hits omitted
1 gene omitted
Position on chr16 (Mb)
picolinate
rs164749
Black vs. red hair color
Freckles
Blond vs. brown hair color
48 GWAS hits omitted
17 genes omitted
Position on chr16 (Mb)

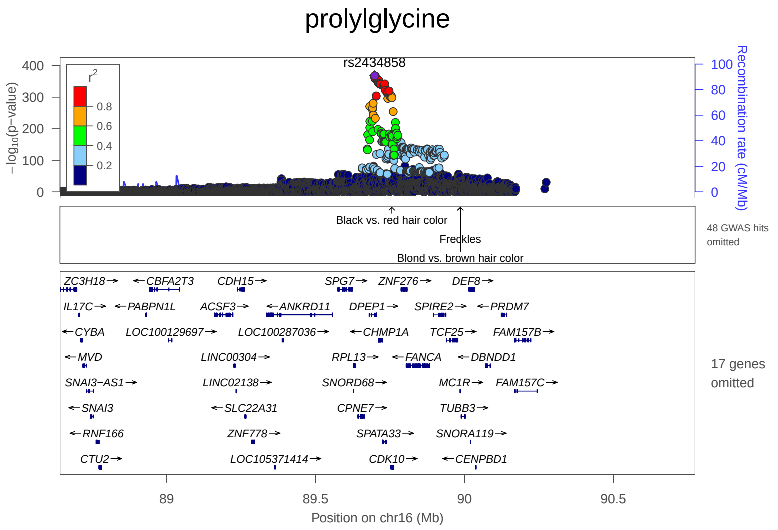
prolylglycine
rs2434858
−log10(p−value)
Recombination rate (cM/Mb)
r2
Black vs. red hair color
Freckles
Blond vs. brown hair color
48 GWAS hits omitted
ZC3H18 CBFA2T3 CDH15 SPG7 ZNF276 DEF8
IL17C PABPN1L ACSF3 ANKRD11 DPEP1 SPIRE2 PRDM7
CYBA LOC100129697 LOC100287036 CHMP1A TCF25 FAM157B
MVD LINC00304 RPL13 FANCA DBNDD1
SNAI3−AS1 LINC02138 SNORD68 MC1R FAM157C
SNAI3 SLC22A31 CPNE7 TUBB3
RNF166 ZNF778 SPATA33 SNORA119
CTU2 LOC105371414 CDK10 CENPBD1
17 genes omitted
Position on chr16 (Mb)

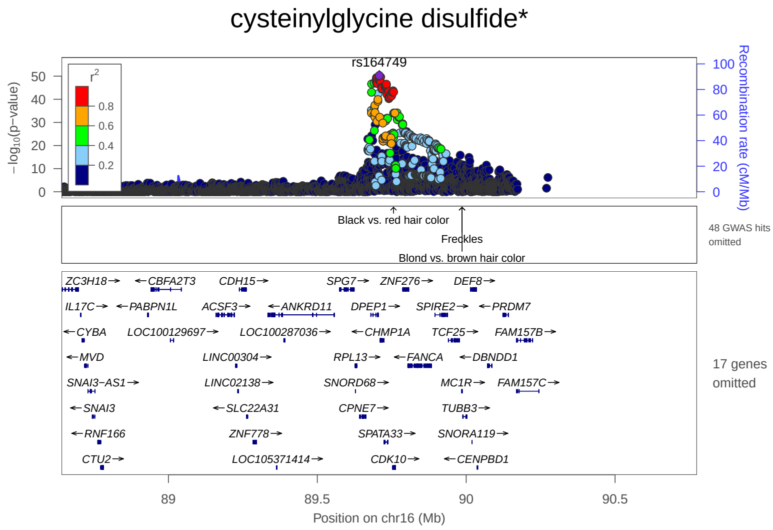
cysteinylglycine disulfide*
rs164749
−log10(p−value)
Recombination rate (cM/Mb)
r2
Black vs. red hair color
Freckles
Blond vs. brown hair color
48 GWAS hits omitted
ZC3H18 CBFA2T3 CDH15 SPG7 ZNF276 DEF8
IL17C PABPN1L ACSF3 ANKRD11 DPEP1 SPIRE2 PRDM7
CYBA LOC100129697 LOC100287036 CHMP1A TCF25 FAM157B
MVD LINC00304 RPL13 FANCA DBNDD1
SNAI3−AS1 LINC02138 SNORD68 MC1R FAM157C
SNAI3 SLC22A31 CPNE7 TUBB3
RNF166 ZNF778 SPATA33 SNORA119
CTU2 LOC105371414 CDK10 CENPBD1
17 genes omitted
Position on chr16 (Mb)

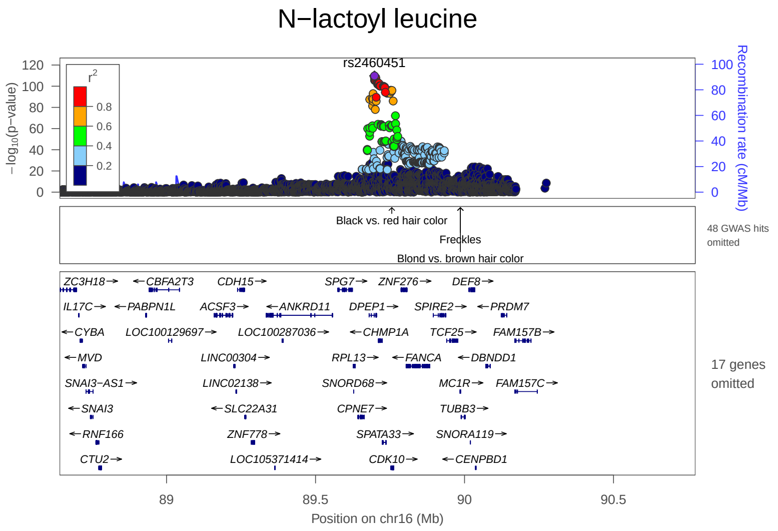
N−lactoyl leucine
rs2460451
−log10(p−value)
Recombination rate (cM/Mb)
r2
Black vs. red hair color
Freckles
Blond vs. brown hair color
48 GWAS hits omitted
ZC3H18 CBFA2T3 CDH15 SPG7 ZNF276 DEF8
IL17C PABPN1L ACSF3 ANKRD11 DPEP1 SPIRE2 PRDM7
CYBA LOC100129697 LOC100287036 CHMP1A TCF25 FAM157B
MVD LINC00304 RPL13 FANCA DBNDD1
SNAI3−AS1 LINC02138 SNORD68 MC1R FAM157C
SNAI3 SLC22A31 CPNE7 TUBB3
RNF166 ZNF778 SPATA33 SNORA119
CTU2 LOC105371414 CDK10 CENPBD1
17 genes omitted
Position on chr16 (Mb)

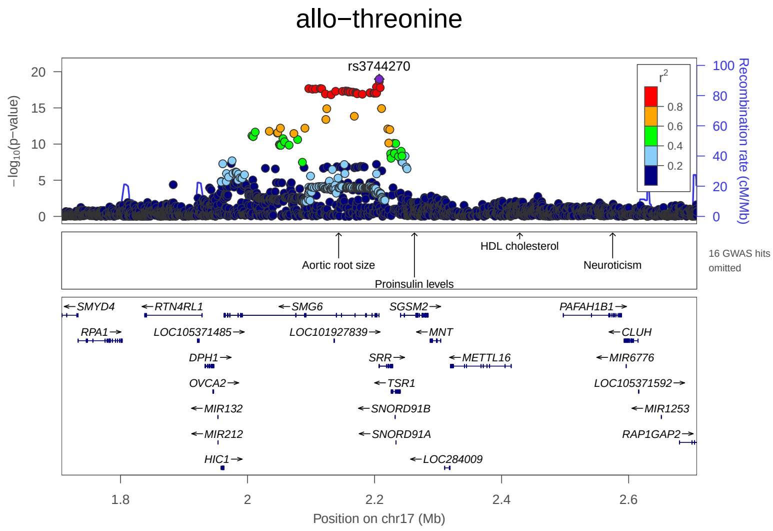
allo−threonine
rs3744270
−log10(p−value)
Recombination rate (cM/Mb)
r2
HDL cholesterol
Aortic root size
Neuroticism
Proinsulin levels
16 GWAS hits omitted
SMYD4 RTN4RL1 SMG6 SGSM2 PAFAH1B1
RPA1 LOC105371485 LOC101927839 MNT CLUH
DPH1 SRR METTL16 MIR6776
OVCA2 TSR1 LOC105371592
MIR132 SNORD91B MIR1253
MIR212 SNORD91A RAP1GAP2
HIC1 LOC284009
Position on chr17 (Mb)

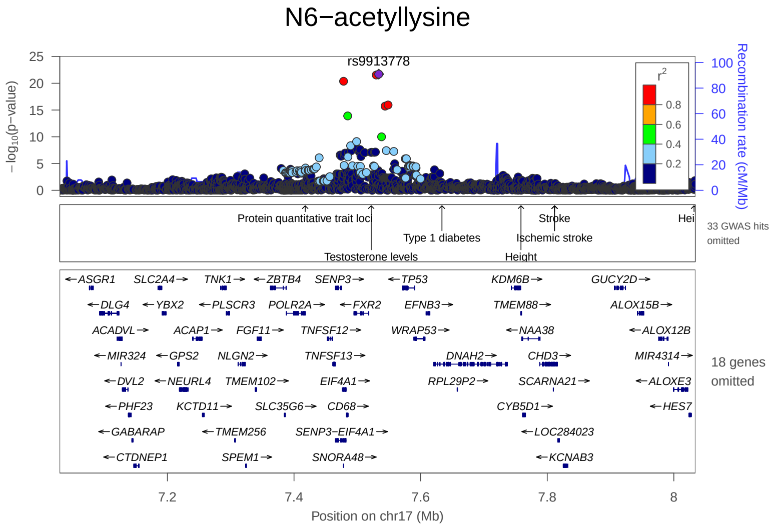
N6−acetyllysine
rs9913778
−log10(p−value)
Recombination rate (cM/Mb)
r2
Protein quantitative trait loci
Stroke
Hei
Type 1 diabetes
Ischemic stroke
Testosterone levels
Height
33 GWAS hits omitted
ASGR1 SLC2A4 TNK1 ZBTB4 SENP3 TP53 KDM6B GUCY2D
DLG4 YBX2 PLSCR3 POLR2A FXR2 EFNB3 TMEM88 ALOX15B
ACADVL ACAP1 FGF11 TNFSF12 WRAP53 NAA38 ALOX12B
MIR324 GPS2 NLGN2 TNFSF13 DNAH2 CHD3 MIR4314
DVL2 NEURL4 TMEM102 EIF4A1 RPL29P2 SCARNA21 ALOXE3
PHF23 KCTD11 SLC35G6 CD68 CYB5D1 HES7
GABARAP TMEM256 SENP3−EIF4A1 LOC284023
CTDNEP1 SPEM1 SNORA48 KCNAB3
18 genes omitted
Position on chr17 (Mb)

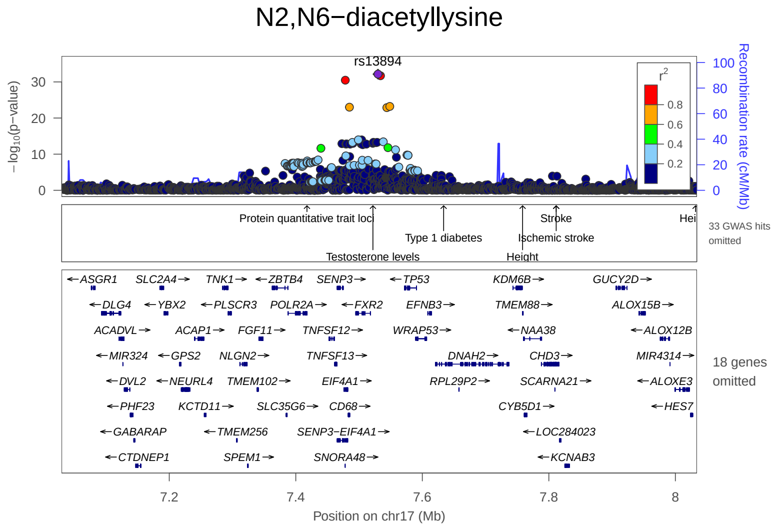
N2,N6−diacetyllysine
rs13894
−log10(p−value)
Recombination rate (cM/Mb)
r2
Protein quantitative trait loci
Stroke
Hei
Type 1 diabetes
Ischemic stroke
Testosterone levels
Height
33 GWAS hits omitted
ASGR1 SLC2A4 TNK1 ZBTB4 SENP3 TP53 KDM6B GUCY2D
DLG4 YBX2 PLSCR3 POLR2A FXR2 EFNB3 TMEM88 ALOX15B
ACADVL ACAP1 FGF11 TNFSF12 WRAP53 NAA38 ALOX12B
MIR324 GPS2 NLGN2 TNFSF13 DNAH2 CHD3 MIR4314
DVL2 NEURL4 TMEM102 EIF4A1 RPL29P2 SCARNA21 ALOXE3
PHF23 KCTD11 SLC35G6 CD68 CYB5D1 HES7
GABARAP TMEM256 SENP3−EIF4A1 LOC284023
CTDNEP1 SPEM1 SNORA48 KCNAB3
18 genes omitted
Position on chr17 (Mb)

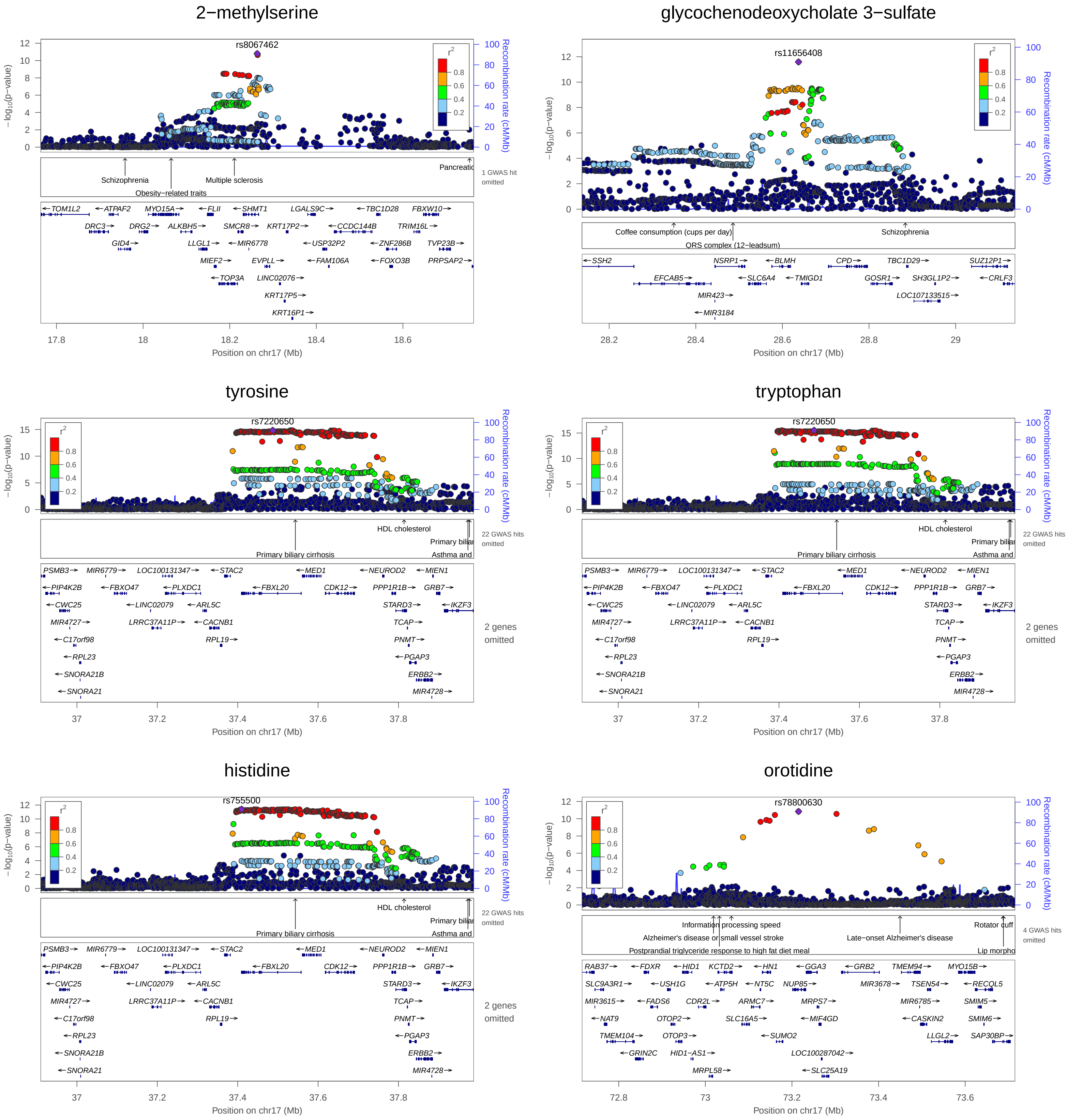
2−methylserine
rs8067462
Schizophrenia
Obesity−related traits
Multiple sclerosis
Pancreatic
1 GWAS hit omitted
TOM1L2
ATPAF2
MYO15A
FLII
SHMT1
LGALS9C
TBC1D28
FBXW10
DRC3
DRG2
ALKBH5
SMCR8
KRT17P2
CCDC144B
TRIM16L
GID4
LLGL1
MIR6778
USP32P2
ZNF286B
TVP23B
MIEF2
EVPLL
FAM106A
FOXO3B
PRPSAP2
TOP3A
LINC02076
KRT17P5
KRT16P1
Position on chr17 (Mb)
glycochenodeoxycholate 3−sulfate
rs11656408
Coffee consumption (cups per day)
QRS complex (12−leadsum)
Schizophrenia
SSH2
NSRP1
BLMH
CPD
TBC1D29
SUZ12P1
EFCAB5
SLC6A4
TMIGD1
GOSR1
SH3GL1P2
CRLF3
MIR423
LOC107133515
MIR3184
Position on chr17 (Mb)
tyrosine
rs7220650
HDL cholesterol
Primary biliar
Primary biliary cirrhosis
Asthma and
22 GWAS hits omitted
2 genes omitted
Position on chr17 (Mb)
tryptophan
rs7220650
HDL cholesterol
Primary biliar
Primary biliary cirrhosis
Asthma and
22 GWAS hits omitted
2 genes omitted
Position on chr17 (Mb)
histidine
rs755500
HDL cholesterol
Primary biliar
Primary biliary cirrhosis
Asthma and
22 GWAS hits omitted
2 genes omitted
PSMB3
MIR6779
LOC100131347
STAC2
MED1
NEUROD2
MIEN1
PIP4K2B
FBXO47
PLXDC1
FBXL20
CDK12
PPP1R1B
GRB7
CWC25
LINC02079
ARL5C
STARD3
IKZF3
MIR4727
LRRC37A11P
CACNB1
TCAP
C17orf98
RPL19
PNMT
RPL23
PGAP3
SNORA21B
ERBB2
SNORA21
MIR4728
Position on chr17 (Mb)
orotidine
rs78800630
Information processing speed
Alzheimer's disease or small vessel stroke
Postprandial triglyceride response to high fat diet meal
Late−onset Alzheimer's disease
Rotator cuff
Lip morpho
4 GWAS hits omitted
RAB37
FDXR
HID1
KCTD2
HN1
GGA3
GRB2
TMEM94
MIR15B
SLC9A3R1
USH1G
ATP5H
NT5C
NUP85
MIR3678
TSEN54
RECQL5
MIR3615
FADS6
CDR2L
ARMC7
MRPS7
MIR6785
SMIM5
NAT9
OTOP2
SLC16A5
MIF4GD
CASKIN2
SMIM6
TMEM104
OTOP3
SUMO2
LLGL2
SAP30BP
GRIN2C
HID1−AS1
LOC100287042
MRPL58
SLC25A19
Position on chr17 (Mb)

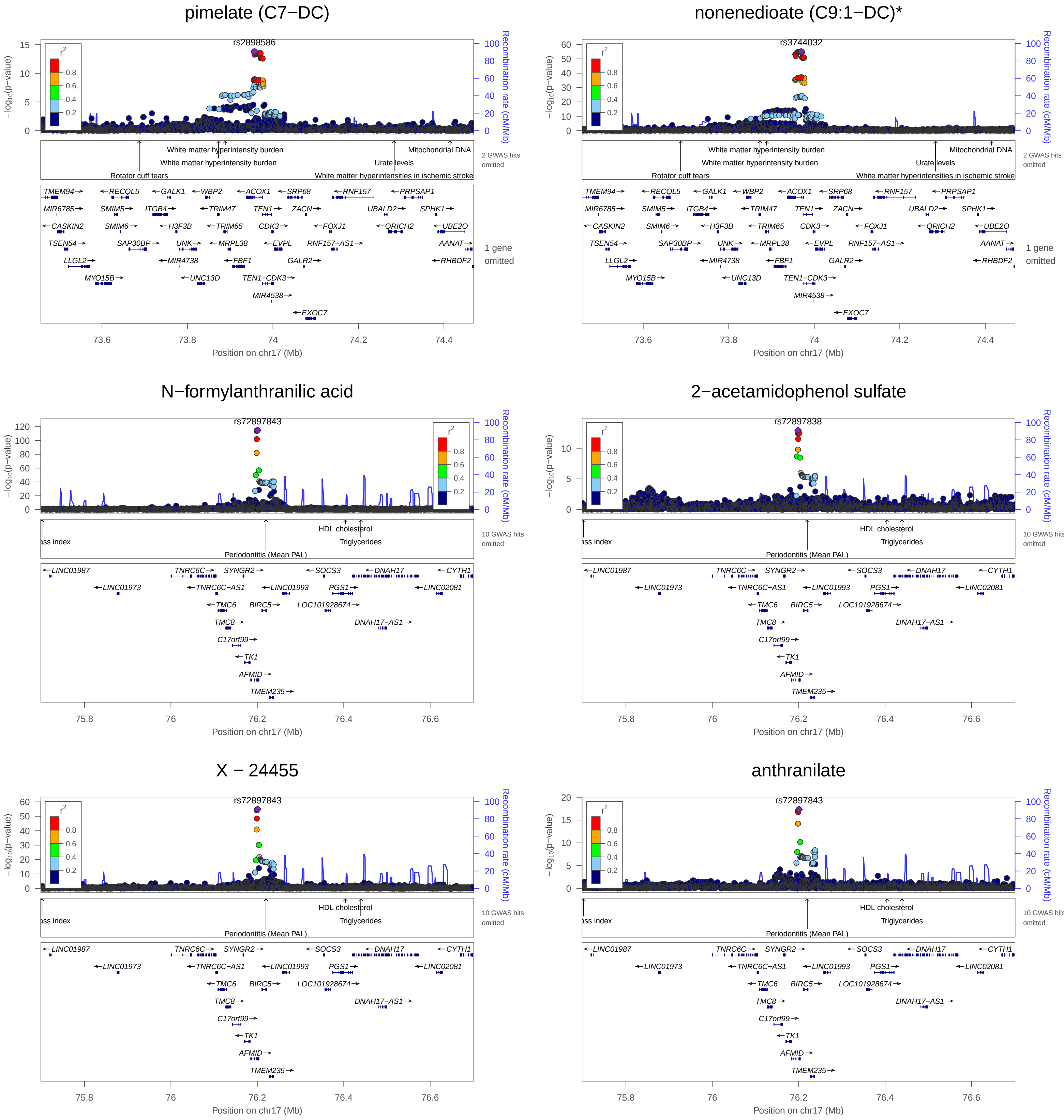
pimelate (C7−DC)
rs2898586
nonenedioate (C9:1−DC)*
rs3744032
N−formylanthranilic acid
rs72897843
2−acetamidophenol sulfate
rs72897838
X − 24455
rs72897843
anthranilate
rs72897843
−log10(p−value)
Recombination rate (cM/Mb)
r2
0.8
0.6
0.4
0.2
White matter hyperintensity burden
Mitochondrial DNA
Rotator cuff tears
Urate levels
White matter hyperintensities in ischemic stroke
2 GWAS hits omitted
TMEM94 RECQL5 GALK1 WBP2 ACOX1 SRP68 RNF157 PRPSAP1
MIR6785 SMIM5 ITGB4 TRIM47 TEN1 ZACN UBALD2 SPHK1
CASKIN2 SMIM6 H3F3B TRIM65 CDK3 FOXJ1 QRICH2 UBE2O
TSEN54 SAP30BP UNK MRPL38 EVPL RNF157−AS1 AANAT
LLGL2 MIR4738 FBF1 GALR2 RHBDF2
MYO15B UNC13D TEN1−CDK3
MIR4538
EXOC7
1 gene omitted
Position on chr17 (Mb)
HDL cholesterol
Triglycerides
Periodontitis (Mean PAL)
10 GWAS hits omitted
LINC01987 TNRC6C SYNGR2 SOCS3 DNAH17 CYTH1
LINC01973 TNRC6C−AS1 LINC01993 PGS1 LINC02081
TMC6 BIRC5 LOC101928674
TMC8 DNAH17−AS1
C17orf99
TK1
AFMID
TMEM235

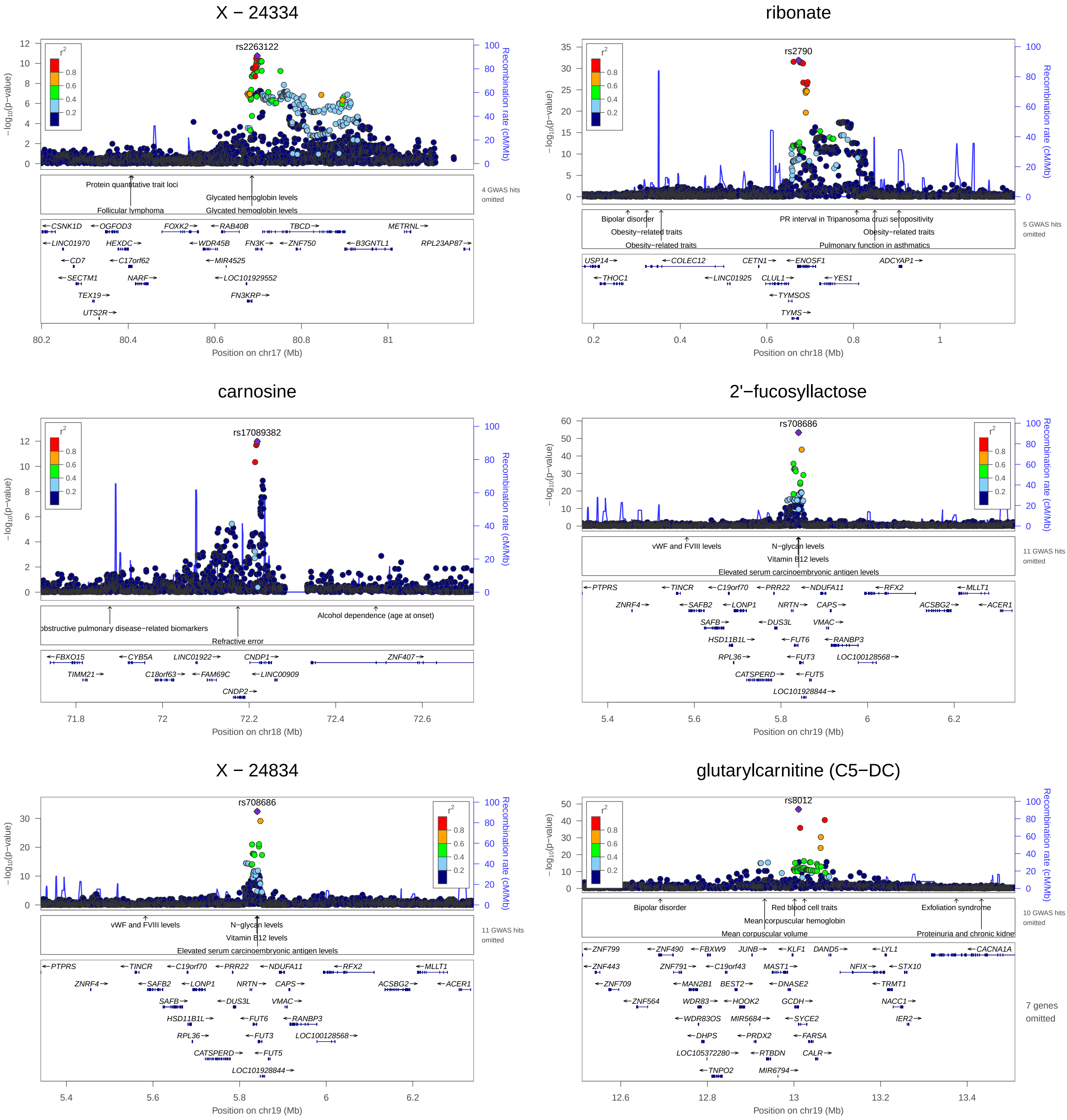
X − 24334
rs2263122
−log10(p−value)
Recombination rate (cM/Mb)
r2
Protein quantitative trait loci
Glycated hemoglobin levels
Follicular lymphoma
Glycated hemoglobin levels
4 GWAS hits omitted
CSNK1D
OGFOD3
FOXK2
RAB40B
TBCD
METRNL
LINC01970
HEXDC
WDR45B
FN3K
ZNF750
B3GNTL1
RPL23AP87
CD7
C17orf62
MIR4525
SECTM1
NARF
LOC101929552
TEX19
FN3KRP
UTS2R
Position on chr17 (Mb)
ribonate
rs2790
Bipolar disorder
Obesity−related traits
Obesity−related traits
PR interval in Tripanosoma cruzi seropositivity
Obesity−related traits
Pulmonary function in asthmatics
5 GWAS hits omitted
USP14
COLEC12
CETN1
ENOSF1
ADCYAP1
THOC1
LINC01925
CLUL1
YES1
TYMSOS
TYMS
Position on chr18 (Mb)
carnosine
rs17089382
obstructive pulmonary disease−related biomarkers
Refractive error
Alcohol dependence (age at onset)
FBXO15
CYB5A
LINC01922
CNDP1
ZNF407
TIMM21
C18orf63
FAM69C
LINC00909
CNDP2
Position on chr18 (Mb)
2'−fucosyllactose
rs708686
vWF and FVIII levels
N−glycan levels
Vitamin B12 levels
Elevated serum carcinoembryonic antigen levels
11 GWAS hits omitted
PTPRS
TINCR
C19orf70
PRR22
NDUFA11
RFX2
MLLT1
ZNF4
SAFB2
LONP1
NRTN
CAPS
ACSBG2
ACER1
SAFB
DUS3L
VMAC
HSD11B1L
FUT6
RANBP3
RPL36
FUT3
LOC100128568
CATSPERD
FUT5
LOC101928844
Position on chr19 (Mb)
X − 24834
rs708686
vWF and FVIII levels
N−glycan levels
Vitamin B12 levels
Elevated serum carcinoembryonic antigen levels
11 GWAS hits omitted
Position on chr19 (Mb)
glutarylcarnitine (C5−DC)
rs8012
Bipolar disorder
Red blood cell traits
Mean corpuscular hemoglobin
Mean corpuscular volume
Exfoliation syndrome
Proteinuria and chronic kidne
10 GWAS hits omitted
ZNF799
ZNF490
FBXW9
JUNB
KLF1
DAND5
LYL1
CACNA1A
ZNF443
ZNF791
C19orf43
MAST1
NFIX
STX10
ZNF709
MAN2B1
BEST2
DNASE2
TRMT1
ZNF564
WDR83
HOOK2
GCDH
NACC1
WDR83OS
MIR5684
SYCE2
IER2
DHPS
PRDX2
FARSA
LOC105372280
RTBDN
CALR
TNPO2
MIR6794
7 genes omitted
Position on chr19 (Mb)

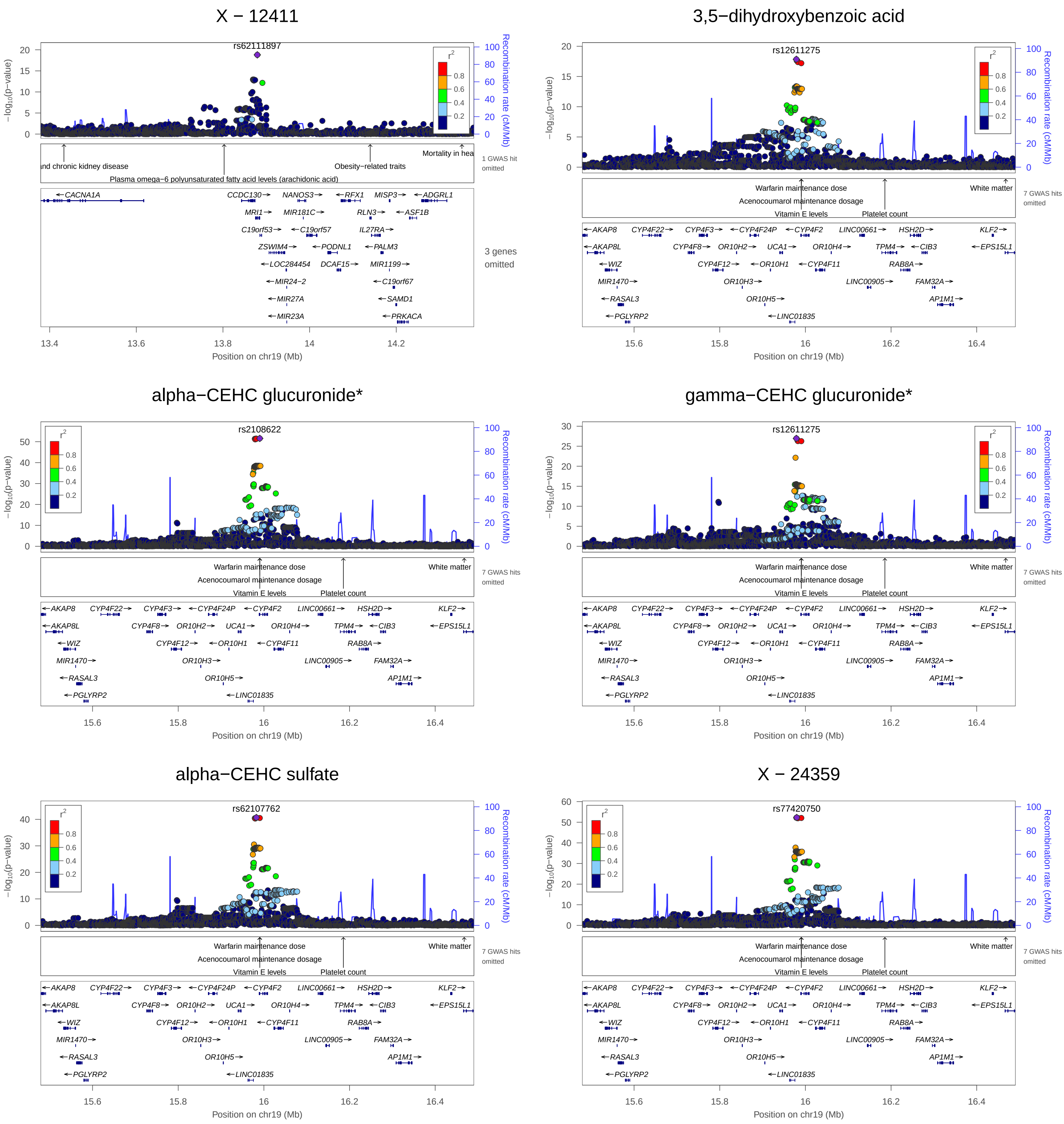

X − 12411
rs62111897
−log10(p−value)
Recombination rate (cM/Mb)
r2
0.8
0.6
0.4
0.2
Mortality in hea
nd chronic kidney disease
Obesity−related traits
Plasma omega−6 polyunsaturated fatty acid levels (arachidonic acid)
1 GWAS hit omitted
CACNA1A
CCDC130
NANOS3
RFX1
MISP3
ADGRL1
MRI1
MIR181C
RLN3
ASF1B
C19orf53
C19orf57
IL27RA
ZSWIM4
PODNL1
PALM3
LOC284454
DCAF15
MIR1199
MIR24−2
C19orf67
MIR27A
SAMD1
MIR23A
PRKACA
3 genes omitted
Position on chr19 (Mb)
3,5−dihydroxybenzoic acid
rs12611275
alpha−CEHC glucuronide*
rs2108622
gamma−CEHC glucuronide*
rs12611275
alpha−CEHC sulfate
rs62107762
X − 24359
rs77420750
Warfarin maintenance dose
Acenocoumarol maintenance dosage
Vitamin E levels
Platelet count
White matter
7 GWAS hits omitted
AKAP8
CYP4F22
CYP4F3
CYP4F24P
CYP4F2
LINC00661
HSH2D
KLF2
AKAP8L
CYP4F8
OR10H2
UCA1
OR10H4
TPM4
CIB3
EPS15L1
WIZ
CYP4F12
OR10H1
CYP4F11
RAB8A
MIR1470
OR10H3
LINC00905
FAM32A
RASAL3
OR10H5
AP1M1
PGLYRP2
LINC01835
Position on chr19 (Mb)

alpha−CEHC glycine*

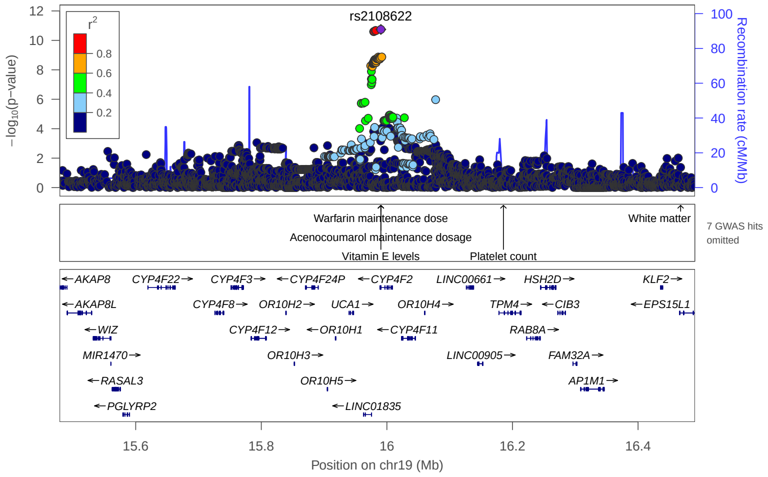

alpha−CEHC taurine*

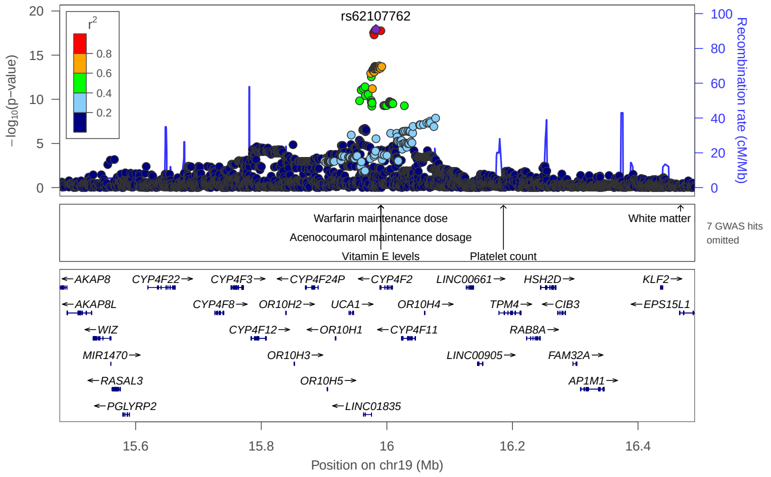

X − 24807

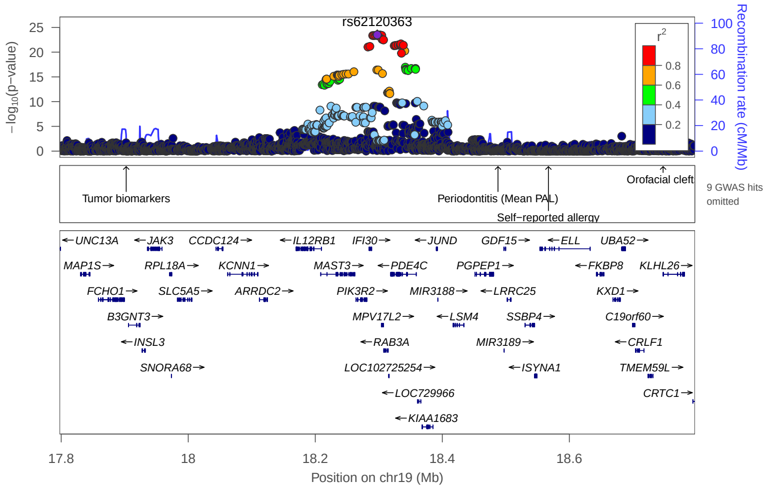

carnosine

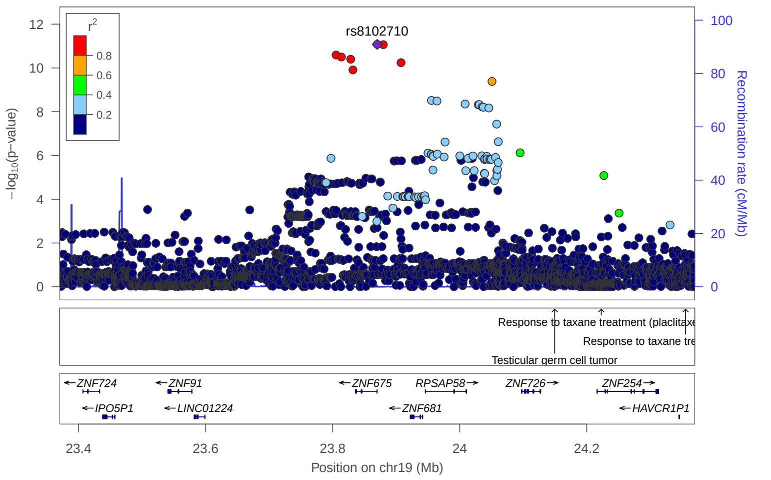

vanillic acid glycine

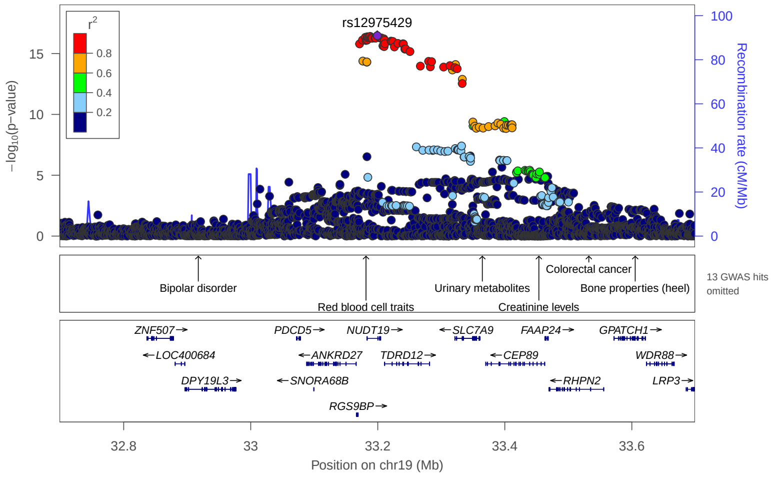

lysine

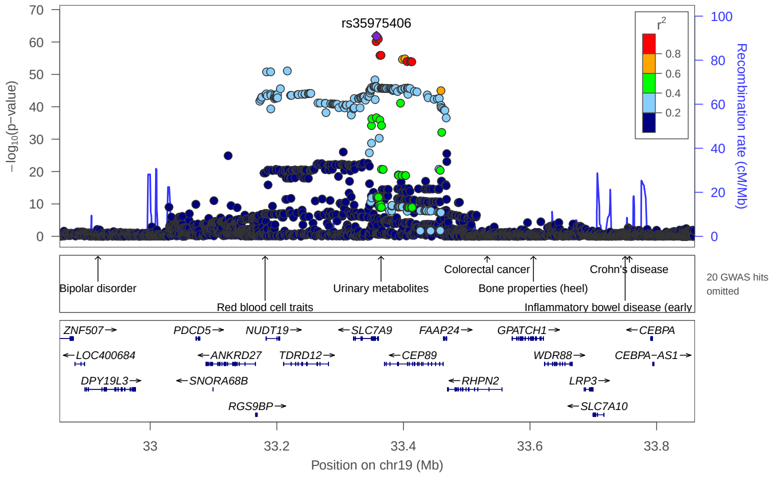

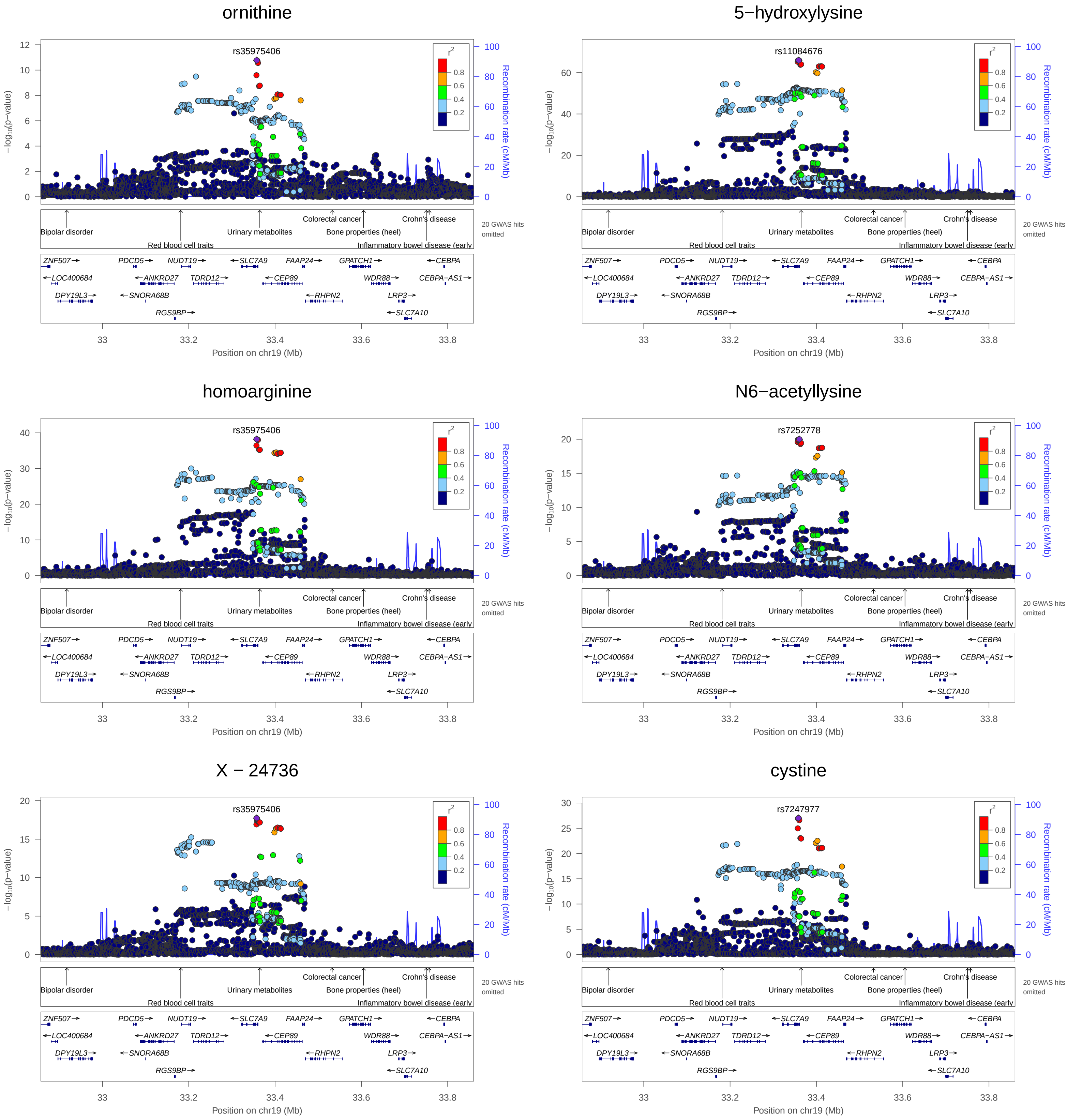

ornithine
rs35975406
5−hydroxylysine
rs11084676
homoarginine
rs35975406
N6−acetyllysine
rs7252778
X − 24736
rs35975406
cystine
rs7247977
−log10(p−value)
Recombination rate (cM/Mb)
r2
0.8
0.6
0.4
0.2
Bipolar disorder
Red blood cell traits
Urinary metabolites
Colorectal cancer
Bone properties (heel)
Crohn's disease
Inflammatory bowel disease (early
20 GWAS hits omitted
ZNF507
LOC400684
DPY19L3
PDCD5
ANKRD27
SNORA68B
RGS9BP
NUDT19
TDRD12
SLC7A9
CEP89
FAAP24
RHPN2
GPATCH1
WDR88
LRP3
SLC7A10
CEBPA
CEBPA−AS1
33
33.2
33.4
33.6
33.8
Position on chr19 (Mb)

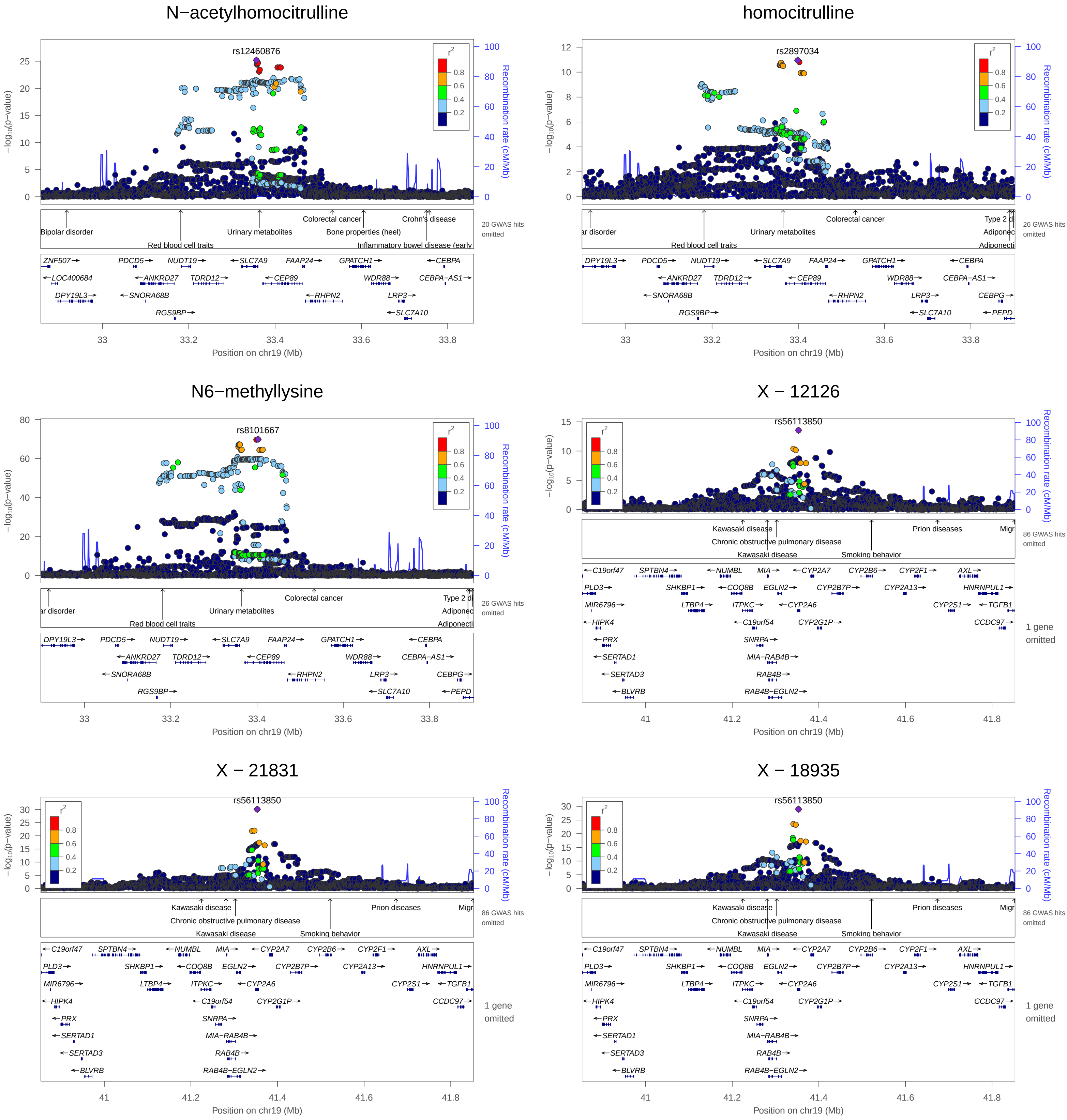

N−acetylhomocitrulline
rs12460876
homocitrulline
rs2897034
N6−methyllysine
rs8101667
X − 12126
X − 21831
X − 18935
rs56113850
−log10(p−value)
Recombination rate (cM/Mb)
r2
0.8
0.6
0.4
0.2
Position on chr19 (Mb)
Bipolar disorder
Red blood cell traits
Urinary metabolites
Colorectal cancer
Bone properties (heel)
Crohn's disease
Inflammatory bowel disease (early
20 GWAS hits omitted
26 GWAS hits omitted
86 GWAS hits omitted
1 gene omitted
Type 2 di
Adiponec
Adiponecti
Kawasaki disease
Chronic obstructive pulmonary disease
Smoking behavior
Prion diseases
Migr
ZNF507
LOC400684
DPY19L3
PDCD5
ANKRD27
SNORA68B
RGS9BP
NUDT19
TDRD12
SLC7A9
CEP89
FAAP24
RHPN2
GPATCH1
WDR88
LRP3
SLC7A10
CEBPA
CEBPA−AS1
CEBPG
PEPD
C19orf47
PLD3
MIR6796
HIPK4
PRX
SERTAD1
SERTAD3
BLVRB
SPTBN4
SHKBP1
LTBP4
NUMBL
COQ8B
ITPKC
C19orf54
SNRPA
MIA−RAB4B
RAB4B
RAB4B−EGLN2
MIA
EGLN2
CYP2A6
CYP2A7
CYP2G1P
CYP2B6
CYP2B7P
CYP2A13
CYP2F1
CYP2S1
AXL
HNRNPUL1
TGFB1
CCDC97

epiandrosterone glucuronide

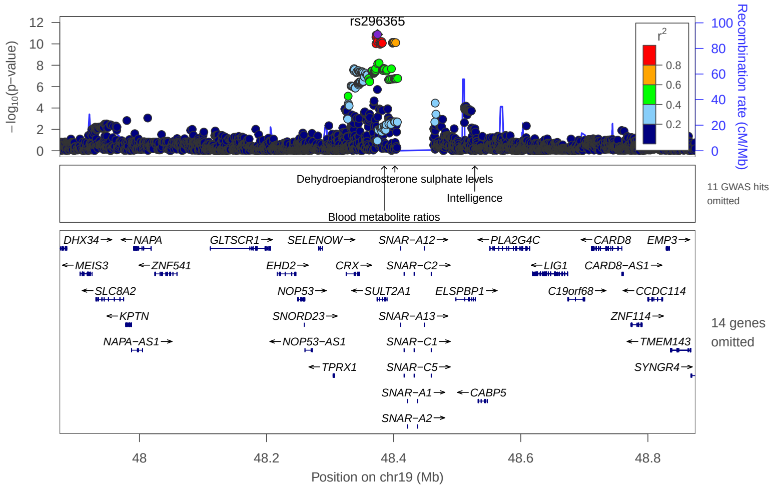

pregnenediol disulfate (C21H34O8S2)*

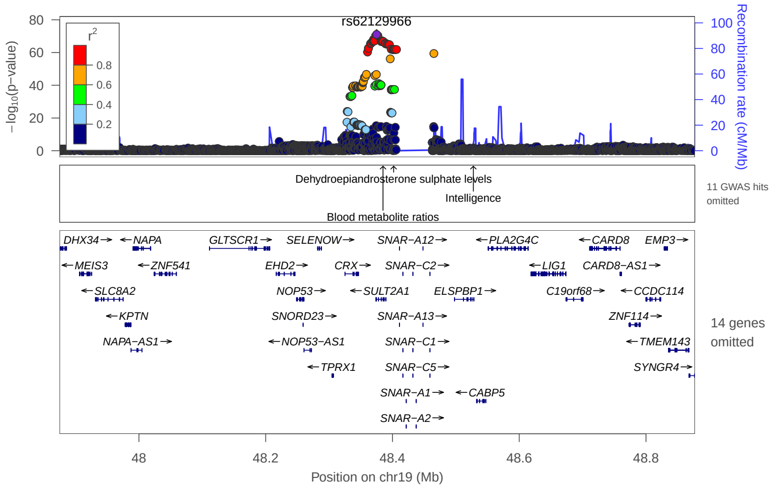

androstenediol (3beta,17beta) disulfate (1)

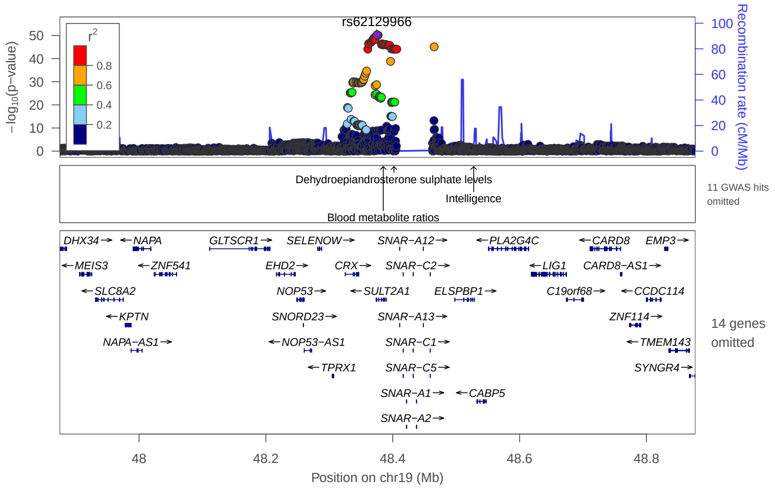

11−ketoetiocholanolone sulfate

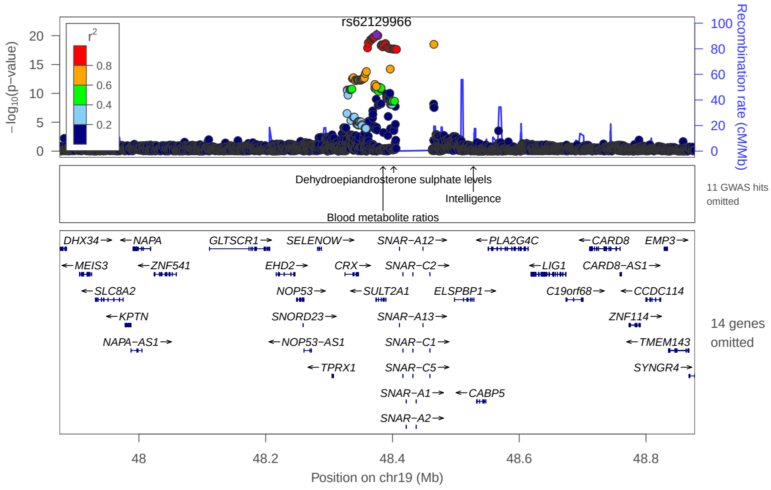

X − 21470

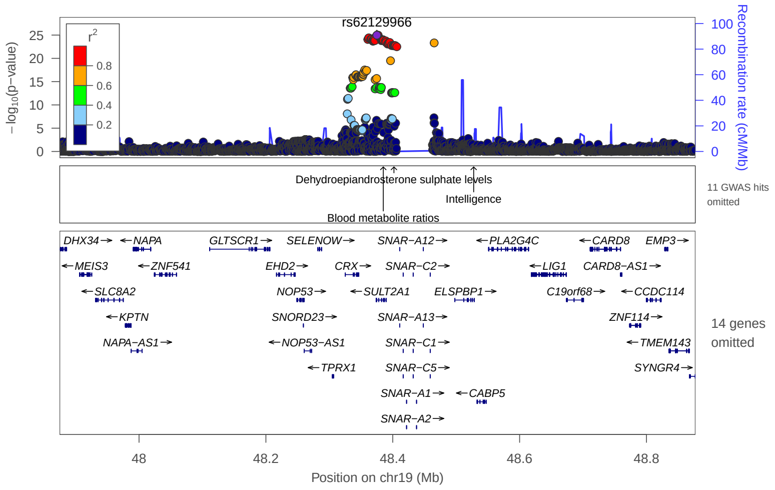

X − 21851

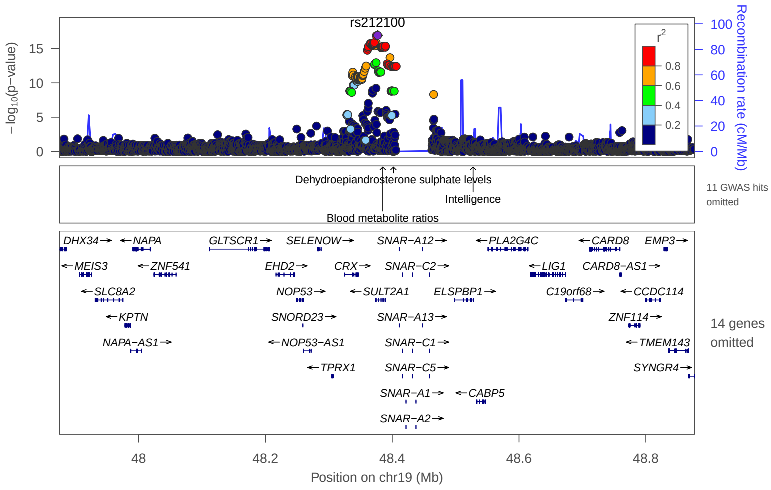

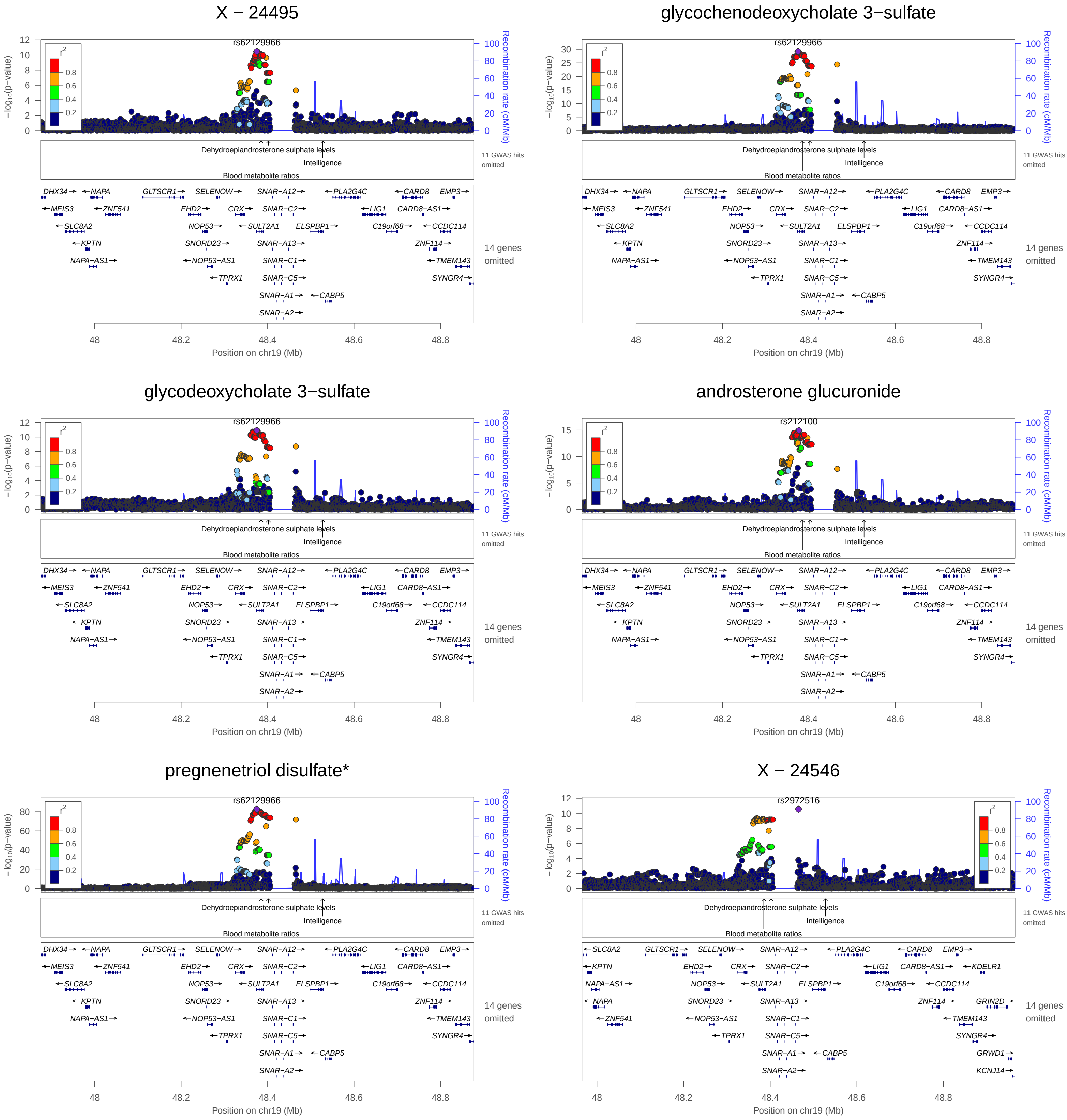

X − 24495
rs62129966
glycochenodeoxycholate 3−sulfate
rs62129966
glycodeoxycholate 3−sulfate
rs62129966
androsterone glucuronide
rs212100
pregnenetriol disulfate*
rs62129966
X − 24546
rs2972516
−log10(p−value)
Recombination rate (cM/Mb)
r2
Dehydroepiandrosterone sulphate levels
Intelligence
Blood metabolite ratios
11 GWAS hits omitted
14 genes omitted
DHX34 NAPA MEIS3 ZNF541 SLC8A2 KPTN NAPA−AS1 GLTSCR1 SELENOW EHD2 CRX NOP53 SULT2A1 SNORD23 NOP53−AS1 TPRX1 SNAR−A12 SNAR−C2 SNAR−A13 SNAR−C1 SNAR−C5 SNAR−A1 SNAR−A2 ELSPBP1 CABP5 PLA2G4C LIG1 C19orf68 CARD8 CARD8−AS1 CCDC114 ZNF114 TMEM143 SYNGR4 EMP3
KDELR1 GRIN2D GRWD1 KCNJ14
Position on chr19 (Mb)

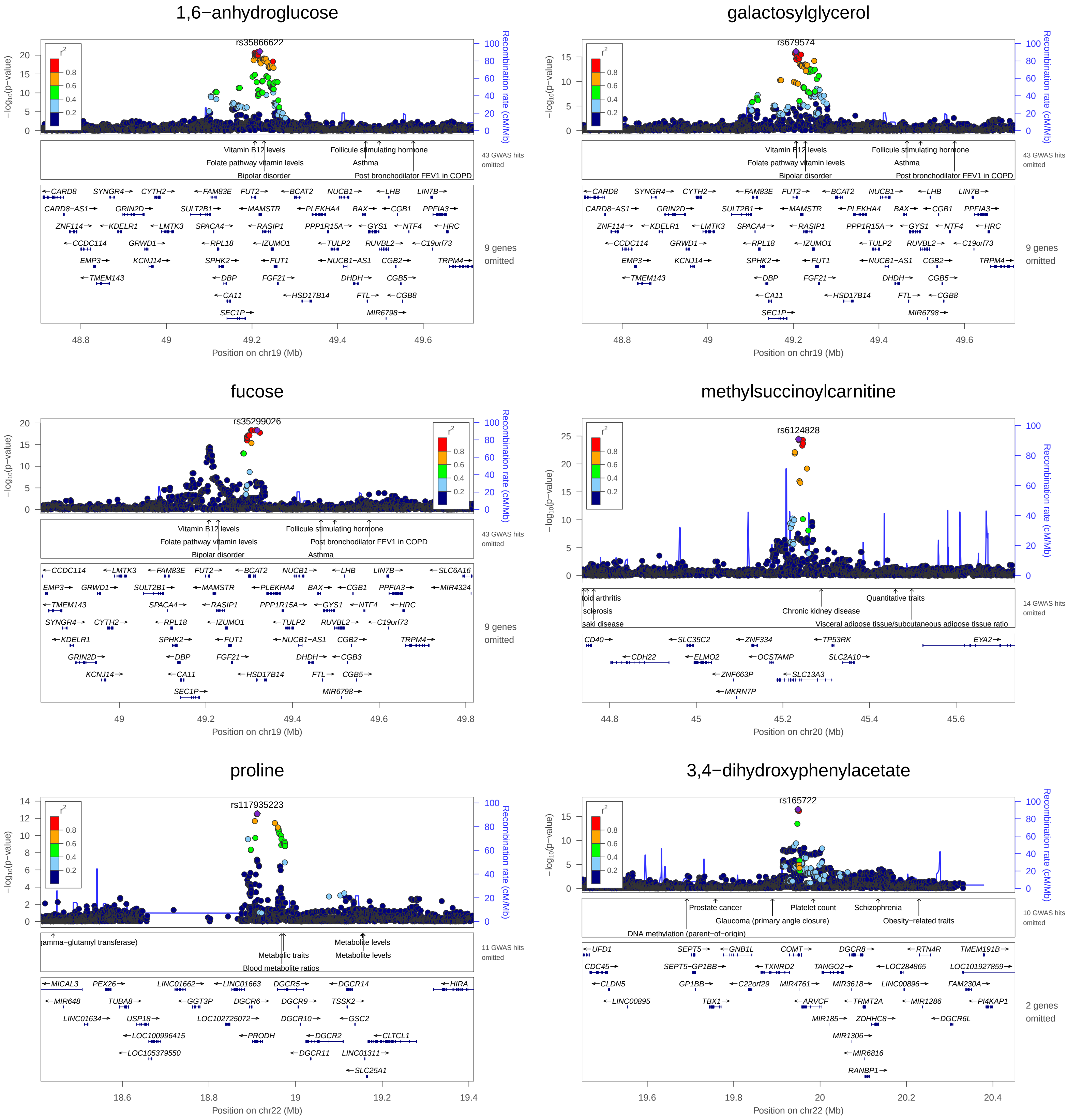
1,6−anhydroglucose
rs35866622
−log10(p−value)
Recombination rate (cM/Mb)
Vitamin B12 levels
Folate pathway vitamin levels
Bipolar disorder
Follicle stimulating hormone
Asthma
Post bronchodilator FEV1 in COPD
43 GWAS hits omitted
CARD8 SYNGR4 CYTH2 FAM83E FUT2 BCAT2 NUCB1 LHB LIN7B
CARD8−AS1 GRIN2D SULT2B1 MAMSTR PLEKHA4 BAX CGB1 PPFIA3
ZNF114 KDELR1 LMTK3 SPACA4 RASIP1 PPP1R15A GYS1 NTF4 HRC
CCDC114 GRWD1 RPL18 IZUMO1 TULP2 RUVBL2 C19orf73
EMP3 KCNJ14 SPHK2 FUT1 NUCB1−AS1 CGB2 TRPM4
TMEM143 DBP FGF21 DHDH CGB5
CA11 HSD17B14 FTL CGB8
SEC1P MIR6798
9 genes omitted
Position on chr19 (Mb)
galactosylglycerol
rs679574
−log10(p−value)
Recombination rate (cM/Mb)
43 GWAS hits omitted
9 genes omitted
Position on chr19 (Mb)
fucose
rs35299026
CCDC114 LMTK3 FAM83E FUT2 BCAT2 NUCB1 LHB LIN7B SLC6A16
EMP3 GRWD1 SULT2B1 MAMSTR PLEKHA4 BAX CGB1 PPFIA3 MIR4324
TMEM143 SPACA4 RASIP1 PPP1R15A GYS1 NTF4 HRC
SYNGR4 CYTH2 RPL18 IZUMO1 TULP2 RUVBL2 C19orf73
KDELR1 SPHK2 FUT1 NUCB1−AS1 CGB2 TRPM4
GRIN2D DBP FGF21 DHDH CGB3
KCNJ14 CA11 HSD17B14 FTL CGB5
SEC1P MIR6798
43 GWAS hits omitted
9 genes omitted
Position on chr19 (Mb)
methylsuccinoylcarnitine
rs6124828
toid arthritis
sclerosis
saki disease
Chronic kidney disease
Quantitative traits
Visceral adipose tissue/subcutaneous adipose tissue ratio
14 GWAS hits omitted
CD40 SLC35C2 ZNF334 TP53RK EYA2
CDH22 ELMO2 OCSTAMP SLC2A10
ZNF663P SLC13A3
MKRN7P
Position on chr20 (Mb)
proline
rs117935223
gamma−glutamyl transferase)
Metabolic traits
Blood metabolite ratios
Metabolite levels
Metabolite levels
11 GWAS hits omitted
MICAL3 PEX26 LINC01662 LINC01663 DGCR5 DGCR14 HIRA
MIR648 TUBA8 GGT3P DGCR6 DGCR9 TSSK2
LINC01634 USP18 LOC102725072 DGCR10 GSC2
LOC100996415 PRODH DGCR2 CLTCL1
LOC105379550 DGCR11 LINC01311
SLC25A1
Position on chr22 (Mb)
3,4−dihydroxyphenylacetate
rs165722
Prostate cancer
Glaucoma (primary angle closure)
DNA methylation (parent−of−origin)
Platelet count
Schizophrenia
Obesity−related traits
10 GWAS hits omitted
UFD1 SEPT5 GNB1L COMT DGCR8 RTN4R TMEM191B
CDC45 SEPT5−GP1BB TXNRD2 TANGO2 LOC284865 LOC101927859
CLDN5 GP1BB C22orf29 MIR4761 MIR3618 LINC00896 FAM230A
LINC00895 TBX1 ARVCF TRMT2A MIR1286 PI4KAP1
MIR185 ZDHHC8 DGCR6L
MIR1306
MIR6816
RANBP1
2 genes omitted
Position on chr22 (Mb)

2−O−methylascorbic acid

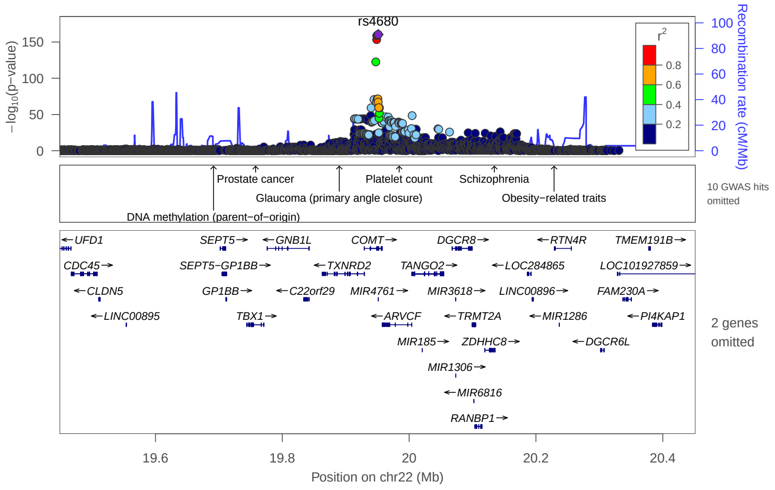

X − 12707

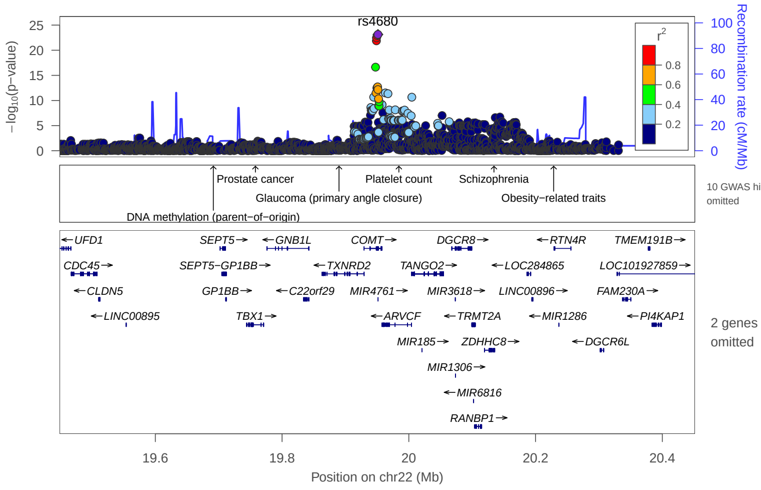

X − 12708

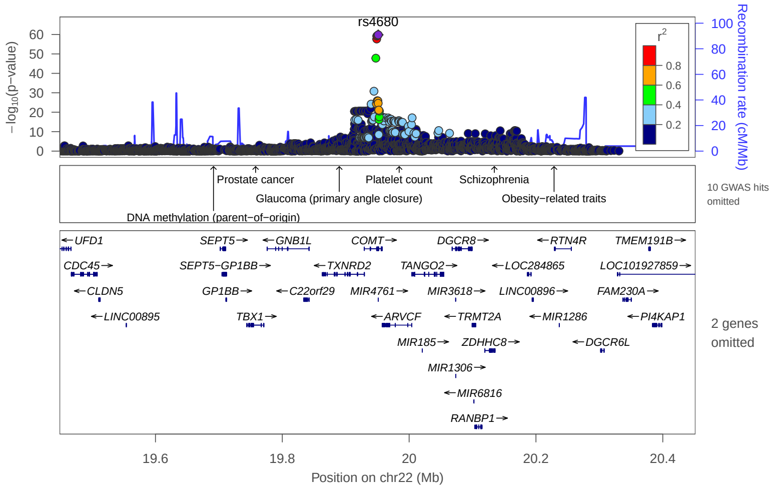

X − 12713

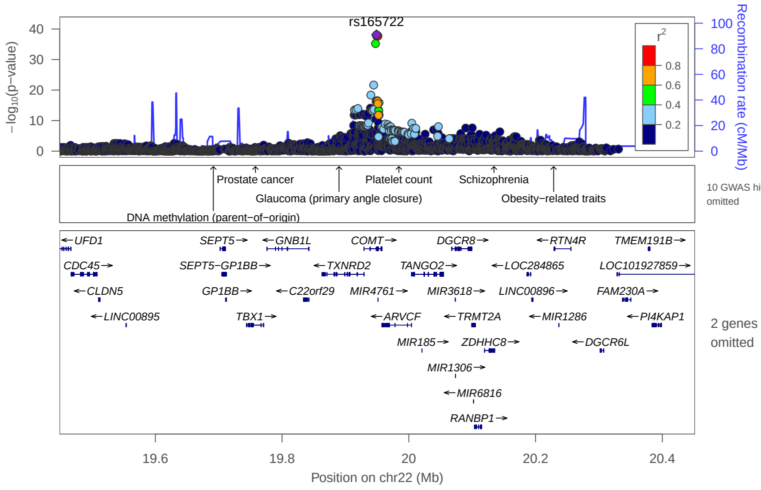

X − 17342

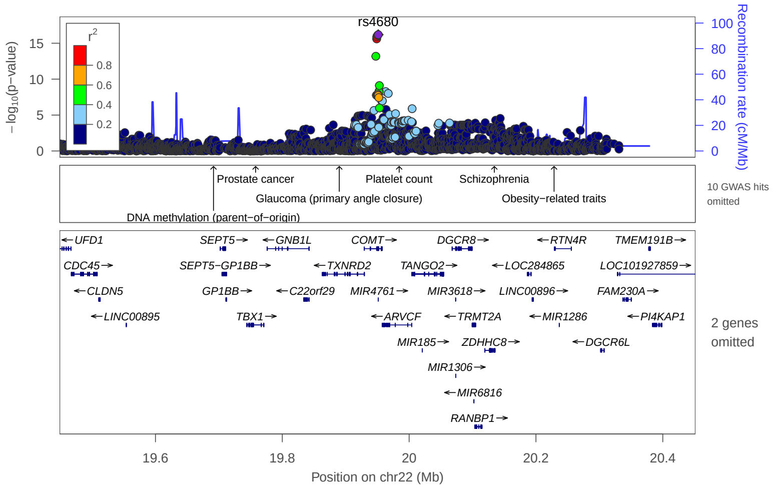

glucuronide of piperine metabolite C17H21NO3 (3)*

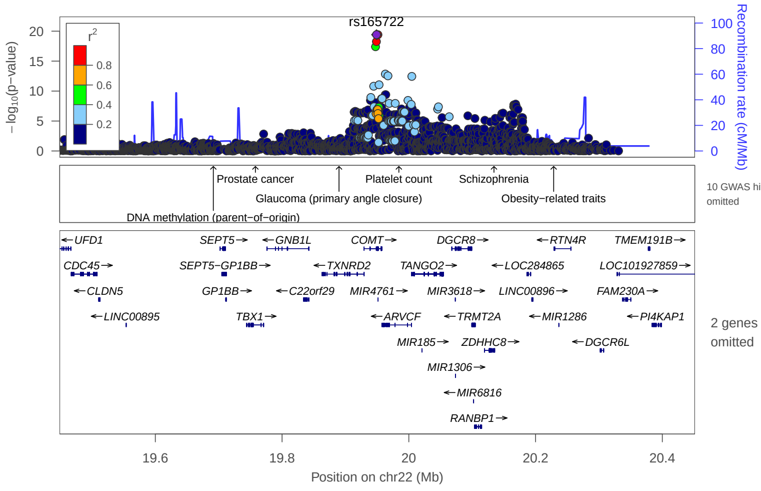

glucuronide of piperine metabolite C17H21NO3 (4)*

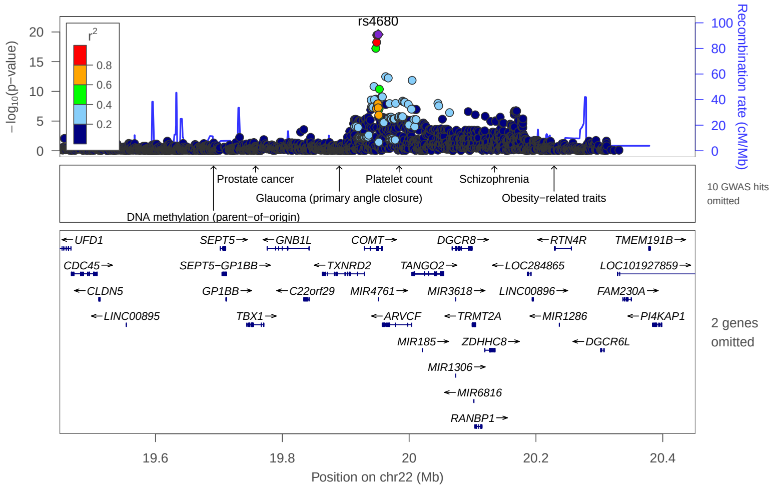

glucuronide of piperine metabolite C17H21NO3 (5)*

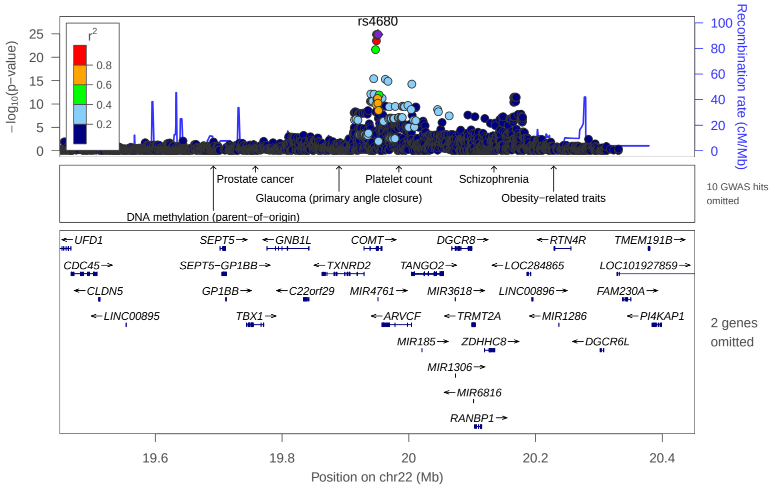

3−hydroxyindolin−2−one sulfate

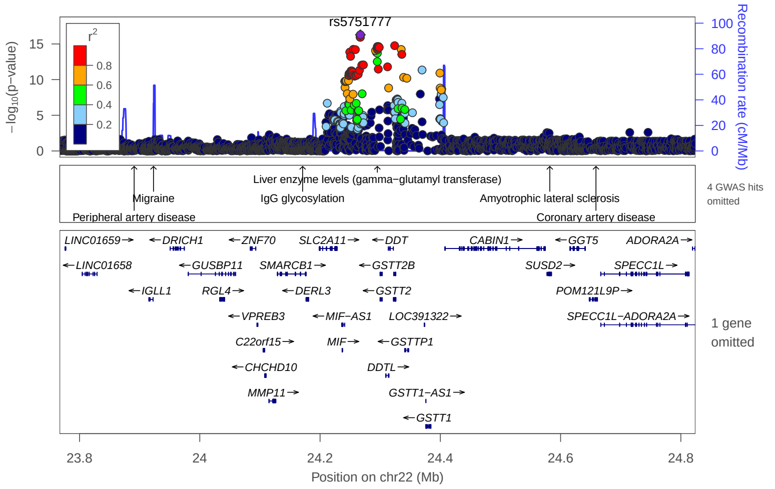

3−formylindole

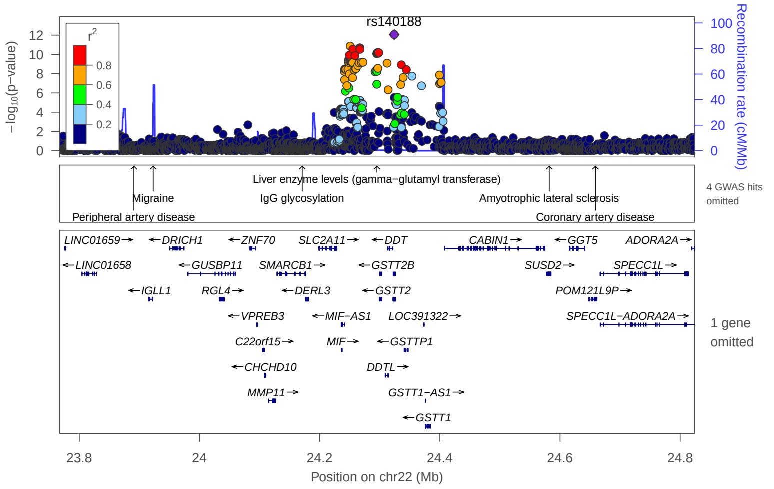

glycerophosphoethanolamine

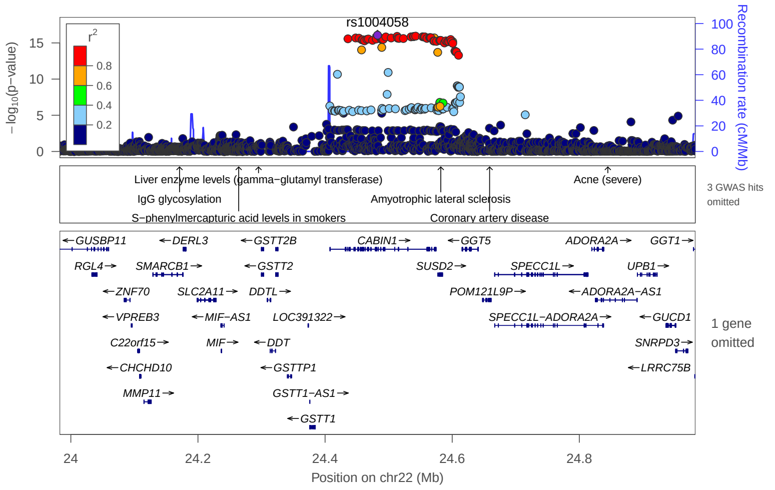

glycerophosphoserine*

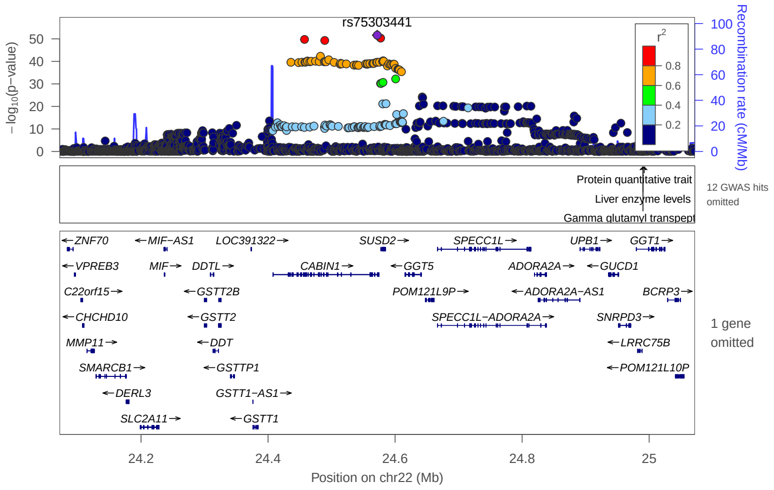

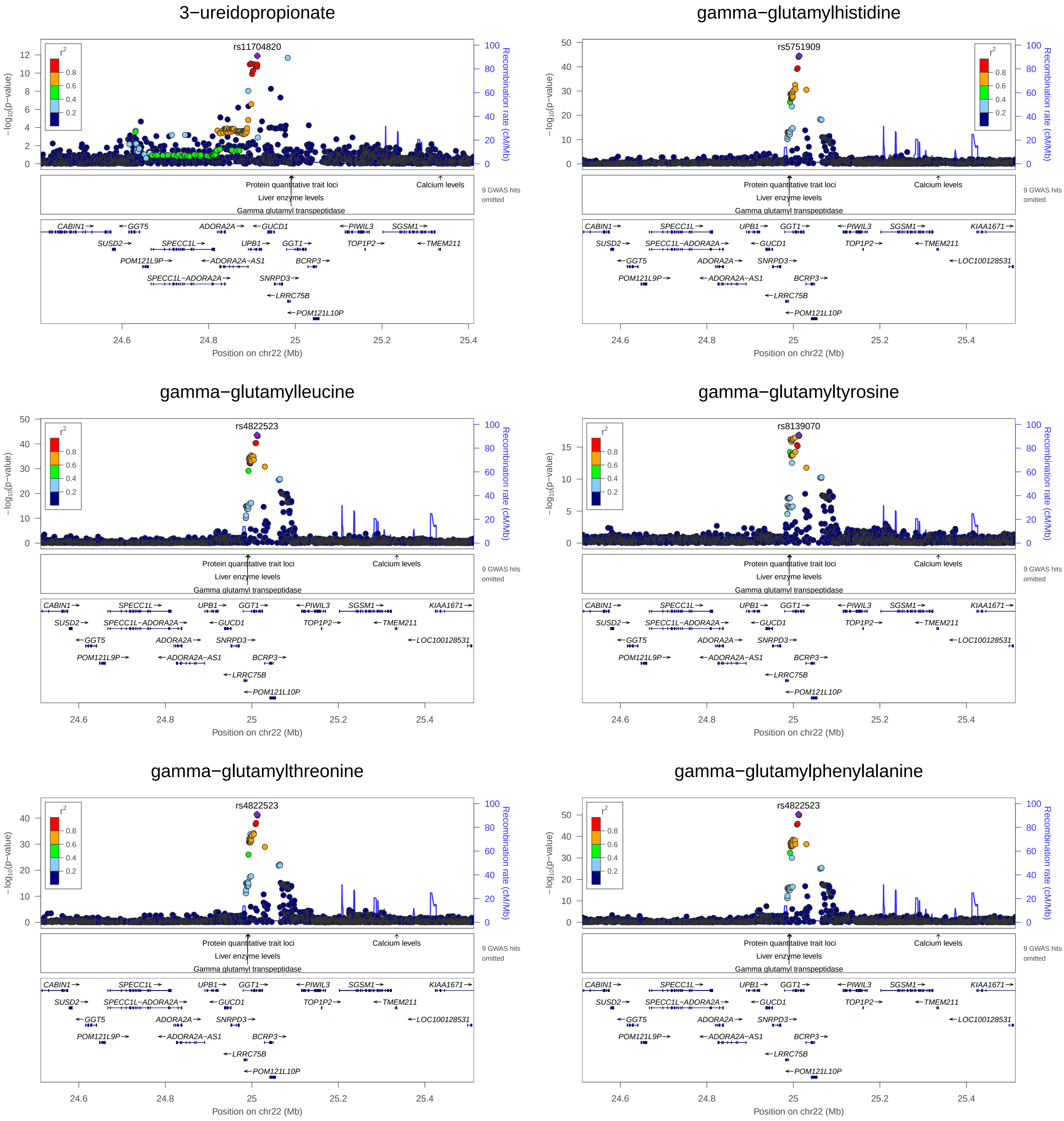

3−ureidopropionate
rs11704820
gamma−glutamylhistidine
rs5751909
gamma−glutamylleucine
rs4822523
gamma−glutamyltyrosine
rs8139070
gamma−glutamylthreonine
rs4822523
gamma−glutamylphenylalanine
rs4822523
−log10(p−value)
Recombination rate (cM/Mb)
Position on chr22 (Mb)
r2
0.8
0.6
0.4
0.2
Protein quantitative trait loci
Liver enzyme levels
Gamma glutamyl transpeptidase
Calcium levels
9 GWAS hits omitted
CABIN1
SUSD2
GGT5
POM121L9P
SPECC1L
SPECC1L−ADORA2A
ADORA2A
ADORA2A−AS1
UPB1
GUCD1
SNRPD3
LRRC75B
GGT1
POM121L10P
BCRP3
PIWIL3
TOP1P2
SGSM1
TMEM211
KIAA1671
LOC100128531

gamma−glutamyl−epsilon−lysine

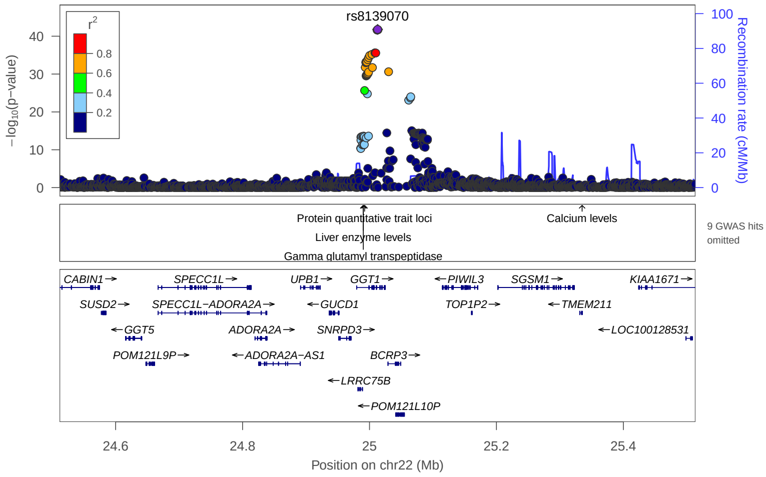

gamma−glutamylglycine

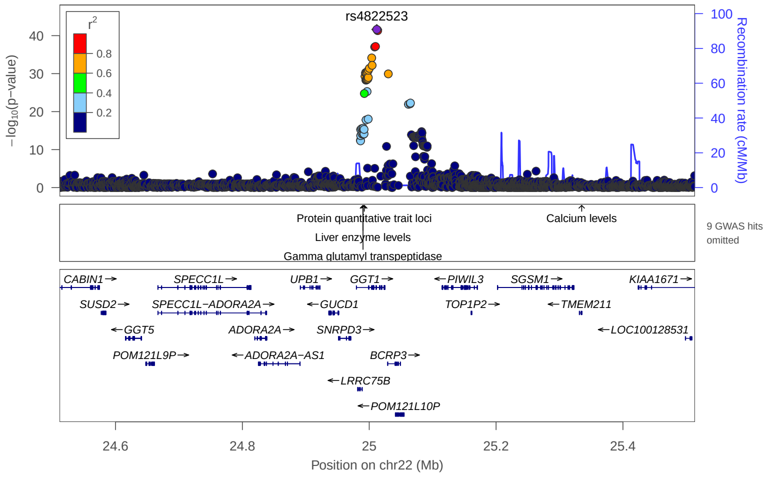

gamma−glutamylisoleucine*

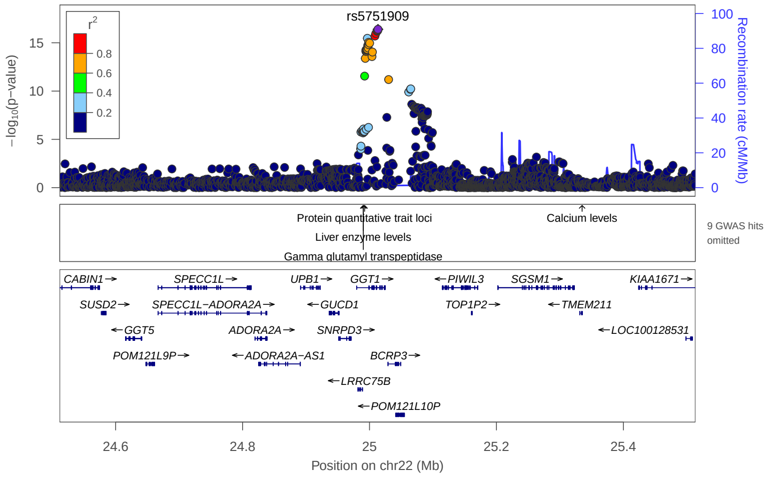

gamma−glutamylvaline

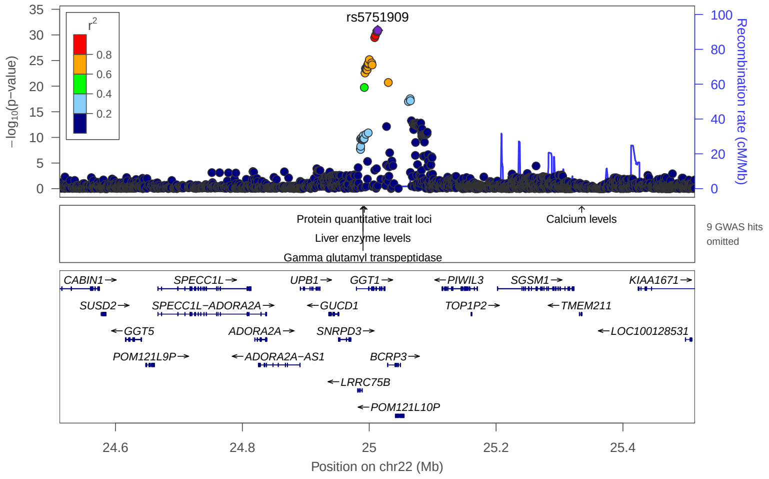

alpha−hydroxymetoprolol

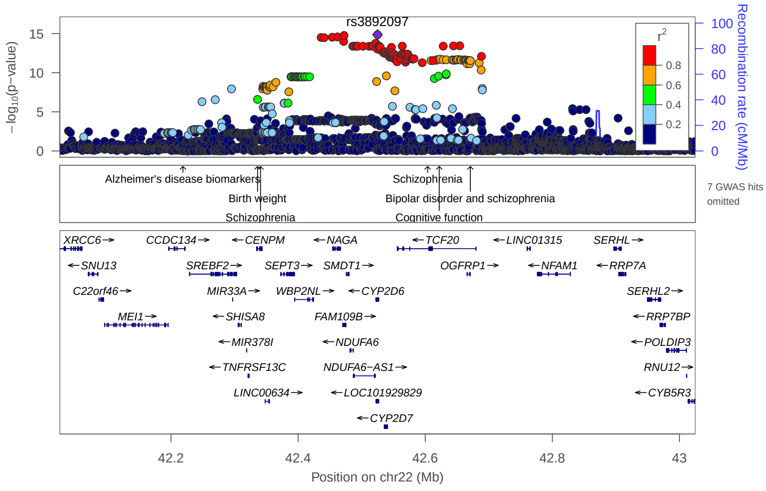

X − 22162

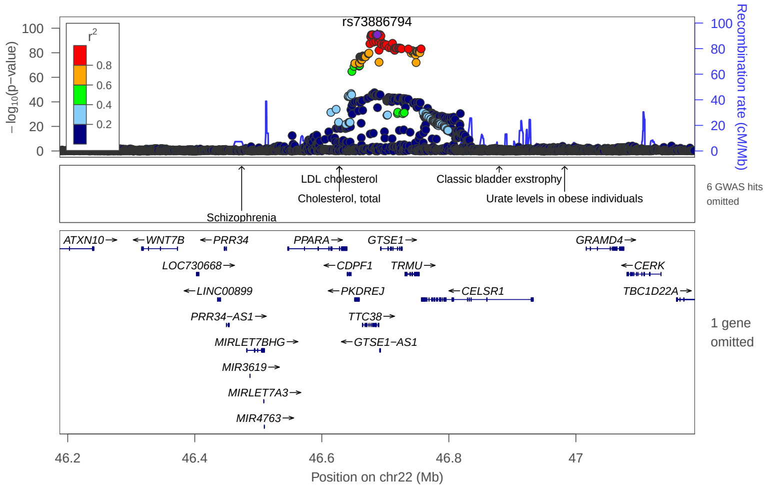

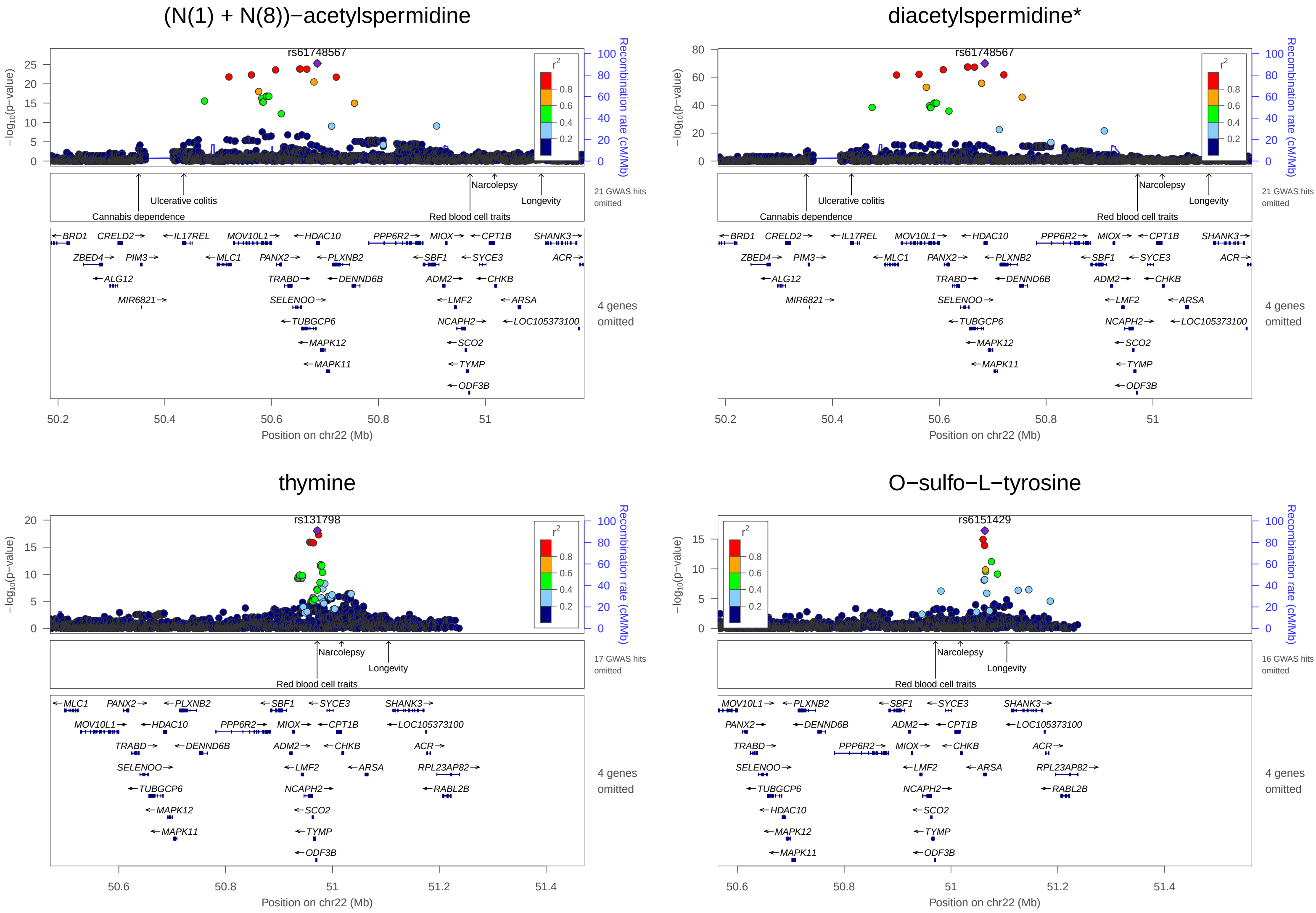
(N(1) + N(8))−acetylspermidine
rs61748567
diacetylspermidine*
rs61748567
thymine
rs131798
O−sulfo−L−tyrosine
rs6151429
−log10(p−value)
Recombination rate (cM/Mb)
r2
0.8
0.6
0.4
0.2
Cannabis dependence
Ulcerative colitis
Narcolepsy
Red blood cell traits
Longevity
21 GWAS hits omitted
17 GWAS hits omitted
16 GWAS hits omitted
4 genes omitted
BRD1
CRELD2
IL17REL
MOV10L1
HDAC10
PPP6R2
MIOX
CPT1B
SHANK3
ZBED4
PIM3
MLC1
PANX2
PLXNB2
SBF1
SYCE3
ACR
ALG12
TRABD
DENND6B
ADM2
CHKB
MIR6821
SELENOO
LMF2
ARSA
TUBGCP6
NCAPH2
LOC105373100
MAPK12
SCO2
MAPK11
TYMP
ODF3B
RPL23AP82
RABL2B
Position on chr22 (Mb)
